# Supplementary material for: Chemoselectivity change in catalytic hydrogenolysis enabling urea-reduction to formamide/amine over more reactive carbonyl compounds
Source: Nat Commun. 2023 Jun 12;14:3279. doi: 10.1038/s41467-023-38997-2 (PMC10261097; doi:10.1038/s41467-023-38997-2)
Supplement: Supplementary file 1 — Supplementary Information [file 41467_2023_38997_MOESM1_ESM.pdf]

Supplementary Information for

Chemoselectivity Change in Catalytic Hydrogenolysis enabling Urea-reduction to Formamide/Amine over More Reactive Carbonyl Compounds

Takanori Iwasaki, Kazuki Tsuge, Naoki Naito, and Kyoko Nozaki

Correspondence to: Takanori Iwasaki, iwasaki@chembio.t.u-tokyo.ac.jp; Kyoko Nozaki, nozaki@chembio.t.u-tokyo.ac.jp

This PDF file includes:

Materials and Methods
Supplementary Text
Supplementary Figs 1 to 114
Supplementary Tabs 1 to 30

Table of contents

1. Supplementary Text **S5**
1-1. Condition screening S5
1-2. Synthesis of cationic Ir complexes **23** and **24** and their catalytic activity S7
1-3. X-ray crystallography of metal complexes S8
1-4. Initial assessment of functional group tolerance and hydrogenolysis limitations S10
1-5. Possible reaction pathways S12
1-6. Observation of isocyanate as an intermediate S15
1-7. Hydrogenation of formamide and isocyanate, and competitive reactions S17
1-8. NBO analysis S19
1-9. Kinetic studies S20
1-10. Observation of Ir hydride species S22
1-11. Regioselectivity of unsymmetric ureas S23
1-12. Degradation of polyurea resins and their chemical recycling S25
1-13. Condition screening for the hydrogenolysis of polyurea **17** using Ir catalyst **4** S28

2. General Information **S29**
2-1. Manipulation S29
2-2. Instrumentation S29
2-3. Materials S29

3. X-ray Crystallography **S31**

4. Kinetic Studies of Catalytic Hydrogenolysis **S32**
4-1. Kinetic profile for the hydrogenolysis of 1,3-diphenylurea (**1a**) using **4** under Condition A S32
4-2. Determination of rate order of urea **1a** under Condition A S34
4-3. Determination of rate order of H_2 pressure under Condition A S38
4-4. Determination of rate order of catalyst **4** under Condition A S42
4-5. Determination of rate order of KO^{t}Bu S47

5. Synthesis and Characterization of Urea Derivatives **S52**
5-1. Synthesis of symmetric urea derivatives S52
5-2. Synthesis of unsymmetric urea derivatives S54
5-3. Synthesis of polyurea **17** S55
5-4. Synthesis of authentic samples of products in the hydrogenolysis of polyurea **17** S56

6. Synthesis and Characterization of Catalysts **S57**

7. General Procedures **S76**
7-1. Catalyst screening (Fig. 2) S76
7-2. Condition A: the hydrogenolysis of urea derivatives using Ir catalyst **4** S77
7-3. Condition B: the hydrogenolysis of urea derivatives using Ir catalyst **4** in the presence of KO^{t}Bu S78
7-4. Competitive reactions of urea **1a** with ester **13**, carbamate **14**, ketone **25**, or formamide **2b** (Fig 3b and Supplementary Figs 5 and 12) S78

7-5. Hydrogenolysis of ester **13**, carbamate **14**, or formamide **2b** (Fig 3c and Supplementary Fig 11) S82
7-6. Gram-scale hydrogenolysis (Fig. 3e) S83
7-7. Reducing catalyst loading (Fig. 3f) S83
7-8. Hydrogenolysis of polyurea **17** (Fig. 5 and Supplementary Tab 5) S83
7-9. Optimization of reaction conditions using Ir catalyst **4** (Supplementary Tab 1) S84
7-10. Optimization of reaction conditions using Ir catalyst **4** (Supplementary Tab 2) S84
7-11. Hydrogenolysis of urea **1a** using a tautomeric mixture of **23** and **24** (Supplementary Fig 2) S84
7-12. Competitive reaction of urea **1a** with arenes having various functionalities (Supplementary Fig 4) S85
7-13. Thermal decomposition of urea **1a** in Kugelrohr apparatus (Supplementary Fig 9) S85
7-14. Thermal decomposition of urea **1h** in the presence of phenyl isocyanate (**27a**) (Supplementary Fig 10) S89
7-15. Hydrogenation of phenyl isocyanate (**27a**) (Supplementary Fig 13) S89
7-16. Competitive reaction of phenyl isocyanate (**27a**) with formamide **2b** (Supplementary Fig 14) S90
7-17. Competitive reaction of urea **1a** with aniline (**3a**) (Supplementary Fig 17) S90
7-18. The reaction of Ir complex **4** with H_2 (Supplementary Fig 18) S90
7-19. Formyl group transfer under the neutral conditions (Supplementary Figs 19 and 20) S95
7-20. Formyl group transfer under the basic conditions (Supplementary Figs 19 and 20) S95

8. Characterization of Products S96
8-1. The hydrogenolysis of 1,3-diphenylurea (**1a**) (Condition A) S96
8-2. The hydrogenolysis of 1,3-diphenylurea (**1a**) (Condition B) S98
8-3. The hydrogenolysis of 1,3-bis(4-fluorophenyl)urea (**1b**) S99
8-4. The hydrogenolysis of 1,3-bis(4-chlorophenyl)urea (**1c**) S101
8-5. The hydrogenolysis of 1,3-bis(3-chlorophenyl)urea (**1d**) S103
8-6. The hydrogenolysis of 1,3-bis(2-chlorophenyl)urea (**1e**) S105
8-7. The hydrogenolysis of 1,3-bis(4-bromophenyl)urea (**1f**) S107
8-8. The hydrogenolysis of 1,3-bis(4-*tert*-butylphenyl)urea (**1g**) S109
8-9. The hydrogenolysis of 1,3-bis(4-methoxyphenyl)urea (**1h**) S111
8-10. The hydrogenolysis of 1,3-bis(4-dimethylaminophenyl)urea (**1i**) S113
8-11. The hydrogenolysis of diethyl 4,4’-(carbonylbis(azanediyl))dibenzoate (**1j**) S115
8-12. The hydrogenolysis of 4,4’-(carbonylbis(azanediyl))-bis(*N*,*N*-dipropylbenzamide) (**1k**) S117
8-13. The hydrogenolysis of 1,3-bis(4-cyanophenyl)urea (**1*l***) S119
8-14. The hydrogenolysis of 1,3-dibenzylurea (**1m**) S121
8-15. The hydrogenolysis of 1,3-dicyclohexylurea (**1n**) S123
8-16. The hydrogenolysis of 1,3-diphenyl-1-methylurea (**1ao**) (Condition A) S125
8-17. The hydrogenolysis of 1,3-diphenyl-1-methylurea (**1ao**) (Condition B) S127
8-18. The hydrogenolysis of 1-(4-chlorophenyl)-1-methyl-3-phenylurea (**1ap**) S128
8-19. The hydrogenolysis of 1,3-bis(4-chlorophenyl)-1-methylurea (**1cp**) S129

8-20. The hydrogenolysis of 3-(4-butylphenyl)-1-(4-chlorophenyl)-1-methylurea (**1qp**) S130
8-21. The hydrogenolysis of 3-(4-chlorophenyl)-1-(4-methoxylphenyl)-1-methylurea (**1cr**) S131
8-22. The hydrogenolysis of 3-(4-butylphenyl)-1-(4-methoxylphenyl)-1-methylurea (**1qr**) S132
8-23. The hydrogenolysis of *N*-phenylmorpholine-4-carboxamide (**1as**) (Condition A) S133
8-24. The hydrogenolysis of *N*-phenylmorpholine-4-carboxamide (**1as**) (Condition B) S134
8-25. The hydrogenolysis of polyurea **17** (Fig. 5) S135

9. Copies of Spectra for Isolated Products S138

10. Computational Studies S244

11. References S247

1. Supplementary Text

1-1. Condition screening

The reaction conditions were optimized using 3 mol% of catalyst **4** (Supplementary Tab 1), and the result obtained using **4** in toluene (Fig. 2 in the main text) is shown in entry 1. When the reaction was conducted in THF, the yield of **2a** and **3a** decreased to 52% and 54%, respectively, but the selectivity remained the same (entry 2). The use of 1,4-dioxane lowered the yields with a negligible effect on the selectivity (entry 3). Almost no conversion was observed when DMF or cyclohexane was used (entries 4 and 5). In the latter case, **1a** remained undissolved during the reaction. Subsequently, the reaction temperature was investigated applying toluene as the solvent. At 110 °C, the reaction was sluggish, resulting in <5% conversion (entry 6). When the reaction temperature was increased, full conversion of **1a** and a slightly lower selectivity (94%) were observed due to the over-reduction of **2a** to give **3a** in 108% yield based on the amount of urea **1a** (entry 7). Therefore, the optimal reaction temperature was determined to be 130 °C. Further investigation regarding the H_2 pressure revealed that an increase from 1 to 3 MPa did not improve the conversion (compare entries 8 and 9), but a decrease to 0.5 MPa resulted in lower conversion (entry10). Therefore, the optimal H_2 pressure was determined to be 1 MPa.

Supplementary Tab 1.

Condition screening for the hydrogenolysis of urea **1a** using Ir catalyst **4***.

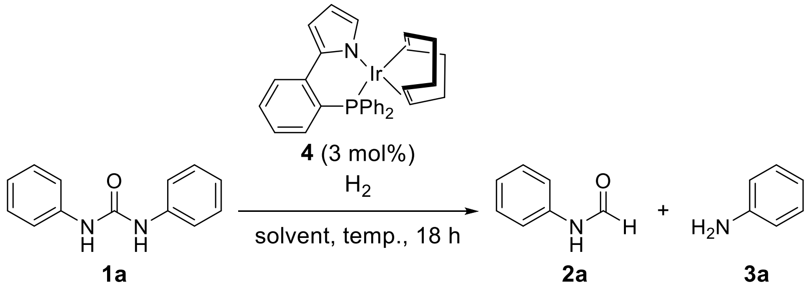

entry	solvent	H_2 (MPa)	temp. (°C)	conv. (%)	**2a** (%)	**3a** (%)	selectivity (%)
1	toluene	2	130	85	82	83	99
2	THF	2	130	57	52	54	98
3	1,4-dioxane	2	130	26	24	29	91
4	DMF	2	130	6	n.d.	9	—
5	cyclohexane	2	130	5	6	6	100
6	toluene	2	110	<5	<5	<5	—
7	toluene	2	150	>99	95	108	94
8	toluene	3	130	71	68	70	99
9	toluene	1	130	82	77	80	98
10	toluene	0.5	130	67	64	66	98

*, Reaction conditions: **1a** (0.17 mmol), **4** (5 µmol, 3 mol%), solvent (2 mL), H_2 for 18 h. The conversion and yields were determined by ^{1}H NMR spectroscopy with the internal standard (dibromomethane). Selectivity value = yield of **2a** / {(yield of **2a** + yield of **3a**) / 2} × 100 (%).

Further optimization of the reaction condition, including the reaction scale and additives, is presented in Supplementary Tab 2. When the amount of substrate **1a** was increased from 0.17 mmol to 0.50 mmol in the same amount of toluene, the conversion decreased to 50% due to the low solubility of **1a** (entry 1). Under high-concentration conditions (0.25 M — 0.50 mmol of substrate in 2 mL of solvent), conversion was higher in THF (71%, entry 2) than in toluene, reaching 85% when the solvent volume was increased to 3 mL (0.17 M) and the reaction time prolonged to 48 h (entry 3). When 1 equiv of H_2O was added to the conditions shown in entry 3, 42% of **2a** and a significant amount of **3a** (95%) were obtained, with a slightly lower conversion of 71% (entry 4). The formation of the significant amount of **3a** by adding H_2O is probably due to the hydrolysis of **1a** and/or phenyl isocyanate (**27a**) and subsequent decarboxylation. While the addition of a Brønsted acid, $HNTf_2$ (*31*), shut down the hydrogenolysis reaction (entry 5, see also Section 1-2), that of KO^{t}Bu (10 mol%) significantly improved the conversion to >99% even at a lower catalyst loading of 1 mol% (entry 6). Further screening of base additives at a shorter reaction time (4 h) revealed that KO^{t}Bu provided the best results, and replacement of the K cation by Li or Na resulted in lower conversions (entries 7–9).

Supplementary Tab 2.

Additive effect on the hydrogenolysis of urea **1a** using Ir catalyst **4***.

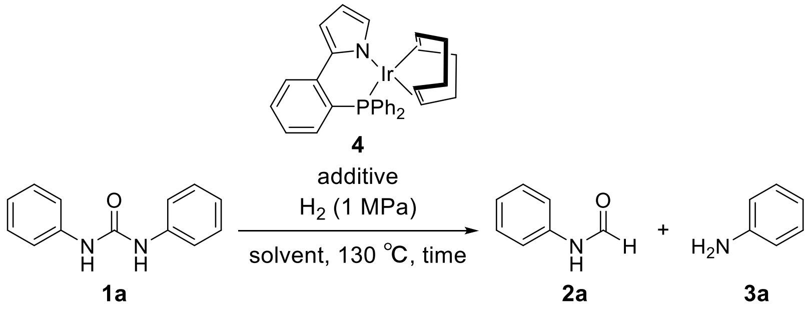

entry	**4** (mol%)	additive (mol%)	solvent	time (h)	conv. (%)	**2a** (%)	**3a** (%)	selectivity (%)
1	3	—	toluene	18	50	41	49	91
2	3	—	THF	18	71	69	72	98
3†	3	—	THF	48	85	80	82	99
4†	3	H_2O (100)	THF	48	71	42	95	61
5	1	$HNTf_2$ (10)	toluene	18	9	n.d.	15	—
6	1	KO^{t}Bu (10)	toluene	18	>99	83	114	84
7	1	KO^{t}Bu (10)	toluene	4	77	66	85	87
8	1	NaO^{t}Bu (10)	toluene	4	25	25	28	94
9	1	LiO^{t}Bu (10)	toluene	4	16	8	18	62

*, Reaction conditions: **1a** (0.50 mmol), **4**, additive, solvent (2 mL), H_2 (1 MPa) at 130 °C. The conversion and yields were determined by ^{1}H NMR spectroscopy with the internal standard (dibromomethane). Selectivity value = yield of **2a** / {(yield of **2a** + yield of **3a**) / 2} × 100 (%); †, THF (3 mL).

1-2. Synthesis of cationic Ir complexes **23** and **24** and their catalytic activity

As an analog of the neutral Ir complex **4**, a cationic complex bearing ligand **5** was synthesized via the reaction of **5** with the cationic Ir complex, $[Ir(cod)_2]BAr^F_4$ (BAr^F_4 = tetrakis[3,5-bis(trifluoromethyl)phenyl]borate). The reaction afforded a tautomeric mixture of cationic complexes **23** and **24**, which were protonated at the 5-position of the pyrrole ring and the nitrogen atom, respectively, and their ratio varied depending on the solvent used. In THF, complex **24** was mainly observed (**23**:**24** = 20:80), whereas in benzene, complex **23** was predominantly detected (**23**:**24** = 89:11) (Supplementary Fig 1). Single crystals of **23** were obtained and the structure was elucidated using X-ray crystallography (Section 1-3). In the ^1H NMR spectrum of **24**, the NH signal appeared at 11.81 ppm, being significantly down-field shifted from that of parent ligand **5** (10.09 ppm). This suggests π-coordination of the pyrrole moiety to the cationic Ir center (Supplementary Fig 68).

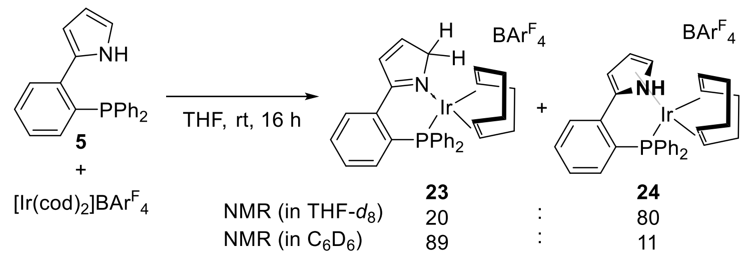

Supplementary Fig 1.

Complexation of ligand **5** with cationic Ir complex, $[Ir(cod)_2]BAr^F_4$.

Because **23** and **24** were inseparable due to their interconversion in solution, we conducted the hydrogenolysis of **1a** using a mixture of the two complexes in THF and toluene (Supplementary Fig 2). In both cases, the cationic complexes exhibited low catalytic activities and chemoselectivities. These results are consistent with the negative effects of the acidic additive $HNTf_2$ (Supplementary Tab 2, entry 4).

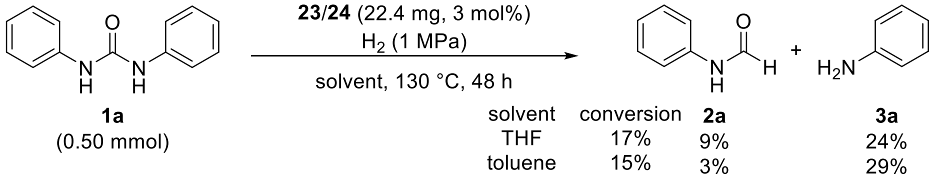

Supplementary Fig 2.

Hydrogenolysis of **1a** using a tautomeric mixture of **23** and **24**.

1-3. X-ray crystallography of metal complexes

The molecular structures of complexes **4**, **7**, **9**, and **23** used in this study as catalysts were unambiguously determined using X-ray diffraction analysis, as shown in Supplementary Fig 3 (see Section 3 for details).

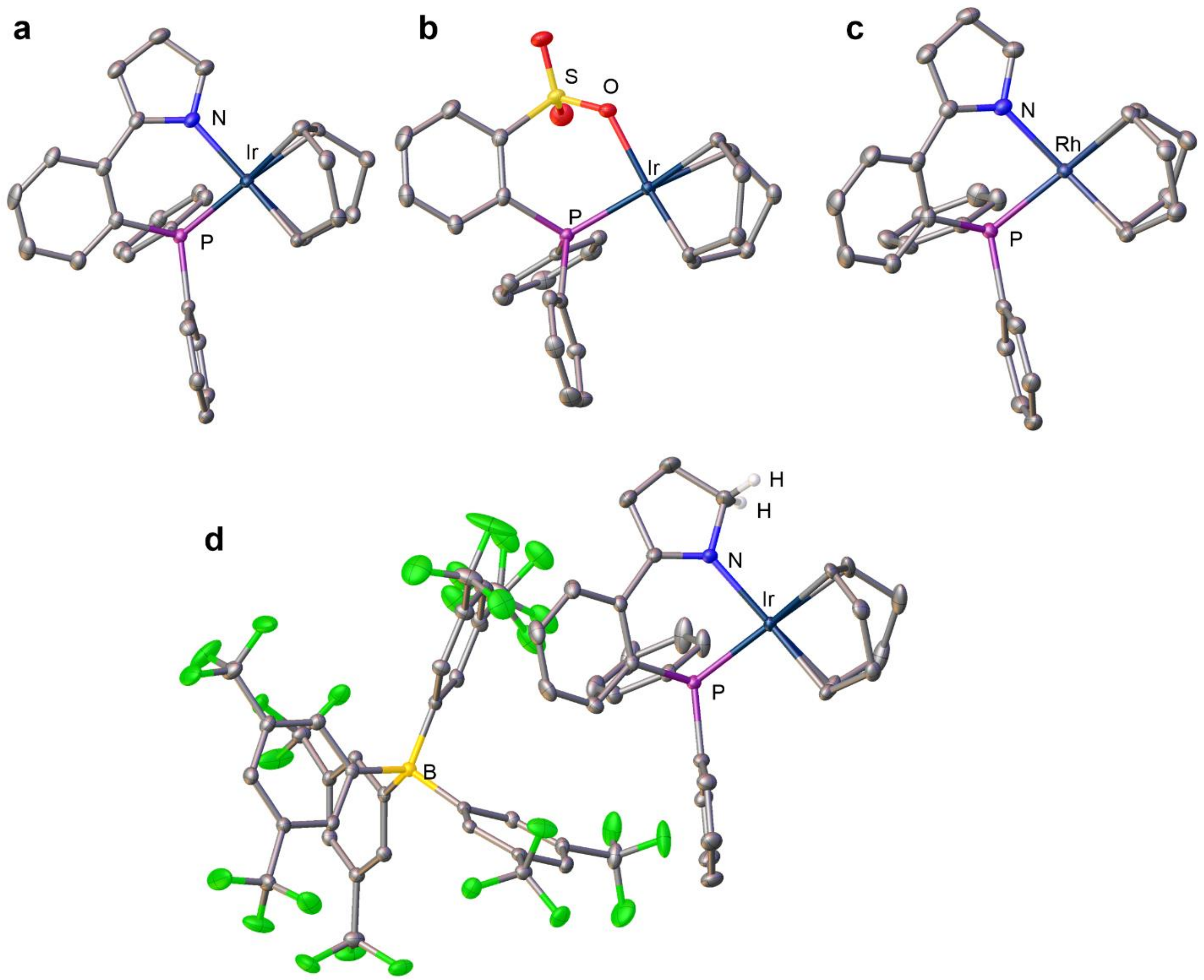

Supplementary Fig 3.

ORTEP drawing of complexes with thermal ellipsoids at the 50% probability level. H atoms and disordered molecules are omitted for clarity. (**a**) Molecular structure of Ir complex **4**. (**b**) Molecular structure of Ir complex **7**. (**c**) Molecular structure of Rh complex **9**. (**d**) Molecular structure of cationic Ir complex **23**. Hydrogen atoms on the sp^3-hybridized carbon in the pyrrole ring are shown.

The bond lengths and bond angles are listed in Supplementary Tab 3. In the crystal structure of Ir complex **4**, the Ir center is coordinated by ligand **5** and COD in square planar geometry, with the sum of the angles around Ir being 360.0°. The bond lengths in the pyrrole ring showed non-bond alternation, indicating that the pyrrolido moiety in complex **4** maintained its aromaticity. The COD C=C bond was longer (1.417 Å) at the pyrrolido *trans*-position than at the *cis*-position (1.386 Å), implying that the π-accepting capacity of the pyrrolido moiety was lower than that of the diphenylphosphino moiety. Analogous neutral complexes bearing a sulfonate coordinating site (**7**) and Rh as the metal center (**9**) presented similar geometries.

The crystal structure of complex **23** consists of cationic Ir and the borate anion. The Ir center is coordinated by ligand **5** and COD in square planar geometry. Bond alternation was observed in the pyrrole ring, as evidenced by shorter C2–C3 (1.331 Å) and C4–N (1.308 Å) bonds and longer N–C1 (1.468 Å), C1–C2 (1.483 Å), and C3–C4 (1.465 Å) bonds. Therefore, in complex **23**, C1 (the 5-position in pyrrole) was protonated, resulting in sp^3-hybridization, which is consistent with the appearance of the CH_2 proton signals at 4.96 ppm in its ^1H NMR spectrum (Supplementary Fig 68).

Supplementary Tab 3.

Selected bond lengths and angles of complexes **4**, **7**, **9**, and **23**.

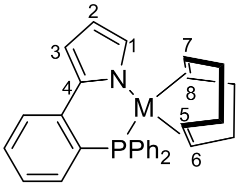

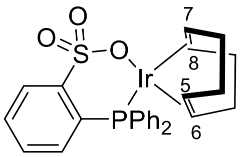

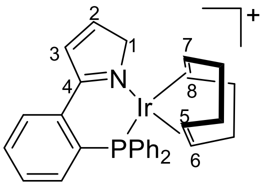

	**4**	**7**	**9**	**23**
M–N/O	2.064(3)	2.092(2)	2.0785(15)	2.0881(18)
M–P	2.2784(10)	2.2803(7)	2.2640(4)	2.2645(5)
N–C1	1.378(6)	—	1.368(2)	1.468(3)
C1–C2	1.381(6)	—	1.377(3)	1.483(3)
C2–C3	1.395(7)	—	1.403(3)	1.331(4)
C3–C4	1.393(6)	—	1.395(3)	1.465(3)
C4–N	1.393(5)	—	1.385(2)	1.308(3)
M–(C5–C6)	2.014	1.979	2.023	2.024
C5–C6	1.417(6)	1.423(4)	1.400(3)	1.409(3)
M–(C7–C8)	2.095	2.092	2.128	2.102
C7–C8	1.386(6)	1.385(4)	1.375(3)	1.384(4)
P–M–N/O	86.37(11)	92.13(6)	85.74(4)	85.60(5)
Sum around M*	360.0	360.0	360.0	360.0

*, Sum of angles P–M–N/O, P–M–(C5–C6 centroid), (C5–C6 centroid)–M–(C7–C8 centroid), and (C7–C8 centroid)–M–N/O.

1-4. Initial assessment of functional group tolerance and hydrogenolysis limitations

Prior to considering the hydrogenolysis of functionalized urea derivatives (Fig. 3a), functional group tolerance was initially investigated by adding a mixture of anisole, nitrobenzene, chlorobenzene, bromobenzene, and benzonitrile (0.10 mmol for each) to the hydrogenolysis reaction of **1a** (Supplementary Fig 4). GC analysis revealed that almost quantitative amounts of anisole (93%), chlorobenzene (>99%), bromobenzene (98%), and benzonitrile (>99%) were recovered. In contrast, nitrobenzene was completely consumed to afford aniline **3a** under Condition A.

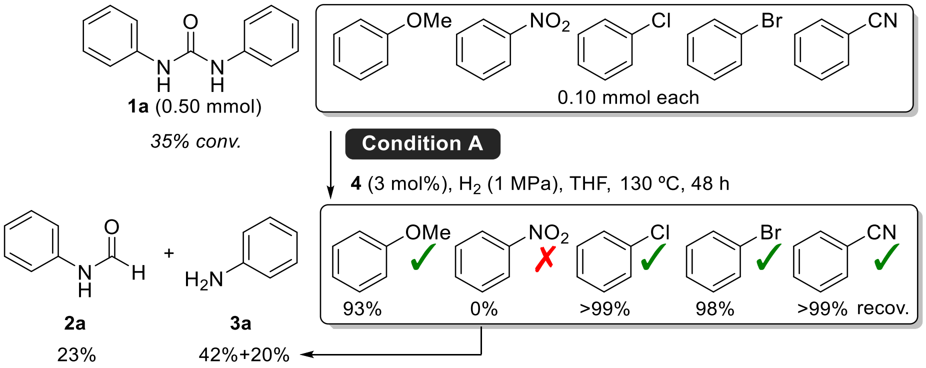

Supplementary Fig 4.

Competitive reaction with arenes having various functionalities.

Similar to the experiments shown in Fig. 3b, a competitive reaction between **1a** and ketone **25** was performed. In this case however, the ketone functionality was preferentially reduced to alcohol **26** (Supplementary Fig 5). Based on these experiments and the reactions shown in Fig. 3, ether, nitrile, ester, amide, and carbamate functionalities, as well as C–Cl and C–Br bonds are compatible with the hydrogenolysis reaction, but nitro and ketone functionalities are readily reduced.

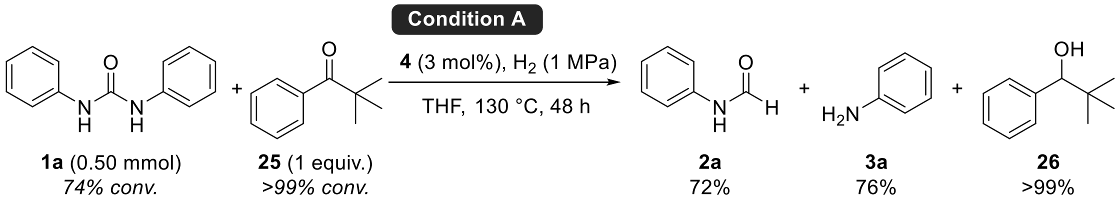

Supplementary Fig 5.

Competitive reaction with ketone **25**.

Because urea can accommodate up to four substituents on the two nitrogen atoms, tetrasubstituted urea **1o** was tested to better understand the scope and limitations of the present catalytic system (Supplementary Fig 6a). Although no conversion of **1o** was observed under Condition A, 16% of **1o** was reduced to the corresponding *N*-methylaniline (**3o**) in 22% yield under Condition B. As shown in Fig. 3, di- and tri-substituted urea derivatives are suitable for chemoselective hydrogenolysis, while the tetrasubstituted derivative displayed low reactivity and chemoselectivity. Another limitation of the present system is the low conversion of cyclic urea derivatives. For example, the hydrogenolysis of 2-imidazolidinone (**1t**) did not afford **2t** or **3t** under either Condition A or B (Supplementary Fig 6b).

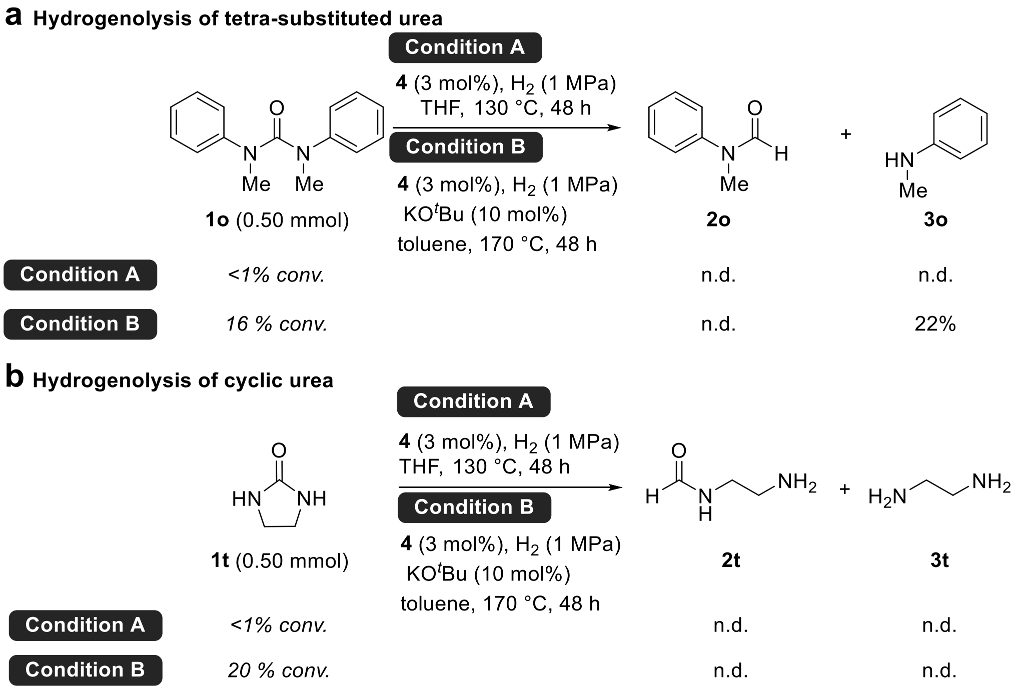

Supplementary Fig 6.

Substrate limitations. (**a**) Hydrogenolysis of *N*,*N*'-dimethyl-*N*,*N*'-diphenylurea (**1o**). (**b**) Hydrogenolysis of 2-imidazolidinone (**1t**).

1-5. Possible reaction pathways

As illustrated in Fig. 4, we postulated two possible reaction pathways to explain the unique chemoselectivity observed for the hydrogenolysis of ureas to formamides and amines based on literature reports and the mechanistic studies described below.

To discuss the mechanism in detail, two possible reaction pathways are shown in Supplementary Figs 7 and 8. In the *Metal-ligand cooperative pathway* (Supplementary Fig 7), Ir precatalyst **4** reacts with H_2 to generate catalytically active Ir hydride species **A** via reduction of the COD ancillary ligand. Further reaction with H_2 occurs via the heterolytic cleavage of H_2 by the Ir center and pyrrolido moiety to generate complex **B**. These hydride species are in equilibrium with the hydride-bridged oligomeric dormant species (Section 1-10). Protonation of the carbonyl oxygen by the acidic N–H bond in the pyrrole moiety favors the more basic carbonyl oxygen of ureas over that of formamides (**C**) (Sections 1-7 and 1-8). This discrimination is the origin of the unprecedented chemoselectivity observed in the present catalytic system. Further hydride transfer from the Ir center (**C'**) or concomitant proton and hydride transfer to the carbonyl C=O bond of urea (**C**) forms intermediate **D** and regenerates Ir complex **A**. Elimination of one amino group from intermediate **D** yields a formamide and an amine in a selective manner (Section 1-11).

In this catalytic cycle, the Ir center and pyrrole moiety cooperatively reduce the C=O bond of the urea moiety. Although such metal-ligand cooperation[1,2] has been well established for the catalytic reduction of carbonyl compounds, including urea derivatives, the proposed catalytic cycle involves a unique mechanism, namely, the N atom of the pyrrole ring participates directly in the heterolytic cleavage of H_2 (**A** to **B**) as well as the proton transfer step (**C** to **C'**) as a result of its coordination mode being switched between σ- and π-coordination. The formation of complex **24** comprising π-coordinated pyrrole (Section 1-2) suggests that the N–Ir bond participates in the heterolytic cleavage of H_2, and the resultant protonated pyrrole then acts as a Brønsted acid to cooperatively activate the urea moiety (**C** and **C'**). Protonation[3] of the N atom (**A** to **B**) is consistent with the finding that complex **6**, which contains a benzo-fused structure next to the N atom, exhibited comparable catalytic activity (Fig. 2). Moreover, N protonation is more plausible than that of the carbon at the pyrrole 5-position, as observed in the cationic complex **24** (Section 1-2).

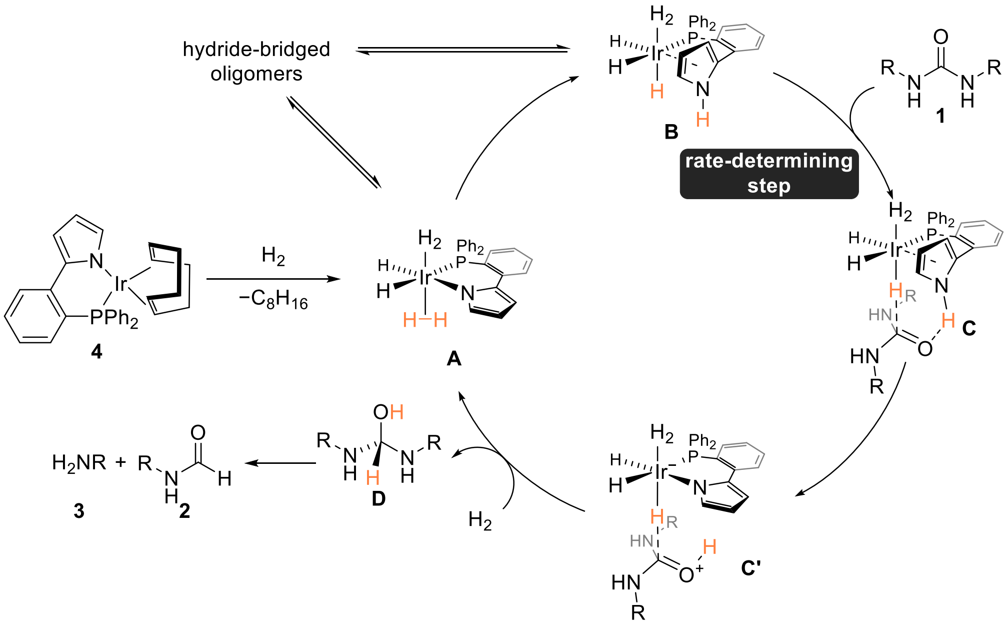

Supplementary Fig 7.

A proposed catalytic cycle: metal-ligand cooperative hydrogenolysis mechanism (*metal-ligand cooperative pathway*).

The *thermal decomposition pathway* is another possible reaction mechanism involving the thermal decomposition of urea **1** to isocyanate **F** via zwitterion **E**, as shown in Supplementary Fig 8. Ir precatalyst **4** reacts with H_2 to generate catalytically active Ir hydride species **G** via reduction of the COD ancillary ligand (Section 1-10). Intermediate **G** selectively reacts with isocyanate **F** generated *in situ* (Section 1-6) because the electrophilicity of **F** is higher than that of the formamides (Section 1-7). Resultant intermediate **H** can undergo two scenarios: insertion of the C=N double bond or that of the C=O double bond into the Ir–H bond to form intermediates **I** and **J**, respectively. Subsequent reductive elimination[4] forms Ir(I) complex **K** and formamide or its tautomer, or the heterolytic cleavage[3] of the dihydrogen ligand forms Ir(III) complex **L** and formamide or its tautomer. Finally, the oxidative addition of H_2 and/or coordination of dihydrogen regenerates intermediate **G**.

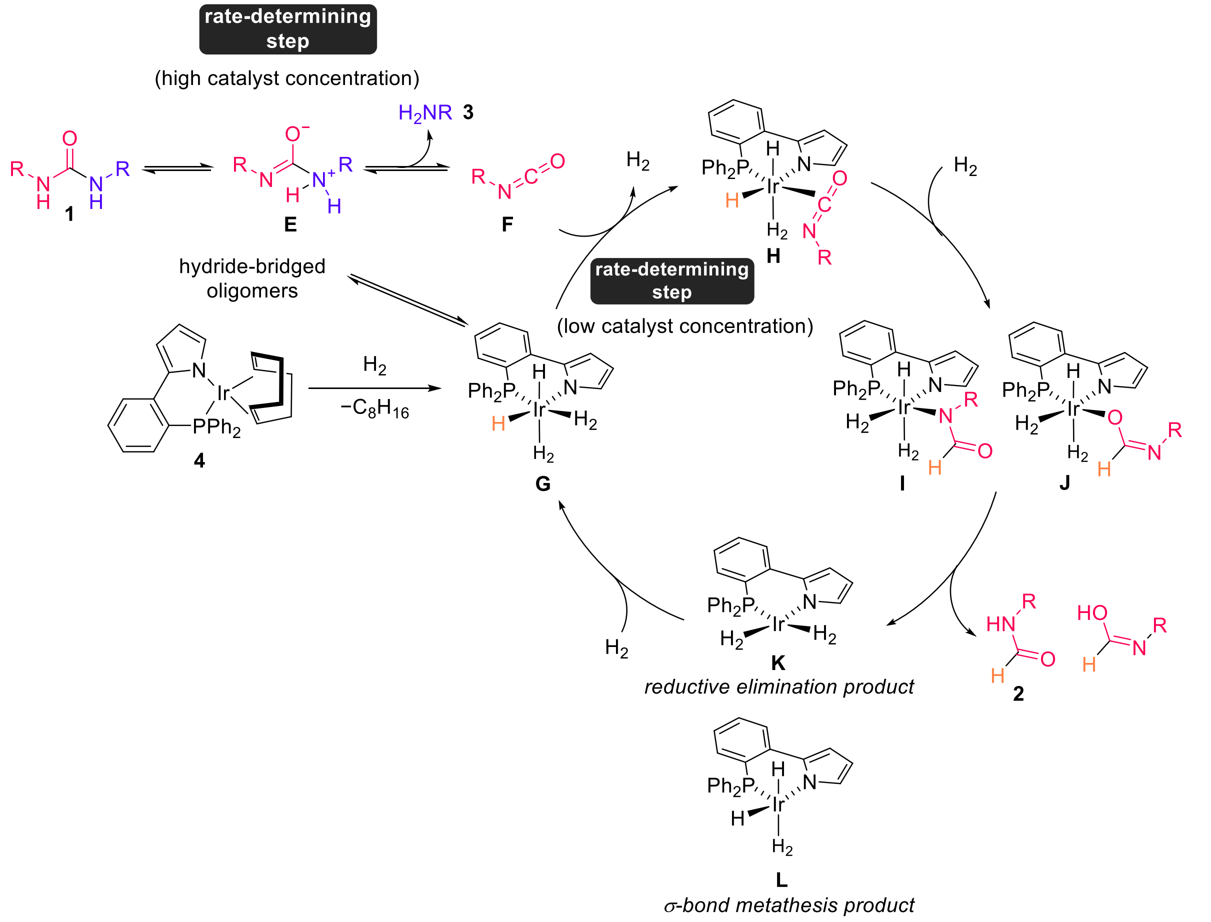

Supplementary Fig 8.

A proposed catalytic cycle: thermal decomposition of urea to isocyanate and its hydrogenation mechanism (*thermal decomposition pathway*). One of the possible configuration(s) is drawn for each intermediate.

The experimental results pertaining to the mechanistic study (Sections 1-6 to 1-11) and their consistency with the two above-described reaction mechanisms are summarized in Supplementary Tab 4.

Supplementary Tab 4.

Consistency of the proposed reaction mechanisms with mechanistic studies.

Mechanistic studies	metal-ligand cooperative pathway	thermal decomposition pathway
Observation of isocyanate as an intermediate (Section 1-6)	—	indicates that isocyanate is a possible reaction intermediate under the conditions.
Hydrogenation of formamide and isocyanate, and competitive reactions (Section 1-7)	clearly show the inertness of formamides under the catalysis.	could be interpreted that either hydrogenation of isocyanate is not the major pathway or higher concentration of isocyanate retards the hydrogenolysis to formamides.
NBO analysis (Section 1-8)	consists with the proposed origin of the chemoselectivity.	—
Kinetic studies (Section 1-9)	indicate the reaction of Ir intermediate **B** with urea as the rate-determining step and that **B** is in an equilibrium between oligomeric Ir-H species.	indicate alternating the rate-determining step: thermal decomposition of urea at high concentrations of catalyst; the reaction of isocyanate **F** with Ir intermediate **G** at low concentrations of catalyst.
Observation of Ir hydride species (Section 1-10)	consists with the observed saturation kinetics respects with Ir complex **4** arising from an equilibrium between monomeric and oligomeric Ir-H species.	consists with the observed 0.6^{th}-order kinetics of Ir complex **4** at lower catalyst loading.
Regioselectivity of unsymmetric ureas (Section 1-11)	could be explained by steric hindrance of leaving amino moiety.	could be explained by selective formation of zwitterionic intermediate **E**.

1-6. Observation of isocyanate as an intermediate

In the *thermal decomposition pathway*, an isocyanate is proposed as a key intermediate. It has been reported that 1,3-diphenylurea undergoes thermal decomposition at approximately 500 K in the solid state to produce phenyl isocyanate and aniline[5]. This thermal decomposition has been addressed employing theoretical calculations, and isocyanate formation from 1,3-dimethylurea was calculated to be endothermic by 95.3 kJ/mol with an energy barrier of 217.6 kJ/mol[6]. Although previous studies have performed catalytic hydrogenolyses of urea derivatives at temperatures similar to or higher than those in the present study, isocyanates have never been proposed as intermediates[7–16]. To address the possibility of isocyanate intermediacy in the hydrogenolysis of urea derivatives, we performed several control experiments.

When a mixture of **1a** (1.0 mmol) and tetraglyme (3 mL) was heated at 130 °C in Kugelrohr distillation apparatus (at 1.7×10^{-2} to 2.3×10^{-2} MPa), 0.15% of phenyl isocyanate **27a** was collected by distillation, along with a small amount of **1a** and unidentified compounds (Supplementary Figs 9 and 79–83).

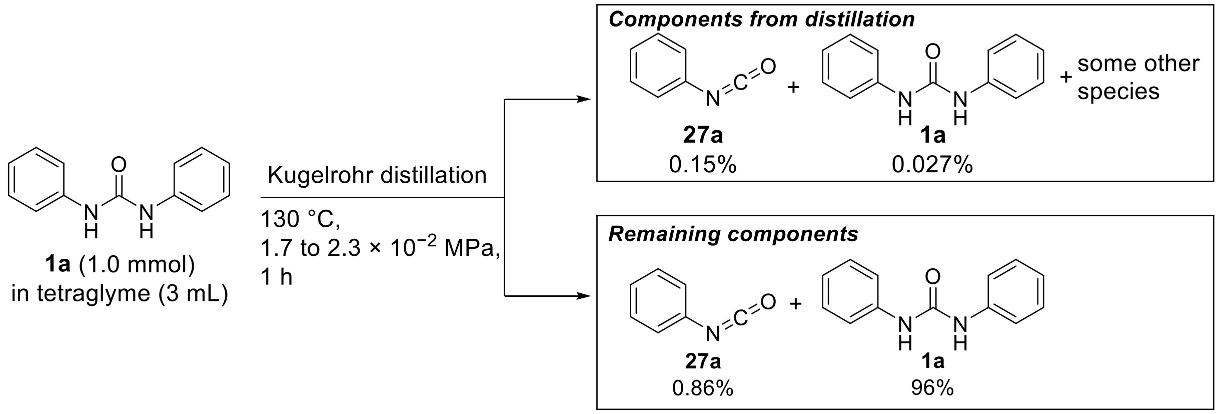

Supplementary Fig 9.

Thermal decomposition of urea **1a** to isocyanate **27a** (direct observation).

Thermal decomposition of urea to isocyanate was also suggested by the results of the exchange reaction between **1h** and **27a** (Supplementary Fig 10). When a mixture of urea **1h** and isocyanate **27a** was heated at 130 °C for 48 h, followed by the addition of morpholine (**3s**) for isocyanate trapping, unsymmetric urea **1ah** was obtained in 34% yield along with **1as** (19%) and **1hs** (53%). The formation of **1ah** can be explained by the thermal decomposition of **1h** to **27h** and *p*-anisidine (**3h**) and the subsequent coupling of **3h** with **27a**. Indeed, not only **27a** but also **27h** was trapped by **3s** to give **1as** and **1hs**, respectively.

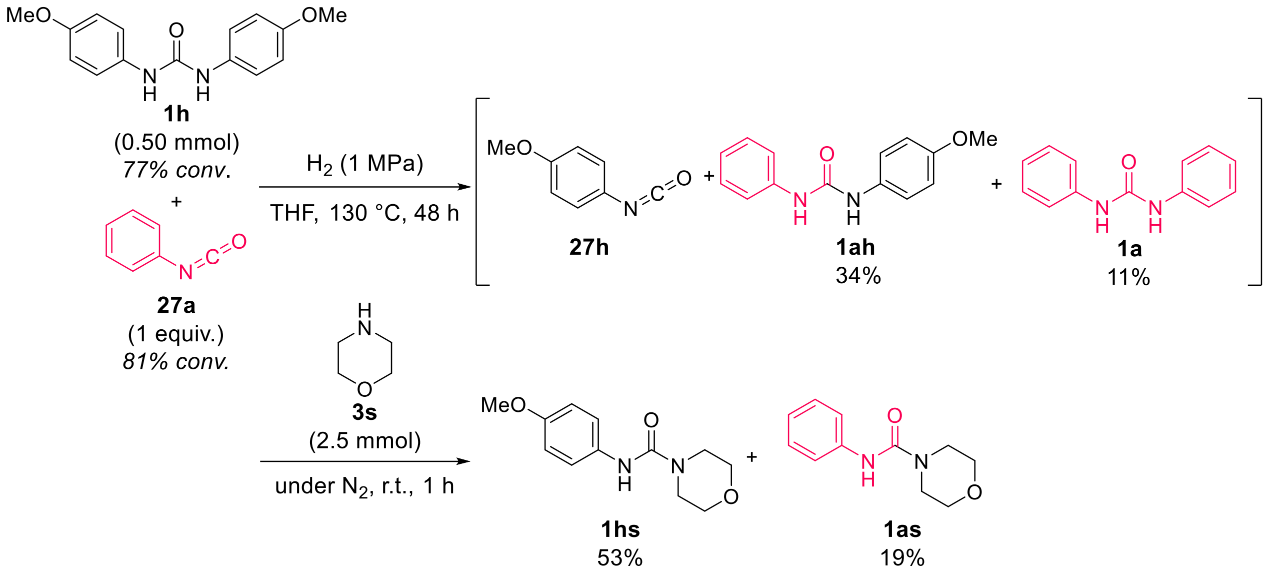

Supplementary Fig 10.

Thermal decomposition of urea **1h** to isocyanate **27h** (indirect observation).

Thermal decomposition pathway: In the thermal decomposition experiment (Supplementary Fig 9), the yield of **27a** was low because the equilibrium between **1a** and the mixture of **27a** and aniline (**3a**) largely shifted to favor **1a**. However, the formation of isocyanate **27a** at 130 °C was indeed observed. The exchange reaction also indicated the thermal decomposition of urea to isocyanate at 130 °C (Supplementary Fig 10).

1-7. Hydrogenation of formamide and isocyanate, and competitive reactions

To understand the reactivity of the possible intermediates, control and competitive reactions using formamide and isocyanate were conducted. When formamide **2b** was subjected to hydrogenolysis under Condition A, reduction to aniline **3b** proceeded in 8% yield and 92% of **2b** was recovered (Supplementary Fig 11). Furthermore, a competitive reaction using a 1:1 mixture of 1,3-diphenylurea (**1a**) and formamide **2b** under Condition A resulted in the recovery of **2b** in 95% yield, while the coexistence of **2b** did not affect the hydrogenolysis of **1a** (Supplementary Fig 12). These results clearly demonstrate that formamides are neglected by catalyst **4**, which is in sharp contrast to previously reported Ru catalyst that preferentially reacts with formamides over ureas[7].

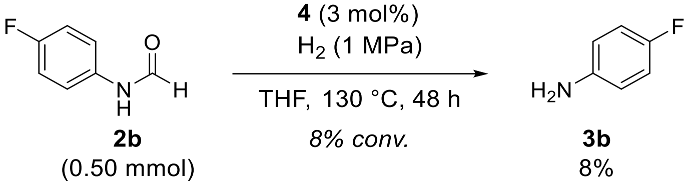

Supplementary Fig 11.

Hydrogenolysis of *N*-(4-fluorophenyl)formamide (**2b**) using Ir catalyst **4**.

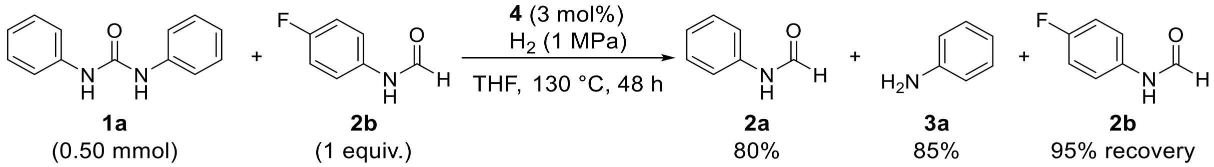

Supplementary Fig 12.

Competitive reaction of urea **1a** and formamide **2b**.

To elucidate isocyanate intermediacy, the reactivity of phenyl isocyanate (**27a**) as a key intermediate in the *thermal decomposition pathway* was investigated. Under Condition A, **27a** was completely consumed to give **2a** (37%), **3a** (9%), and **1a** (26%) (Supplementary Fig 13). The formation of **1a** can be explained by the addition of **3a** to **27a**. Although hydrolysis of **27a** by H_2O present as a contaminant would also form **3a**, a control experiment revealed that this process was negligible (see Section 7-15). Assuming that **1a** was formed by the reaction of **27a** and **3a**, it can be deduced that the catalytic hydrogenolysis of **27a** formed **3a** in 35% yield. The product distribution differed from the values in Fig. 3 (**2a**/**3a** = 80/82), implying that **27a** may not be the sole intermediate. The competitive reaction between **27a** and formamide **2b** resulted in 93% recovery of **2b** and a low yield of **2a** (Supplementary Fig 14, **3a** and **1a** were not quantified). This could be rationalized by two possible scenarios: either the hydrogenation of **27a** is not the major pathway, or a higher concentration of **27a** retards hydrogenation to **2a**.

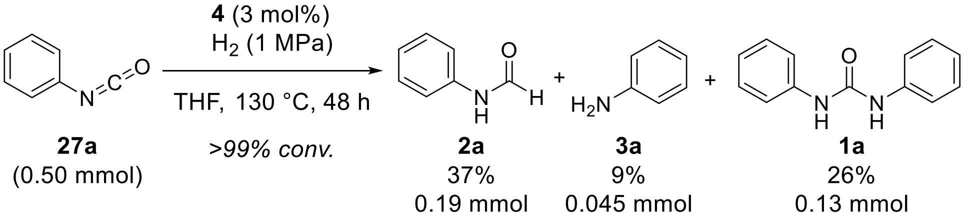

Supplementary Fig 13.

Hydrogenation of isocyanate **27a** using Ir catalyst **4**.

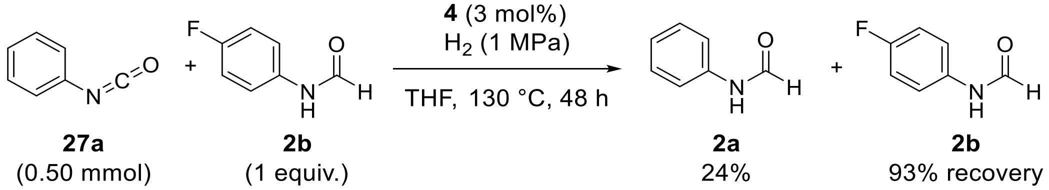

Supplementary Fig 14.

Competitive reaction of isocyanate **27a** and formamide **2b**.

1-8. NBO analysis

In natural bond orbital (NBO) analysis, a large negative value indicates a higher electron density and a large positive value indicates a higher electron affinity. We performed NBO analysis for 1,3-diphenylurea (**1a**), formanilide (**2a**), ethyl benzoate (**13**), and benzamides **1k’** and **1k”** as models of **1k** (Supplementary Fig 15). The carbonyl oxygen of **1a** was determined to have the highest electron density (−0.655) compared to **2a**, ester **13**, and benzamides **1k’** and **1k”**. Similarly, the electrophilicity of the carbonyl compounds was estimated by NBO analysis. Although urea has resonance forms with two nitrogen atoms, the carbonyl carbon has a relatively high NBO value (+0.792) compared to other carbonyl functionalities calculated.

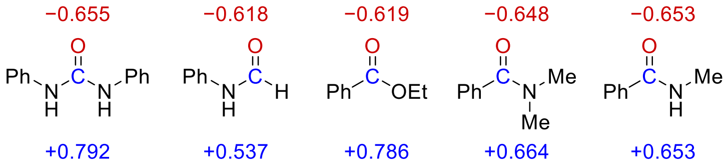

1a **2a** **13** **1k'** **1k"**

Supplementary Fig 15.

NBO analysis of urea **1a**, formanilide (**2a**), ethyl benzoate (**13**), and benzamides (**1k’** and **1k”**) at M06/Def2TZVP SMD(THF) level of theory (see Section 10).

Metal-ligand cooperative pathway: Because urea has a higher electron density than formamide, NBO analysis supports the proposed origin of the chemoselectivity based on the basicity of the oxygen atom in the protonation step. The observed functional group tolerance can also be explained by NBO analysis. Compared to urea **1a**, although ester **13** has a comparable electron affinity at the carbonyl carbon (+0.792 (**1a**) vs. +0.786 (**13**)), the basicity of its ester oxygen is much weaker (−0.655 (**1a**) vs. −0.619 (**13**)). Therefore, protonation by pyrrole preferentially occurs at the urea oxygen. Tertiary and secondary benzamides **1k’** and **1k”** have a comparable electron densities at the carbonyl oxygen to that of urea **1a** (−0.655 (**1a**) vs. −0.648 (**1k’**)/−0.653 (**1k”**)). However, the electron affinity of the carbonyl carbon in **1k’** and **1k”** is much weaker than that of **1a** (+0.792 (**1a**) vs. +0.664 (**1k’**)/+0.653 (**1k”**)). Therefore, the hydride transfer to benzamides is unfavorable. These electronic properties of the carbonyl moiety well explain the observed chemoselectivities.

1-9. Kinetic studies

To identify the rate-determining step in the catalytic cycles, we performed reactions at varying initial concentrations for each reaction component, urea **1a**, H_2, catalyst **4**, and KO*ᵗ*Bu additive, to determine the initial reaction rate (Section 4, Supplementary Figs 26–49 and Supplementary Tabs 8–30). Using the data for the consumption of urea **1a**, double logarithm plots of the initial reaction rate versus the component initial concentration were constructed (Supplementary Fig 16).

The plot of the reaction rate against the initial concentration of **1a** shows 1st-order kinetics with respect to **1a** (Supplementary Fig 16a), whereas the reaction obeys 0th-order kinetics with respect to H_2 pressure (Supplementary Fig 16b). For Ir catalyst **4**, the reaction order was determined to be 0.6^{th}-order at <2 mol% catalyst loading, but it saturated at catalyst loading of >2 mol% (Supplementary Fig 16c). The reaction obeyed 0.5^{th}-order kinetics with respect to KO*ᵗ*Bu. This result is consistent with the acceleration of the hydrogenolysis by the addition of KO*ᵗ*Bu (Supplementary Fig 16d).

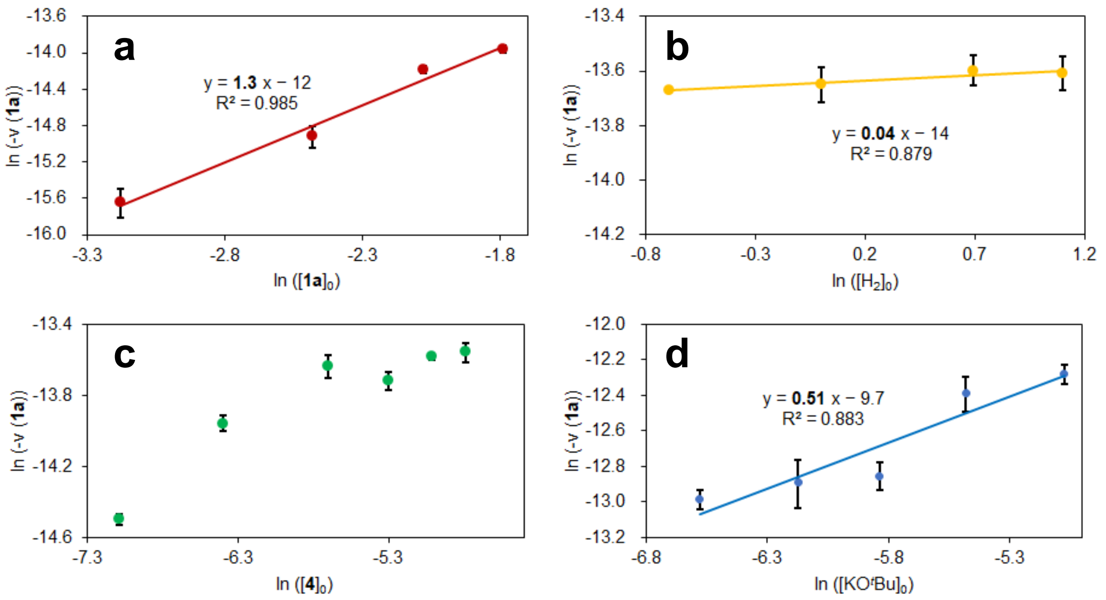

Supplementary Fig 16.

Double logarithm plots of initial reaction rate against initial concentration of reaction components. (**a**) Dependency on urea **1a**: **1a** (0.042–0.167 M), **4** (1.7 mM), and H_2 (1 MPa) in THF at 130 °C. (**b**) Dependency on H_2: **1a** (0.17 M), **4** (5.0 mM), and H_2 (0.5–3.0 MPa) in THF at 130 °C. (**c**) Dependency on catalyst **4**: **1a** (0.17 M), **4** (0.83–8.33 mM), and H_2 (1 MPa) in THF at 130 °C. (**d**) Dependency on KO*ᵗ*Bu: **1a** (0.17 M), **4** (0.42 mM), KO*ᵗ*Bu (1.4–6.3 mM), and H_2 (1 MPa) in THF at 130 °C. Error bars represent the standard error (SE).

In addition to the effects of the reactants and catalysts, those of the products were further investigated. In contrast to formamides, which did not affect hydrogenolysis (Section 1-7, Supplementary Fig 12), the addition of aniline (**3a**) during the hydrogenolysis of **1a** affected the reaction efficiency (Supplementary Fig 17). When the reaction of **1a** was stopped at an early stage (12 h), **2a** was obtained in 27% yield. The addition of 0.6 equiv of **3a** resulted in 12% conversion of **1a** to give **2a** in 10% yield, clearly indicating the negative effects of aniline (**3a**) on the hydrogenolysis.

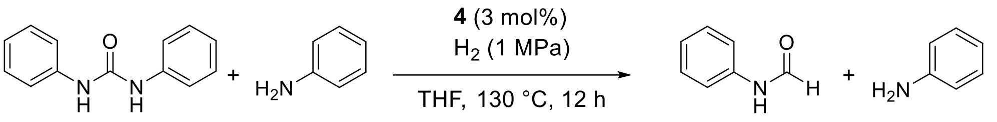

1a (0.50 mmol) **3a** (*x* mmol) **2a** **3a**

**3a**	conv. of **1a**	yield of **2a**	yield of **3a**
none	29%	27%	29%
0.30 mmol	12%	10%	10%*

Supplementary Fig 17.

Effects of aniline (**3a**) on the hydrogenolysis of urea **1a**. *, Except for the added aniline.

Metal-ligand cooperative pathway: Considering the observed kinetics, the rate-determining step in this catalytic cycle was deduced to be the reaction of Ir hydride species **B** with urea, as shown in Supplementary Fig 7. The kinetic profile of the Ir catalyst could be explained by assuming equilibrium between a catalytically active monomeric Ir complex and dormant species (see Section 1-10). KO^{t}Bu participation in the rate-determining step with a relatively small reaction order can be explained by the assumption that the potassium cation participates in the rate-determining protonation/hydride transfer processes as a Lewis acid to activate the C=O double bond of urea. On the other hand, the negative effect of aniline likely arises due to competing protonation between urea and aniline.

Thermal decomposition pathway: In the reaction pathway shown in Supplementary Fig 8, the concentration of the Ir catalyst determines whether the rate-limiting step is isocyanate formation (**1** to **F**) or hydrogenation thereof (**G** to **H**). When the concentration of the Ir catalyst is low, the rate-determining step is isocyanate hydrogenation (**G** to **H**). At higher concentrations of Ir complex **4**, the formation of isocyanate from urea (**1** to **F**) is the rate-determining step. Because the thermal decomposition of urea to an isocyanate and amine is an equilibrium process, the addition of aniline shifts the equilibrium to urea, reducing the reaction efficiency, whereas KO^{t}Bu promotes the formation of isocyanate from urea.

1-10. Observation of Ir hydride species

The treatment of complex **4** with H_2 (0.5 MPa) in THF-d_8 resulted in a complex mixture of many Ir hydride species along with the formation of cyclooctane (Supplementary Figs 18 and 84). This likely resulted from the formation of hydride-bridged dimers and oligomers[17,18]. The addition of 2 equiv of PPh_3 to the reaction led to the formation of mononuclear Ir(III) dihydride species **28** and **29** in an 85:15 ratio at room temperature (Supplementary Figs 18 and 86). Isomer **29** was converted to **28** (93% purity, ^{31}P NMR) upon heating at 50 °C for 12 h (Supplementary Figs 18 and 87). Therefore, the initial step wherein the hydride species is generated from **4** is operable. Because the addition of urea to the complex mixture of hydride species did not give monomeric Ir hydride species, unlike PPh_3 (see Section 7-18), an equilibrium between monomeric species and hydride-bridged dimers and oligomers possibly exists in the catalytic reaction.

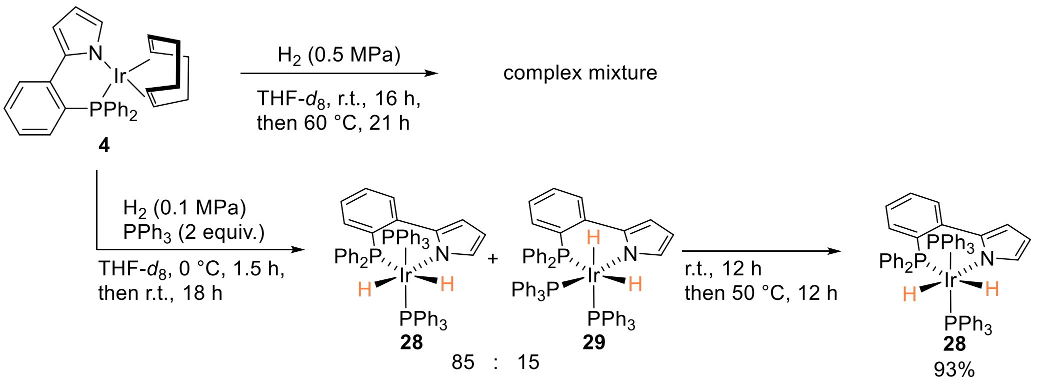

Supplementary Fig 18.

The reaction of Ir complex **4** with H_2.

Metal-ligand cooperative pathway: An equilibrium between monomeric Ir complex and hydride-bridged dimers and oligomers was observed as previous reports that proposed both hydride-bridged dimers and trimers[17,18]. Therefore, the observed saturation kinetics of Ir catalyst **4** (Supplementary Fig 16c) can be interpreted by assuming second- and higher-order kinetics for the oligomerization between Ir hydride species.

Thermal decomposition pathway: When the concentration of Ir catalyst **4** is <2 mol%, the rate-determining step is the hydrogenation of isocyanate. The relatively small reaction order of 0.6 with respect to **4** can be explained by assuming an equilibrium between the catalytically active monomeric species and dimers or oligomers.

1-11. Regioselectivity of unsymmetric ureas

As shown in Fig. 3d, unsymmetric ureas consisting of aniline and *N*-methylaniline, such as **1ao**, afforded formanilides and *N*-methylanilines under both Conditions A and B. On the other hand, regioselectivity was altered depending on the reaction conditions in the case of **1as**, which contained aniline and morpholine groups. Under Condition A, formanilide (**2a**) and morpholine (**3s**) were obtained in low yield, while *N*-formylmorpholine (**2s**) and aniline (**3a**) were obtained under Condition B using KO^{t}Bu. The absence or trace amounts of symmetric ureas observed in these reactions indicated that the exchange reaction between urea and amine was less significant in the hydrogenolysis of **1ao** and **1as**.

Considering previously reported base-promoted transamidation[19], we conducted control experiments in the absence of Ir catalyst **4** (Supplementary Figs 19 and 20). No transamidation was observed when a 1:1 mixture of **2a** and **3o** was heated at 130 °C in THF for 48 h (Condition A). The reversed combination of **2o** and **3a** also resulted in no transamidation. The combination of formanilide (**2a**) and morpholine (**3s**) resulted in <10% conversion. Therefore, formyl group transfer via transamidation was negligible under neutral conditions. In contrast, the addition of 10 mol% KO^{t}Bu promoted the transamidation reaction. Although transamidation between **2a** and **3o** (12% and 7% conversion, respectively) or **2o** and **3a** (13% and 13% conversion, respectively) was sluggish, that between **2a** and secondary amine **3s** proceeded in 90% yield. Therefore, the observed regioselectivity for unsymmetric ureas possessing the *N*-methylaniline moiety reflects the regioselectivity of C–N bond cleavage. On the other hand, it could not be distinguished whether the observed regioselectivity for **1as** originated from C–N bond cleavage or transamidation when **2s** was obtained.

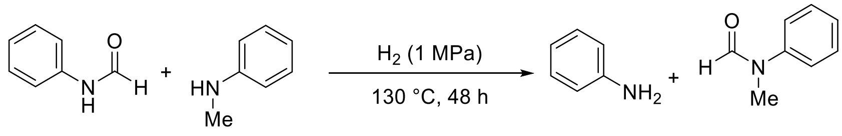

	**2a** (0.50 mmol)	**3o** (0.50 mmol)	**3a**	**2o**
with KO^{t}Bu (10 mol%, toluene)	*12% conv.*	*7% conv.*	12%	6%
without KO^{t}Bu (THF)	*<1% conv.*	*1% conv.*	n.d.	n.d.

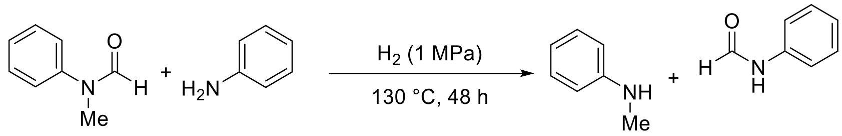

	**2o** (0.50 mmol)	**3a** (0.50 mmol)	**3o**	**2a**
with KO^{t}Bu (10 mol%, toluene)	*13% conv.*	*13% conv.*	15%	13%
without KO^{t}Bu (THF)	*<1% conv.*	*<1% conv.*	n.d.	n.d.

Supplementary Fig 19.

Formyl group transfer from formanilide (**2a**) to *N*-methylaniline (**3o**)/from *N*-methyl formanilide (**2o**) to aniline (**3a**).

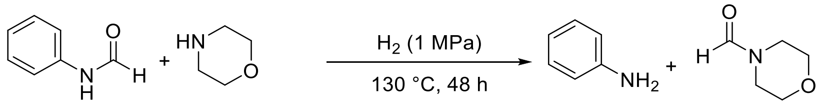

	**2a** (0.50 mmol)	**3s** (0.50 mmol)	**3a**	**2s**
with KO^{t}Bu (10 mol%, toluene)	*90% conv.*	*90% conv.*	90%	90%
without KO^{t}Bu (THF)	*7% conv.*	*6% conv.*	8%	8%

Supplementary Fig 20.

Formyl group transfer from formanilide (**2a**) to morpholine (**3s**).

Metal-ligand cooperative pathway: Because hydrogenated intermediate **D** undergoes C–N bond cleavage (Supplementary Fig 7), the regioselective cleavage of unsymmetric urea **1ao** and related substrates is controlled by steric hindrance to eliminate bulky *N*-methylanilines. Similarly, sterically hindered **3s** may undergo elimination under neutral conditions. Because of competing transamidation, it could not be determined whether the observed regioselectivity of **1as** under Condition B arose from the C–N bond cleavage event or transamidation.

Thermal decomposition pathway: Because the thermal decomposition of urea is triggered by the formation of zwitterionic intermediate **E** (Supplementary Fig 8), the C–N bond of the secondary amino moiety is selectively cleaved, as observed in the hydrogenolysis of trisubstituted ureas **1ao** and **1as**. When formanilide (**2a**) and morpholine (**3s**) were reacted in the presence of KO^tBu, transamidation occurred to give aniline (**3a**) and *N*-formylmorpholine (**2s**) (Supplementary Fig 20). Therefore, the opposite regioselectivity observed with **1as** under Condition B can be explained by transamidation. Under Condition B, hydrogenolysis of **1ao** did not proceed regioselectively, probably because the reaction rate of the over-reduction of **2a** to **3a** was faster than that of the transamidation between **2a** and **3o**. Indeed, the transamidation was slow, even in the presence of KO^tBu (Supplementary Fig 19).

1-12. Degradation of polyurea resins and their chemical recycling

Polyurea resins are robust materials with applications in coatings[20], adhesives[21], and drug delivery systems[22]. Therefore, the global polyurea market size is growing and is estimated to reach USD 1.4 billion by 2030[23]. Polyurea resins are industrially synthesized from diisocyanate and diamine via addition of the amino moiety to the carbon in the isocyanate moiety (Supplementary Fig 21). The copolymerization of these two monomers allows the incorporation of two different diamine segments in one polyurea chain, which possesses two different functionalities in an alternating manner[20].

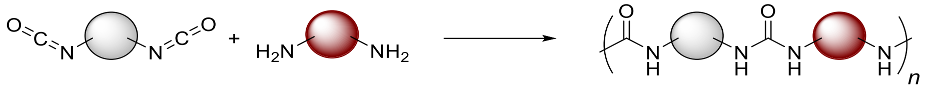

Supplementary Fig 21.

Industrial synthesis of polyurea resins: the reaction of diisocyanate with diamine.

Degradation of polyurea resins by chemical processes reported so far can be classified into the following three patterns. **A:** Catalytic hydrogenolysis of polyurea resins to MeOH and diamines using Mn, Ru, or Ir catalysts (Supplementary Fig 22a)[13,16,24,25]. **B:** Hydrolysis of polyurea resins to diamines and CO_2 at 190 °C under 7 MPa of CO_2 in the presence of H_2O (Supplementary Fig 22b)[26]. This method has solely been examined for polyurea resins consisting of one kind of diamine segment. **C:** Transesterification/transamidation of urea resins to dicarbamates using carbonates and MgO–ZnO catalyst[27] or using urea, alcohols, and CuO–ZnO catalyst[28]. These methods have been employed to polyurea resins with one kind of diamine segment (Supplementary Fig 22c).

In contrast to these methods, the present catalytic hydrogenolysis of polyurea **17**, which consists with two different diamine segments, afforded one diamine segment as diformamide and the other as diamine under milder conditions, enabling easy separation of degraded products (Supplementary Fig 22d). Each of the major components **18** and **16** was further purified by extraction with hexane from the reaction mixture (**16**) and then silica gel column chromatography with CH_2Cl_2/ethyl acetate (**18**).

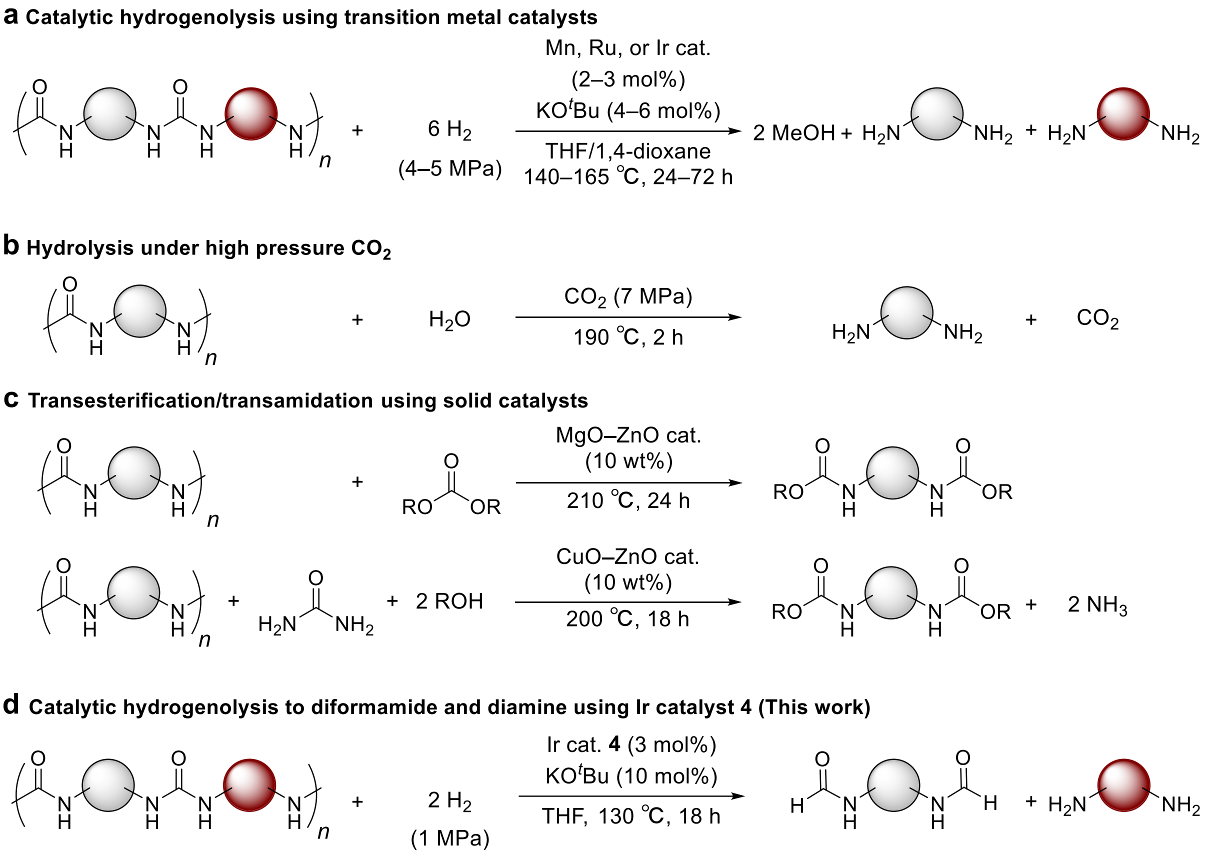

Supplementary Fig 22.

Degradation of polyurea resins by chemical processes. (**a**) Catalytic hydrogenolysis using transition metal catalysts. (**b**) Hydrolysis under high pressure CO_2. (**c**) Transesterification/ transamidation using solid catalysts. (**d**) Catalytic hydrogenolysis to diformamide and diamine using Ir catalyst **4** (This work).

As a synthetic method for urea moiety, dehydrogenative coupling of formamides and amines[29–34] or that of MeOH and amines via a formamide intermediates[35,36] has been reported in literature. The former reaction was applied to the dehydrogenative polymerization of diformamides and diamines to form polyurea resins by using Ru catalyst[29]. Therefore, alternating copolymers could selectively be resynthesized from the degraded products in this work by the means of dehydrogenative coupling (Supplementary Fig 23a). As an alternative dehydrogenative polymerization for the polyurea synthesis, reaction of MeOH and diamines using Ru or Mn catalyst was also reported[34,37]. However, this method has only been examined for polyurea resins consisting of one kind of diamine segment, unlike the reaction of diisocyanate with diamine, because this method cannot be applied to the alternating copolymerization of different diamine molecules and MeOH (Supplementary Fig 23b).

Therefore, the combination of the present chemo- and regioselective hydrogenolysis and dehydrogenative coupling sequence enables an efficient chemical recycling of polyurea resins consisting of two alternating different diamine segments.

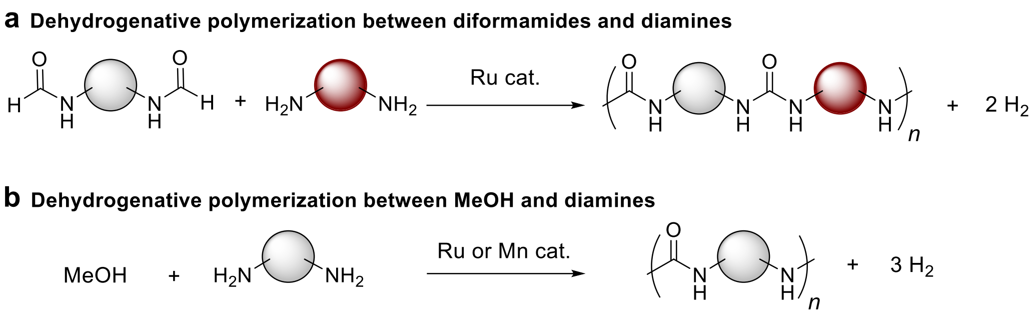

Supplementary Fig 23.

Dehydrogenative polymerization. (**a**) Dehydrogenative polymerization between diformamides and diamines. (**b**) Dehydrogenative polymerization between MeOH and diamines.

1-13. Condition screening for the hydrogenolysis of polyurea **17** using Ir catalyst **4**

Optimization of the reaction conditions for the hydrogenolysis of polyurea resin **17** is summarized in Supplementary Tab 5. In the absence of KO^{t}Bu, monomeric products **16** and **18**–**22** were not obtained at 130 °C (entry 1) and only trace amounts of **16** and **20** were observed at an elevated temperature (entry 2). The addition of 10 mol% KO^{t}Bu promoted hydrogenolysis to afford diformanilide **18** (66%), monoformanilide **19** (28%), and aliphatic diamine **16** (83%) (entry 3). The formation of **19** was attributed primarily to the over-reduction of the formamides. Therefore, the reaction time was shortened. When the reaction time was shortened from 22 to 18 h, the yield of **19** decreased to 24% and that of **18** increased to 72% (entry 4 and Fig. 5). However, shorter reaction times of 14 or 10 h did not improve the yield of **18** and led to a decreased yield of **16** (entries 5 and 6). A higher reaction temperature (170 °C) and the use of KO^{t}Bu resulted in the formation of aromatic diamine **20** (90%) and diformamide **21** (28%) via transamidation and over-reduction (entry 7).

Supplementary Tab 5.

Hydrogenolysis of polyurea resin **17** using Ir catalyst **4**.*

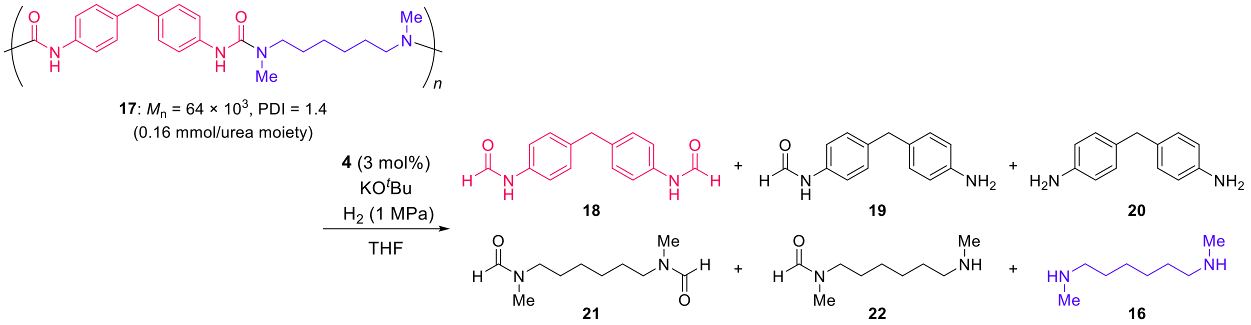

entry	KO^{t}Bu (mol%)	temp. (°C)	time (h)	**18** (%)	**19** (%)	**20** (%)	**21** (%)	**22** (%)	**16** (%)
1	0	130	22	n.d.	n.d.	n.d.	n.d.	n.d.	n.d.
2	0	170	22	n.d.	n.d.	<5	n.d.	n.d.	<5
3	10	130	22	66	28	4	5	n.d.‡	83
**4**	**10**	**130**	**18**	**72 (65)**	**24**	**2**	**n.d.**	**n.d.**	**88 (48)**
5	10	130	14	72	23	2	n.d.	n.d.	84
6	10	130	10	65	21	2	n.d.	n.d.	77
7†	10	170	22	n.d.	n.d.	90	28	n.d.‡	34

*, Reaction conditions: **17** (0.16 mmol), **4** (3 mol%), KO^{t}Bu, THF (3 mL), H_2 (1 MPa). The yields were determined by ^{1}H NMR spectroscopy with the internal standard (dibromomethane). Isolated yields are given in parenthesis; †, THF (2 mL) was used as solvent; ‡, **22** was not observed on GC analysis.

2. General Information

2-1. Manipulation

All reactions were carried out using a standard glovebox technique under argon gas or a Schlenk technique under nitrogen purified by passing through Nikka Seiko dry column DC-L4 (NIKKA SEIKO Co., LTD). All hydrogenolysis reactions were performed in a 50 mL stainless steel autoclave.

2-2. Instrumentation

Nuclear magnetic resonance (NMR) spectra were recorded on BRUKER Ascend500 (^{1}H: 500 MHz, ^{13}C: 126 MHz, ^{19}F: 471 MHz, ^{31}P: 202 MHz with digital resolution of 0.305, 0.908, 1.73, 2.49 Hz, respectively), or JEOL ECS400 (^{1}H: 400 MHz, ^{13}C: 101 MHz, ^{19}F: 376 MHz with digital resolution of 0.114, 0.958, 11.6 Hz, respectively) at ambient temperature unless otherwise noted. Chemical shift values for protons were referenced to the residual proton resonance of chloroform-*d* ($CDCl_3$, δ: 7.26), dimethyl sulfoxide-d_6 (DMSO-d_6, δ: 2.50), water-d_2 (D_2O, δ: 4.79), dichloromethane-d_2 (CD_2Cl_2, δ: 5.32), benzene-d_6 (C_6D_6, δ: 7.16), or tetrahydrofuran-d_8 (THF-d_8, δ: 3.58). Chemical shift values for carbons were referenced to the carbon resonance of $CDCl_3$ (δ: 77.2), DMSO-d_6 (δ: 39.5), CD_2Cl_2 (δ: 53.8), C_6D_6, (δ: 128.1), or THF-d_8 (δ: 67.2). In determining NMR yields, ^{1}H NMR spectra recorded on JEOL ECS400 or BRUKER Ascend500 using pulse sequence with 45° angle pulse and 30.0 s of relaxation delay were used for precise quantification. Mass spectra (MS) were taken with an electron spray ionization time-of-flight (ESI-TOF) method on a JEOL JMS-T100LP AccuTOF LC-plus mass spectrometer. In determining GC yields, a Shimadzu GC-2014 gas chromatography instrument (carrier gas: N_2) equipped with InertCap 5MS/Sil capillary column (0.25 mm i.d., 0.25 μm df, 30 m, GL Sciences) and a flame ionization detector (FID) was used with calibration curves made with decane as an internal standard. Gas chromatography-mass spectroscopy (GC-MS) analyses were carried out with a Shimadzu GCMS-QP2010 gas chromatograph mass spectrometer with electron-ionization method and gas-chromatograph equipped an InertCap 5MS/Sil capillary column (0.25 mm i.d., 0.25 μm df, 30 m, GL Sciences) with helium as the carrier gas. The molecular weight of polyurea resin **17** was analyzed by a Shimadzu prominence liquid chromatography system equipped with GPC KD-804 (Shodex) calibrated with standard polystyrene sample. X-ray crystallographic analyses were performed on a Rigaku Varimax dual with a hybrid photon counting detector. Elemental analyses were performed by Microanalytical Laboratory of Department of Chemistry, Graduate School of Science, the University of Tokyo.

2-3. Materials

Anhydrous hexane, THF, and toluene were purchased from Kanto Chemical Co. Inc. (Kanto) and purified by the method of Pangborn *et al.* [38]. C_6D_6 was purchased from Sigma-Aldrich Chemical Co. LLC (Aldrich) and dried over activated 4Å molecular sieves and degassed by freeze-pump-thaw cycles. THF-d_8 was purchased from Kanto and dried over Na/K alloy (1:1) in a glovebox and used after passing through a plug of dry silica-gel. The following reagents were purchased and used as received: H_2 gas (Suzuki Shokan Co., LTD.), *n*-pentane (super dehydrated, FUJIFILM Wako Pure Chemical Co., Ltd. (Wako)), 1,4-dioxane (super dehydrated, Wako), *N*,*N*-dimethylformamide (super dehydrated, Wako), cyclohexane (super dehydrated, Wako), $CDCl_3$ (Kanto), DMSO-d_6 (Kanto), D_2O (Kanto), CD_2Cl_2 (Kanto), sodium hydride (dry, 90%, Aldrich), 1,3-diphenylurea (TCI), formanilide (TCI), aniline (Wako), phenyl isocyanate (TCI), heptane (TCI), dibromomethane (Wako), 1,3,5-trimethoxybenzene (TCI), potassium *tert*-butoxide (TCI), sodium *tert*-butoxide (TCI), lithium *tert*-butoxide (Wako), bis(trifluoromethanesulfonyl)imide (TCI), 1,3-dicyclohexylurea (TCI), *N*-cyclohexylformamide (TCI), cyclohexylamine (TCI), benzylurea (Aldrich), di-*tert*-butyl dicarbonate (TCI), benzylamine (TCI), 4-butylaniline (TCI), 4-butylphenyl isocyanate (TCI), *p*-fluoroaniline (Wako), *N*-(4-fluorophenyl)formamide (TCI), 4-chloroaniline (TCI), 4-chlorophenyl isocyanate (TCI), 3-chloroaniline (TCI), 2-chloroaniline (TCI), 4-bromoaniline (TCI), 4-*tert*-butylaniline (TCI), *p*-anisidine (TCI), 4'-methoxyformanilide (TCI), *N*,*N*-dimethylaniline (Wako), 4-aminobenzonitrile (TCI), morpholine (Kanto), 1,4-diazabicyclo[2.2.2]octane (DABCO) (TCI), 1,1'-carbonyldiimidazole (TCI), di-*tert*-butyl decarbonate

(TCI), 4-aminobenzoic acid (TCI), triethylamine (TCI), dipropylamine (TCI), 1-(3-dimethylaminopropyl)-3-ethylcarbodiimide hydrochloride (EDCI) (TCI), 1,5,7-triazabicyclo[4.4.0]dec-5-ene (TBD) (TCI), triphenylphosphine (TCI), 2,2-dimethylpropiophenone (Aldrich), ethyl benzoate (TCI), 2-(diphenylphosphino)benzoic acid (Aldrich), methylenediphenyl 4,4'-diisocyanate (TCI), *N*,*N*'-dimethyl-1,6-diaminohexane (TCI), 4,4'-diaminodiphenylmethane (TCI), ethyl formate (TCI), tetraglyme (TCI), *N*,*N*'-dimethyl-*N*,*N'*-diphenylurea (TCI), 2-imidazolidinone (TCI), 1,3-dimethyl-2-imidazolidinone (dehydrated, Kanto), trichlorosilane (TCI), chlorobis(1,5-cyclooctadiene)rhodium dimer (TCI), (1,5-cyclooctadiene)(pyridine)(tricyclohexylphosphine)iridium(I) hexafluorophosphate (TCI), tris(triphenylphosphine)rhodium(I) chloride (Aldrich), carbonylchlorobis(triphenylphosphine)iridium(I) (TCI). The following reagents were prepared according to literature procedures: chlorobis(1,5-cyclooctadiene)iridium dimer[39], 2-(2-diphenylphosphinophenyl)pyrrole[40], 2-(diphenylphosphino)benzenesulfonic acid[41], octyl phenylcarbamate[42], bis(1,5-cyclooctadiene)iridium(I) tetrakis(3,5-bis(trifluoromethyl)phenyl)borate[43], *N*,*N'*-(methylenebis(4,1-phenylene))diformamide[44], *N*-(4-(4-aminobenzyl)phenyl)formamide[44].

3. X-ray Crystallography

The crystals were mounted on a CryoLoop (Hampton Research Corp.) with a layer of light mineral oil (dried over metallic Na at 100 °C for overnight) and placed in a nitrogen stream at 93–123(2) K. The structures were solved by direct methods (SHELXT Version 2014/5[45]). The structure was refined on F^2 by the full-matrix least-squares method using SHELXL Version 2017/1[46] using Olex2 program[47]. The non-hydrogen atoms were anisotropically refined, while the hydrogen atoms were refined using the riding model. In complex **23**, one CF_3 group was disordered in two conformations and was refined with a 0.60:0.40 ratio. Occupancy of these disordered atoms was not refined. Crystallographic data are summarized in Supplementary Tab 6. The Olex2 program[47] was used to draw the molecular structures shown in Supplementary Fig 3.

Supplementary Tab 6.

Crystal data and data collection parameters.

	**4**	**7**	**9**	**23**
CCDC	2225764	2225765	2225766	2225767
Empirical formula	$C_{30}H_{29}IrNP$	$C_{26}H_{26}IrO_3PS$	$C_{36}H_{35}RhNP$	$C_{62}H_{42}BF_{24}IrNP$
Formula weight	626.71	641.70	615.53	1490.94
Color, description	orange, block	orangish yellow, block	yellow, block	red, block
Temperature, K	108	123	93	93
Crystal system	monoclinic	monoclinic	triclinic	monoclinic
Space group	$P2_1/n$	$P2_1/n$	$P{-1}$	$P2_1/n$
a, Å	9.71940(10)	12.5868(3)	11.2322(2)	18.9768(4)
b, Å	9.21000(10)	13.2059(3)	11.5019(2)	12.5395(3)
c, Å	26.7818(2)	14.0654(4)	11.8839(2)	25.3631(5)
α, °	90	90	70.479(2)	90
β, °	96.4380(10)	106.531(3)	76.655(2)	108.585(2)
γ, °	90	90	83.630(2)	90
V, Å^3	2382.27(4)	2241.31(10)	1407.02(5)	5720.7(2)
Z	4	4	2	4
ρ_{calc} g/cm^3	1.747	1.902	1.453	1.731
μ/mm^{-1}	5.691	6.149	0.690	2.483
F(000)	1232.0	1256.0	636.0	2936.0
Crystal size/mm^3	0.2 × 0.1 × 0.1	0.4 × 0.15 × 0.15	0.6 × 0.6 × 0.6	0.5 × 0.3 × 0.3
Radiation	Mo-Kα (λ = 0.71073)	Mo-Kα (λ = 0.71073)	Mo-Kα (λ = 0.71073)	Mo-Kα (λ = 0.71073)
2Θ range for data collection/°	4.322 to 57.832	4.922 to 57.756	4.69 to 58.494	5.39 to 58.684
Index ranges	−13 ≤ h ≤ 13, −12 ≤ k ≤ 12, −36 ≤ l ≤ 36	−15 ≤ h ≤ 16, −17 ≤ k ≤ 17, −19 ≤ l ≤ 18	−14 ≤ h ≤ 15, −15 ≤ k ≤ 14, −16 ≤ l ≤ 15	−25 ≤ h ≤ 25, −17 ≤ k ≤ 16, −33 ≤ l ≤ 33
Reflections collected	535643	35077	34057	138109
Independent reflections	6204 [R_{int} = 0.0577, R_{sigma} = 0.0093]	5376 [R_{int} = 0.0773, R_{sigma} = 0.0405]	6642 [R_{int} = 0.0642, R_{sigma} = 0.0387]	14436 [R_{int} = 0.0513, R_{sigma} = 0.0256]
Data/restraints/parameter	6204/0/298	5376/0/289	6642/0/352	14436/0/802
Goodness-of-fit on F_2	1.368	1.041	1.045	1.022
Final R indexes [I>=2σ (I)]	R_1 = 0.0279, wR_2 = 0.0714	R_1 = 0.0239, wR_2 = 0.0563	R_1 = 0.0288, wR_2 = 0.0735	R_1 = 0.0245, wR_2 = 0.0573
Final R indexes [all data]	R_1 = 0.0290, wR_2 = 0.0717	R_1 = 0.0269, wR_2 = 0.0573	R_1 = 0.0306, wR_2 = 0.0741	R_1 = 0.0277, wR_2 = 0.0584
Largest diff. peak/hole / e Å^{-3}	1.86/−1.42	0.89/−0.98	0.53/−0.83	1.64/−1.46

4. Kinetic Studies of Catalytic Hydrogenolysis

4-1. Kinetic profile for the hydrogenolysis of 1,3-diphenylurea (**1a**) using **4** under Condition A

Experiments were performed as follows: A 50 mL stainless steel autoclave, a glass tube, and a stirring bar were dried in an oven at 150 °C, and then cooled inside a glovebox under argon atmosphere. Catalyst **4** (9.4 mg, 15 µmol), 1,3-diphenylurea (**1a**) (106.1 mg, 0.50 mmol), and THF (3 mL) were added into the glass tube, and the tube was capped with a funnel to prevent evaporation of the solvent. After the glass tube was set in the autoclave and the autoclave was sealed, the autoclave was brought out from the glovebox. The autoclave was degassed three times using H_2 and was pressurized with 1 MPa of H_2 for 5 min with stirring. The reaction mixture was stirred in an isothermal heating block at 130 °C for each reaction time. After cooling to room temperature, H_2 was vented off carefully. To the reaction mixture, DMSO-d_6 (ca. 2 mL) was added to homogenize the reaction mixture, and dibromomethane (86.9 mg, 0.50 mmol) was added as an internal standard. The solution (THF/DMSO-d_6 = 3/2, 0.3 mL) was transferred into an NMR tube and diluted with DMSO-d_6 (0.3 mL) for NMR analysis. The conversion of **1a** and the yields of formanilide (**2a**) and aniline (**3a**) were determined by ^{1}H NMR spectroscopy with the internal standard.

The time-course was summarized in Supplementary Tab 7 and was plotted in Supplementary Fig 25, showing no significant induction period.

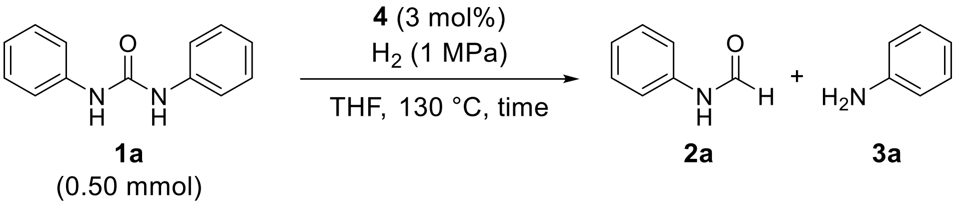

Supplementary Fig 24.

Hydrogenolysis of **1a** using **4** under Condition A.

Supplementary Tab 7.

Hydrogenolysis of **1a** using **4** under Condition A.

entry	time (h)	**1a** (M)	**2a** (M)	**3a** (M)
1	0	0.167	0	0
2	2	0.155	0.007	0.010
3	4	0.150	0.015	0.017
4	6	0.143	0.022	0.023
5	8	0.132	0.028	0.032
6	12	0.118	0.045	0.048
7	16	0.095	0.067	0.070
8	20	0.083	0.078	0.082
9	28	0.052	0.110	0.115
10	38	0.033	0.125	0.128
11	48	0.025	0.133	0.137

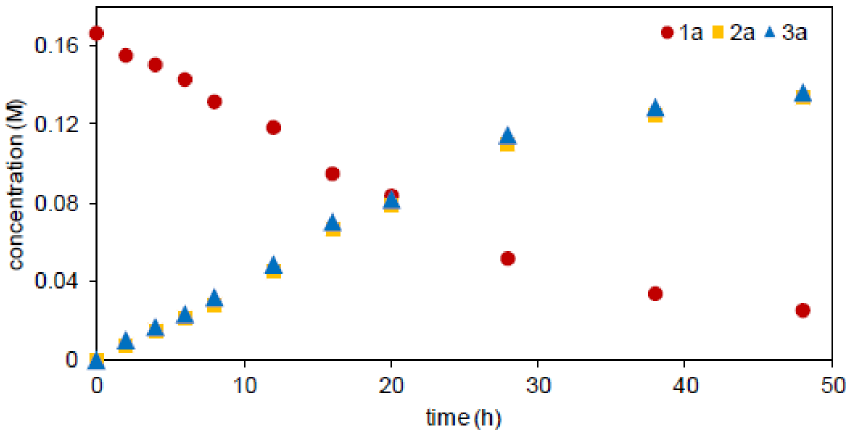

Supplementary Fig 25.
Kinetic profile for the hydrogenolysis of **1a** using **4** under Condition A.

4-2. Determination of rate order of urea **1a** under Condition A

Experiments were performed as follows: A 50 mL stainless steel autoclave, a glass tube, and a stirring bar were dried in an oven at 150 °C, and then cooled inside a glovebox under argon atmosphere. Catalyst **4** (3.1 mg, 5 µmol), 1,3-diphenylurea (**1a**) and THF (3 mL) were added into the glass tube, and the tube was capped with a funnel to prevent evaporation of the solvent. After the glass tube was set in the autoclave and the autoclave was sealed, the autoclave was brought out from the glovebox. The autoclave was degassed three times using H_2 and was pressurized with 1 MPa of H_2 for 5 min with stirring. The reaction mixture was stirred in an isothermal heating block at 130 °C for each reaction time. After cooling to room temperature, H_2 was vented off carefully. To the reaction mixture, DMSO-d_6 (ca. 2 mL) was added to homogenize the reaction mixture, and the same amount of dibromomethane as **1a** was added as an internal standard. The solution (THF/DMSO-d_6 = 3/2, 0.4 mL) was transferred into an NMR tube and diluted with DMSO-d_6 (0.2 mL) for NMR analysis. The conversion of **1a** and the yields of formanilide (**2a**) and aniline (**3a**) were determined by ^{1}H NMR spectroscopy with the internal standard.

Time-courses of each reaction at different concentrations of **1a** (Supplementary Fig 26) were summarized in Supplementary Tabs 8–11 and plotted against reaction time (Supplementary Figs 27–29). From the slope of these plots, initial reaction rates were determined (Supplementary Tab 12). The data of consumption of **1a** were representatively used to make Supplementary Fig 16a. Similar plots using the formation of **2a** or **3a** showed almost the same reaction order (Supplementary Figs 30 and 31).

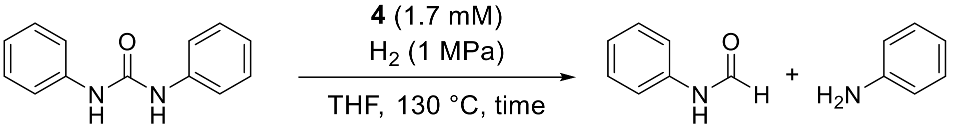

1a **2a** **3a**

(0.042 M, 0.083 M, 0.125 M, or 0.167 M)

Supplementary Fig 26.

Hydrogenolysis of **1a** under various concentrations of **1a**.

Supplementary Tab 8.

Hydrogenolysis of **1a** under various concentrations of **1a** ([**1a**]$_0$ = 0.042 M).

entry	time (s)	**1a** (M)	**2a** (M)	**3a** (M)
1	0	0.042	0	0
2	21600	0.038	0.003	0.004
3	28800	0.036	0.005	0.006
4	43200	0.035	0.007	0.007

Supplementary Tab 9.

Hydrogenolysis of **1a** under various concentrations of **1a** ([**1a**]$_0$ = 0.083 M).

entry	time (s)	**1a** (M)	**2a** (M)	**3a** (M)
1	0	0.083	0	0
2	21600	0.074	0.008	0.008
3	28800	0.073	0.009	0.010
4	43200	0.069	0.014	0.015

Supplementary Tab 10.

Hydrogenolysis of **1a** under various concentrations of **1a** ([**1a**]$_0$ = 0.125 M).

entry	time (s)	**1a** (M)	**2a** (M)	**3a** (M)
1	0	0.125	0	0
2	21600	0.111	0.013	0.013
3	28800	0.106	0.018	0.018
4	43200	0.095	0.028	0.029

Supplementary Tab 11.

Hydrogenolysis of **1a** under various concentrations of **1a** ([**1a**]$_0$ = 0.167 M).

entry	time (s)	**1a** (M)	**2a** (M)	**3a** (M)
1	0	0.167	0	0
2	14400	0.155	0.010	0.012
3	21600	0.148	0.017	0.017
4	28800	0.140	0.025	0.025
5	43200	0.130	0.035	0.035

Supplementary Tab 12.

Initial reaction rates under various initial concentrations of **1a**.

entry	[**1a**]$_0$ (M)	v (**1a**) (M s^{-1})	v (**2a**) (M s^{-1})	v (**3a**) (M s^{-1})
1	4.17×10^{-2}	$-1.6 \pm 0.3 \times 10^{-7}$	$1.6 \pm 0.2 \times 10^{-7}$	$1.6 \pm 0.3 \times 10^{-7}$
2	8.33×10^{-2}	$-3.3 \pm 0.4 \times 10^{-7}$	$3.2 \pm 0.3 \times 10^{-7}$	$3.5 \pm 0.2 \times 10^{-7}$
3	1.25×10^{-1}	$-6.9 \pm 0.3 \times 10^{-7}$	$6.3 \pm 0.2 \times 10^{-7}$	$6.6 \pm 0.4 \times 10^{-7}$
4	1.67×10^{-1}	$-8.7 \pm 0.4 \times 10^{-7}$	$8.3 \pm 0.4 \times 10^{-7}$	$8.2 \pm 0.3 \times 10^{-7}$

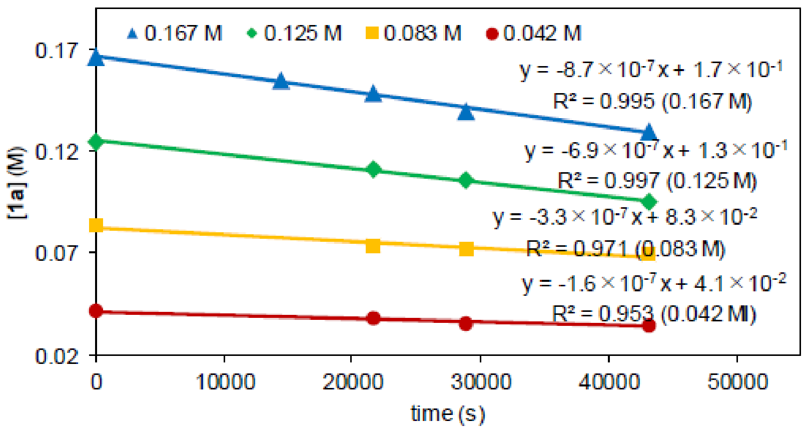

Supplementary Fig 27.

Plots of concentration of **1a** against reaction time under various initial concentrations of **1a**.

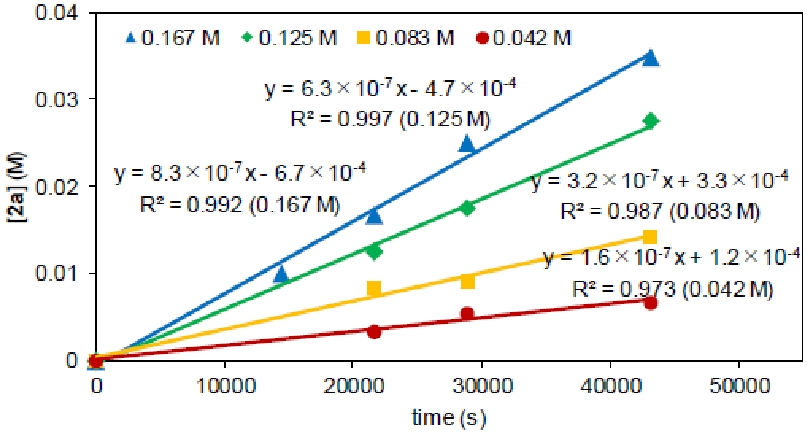

Supplementary Fig 28.

Plots of concentration of **2a** against reaction time under various initial concentrations of **1a**.

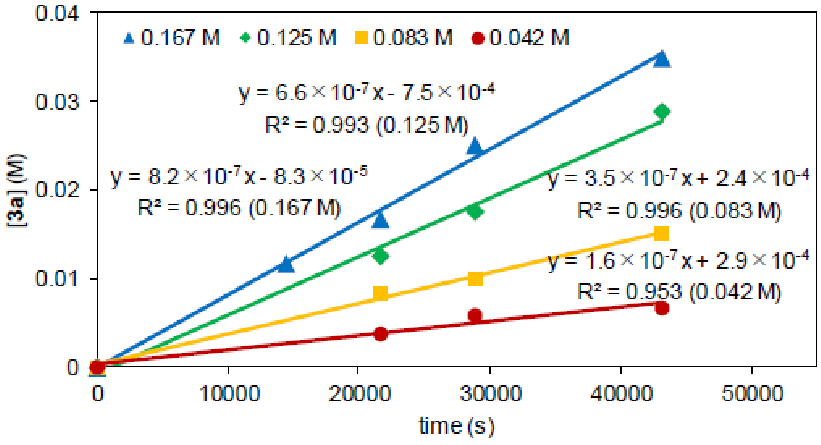

Supplementary Fig 29.

Plots of concentration of **3a** against reaction time under various initial concentrations of **1a**.

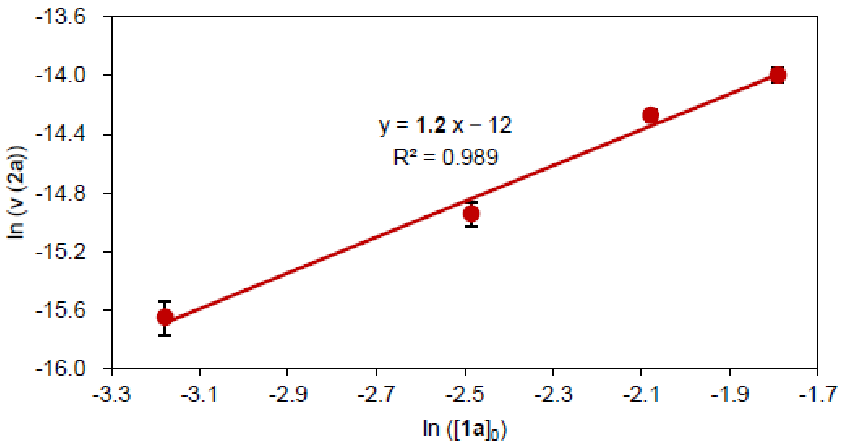

Supplementary Fig 30.

Double logarithm plots of initial reaction rate against initial concentration of **1a** based on formation of **2a**. Error bars represent SE.

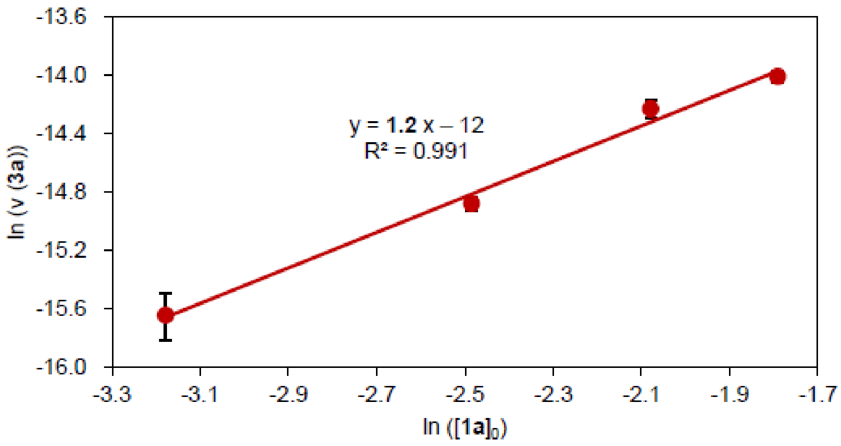

Supplementary Fig 31.

Double logarithm plots of initial reaction rate against initial concentration of **1a** based on formation of **3a**. Error bars represent SE.

4-3. Determination of rate order of H_2 pressure under Condition A

Experiments were performed as follows: A 50 mL stainless steel autoclave, a glass tube, and a stirring bar were dried in an oven at 150 °C, and then cooled inside a glovebox under argon atmosphere. Catalyst **4** (9.4 mg, 15 µmol), 1,3-diphenylurea (**1a**) (106.1 mg, 0.50 mmol) and THF (3 mL) were added into the glass tube, and the tube was capped with a funnel to prevent evaporation of the solvent. After the glass tube was set in the autoclave and the autoclave was sealed, the autoclave was brought out from the glovebox. The autoclave was degassed three times using H_2 and was pressurized with each pressure of H_2 for 5 min with stirring. The reaction mixture was stirred in an isothermal heating block at 130 °C for each reaction time. After cooling to room temperature, H_2 was vented off carefully. To the reaction mixture, DMSO-d_6 (ca. 2 mL) was added to homogenize the reaction mixture, and dibromomethane (86.9 mg, 0.50 mmol) was added as an internal standard. The solution (THF/DMSO-d_6 = 3/2, 0.4 mL) was transferred into an NMR tube and diluted with DMSO-d_6 (0.2 mL) for NMR analysis. The conversion of **1a** and the yields of formanilide (**2a**) and aniline (**3a**) were determined by ^{1}H NMR spectroscopy with the internal standard.

Time-courses of each reaction at different H_2 pressure (Supplementary Fig 32) were summarized in Supplementary Tabs 13–16 and plotted against reaction time (Supplementary Figs 33–35). From the slope of these plots, initial reaction rates were determined (Supplementary Tab 17). The data of consumption of **1a** were representatively used to make Supplementary Fig 16b. Similar plots using the formation of **2a** or **3a** showed almost the same reaction order (Supplementary Figs 36 and 37).

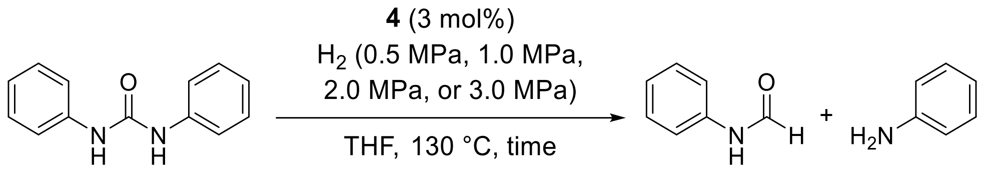

Supplementary Fig 32.

Hydrogenolysis of **1a** under various H_2 pressures.

Supplementary Tab 13.

Hydrogenolysis of **1a** under various H_2 pressures (H_2 pressure = 0.5 MPa).

entry	time (s)	**1a** (M)	**2a** (M)	**3a** (M)
1	0	0.167	0	0
2	14400	0.150	0.015	0.017
3	21600	0.142	0.023	0.025
4	28800	0.133	0.032	0.033

Supplementary Tab 14.

Hydrogenolysis of **1a** under various H_2 pressures (H_2 pressure = 1.0 MPa).

entry	time (s)	**1a** (M)	**2a** (M)	**3a** (M)
1	0	0.167	0	0
2	14400	0.150	0.015	0.017
3	21600	0.143	0.022	0.023
4	28800	0.132	0.028	0.032

Supplementary Tab 15.

Hydrogenolysis of **1a** under various H_2 pressures (H_2 pressure = 2.0 MPa).

entry	time (s)	**1a** (M)	**2a** (M)	**3a** (M)
1	0	0.167	0	0
2	14400	0.150	0.015	0.017
3	21600	0.138	0.023	0.025
4	28800	0.132	0.028	0.032

Supplementary Tab 16.

Hydrogenolysis of **1a** under various H_2 pressures (H_2 pressure = 3.0 MPa).

entry	time (s)	**1a** (M)	**2a** (M)	**3a** (M)
1	0	0.167	0	0
2	14400	0.147	0.017	0.018
3	21600	0.138	0.025	0.023
4	28800	0.132	0.033	0.035

Supplementary Tab 17.

Initial reaction rates under various initial H_2 pressures.

entry	$[H_2]_0$ (MPa)	v (**1a**) ($M\ s^{-1}$)	v (**2a**) ($M\ s^{-1}$)	v (**3a**) ($M\ s^{-1}$)
1	0.5	$-1.2 \pm 0.0 \times 10^{-6}$	$1.10 \pm 0.02 \times 10^{-6}$	$1.2 \pm 0.0 \times 10^{-6}$
2	1.0	$-1.2 \pm 0.1 \times 10^{-6}$	$9.9 \pm 0.3 \times 10^{-7}$	$1.09 \pm 0.03 \times 10^{-6}$
3	2.0	$-1.2 \pm 0.1 \times 10^{-6}$	$1.0 \pm 0.1 \times 10^{-6}$	$1.11 \pm 0.03 \times 10^{-6}$
4	3.0	$-1.2 \pm 0.1 \times 10^{-6}$	$1.2 \pm 0.0 \times 10^{-6}$	$1.2 \pm 0.1 \times 10^{-6}$

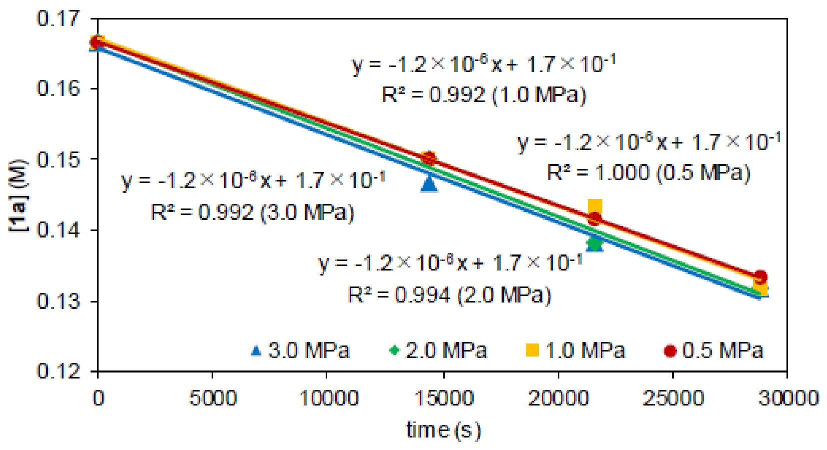

Supplementary Fig 33.

Plots of concentration of **1a** against reaction time under various initial H_2 pressures.

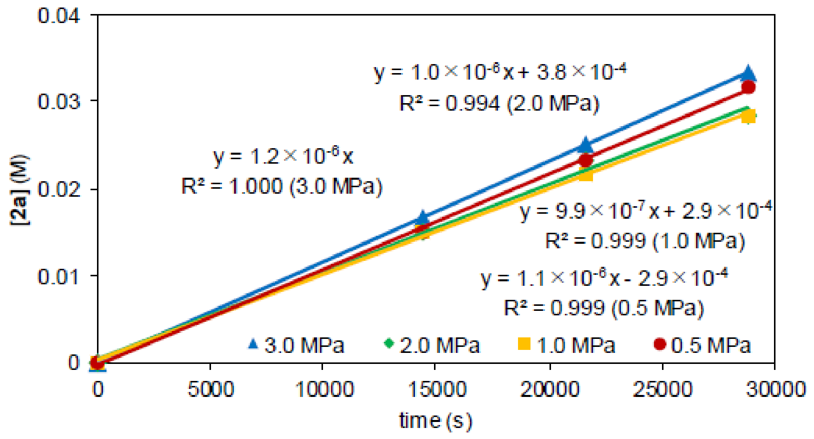

Supplementary Fig 34.

Plots of concentration of **2a** against reaction time under various initial H_2 pressures.

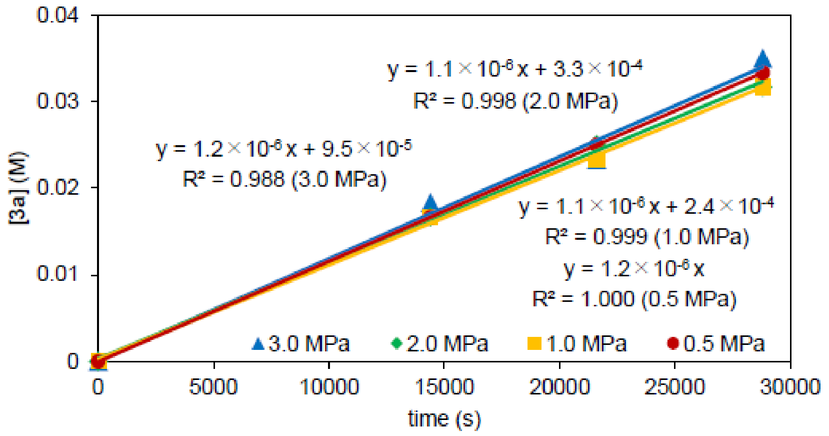

Supplementary Fig 35.

Plots of concentration of **3a** against reaction time under various initial H_2 pressures.

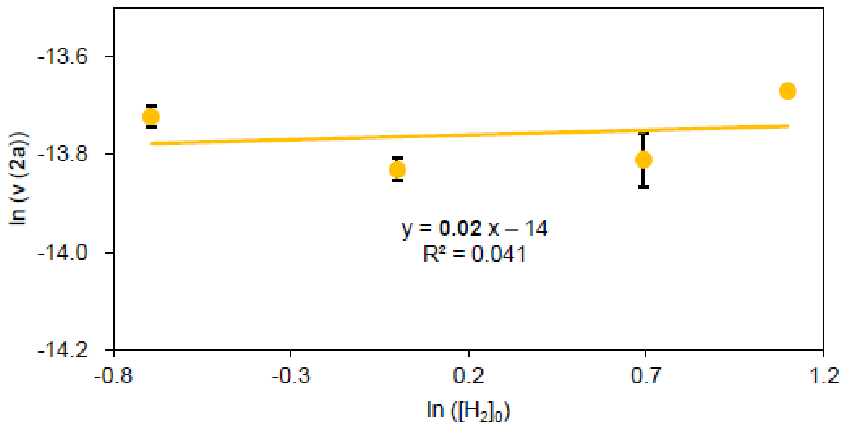

Supplementary Fig 36.

Double logarithm plots of initial reaction rate against initial H_2 pressure based on formation of **2a**. Error bars represent SE.

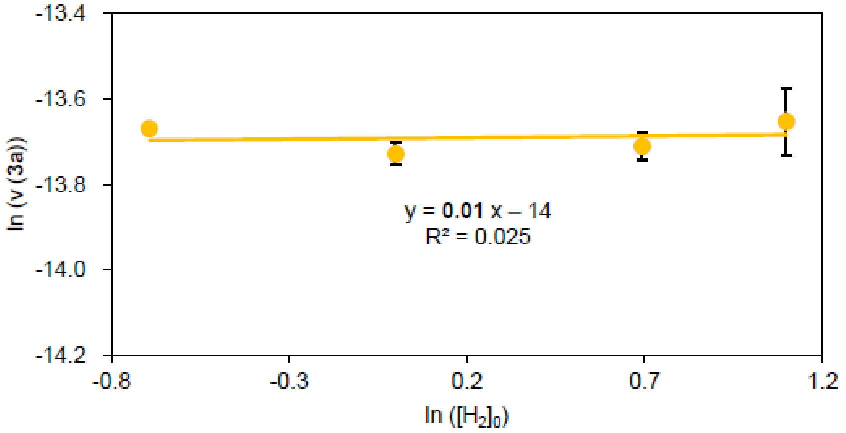

Supplementary Fig 37.

Double logarithm plots of initial reaction rate against initial H_2 pressure based on formation of **3a**. Error bars represent SE.

4-4. Determination of rate order of catalyst **4** under Condition A

Experiments were performed as follows: A 50 mL stainless steel autoclave, a glass tube, and a stirring bar were dried in an oven at 150 °C, and then cooled inside a glovebox under argon atmosphere. Catalyst **4**, 1,3-diphenylurea (**1a**) (106.1 mg, 0.50 mmol) and THF (3 mL) were added into the glass tube, and the tube was capped with a funnel to prevent evaporation of the solvent. After the glass tube was set in the autoclave and the autoclave was sealed, the autoclave was brought out from the glovebox. The autoclave was degassed three times using H_2 and was pressurized with 1 MPa of H_2 for 5 min with stirring. The reaction mixture was stirred in an isothermal heating block at 130 °C for each reaction time. After cooling to room temperature, H_2 was vented off carefully. To the reaction mixture, DMSO-d_6 (ca. 2 mL) was added to homogenize the reaction mixture, and dibromomethane (86.9 mg, 0.50 mmol) was added as an internal standard. The solution (THF/DMSO-d_6 = 3/2, 0.4 mL) was transferred into an NMR tube and diluted with DMSO-d_6 (0.2 mL) for NMR analysis. The conversion of **1a** and the yields of formanilide (**2a**) and aniline (**3a**) were determined by ^{1}H NMR spectroscopy with the internal standard.

Time-courses of each reaction at different concentrations of **4** (Supplementary Fig 38) were summarized in Supplementary Tabs 18–23 and plotted against reaction time (Supplementary Figs 39–41). From the slope of these plots, initial reaction rates were determined (Supplementary Tab 24). The data of consumption of **1a** were representatively used to make Supplementary Fig 16c. Similar plots using the formation of **2a** or **3a** showed almost the same reaction order (Supplementary Figs 42 and 43).

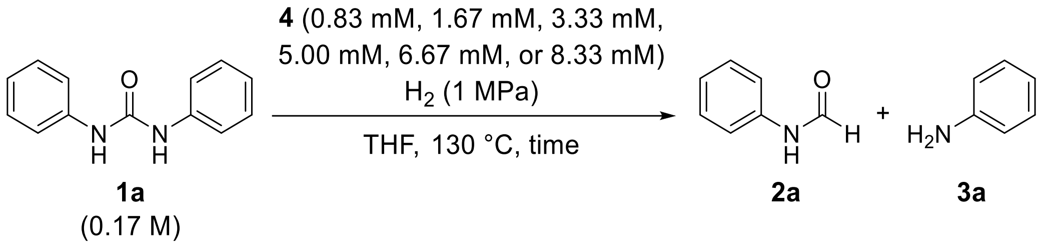

Supplementary Fig 38.

Hydrogenolysis of **1a** under various concentrations of **4**.

Supplementary Tab 18.

Hydrogenolysis of **1a** under various concentrations of **4** ([**4**]$_0$ = 0.83 mM).

entry	time (s)	**1a** (M)	**2a** (M)	**3a** (M)
1	0	0.167	0	0
2	14400	0.158	0.007	0.008
3	28800	0.150	0.015	0.017
4	43200	0.143	0.022	0.023
5	57600	0.137	0.030	0.032
6	72000	0.130	0.035	0.037

Supplementary Tab 19.

Hydrogenolysis of **1a** under various concentrations of **4** ([**4**]$_0$ = 1.67 mM).

entry	time (s)	**1a** (M)	**2a** (M)	**3a** (M)
1	0	0.167	0	0
2	14400	0.155	0.010	0.012
3	21600	0.148	0.017	0.017
4	28800	0.140	0.025	0.025
5	43200	0.130	0.035	0.035

Supplementary Tab 20.

Hydrogenolysis of **1a** under various concentrations of **4** ([**4**]$_0$ = 3.33 mM).

entry	time (s)	**1a** (M)	**2a** (M)	**3a** (M)
1	0	0.167	0	0
2	14400	0.148	0.017	0.017
3	21600	0.137	0.027	0.028
4	43200	0.115	0.048	0.052

Supplementary Tab 21.

Hydrogenolysis of **1a** under various concentrations of **4** ([**4**]$_0$ = 5.00 mM).

entry	time (s)	**1a** (M)	**2a** (M)	**3a** (M)
1	0	0.167	0	0
2	7200	0.155	0.007	0.010
3	14400	0.150	0.015	0.017
4	21600	0.143	0.022	0.023
5	28800	0.132	0.028	0.032
6	43200	0.118	0.045	0.048

Supplementary Tab 22.

Hydrogenolysis of **1a** under various concentrations of **4** ([**4**]$_0$ = 6.67 mM).

entry	time (s)	**1a** (M)	**2a** (M)	**3a** (M)
1	0	0.167	0	0
2	14400	0.148	0.018	0.018
3	21600	0.140	0.025	0.027
4	28800	0.130	0.035	0.037

Supplementary Tab 23.

Hydrogenolysis of **1a** under various concentrations of **4** ([**4**]$_0$ = 8.33 mM).

entry	time (s)	**1a** (M)	**2a** (M)	**3a** (M)
1	0	0.167	0	0
2	14400	0.147	0.017	0.018
3	21600	0.137	0.027	0.028
4	28800	0.130	0.033	0.035

Supplementary Tab 24.

Initial reaction rates under various initial concentrations of **4**.

entry	[**4**]$_0$ (M)	v (**1a**) (M s^{-1})	v (**2a**) (M s^{-1})	v (**3a**) (M s^{-1})
1	8.33×10^{-4}	$-5.1 \pm 0.1 \times 10^{-7}$	$5.0 \pm 0.1 \times 10^{-7}$	$5.2 \pm 0.2 \times 10^{-7}$
2	1.67×10^{-3}	$-8.7 \pm 0.4 \times 10^{-7}$	$8.3 \pm 0.4 \times 10^{-7}$	$8.2 \pm 0.3 \times 10^{-7}$
3	3.33×10^{-3}	$-1.2 \pm 0.1 \times 10^{-6}$	$1.12 \pm 0.05 \times 10^{-6}$	$1.2 \pm 0.1 \times 10^{-6}$
4	5.00×10^{-3}	$-1.1 \pm 0.1 \times 10^{-6}$	$1.04 \pm 0.02 \times 10^{-6}$	$1.09 \pm 0.03 \times 10^{-6}$
5	6.67×10^{-3}	$-1.26 \pm 0.02 \times 10^{-6}$	$1.20 \pm 0.05 \times 10^{-6}$	$1.26 \pm 0.02 \times 10^{-6}$
6	8.33×10^{-3}	$-1.3 \pm 0.1 \times 10^{-6}$	$1.18 \pm 0.05 \times 10^{-6}$	$1.2 \pm 0.1 \times 10^{-6}$

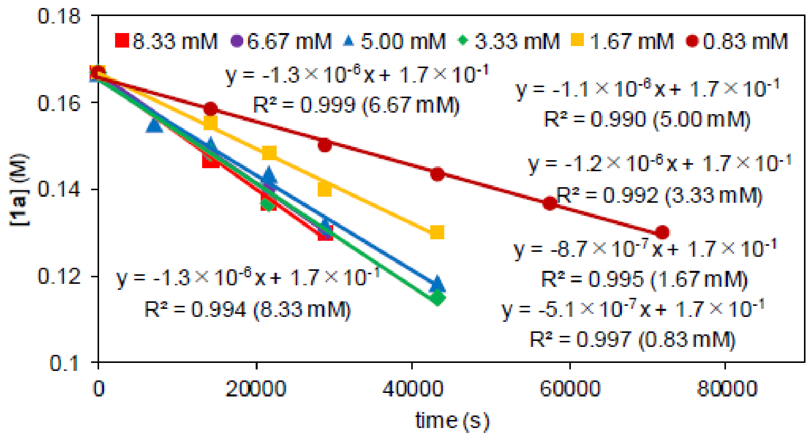

Supplementary Fig 39.

Plots of concentration of **1a** against reaction time under various initial concentrations of **4**.

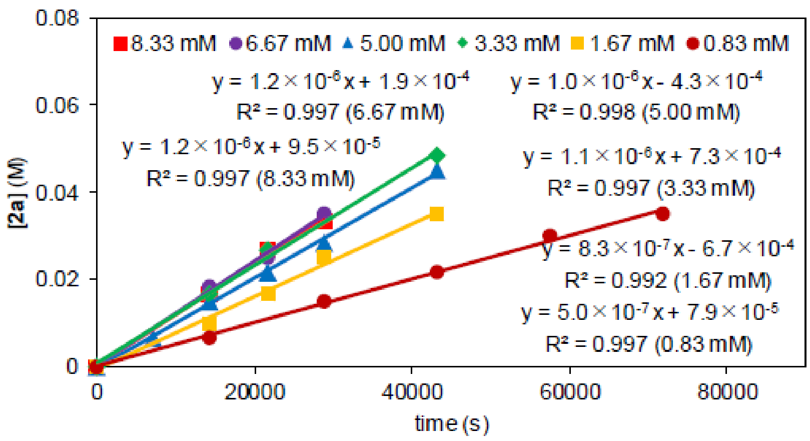

Supplementary Fig 40.

Plots of concentration of **2a** against reaction time under various initial concentrations of **4**.

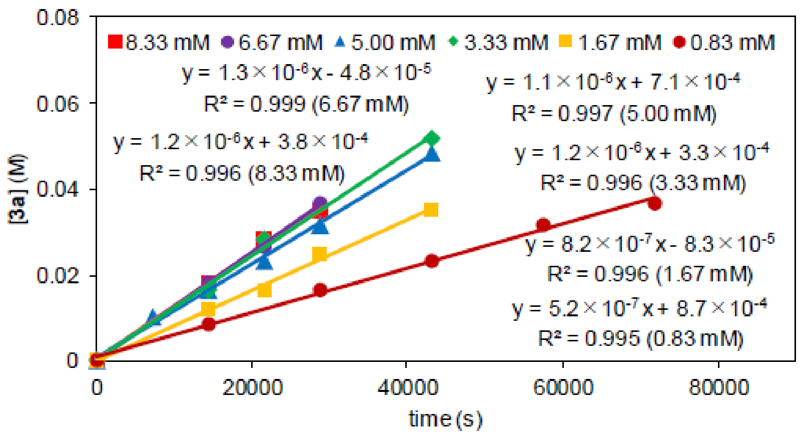

Supplementary Fig 41.

Plots of concentration of **3a** against reaction time under various initial concentrations of **4**.

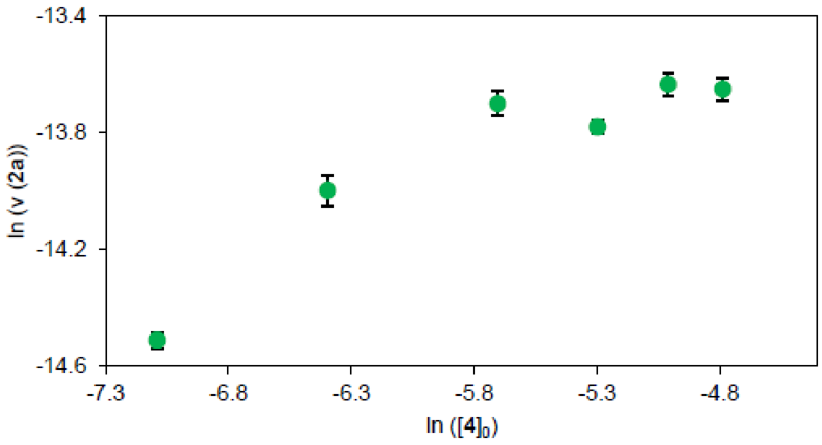

Supplementary Fig 42.

Double logarithm plots of initial reaction rate against initial concentration of **4** based on formation of **2a**. Error bars represent SE.

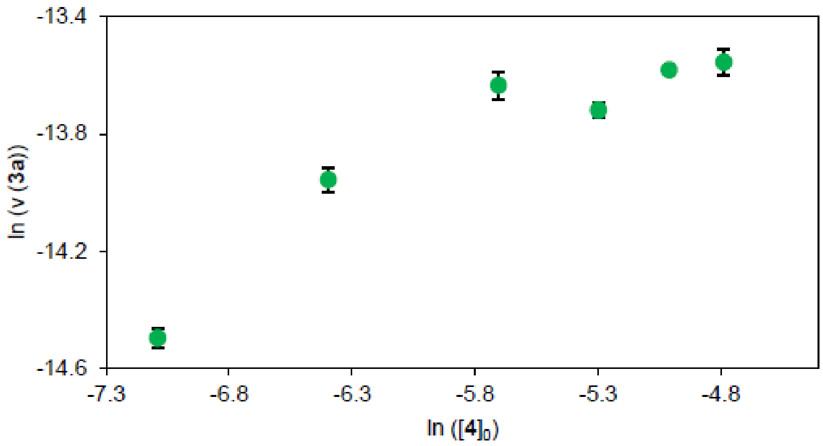

Supplementary Fig 43.

Double logarithm plots of initial reaction rate against initial concentration of **4** based on formation of **3a**. Error bars represent SE.

4-5. Determination of rate order of KO^{t}Bu

Experiments were performed as follows: A 50 mL stainless steel autoclave, a glass tube, and a stirring bar were dried in an oven at 150 °C, and then cooled inside a glovebox under argon atmosphere. Catalyst **4** (3.1 mg, 5 µmol), 1,3-diphenylurea (**1a**) (424.5 mg, 2.0 mmol), KO^{t}Bu, and THF (12 mL) were added into the glass tube, and the tube was capped with a funnel to prevent evaporation of the solvent. After the glass tube was set in the autoclave and the autoclave was sealed, the autoclave was brought out from the glovebox. The autoclave was degassed three times using H_2 and was pressurized with 1 MPa of H_2 for 5 min with stirring. The reaction mixture was stirred in an isothermal heating block at 130 °C for each reaction time. After cooling to room temperature, H_2 was vented off carefully. To the reaction mixture, DMSO-d_6 (ca. 2 mL) and 0.5 M HCl aq. was added to homogenize and neutralize the reaction mixture, and dibromomethane (173.8 mg, 1.0 mmol) was added as an internal standard. The solution (THF/DMSO-d_6 = 6/1, 0.2 mL) was transferred into an NMR tube and diluted with DMSO-d_6 (0.4 mL) for NMR analysis. The conversion of **1a** and the yields of formanilide (**2a**) and aniline (**3a**) were determined by ^{1}H NMR spectroscopy with the internal standard.

Time-courses of each reaction at different concentrations of KO^{t}Bu (Supplementary Fig 44) were summarized in Supplementary Tabs 25–29 and plotted against reaction time (Supplementary Figs 45–47). From the slope of these plots, initial reaction rates were determined (Supplementary Tab 30). The data of consumption of **1a** were representatively used to make Supplementary Fig 16d. Similar plots using the formation of **2a** or **3a** showed almost the same reaction order with higher R^2 value (Supplementary Figs 48 and 49).

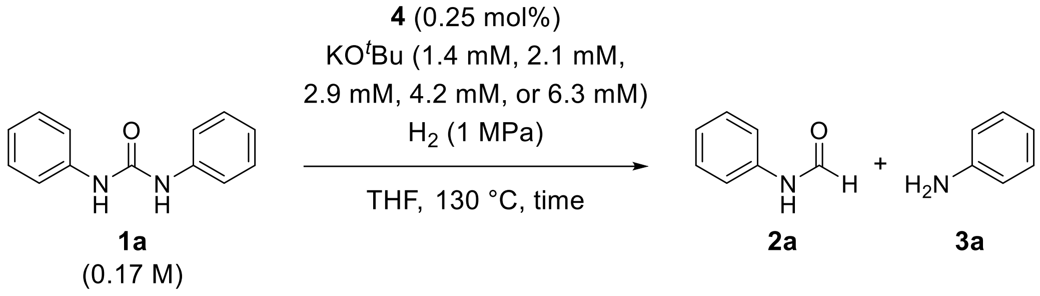

Supplementary Fig 44.

Hydrogenolysis of **1a** under various concentrations of KO^{t}Bu.

Supplementary Tab 25.

Hydrogenolysis of **1a** under various concentrations of KO^{t}Bu ([KO^{t}Bu]$_0$ = 1.4 mM).

entry	time (s)	**1a** (M)	**2a** (M)	**3a** (M)
1	0	0.167	0	0
2	7200	0.147	0.013	0.015
3	14400	0.132	0.030	0.032
4	21600	0.117	0.042	0.045

Supplementary Tab 26.

Hydrogenolysis of **1a** under various concentrations of KO^tBu ($[KO^tBu]_0$ = 2.1 mM).

entry	time (s)	**1a** (M)	**2a** (M)	**3a** (M)
1	0	0.167	0	0
2	7200	0.140	0.017	0.018
3	14400	0.123	0.035	0.035
4	21600	0.112	0.045	0.050

Supplementary Tab 27.

Hydrogenolysis of **1a** under various concentrations of KO^tBu ($[KO^tBu]_0$ = 2.9 mM).

entry	time (s)	**1a** (M)	**2a** (M)	**3a** (M)
1	0	0.167	0	0
2	5400	0.148	0.017	0.018
3	10800	0.137	0.030	0.030
4	14400	0.128	0.037	0.038

Supplementary Tab 28.

Hydrogenolysis of **1a** under various concentrations of KO^tBu ($[KO^tBu]_0$ = 4.2 mM).

entry	time (s)	**1a** (M)	**2a** (M)	**3a** (M)
1	0	0.167	0	0
2	3600	0.155	0.012	0.012
3	7200	0.132	0.027	0.027
4	14400	0.108	0.045	0.050

Supplementary Tab 29.

Hydrogenolysis of **1a** under various concentrations of KO^tBu ($[KO^tBu]_0$ = 6.3 mM).

entry	time (s)	**1a** (M)	**2a** (M)	**3a** (M)
1	0	0.167	0	0
2	5400	0.145	0.013	0.022
3	8100	0.130	0.033	0.037
4	10800	0.117	0.040	0.048

Supplementary Tab 30.

Initial reaction rates under various initial concentrations of KO^tBu.

entry	$[KO^tBu]_0$ (M)	v (**1a**) ($M\ s^{-1}$)	v (**2a**) ($M\ s^{-1}$)	v (**3a**) ($M\ s^{-1}$)
1	1.39×10^{-3}	$-2.3 \pm 0.1 \times 10^{-6}$	$2.0 \pm 0.1 \times 10^{-6}$	$2.1 \pm 0.1 \times 10^{-6}$
2	2.08×10^{-3}	$-2.5 \pm 0.3 \times 10^{-6}$	$2.1 \pm 0.2 \times 10^{-6}$	$2.3 \pm 0.1 \times 10^{-6}$
3	2.92×10^{-3}	$-2.6 \pm 0.2 \times 10^{-6}$	$2.6 \pm 0.2 \times 10^{-6}$	$2.6 \pm 0.2 \times 10^{-6}$
4	4.17×10^{-3}	$-4.2 \pm 0.4 \times 10^{-6}$	$3.1 \pm 0.2 \times 10^{-6}$	$3.5 \pm 0.1 \times 10^{-6}$
5	6.25×10^{-3}	$-4.6 \pm 0.2 \times 10^{-6}$	$3.9 \pm 0.6 \times 10^{-6}$	$4.5 \pm 0.2 \times 10^{-6}$

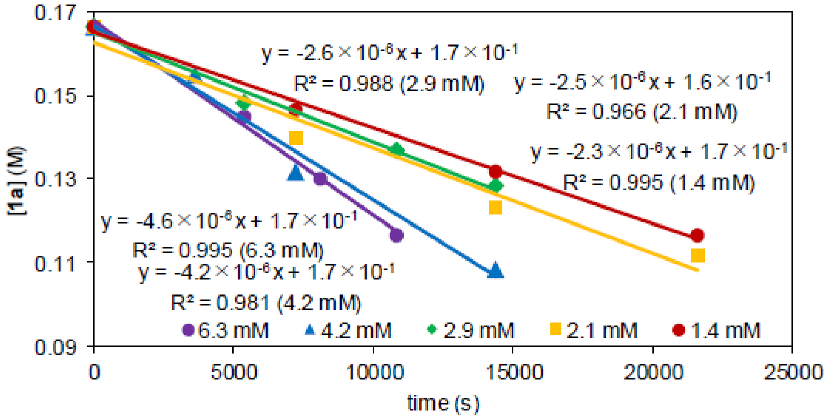

Supplementary Fig 45.

Plots of concentration of **1a** against reaction time under various initial concentrations of KO^tBu.

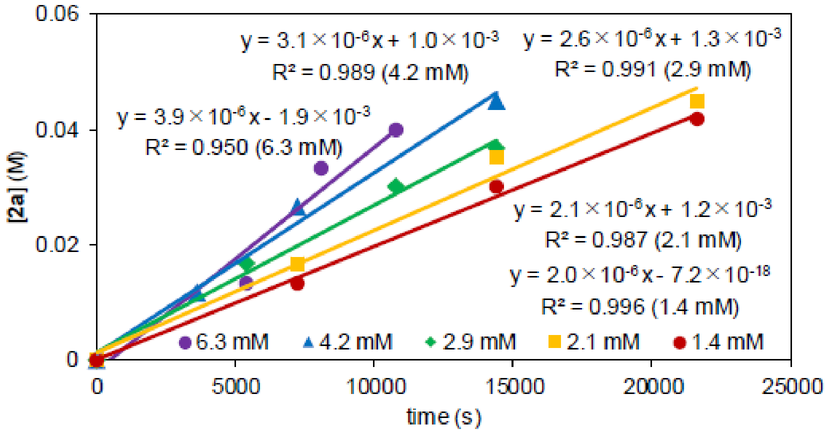

Supplementary Fig 46.

Plots of concentration of **2a** against reaction time under various initial concentrations of KO^tBu.

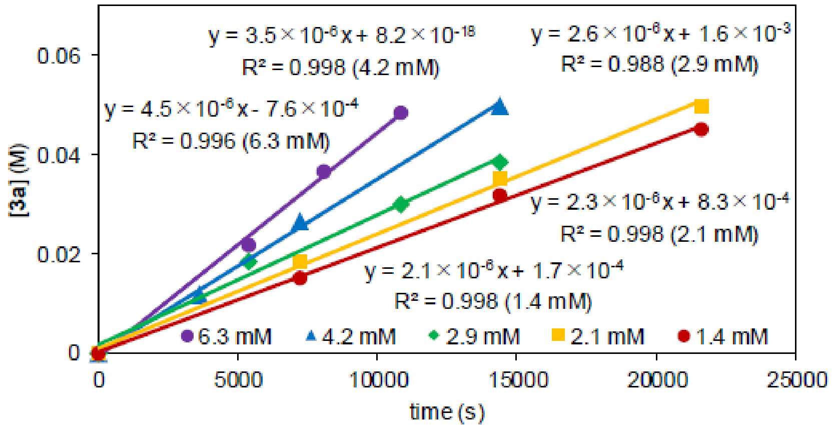

Supplementary Fig 47.

Plots of concentration of **3a** against reaction time under various initial concentrations of KO^{t}Bu.

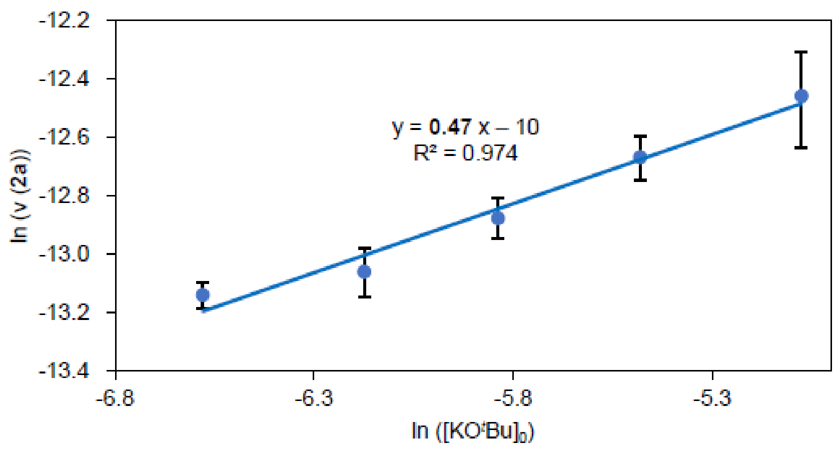

Supplementary Fig 48.
Double logarithm plots of initial reaction rate against initial concentration of KO^{t}Bu based on formation of **2a**. Error bars represent SE.

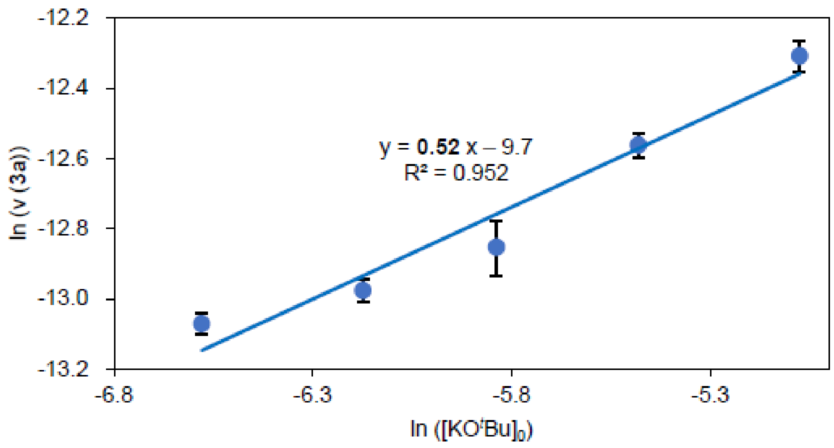

Supplementary Fig 49.
Double logarithm plots of initial reaction rate against initial concentration of KO^{t}Bu based on formation of **3a**. Error bars represent SE.

5. Synthesis and Characterization of Urea Derivatives

5-1. Synthesis of symmetric urea derivatives

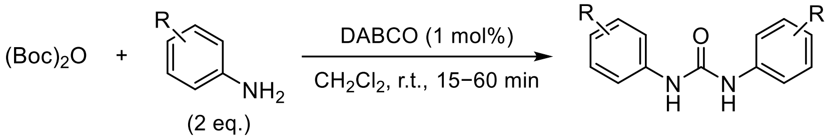

General procedure: Symmetric ureas were synthesized according to the literature[48] with some modifications.

To a solution of aniline (20 mmol) and DABCO (11.2 mg, 0.1 mmol) in CH_2Cl_2 (20 mL) was slowly added $(Boc)_2O$ (2.15 mL, 10 mmol) at ambient temperature. The gas generation and precipitation were observed immediately. After stirring for 15–60 min, white precipitates were formed. To this reaction mixture, hexane (100 mL) was added. The suspension was cooled to 0 °C, and the precipitates were collected by filtration. The white solid was washed with hexane, suspended in 50 mL of boiling MeOH, cooled to 0 °C, and collected by filtration.

1,3-Bis(4-fluorophenyl)urea (**1b**)

The representative general procedure was followed to obtain the title compound as colorless needle crystals (2.12 g, 86%): ^{1}H NMR (400 MHz, DMSO-d_6): δ 8.72 (s, 2H), 7.49 (dd, J = 8.8, 4.8 Hz, 4H), 7.15 (t, J = 8.8 Hz, 4H); ^{13}C NMR (101 MHz, DMSO-d_6): δ 157.1, 156.6 (d, J = 580.6 Hz), 153.7, 137.0, 121.1 (d, J = 7.6 Hz), 116.2 (d, J = 21.9 Hz); ^{19}F NMR (376 MHz, DMSO-d_6): δ −121.4 (s).

These data were consistent with those in literature[48].

1,3-Bis(4-chlorophenyl)urea (**1c**)

The representative general procedure was followed to obtain the title compound as a white solid (1.76 g, 62%): ^{1}H NMR (400 MHz, DMSO-d_6): δ 8.84 (s, 2H), 7.48 (d, J = 8.8 Hz, 4H), 7.32 (d, J = 8.8 Hz, 4H); ^{13}C NMR (101 MHz, DMSO-d_6): δ 152.3, 138.5, 128.6, 125.5, 119.8.

These data were consistent with those in literature[48].

1,3-Bis(3-chlorophenyl)urea (**1d**)

The representative general procedure was followed to obtain the title compound as a white solid (1.50 g, 53%): ^{1}H NMR (400 MHz, DMSO-d_6): δ 8.96 (s, 2H), 7.70 (t, J = 1.6 Hz, 2H), 7.32–7.28 (m, 4H), 7.03 (dt, J = 6.8, 2.0 Hz, 2H); ^{13}C NMR (101 MHz, DMSO-d_6): δ 152.2, 141.0, 133.2, 130.4, 121.7, 117.8, 116.8.

These data were consistent with those in literature[49].

1,3-Bis(2-chlorophenyl)urea (**1e**)

The representative general procedure was followed to obtain the title compound as a white solid (1.18 g, 42%): ^{1}H NMR (400 MHz, DMSO-d_6): δ 9.03 (s, 2H), 8.07 (dm, J = 8.0 Hz, 2H), 7.47 (dm, J = 8.0 Hz, 2H), 7.30 (tm, J = 8.0 Hz, 2H), 7.06 (tm, J = 8.0 Hz, 2H); ^{13}C NMR (101 MHz, DMSO-d_6): δ 152.2, 135.7, 129.3, 127.5, 123.8, 122.8, 122.5.

These data were consistent with those in literature[48].

1,3-Bis(4-bromophenyl)urea (**1f**)

The representative general procedure was followed to obtain the title compound as a white solid (2.31 g, 62%): ^{1}H NMR (400 MHz, DMSO-d_6): δ 8.83 (s, 2H), 7.44 (d like, J = 3.2 Hz, 8H); ^{13}C NMR (101 MHz, DMSO-d_6): δ 152.2, 138.9, 131.5, 120.2, 113.4.

These data were consistent with those in literature[50].

1,3-Bis(4-*tert*-butylphenyl)urea (**1g**)

The title compound was obtained as a white solid based on literature[51] (1.92 g, 30%): ^{1}H NMR (500 MHz, DMSO-d_6): δ 8.51 (s, 2H), 7.35 (d, J = 8.5 Hz, 4H), 7.28 (d, J = 9.0 Hz, 4H), 1.26 (s, 18H); ^{13}C NMR (126 MHz, DMSO-d_6): δ 152.6, 144.0, 137.2, 125.4, 118.0, 33.9, 31.3.

These data were consistent with those in literature[52].

1,3-Bis(4-methoxyphenyl)urea (**1h**)

The representative general procedure was followed to obtain the title compound as a white solid (2.42 g, 89%): ^{1}H NMR (400 MHz, DMSO-d_6): δ 8.36 (s, 2H), 7.34 (d, J = 8.8 Hz, 4H), 6.85 (d, J = 8.8 Hz, 4H), 3.71 (s, 6H); ^{13}C NMR (101 MHz, DMSO-d_6): δ 154.3, 152.9, 132.9, 119.9, 113.9, 55.1.

These data were consistent with those in literature[48].

1,3-Bis(4-dimethylaminophenyl)urea (**1i**)

The representative general procedure was followed to obtain the title compound as a white solid (2.72 g, 91%): ^{1}H NMR (400 MHz, DMSO-d_6): δ 8.05 (s, 2H), 7.24 (d, J = 8.8 Hz, 4H), 6.68 (d, J = 8.8 Hz, 4H), 2.82 (s, 12H); ^{13}C NMR (101 MHz, DMSO-d_6): δ 153.0, 146.2, 129.8, 119.9, 113.1, 40.6.

These data were consistent with those in literature[50].

1,3-Bis(4-cyanophenyl)urea (**1*l***)

The representative general procedure was followed to obtain the title compound as a white solid (0.29 g, 11%): ^{1}H NMR (400 MHz, DMSO-d_6): δ 9.37 (s, 2H), 7.75 (d, J = 8.8 Hz, 4H), 7.64 (d, J = 8.8 Hz, 4H); ^{13}C NMR (101 MHz, DMSO-d_6): δ 151.8, 143.6, 133.3, 119.2, 118.4, 103.8.

These data were consistent with those in literature[48].

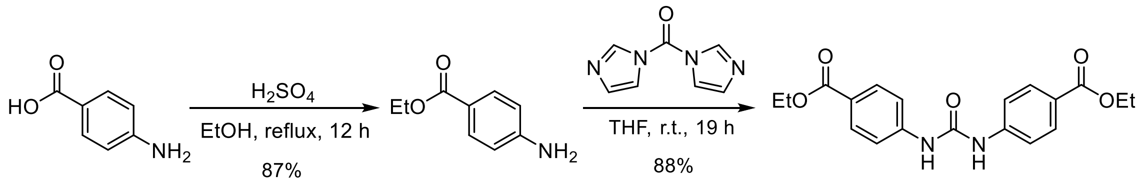

Diethyl 4,4'-(carbonylbis(azanediyl))dibenzoate (**1j**)

To a solution of 4-aminobenzoic acid (7.15 g, 52.1 mmol) in 100 mL EtOH, conc. H_2SO_4 was slowly added. The resulting white suspension was gently refluxed for 12 h to form clear solution. After cooling to r.t., the reaction mixture was poured into a stirring mixture of K_2CO_3 (10.5 g), H_2O (100 mL), and ice (200 mL) to neutralize. The resulting white solid was collected by filtration, washed with H_2O for several times, and dried in vacuum to obtain ethyl 4-aminobenzoate as a white solid (7.46 g, 87%). The product was used for the next step without further purification.

A solution of ethyl 4-aminobenzoate (9.88 g, 59.8 mmol) and carbonyldiimidazole (4.82 g, 29.7 mmol) in dry THF was stirred at r.t. for 19 h. The reaction mixture was concentrated and dissolved in EtOAc. The organic layer was washed with 1M HCl aq. (twice) and brine (twice), dried over Na_2SO_4, filtrated, and concentrated. The crude product was suspended in hot hexane, cooled, and filtrated to obtain the title compound as a pale yellow solid (9.31 g, 88%). Further purification was performed by recrystallization from hot EtOAc: ^{1}H NMR (400 MHz, DMSO-d_6): δ 9.21 (s, 2H), 7.89 (d, J = 8.8 Hz, 4H), 7.60 (d, J = 8.8 Hz, 4H), 4.27 (q, J = 7.2 Hz, 4H), 1.30 (t, J = 7.2 Hz, 6H); ^{13}C NMR (100 MHz, DMSO-d_6): δ 165.4, 151.9, 143.9, 130.3, 123.1, 117.5, 60.3, 14.2.

These data were consistent with those in literature[50].

4,4'-(Carbonylbis(azanediyl))dibenzoic acid

A solution of diethyl 4,4'-(carbonylbis(azanediyl))dibenzoate (3.56 g, 10.0 mmol) and KOH (4.03 g) in 30 mL MeOH was refluxed for 12 h. The resulting white precipitate was filtrated and washed with MeOH. The white solid was dissolved in H_2O and acidified by conc. HCl aq. to form white solid. The white solid was filtrated and washed with H_2O (for three times) to obtain a beige solid, which was

dissolved in a minimal amount of hot DMF and reprecipitated from H_2O to obtain the title compound as a beige solid (2.71 g, 90%). The product was used for the next step without further purification.

4,4'-(Carbonylbis(azanediyl))bis(*N*,*N*-dipropylbenzamide) (**1k**)

A mixture of 4,4'-(carbonylbis(azanediyl))dibenzoic acid (1.50 g, 5.0 mmol), dipropylamine (1.65 mL, 12 mmol), EDCI (2.11 g, 11 mmol), and Et_3N (1.65 mL) in CH_2Cl_2 (20 mL) was stirred at room temperature for 14 h under N_2 atmosphere. The reaction mixture was diluted with EtOAc, washed with H_2O, 1M HCl aq., H_2O, and brine, dried over Na_2SO_4, filtrated, and concentrated. The product was purified by silica gel column chromatography (hexane/EtOAc = 90/10 to 0/100) to obtain the title compound as a white solid (567 mg, 24%): ^{1}H NMR (400 MHz, DMSO-d_6): δ 8.97 (s, 2H), 7.50 (d, *J* = 8.4 Hz, 4H), 7.26 (d, *J* = 8.4 Hz, 4H), 3.3 (br, *J* = 7.2 Hz, 8H), 1.52 (br, 8H), 1.00–0.60 (br d, 12H); ^{13}C NMR (101 MHz, DMSO-d_6): δ 170.4, 152.3, 140.3, 130.6, 127.3, 117.6, 50.2 (br), 46.0 (br), 21.0 (br), 11.1; HRMS (ESI) *m*/*z* calcd. for $C_{27}H_{39}N_4O_3$ ($[M+H]^+$) 467.3022, found 467.3027.

5-2. Synthesis of unsymmetric urea derivatives

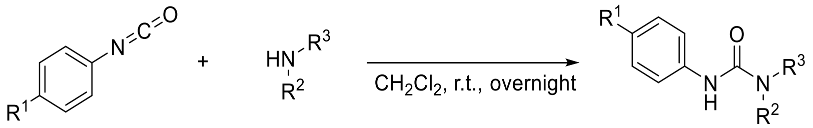

General procedure: To a solution of amine (2 mmol) in CH_2Cl_2 (2 mL), isocyanate (2 mmol) was added at room temperature under N_2 atmosphere. After stirring for overnight, the reaction mixture was concentrated. The resulting crude product was purified by washing with hexane unless otherwise noted.

1-Methyl-1,3-diphenylurea (**1ao**)

The representative general procedure was followed in 10 mmol scale using phenyl isocyanate (1.09 mL, 10.0 mmol) and *N*-methylaniline (1.08 mL, 10.0 mmol) for overnight to obtain the title compound as a white solid (1.916 g, 85%): ^{1}H NMR (400 MHz, DMSO-d_6): δ 8.12 (s, 1H), 7.45–7.38 (m, 4H), 7.32 (d, *J* = 8.0 Hz, 2H), 7.27–7.19 (m, 3H), 6.94 (t, *J* = 7.6 Hz, 1H), 3.27 (s, 3H); ^{13}C NMR (101 MHz, DMSO-d_6): δ 154.7, 144.1, 140.0, 129.2, 128.2, 126.2, 125.7, 122.0, 119.9, 37.5.

These data were consistent with those in literature[53].

1-(4-Chlorophenyl)-1-methyl-3-phenylurea (**1ap**)

The representative general procedure was followed in 3 mmol scale using phenyl isocyanate (0.32 mL, 3.0 mmol) and *N*-methyl-*p*-chloroaniline (427 mg, 3.0 mmol) for overnight to obtain the title compound as a white solid (757 mg, 97%): ^{1}H NMR (400 MHz, $CDCl_3$): δ 7.43 (d, *J* = 8.4 Hz, 2H), 7.28–7.20 (m, 6H), 6.99 (t, *J* = 7.2 Hz, 1H), 6.17 (br, 1H), 3.30 (s, 3H); ^{13}C NMR (101 MHz, $CDCl_3$): δ 154.3, 141.7, 138.8, 133.7, 130.7, 129.0, 128.9, 123.3, 119.5, 37.5; HRMS (ESI) *m*/*z* calcd. for $C_{14}H_{13}ClN_2ONa$ ($[M+Na]^+$) 283.0614, found 283.0604.

1,3-Bis(4-chlorophenyl)-1-methylurea (**1cp**)

The representative general procedure was followed in 3 mmol scale using 4-chlorophenyl isocyanate (469 mg, 3.0 mmol) and *N*-methyl-*p*-chloroaniline (422 mg, 3.0 mmol) for overnight to obtain the title compound as a white solid (866 mg, 98%): ^{1}H NMR (400 MHz, $CDCl_3$): δ 7.44 (d, *J* = 8.4 Hz, 2H), 7.26 (d, *J* = 8.8 Hz, 2H), 7.22 (d, *J* = 8.8 Hz, 2H), 7.17 (d, *J* = 8.8 Hz, 2H), 6.16 (br, 1H), 3.29 (s, 3H); ^{13}C NMR (101 MHz, $CDCl_3$): δ 154.1, 141.4, 137.4, 133.9, 130.8, 129.0, 128.2, 120.7, 37.5.

These data were consistent with those in literature[54].

3-(4-Butylphenyl)-1-(4-chlorophenyl)-1-methylurea (**1qp**)

The representative general procedure was followed in 3 mmol scale using 4-butylphenyl isocyanate (0.53 mL, 3.0 mmol) and *N*-methyl-*p*-chloroaniline (421 mg, 3.0 mmol) for overnight to obtain the title

compound as a white solid (846 mg, 89%): ^{1}H NMR (400 MHz, $CDCl_3$): δ 7.44 (d, *J* = 8.8 Hz, 2H), 7.28 (d, *J* = 8.8 Hz, 2H), 7.18 (d, *J* = 8.4 Hz, 2H), 7.05 (d, *J* = 8.4 Hz, 2H), 6.11 (br, 1H), 3.31 (s, 3H), 2.25 (t, *J* = 7.6 Hz, 2H), 1.55 (quintet, *J* = 7.6 Hz, 2H), 1.32 (sextet, *J* = 7.6 Hz, 2H), 0.90 (t, *J* = 7.6 Hz, 3H); ^{13}C NMR (101 MHz, $CDCl_3$): δ 154.5, 141.8, 138.1, 136.4, 133.6, 130.6, 128.9(4C), 119.7, 37.5, 35.1, 33.9, 22.4, 14.1; HRMS (ESI) *m*/*z* calcd. for $C_{18}H_{21}ClN_2ONa$ ($[M+Na]^+$) 339.1240, found 339.1240.

3-(4-Chlorophenyl)-1-(4-methoxylphenyl)-1-methylurea (1**cr**)

The representative general procedure was followed using 4-chlorophenyl isocyanate (313 mg, 2.0 mmol) and *N*-methyl-*p*-anisidine (273 mg, 2.0 mmol) for overnight to obtain the title compound as a white solid (441 mg, 76%): ^{1}H NMR (400 MHz, $CDCl_3$): δ 7.26–7.21 (m, 4H), 7.17 (d, *J* = 8.8 Hz, 2H), 6.99 (d, *J* = 8.8 Hz, 2H), 6.19 (s, 1H), 3.85 (s, 3H), 3.29 (s, 3H); ^{13}C NMR (101 MHz, $CDCl_3$): δ 159.4, 154.7, 137.8, 135.3, 129.1, 128.9, 127.8, 120.5, 115.8, 55.8, 37.7.

These data were consistent with those in literature[54].

3-(4-Butylphenyl)-1-(4-methoxylphenyl)-1-methylurea (**1qr**)

The representative general procedure was followed using 4-butylphenyl isocyanate (0.35 mL, 2.0 mmol) and *N*-methyl-*p*-anisidine (273 mg, 2.0 mmol) for overnight to obtain the title compound as a white solid (409 mg, 65%): ^{1}H NMR (400 MHz, $CDCl_3$): δ 7.25 (d, *J* = 8.8 Hz, 2H), 7.18 (d, *J* = 8.4 Hz, 2H), 7.04 (d, *J* = 8.4 Hz, 2H), 6.98 (d, *J* = 8.8 Hz, 2H), 6.14 (br, 1H), 3.86 (s, 3H), 3.30 (s, 3H), 2.53 (d, *J* = 7.6 Hz, 2H), 1.54 (quintet, *J* = 7.6 Hz, 2H), 1.32 (sextet, *J* = 7.6 Hz, 2H), 0.90 (t, *J* = 7.6 Hz, 3H); ^{13}C NMR (101 MHz, $CDCl_3$): δ 159.2, 155.1, 137.6, 136.7, 135.7, 129.1, 128.8, 119.5, 115.6, 55.7, 37.6, 35.1, 33.9, 22.4, 14.1; HRMS (ESI) *m*/*z* calcd. for $C_{19}H_{25}N_2O_2$ ($[M+H]^+$) 313.1916, found 313.1912.

N-Phenylmorpholine-4-carboxamide (**1as**)

The representative general procedure was followed in 10 mmol scale using phenyl isocyanate (1.09 mL, 10.0 mmol) and morpholine (0.87 mL, 10.0 mmol) for overnight to obtain the title compound as a white solid (1.93 g, 94%): ^{1}H NMR (400 MHz, DMSO-d_6): δ 8.51 (s, 1H), 7.46 (d, *J* = 8.4 Hz, 2H), 7.23 (t, *J* = 8.0 Hz, 2H), 6.94 (t, *J* = 7.6 Hz, 1H), 3.61 (t, *J* = 4.8 Hz, 4 H), 3.42 (t, *J* = 4.8 Hz, 4H); ^{13}C NMR (101 MHz, DMSO-d_6): δ 155.1, 140.3, 128.3, 121.8, 119.6, 66.0, 44.1.

These data were consistent with those in literature[55].

5-3. Synthesis of polyurea **17**

To a colorless solution of methylenediphenyl 4,4'-diisocyanate (2.50 g, 10 mmol) in dehydrated DMF (80 mL), a solution of *N*,*N*'-dimethyl-1,6-diaminohexane (**16**) (1.74 mL, 10 mmol) in dehydrated DMF (20 mL) was dropped for 6 min at 80 °C under N_2 atmosphere. After stirring for 17 h at 80 °C, to the resulting yellow solution, water (5 mL) was added. After stirring for 30 min at room temperature, the resulting solution was dried *in vacuo* for 3 h at 60 °C. The residue was dissolved in DMF (10 mL) and reprecipitated with THF (200 mL) twice. The obtained solid was dried *in vacuo* for 10 h at 100 °C to afford polyurea resin **17** as an off-white solid (1.92 g, 49%, 96.1% purity, including 2.2% of polyurea resin **30**, 0.7% of *N*,*N*'-dimethyl-1,6-diaminohexane (**16**), 0.5% of 4,4'-diaminodiphenylmethane (**20**), and 0.5% of DMF): ^{1}H NMR (500 MHz, DMSO-d_6): δ 8.09 (s, 2H), 7.34 (d, *J* = 8.5 Hz, 4H), 7.02 (d, *J* = 8.0 Hz, 4H), 3.75 (s, 2H), 3.25 (t, *J* = 6.5 Hz, 4H), 2.88 (s, 6H), 1.46 (br, 4H), 1.25 (br, 4H); ^{13}C NMR (126 MHz, DMSO-d_6): δ 155.3, 138.5, 134.7, 128.3, 120.0, 47.9, 39.9 (overlapped with solvent peaks and confirmed by DEPT analysis), 34.2, 27.3, 26.0; molecular weight (M_n): 64 × 10^3 (PDI = 1.4).

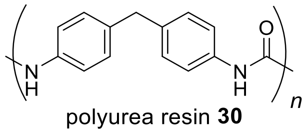

polyurea resin **30**

5-4. Synthesis of authentic samples of products in the hydrogenolysis of polyurea **17**

N,*N'*-(Hexane-1,6-diyl)bis(*N*-methylformamide) (**21**)

To a solution of *N*,*N'*-dimethyl-1,6-diaminohexane (**16**) (1.4 mL, 8 mmol) in THF (8 mL) was added ethyl formate (6.4 mL, 79 mmol) at room temperature, and the mixture was stirred under reflux for 5 h. The solvent was removed under reduced pressure and the residue was purified by silica gel column chromatography with CH_2Cl_2/MeOH (9:1) as an eluent to obtain the title compound as a white solid (1.16 g, 72%): ^{1}H NMR (500 MHz, $CDCl_3$): δ 8.00 (s, 2H), 3.31–3.18 (m, 4H), 2.90–2.82 (m, 6H), 1.56–1.48 (m, 4H), 1.33–1.23 (m, 4H); ^{13}C NMR (126 MHz, $CDCl_3$): δ 162.7, 162.65, 162.60, 162.57, 49.54, 49.48, 44.0, 43.9, 34.57, 34.55, 29.5, 28.0, 26.60, 26.58, 26.44, 26.37, 26.1; HRMS (ESI) *m/z* calcd. for $C_{10}H_{20}N_2O_2Cs$ ($[M+Cs]^+$) 333.0579, found 333.0595.

Multi isomers were observed.

N-Methyl-*N*-(6-(methylamino)hexyl)formamide (**22**)

The title compound was obtained as a colorless oil based on literature[56] (0.97 g, 38%): ^{1}H NMR (500 MHz, $CDCl_3$): δ 8.01 (d, *J* = 5.5 Hz, 1H), 3.32–3.19 (m, 2H), 2.90–2.83 (m, 3H), 2.60–2.56 (m, 2H), 2.43 (d, *J* = 2.0 Hz, 3H), 2.30 (br, 1H), 1.58–1.47 (m, 4H), 1.38–1.24 (m, 4H); ^{13}C NMR (126 MHz, $CDCl_3$): δ 162.8, 162.6, 51.8, 51.7, 49.6, 44.1, 36.3, 36.2, 34.6, 29.53, 29.51, 29.4, 28.0, 27.0, 26.7, 26.3; MS (EI) *m/z* (relative intensity, %): 172 ($[M]^+$, 0.5), 142 (6), 100 (19), 86 (10), 73 (7), 72 (8), 58 (6), 44 (100).

Multi isomers were observed.

6. Synthesis and Characterization of Catalysts

(1,5-Cyclooctadiene)[2-(2-diphenylphosphinophenyl)pyrrolido]iridium(I) (**4**)

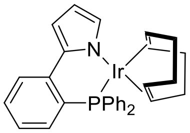

To a solution of 2-(2-diphenylphosphinophenyl)pyrrole (**5**) (163.7 mg, 0.50 mmol) in THF (9 mL), sodium hydride (oil free, 90% purity, 20 mg, 0.75 mmol) was added at room temperature. After stirring for 16 h at room temperature, the resulting light yellow solution was filtered through a membrane filter. To a solution of chlorobis(1,5-cyclooctadiene)iridium dimer (167.9 mg, 0.25 mmol) in THF (2 mL), the filtrate was added at room temperature, and stirred at the same temperature for 22 h to give an orange solution. The solvent was removed under reduced pressure. The residue was washed with hexane and extracted with toluene. The solvent was removed under reduced pressure to afforded complex **4** as orange solids (294.9 mg, 0.47 mmol, 94%). Further purification was performed by recrystallization from THF/hexane. Crystals suitable for X-ray crystallography were obtained by recrystallization from benzene/pentane: ^{1}H NMR (500 MHz, C_6D_6): δ 7.87 (dd, *J* = 8.0, 5.0 Hz, 1H), 7.52–7.48 (m, 4H), 7.22–7.19 (m, 2H), 7.11 (dd, *J* = 7.8, 7.8 Hz, 1H), 6.96–6.89 (m, 7H), 6.74 (dd, *J* = 7.5, 7.5 Hz, 1H), 6.60 (dd, *J* = 2.8, 2.8 Hz, 1H), 5.46 (d, *J* = 4.0 Hz, 2H), 2.82 (d, *J* = 3.0 Hz, 2H), 2.23–2.14 (m, 2H), 2.07–1.98 (m, 2H), 1.76–1.64 (m, 4H); ^{31}P NMR (202 MHz, C_6D_6): δ 16.5; ^{13}C NMR (126 MHz, C_6D_6): δ 145.3 (d, *J* = 16.4 Hz), 137.0 (d, *J* = 9.1 Hz), 134.7 (d, *J* = 11.0 Hz), 132.0 (d, *J* = 3.5 Hz), 131.2 (d, *J* = 2.6 Hz), 131.1, 130.6, 130.5 (d, *J* = 2.6 Hz), 130.1, 128.6 (d, *J* = 10.0 Hz), 128.3 (overlapped with solvent peaks and confirmed by DEPT analysis, d, *J* = 10.5 Hz), 123.5 (d, *J* = 7.2 Hz), 117.8 (d, *J* = 51.9 Hz), 112.2 (d, *J* = 95.5 Hz), 91.9 (d, *J* = 12.9 Hz), 55.8, 33.3 (d, *J* = 3.7 Hz), 29.6 (d, *J* = 1.9 Hz); m.p.: no clear melting point was observed upon heating until 234 ºC; Elem. Anal. (M+THF) calcd. for $C_{34}H_{37}IrNOP$ C, 58.43; H, 5.34; N, 2.00; found C, 58.29; H, 5.13; N, 2.21.

NMR spectra of complex **4** are shown in Supplementary Figs 50–52.

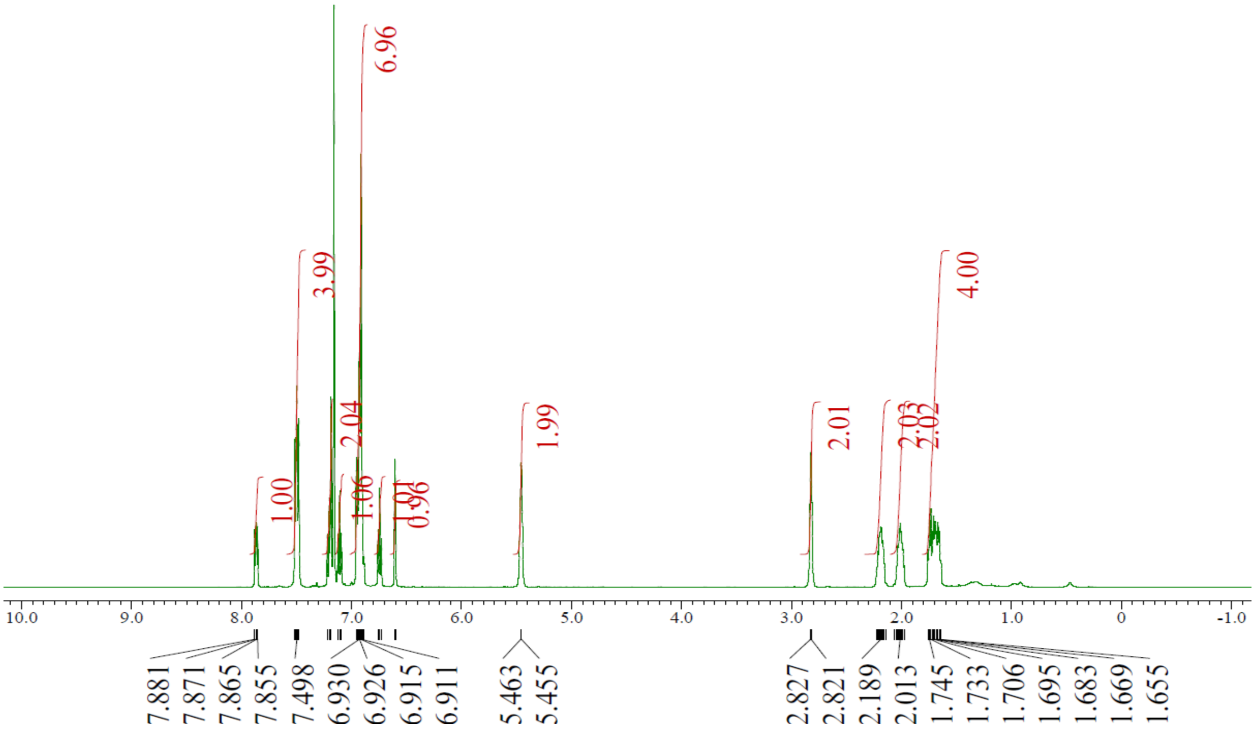

Supplementary Fig 50.

^{1}H NMR spectrum (500 MHz, C_6D_6) of complex **4**.

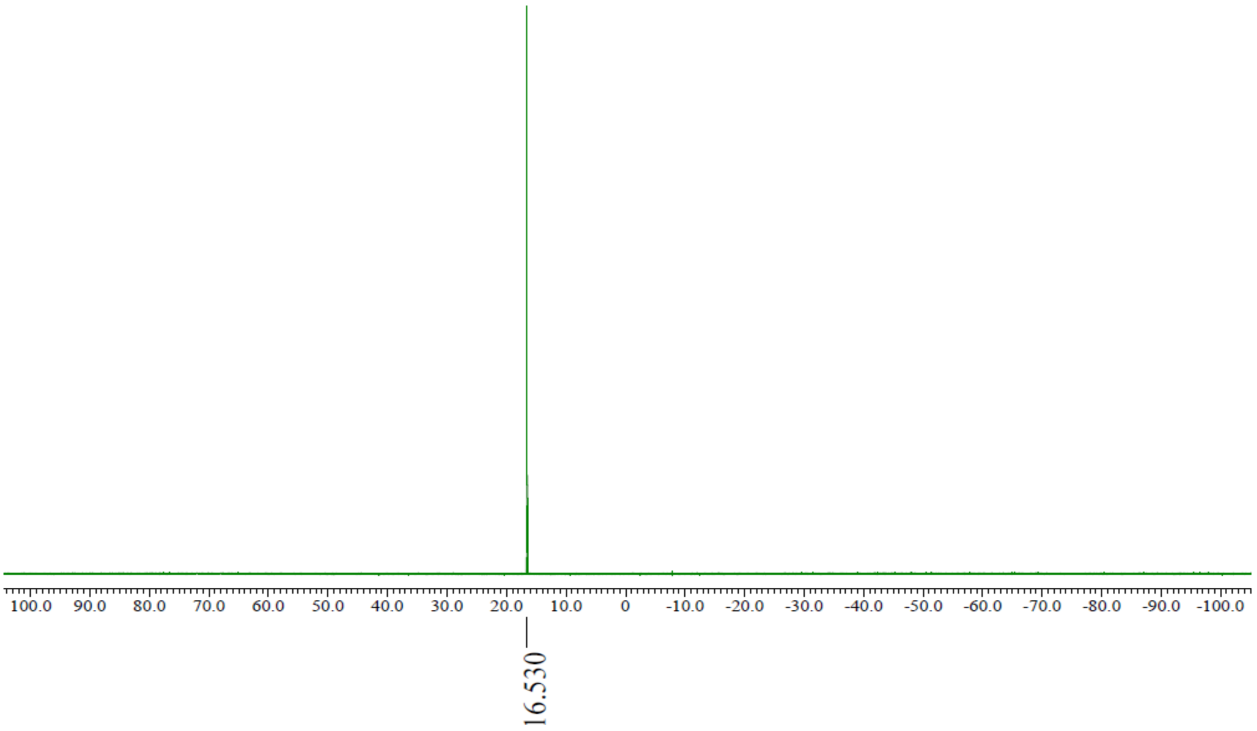

Supplementary Fig 51.

^{31}P NMR spectrum (202 MHz, C_6D_6) of complex **4**.

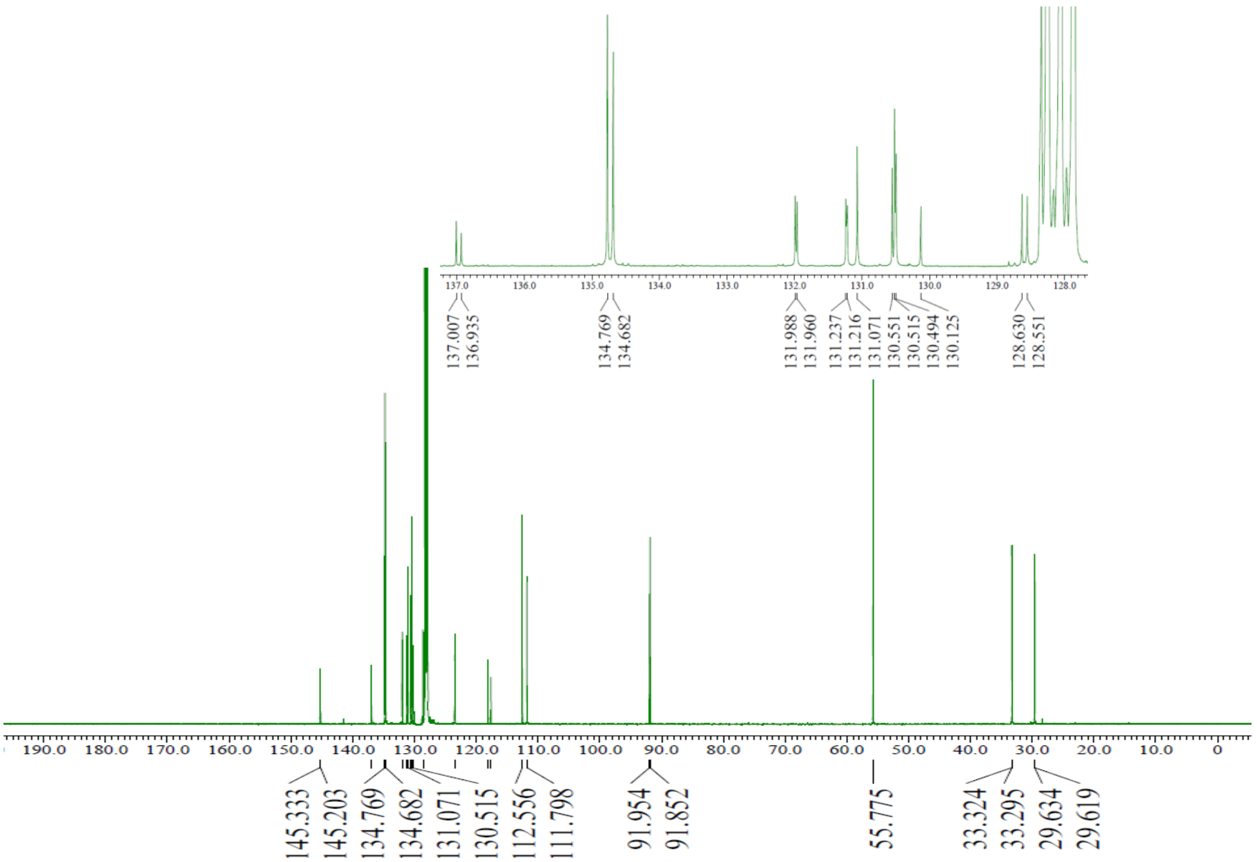

Supplementary Fig 52.
^{13}C NMR spectrum (126 MHz, C_6D_6) of complex **4**.

2-(2-Diphenylphosphinophenyl)benzimidazole (the ligand of complex **6**)

2-(2-Diphenylphosphinophenyl)benzimidazole ligand was synthesized according to the literature[57] with some modifications.

To a solution of (2-(benzoimidazol-2-yl)phenyl)diphenylphosphine oxide (252.7 mg, 0.64 mmol) in toluene (6.4 mL), trichlorosilane (566.8 mg, 4.18 mmol) and anhydrous triethylamine (479.9 mg, 4.74 mmol) were added at 0 °C. The solution was refluxed for 17 h at 130 °C to give a white suspension and pale yellow precipitates. After the solvent and excess reagents were removed under reduced pressure, degassed saturated $NaHCO_3$ aq. (10 mL) was added. The solution was extracted with dichloromethane (8 mL, three times) under N_2 atmosphere. The dichloromethane solution was concentrated under reduced pressure. The obtained solid was washed with hexane (5 mL) to give a white solid (235.8 mg, 0.62 mmol, 97%). ^1H NMR (500 MHz, DMSO-d_6): δ 12.7 (s, 1H), 7.88 (dd, J = 7.5, 3.5 Hz, 1H), 7.56 (t, J = 5.0 Hz, 1H), 7.49–7.43 (m, 3H), 7.35–7.30 (m, 6H), 7.23–7.17 (m, 5H), 7.14–7.11 (m, 1H), 7.04–7.02 (dd, J = 7.5, 3.5 Hz, 1H); ^{31}P NMR (202 MHz, DMSO-d_6): δ −10.7; ^{13}C NMR (126 MHz, DMSO-d_6): δ 151.2, 143.2, 138.4 (d, J = 11.8 Hz), 137.6 (d, J = 23.7 Hz), 135.5 (d, J = 23.7 Hz), 134.4 (d, J = 18.3 Hz), 133.3 (d, J = 20.0 Hz), 129.3, 128.8, 128.4 (d, J = 7.3 Hz), 122.5, 121.3, 119.0, 111.3.

NMR spectra of the ligand are shown in Supplementary Figs 53–55.

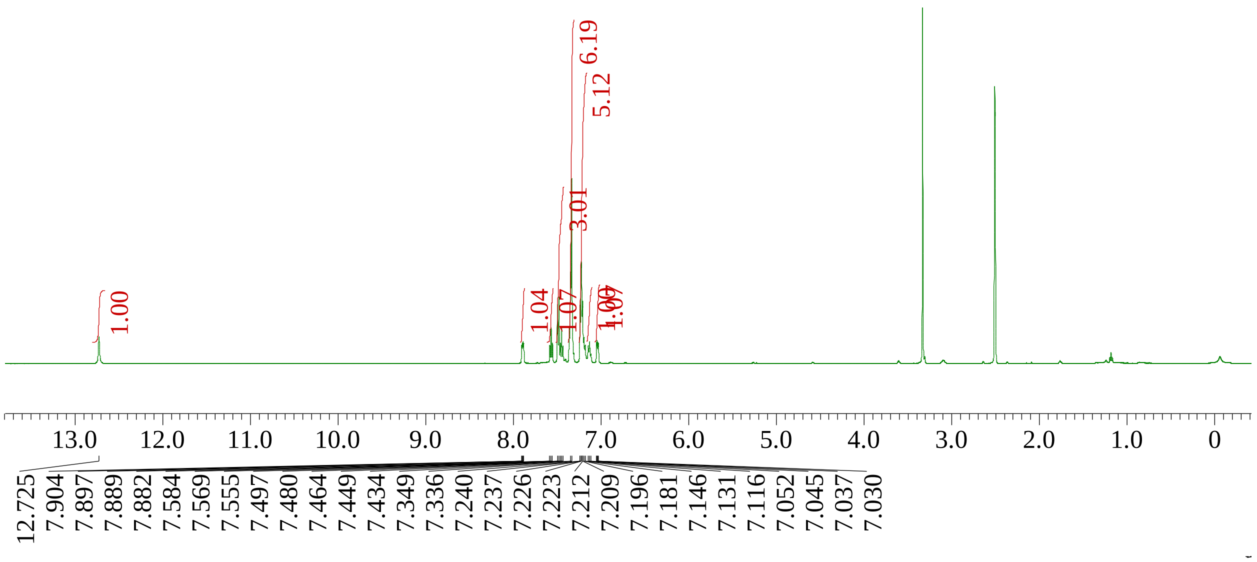

Supplementary Fig 53.
^{1}H NMR spectrum (500 MHz, DMSO-d_6) of 2-(2-diphenylphosphinophenyl)benzimidazole.

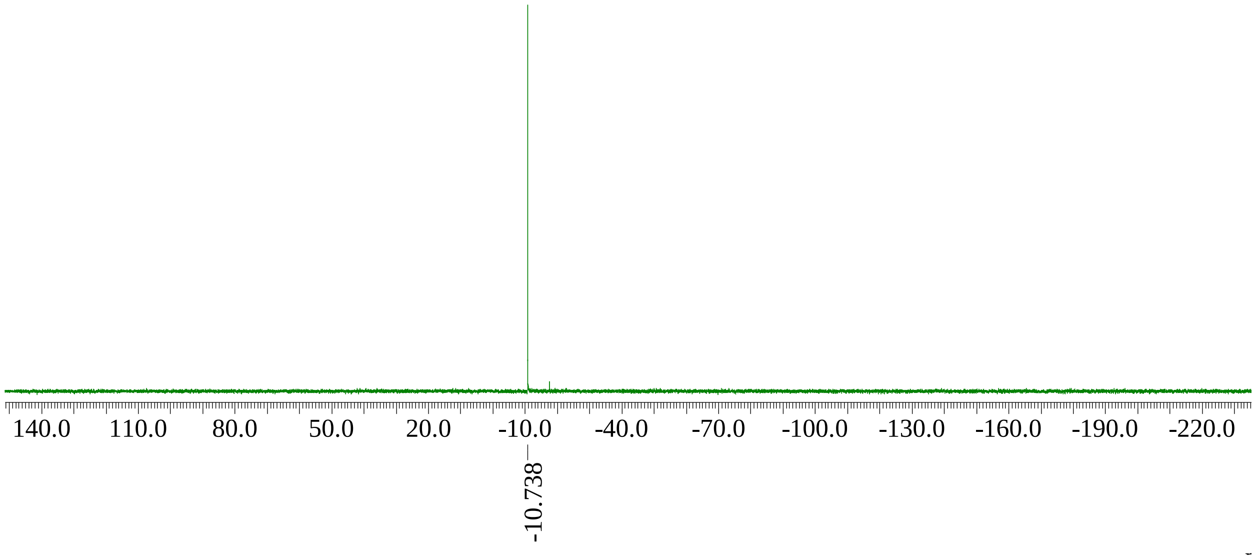

Supplementary Fig 54.
^{31}P NMR spectrum (202 MHz, DMSO-d_6) of 2-(2-diphenylphosphinophenyl)benzimidazole.

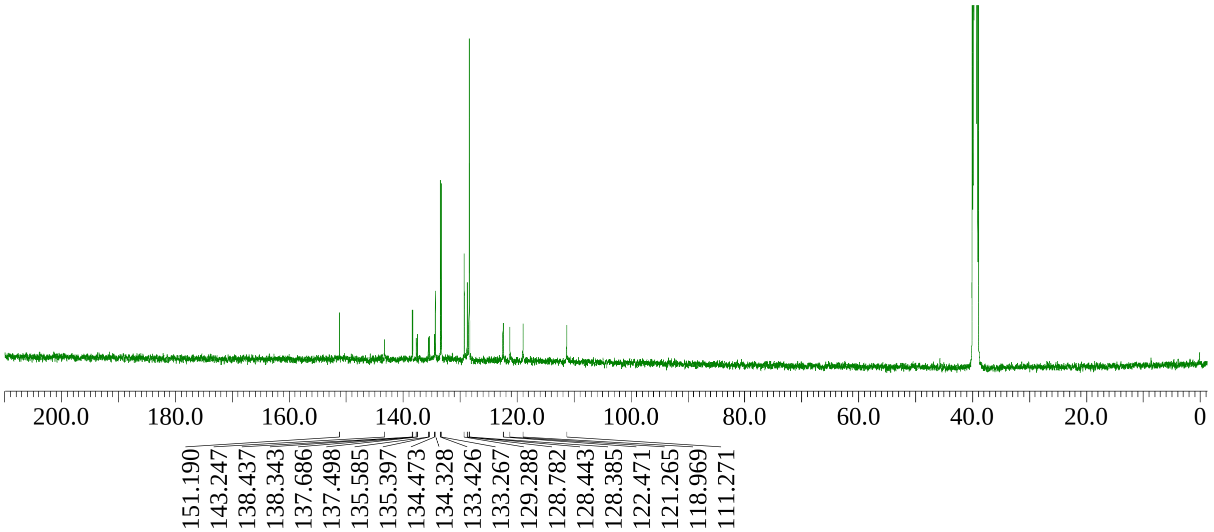

Supplementary Fig 55.
^{13}C NMR spectrum (500 MHz, DMSO-d_6) of 2-(2-diphenylphosphinophenyl)benzimidazole.

(1,5-Cyclooctadiene)[2-(2-diphenylphosphinophenyl)benzimidazolido]iridium(I) (**6**)

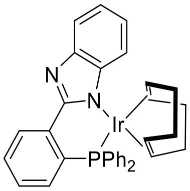

To a solution of 2-(2-diphenylphosphinophenyl)benzimidazole (127.6 mg, 0.34 mmol) in THF (9 mL), sodium hydride (oil free, 90% purity, 13.5 mg, 0.51 mmol) was added at room temperature. After stirring for 24 h at room temperature, the resulting light yellow solution was filtered through a membrane filter. To the filtrate, chlorobis(1,5-cyclooctadiene)iridium dimer (113.2 mg, 0.17 mmol) was added at room temperature, and stirred at the same temperature for 24 h to give an orange solution. The solvent was removed under reduced pressure. The residue was extracted with hexane to afford dark red solid. The obtained red solid was dissolved in a small amount of toluene and charged on a short pad of basic alumina. The alumina column was washed with ca. 2 CV of Et_2O and then eluted with 4 CV of THF to obtain complex **6** as an orange solid after concentration (46.1 mg, 0.068 mmol, 23%): ^{1}H NMR (500 MHz, C_6D_6): δ 9.32–9.29 (m, 1H), 8.11 (d, *J* = 7.5 Hz, 1H), 7.97 (d, *J* = 8.0 Hz, 1H), 7.44–7.41 (m, 4H), 7.32–7.27 (m, 1H), 7.24–7.20 (m, 2H), 6.89–6.80 (m, 7H), 5.36 (br, 2H), 3.11 (d, *J* = 2.0 Hz, 2H), 2.09–2.06 (m, 2H), 1.91–1.83 (m, 2H), 1.60–1.51 (m, 4H), the one remaining proton peak is overlapped with the solvent peak; ^{31}P NMR (202 MHz, C_6D_6): δ 22.3; ^{13}C NMR (126 MHz, C_6D_6): δ 156.0 (d, *J* = 6.4Hz), 150.3, 145.7, 142.5 (d, *J* = 13.9 Hz), 134.6 (d, *J* = 11.1 Hz), 132.1 (d, *J* = 9.2 Hz), 131.4(d, *J* = 1.9 Hz), 130.8 (d, *J* = 1.9 Hz), 130.7 (d, *J* = 2.8 Hz), 129.5 (d, *J* = 52.5 Hz), 128.4 (overlapped with solvent peaks and confirmed by DEPT analysis, d, *J* = 10.5 Hz), 127.6 (d, *J* = 7.3 Hz), 123.0 (d, *J* = 49.6 Hz), 121.3, 120.3 (d, *J* = 8.3 Hz), 116.1, 92.0 (d, *J* = 12.0 Hz), 58.5, 33.0, 30.2; m.p.: no clear melting point was observed upon heating until 227 ºC; HRMS (ESI) *m/z* calcd. for $C_{33}H_{30}IrN_2P$ ($[M]^+$) 678.1776, found 678.1747; Elem. Anal. calcd. for $C_{33}H_{30}N_2PIr$ C, 58.48; H, 4.46; N, 4.13; found C, 58.89; H, 4.69; N, 3.74.

NMR spectra of complex **6** are shown in Supplementary Figs 56–58.

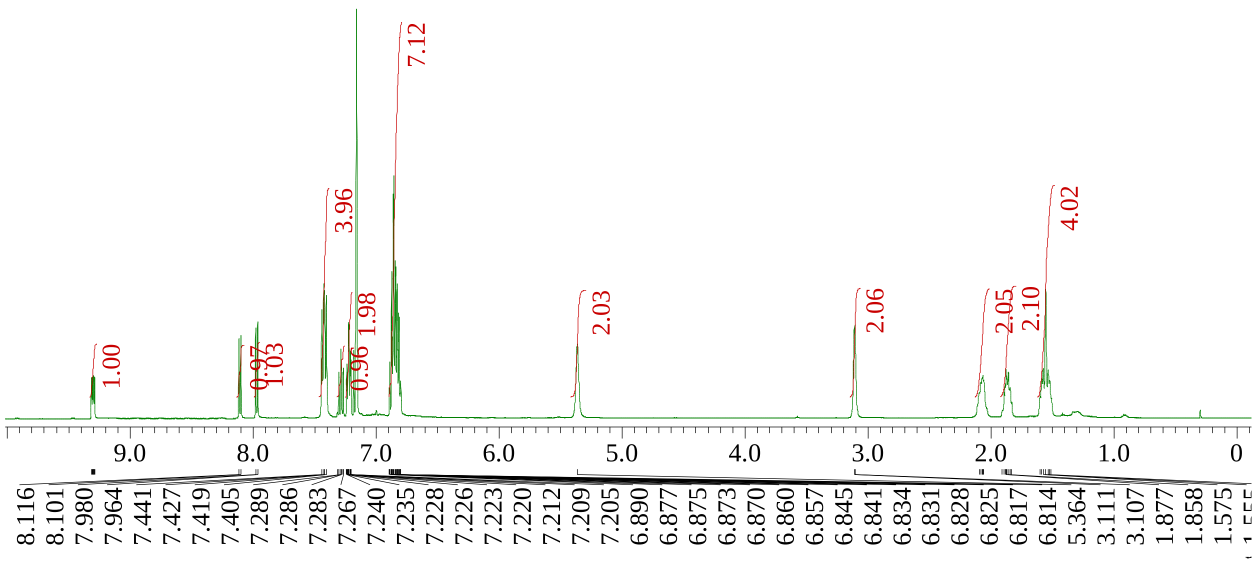

Supplementary Fig 56.
^{1}H NMR spectrum (500 MHz, C_6D_6) of complex **6**.

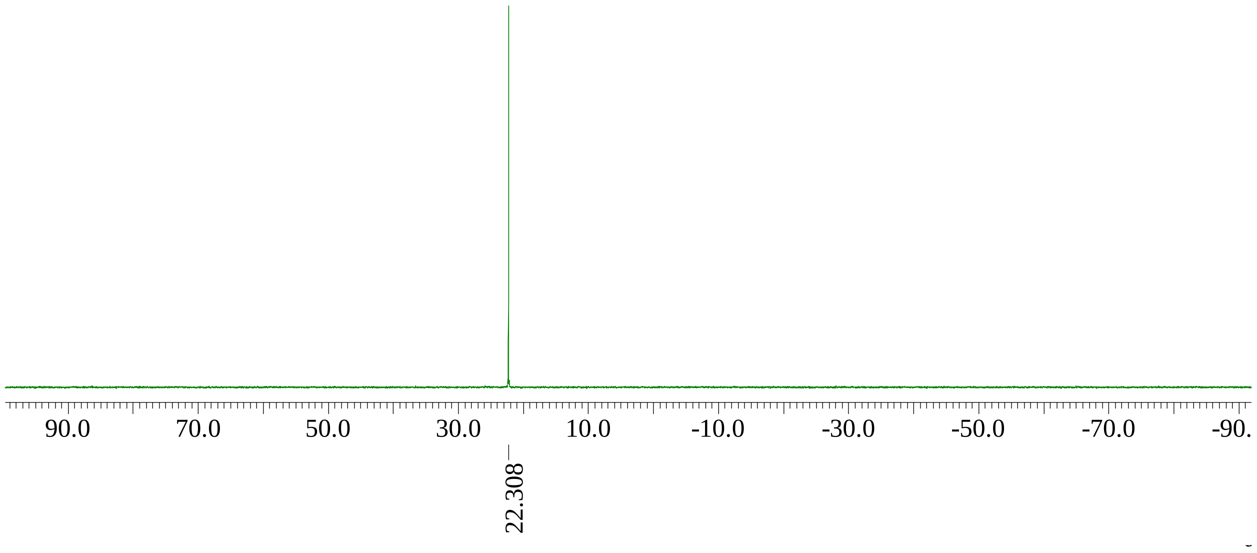

Supplementary Fig 57.
^{31}P NMR spectrum (202 MHz, C_6D_6) of complex **6**.

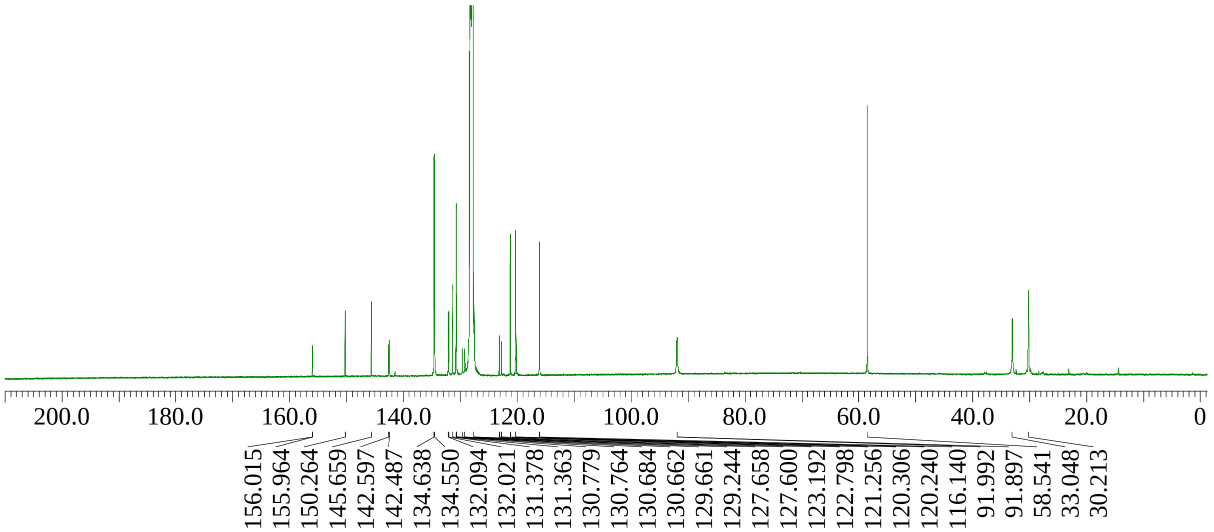

Supplementary Fig 58.
^{13}C NMR spectrum (126 MHz, C_6D_6) of complex **6**.

(1,5-Cyclooctadiene)[2-(diphenylphosphino)benzenesulfonato]iridium(I) (**7**)

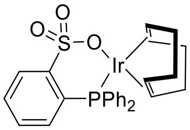

To a solution of 2-(diphenylphosphino)benzenesulfonic acid (171.2 mg, 0.50 mmol) in THF (6 mL), sodium hydride (oil free, 90% purity, 16 mg, 0.60 mmol) was added at room temperature, and the mixture was stirred at the same temperature for 17 h. To the resulting yellow solution, chlorobis(1,5-cyclooctadiene)iridium dimer (167.9 mg, 0.25 mmol) was added at room temperature. After stirring for 4 h at the same temperature to give an orange solution, the solvent was removed under reduced pressure. The residue was washed with hexane and extracted with toluene. The solvent was removed under reduced pressure to afford complex **7** as an orange solid (106.5 mg, 0.17 mmol, 33%). Crystals suitable for X-ray crystallography were obtained by recrystallization from benzene/pentane: ^{1}H NMR (500 MHz, CD_2Cl_2): δ 8.10 (dd, *J* = 7.8, 4.3 Hz, 1H), 7.62–7.56 (m, 5H), 7.50–7.45 (m, 7H), 7.33–7.29 (m, 1H), 5.16 (br, 2H), 2.69 (br, 2H), 2.36–2.19 (m, 4H), 1.92–1.83 (m, 2H), 1.73–1.67 (m, 2H); ^{31}P NMR (202 MHz, CD_2Cl_2): δ 2.75; ^{13}C NMR (126 MHz, CD_2Cl_2): δ 147.6 (d, *J* = 12.7 Hz), 135.3 (d, *J* = 1.8 Hz), 135.1 (d, *J* = 11.8 Hz), 131.5 (d, *J* = 2.6 Hz), 131.2 (d, *J* = 2.6 Hz), 131.0 (d, *J* = 6.4 Hz), 130.9 (d, *J* = 8.2 Hz), 129.5 (d, *J* = 54.6 Hz), 128.9 (d, *J* = 11.0 Hz), 128.7 (d, *J* = 7.2 Hz), 97.5 (d, *J* = 11.8 Hz), 53.6, 33.5 (d, *J* = 3.7 Hz), 28.9 (d, *J* = 1.8 Hz).

These data were consistent with those in literature[58]. NMR spectra of complex **7** are shown in Supplementary Figs 59–61.

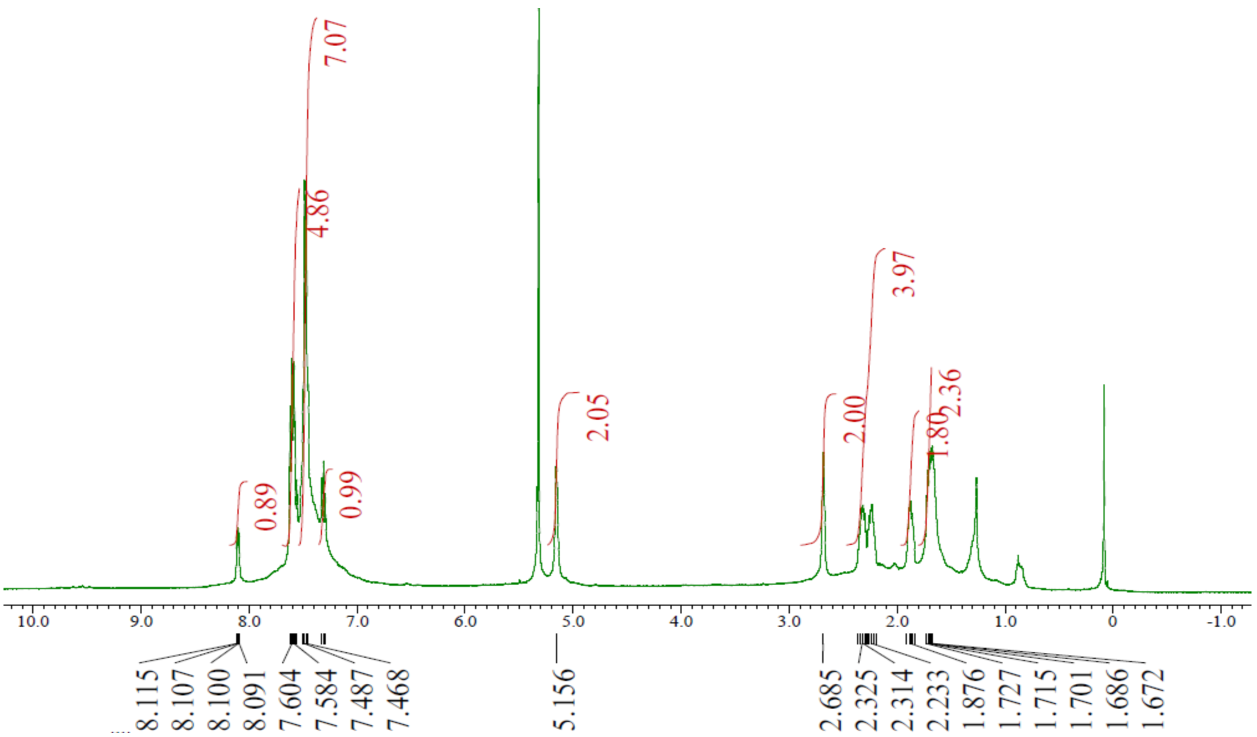

Supplementary Fig 59.
^{1}H NMR spectrum (500 MHz, CD_2Cl_2) of complex **7**.

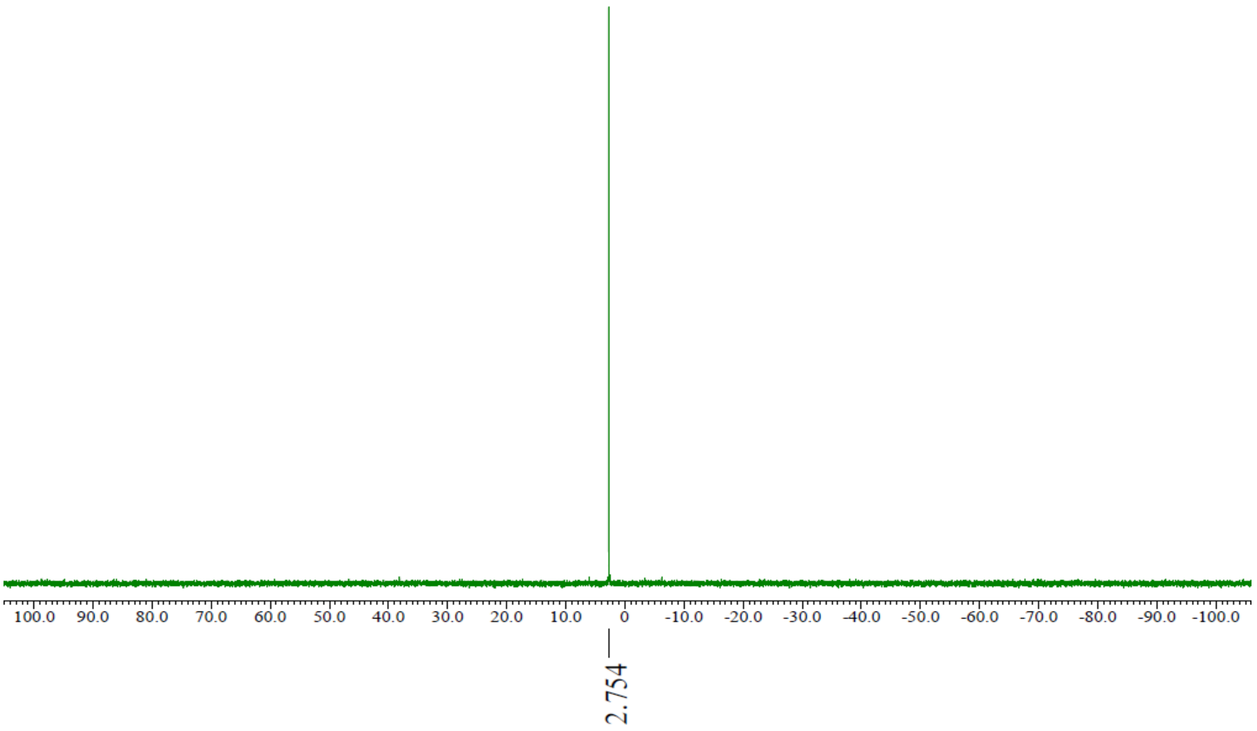

Supplementary Fig 60.
^{31}P NMR spectrum (202 MHz, CD_2Cl_2) of complex **7**.

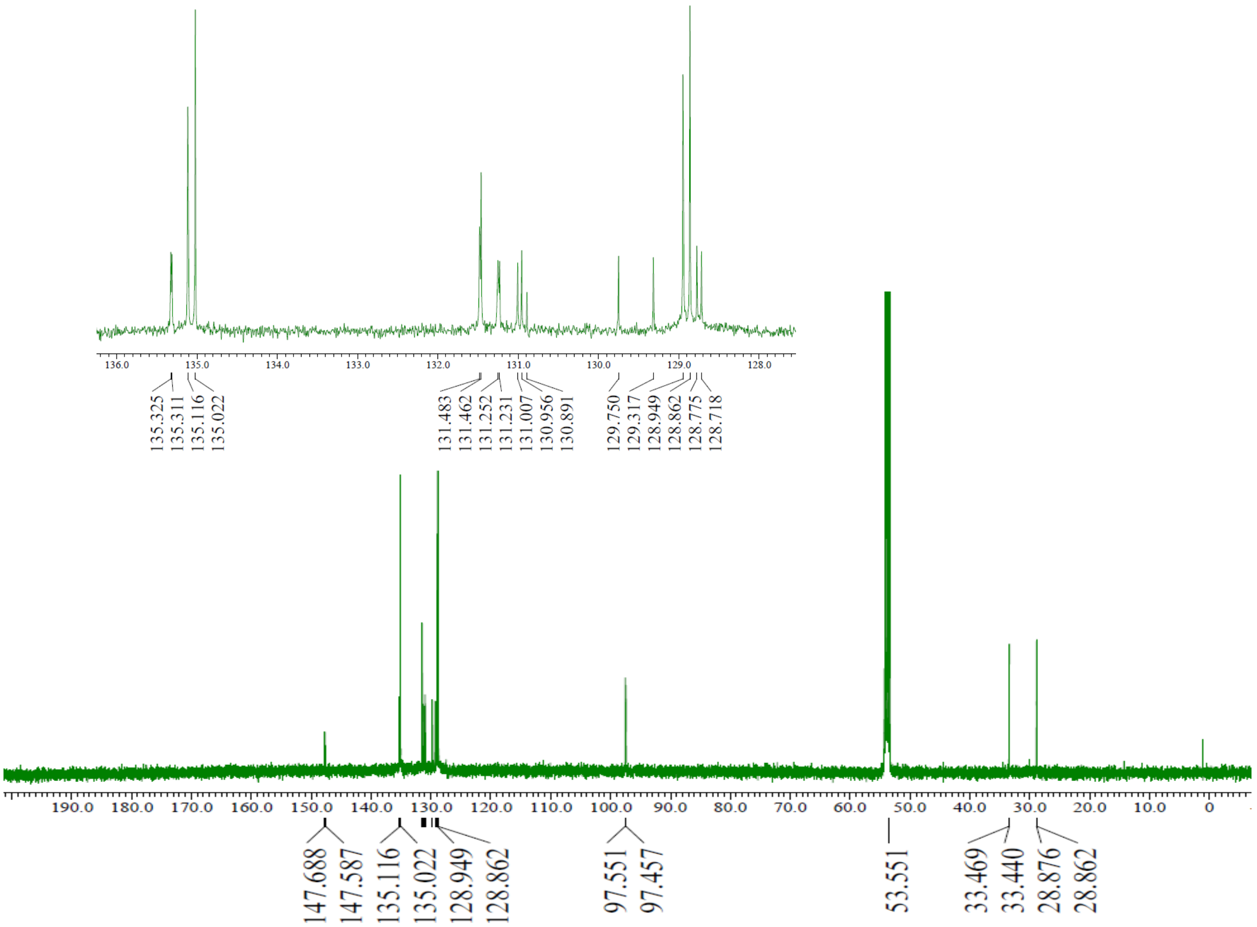

Supplementary Fig 61.

^{13}C NMR spectrum (126 MHz, CD_2Cl_2) of complex **7**.

(1,5-Cyclooctadiene)[2-(diphenylphosphino)benzoato]iridium(I) (**8**)

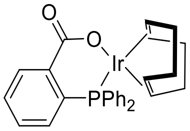

To a solution of 2-(diphenylphosphino)benzoic acid (45.9 mg, 0.15 mmol) in THF (5 mL), sodium hydride (oil free, 90% purity, 8.0 mg, 0.30 mmol) was added at room temperature, and the mixture was stirred at the same temperature for 15 h. To the resulting white suspension, chlorobis(1,5-cyclooctadiene)iridium dimer (50.4 mg, 0.075 mmol) was added at room temperature. After stirring for 4 h at the same temperature to give an orange solution, the solvent was removed under reduced pressure. The residue was washed with hexane and extracted with toluene. The solvent was removed under reduced pressure to afford complex **8** as an orange solid (88.1 mg, 0.15 mmol, 97%): ^{1}H NMR (500 MHz, C_6D_6): δ 8.71 (ddd, J = 7.6, 2.3, 0.9 Hz, 1H), 7.50–7.46 (m, 4H), 7.08–7.02 (m, 2H), 6.96–6.95 (m, 6H), 6.84 (ddt, J = 7.5, 7.5, 1.3 Hz, 1H), 5.37 (dd, J = 6.0, 3.0 Hz, 2H), 2.63 (dd, J = 6.0, 3.5 Hz, 2H), 2.12–1.99 (m, 4H), 1.59–1.53 (m, 4H); ^{31}P NMR (202 MHz, C_6D_6): δ 8.21; ^{13}C NMR (126 MHz, C_6D_6): δ 167.0 (d, J = 7.3 Hz), δ 144.5 (d, J = 14.6 Hz), 134.6, 134.5 (d, J = 11.8 Hz), 131.6 (d, J = 2.6 Hz), 130.9 (d, J = 1.9 Hz), 130.9 (d, J = 1.8 Hz), 129.7 (d, J = 53.7 Hz), 129.2, 129.2 (d, J = 7.3 Hz), 128.8 (d, J = 10.8 Hz), 99.7 (d, J = 13.7 Hz), 50.6, 33.9 (d, J = 3.7 Hz), 28.8 (d, J = 1.9 Hz); m.p.: no clear melting point was

observed upon heating until 213 ºC; Elem. Anal. calcd. for $C_{27}H_{26}IrO_2P$ C, 53.54; H, 4.33; N, 0.00; found C, 53.42; H, 4.32; N, 0.00.

NMR spectra of complex **8** are shown in Supplementary Figs 62–64.

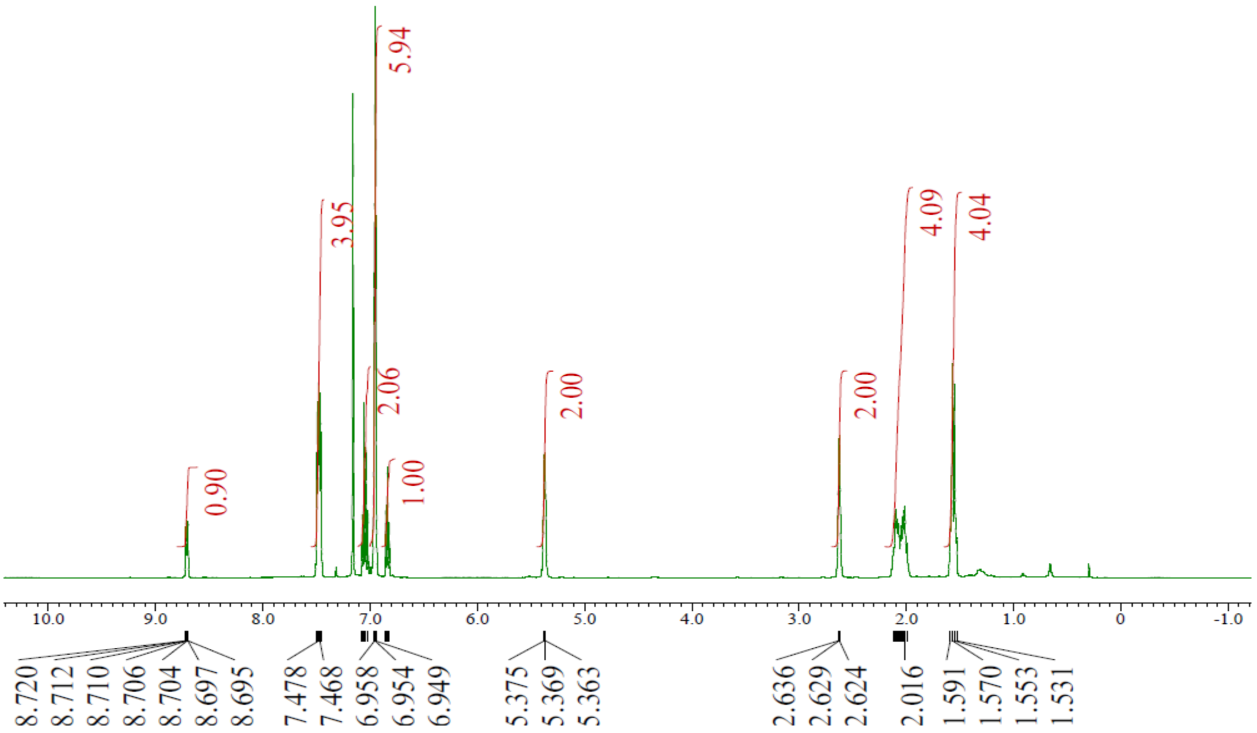

Supplementary Fig 62.

^{1}H NMR spectrum (500 MHz, C_6D_6) of complex **8**.

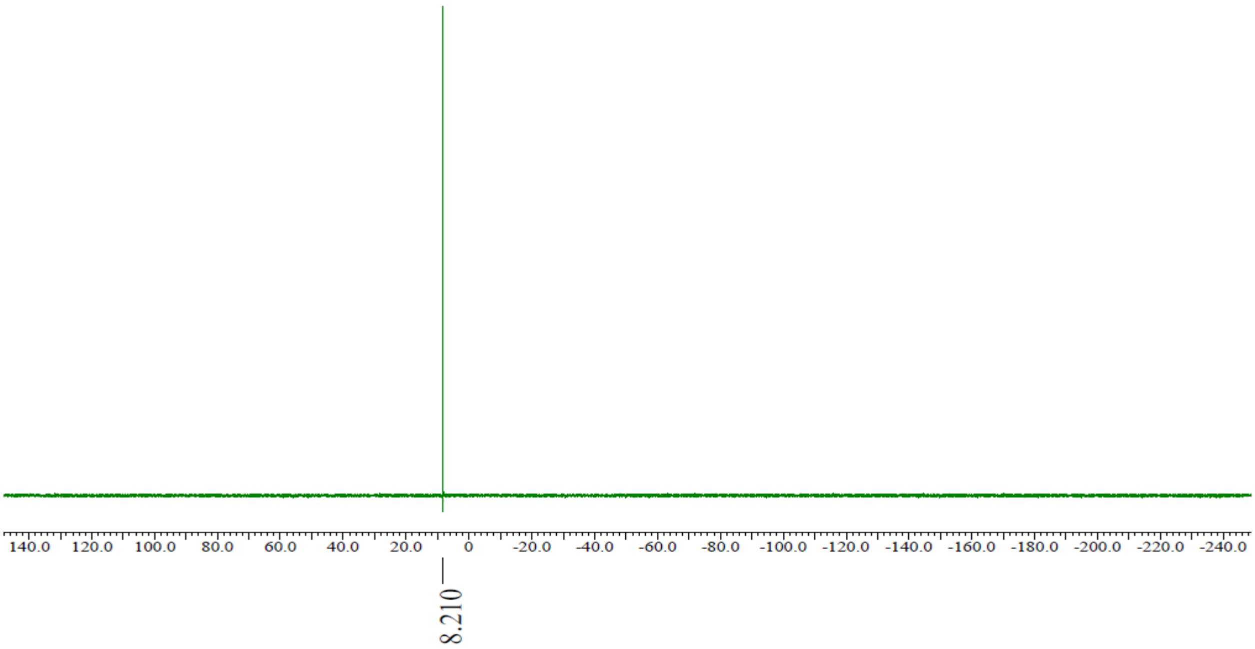

Supplementary Fig 63.
^{31}P NMR spectrum (202 MHz, C_6D_6) of complex **8**.

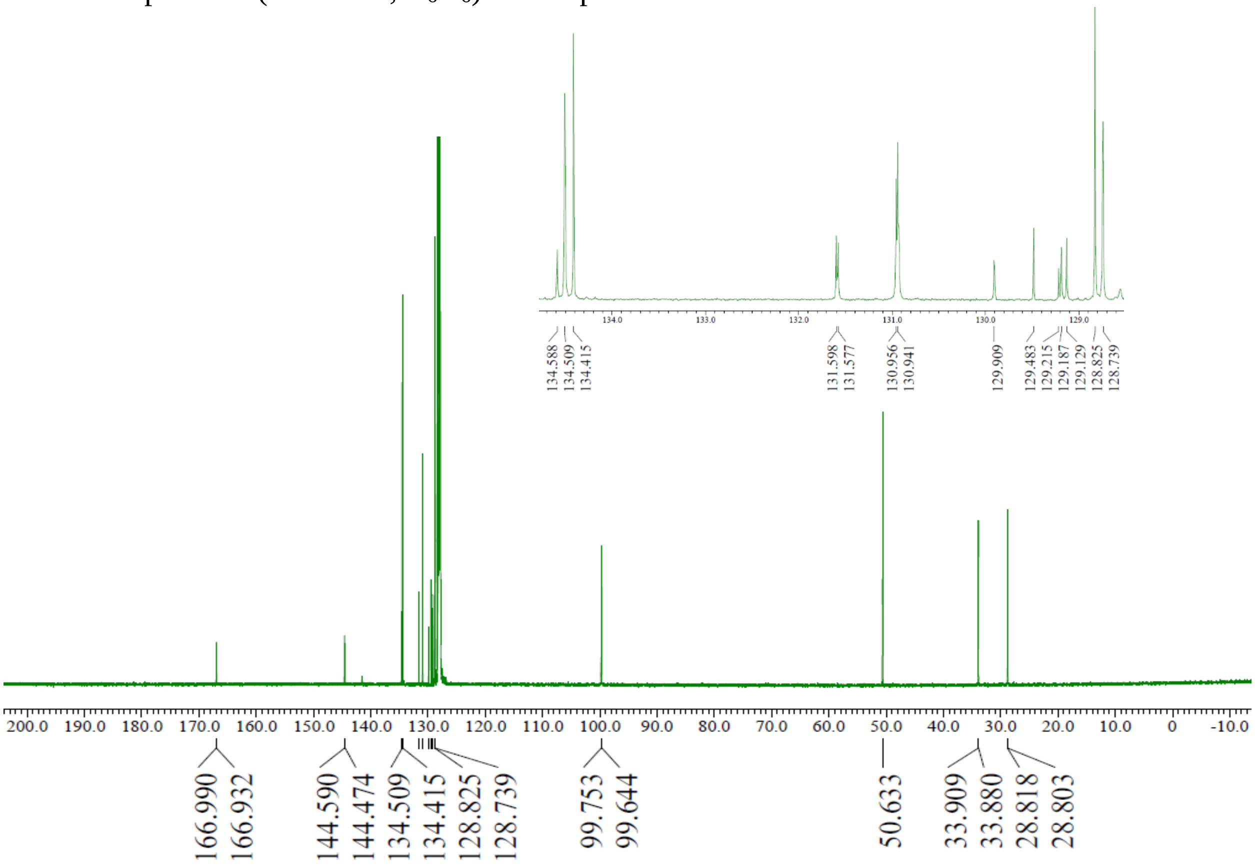

Supplementary Fig 64.
^{13}C NMR spectrum (126 MHz, C_6D_6) of complex **8**.

(1,5-Cyclooctadiene)[2-(2-diphenylphosphinophenyl)pyrrolido]rhodium(I) (**9**)

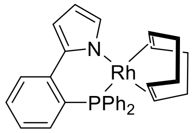

To a solution of 2-(2-diphenylphosphinophenyl)pyrrole (**5**) (49.1 mg, 0.15 mmol) in THF (3 mL), sodium hydride (oil free, 90% purity, 6.0 mg, 0.23 mmol) was added at room temperature. After stirring for 23 h at room temperature, the resulting light yellow solution was filtered through a membrane filter. To a solution of chlorobis(1,5-cyclooctadiene)rhodium dimer (37.0 mg, 0.075 mmol) in THF (3 mL), the filtrate was added at room temperature, and stirred at the same temperature for 24 h to give a brown solution. The solvent was removed under reduced pressure. The residue was washed with hexane and extracted with toluene. The solvent was removed under reduced pressure to afford complex **9** as an yellow solid (75.6 mg, 0.14 mmol, 94%). Crystals suitable for X-ray crystallography were obtained by recrystallization from benzene/pentane: ^{1}H NMR (500 MHz, C_6D_6): δ 7.91 (dd, *J* = 8.0, 4.5 Hz, 1H), 7.54–7.50 (m, 4H), 7.20 (dd, *J* = 8.5, 8.5 Hz, 1H), 7.13 (dd, *J* = 7.8, 7.8 Hz, 1H), 6.94–6.91 (m, 8H), 6.76 (dd, *J* = 7.5, 7.5 Hz, 1H), 6.65 (dd, *J* = 2.8, 2.8 Hz, 1H), 5.73 (br, 2H), 3.21 (br, 2H), 2.26–2.19 (m, 2H), 2.07–2.00 (m, 2H), 1.84–1.74 (m, 4H); ^{31}P NMR (202 MHz, C_6D_6): δ 31.2 (d, *J* = 156 Hz); ^{13}C NMR (126 MHz, C_6D_6): δ 145.6 (d, *J* = 17.3 Hz), 137.0 (d, *J* = 10.8 Hz), 134.4 (d, *J* = 11.0 Hz), 131.6, 131.5 (d, *J* = 2.8 Hz), 131.35, 131.25, 131.2 (d, *J* = 2.8 Hz), 128.4 (overlapped with solvent peaks and confirmed by DEPT analysis, d, *J* = 9.8 Hz), 128.1 (overlapped with solvent peaks and confirmed by DEPT analysis, d, *J* = 9.8 Hz), 123.0 (d, *J* = 7.2 Hz), 118.4 (d, *J* = 45.5 Hz), 111.7 (d, *J* = 109.2 Hz), 104.4 (dd, *J* = 10.9, 7.2 Hz), 72.2 (d, *J* = 11.8 Hz), 32.9 (d, *J* = 2.6 Hz), 28.7 (d, *J* = 1.9 Hz); m.p.: no clear melting point was observed upon heating until 227 °C; Elem. Anal. calcd. for $C_{30}H_{29}NPRh$ C, 67.04; H, 5.44; N, 2.61; found C, 67.16; H, 5.43; N, 2.76.

NMR spectra of complex **9** are shown in Supplementary Figs 65–67.

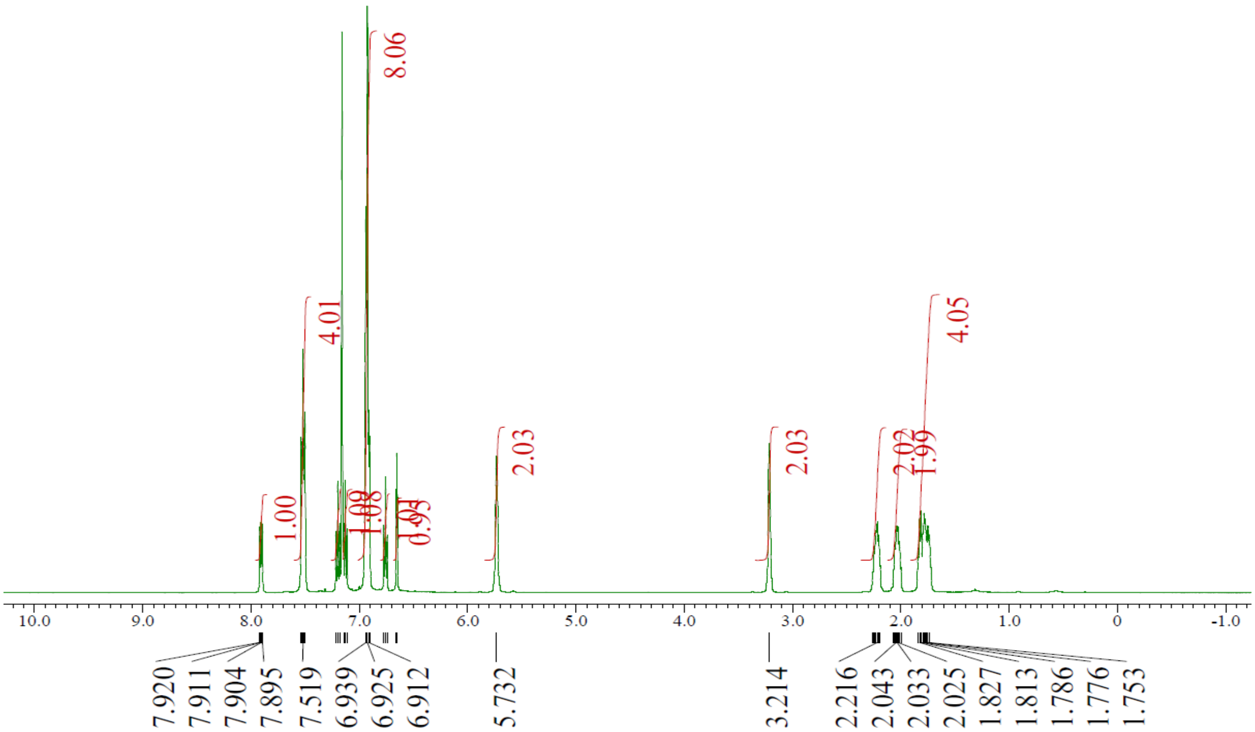

Supplementary Fig 65.

^{1}H NMR spectrum (500 MHz, C_6D_6) of complex **9**.

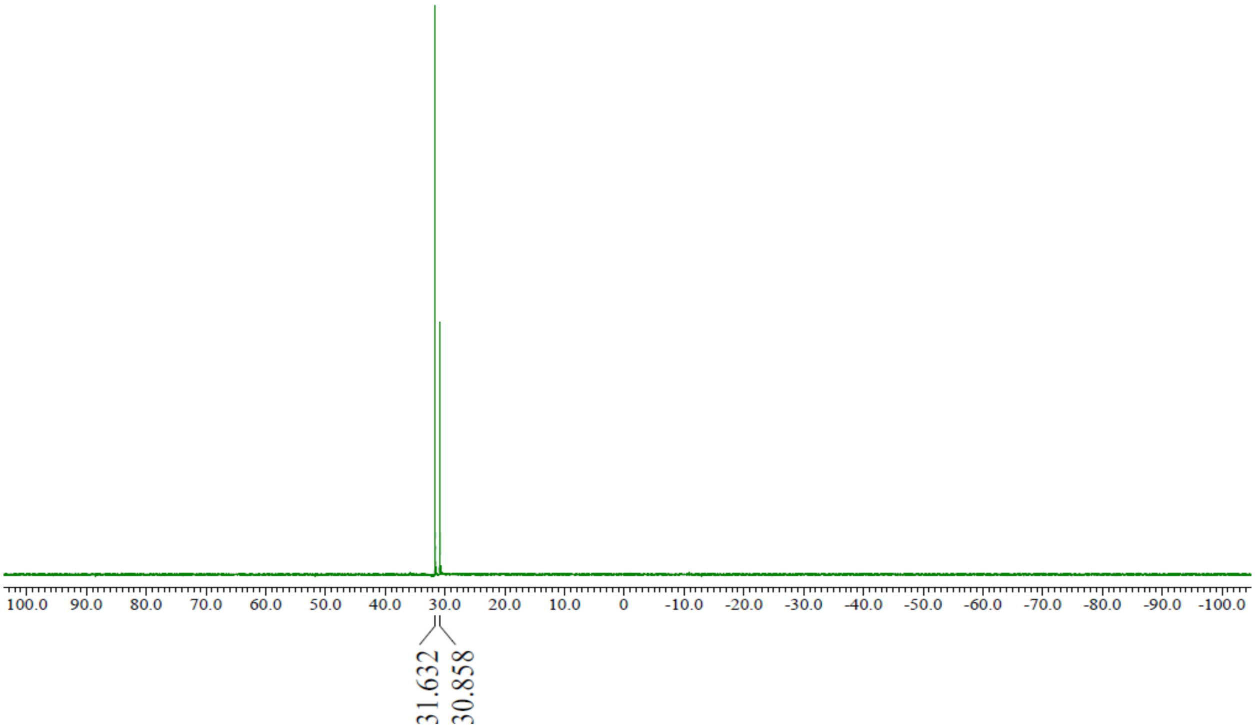

Supplementary Fig 66.
^{31}P NMR spectrum (202 MHz, C_6D_6) of complex **9**.

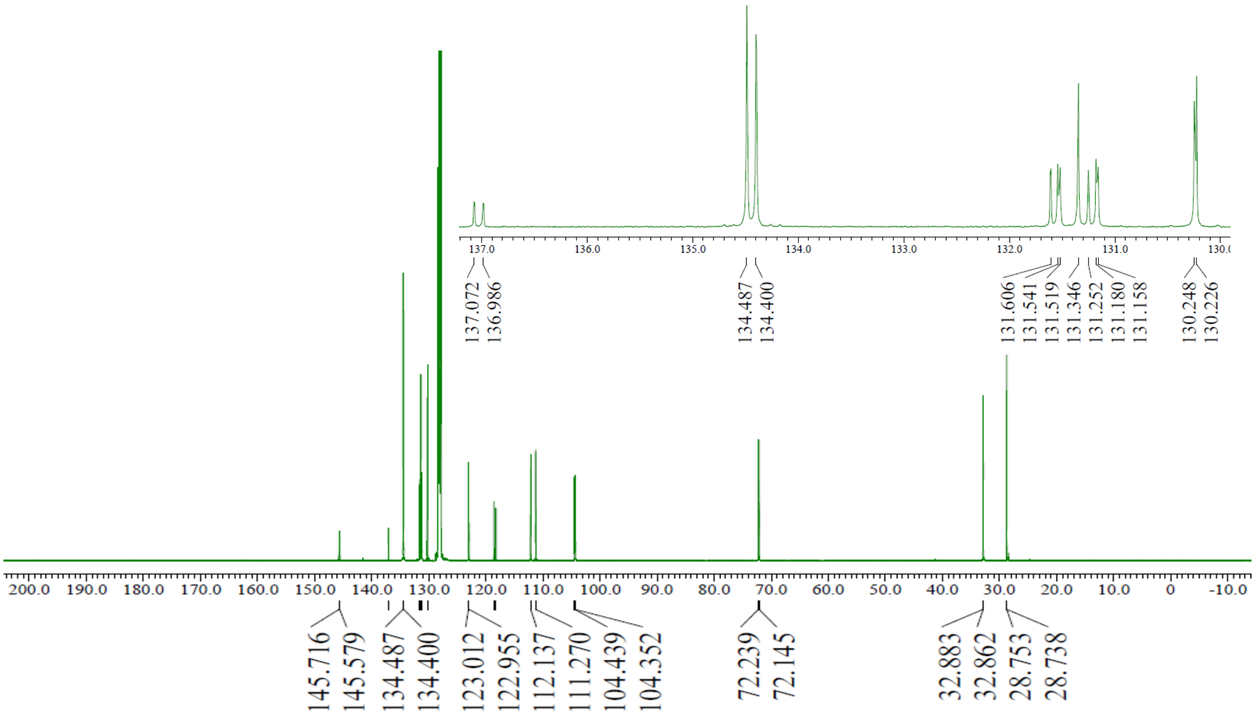

Supplementary Fig 67.
^{13}C NMR spectrum (126 MHz, C_6D_6) of complex **9**.

The reaction of ligand **5** with $[Ir(cod)_2]BAr^F_4$ (Supplementary Fig 1)

To a solution of 2-(2-diphenylphosphinophenyl)pyrrole (**5**) (49 mg, 0.15 mmol) in THF (3 mL), $[Ir(cod)_2]BAr^F_4$ (191 mg, 0.15 mmol) was added at room temperature. After stirring for 16 h at room temperature, the resulting purple solution was concentrated under reduced pressure. The residue was washed with hexane to afford the tautomeric mixture of complex **23** and **24** as a purple solid (220 mg). ^{31}P NMR analyses showed that the major product was complex **24** in THF-d_8 and complex **23** in C_6D_6: ^{1}H NMR (500 MHz, THF-d_8): δ 11.8 (br, 0.8H (**24**)), 8.11–7.46 (m, 33H), 7.39 (s, 0.8H (**24**)), 7.24 (d, J = 5.5 Hz, 0.2H (**23**)), 7.04 (d, J = 2.0 Hz, 0.8H (**24**)), 6.25 (s, 0.8H (**24**)), 5.38 (br, 0.4H (**23**)), 5.21 (br, 1.6H (**24**)), 4.96 (s, 0.4H (**23**)), 3.45 (br, 0.4H (**23**)), 3.41 (br, 1.6H (**24**)), 2.33–2.08 (m, 8H), 1.63–1.31 (m, 8H); ^{31}P NMR (202 MHz, THF-d_8**)**: δ 26.1 (**24**), 18.0 (**23**) (**23**:**24** = 0.20:0.80); ^{31}P NMR (202 MHz, C_6D_6**)**: δ 26.0 (**24**), 17.4 (**23**) (**23**:**24** = 0.89:0.11); ^{13}C NMR (126 MHz, THF-d_8): δ 162.9 (q, J = 49.7 Hz), 156.2, 141.7 (d, J = 7.3 Hz), 141.1 (d, J = 19.0 Hz), 137.0 (d, J = 14.5 Hz), 136.6, 135.7, 135.3 (d, J = 11.0 Hz), 134.5 (d, J = 8.2 Hz), 133.9, 133.8 (d, J = 1.8 Hz), 133.7 (d, J = 5.5 Hz), 133.6 (d, J = 20.0 Hz), 133.0 (d, J = 2.6 Hz), 132.9 (d, J = 2.8 Hz), 131.4 (d, J = 6.3 Hz), 131.0, 130.5–129.7 (m), 129.4, 128.8, 127.4 (d, J = 55.6 Hz), 126.7, 126.2 (d, J = 47.3 Hz), 125.7, 124.5, 122.3, 118.3–118.2 (m), 95.9 (d, J = 12.7 Hz), 85.8, 72.6, 71.4, 66.0, 33.2 (d, J = 2.6 Hz), 31.2, 30.6 (d, J = 1.9 Hz), 27.7.

NMR spectra of the tautomeric mixture of complex **23** and **24** are shown in Supplementary Figs 68–73.

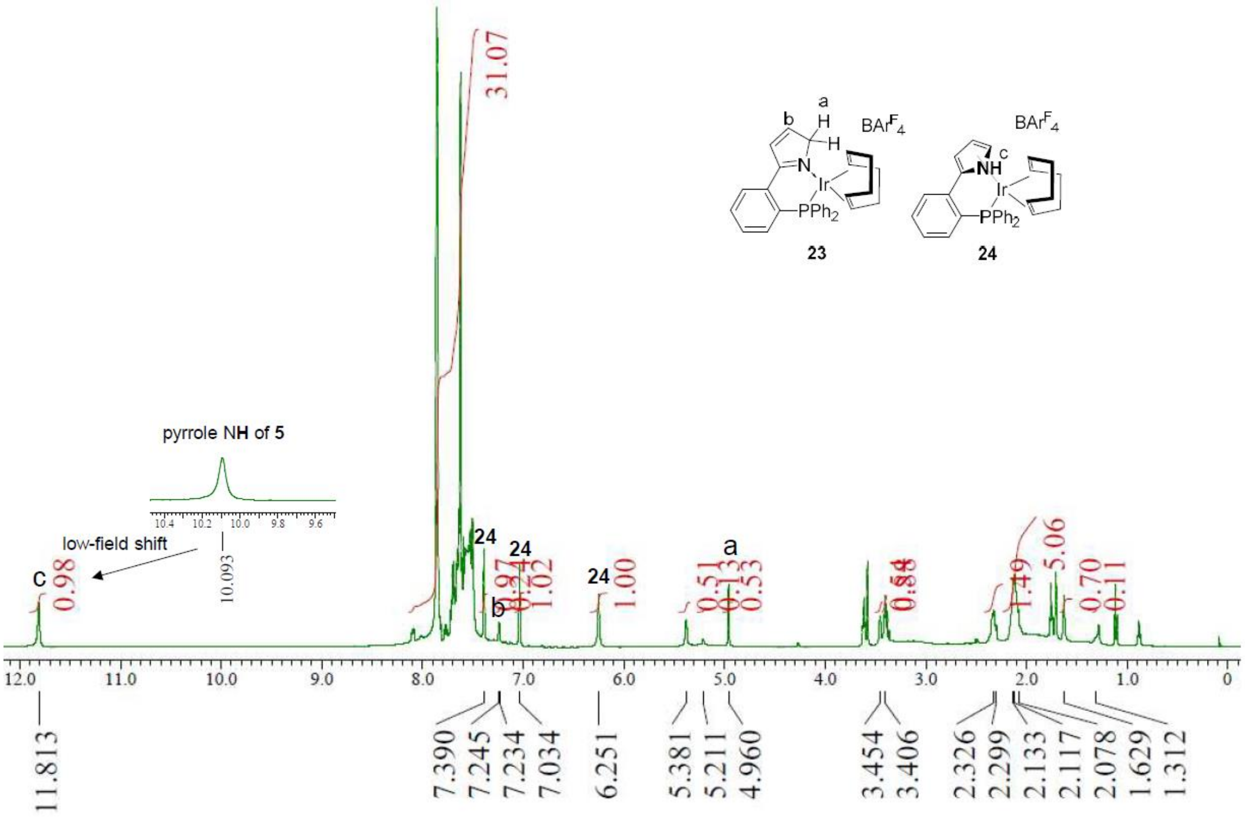

Supplementary Fig 68.

^{1}H NMR spectrum (500 MHz, THF-d_8, 25 °C) of mixture of **23** and **24**.

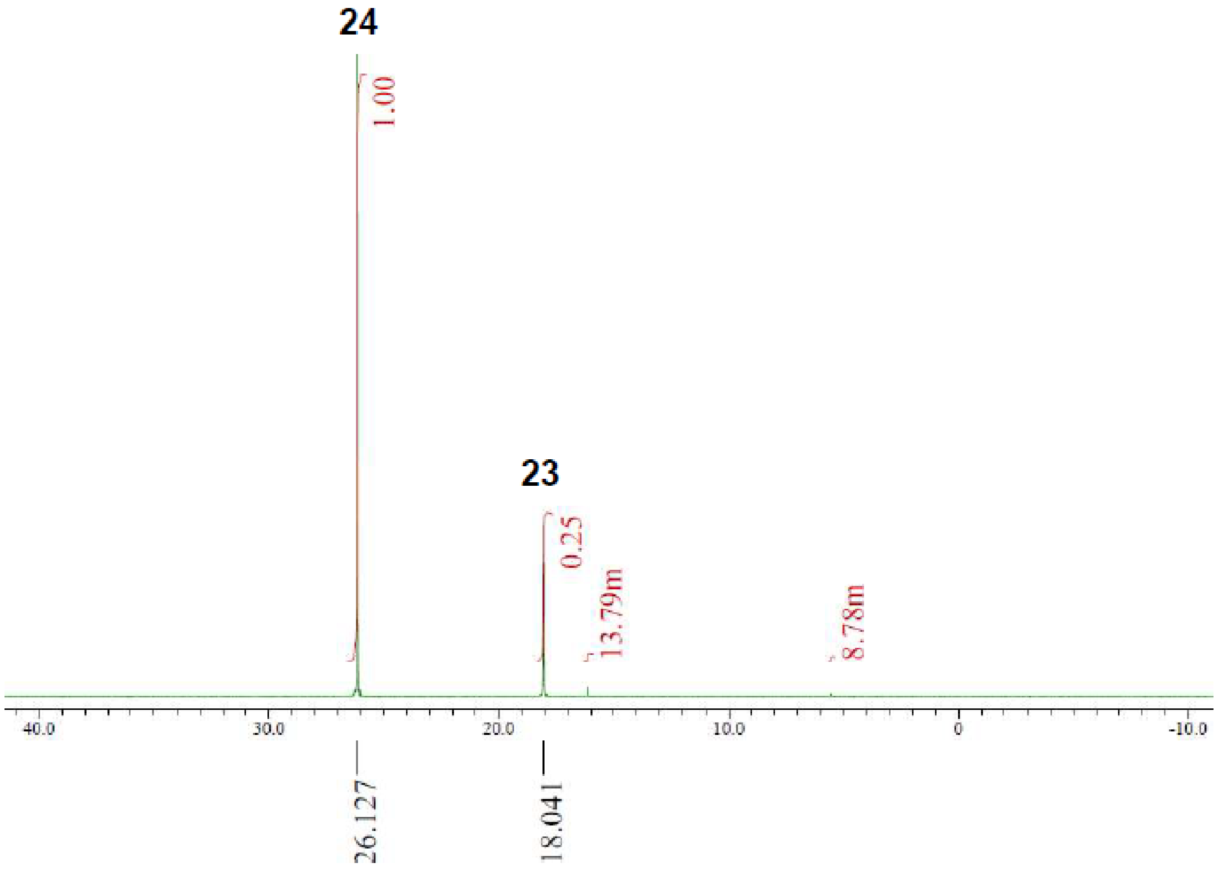

Supplementary Fig 69.
^{31}P NMR spectrum (202 MHz, THF-d_8, 25 °C) of mixture of **23** and **24**.

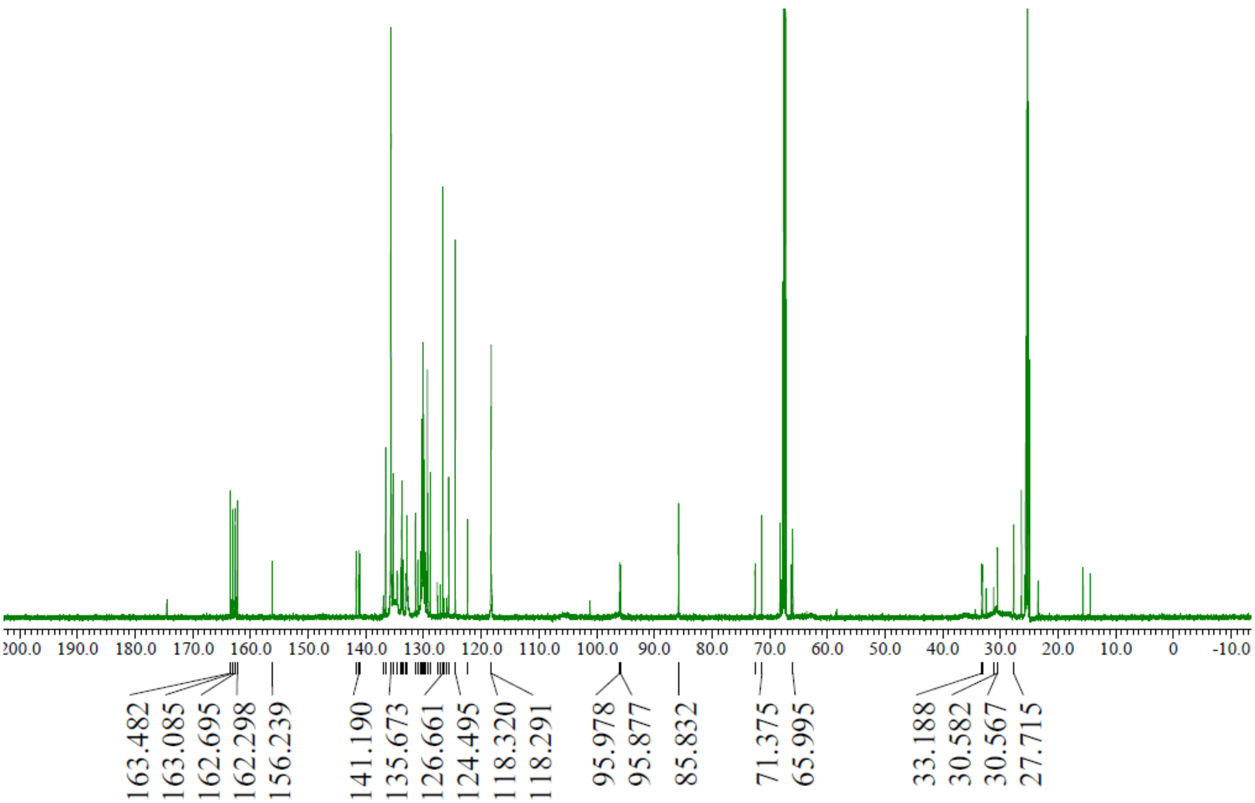

Supplementary Fig 70.
^{13}C NMR spectrum (126 MHz, THF-d_8, 25 °C) of mixture of **23** and **24**.

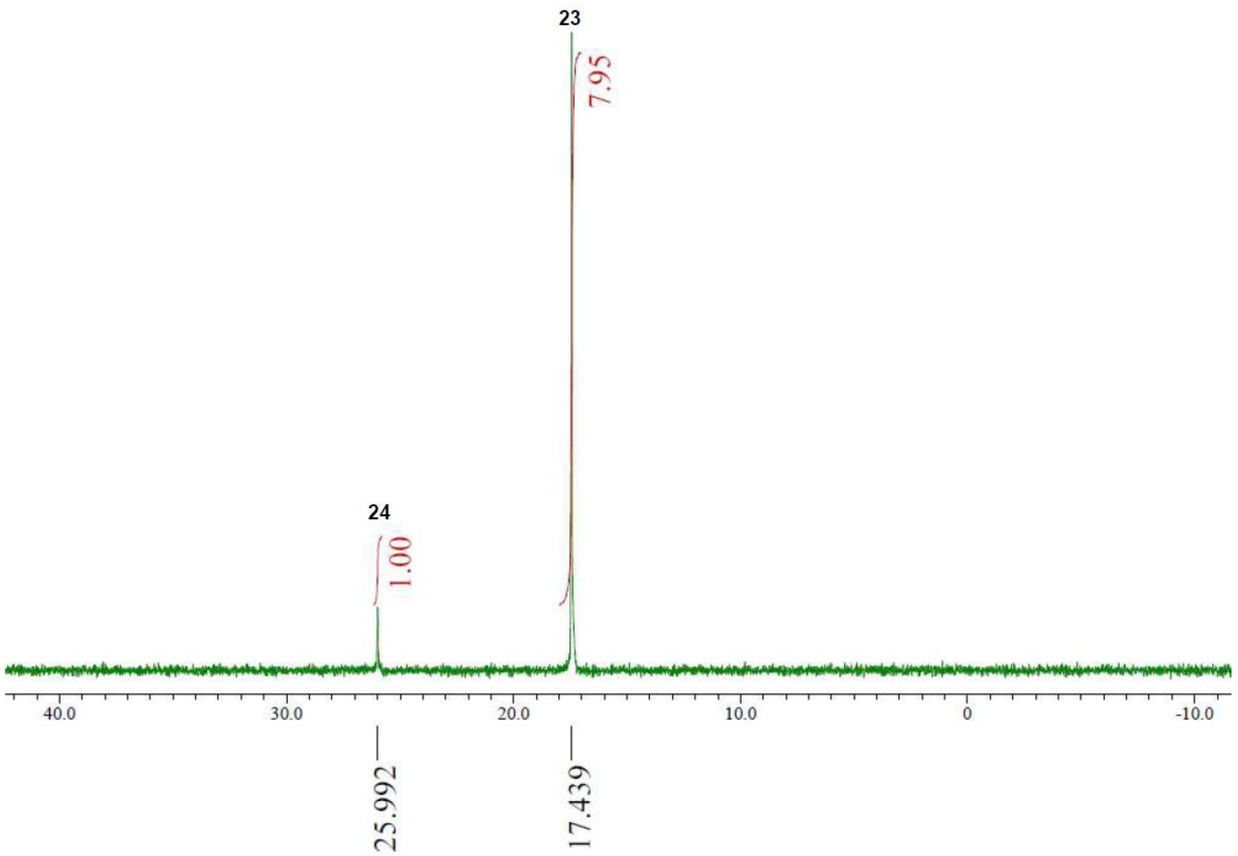

Supplementary Fig 71.
^{31}P NMR spectrum (202 MHz, C_6D_6, 25 °C) of mixture of **23** and **24**.

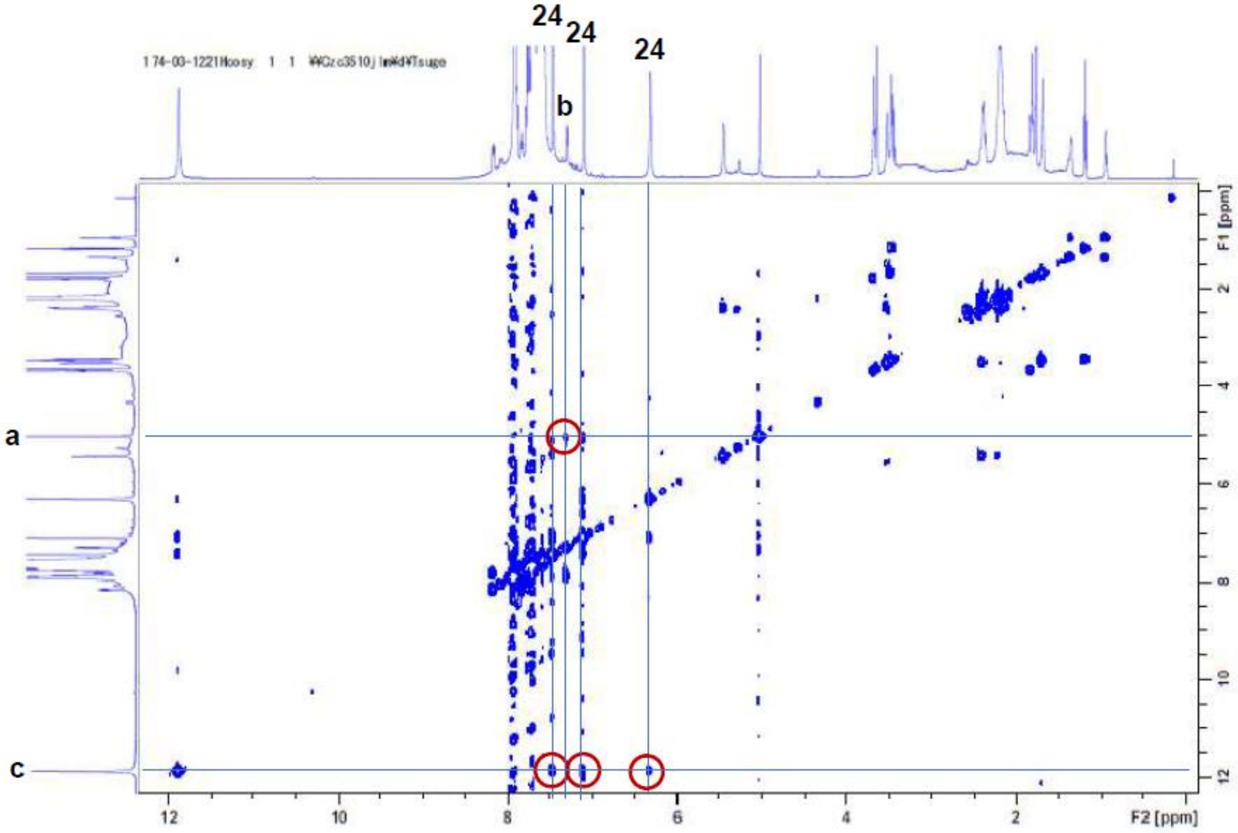

Supplementary Fig 72.
COSY spectrum (THF-d_8, 25 °C) of mixture of **23** and **24**.

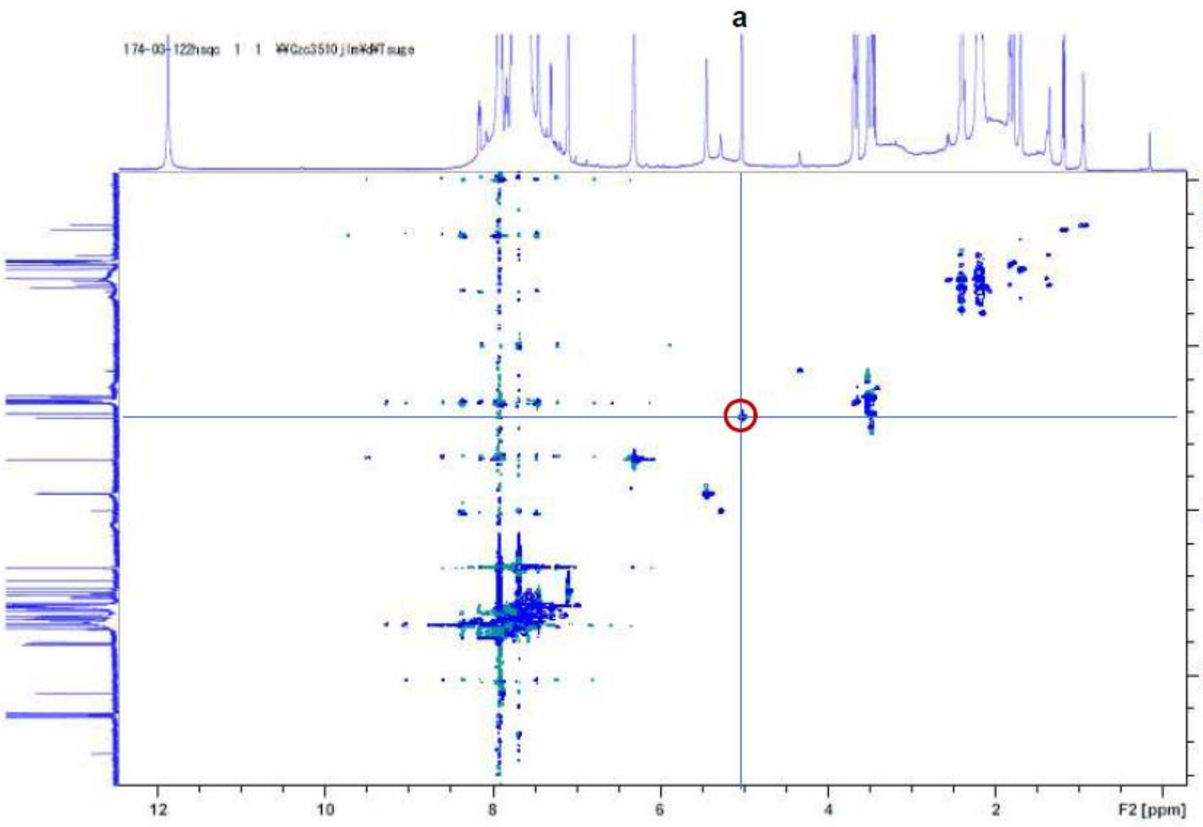

Supplementary Fig 73.
HSQC spectrum (THF-d_8, 25 °C) of mixture of **23** and **24**.

7. General Procedures

7-1. Catalyst screening (Fig. 2)

A 50 mL stainless steel autoclave, a glass tube, and a stirring bar were dried in an oven at 150 °C, and then cooled inside a glovebox under argon atmosphere. Complex (5 μmol), 1,3-diphenylurea (**1a**) (35.4 mg, 0.17 mmol), and toluene (2 mL) were added into the glass tube, and the tube was capped with a funnel to prevent evaporation of the solvent. After the glass tube was set in the autoclave and the autoclave was sealed, the autoclave was brought out from the glovebox. The autoclave was degassed three times using H_2 and was pressurized with 2 MPa of H_2 for 5 min with stirring. The reaction mixture was stirred in an isothermal heating block at 130 °C for 18 h. After cooling to room temperature, H_2 was vented off carefully. To the reaction mixture, DMSO-d_6 (ca. 2 mL) was added to homogenize the reaction mixture, and dibromomethane (57.9 mg, 0.33 mmol) was added as an internal standard. The solution (toluene/DMSO-d_6 = 1/1, 0.4 mL) was transferred into an NMR tube and diluted with DMSO-d_6 (0.2 mL) for NMR analysis. The conversion of **1a** and the yields of formanilide (**2a**) and aniline (**3a**) were determined by ^{1}H NMR spectroscopy with the internal standard.

A representative example using complex **4** is shown in Supplementary Fig 74.

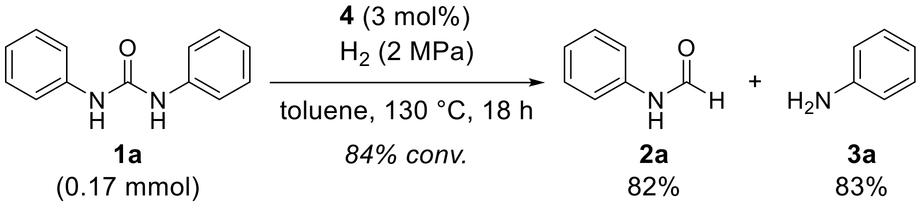

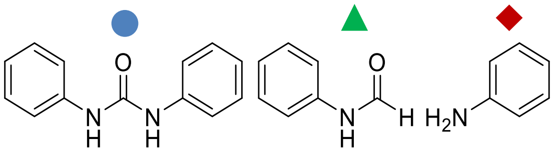

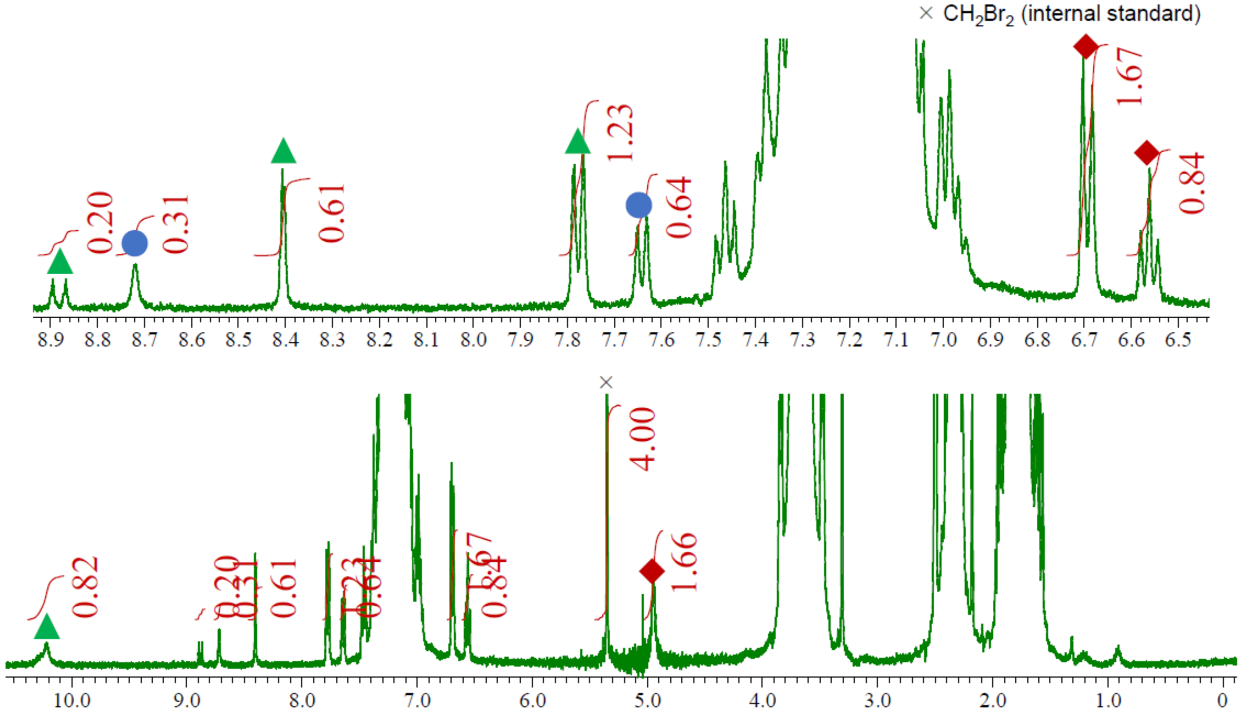

Supplementary Fig 74.
^{1}H NMR spectrum (400 MHz, DMSO-d_6/toluene/THF) of the hydrogenolysis of 1,3-diphenylurea (**1a**) using complex **4**.

<u>7-2. Condition A: the hydrogenolysis of urea derivatives using Ir catalyst **4**</u>

A 50 mL stainless steel autoclave, a glass tube, and a stirring bar were dried in an oven at 150 °C, and then cooled inside a glovebox under argon atmosphere. Catalyst **4** (9.4 mg, 15 µmol), urea (0.50 mmol), and THF (3 mL) were added into the glass tube, and the tube was capped with a funnel to prevent evaporation of the solvent. After the glass tube was set in the autoclave, the autoclave was sealed. The autoclave was brought out from the glovebox. The autoclave was degassed three times using H_2 and was pressurized with 1 MPa of H_2 for 5 min with stirring. The reaction mixture was stirred in an isothermal heating block at 130 °C for 48 h. After cooling to room temperature, H_2 was vented off carefully. To the reaction mixture, DMSO-d_6 (ca. 2 mL) was added to homogenize the reaction mixture, and heptane (25.1 mg, 0.25 mmol) or dibromomethane (86.9 mg, 0.50 mmol) was added as an internal standard. The solution (THF/DMSO-d_6 = 3/2, 0.3 mL) was transferred into an NMR tube and diluted with DMSO-d_6

(0.3 mL) for NMR analysis. The conversion of urea and the yields of formamide and amine were determined by ^{1}H NMR spectroscopy with the internal standard (**1d**: heptane, others: dibromomethane). To the combined reaction mixture, ethyl acetate (30 mL) was added, and the solution was extracted with 1 M HCl aq. (20 mL × 3). The combined aqueous layers were neutralized with $NaHCO_3$ and extracted with CH_2Cl_2 (50 mL × 3). The combined CH_2Cl_2 layers were washed with brine (100 mL), dried over anhydrous Na_2SO_4, and filtered. After adding 1M HCl in MeOH, the solution was stirred at room temperature for 3 h and concentrated under reduced pressure to obtain the corresponding anilinium chloride salt. The ethyl acetate layer was washed with brine, dried over anhydrous Na_2SO_4, filtered, and concentrated under reduced pressure. The crude residue was purified by silica gel column chromatography with CH_2Cl_2/ethyl acetate as an eluent to obtain formamide.

7-3. Condition B: the hydrogenolysis of urea derivatives using Ir catalyst **4** in the presence of KO^{t}Bu

A 50 mL stainless steel autoclave, a glass tube, and a stirring bar were dried in an oven at 150 °C, and then cooled inside a glovebox under argon atmosphere. Catalyst **4** (9.4 mg, 15 µmol), urea (0.50 mmol), KO^{t}Bu (5.6 mg, 0.05 mmol), and toluene (3 mL) were added into the glass tube, and the tube was capped with a funnel to prevent evaporation of the solvent. After the glass tube was set in the autoclave, the autoclave was sealed. The autoclave was brought out from the glovebox. The autoclave was degassed three times using H_2 and was pressurized with 1 MPa of H_2 for 5 min with stirring. The reaction mixture was stirred in an isothermal heating block at 130 °C for 48 h. After cooling to room temperature, H_2 was vented off carefully. To the reaction mixture, DMSO-d_6 (ca. 2 mL) and 0.5 M HCl aq. (0.1 mL) were added to homogenize and neutralize the reaction mixture, and dibromomethane (86.9 mg, 0.50 mmol) was added as an internal standard. The solution (toluene/DMSO-d_6 = 3/2, 0.3 mL) was transferred into an NMR tube and diluted with DMSO-d_6 (0.3 mL) for NMR analysis. The conversion of urea and the yields of formamide and amine were determined by ^{1}H NMR spectroscopy with the internal standard. To the combined reaction mixture, ethyl acetate (30 mL) was added, and the solution was extracted with 1 M HCl aq. (20 mL × 3). The combined aqueous layers were neutralized with $NaHCO_3$ and extracted with CH_2Cl_2 (50 mL× 3). The combined CH_2Cl_2 layers were washed with brine (100 mL), dried over anhydrous Na_2SO_4, and filtered. After adding 1M HCl in MeOH, the solution was stirred at room temperature for 3 h and concentrated under reduced pressure to obtain the corresponding anilinium chloride salt. The ethyl acetate layer was washed with brine, dried over anhydrous Na_2SO_4, filtered, and concentrated under reduced pressure. The crude residue was purified by silica gel column chromatography with CH_2Cl_2/ethyl acetate as an eluent to obtain formamide.

7-4. Competitive reactions of urea **1a** with ester **13**, carbamate **14**, ketone **25**, or formamide **2b** (Fig. 3b and Supplementary Figs 5 and 12)

A 50 mL stainless steel autoclave, a glass tube, and a stirring bar were dried in an oven at 150 °C, and then cooled inside a glovebox under argon atmosphere. Catalyst **4** (9.4 mg, 15 µmol), 1,3-diphenylurea (**1a**) (106.1 mg, 0.50 mmol), ethyl benzoate (**13**) (75.1 mg, 0.50 mmol), octyl phenylcarbamate (**14**) (124.7 mg, 0.50 mmol), *tert*-butyl phenyl ketone (**25**) (81.1 mg, 0.50 mmol), or *N*-(4-fluorophenyl)formamide (**2b**) (69.6 mg, 0.50 mmol) and THF (3 mL) were added into the glass tube, and the tube was capped with a funnel to prevent evaporation of the solvent. After the glass tube was set in the autoclave, the autoclave was sealed. The autoclave was brought out from the glovebox. The autoclave was degassed three times using H_2 and was pressurized with 1 MPa of H_2 for 5 min with stirring. The reaction mixture was stirred in an isothermal heating block at 130 °C for 48 h. After cooling to room temperature, H_2 was vented off carefully. To the reaction mixture, DMSO-d_6 (ca. 2 mL) was added to homogenize the reaction mixture, and dibromomethane (86.9 mg, 0.50 mmol) was added as an internal standard. The solution (THF/DMSO-d_6 = 3/2, 0.3 mL) was transferred into an NMR tube and diluted with DMSO-d_6 (0.3 mL) for NMR analysis. The conversions and yields were determined by ^{1}H

NMR spectroscopy with the internal standard. ^{1}H NMR spectra of the competitive reactions are shown in Supplementary Figs 75–78.

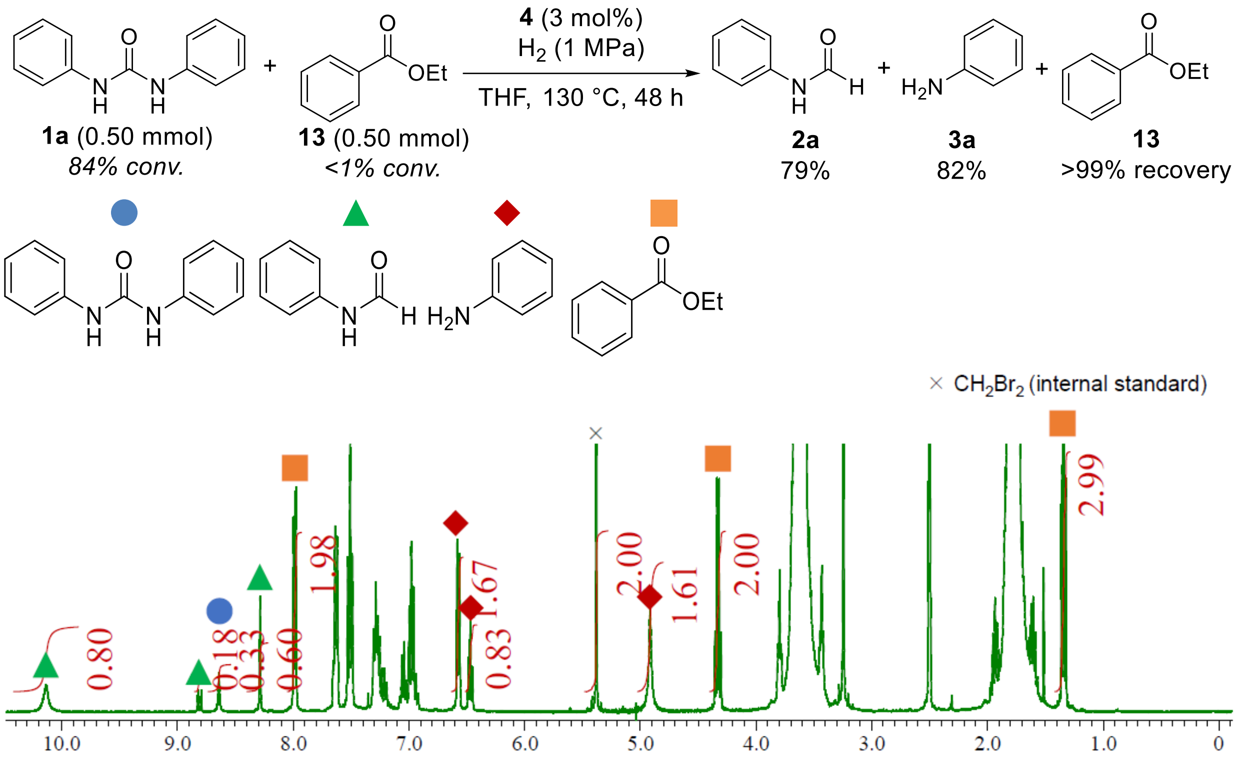

Supplementary Fig 75.

^{1}H NMR spectrum (400 MHz, DMSO-d_6/THF) of the competitive reaction with ester **13**.

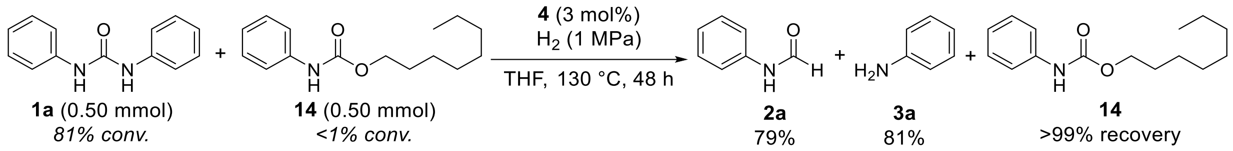

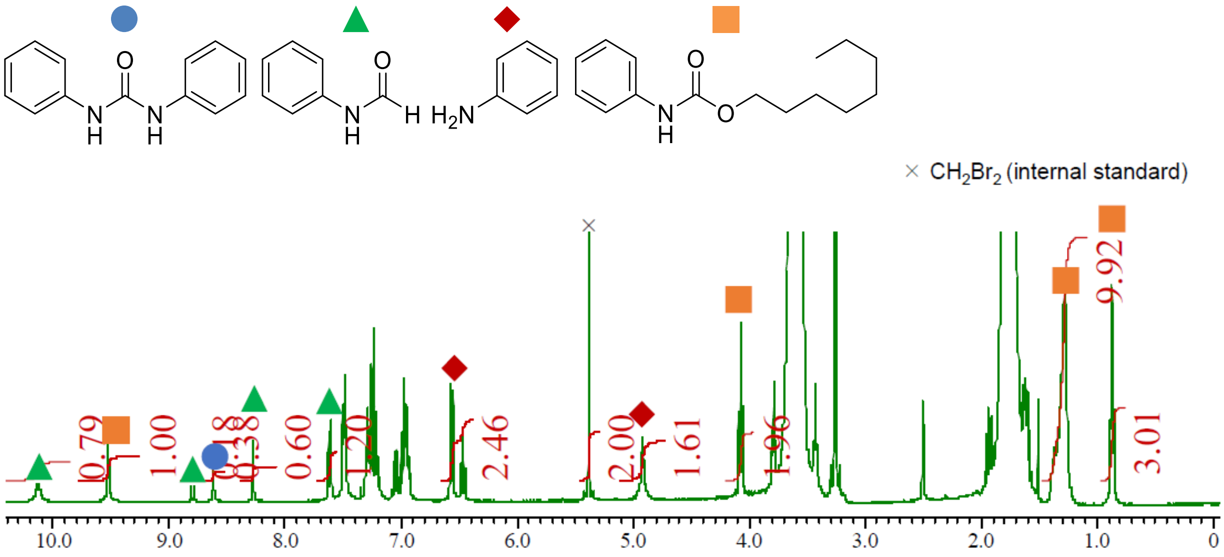

Supplementary Fig 76.
^{1}H NMR spectrum (400 MHz, DMSO-d_6/THF) of the competitive reaction with carbamate **14**.

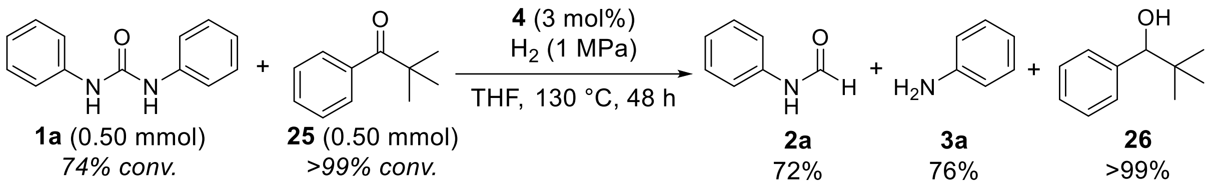

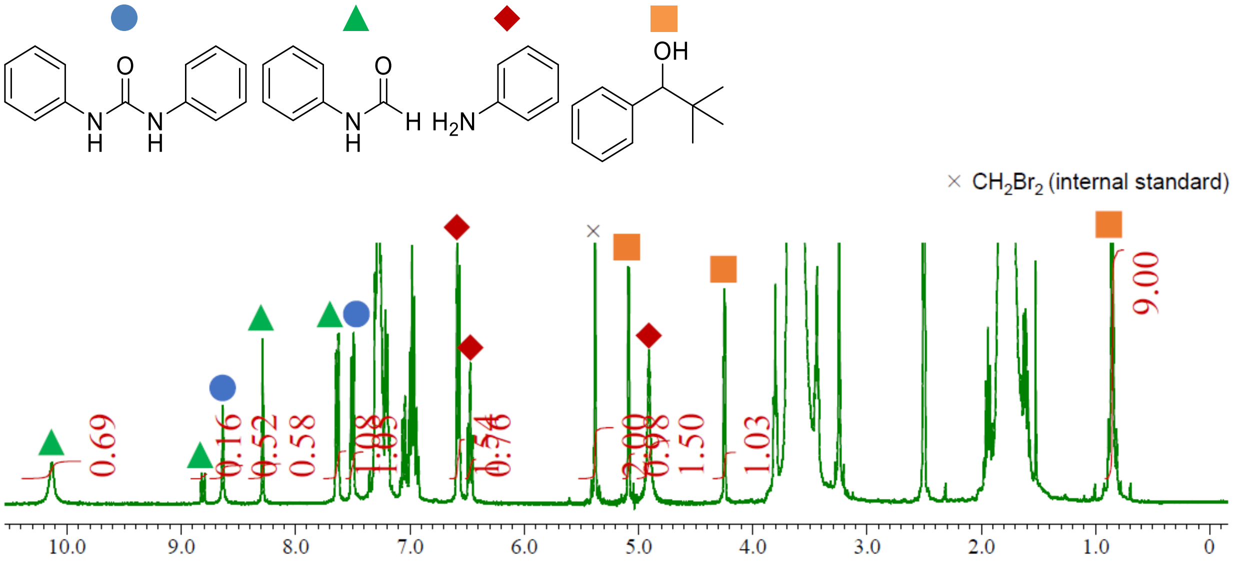

Supplementary Fig 77.

^{1}H NMR spectrum (400 MHz, DMSO-d_6/THF) of the competitive reaction with ketone **25**.

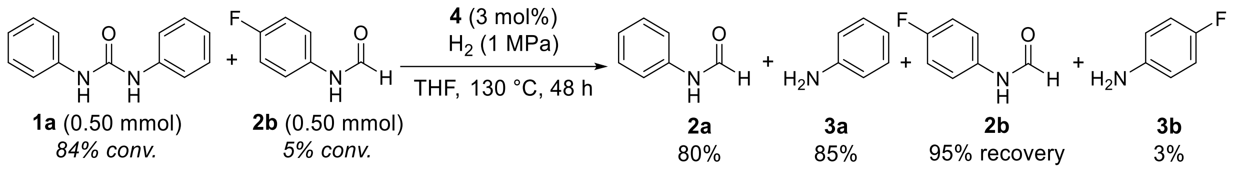

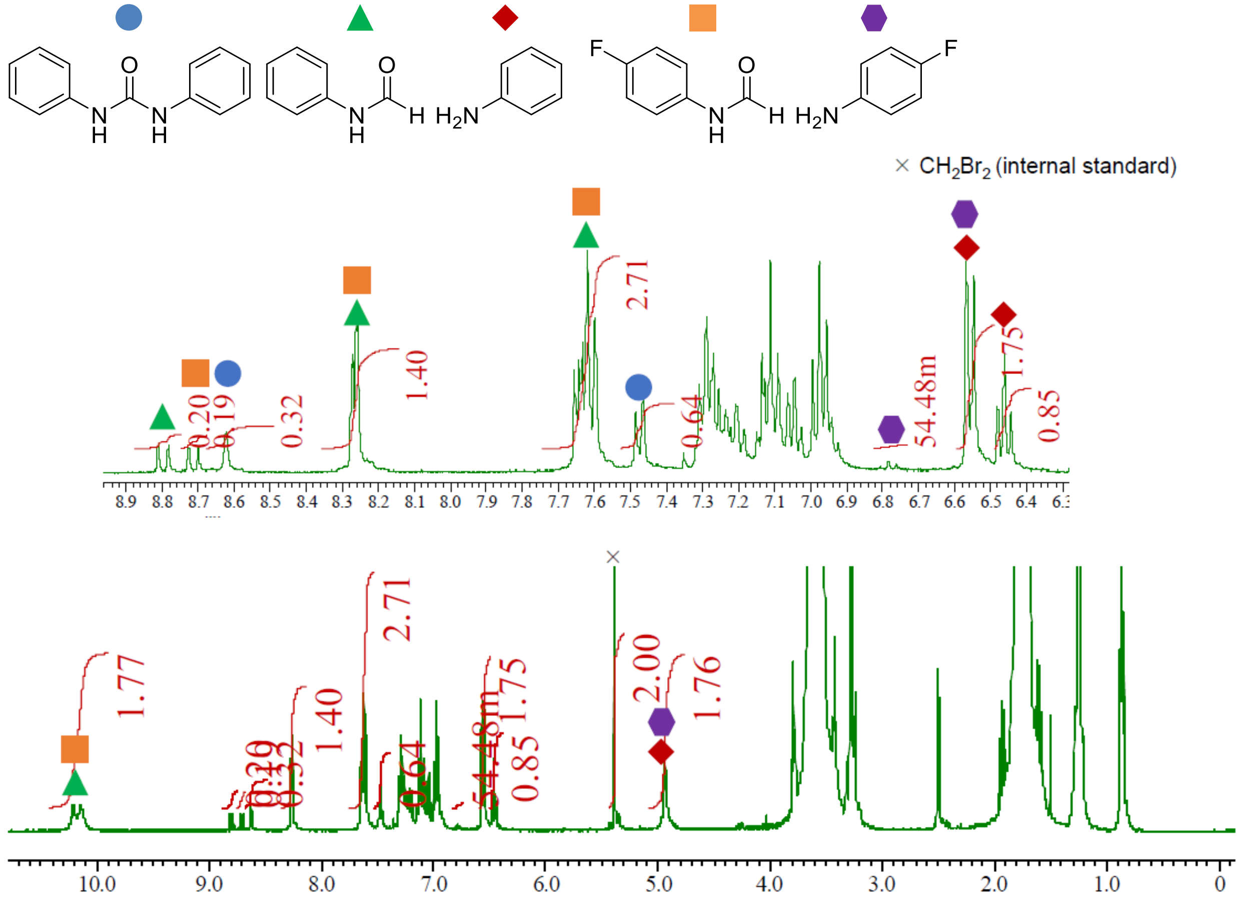

Supplementary Fig 78.

^{1}H NMR spectrum (400 MHz, DMSO-d_6/THF) of the competitive reaction with formamide **2b**.

7-5. Hydrogenolysis of ester **13**, carbamate **14**, or formamide **2b** (Fig. 3c and Supplementary Fig 11)

A 50 mL stainless steel autoclave, a glass tube, and a stirring bar were dried in an oven at 150 °C, and then cooled inside a glovebox under argon atmosphere. Catalyst **4** (9.4 mg, 15 µmol), ethyl benzoate (**13**) (75.1 mg, 0.50 mmol), octyl phenylcarbamate (**14**) (124.7 mg, 0.50 mmol), or *N*-(4-fluorophenyl)formamide (**2b**) (69.6 mg, 0.50 mmol) and THF (3 mL) were added into the glass tube, and the tube was capped with a funnel to prevent evaporation of the solvent. After the glass tube was set in the autoclave, the autoclave was sealed. The autoclave was brought out from the glovebox. The autoclave was degassed three times using H_2 and was pressurized with 1 MPa of H_2 for 5 min with stirring. The reaction mixture was stirred in an isothermal heating block at 130 °C for 48 h. After cooling to room temperature, H_2 was vented off carefully. To the reaction mixture, DMSO-d_6 (ca. 2 mL) was added to homogenize the reaction mixture, and dibromomethane (86.9 mg, 0.50 mmol) was added as an internal standard. The solution (THF/DMSO-d_6 = 3/2, 0.3 mL) was transferred into an NMR tube and diluted with

DMSO-d_6 (0.3 mL) for NMR analysis. The conversion and yields were determined by ^{1}H NMR spectroscopy with the internal standard.

7-6. Gram-scale hydrogenolysis (Fig. 3e)

A 50 mL stainless steel autoclave and a stirring bar were dried in an oven at 150 °C, and then cooled inside a glovebox under argon atmosphere. Catalyst **4** (9.4 mg, 15 µmol), 1,3-diphenylurea (**1a**) (1.06 g, 5.0 mmol), KO^{t}Bu (5.6 mg, 0.05 mmol), and THF (30 mL) were added into the autoclave. After the autoclave was sealed, it was brought out from the glovebox. The autoclave was degassed three times using H_2 and was pressurized with 1 MPa of H_2 for 5 min with stirring. The reaction mixture was stirred in an isothermal heating block at 130 °C for 48 h. After cooling to room temperature, H_2 was vented off carefully. To the reaction mixture, DMSO-d_6 (ca. 2 mL) and 0.5 M HCl aq. (0.1 mL) were added to homogenize and neutralize the reaction mixture, and dibromomethane (173.8 mg, 1.0 mmol) was added as an internal standard. The solution (THF/DMSO-d_6 = 15/1, 0.2 mL) was transferred into an NMR tube and diluted with DMSO-d_6 (0.4 mL) for NMR analysis. The conversion of **1a** and the yields of formanilide (**2a**) and aniline (**3a**) were determined by ^{1}H NMR spectroscopy with the internal standard.

7-7. Reducing catalyst loading (Fig. 3f)

A 50 mL stainless steel autoclave, a glass tube, and a stirring bar were dried in an oven at 150 °C, and then cooled inside a glovebox under argon atmosphere. A solution of catalyst **4** (3.0 mM in THF, 0.5 mL, 1.5 µmol), 1,3-diphenylurea (**1a**) (353.7 mg, 1.67 mmol), a solution of KO^{t}Bu (0.02 M in THF, 0.25 mL, 5 µmol), and THF (9.25 mL, total: 10.0 mL) were added into the glass tube, and the tube was capped with a funnel to prevent evaporation of the solvent. After the glass tube was set in the autoclave, the autoclave was sealed. The autoclave was brought out from the glovebox. The autoclave was degassed three times using H_2 and was pressurized with 1 MPa of H_2 for 5 min with stirring. The reaction mixture was stirred in an isothermal heating block at 130 °C for 48 h. After cooling to room temperature, H_2 was vented off carefully. To the reaction mixture, DMSO-d_6 (ca. 2 mL) and 0.5 M HCl aq. (0.01 mL) were added to homogenize and neutralize the reaction mixture, and dibromomethane (173.8 mg, 1.0 mmol) was added as an internal standard. The solution (THF/DMSO-d_6 = 5/1, 0.2 mL) was transferred into an NMR tube and diluted with DMSO-d_6 (0.4 mL) for NMR analysis. The conversion of **1a** and the yields of formanilide (**2a**) and aniline (**3a**) were determined by ^{1}H NMR spectroscopy with the internal standard.

7-8. Hydrogenolysis of polyurea **17** (Fig. 5 and Supplementary Tab 5)

Hydrogenolysis of polyurea **17** shown in Fig. 5 (entry 4, Supplementary Tab 5) was conducted as follows: A 50 mL stainless steel autoclave, a glass tube, and a stirring bar were dried in an oven at 150 °C, and then cooled inside a glovebox under argon atmosphere. Catalyst **4** (3.1 mg, 5 µmol), polyurea resin **17** (32.9 mg, 0.16 mmol/urea moiety), KO^{t}Bu (1.9 mg, 0.017 mmol), and THF (3 mL) were added into the glass tube, and the tube was capped with a funnel to prevent evaporation of the solvent. After the glass tube was set in the autoclave, the autoclave was sealed. The autoclave was brought out from the glovebox. The autoclave was degassed three times using H_2 and was pressurized with 1 MPa of H_2 for 5 min with stirring. The reaction mixture was stirred in an isothermal heating block at 130 ℃ for 18 h. After cooling to room temperature, H_2 was vented off carefully. To the reaction mixture, 0.5 M HCl aq. (33 µL) was added to neutralize the reaction mixture, and dibromomethane (57.9 mg, 0.33 mmol) was added as an internal standard. The solution (0.1 mL) was transferred into an NMR tube and added with DMSO-d_6 for NMR analysis. The yields of products were determined by ^{1}H NMR spectroscopy with the internal standard (NMR yields were calculated by subtracting impurities including **17**). The combined reaction mixture was evaporated and extracted with hexane (3 mL × 6). The combined hexane layers were evaporated to obtain *N*,*N*'-dimethyl-1,6-diaminohexane (**16**) as a colorless liquid (5.8 mg, 48%). The residue was purified by silica gel column chromatography with CH_2Cl_2/ethyl acetate as an eluent to obtain *N*,*N*'-(methylenebis(4,1-phenylene))diformamide (**18**) as a white solid (13.8 mg, 65%).

For the condition screening shown in Supplementary Tab 5, entries 1–3 and 5–7 were

conducted following above-mentioned procedure. For entries 1 and 2, no KO^{t}Bu was added, and DMSO-d_6 (ca. 2 mL) was added after reaction to homogenize the reaction mixture. For entries 1–3 and 7, the reaction time was 22 h. The reactions shown in entries 5 and 6 were conducted with shorter reaction time of 14 h and 10 h, respectively. Higher reaction temperature (170 ℃) was employed for entries 2 and 7, and in the latter entry, THF (2 mL) was used as the reaction solvent. The yields of products were determined by ^{1}H NMR spectroscopy with the internal standard (NMR yields were calculated by subtracting impurities including **17**).

7-9. Optimization of reaction conditions using Ir catalyst **4** (Supplementary Tab 1)

A 50 mL stainless steel autoclave, a glass tube, and a stirring bar were dried in an oven at 150 °C, and then cooled inside a glovebox under argon atmosphere. Catalyst **4** (3.1 mg, 5 µmol), 1,3-diphenylurea (**1a**) (35.4 mg, 0.17 mmol), and solvent (2 mL) were added into the glass tube, and the tube was capped with a funnel to prevent evaporation of the solvent. After the glass tube was set in the autoclave, the autoclave was sealed. The autoclave was brought out from the glovebox. The autoclave was degassed three times using H_2 and was pressurized with each pressure of H_2 for 5 min with stirring. The reaction mixture was stirred in an isothermal heating block at each temperature for 18 h. After cooling to room temperature, H_2 was vented off carefully. To the reaction mixture, DMSO-d_6 (ca. 2 mL) was added to homogenize the reaction mixture, and dibromomethane (57.9 mg, 0.33 mmol) was added as an internal standard. The solution (reaction solvent/DMSO-d_6 = 1/1, 0.4 mL) was transferred into an NMR tube and diluted with DMSO-d_6 (0.2 mL) for NMR analysis. The conversion of **1a** and the yields of formanilide (**2a**) and aniline (**3a**) were determined by ^{1}H NMR spectroscopy with the internal standard.

7-10. Optimization of reaction conditions using Ir catalyst **4** (Supplementary Tab 2)

A 50 mL stainless steel autoclave, a glass tube, and a stirring bar were dried in an oven at 150 °C, and then cooled inside a glovebox under argon atmosphere. Catalyst **4** (9.4 mg, 15 µmol), 1,3-diphenylurea (**1a**) (106.1 mg, 0.50 mmol), additive (0.05 mmol, entries 4–8), and solvent (2 mL) were added into the glass tube, and the tube was capped with a funnel to prevent evaporation of the solvent. After the glass tube was set in the autoclave, the autoclave was sealed. The autoclave was brought out from the glovebox. The autoclave was degassed three times using H_2 and was pressurized with 1 MPa of H_2 for 5 min with stirring. The reaction mixture was stirred in an isothermal heating block at 130 °C for 18 h. After cooling to room temperature, H_2 was vented off carefully. To the reaction mixture, DMSO-d_6 (ca. 2 mL) and 0.5 M HCl aq. (0.1 mL, entries 5–8) or 0.5 M NaOH aq. (0.1 mL, entry 4) were added to homogenize and neutralize (entries 4–8) the reaction mixture, and dibromomethane (86.9 mg, 0.50 mmol) was added as an internal standard. The solution (reaction solvent/DMSO-d_6 = 1/1, 0.4 mL) was transferred into an NMR tube and diluted with DMSO-d_6 (0.2 mL) for NMR analysis. The conversion of **1a** and the yields of formanilide (**2a**) and aniline (**3a**) were determined by ^{1}H NMR spectroscopy with the internal standard.

7-11. Hydrogenolysis of urea **1a** using a tautomeric mixture of **23** and **24** (Supplementary Fig 2)

A 50 mL stainless steel autoclave, a glass tube, and a stirring bar were dried in an oven at 150 °C, and then cooled inside a glovebox under argon atmosphere. Mixture of **23** and **24** (22.4 mg, 15 µmol), 1,3-diphenylurea (**1a**) (106.1 mg, 0.50 mmol), and THF or toluene (3 mL) were added into the glass tube, and the tube was capped with a funnel to prevent evaporation of the solvent. After the glass tube was set in the autoclave and the autoclave was sealed, the autoclave was brought out from the glovebox. The autoclave was degassed three times using H_2 and was pressurized with 1 MPa of H_2 for 5 min with stirring. The reaction mixture was stirred in an isothermal heating block at 130 °C for 48 h. After cooling to room temperature, H_2 was vented off carefully. To the reaction mixture, DMSO-d_6 (ca. 1.5 mL) was added to homogenize the reaction mixture, and dibromomethane (86.9 mg, 0.50 mmol) was added as an internal standard. The solution (THF or toluene/DMSO-d_6 = 2/1, 0.3 mL) was transferred into an NMR

tube and diluted with DMSO-d_6 (0.3 mL) for NMR analysis. The conversion of **1a** and the yields of formanilide (**2a**) and aniline (**3a**) were determined by ^{1}H NMR spectroscopy with the internal standard.

7-12. Competitive reaction of urea **1a** with arenes having various functionalities (Supplementary Fig 4)

A 50 mL stainless steel autoclave, a glass tube, and a stirring bar were dried in an oven at 150 °C, and then cooled inside a glovebox under argon atmosphere. Iridium complex **4** (9.4 mg, 15 µmol), 1,3-diphenylurea (**1a**) (106.1 mg, 0.50 mmol) and THF (1.5 mL) were added into the glass tube, and the tube was capped with a funnel to prevent evaporation of the solvent. After the glass tube was set in the autoclave, the autoclave was sealed. The autoclave was brought out from the glovebox, and anisole (10.8 mg, 0.10 mmol), nitrobenzene (12.3 mg, 0.10 mmol), chlorobenzene (11.3 mg, 0.10 mmol), bromobenzene (15.7 mg, 0.10 mmol), and benzonitrile (10.3 mg, 0.10 mmol) in THF solution (1.5 mL) was added into the glass tube under N_2 atmosphere. The autoclave was degassed three times using H_2 and was pressurized with 1 MPa of H_2 for 5 min with stirring. The reaction mixture was stirred in an isothermal heating block at 130 °C for 48 h. After cooling to room temperature, H_2 was vented off carefully. To the reaction mixture, DMSO-d_6 (ca. 2 mL) was added to homogenize the reaction mixture, and dibromomethane (86.9 mg, 0.50 mmol) and decane (14.2 mg, 0.10 mmol) were added as internal standards. The solution (THF/DMSO-d_6 = 3/2, 0.3 mL) was transferred into an NMR tube and diluted with DMSO-d_6 (0.3 mL) for NMR analysis. The conversion of **1a** and the yields of formanilide (**2a**) and aniline (**3a**) were determined by ^{1}H NMR spectroscopy with the internal standard (dibromomethane). The conversions of arenes were determined by GC with the internal standard (decane).

7-13. Thermal decomposition of urea **1a** in Kugelrohr apparatus (Supplementary Fig 9)

1,3-Diphenylurea (**1a**) (212.3 mg, 1.0 mmol) and dehydrated tetraglyme (3 mL) were added into the sample flask, and 1,3,5-trimethoxybenzene (8.4 mg, 0.05 mmol) was added into the receipting flask. The sample flask was rotated at 130 °C for 1 h at 1.7×10^{-2} to 2.3×10^{-2} MPa. To the mixture in the receipting flask, C_6D_6 (0.6 mL) was added and the solution was transferred into an NMR tube for NMR analysis. To the mixture in the sample flask, 1,3,5-trimethoxybenzene (33.6 mg, 0.20 mmol) was added and the solution was transferred into an NMR tube and diluted with C_6D_6 for NMR analysis. The conversion and yields were determined by ^{1}H NMR spectroscopy with the internal standard (1,3,5-trimethoxybenzene). ^{1}H and ^{13}C NMR spectra of the reaction mixture are shown in Supplementary Figs 79–83.

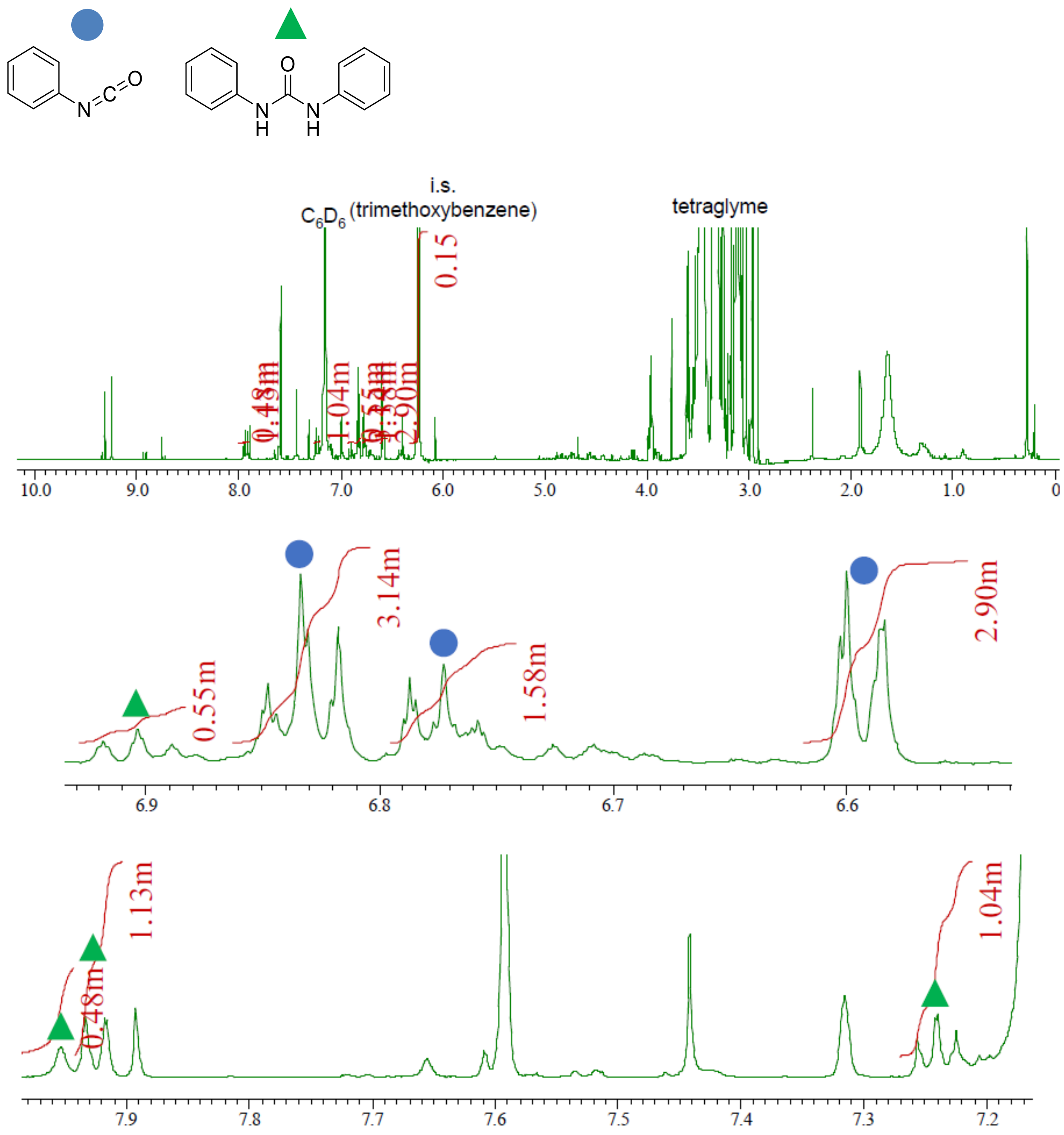

Supplementary Fig 79.

^{1}H NMR spectra (500 MHz, C_6D_6) of the mixture in receipting flask by Kugelrohr distillation of 1,3-diphenylurea (**1a**).

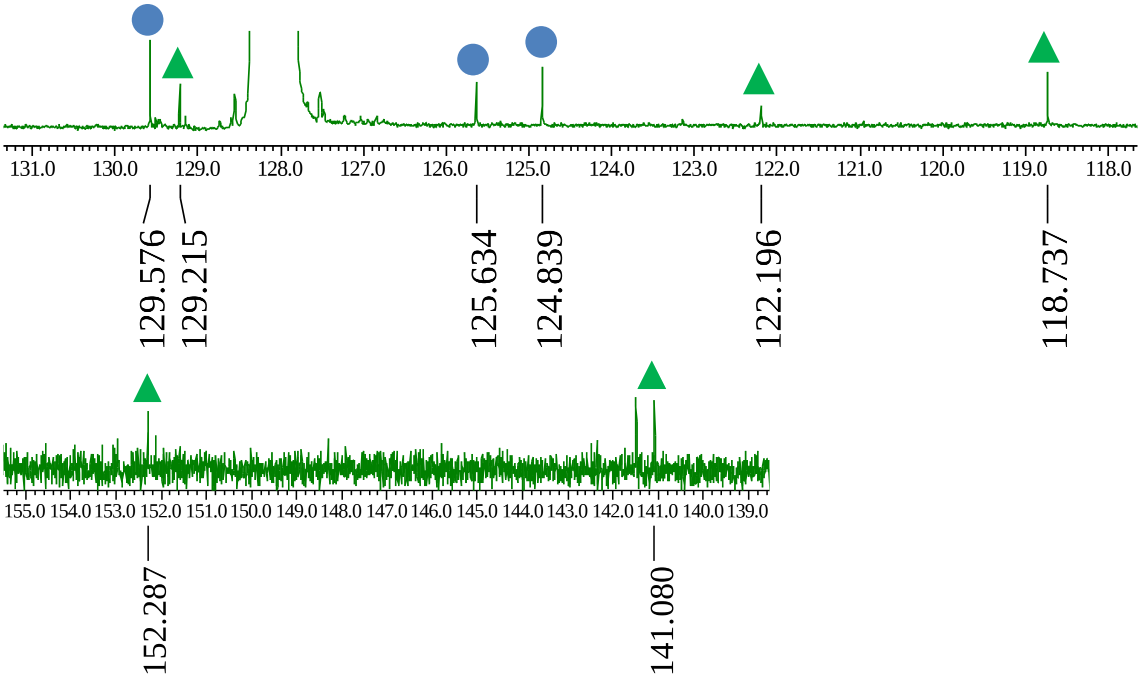

Supplementary Fig 80.
^{13}C NMR spectra (126 MHz, C_6D_6) of the mixture in receipting flask by Kugelrohr distillation of 1,3-diphenylurea (**1a**)

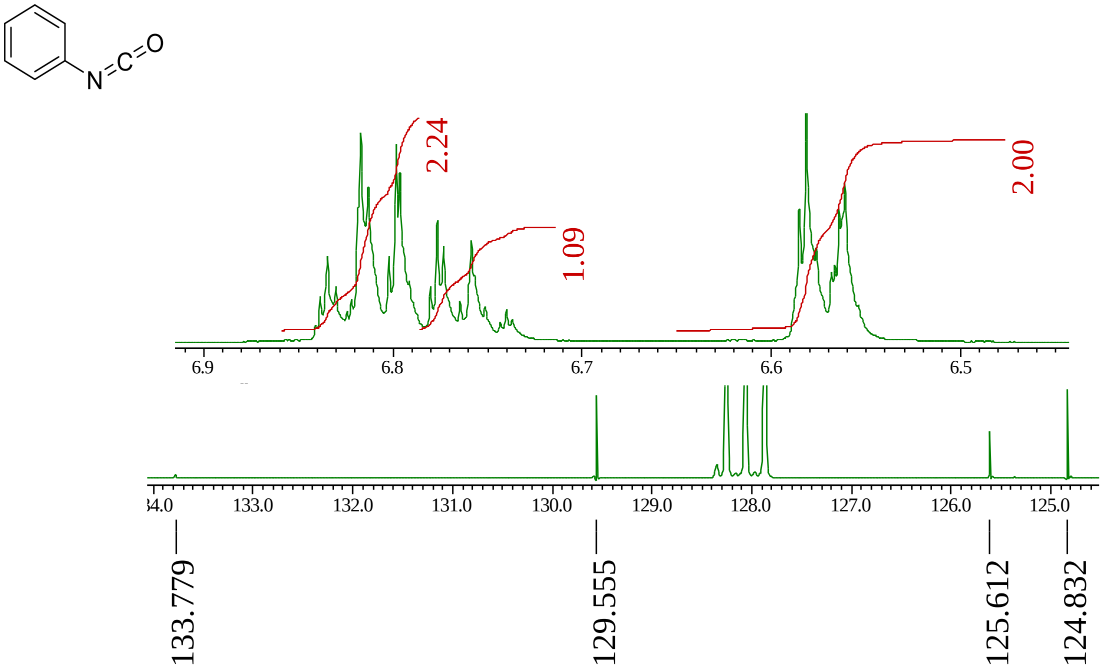

Supplementary Fig 81.
^{1}H and ^{13}C NMR spectra (^{1}H: 500 MHz, ^{13}C: 126 MHz, C_6D_6) of **27a**.

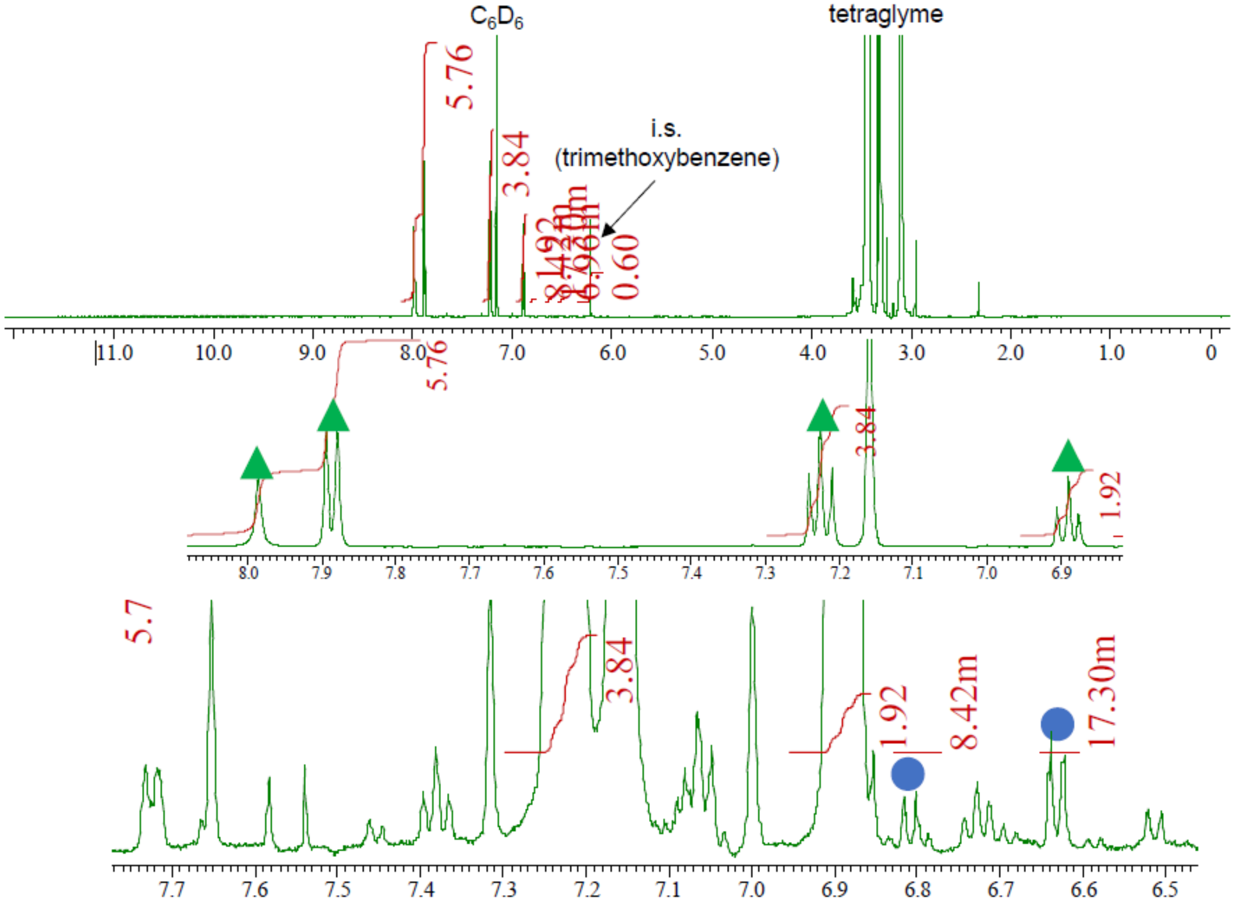

Supplementary Fig 82.

^{1}H NMR spectra (500 MHz, C_6D_6) of the mixture in sample flask by Kugelrohr distillation of 1,3-diphenylurea (**1a**).

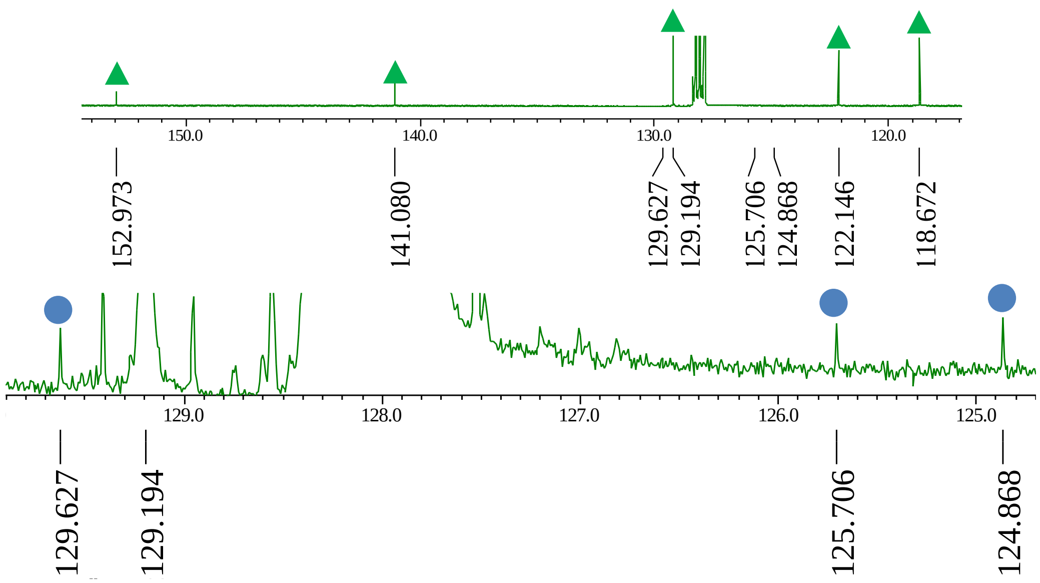

Supplementary Fig 83.

^{13}C NMR spectra (126 MHz, C_6D_6) of the mixture in sample flask by Kugelrohr distillation of 1,3-diphenylurea (**1a**).

7-14. Thermal decomposition of urea **1h** in the presence of phenyl isocyanate (**27a**) (Supplementary Fig 10)

A 50 mL stainless steel autoclave, a glass tube, and a stirring bar were dried in an oven at 150 °C, and then cooled inside a glovebox under argon atmosphere. 1,3-Bis(4-methoxyphenyl)urea (**1h**) (136.2 mg, 0.50 mmol), phenyl isocyanate (**27a**) (59.6 mg, 0.50 mmol), and THF (3 mL) were added into the glass tube, and the tube was capped with a funnel to prevent evaporation of the solvent. After the glass tube was set in the autoclave, the autoclave was sealed. The autoclave was brought out from the glovebox. The autoclave was degassed three times using H_2 and was pressurized with 1 MPa of H_2 for 5 min with stirring. The reaction mixture was stirred in an isothermal heating block at 130 °C for 48 h. After cooling to room temperature, H_2 was vented off carefully. To the reaction mixture, morpholine (0.22 mL, 2.5 mmol) was added under N_2 atmosphere, and the solution was stirred at room temperature for 1 h. To the reaction mixture, DMSO-d_6 (ca. 2 mL) was added to homogenize the reaction mixture, and dibromomethane (86.9 mg, 0.50 mmol) was added as an internal standard. The solution (THF/DMSO-d_6 = 3/2, 0.3 mL) was transferred into an NMR tube and diluted with DMSO-d_6 (0.3 mL) for NMR analysis. The conversions and the yields were determined by ^1H NMR spectroscopy with the internal standard.

7-15. Hydrogenation of phenyl isocyanate (**27a**) (Supplementary Fig 13)

A 50 mL stainless steel autoclave, a glass tube, and a stirring bar were dried in an oven at 150 °C, and then cooled inside a glovebox under argon atmosphere. Catalyst **4** (9.4 mg, 15 µmol), phenyl isocyanate (**27a**) (59.6 mg, 0.50 mmol) and THF (3 mL) were added into the glass tube, and the tube was capped with a funnel to prevent evaporation of the solvent. After the glass tube was set in the autoclave, the autoclave was sealed. The autoclave was brought out from the glovebox. The autoclave was degassed three times using H_2 and was pressurized with 1 MPa of H_2 for 5 min with stirring. The reaction mixture was stirred in an isothermal heating block at 130 °C for 48 h. After cooling to room temperature, H_2 was vented off carefully. To the reaction mixture, 1,3,5-trimethoxybenzene (16.8 mg, 0.10 mmol) was added, and the solution was transferred into an NMR tube and diluted with C_6D_6 for NMR analysis in the

glovebox. The conversion of **27a** and yields of formanilide (**2a**), aniline (**3a**) and 1,3-diphenylurea (**1a**) were determined by ^{1}H NMR spectroscopy with the internal standard (1,3,5-trimethoxybenzene).

To exclude the possibility of the formation of **3a** by the hydrolysis of **27a**, the control experiment without catalyst **4** was also conducted under the same conditions. The control experiment resulted in the 85% recovery of **27a** and 4% yield of **1a**, indicating that the hydrolysis of **27a** is negligible.

7-16. Competitive reaction of phenyl isocyanate (**27a**) with formamide **2b** (Supplementary Fig 14)

A 50 mL stainless steel autoclave, a glass tube, and a stirring bar were dried in an oven at 150 °C, and then cooled inside a glovebox under argon atmosphere. Catalyst **4** (9.4 mg, 15 μmol), phenyl isocyanate (**27a**) (59.6 mg, 0.50 mmol), *N*-(4-fluorophenyl)formamide (**2b**) (69.6 mg, 0.50 mmol), and THF (3 mL) were added into the glass tube, and the tube was capped with a funnel to prevent evaporation of the solvent. After the glass tube was set in the autoclave, the autoclave was sealed. The autoclave was brought out from the glovebox. The autoclave was degassed three times using H_2 and was pressurized with 1 MPa of H_2 for 5 min with stirring. The reaction mixture was stirred in an isothermal heating block at 130 °C for 48 h. After cooling to room temperature, H_2 was vented off carefully. To the reaction mixture, DMSO-d_6 (ca. 2 mL) was added to homogenize the reaction mixture, and decane (71.1 mg, 0.50 mmol) was added as an internal standard. The conversion of **2b** and the yield of formanilide (**2a**) were determined by GC with the internal standard. The conversion of **27a** and the yields of aniline (**3a**) and 1,3-diphenylurea (**1a**) were not quantified.

7-17. Competitive reaction of urea **1a** with aniline (**3a**) (Supplementary Fig 17)

A 50 mL stainless steel autoclave, a glass tube, and a stirring bar were dried in an oven at 150 °C, and then cooled inside a glovebox under argon atmosphere. Catalyst **4** (9.4 mg, 15 μmol), 1,3-diphenylurea (**1a**) (106.1 mg, 0.50 mmol) and THF (1.5 mL) were added into the glass tube, and the tube was capped with a funnel to prevent evaporation of the solvent. After the glass tube was set in the autoclave, the autoclave was sealed. The autoclave was brought out from the glovebox, and aniline (**3a**) (27.9 mg, 0.30 mmol) in THF solution (1.5 mL) was added into the glass tube under N_2 atmosphere. The autoclave was degassed three times using H_2 and was pressurized with 1 MPa of H_2 for 5 min with stirring. The reaction mixture was stirred in an isothermal heating block at 130 °C for 12 h. After cooling to room temperature, H_2 was vented off carefully. To the reaction mixture, DMSO-d_6 (ca. 2 mL) was added to homogenize the reaction mixture, and dibromomethane (86.9 mg, 0.50 mmol) was added as an internal standard. The solution (THF/DMSO-d_6 = 3/2, 0.3 mL) was transferred into an NMR tube and diluted with DMSO-d_6 (0.3 mL) for NMR analysis. The conversion and yields were determined by ^{1}H NMR spectroscopy with the internal standard.

7-18. The reaction of Ir complex **4** with H_2 (Supplementary Fig 18)

Ir complex **4** (9.4 mg, 15 μmol) and THF-d_8 (0.2 mL) were added into the high pressure valved NMR tube. The NMR tube was degassed five times using H_2 and was pressurized with 0.5 MPa of H_2. After the reaction at room temperature for 16 h, complex mixture was observed by NMR analysis. After further reaction at 60 °C for 21 h, complex mixture was still observed on NMR spectra. ^{1}H and ^{31}P NMR spectra of the reaction of **4** with H_2 are shown in Supplementary Fig 84. When 2 equiv of 1,3-dimethyl-2-imidazolidinone (DMI) (3.4 mg, 30 μmol) were added to the reaction under the same conditions, complex mixture was also observed after the reaction (Supplementary Fig 85).

Ir complex **4** (9.4 mg, 15 μmol), triphenylphosphine (7.9 mg, 30 μmol), and THF-d_8 (0.5 mL) were added into the J. Young valved NMR tube. The NMR tube was degassed three times using H_2 and was filled with 0.1 MPa of H_2. After the reaction at 0 °C for 1.5 h and then at room temperature for 18 h, the mixture of Ir complex **28** and **29** in 85:15 ratio was observed on NMR spectra. ^{1}H and ^{31}P NMR spectra of the reaction of **4** with H_2 in the presence of PPh_3 are shown in Supplementary Fig 86. After further reaction at room temperature for 12 h and then 50 °C for 12 h, only Ir complex **28** was observed on NMR

spectra (93% purity, ^{31}P NMR). ^{1}H and ^{31}P NMR spectra of the reaction of **4** with H_2 in the presence of PPh_3 after heating at 50 °C are shown in Supplementary Fig 87.

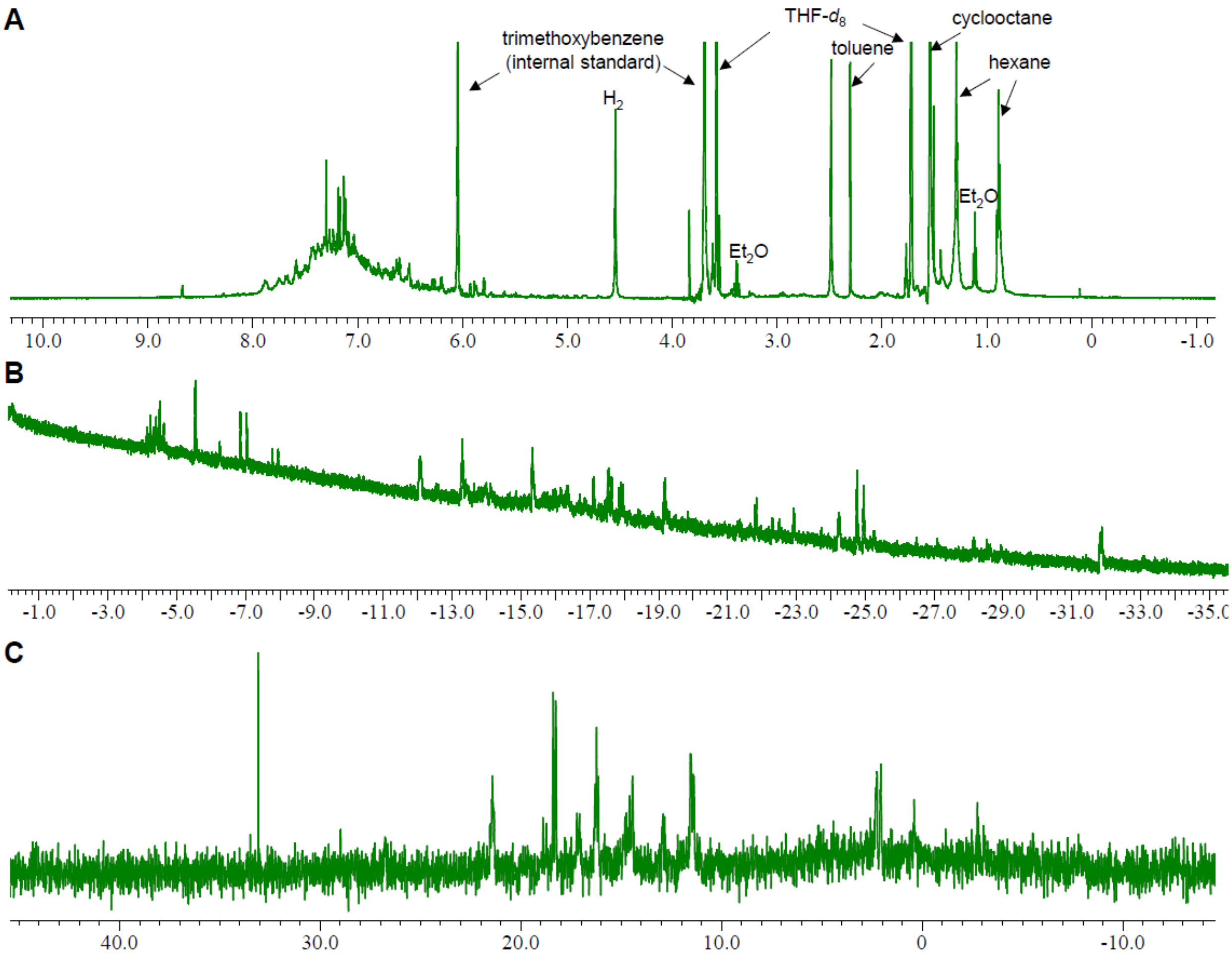

Supplementary Fig 84.

^{1}H and ^{31}P NMR spectra of the reaction of **4** with H_2. (**A**) ^{1}H NMR (500 MHz, THF-d_8, 25 °C). (**B**) Hydride region of ^{1}H NMR. (**C**) ^{31}P NMR (202 MHz, THF-d_8, 25 °C).

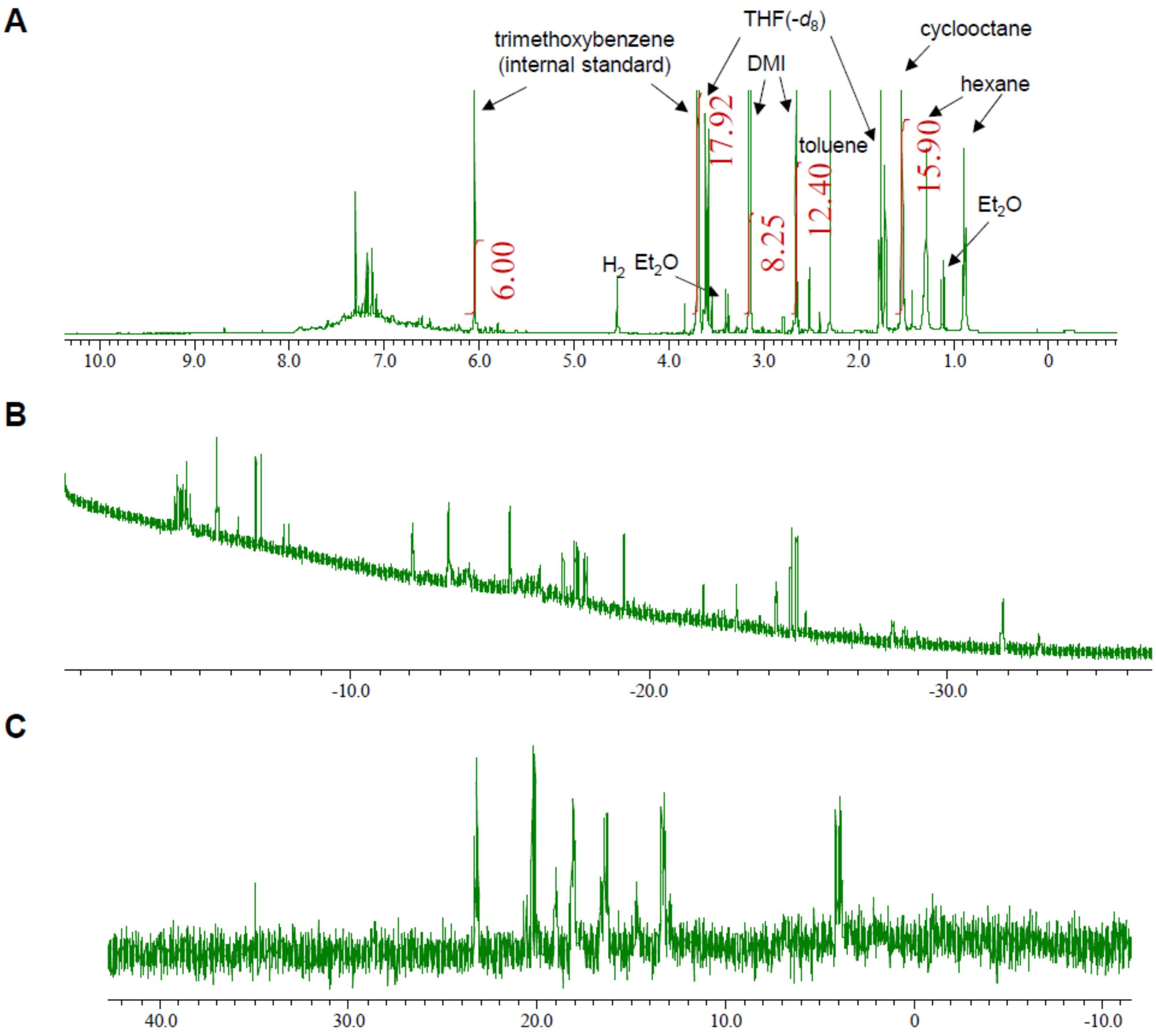

Supplementary Fig 85.

^{1}H and ^{31}P NMR spectra of the reaction of **4** with H_2 in the presence of DMI. (**A**) ^{1}H NMR (500 MHz, THF-d_8, 25 °C). (**B**) Hydride region of ^{1}H NMR. (**C**) ^{31}P NMR (202 MHz, THF-d_8, 25 °C).

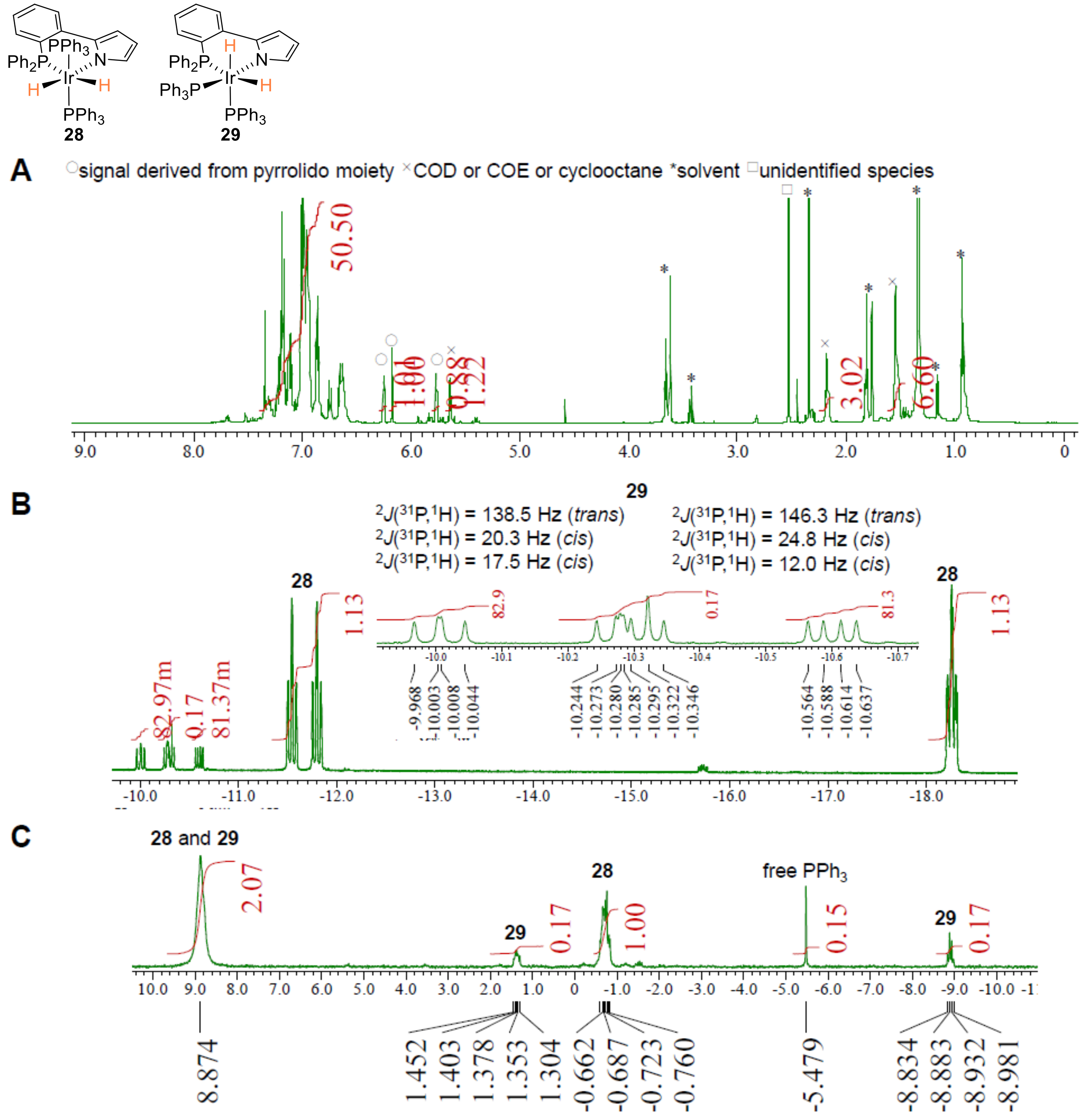

Supplementary Fig 86.

^{1}H and ^{31}P NMR spectra of the reaction of **4** with H_2 in the presence of PPh_3. (**A**) ^{1}H NMR (500 MHz, THF-d_8, 25 °C). (**B**) Hydride region of ^{1}H NMR. (**C**) ^{31}P NMR (202 MHz, THF-d_8, 25 °C).

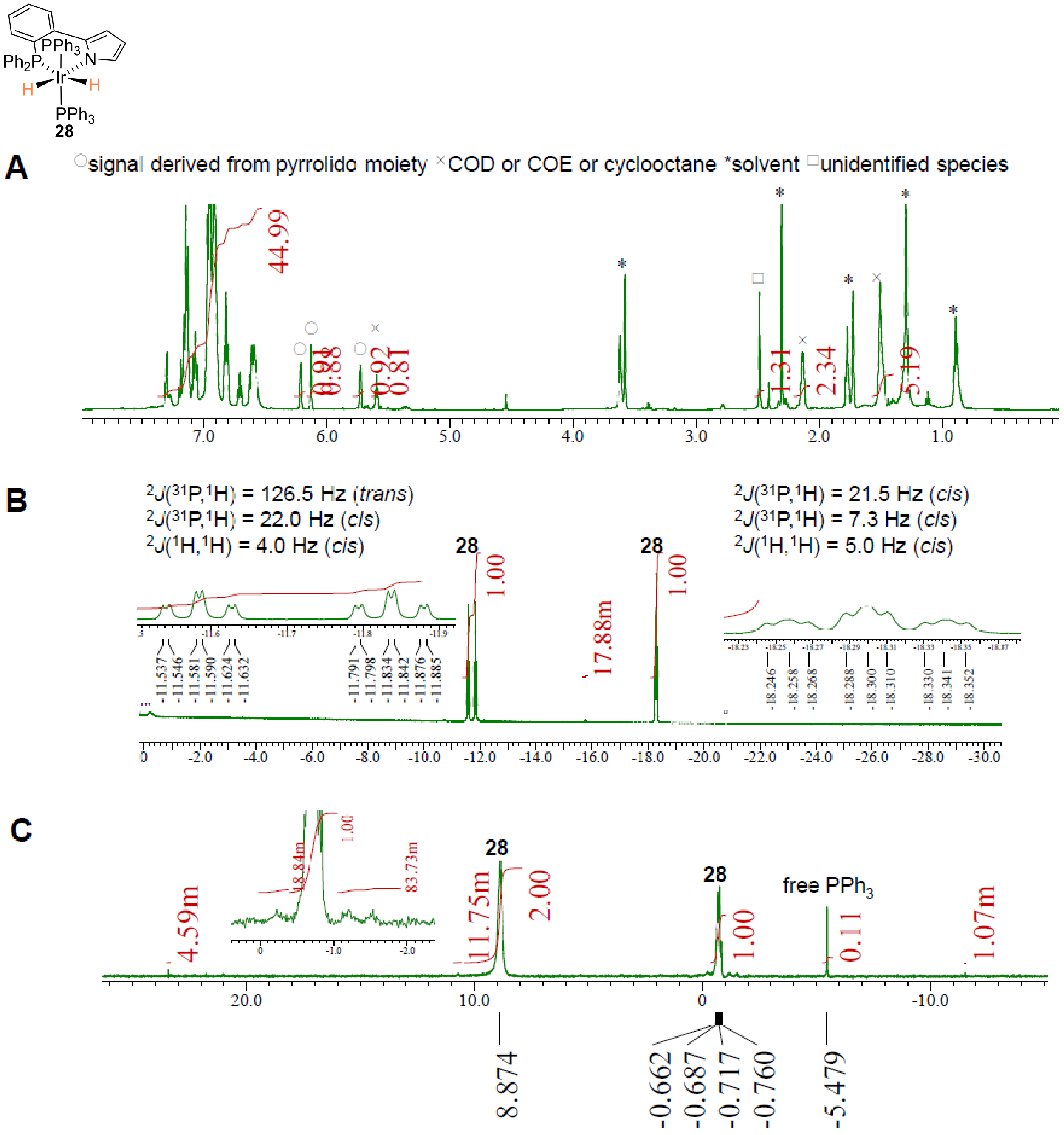

Supplementary Fig 87.

^1H and ^{31}P NMR spectra of the reaction of **4** with H_2 in the presence of PPh_3 after heating at 50 °C. (**A**) ^1H NMR (500 MHz, THF-d_8, 25 °C). (**B**) Hydride region of ^1H NMR. (**C**) ^{31}P NMR (202 MHz, THF-d_8, 25 °C).

7-19. Formyl group transfer under the neutral conditions (Supplementary Figs 19 and 20)

A 50 mL stainless steel autoclave, a glass tube, and a stirring bar were dried in an oven at 150 °C, and then cooled inside a glovebox under argon atmosphere. Formanilide (**2a**) (60.6 mg, 0.50 mmol) or *N*-methylformanilide (**2o**) (67.6 mg, 0.50 mmol) and THF (1.5 mL) were added into the glass tube, and the tube was capped with a funnel to prevent evaporation of the solvent. After the glass tube was set in the autoclave, the autoclave was sealed. The autoclave was brought out from the glovebox, and *N*-methylaniline (**3o**) (53.6 mg, 0.50 mmol), aniline (**3a**) (46.6 mg, 0.50 mmol), or morpholine (**3s**) (43.6 mg, 0.50 mmol) in THF solution (1.5 mL) was added into the glass tube under N_2 atmosphere. The autoclave was degassed three times using H_2 and was pressurized with 1 MPa of H_2 for 5 min with stirring. The reaction mixture was stirred in an isothermal heating block at 130 °C for 48 h. After cooling to room temperature, H_2 was vented off carefully. To the reaction mixture, DMSO-d_6 (ca. 2 mL) was added to homogenize the reaction mixture, and dibromomethane (86.9 mg, 0.50 mmol) was added as an internal standard. The solution (THF/DMSO-d_6 = 3/2, 0.3 mL) was transferred into an NMR tube and diluted with DMSO-d_6 (0.3 mL) for NMR analysis. The conversions and yields were determined by ^{1}H NMR spectroscopy with the internal standard.

7-20. Formyl group transfer under the basic conditions (Supplementary Figs 19 and 20)

A 50 mL stainless steel autoclave, a glass tube, and a stirring bar were dried in an oven at 150 °C, and then cooled inside a glovebox under argon atmosphere. Formanilide (**2a**) (60.6 mg, 0.50 mmol) or *N*-methylformanilide (**2o**) (67.6 mg, 0.50 mmol), KO^{t}Bu (5.6 mg, 0.05 mmol), and toluene (1.5 mL) were added into the glass tube, and the tube was capped with a funnel to prevent evaporation of the solvent. After the glass tube was set in the autoclave, the autoclave was sealed. The autoclave was brought out from the glovebox, and *N*-methylaniline (**3o**) (53.6 mg, 0.50 mmol), aniline (**3a**) (46.6 mg, 0.50 mmol), or morpholine (**3s**) (43.6 mg, 0.50 mmol) in toluene solution (1.5 mL) was added into the glass tube under N_2 atmosphere. The autoclave was degassed three times using H_2 and was pressurized with 1 MPa of H_2 for 5 min with stirring. The reaction mixture was stirred in an isothermal heating block at 130 °C for 48 h. After cooling to room temperature, H_2 was vented off carefully. To the reaction mixture, DMSO-d_6 (ca. 2 mL) and 0.5 M HCl aq. (0.1 mL) were added to homogenize and neutralize the reaction mixture, and dibromomethane (86.9 mg, 0.50 mmol) was added as an internal standard. The solution (toluene/DMSO-d_6 = 3/2, 0.3 mL) was transferred into an NMR tube and diluted with DMSO-d_6 (0.3 mL) for NMR analysis. The conversions and yields were determined by ^{1}H NMR spectroscopy with the internal standard.

8. Characterization of Products

8-1. The hydrogenolysis of 1,3-diphenylurea (**1a**) (Condition A)

The general procedure for Condition A was followed using 1,3-diphenylurea (**1a**) (106.1 mg, 0.50 mmol) and **4** (9.4 mg, 15 µmol) in THF (3 mL) at 130 °C under H_2 (1 MPa) for 48 h. Conversion and yields were determined by ^{1}H NMR analysis by comparing with authentic samples. Hydrochloride of aniline (**3a•HCl**) was isolated as a white solid (50.1 mg, 77%). Formanilide (**2a**) was isolated by silica gel column chromatography (CH_2Cl_2/ethyl acetate) as an yellow solid (33.8 mg, 56%).

^{1}H NMR spectrum of the hydrogenolysis of **1a** under Condition A is shown in Supplementary Fig 88.

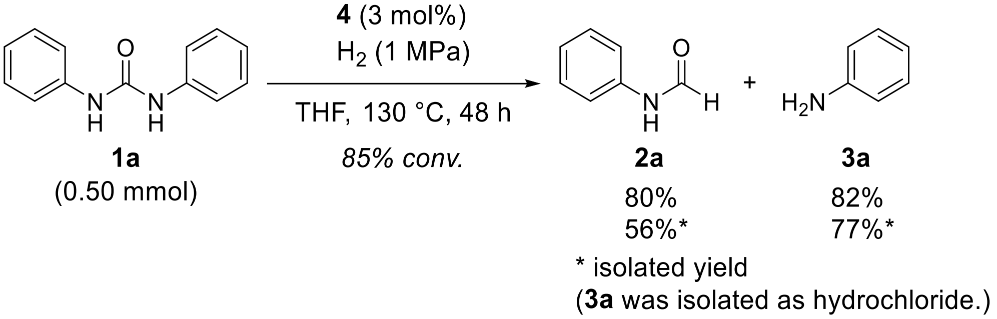

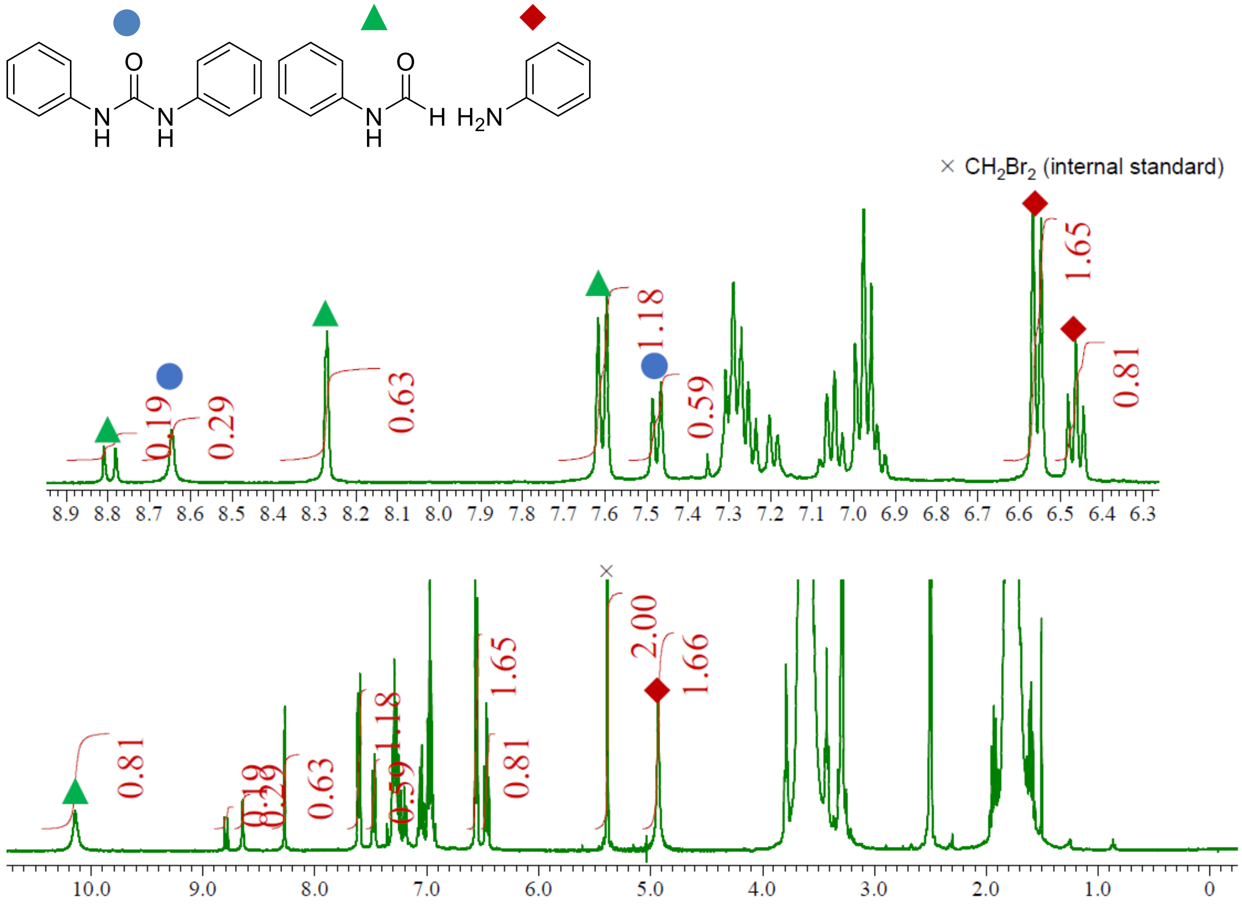

Supplementary Fig 88.

^{1}H NMR spectrum (400 MHz, DMSO-d_6/THF) of the hydrogenolysis of 1,3-diphenylurea (**1a**) (Condition A).

Formanilide (2a)

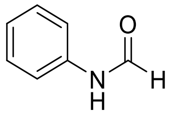

^{1}H NMR (500 MHz, $CDCl_3$): δ 8.70 (d, *J* = 11.5 Hz, 0.5H), 8.38 (s, 0.5H), 8.18 (br, 0.5H), 7.55–7.54 (m, 1H), 7.38–7.32 (m, 2H), 7.21–7.18 (m, 0.5H), 7.16–7.13 (m, 0.5H), 7.10–7.09 (m, 1H); ^{13}C NMR (126 MHz, $CDCl_3$): δ 162.7, 159.1, 136.9, 136.8, 129.9, 129.3, 125.5, 125.0, 120.1, 119.0.
Two tautomers were observed in approximately 1:1 ratio. These data were consistent with those in literature[59,60].

Aniline hydrochloride (3a•HCl)

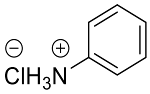

^{1}H NMR (500 MHz, DMSO-d_6): δ 10.34 (br, 3H), 7.47 (dd, *J* = 9.3, 9.3 Hz, 2H), 7.38–7.36 (m, 3H); ^{13}C NMR (126 MHz, DMSO-d_6): δ 132.5, 129.7, 127.6, 122.9.
These data were consistent with those in literature[61].

8-2. The hydrogenolysis of 1,3-diphenylurea (**1a**) (Condition B)

The general procedure for Condition B was followed using 1,3-diphenylurea (**1a**) (106.1 mg, 0.50 mmol), **4** (3.1 mg, 5 µmol), and KO^{t}Bu (5.6 mg, 0.05 mmol) in toluene (2 mL) at 130 °C under H_2 (1 MPa) for 18 h. Conversion and yields were determined by ^{1}H NMR analysis by comparing with authentic samples.

^{1}H NMR spectrum of the hydrogenolysis of **1a** under Condition B is shown in Supplementary Fig 89.

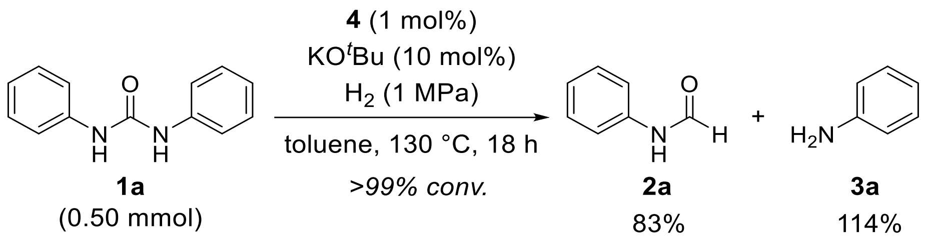

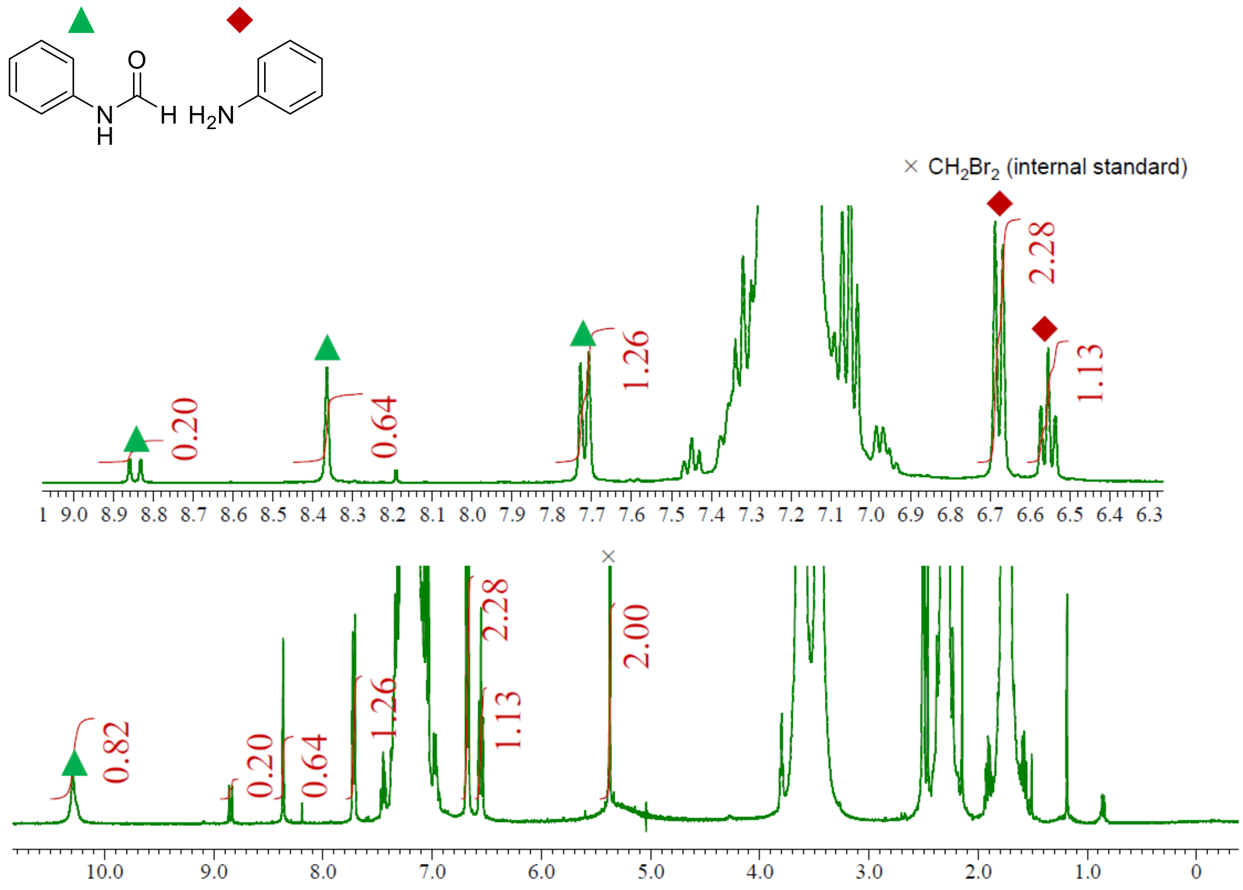

Supplementary Fig 89.

^{1}H NMR spectrum (400 MHz, DMSO-d_6/THF/toluene) of the hydrogenolysis of 1,3-diphenylurea (**1a**) (Condition B).

8-3. The hydrogenolysis of 1,3-bis(4-fluorophenyl)urea (**1b**)

The general procedure for Condition A was followed using 1,3-bis(4-fluorophenyl)urea (**1b**) (124.1 mg, 0.50 mmol) and **4** (9.4 mg, 15 µmol) in THF (3 mL) at 130 °C under H_2 (1 MPa) for 48 h. Conversion and yields were determined by ^{1}H NMR analysis by comparing with authentic samples. Hydrochloride of 4-fluoroaniline (**3b•HCl**) was isolated as a white solid (72.8 mg, 97%). *N*-(4-fluorophenyl)formamide (**2b**) was isolated by silica gel column chromatography (CH_2Cl_2/ethyl acetate) as a white solid (53.3 mg, 77%).

^{1}H NMR spectrum of the hydrogenolysis of **1b** is shown in Supplementary Fig 90.

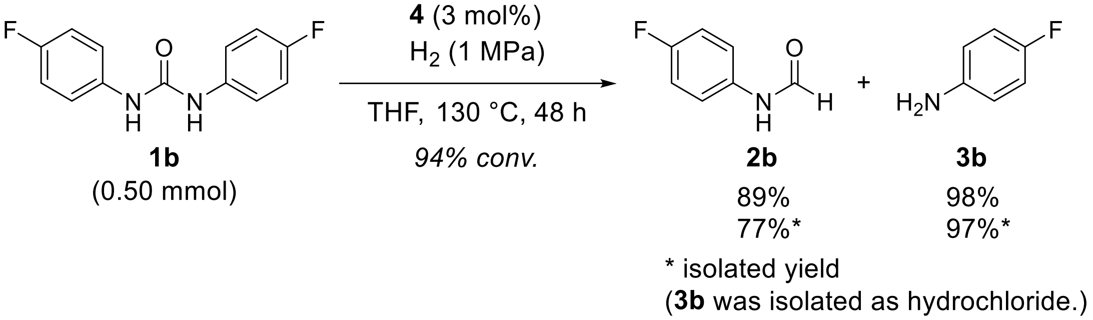

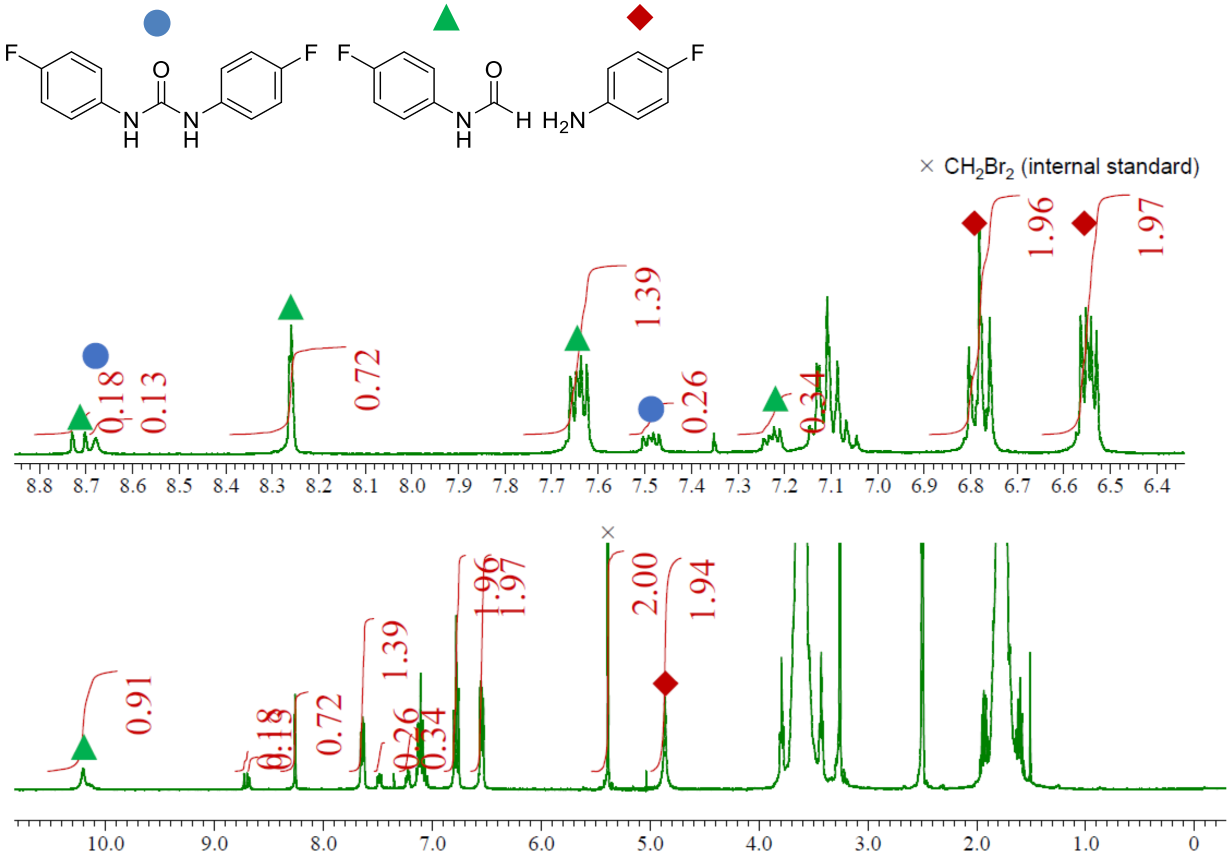

Supplementary Fig 90.

^{1}H NMR spectrum (400 MHz, DMSO-d_6/THF) of the hydrogenolysis of 1,3-bis(4-fluorophenyl)urea (**1b**).

N-(4-Fluorophenyl)formamide (2b)

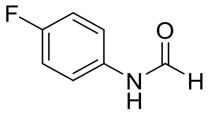

^{1}H NMR (500 MHz, $CDCl_3$): δ 8.57 (d, J = 11.5 Hz, 0.4H), 8.36 (s, 0.6H), 7.85 (br, 0.4H), 7.52–7.49 (m, 1.2H), 7.23 (br, 0.6H), 7.08–7.02 (m, 2.8H); ^{13}C NMR (126 MHz, $CDCl_3$): δ 162.7, 161.6 (C–F), 160.7 (C–F), 159.6 (C–F), 158.9, 158.8 (C–F), 132.9, 132.9, 132.7, 132.7, 121.9, 121.9, 121.5, 121.4, 116.8, 116.7, 116.1, 115.9; ^{19}F NMR (471 MHz, $CDCl_3$): δ −116.73, −116.75, −116.76, −116.77, −116.79, −117.04, −117.05, −117.06, −117.07, −117.08, −117.09, −117.10.
Two tautomers were observed in approximately 2:3 ratio. These data were consistent with those in literature[62].

4-Fluoroaniline hydrochloride (3b•HCl)

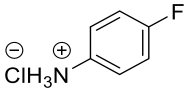

^{1}H NMR (500 MHz, DMSO-d_6): δ 10.17 (br, 3H), 7.42–7.39 (m, 2H), 7.33–7.30 (m, 2H); ^{13}C NMR (126 MHz, DMSO-d_6): δ 160.8 (d, J = 244 Hz), 129.1, 124.8 (d, J = 9.1 Hz), 116.5 (d, J = 22.7 Hz); ^{19}F NMR (471 MHz, CD_3OD): δ −114.29, −114.30, −114.31, −114.32, −114.33, −114.34, −114.35.
These data were consistent with those in literature[61,63].

8-4. The hydrogenolysis of 1,3-bis(4-chlorophenyl)urea (**1c**)

The general procedure for Condition A was followed using 1,3-bis(4-chlorophenyl)urea (**1c**) (140.6 mg, 0.50 mmol) and **4** (9.4 mg, 15 µmol) in THF (3 mL) at 130 °C under H_2 (1 MPa) for 144 h. Conversion and yields were determined by ^{1}H NMR analysis by comparing with authentic samples. The reaction mixture was concentrated and purified by silica gel column chromatography (CH_2Cl_2/ethyl acetate) to obtain *N*-(4-chlorophenyl)formamide (**2c**) as an yellow solid (59.7 mg, 77%) and 4-chloroaniline (**3c**) as an yellow solid (45.0 mg, 71%).

^{1}H NMR spectrum of the hydrogenolysis of **1c** is shown in Supplementary Fig 91.

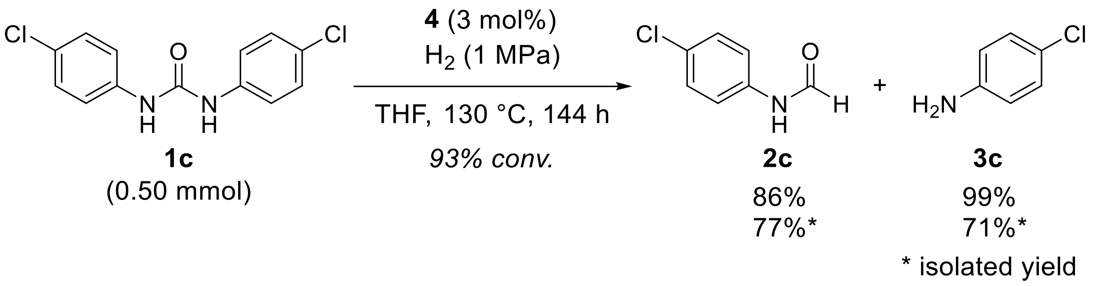

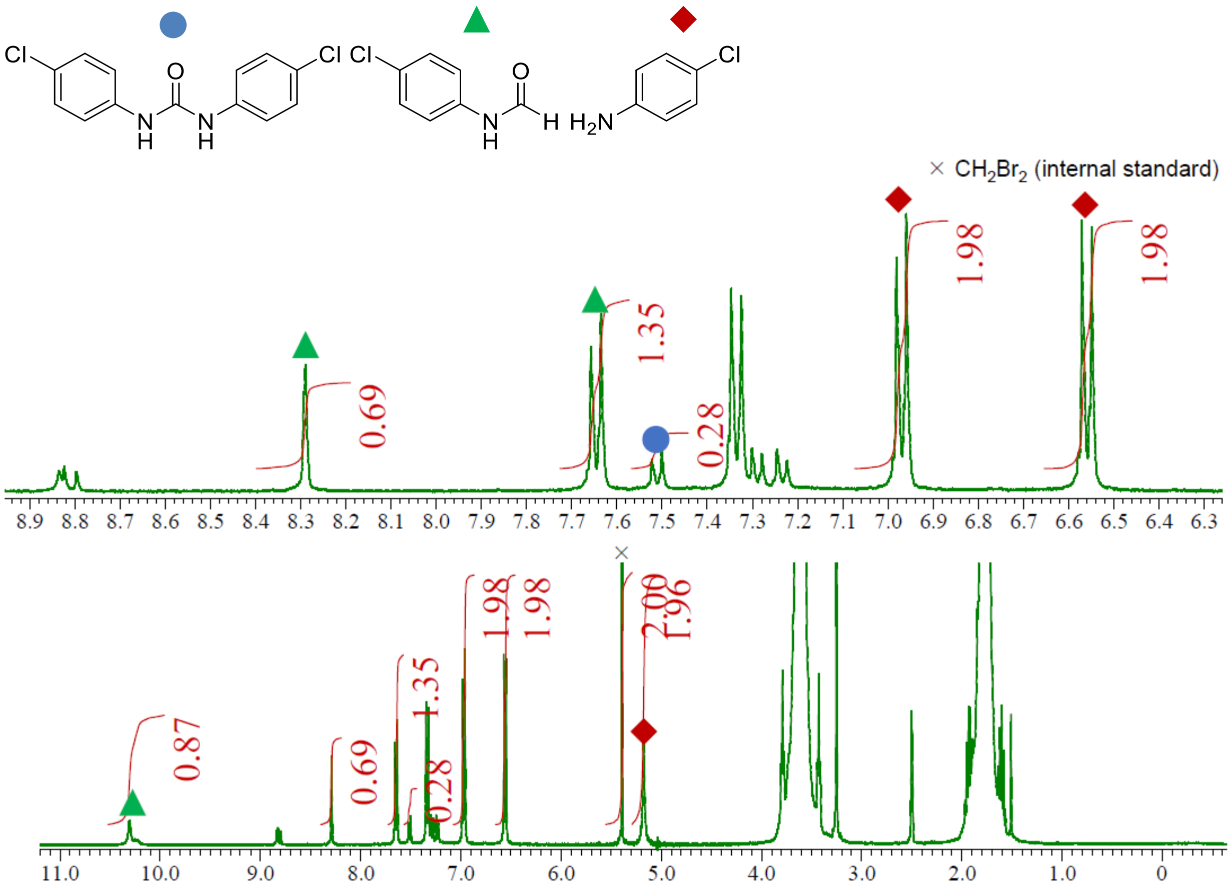

Supplementary Fig 91.

^{1}H NMR spectrum (400 MHz, DMSO-d_6/THF) of the hydrogenolysis of 1,3-bis(4-chlorophenyl)urea (**1c**).

_N_-(4-Chlorophenyl)formamide (2c)

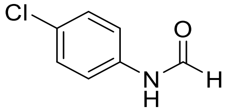

^{1}H NMR (500 MHz, $CDCl_3$): δ 8.65 (d, J = 11.0 Hz, 0.4H), 8.52 (br, 0.4H), 8.36 (d, J = 1.5 Hz, 0.6H), 7.57 (br, 0.6H), 7.49 (d, J = 8.5 Hz, 1.2H), 7.33–7.28 (m, 2H), 7.04 (d, J = 9.0 Hz, 0.8H); ^{13}C NMR (126 MHz, $CDCl_3$): δ 162.7, 159.2, 135.5, 135.4, 130.9, 130.0, 130.0, 129.3, 121.3, 120.2.
Two tautomers were observed in approximately 2:3 ratio. These data were consistent with those in literature[62].

4-Chloroaniline (3c)

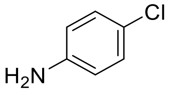

^{1}H NMR (500 MHz, $CDCl_3$): δ 7.10 (d, J = 9.0 Hz, 2H), 6.61 (d, J = 8.5 Hz, 2H), 3.64 (br, 2H); ^{13}C NMR (126 MHz, $CDCl_3$): δ 145.0, 129.2, 123.3, 116.4.
These data were consistent with those in literature[64].

8-5. The hydrogenolysis of 1,3-bis(3-chlorophenyl)urea (**1d**)

The general procedure for Condition A was followed using 1,3-bis(3-chlorophenyl)urea (**1d**) (140.6 mg, 0.50 mmol) and **4** (9.4 mg, 15 µmol) in THF (3 mL) at 130 °C under H_2 (1 MPa) for 48 h. Conversion and yields were determined by ^{1}H NMR analysis by comparing with authentic samples. Hydrochloride of 3-chloroaniline (**3d•HCl**) was isolated as a white solid (63.0 mg, 77%). *N*-(3-chlorophenyl)formamide (**2d**) was isolated by silica gel column chromatography (CH_2Cl_2/ethyl acetate) as a white solid (36.0 mg, 46%).

^{1}H NMR spectrum of the hydrogenolysis of **1d** is shown in Supplementary Fig 92.

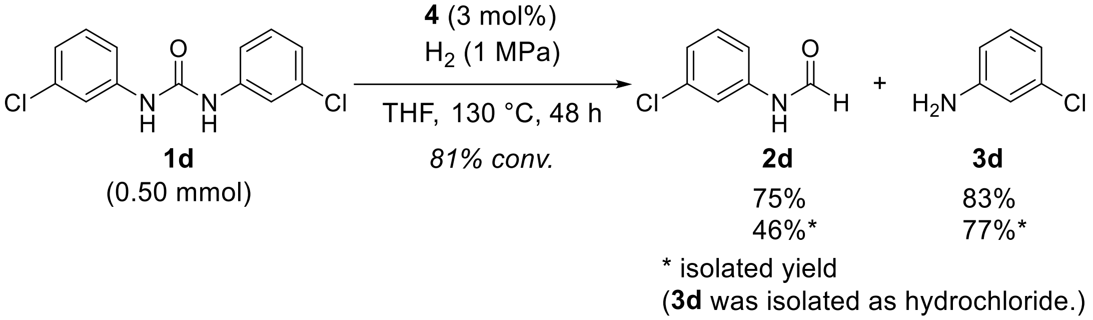

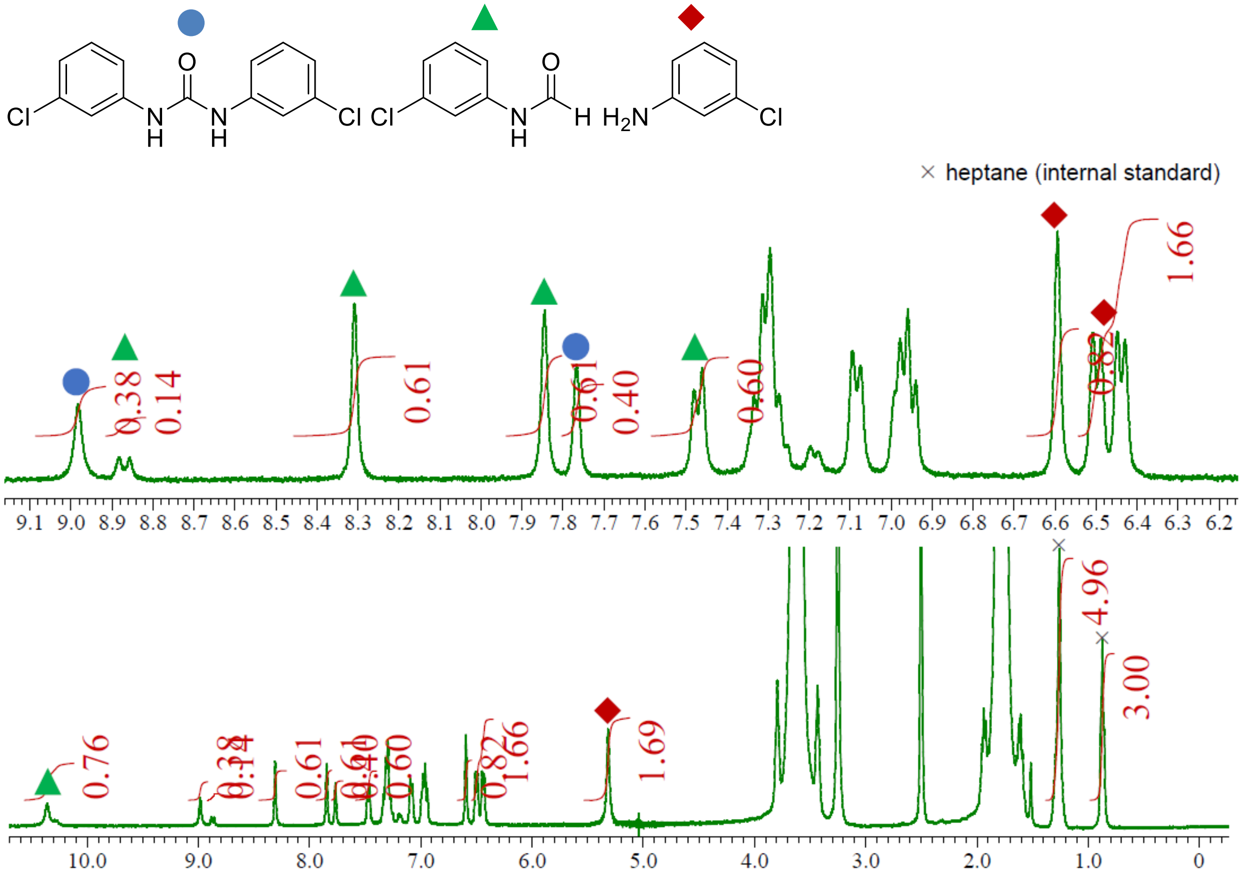

Supplementary Fig 92.

^{1}H NMR spectrum (400 MHz, DMSO-d_6/THF) of the hydrogenolysis of 1,3-bis(3-chlorophenyl)urea (**1d**).

N-(3-Chlorophenyl)formamide (2d)

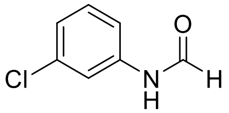

^{1}H NMR (500 MHz, $CDCl_3$): δ 8.70 (d, J = 14.0 Hz, 0.4H), 8.38 (s, 0.6H), 8.35 (br, 0.4H), 7.66 (s, 0.6H), 7.44 (br, 0.6H), 7.39 (d, J = 10.0 Hz, 0.6H), 7.27 (dt, J = 17.5, 9.4 Hz, 0.8H), 7.18–7.11 (m, 1.6H), 6.98 (d, J = 10.0 Hz, 0.4H); ^{13}C NMR (126 MHz, $CDCl_3$): δ 162.5, 159.1, 138.0, 138.0, 135.6, 134.9, 131.0, 130.3, 125.5, 125.1, 120.2, 118.9, 118.0, 116.8.
Two tautomers were observed in approximately 2:3 ratio. These data were consistent with those in literature[62].

3-Chloroaniline hydrochloride (3d•HCl)

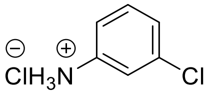

^{1}H NMR (500 MHz, D_2O): δ 7.50–7.45 (m, 3H), 7.33–7.30 (m, 1H); ^{13}C NMR (126 MHz, D_2O): δ 134.8, 131.4, 131.2, 129.0, 123.0, 121.2.
These data were consistent with those in literature[65].

8-6. The hydrogenolysis of 1,3-bis(2-chlorophenyl)urea (**1e**)

The general procedure for Condition A was followed using 1,3-bis(2-chlorophenyl)urea (**1e**) (140.6 mg, 0.50 mmol) and **4** (9.4 mg, 15 µmol) in THF (3 mL) at 130 °C under H_2 (1 MPa) for 144 h. Conversion and yields were determined by ^{1}H NMR analysis by comparing with authentic samples. Hydrochloride of 2-chloroaniline (**3e•HCl**) was isolated as a white solid (31.0 mg, 38%). *N*-(2-chlorophenyl)formamide (**2e**) was isolated by silica gel column chromatography (CH_2Cl_2/ethyl acetate) as a white solid (38.0 mg, 49%).

^{1}H NMR spectrum of the hydrogenolysis of **1e** is shown in Supplementary Fig 93.

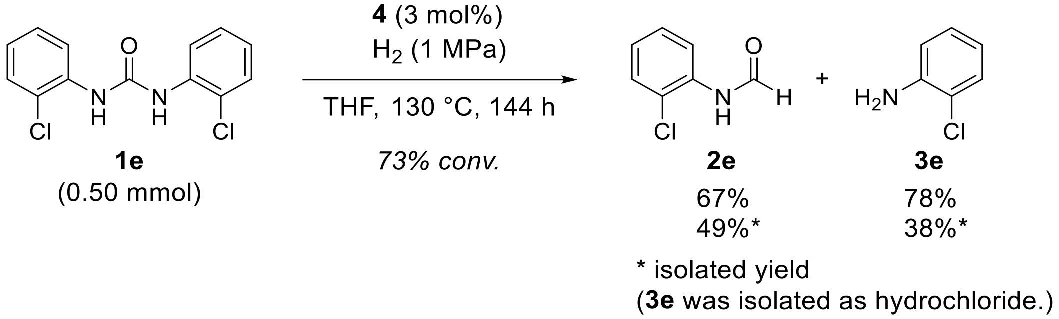

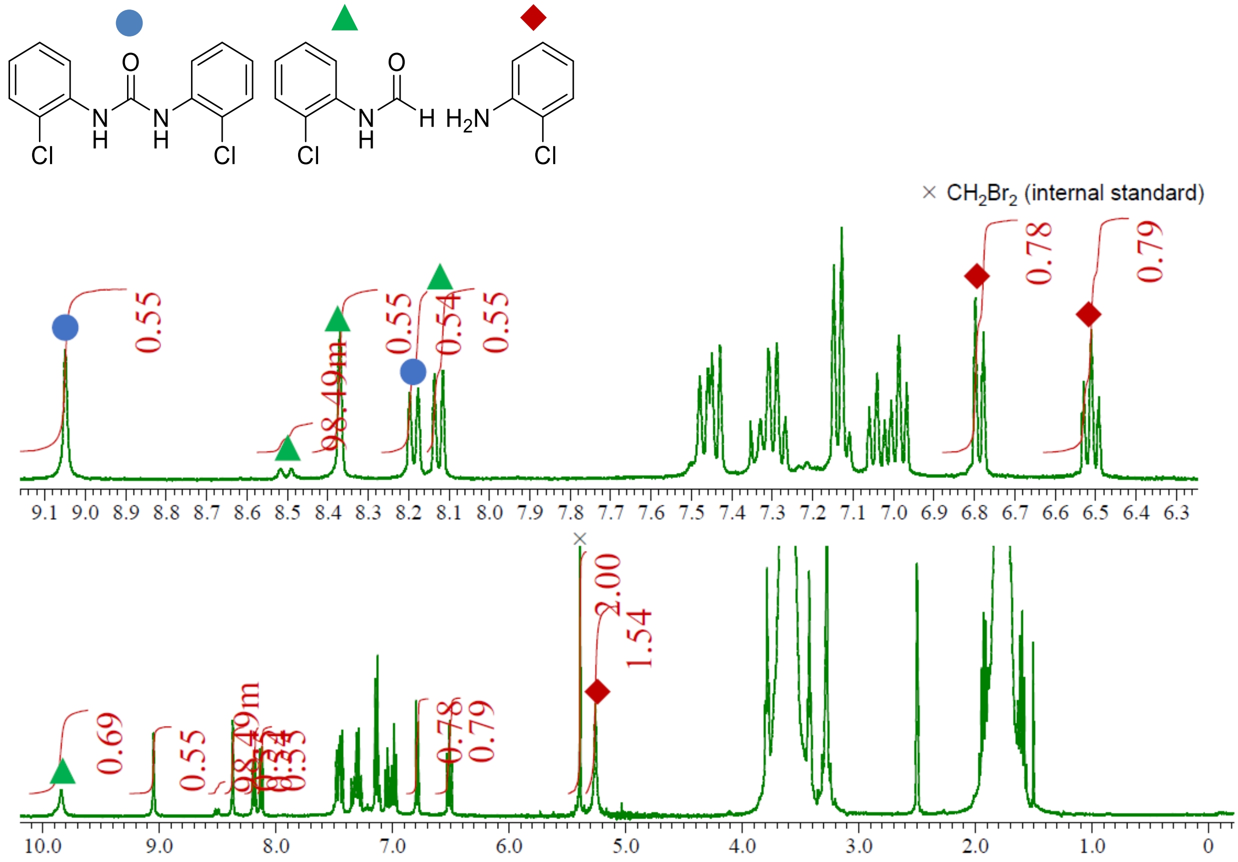

Supplementary Fig 93.

^{1}H NMR spectrum (400 MHz, DMSO-d_6/THF) of the hydrogenolysis of 1,3-bis(2-chlorophenyl)urea (**1e**).

_N_-(2-Chlorophenyl)formamide (2e)

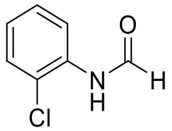

^{1}H NMR (500 MHz, $CDCl_3$): δ 8.72 (d, J = 11.0 Hz, 0.5H), 8.50 (s, 1H), 8.41 (d, J = 8.0 Hz, 1H), 7.73 (br, 1.5H), 7.44–7.37 (m, 1.5H), 7.30–7.26 (m, 2H), 7.16–7.06 (m, 1.5H); ^{13}C NMR (126 MHz, $CDCl_3$): δ 161.5, 158.9, 133.9, 133.8, 130.4, 129.2, 128.2, 128.0, 126.0, 125.3, 124.3, 122.6, 122.1, 118.7.
Two tautomers were observed in approximately 1:2 ratio. These data were consistent with those in literature[66].

3-Chloroaniline hydrochloride (3e•HCl)

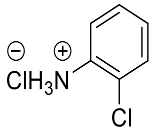

^{1}H NMR (500 MHz, D_2O): δ 7.62–7.59 (m, 1H), 7.49–7.42 (m, 3H); ^{13}C NMR (126 MHz, D_2O): δ 130.5, 129.8, 128.7, 128.5, 127.1, 124.1.
These data were consistent with those in literature[65].

8-7. The hydrogenolysis of 1,3-bis(4-bromophenyl)urea (**1f**)

The general procedure for Condition B was followed using 1,3-bis(4-bromophenyl)urea (**1f**) (185.0 mg, 0.50 mmol), **4** (31.3 mg, 50 µmol), and KO^{t}Bu (18.7 mg, 0.17 mmol) in toluene (3 mL) at 130 °C under H_2 (1 MPa) for 48 h. Conversion and yields were determined by ^{1}H NMR analysis by comparing with authentic samples. The reaction mixture was concentrated and purified by silica gel column chromatography (CH_2Cl_2/ethyl acetate) to obtain *N*-(4-bromophenyl)formamide (**2f**) as a white solid (36.5 mg, 36%) and 4-bromoaniline (**3f**) as an yellow solid (102.4 mg, 119%).

^{1}H NMR spectrum of the hydrogenolysis of **1f** is shown in Supplementary Fig 94.

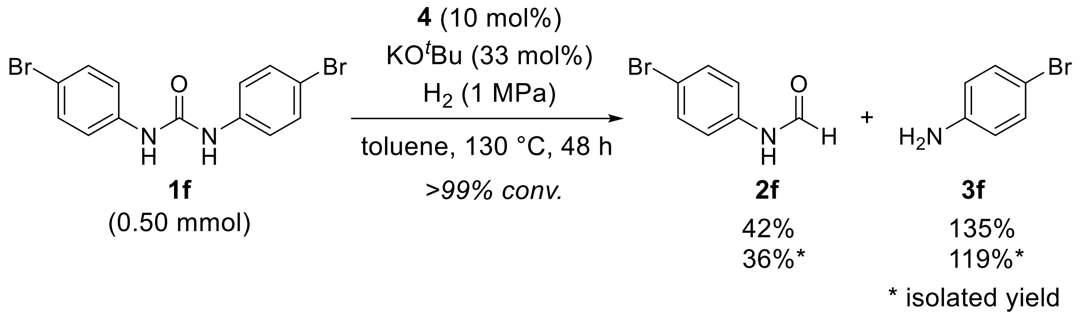

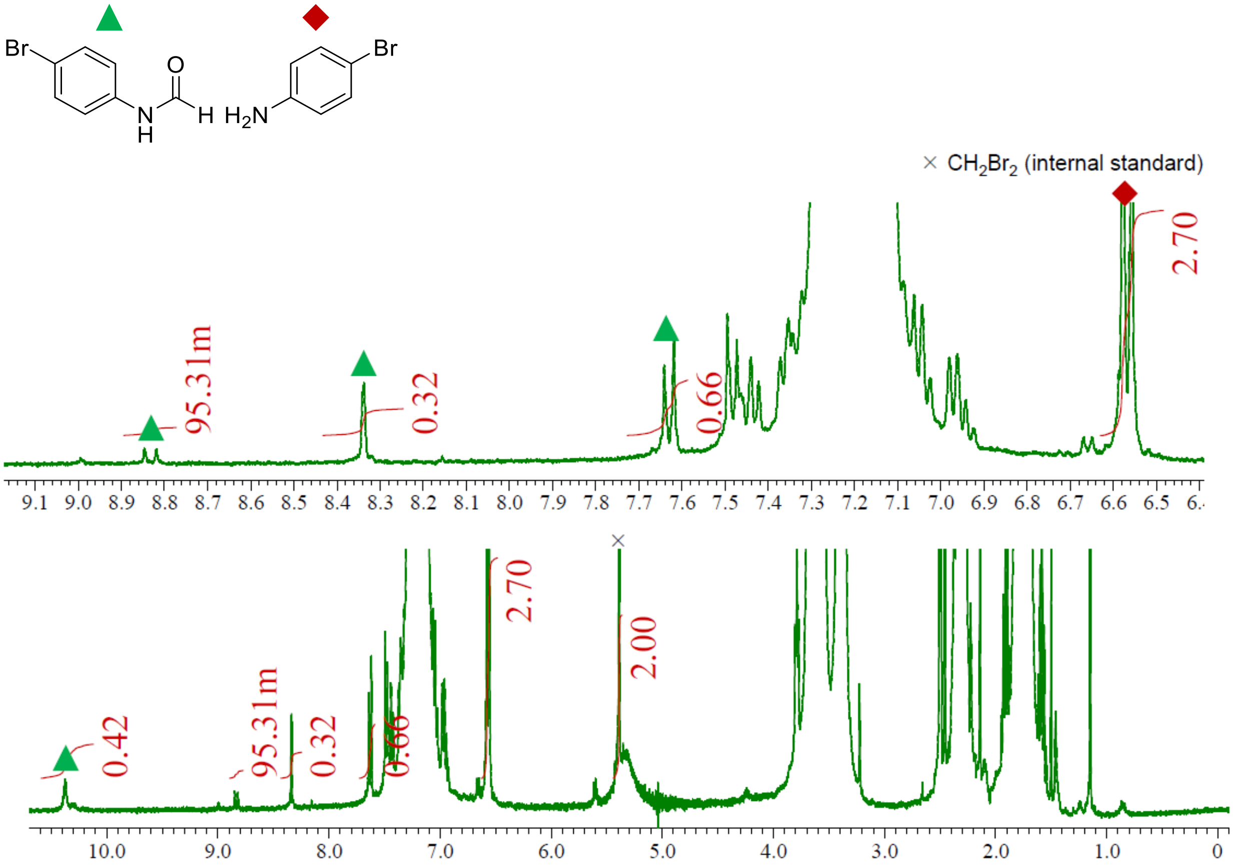

Supplementary Fig 94.

^{1}H NMR spectrum (400 MHz, DMSO-d_6/THF/toluene) of the hydrogenolysis of 1,3-bis(4-bromophenyl)urea (**1f**).

_N_-(4-Bromophenyl)formamide (2f)

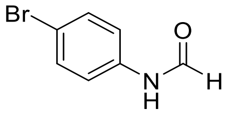

^{1}H NMR (500 MHz, $CDCl_3$): δ 8.66 (d, J = 11.5 Hz, 0.4H), 8.38 (s, 0.6H), 8.14 (br, 0.4H), 7.49–7.45 (m, 3.2H), 7.34 (br, 0.6H), 6.98 (d, J = 8.5 Hz, 0.8H); ^{13}C NMR (126 MHz, $CDCl_3$): δ 162.3, 159.0, 136.0, 135.9, 133.0, 132.3, 121.6, 120.5, 118.4, 117.6.
Two tautomers were observed in approximately 2:3 ratio. These data were consistent with those in literature[67].

4-Bromoaniline (3f)

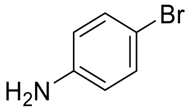

^{1}H NMR (500 MHz, $CDCl_3$): δ 7.23 (d, J = 9.0 Hz, 2H), 6.56 (d, J = 9.0 Hz, 2H), 3.63 (br, 2H); ^{13}C NMR (126 MHz, $CDCl_3$): δ 145.5, 132.1, 116.8, 110.4.
These data were consistent with those in literature[68].

8-8. The hydrogenolysis of 1,3-bis(4-*tert*-butylphenyl)urea (**1g**)

The general procedure for Condition A was followed using 1,3-bis(4-*tert*-butylphenyl)urea (**1g**) (162.2 mg, 0.50 mmol) and **4** (9.4 mg, 15 µmol) in THF (3 mL) at 130 °C under H_2 (1 MPa) for 48 h. Conversion and yields were determined by ^{1}H NMR analysis by comparing with authentic samples. Hydrochloride of 4-*tert*-butylaniline (**3g•HCl**) was isolated as an yellow solid (66.9 mg, 72%). *N*-(4-*tert*-Butylphenyl)formamide (**2g**) was isolated by silica gel column chromatography (CH_2Cl_2/ethyl acetate) as a brown solid (62.5 mg, 71%).

^{1}H NMR spectrum of the hydrogenolysis of **1g** is shown in Supplementary Fig 95.

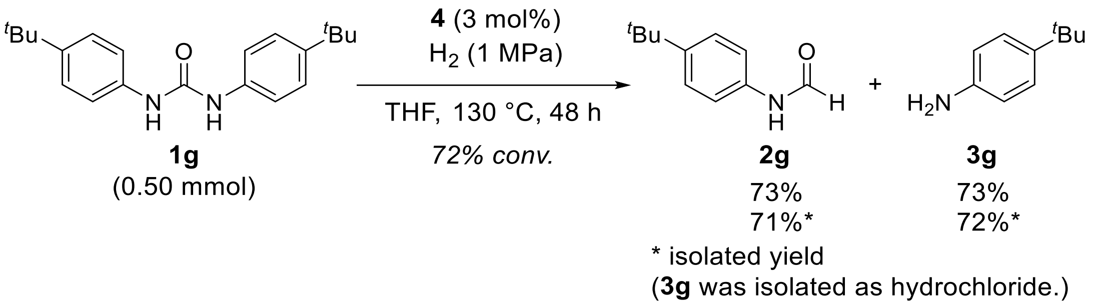

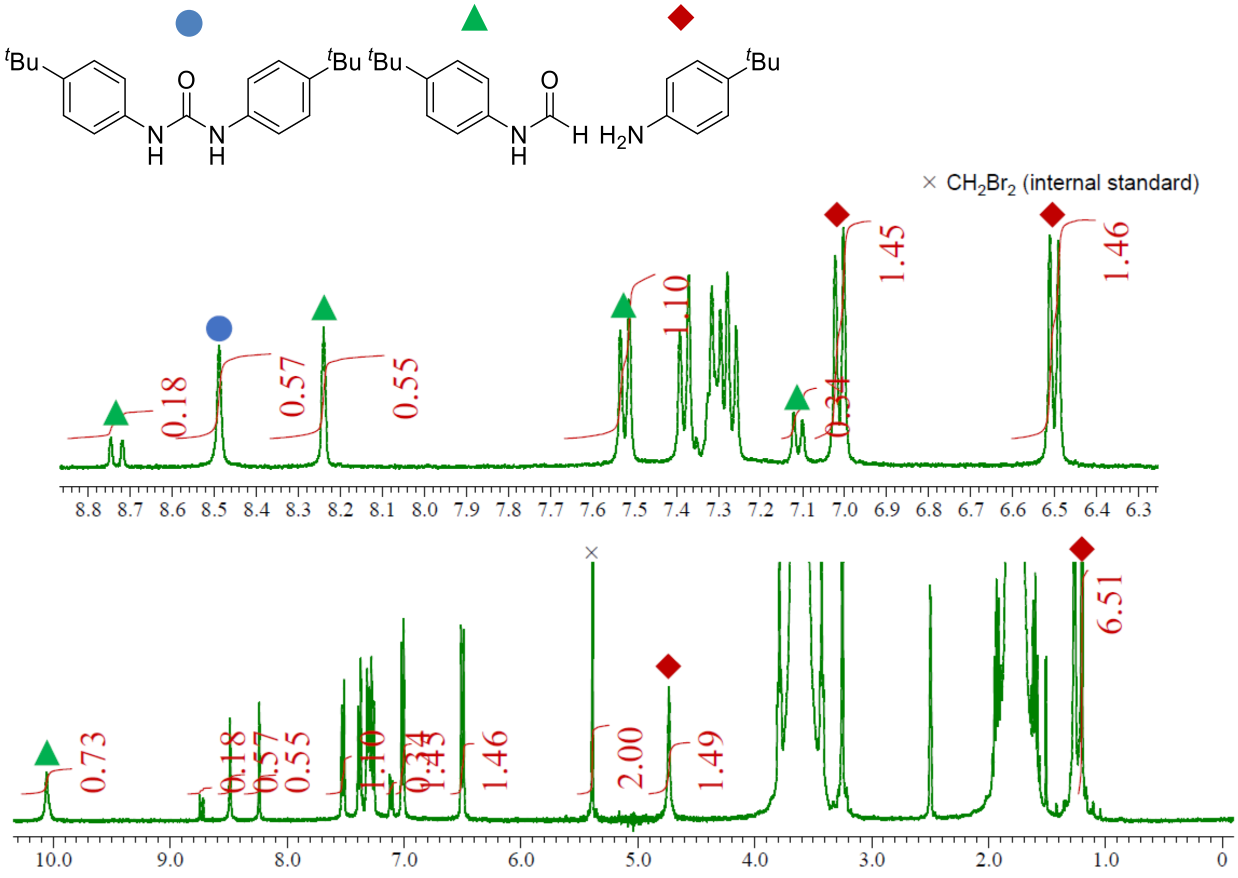

Supplementary Fig 95.

^{1}H NMR spectrum (400 MHz, DMSO-d_6/THF) of the hydrogenolysis of 1,3-bis(4-*tert*-butylphenyl)urea (**1g**).

N-(4-tert-Butylphenyl)formamide (2g)

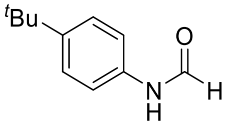

^{1}H NMR (500 MHz, $CDCl_3$): δ 8.65 (d, J = 12.0 Hz, 0.5H), 8.36 (s, 0.5H), 8.15 (br, 0.5H), 7.48–7.45 (m, 1H), 7.40 (br, 0.5H), 7.39–7.34 (m, 2H), 7.05–7.02 (m, 1H), 1.32 (s, 4.5H), 1.30 (s, 4.5H); ^{13}C NMR (126 MHz, $CDCl_3$): δ 162.6, 158.9, 148.8, 148.1, 134.3, 134.0, 126.8, 126.1, 119.9, 119.1, 34.6, 34.6, 31.5, 31.5.

Two tautomers were observed in approximately 1:1 ratio. These data were consistent with those in literature[69].

4-tert-Butylaniline hydrochloride (3g•HCl)

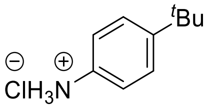

^{1}H NMR (500 MHz, $CDCl_3$): δ 10.48 (br, 3H), 7.48–7.41 (m, 4H), 1.29 (s, 9H); ^{13}C NMR (126 MHz, $CDCl_3$): δ 152.5, 127.1, 127.0, 123.1, 34.9, 31.4; MS (EI) *m/z* (relative intensity, %): 149 ([M (**3g**)]$^+$, 23), 135 (12), 134 (92), 106 (24), 94 (15), 32 (100).

Mass spectrum derived from 4-*tert*-butylaniline was observed under the basic condition using 0.5 M NaOH aq.

8-9. The hydrogenolysis of 1,3-bis(4-methoxyphenyl)urea (**1h**)

The general procedure for Condition A was followed using 1,3-bis(4-methoxyphenyl)urea (**1h**) (136.2 mg, 0.50 mmol) and **4** (9.4 mg, 15 µmol) in THF (3 mL) at 130 °C under H_2 (1 MPa) for 144 h. Conversion and yields were determined by ^{1}H NMR analysis by comparing with authentic samples. Hydrochloride of 4-methoxyaniline (**3h•HCl**) was isolated as a reddish brown solid (57.0 mg, 71%). *N*-(4-Methoxyphenyl)formamide (**2h**) was isolated by silica gel column chromatography (CH_2Cl_2/ethyl acetate) as a white solid (31.0 mg, 41%).

^{1}H NMR spectrum of the hydrogenolysis of **1h** is shown in Supplementary Fig 96.

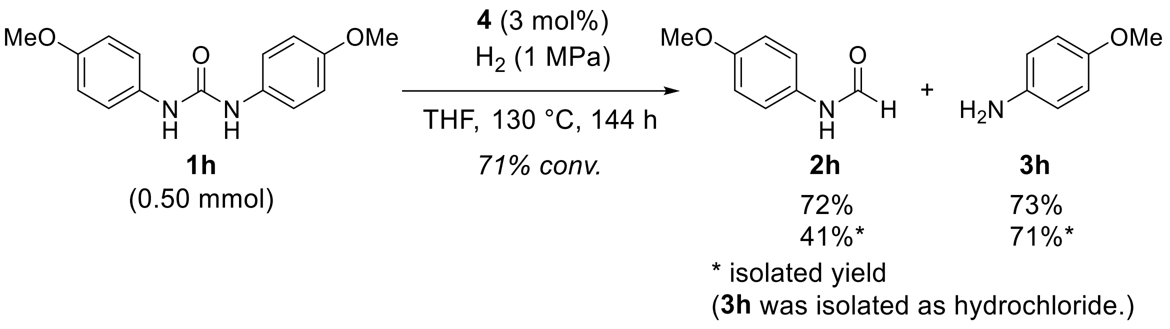

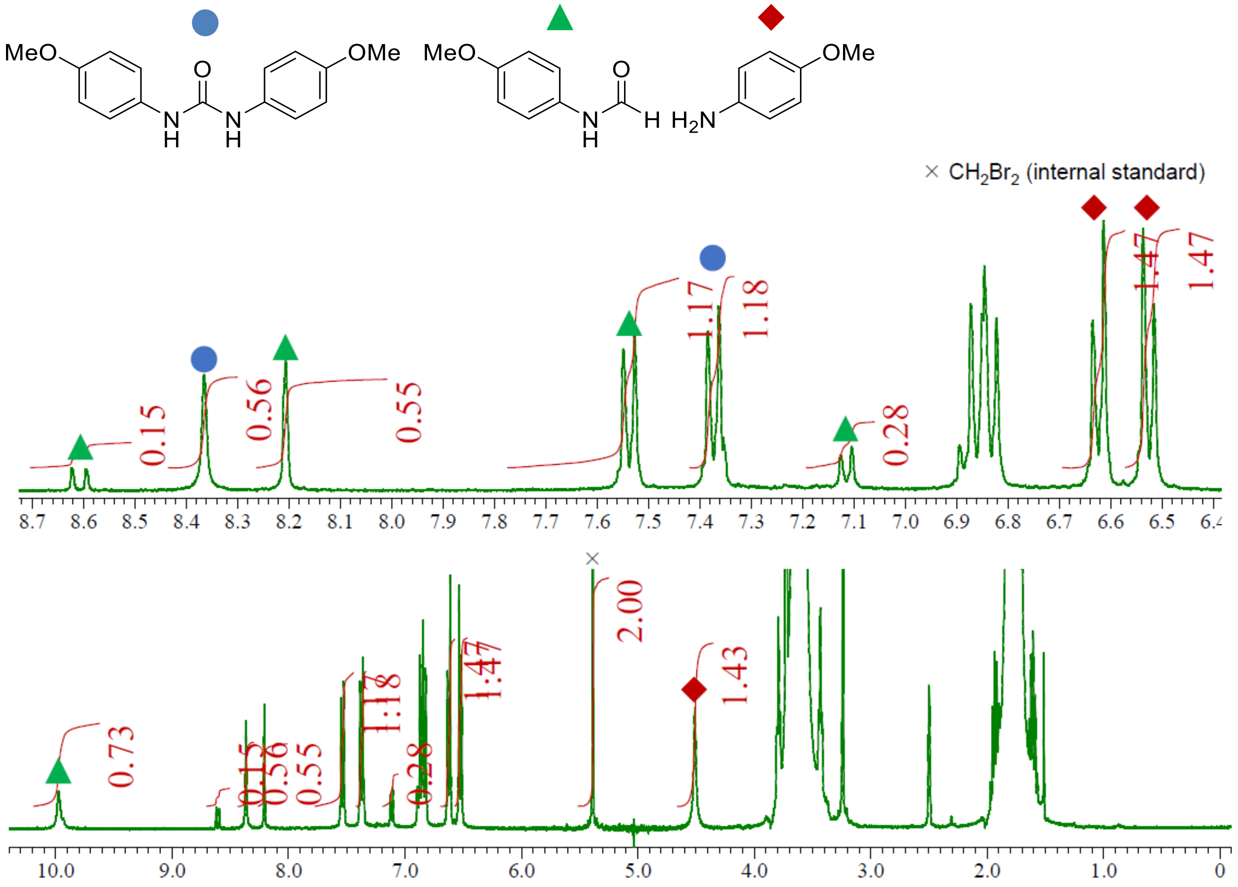

Supplementary Fig 96.

^{1}H NMR spectrum (400 MHz, DMSO-d_6/THF) of the hydrogenolysis of 1,3-bis(4-methoxyphenyl)urea (**1h**).

N-(4-Methoxyphenyl)formamide (2h)

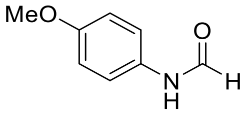

^{1}H NMR (500 MHz, $CDCl_3$): δ 8.51 (d, J = 11.5 Hz, 0.5H), 8.43 (br, 0.5H), 8.29 (s, 0.5H), 7.70 (br, 0.5H), 7.46–7.42 (m, 1H), 7.05–7.02 (m, 1H), 6.89–6.83 (m, 2H), 3.79 (s, 1.5H), 3.77 (s, 1.5H); ^{13}C NMR (126 MHz, $CDCl_3$): δ 163.4, 159,2, 157.7, 156.8, 130.1, 129.7, 121.9, 121.6, 115.0, 114.3, 55.6, 55.6. Two tautomers were observed in approximately 1:1 ratio. These data were consistent with those in literature[62].

4-Methoxyaniline hydrochloride (3h•HCl)

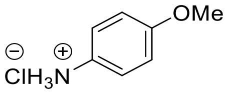

^{1}H NMR (500 MHz, DMSO-d_6): δ 10.31 (br, 3H), 7.33 (d, J = 9.0 Hz, 2H), 7.03 (d, J = 9.0 Hz, 2H), 3.76 (s, 3H); ^{13}C NMR (126 MHz, DMSO-d_6): δ 158.7, 124.5, 124.2, 114.8, 55.5.
These data were consistent with those in literature[61].

8-10. The hydrogenolysis of 1,3-bis(4-dimethylaminophenyl)urea (**1i**)

The general procedure for Condition B was followed using 1,3-bis(4-dimethylaminophenyl)urea (**1i**) (149.2 mg, 0.50 mmol), **4** (9.4 mg, 15 µmol), and KO^{t}Bu (5.6 mg, 0.05 mmol) in toluene (3 mL) at 130 °C under H_2 (1 MPa) for 48 h. Conversion and yields were determined by ^{1}H NMR analysis by comparing with authentic samples. The reaction mixture was concentrated and purified by silica gel column chromatography (CH_2Cl_2/ethyl acetate) to obtain *N*-(4-dimethylaminophenyl)formamide (**2i**) as a black solid (71.0 mg, 86%) and 4-dimethylaminoaniline (**3i**) as a purple solid (55.6 mg, 82%).

^{1}H NMR spectrum of the hydrogenolysis of **1i** is shown in Supplementary Fig 97.

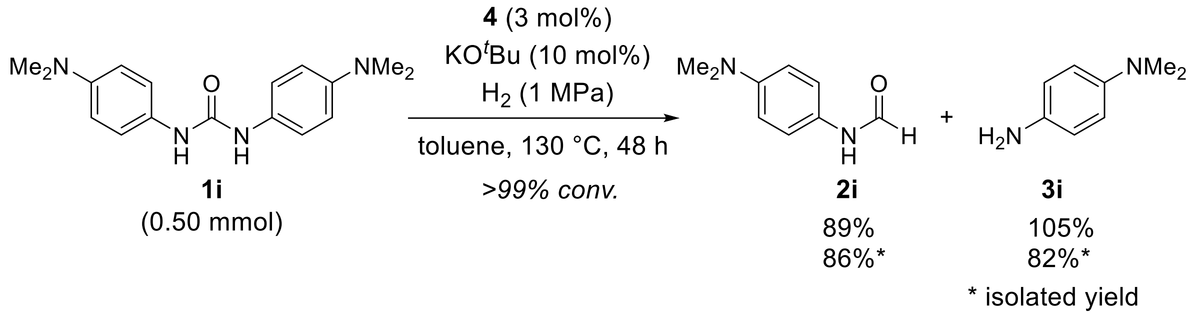

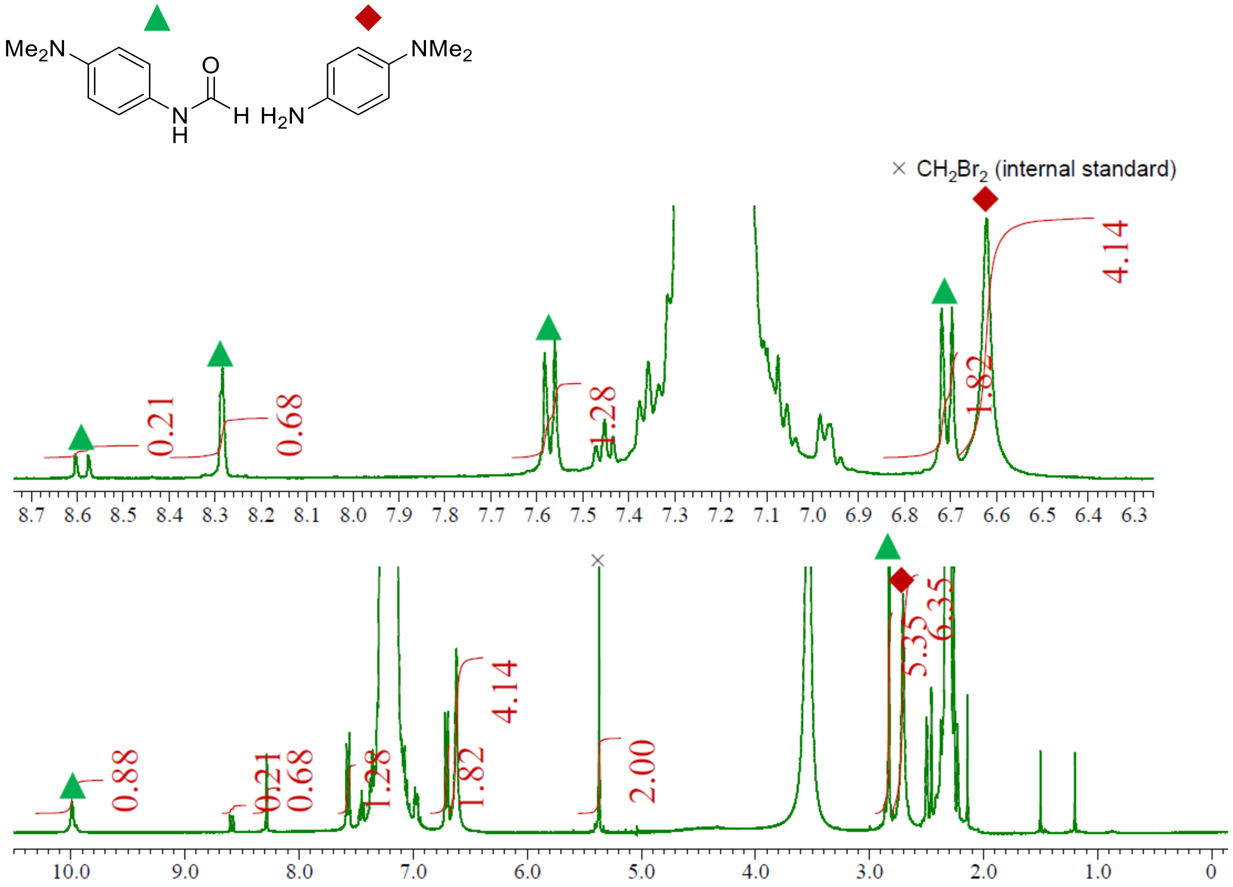

Supplementary Fig 97.

^{1}H NMR spectrum (400 MHz, DMSO-d_6/toluene) of the hydrogenolysis of 1,3-bis(4-dimethylaminophenyl)urea (**1i**).

N-(4-Dimethylaminophenyl)formamide (2i)

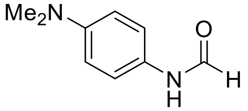

^{1}H NMR (500 MHz, $CDCl_3$): δ 8.44 (d, *J* = 11.5 Hz, 0.5H), 8.27 (d, *J* = 2.0 Hz, 0.5H), 7.98 (br, 0.5H), 7.49 (br, 0.5H), 7.40–7.37 (m, 1H), 7.00–6.97 (m, 1H), 6.71–6.68 (m, 2H), 2.93 (s, 3H), 2.91 (s, 3H); ^{13}C NMR (126 MHz, $CDCl_3$): δ 163.4, 159.0, 149.1, 148.2, 126.9, 125.9, 122.2, 121.9, 113.4, 113.2, 41.0, 40.9.
Two tautomers were observed in approximately 1:1 ratio. These data were consistent with those in literature[67].

4-Dimethylaminoaniline (3i)

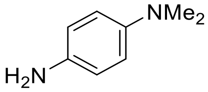

^{1}H NMR (500 MHz, $CDCl_3$): δ 6.71–6.65 (m, 4H), 3.31 (br, 2H), 2.82 (s, 6H); ^{13}C NMR (126 MHz, $CDCl_3$): δ 144.9, 138.0, 116.7, 115.7, 42.2.
These data were consistent with those in literature[70].

8-11. The hydrogenolysis of diethyl 4,4’-(carbonylbis(azanediyl))dibenzoate (**1j**)

The general procedure for Condition A was followed using diethyl 4,4’-(carbonylbis(azanediyl))dibenzoate (**1j**) (178.2 mg, 0.50 mmol) and **4** (9.4 mg, 15 µmol) in THF (3 mL) at 130 °C under H_2 (1 MPa) for 144 h. Conversion and yields were determined by ^{1}H NMR analysis by comparing with authentic samples. The reaction mixture was concentrated and purified by silica gel column chromatography (CH_2Cl_2/ethyl acetate) to obtain ethyl 4-formamidobenzoate (**2j**) as an yellow solid (57.5 mg, 60%) and ethyl 4-aminobenzoate (**3j**) as a white solid (90.5 mg, 110%).

^{1}H NMR spectrum of the hydrogenolysis of **1j** is shown in Supplementary Fig 98.

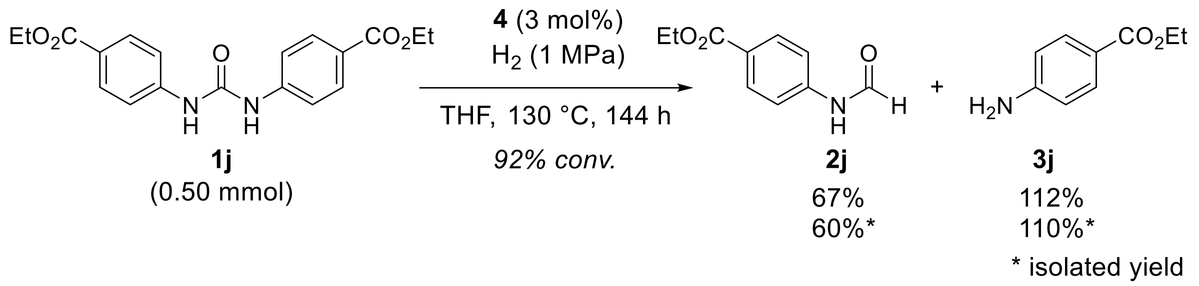

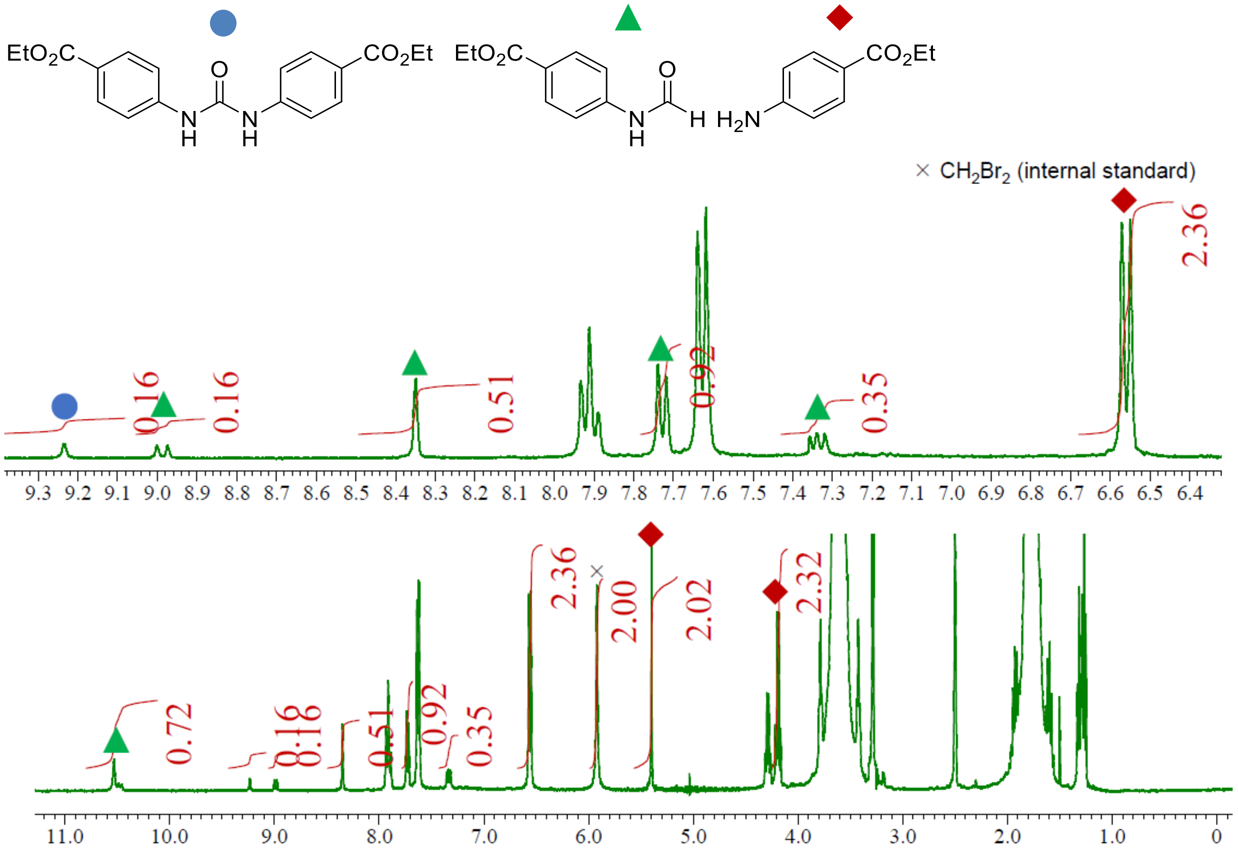

Supplementary Fig 98.

^{1}H NMR spectrum (400 MHz, DMSO-d_6/THF) of the hydrogenolysis of diethyl 4,4’-(carbonylbis(azanediyl))dibenzoate (**1j**).

Ethyl 4-formamidobenzoate (2j)

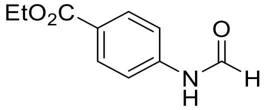

^{1}H NMR (500 MHz, $CDCl_3$): δ 8.85 (d, *J* = 11.0 Hz, 0.44H), 8.56 (d, *J* = 11.0 Hz, 0.44H), 8.43 (d, *J* = 1.5 Hz, 0.56H), 8.03 (dd, *J* = 12.0, 9.0 Hz, 2H), 7.78 (br, 0.56H), 7.63 (d, *J* = 9.0 Hz, 1H), 7.14 (d, *J* = 8.5 Hz, 1H), 4.37 (q, *J* = 7.0 Hz, 0.89H), 4.36 (q, *J* = 7.0 Hz, 1.11H), 1.39 (t, *J* = 7.0 Hz, 1.33H), 1.38 (t, *J* = 7.0 Hz, 1.67H); ^{13}C NMR (126 MHz, $CDCl_3$): δ 166.2, 166.0, 162.1, 159.2, 141.1, 141.0, 131.6, 131.0, 127.1, 126.6, 119.2, 117.3, 61.3, 61.1, 14.5.

Two tautomers were observed in approximately 4:5 ratio. These data were consistent with those in literature[62].

Ethyl 4-aminobenzoate (3j)

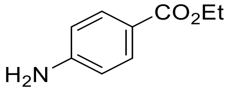

^{1}H NMR (500 MHz, $CDCl_3$): δ 7.87–7.84 (m, 2H), 6.65–6.62 (m, 2H), 4.31 (q, *J* = 7.2 Hz, 2H), 4.06 (br, 2H), 1.36 (t, *J* = 7.2 Hz, 3H); ^{13}C NMR (126 MHz, $CDCl_3$): δ 166.8, 150.8, 131.7, 120.2, 113.9, 60.4, 14.6.

These data were consistent with those in literature[71].

8-12. The hydrogenolysis of 4,4’-(carbonylbis(azanediyl))bis(*N*,*N*-dipropylbenzamide) (**1k**)

The general procedure for Condition A was followed using 4,4’-(carbonylbis(azanediyl))bis(*N*,*N*-dipropylbenzamide) (**1k**) (233.3 mg, 0.50 mmol) and **4** (9.4 mg, 15 μmol) in THF (3 mL) at 130 °C under H_2 (1 MPa) for 144 h. Conversion and yields were determined by ^{1}H NMR analysis by comparing with authentic samples. The reaction mixture was concentrated and purified by silica gel column chromatography (CH_2Cl_2/ethyl acetate) to obtain 4-formamido-*N*,*N*-dipropylbenzamide (**2k**) as an yellow oil (86.1 mg, 69%) and 4-amino-*N*,*N*-dipropylbenzamide (**3k**) as a yellow oil (83.2 mg, 76%).

^{1}H NMR spectrum of the hydrogenolysis of **1k** is shown in Supplementary Fig 99.

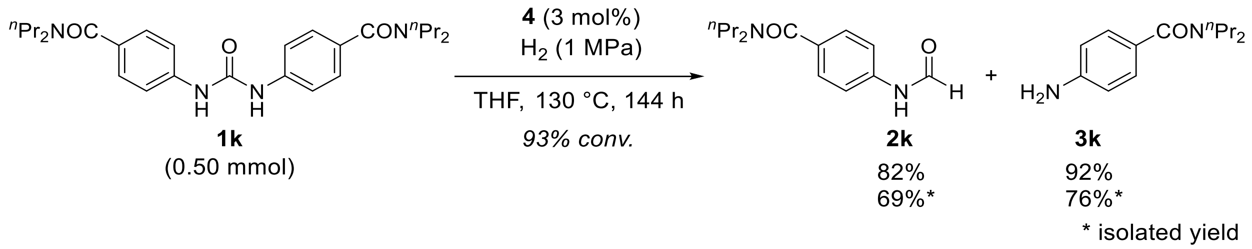

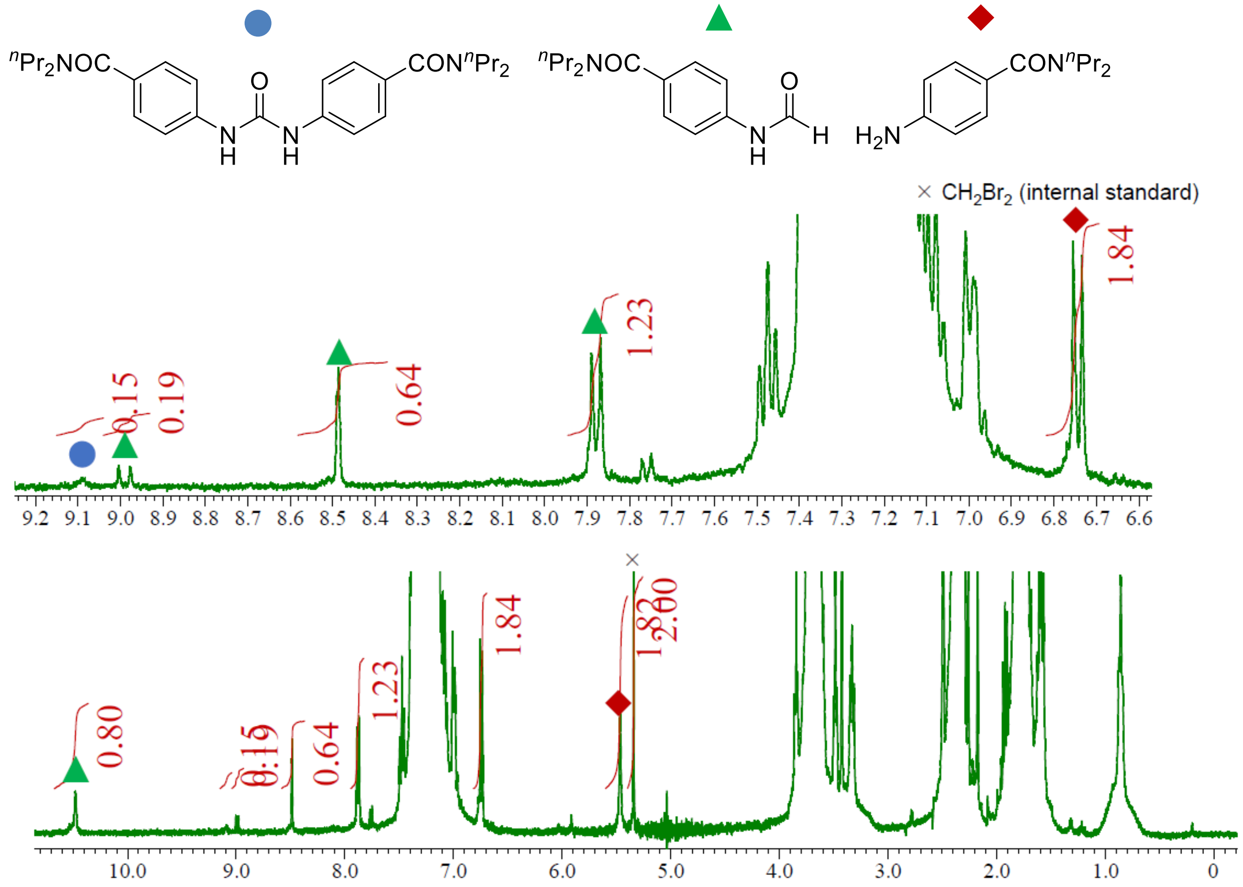

Supplementary Fig 99.

^{1}H NMR spectrum (400 MHz, DMSO-d_6/THF/toluene) of the hydrogenolysis of diethyl 4,4’-(carbonylbis(azanediyl))bis(*N*,*N*-dipropylbenzamide) (**1k**).

4-Formamido-*N*,*N*-dipropylbenzamide (2k)

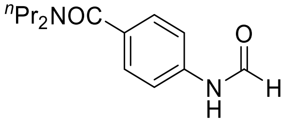

^1H NMR (500 MHz, $CDCl_3$): δ 8.72 (d, *J* = 11.0 Hz, 0.3H), 8.65 (br, 0.7H), 8.27 (d, *J* = 1.5 Hz, 0.7H), 8.22 (d, *J* = 11.5Hz, 0.3H), 7.48 (d, *J* = 8.5 Hz, 1.4H), 7.34 (d, *J* = 8.5 Hz, 0.6H), 7.24 (d, *J* = 8.5 Hz, 1.4H), 7.07 (d, *J* = 8.5 Hz, 0.6H), 3.31 (d, *J* = 133.5 Hz, 4H), 1.60 (d, *J* = 77.0 Hz, 4H), 0.85 (d, *J* = 113.5 Hz, 6H); ^{13}C NMR (126 MHz, $CDCl_3$): δ 171.8, 171.1, 162.1, 159.6, 138.3, 137.7, 134.1, 132.8, 128.4, 127.4, 120.0, 118.4, 51,0, 46.7, 22.0, 20.9, 11.6, 11.2; HRMS (ESI) *m*/*z* calcd. for $C_{14}H_{21}N_2O_2$ $[M+H]^+$ 249.1603, found 249.1605.
Two tautomers were observed in approximately 3:7 ratio.

4-Amino-*N*,*N*-dipropylbenzamide (3k)

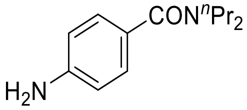

^1H NMR (500 MHz, $CDCl_3$): δ 7.19–7.17 (m, 2H), 6.64–6.62 (m, 2H), 3.63 (br, 2H), 3.31 (br, 4H), 1.58 (br, 4H), 0.84 (br, 6H); ^{13}C NMR (126 MHz, $CDCl_3$): δ 172.3, 147.5, 128.5, 127.2, 114.5, 50.9 (br), 46.7 (br), 21.4 (br), 11.4; HRMS (ESI) *m*/*z* calcd. for $C_{13}H_{20}N_2NaO$ $[M+Na]^+$ 243.1473, found 243.1480.

8-13. The hydrogenolysis of 1,3-bis(4-cyanophenyl)urea (**1*l***)

The general procedure for Condition A was followed using 1,3-bis(4-cyanophenyl)urea (**1*l***) (131.1 mg, 0.50 mmol) and **4** (9.4 mg, 15 µmol) in THF (3 mL) at 130 °C under H_2 (1 MPa) for 144 h. Conversion and yields were determined by ^{1}H NMR analysis by comparing with authentic samples. The reaction mixture was concentrated and purified by silica gel column chromatography (CH_2Cl_2/ethyl acetate) to obtain *N*-(4-cyanophenyl)formamide (**2*l***) as a white solid (44.4 mg, 61%) and 4-aminobenzonitrile (**3*l***) as an yellow solid (49.0 mg, 83%).

^{1}H NMR spectrum of the hydrogenolysis of **1*l*** is shown in Supplementary Fig 100.

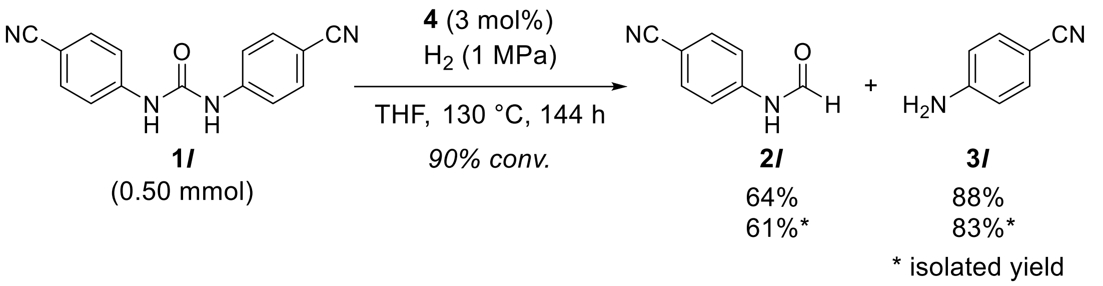

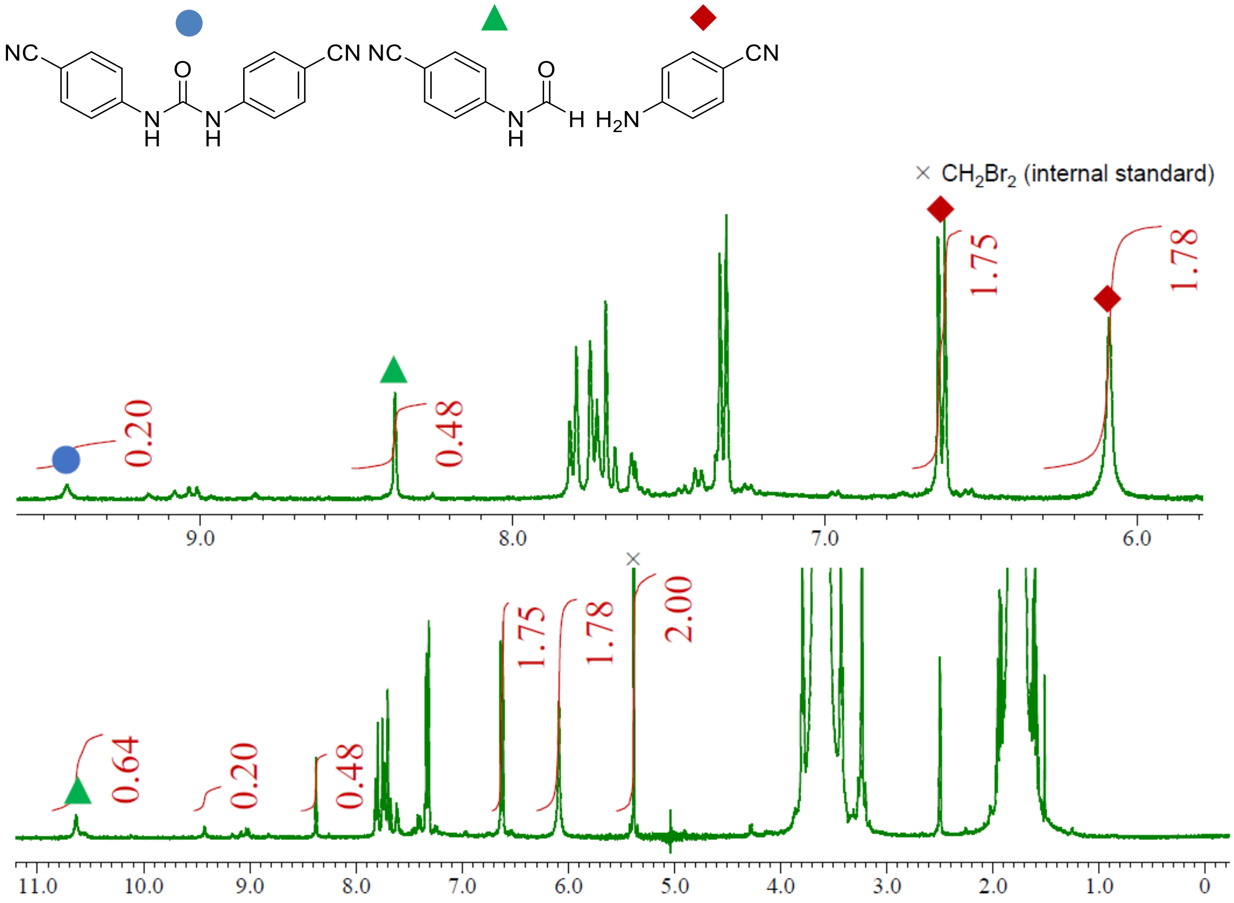

Supplementary Fig 100.

^{1}H NMR spectrum (400 MHz, DMSO-d_6/THF) of the hydrogenolysis of 1,3-bis(4-cyanophenyl)urea (**1*l***).

_N_-(4-Cyanophenyl)formamide (2*l*)

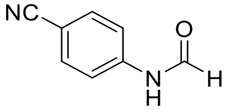

^{1}H NMR (500 MHz, DMSO-d_6): δ 10.64 (s, 0.75H), 10.53 (s, 0.25H), 8.99 (d, J = 9.5 Hz, 0.25H), 8.37 (s, 0.75H), 7.80–7.74 (m, 3.5H), 7.37 (d, J = 8.5 Hz, 0.5H); ^{13}C NMR (126 MHz, DMSO-d_6): δ 162.7, 160.4, 142.9, 142.3, 133.8, 133.4, 119.3, 118.9, 117.1, 105.4, 105.2.
Two tautomers were observed in approximately 1:3 ratio. These data were consistent with those in literature[72].

4-Aminobenzonitrile (3*l*)

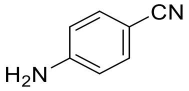

^{1}H NMR (500 MHz, $CDCl_3$): δ 7.40 (d, J = 8.5 Hz, 2H), 6.64 (d, J = 8.5 Hz, 2H), 4.17 (br, 2H); ^{13}C NMR (126 MHz, $CDCl_3$): δ 150.5, 133.9, 120.3, 114.5, 100.3.
These data were consistent with those in literature[68].

8-14. The hydrogenolysis of 1,3-dibenzylurea (**1m**)

The general procedure for Condition B was followed using 1,3-dibenzylurea (**1m**) (120.2 mg, 0.50 mmol), **4** (9.4 mg, 15 μmol), and KO^{t}Bu (5.6 mg, 0.05 mmol) in toluene (3 mL) at 130 °C under H_2 (1 MPa) for 48 h. Conversion and yields were determined by ^{1}H NMR analysis by comparing with authentic samples. Hydrochloride of benzylamine (**3m•HCl**) was isolated as a white solid (6 mg, 8%). *N*-benzylformamide (**2m**) was isolated by silica gel column chromatography (CH_2Cl_2/ethyl acetate) as a white solid (11 mg, 16%).

^{1}H NMR spectrum of the hydrogenolysis of **1m** is shown in Supplementary Fig 101.

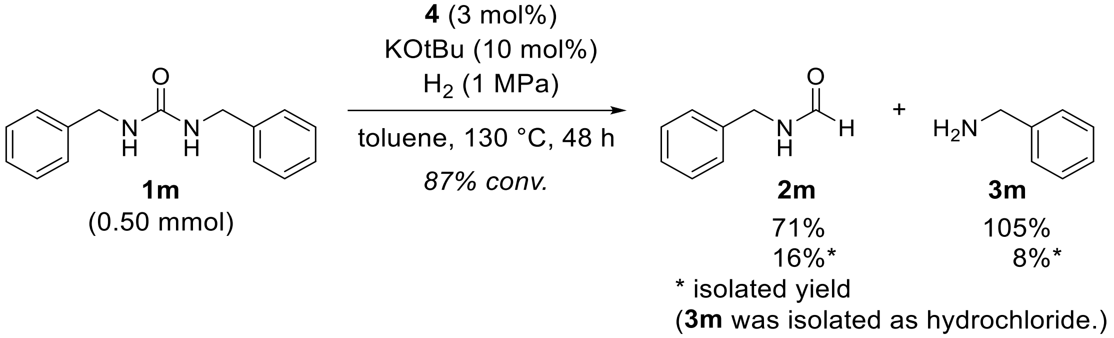

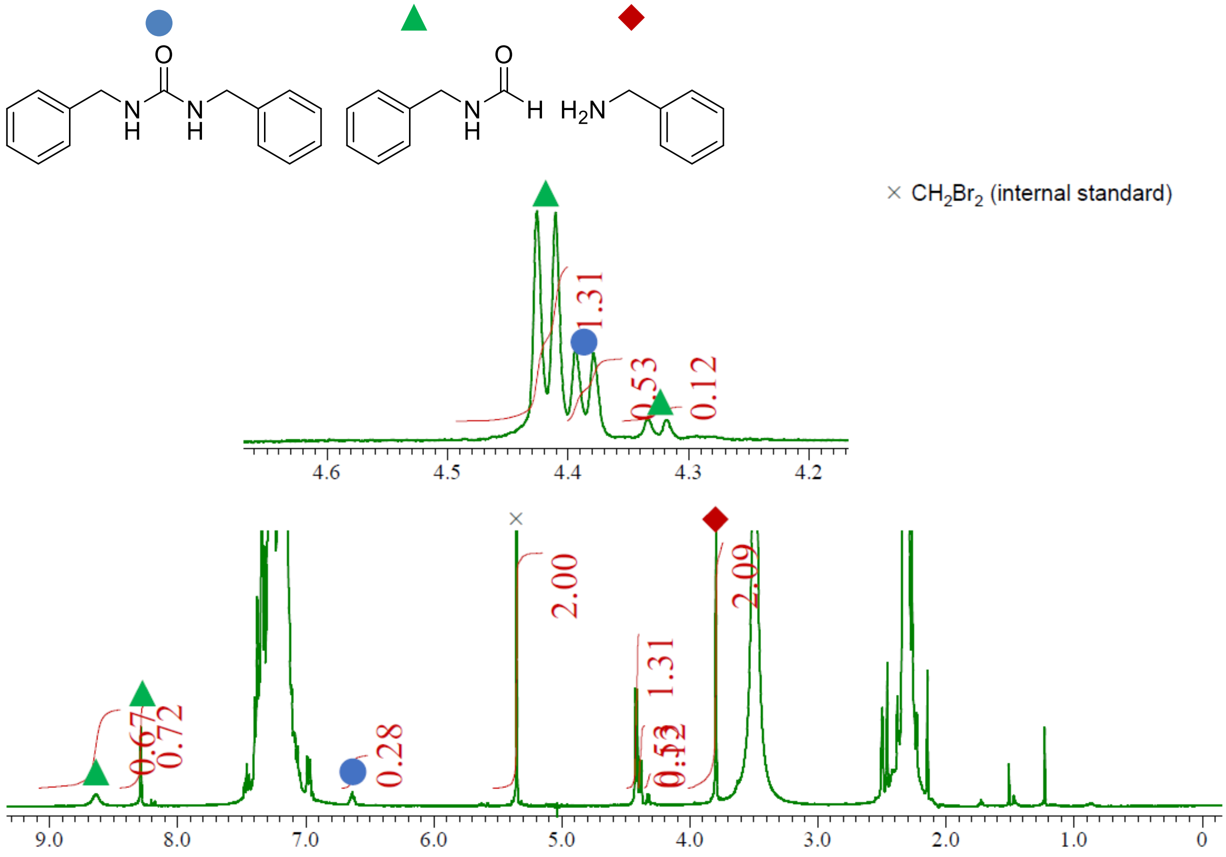

Supplementary Fig 101.
^{1}H NMR spectrum (400 MHz, DMSO-d_6/toluene) of the hydrogenolysis of 1,3-dibenzylurea (**1m**).

N-Benzylformamide (2m)

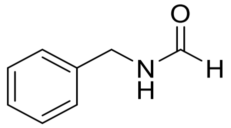

^{1}H NMR (500 MHz, $CDCl_3$): δ 8.24 (s, 0.85H), 8.16 (d, *J* = 13.5 Hz, 0.15H), 7.38–7.24 (m, 5H), 5.99 (br, 1H), 4.47 (d, *J* = 5.5 Hz, 1.7H), 4.40 (d, *J* = 6.5 Hz, 0.3H); ^{13}C NMR (126 MHz, DMSO-d_6): δ 164.7, 161.1, 137.7, 137.6, 129.0, 128.9, 128.1, 127.9, 127.8, 127.1, 45.8, 42.3.
Two tautomers were observed in approximately 3:17 ratio. These data were consistent with those in literature[59].

Benzylamine hydrochloride (3m•HCl)

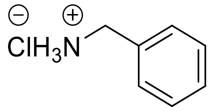

^{1}H NMR (500 MHz, DMSO-d_6): δ 8.60 (br, 3H), 7.51 (d, *J* = 6.5 Hz, 2H), 7.42–7.35 (m, 3H), 3.99 (s, 2H); ^{13}C NMR (126 MHz, DMSO-d_6): δ 134.1, 129.0, 128.5, 128.3, 42.1.
These data were consistent with those in literature[73].

8-15. The hydrogenolysis of 1,3-dicyclohexylurea (**1n**)

The general procedure for Condition B was followed using 1,3-dicyclohexylurea (**1n**) (112.2 mg, 0.50 mmol), **4** (9.4 mg, 15 µmol), and KO*^t^*Bu (5.6 mg, 0.05 mmol) in toluene (3 mL) at 130 °C under H_2 (1 MPa) for 48 h. The conversion was not determined due to the low solubility of 1,3-dicyclohexylurea in DMSO-d_6. The yield of *N*-cyclohexylformamide (**2n**) was determined by ^{1}H NMR spectroscopy using dibromomethane as the internal standard, and the yield of cyclohexylamine (**3n**) was determined by GC using decane as the internal standard.

^{1}H NMR spectrum and GC chart of the hydrogenolysis of **1n** are shown in Supplementary Figs 102 and 103, respectively.

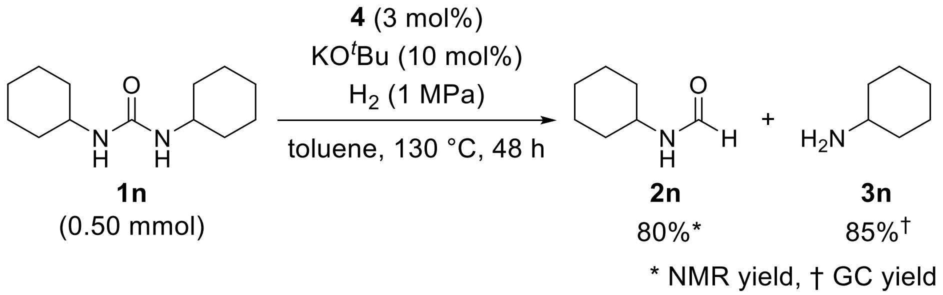

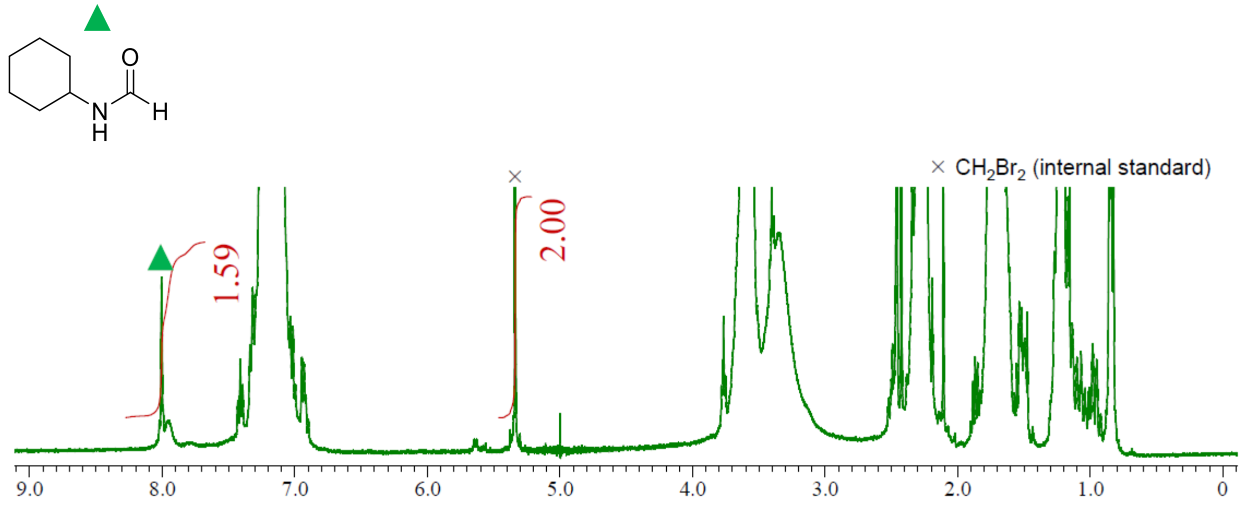

Supplementary Fig 102.

^{1}H NMR spectrum (400 MHz, DMSO-d_6/THF/toluene) of the hydrogenolysis of 1,3-dicyclohexylurea (**1n**).

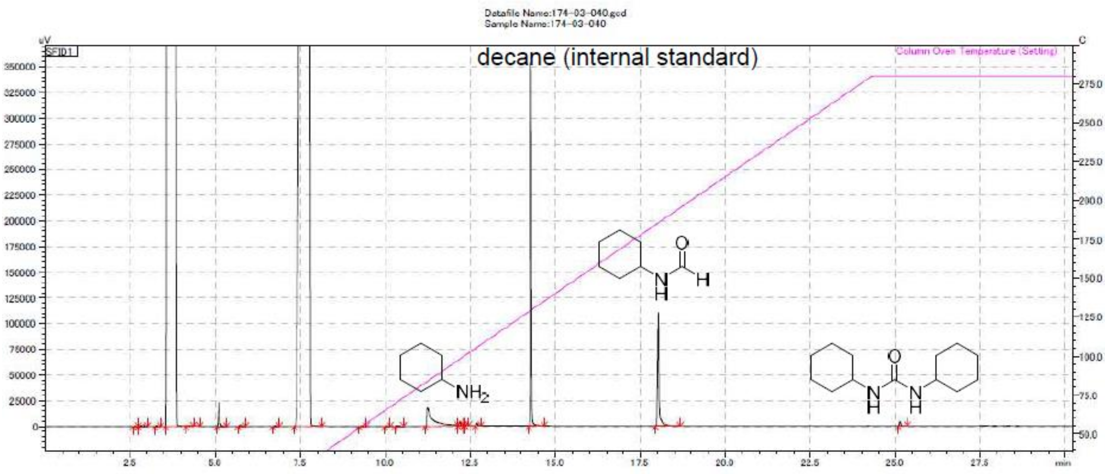

Supplementary Fig 103.

GC chart of the hydrogenolysis of 1,3-dicyclohexylurea (**1n**).

8-16. The hydrogenolysis of 1,3-diphenyl-1-methylurea (**1ao**) (Condition A)

The general procedure for Condition A was followed using 1,3-diphenyl-1-methylurea (**1ao**) (113.1 mg, 0.50 mmol) and **4** (9.4 mg, 15 μmol) in THF (3 mL) at 130 °C under H_2 (1 MPa) for 48 h. Conversion and yields were determined by ^{1}H NMR analysis by comparing with authentic samples. The mixture of *N*-methylbenzenaminium chloride (**3o•HCl**) and aniline hydrochloride (**3a•HCl**) was obtained as a green solid (63.9 mg, 94:6). Formanilide (**2a**) was isolated by silica gel column chromatography (CH_2Cl_2/ethyl acetate) as an yellow solid (39.0 mg, 64%).

^{1}H NMR spectrum of the hydrogenolysis of **1ao** is shown in Supplementary Fig 104.

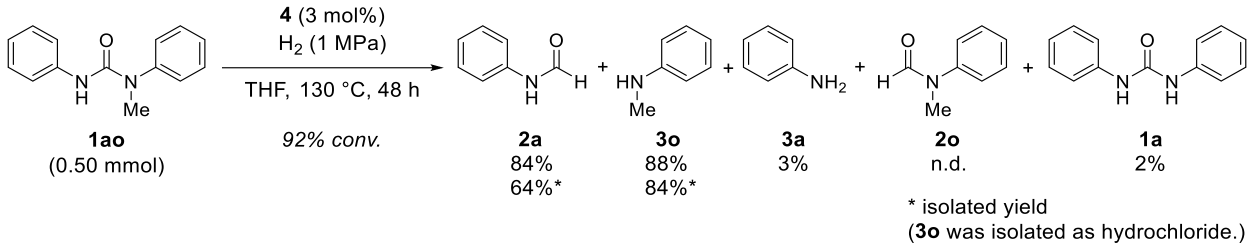

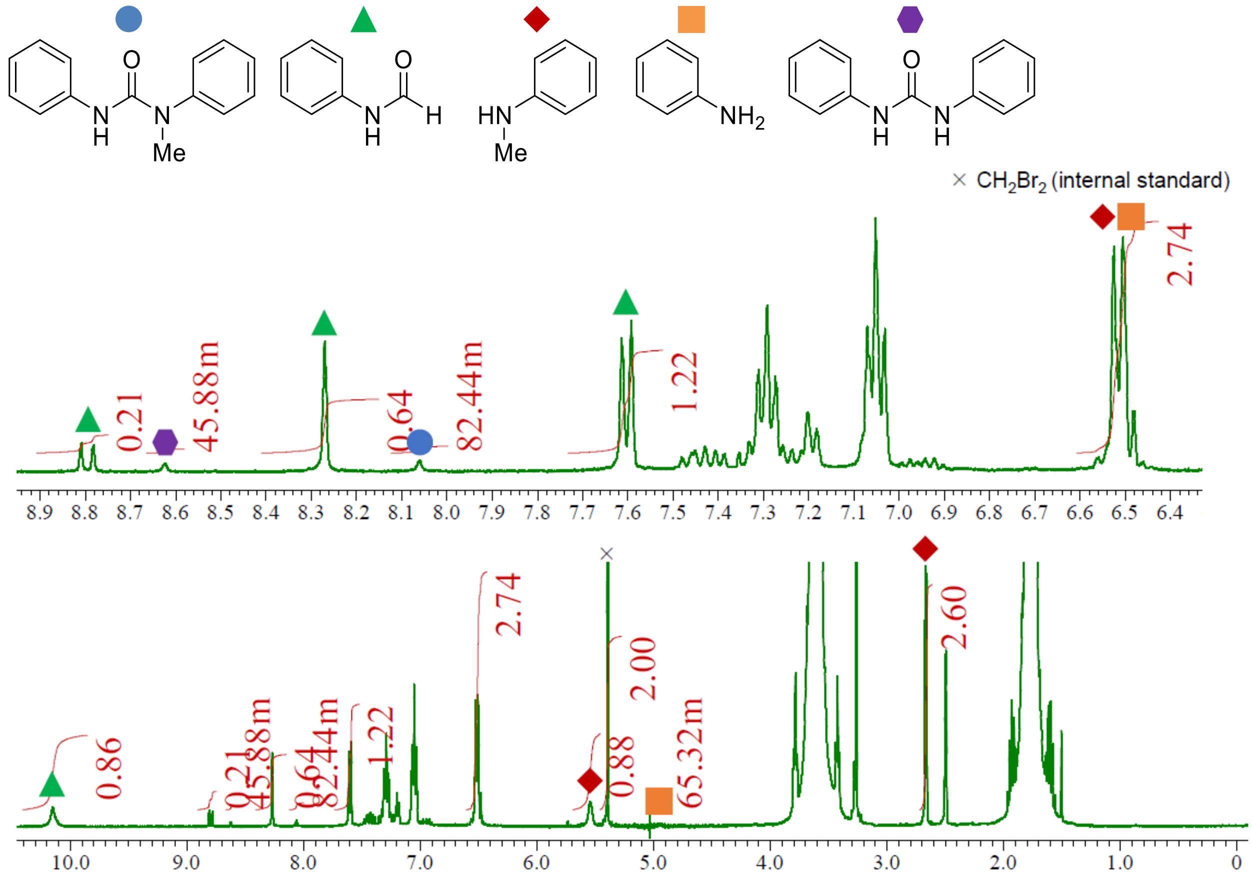

Supplementary Fig 104.

^{1}H NMR spectrum (400 MHz, DMSO-d_6/THF) of the hydrogenolysis of 1,3-diphenyl-1-methylurea (**1ao**) (Condition A).

Formanilide (2a)

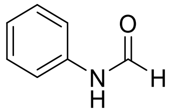

^{1}H NMR (500 MHz, $CDCl_3$): δ 8.70 (d, *J* = 11.5 Hz, 0.5H), 8.53 (br, 0.5H), 8.37 (s, 0.5H), 7.59 (br, 0.5H), 7.54 (d, *J* = 7.5 Hz, 1H), 7.37–7.31 (m, 2H), 7.21–7.09 (m, 2H); ^{13}C NMR (126 MHz, $CDCl_3$): δ 162.9, 159.2, 137.0, 136.8, 129.9, 129.2, 125.4, 124.9, 120.1, 118.9.
Two tautomers were observed in approximately 1:1 ratio. These data were consistent with those in literature[59,60].

***N*-Methylbenzenaminium chloride (3o•HCl)**

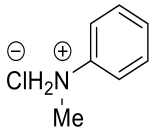

^{1}H NMR (500 MHz, $CDCl_3$): δ 11.36 (br, 2H), 7.62 (d, *J* = 6.5 Hz, 2H), 7.43–7.37 (m, 3H), 3.00 (s, 3H); ^{13}C NMR (126 MHz, $CDCl_3$): δ 137.1, 130.3, 129.6, 122.7, 38.1.
These data were consistent with those in literature[74].

8-17. The hydrogenolysis of 1,3-diphenyl-1-methylurea (**1ao**) (Condition B)

The general procedure for Condition B was followed using 1,3-diphenyl-1-methylurea (**1ao**) (113.1 mg, 0.50 mmol), **4** (9.4 mg, 15 µmol), and KO^{t}Bu (5.6 mg, 0.050 mmol) in toluene (3 mL) at 130 °C under H_2 (1 MPa) for 48 h. Conversion and yields were determined by ^{1}H NMR analysis by comparing with authentic samples.

^{1}H NMR spectrum of the hydrogenolysis of **1ao** is shown in Supplementary Fig 105.

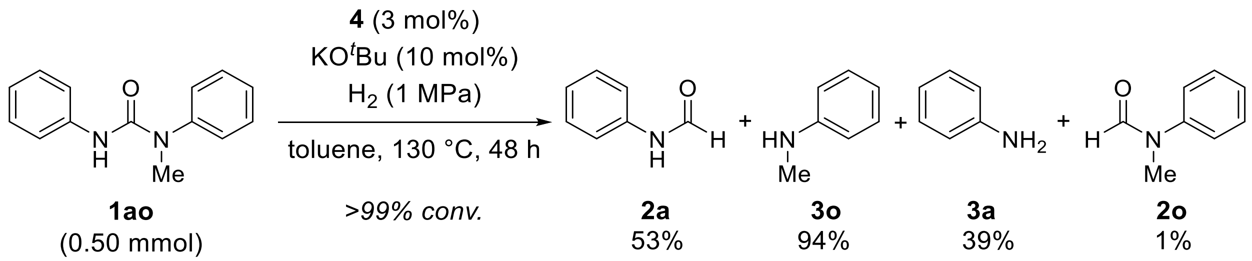

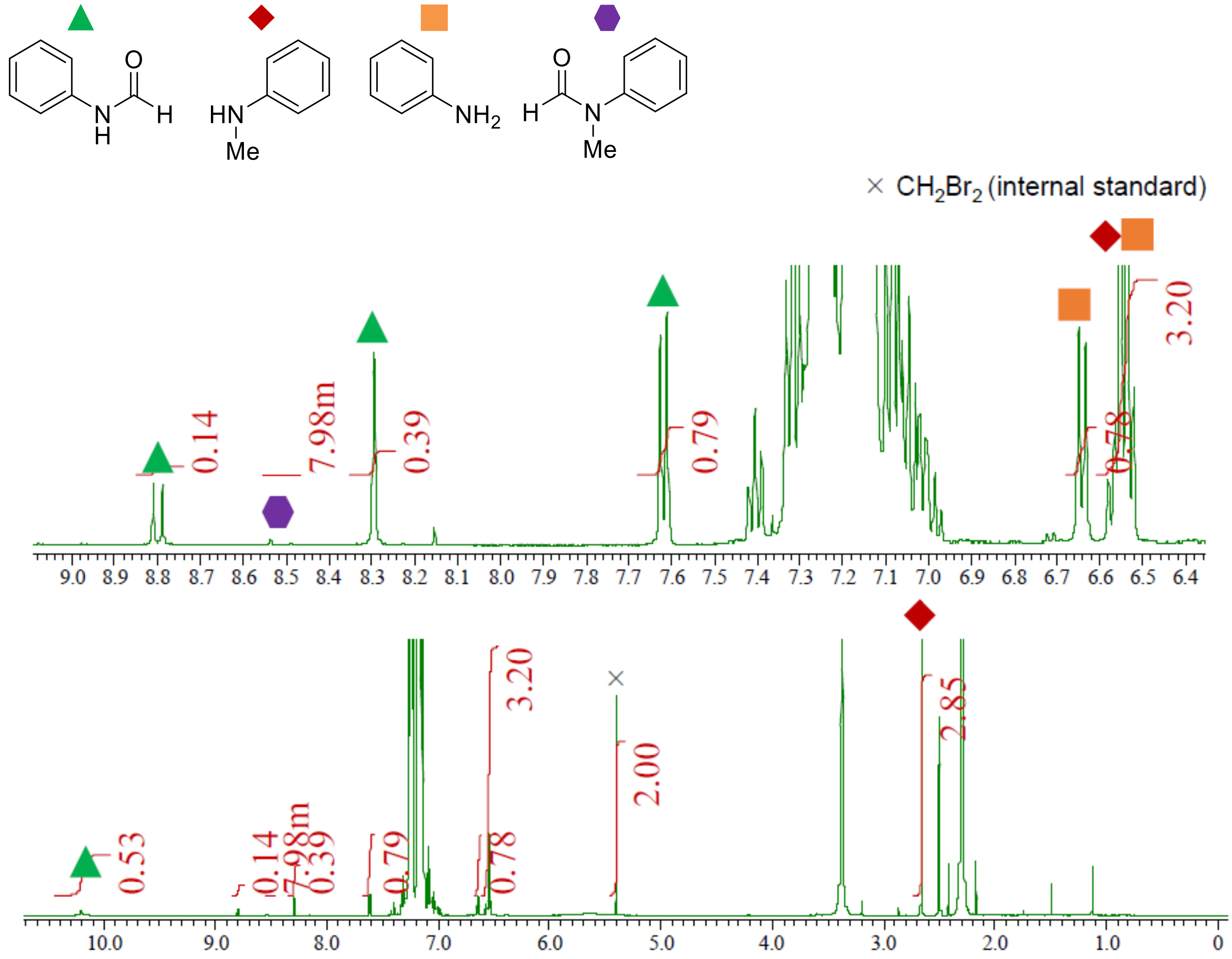

Supplementary Fig 105.

^{1}H NMR spectrum (500 MHz, DMSO-d_6/toluene) of the hydrogenolysis of 1,3-diphenyl-1-methylurea (**1ao**) (Condition B).

8-18. The hydrogenolysis of 1-(4-chlorophenyl)-1-methyl-3-phenylurea (**1ap**)

The general procedure for Condition A was followed using 1-(4-chlorophenyl)-1-methyl-3-phenylurea (**1ap**) (130.4 mg, 0.50 mmol) and **4** (9.4 mg, 15 µmol) in THF (3 mL) at 130 °C under H_2 (1 MPa) for 48 h. Conversion and yields were determined by ^{1}H NMR analysis by comparing with authentic samples.

^{1}H NMR spectrum of the hydrogenolysis of **1ap** is shown in Supplementary Fig 106.

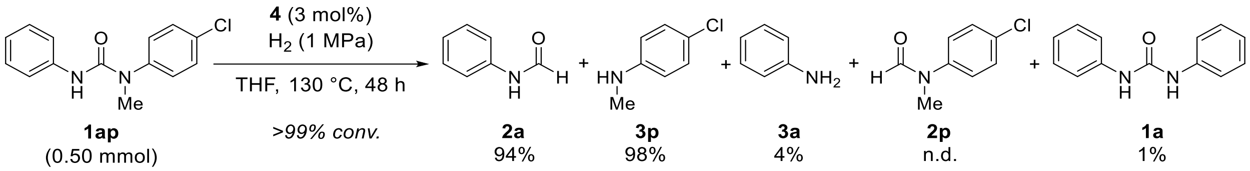

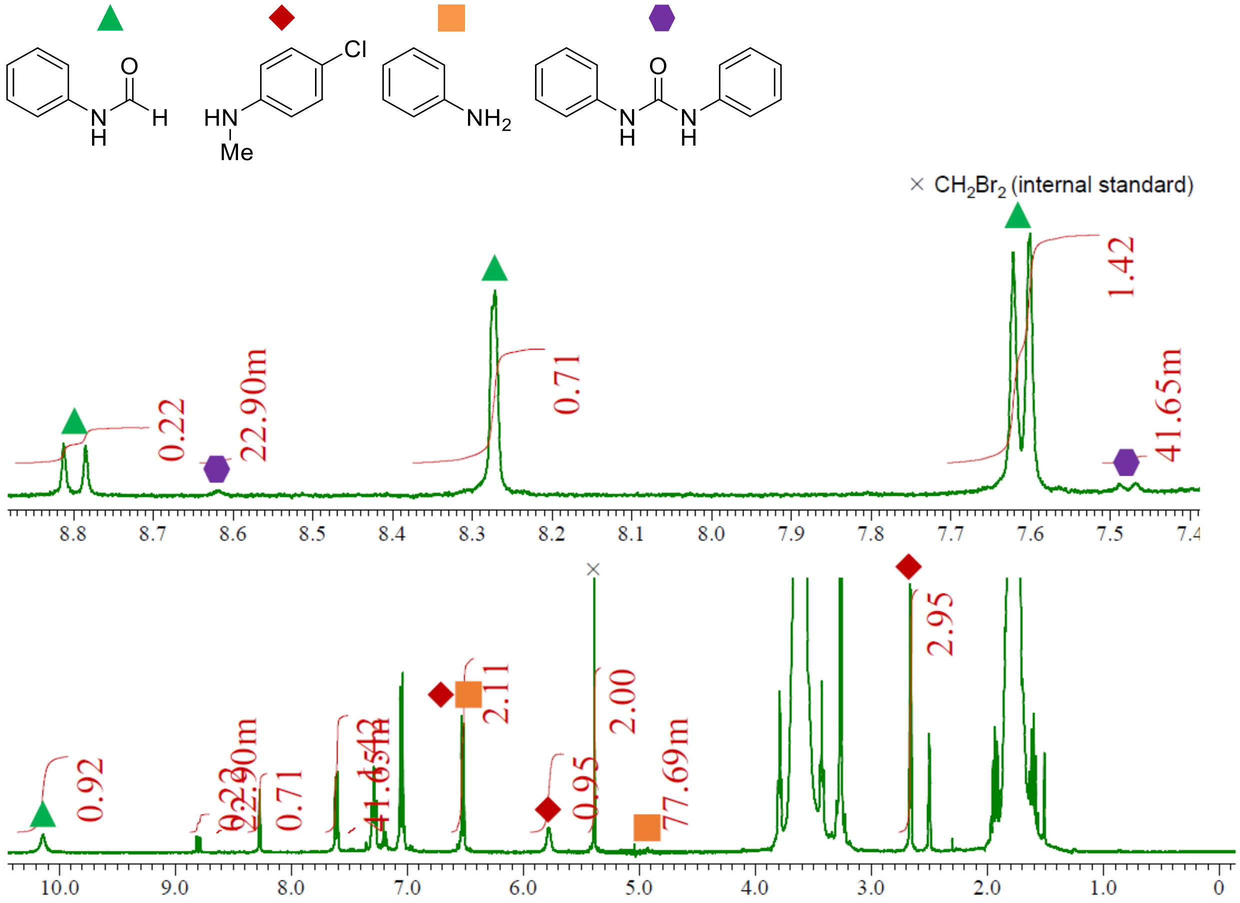

Supplementary Fig 106.

^{1}H NMR spectrum (400 MHz, DMSO-d_6/THF) of the hydrogenolysis of 1-(4-chlorophenyl)-1-methyl-3-phenylurea (**1ap**).

8-19. The hydrogenolysis of 1,3-bis(4-chlorophenyl)-1-methylurea (**1cp**)

The general procedure for Condition A was followed using 1,3-bis(4-chlorophenyl)-1-methylurea (**1cp**) (147.6 mg, 0.50 mmol) and **4** (9.4 mg, 15 μmol) in THF (3 mL) at 130 °C under H_2 (1 MPa) for 48 h. Conversion and yields were determined by ^{1}H NMR analysis by comparing with authentic samples.

^{1}H NMR spectrum of the hydrogenolysis of **1cp** is shown in Supplementary Fig 107.

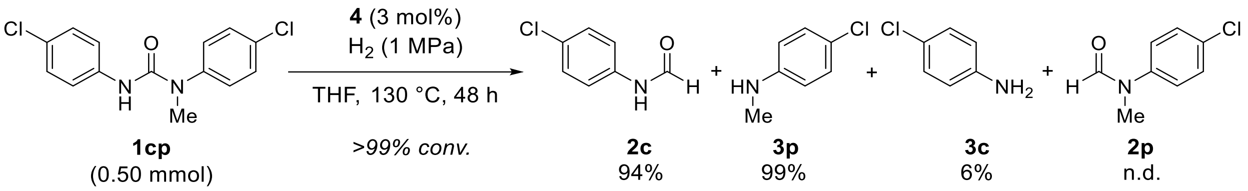

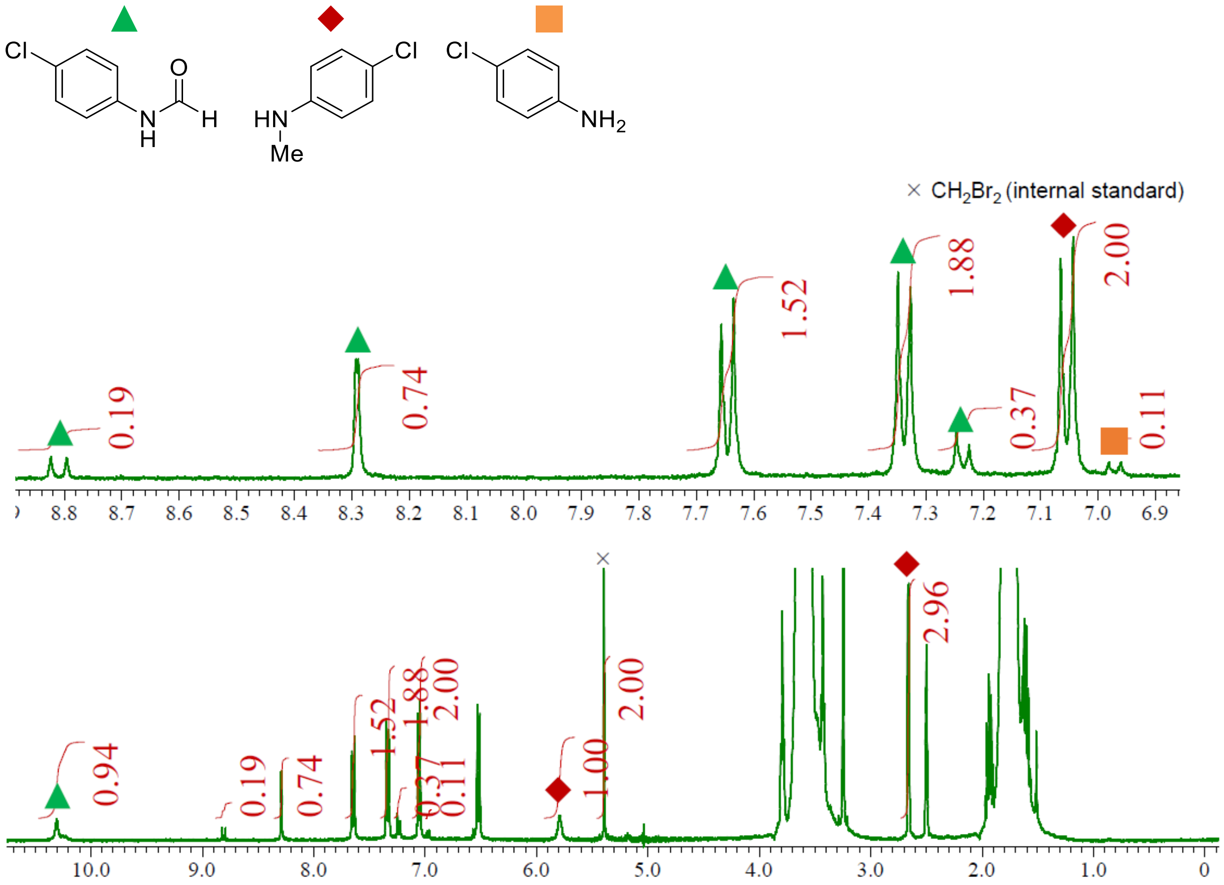

Supplementary Fig 107.

^{1}H NMR spectrum (400 MHz, DMSO-d_6/THF) of the hydrogenolysis of 1,3-bis(4-chlorophenyl)-1-methylurea (**1cp**).

8-20. The hydrogenolysis of 3-(4-butylphenyl)-1-(4-chlorophenyl)-1-methylurea (**1qp**)

The general procedure for Condition A was followed using 3-(4-butylphenyl)-1-(4-chlorophenyl)-1-methylurea (**1qp**) (158.4 mg, 0.50 mmol) and **4** (9.4 mg, 15 μmol) in THF (3 mL) at 130 °C under H_2 (1 MPa) for 48 h. Conversion and yields were determined by ^{1}H NMR analysis by comparing with authentic samples.

^{1}H NMR spectrum of the hydrogenolysis of **1qp** is shown in Supplementary Fig 108.

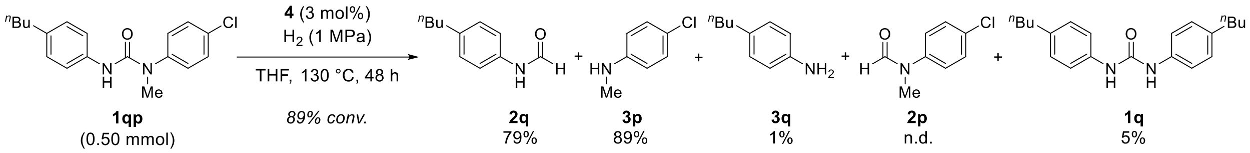

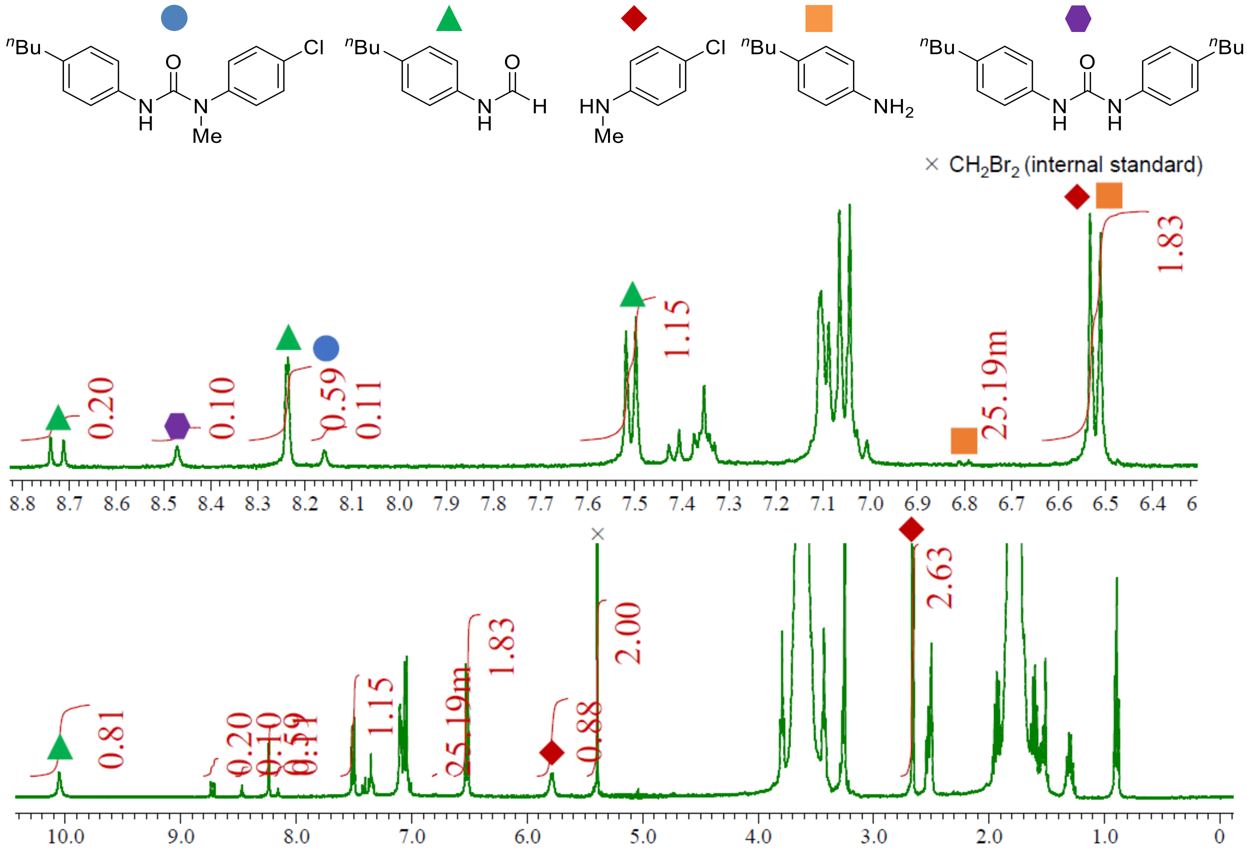

Supplementary Fig 108.

^{1}H NMR spectrum (400 MHz, DMSO-d_6/THF) of the hydrogenolysis of 3-(4-butylphenyl)-1-(4-chlorophenyl)-1-methylurea (**1qp**).

8-21. The hydrogenolysis of 3-(4-chlorophenyl)-1-(4-methoxylphenyl)-1-methylurea (**1cr**)

The general procedure for Condition A was followed using 3-(4-chlorophenyl)-1-(4-methoxylphenyl)-1-methylurea (**1cr**) (145.4 mg, 0.50 mmol) and **4** (9.4 mg, 15 µmol) in THF (3 mL) at 130 °C under H_2 (1 MPa) for 48 h. Conversion and yields were determined by ^{1}H NMR analysis by comparing with authentic samples.

^{1}H NMR spectrum of the hydrogenolysis of **1cr** is shown in Supplementary Fig 109.

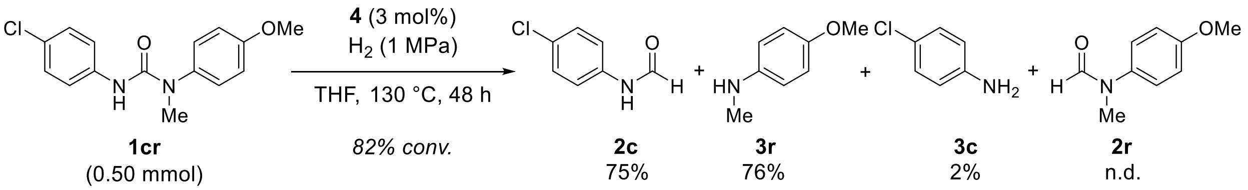

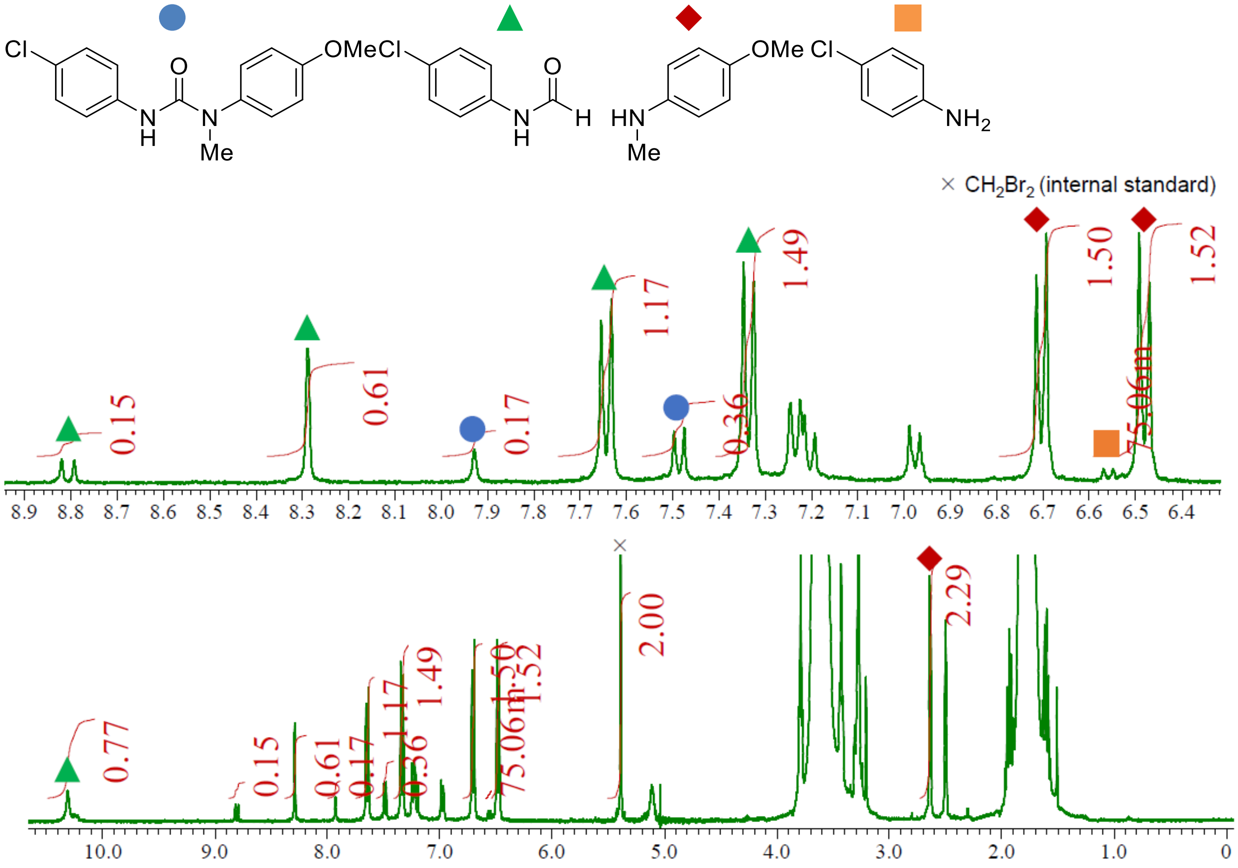

Supplementary Fig 109.

^{1}H NMR spectrum (400 MHz, DMSO-d_6/THF) of the hydrogenolysis of 3-(4-chlorophenyl)-1-(4-methoxylphenyl)-1-methylurea (**1cr**).

8-22. The hydrogenolysis of 3-(4-butylphenyl)-1-(4-methoxylphenyl)-1-methylurea (**1qr**)

The general procedure for Condition A was followed using 3-(4-butylphenyl)-1-(4-methoxylphenyl)-1-methylurea (**1qr**) (156.2 mg, 0.50 mmol) and **4** (9.4 mg, 15 µmol) in THF (3 mL) at 130 °C under H_2 (1 MPa) for 48 h. Conversion and yields were determined by ^{1}H NMR analysis by comparing with authentic samples.

^{1}H NMR spectrum of the hydrogenolysis of **1qr** is shown in Supplementary Fig 110.

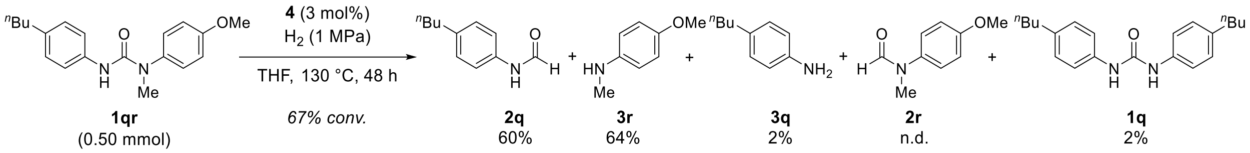

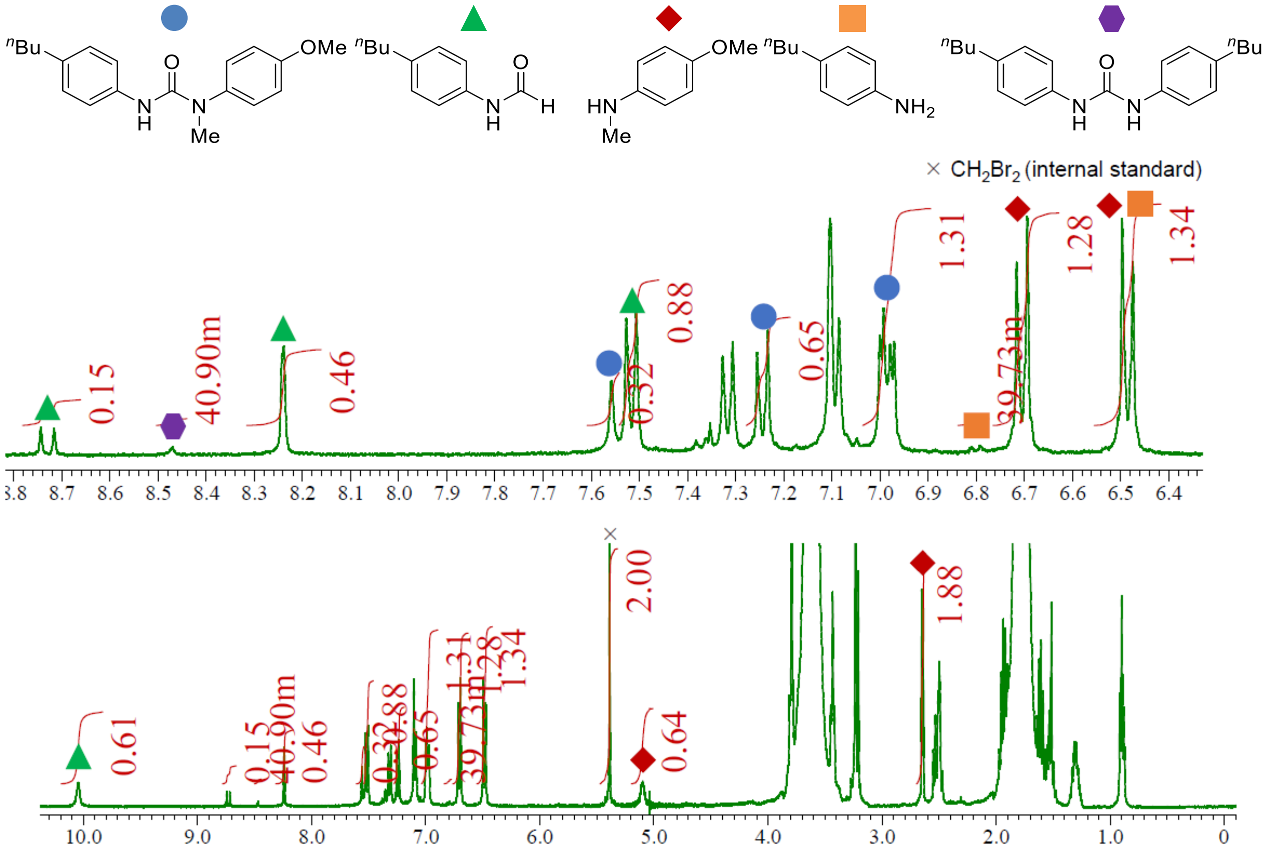

Supplementary Fig 110.

^{1}H NMR spectrum (400 MHz, DMSO-d_6/THF) of the hydrogenolysis of 3-(4-butylphenyl)-1-(4-methoxylphenyl)-1-methylurea (**1qr**).

8-23. The hydrogenolysis of *N*-phenylmorpholine-4-carboxamide (**1as**) (Condition A)

The general procedure for Condition A was followed using *N*-phenylmorpholine-4-carboxamide (**1as**) (103.1 mg, 0.50 mmol) and **4** (9.4 mg, 15 µmol) in THF (3 mL) at 130 °C under H_2 (1 MPa) for 48 h. Conversion and yields were determined by ^1H NMR analysis by comparing with authentic samples.

^1H NMR spectrum of the hydrogenolysis of **1as** is shown in Supplementary Fig 111.

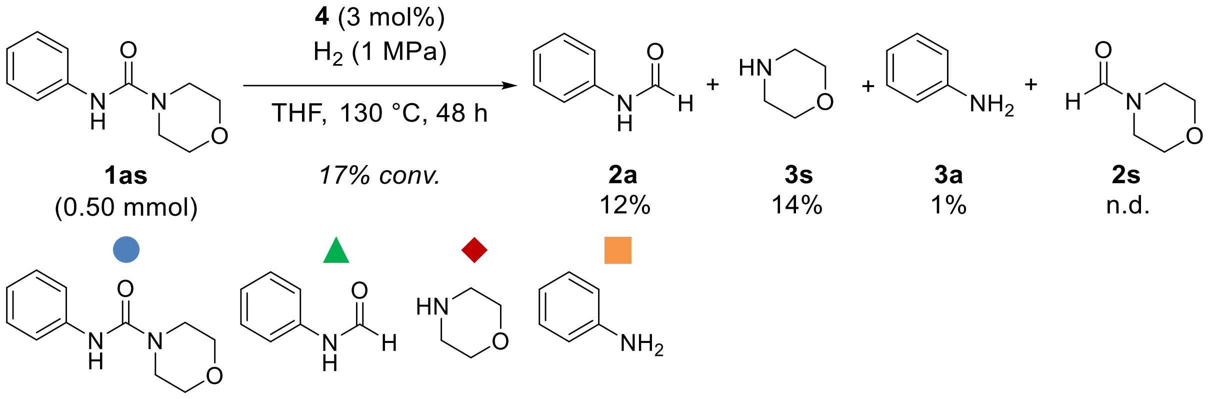

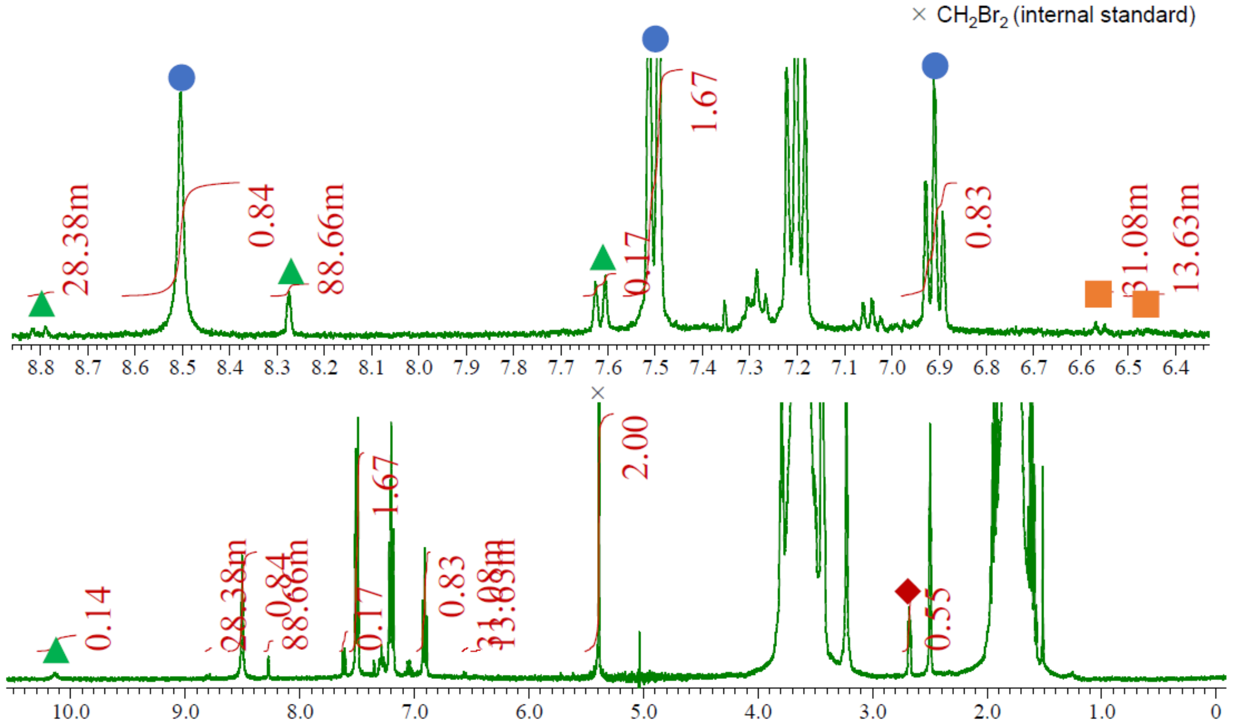

Supplementary Fig 111.

^1H NMR spectrum (400 MHz, DMSO-d_6/THF) of the hydrogenolysis of *N*-phenylmorpholine-4-carboxamide (**1as**) (Condition A).

8-24. The hydrogenolysis of *N*-phenylmorpholine-4-carboxamide (**1as**) (Condition B)

The general procedure for Condition B was followed using *N*-phenylmorpholine-4-carboxamide (**1as**) (103.1 mg, 0.50 mmol), **4** (9.4 mg, 15 µmol), and KO^{t}Bu (5.6 mg, 0.050 mmol) in toluene (3 mL) at 130 °C under H_2 (1 MPa) for 48 h. Conversion and yields were determined by ^{1}H NMR analysis by comparing with authentic samples.

^{1}H NMR spectrum of the hydrogenolysis of **1as** is shown in Supplementary Fig 112.

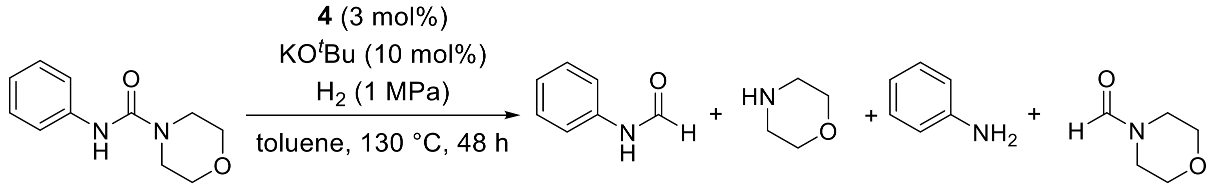

1as (0.50 mmol) | >99% conv. | **2a** 6% | **3s** 16% | **3a** 83% | **2s** 77%

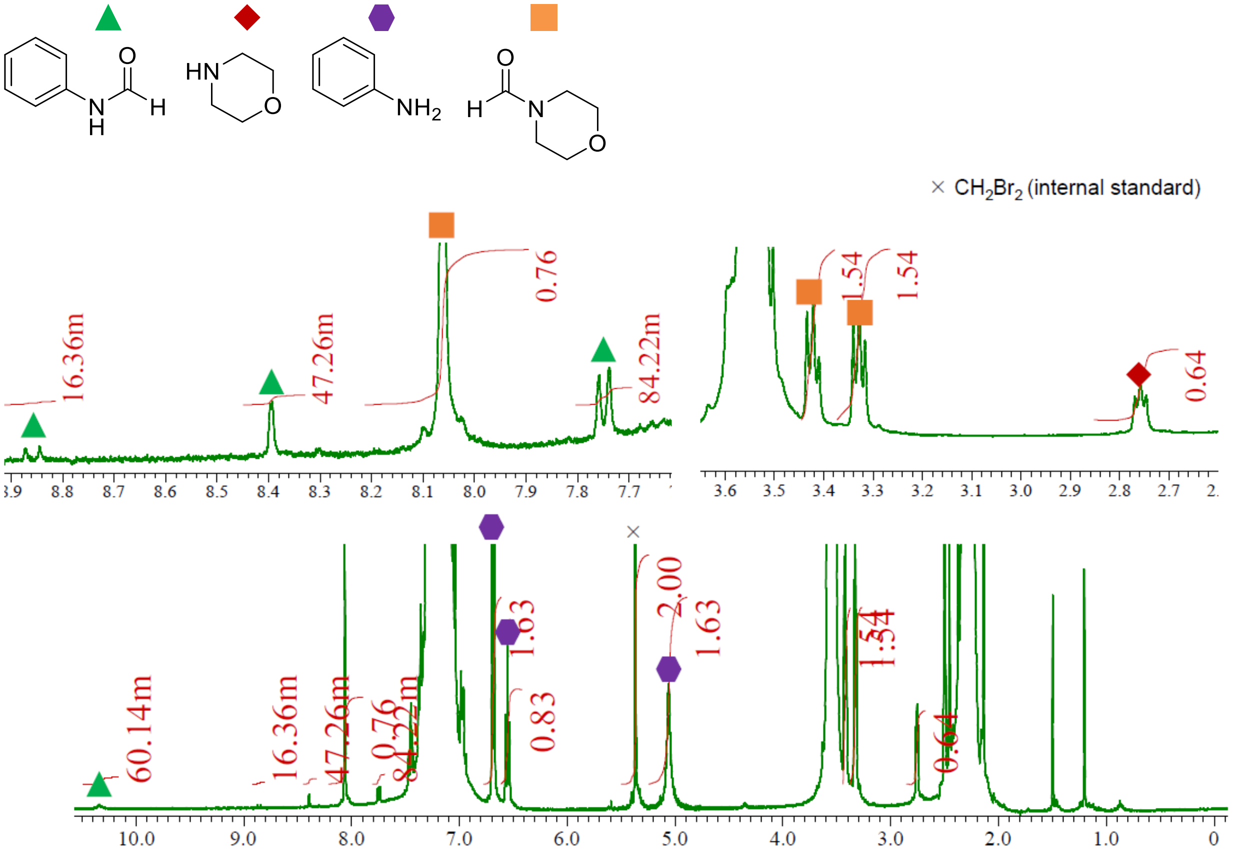

Supplementary Fig 112.

^{1}H NMR spectrum (400 MHz, DMSO-d_6/toluene) of the hydrogenolysis of *N*-phenylmorpholine-4-carboxamide (**1as**) (Condition B).

8-25. The hydrogenolysis of polyurea **17** (Fig. 5)

The general procedure for the hydrogenolysis of polyurea resin **17** was followed using **17** (32.9 mg, 0.16 mmol/urea moiety), **4** (3.1 mg, 5 μmol), and KO^{t}Bu (1.9 mg, 0.017 mmol) in THF (3 mL) at 130 °C under H_2 (1 MPa) for 18 h. Conversion and yields were determined by ^{1}H NMR analysis by comparing with authentic samples. *N*,*N*'-Dimethyl-1,6-diaminohexane (**16**) was isolated as a colorless liquid (5.8 mg, 48%). *N*,*N'*-(Methylenebis(4,1-phenylene))diformamide (**18**) was isolated by silica gel column chromatography (CH_2Cl_2/ethyl acetate) as a white solid (13.8 mg, 65%).

^{1}H NMR spectrum of the hydrogenolysis of **17** is shown in Supplementary Fig 113.

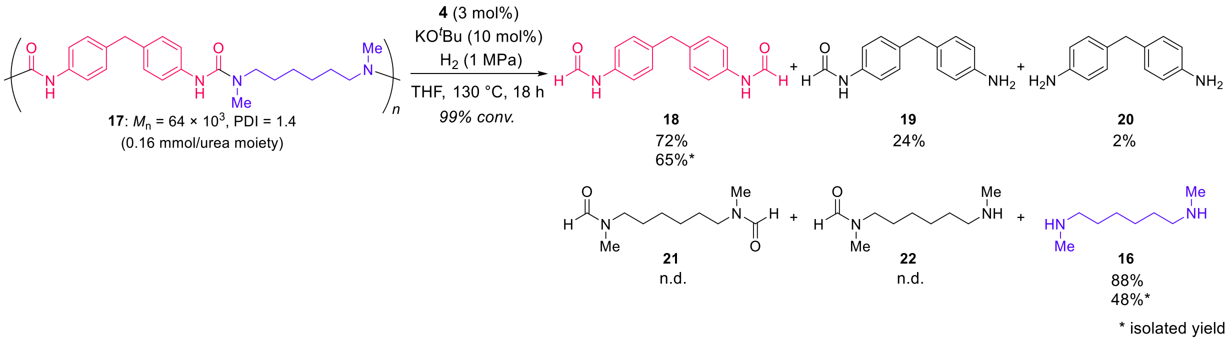

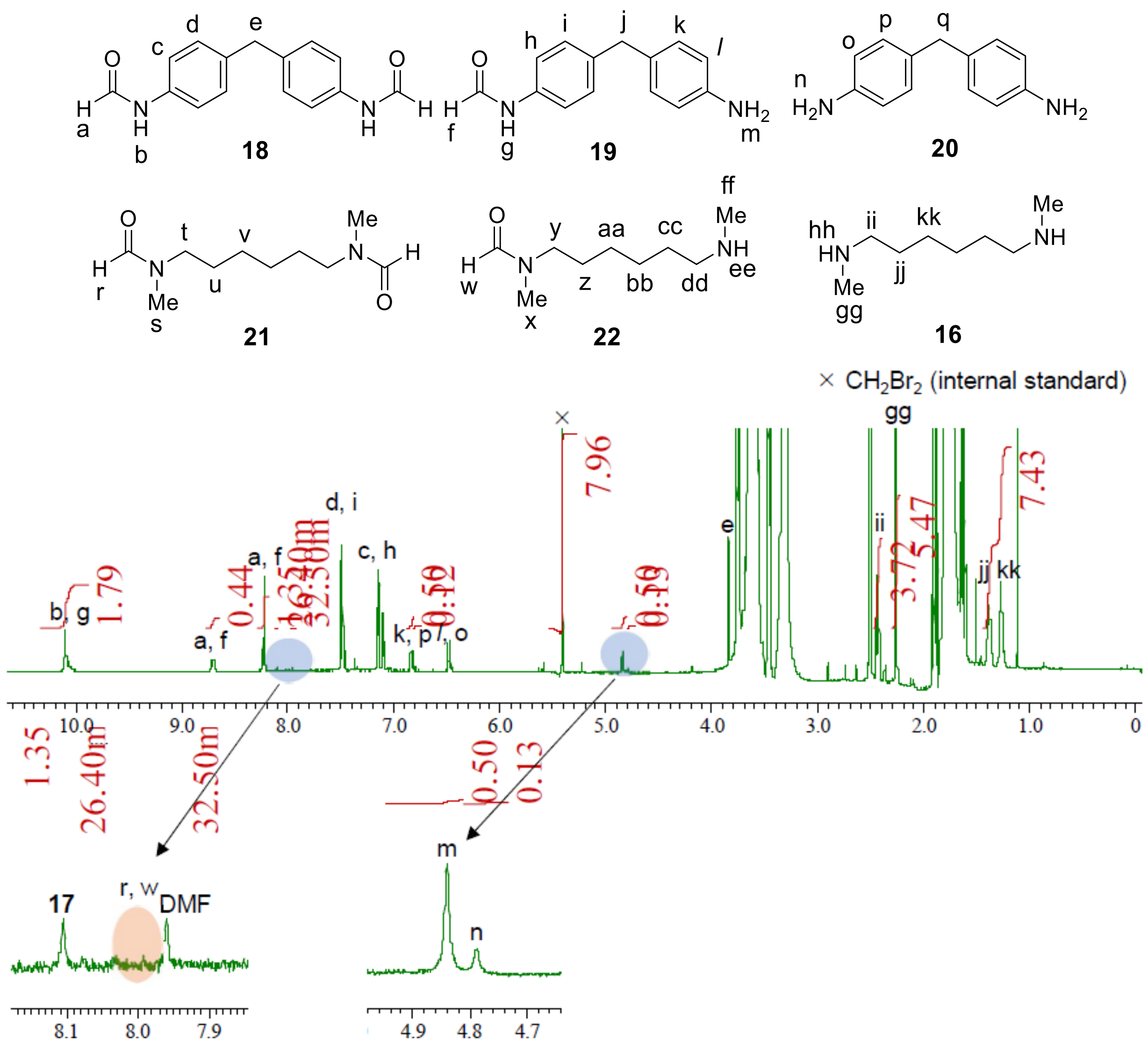

Supplementary Fig 113.

^{1}H NMR spectrum (500 MHz, DMSO-d_6/THF) of the hydrogenolysis of polyurea resin **17** (Fig. 5).

_N,N'_-(methylenebis(4,1-phenylene))diformamide (18)

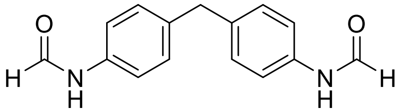

^{1}H NMR (500 MHz, DMSO-d_6): δ 10.1 (br, 1.5H), 10.0 (d, J = 11.0 Hz, 0.5H), 8.70 (d, J = 10.5 Hz, 0.5H), 8.22 (d, J = 2.0 Hz, 1.5H), 7.48 (d, J = 8.5 Hz, 3H), 7.14 (d, J = 8.5 Hz, 4H), 7.09 (d, J = 8.5 Hz, 1H), 3.83 (s, 2H); ^{13}C NMR (126 MHz, DMSO-d_6): δ 162.5, 159.4, 136.8, 136.6, 136.3, 136.2, 129.5, 129.0, 119.3, 117.8, 40.0 (overlapped with solvent peaks and confirmed by DEPT analysis), 39.8 (overlapped with solvent peaks and confirmed by DEPT analysis).
Two tautomers were observed in approximately 1:3 ratio. These data were consistent with those in literature[44].

_N,N'_-dimethyl-1,6-diaminohexane (16)

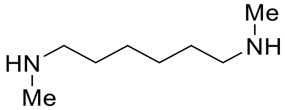

^{1}H NMR (500 MHz, $CDCl_3$): δ 2.55 (t, J = 7.3 Hz, 4H), 2.42 (s, 6H), 1.51–1.45 (m, 4H), 1.34–1.32 (m, 4H), 1.25 (br, 2H); ^{13}C NMR (126 MHz, $CDCl_3$): δ 52.3, 36.7, 30.1, 27.5.
These data were consistent with authentic sample.

9. Copies of Spectra for Isolated Products

^{1}H NMR spectrum (400 MHz, DMSO-d_6) of 1,3-bis(4-fluorophenyl)urea (**1b**).

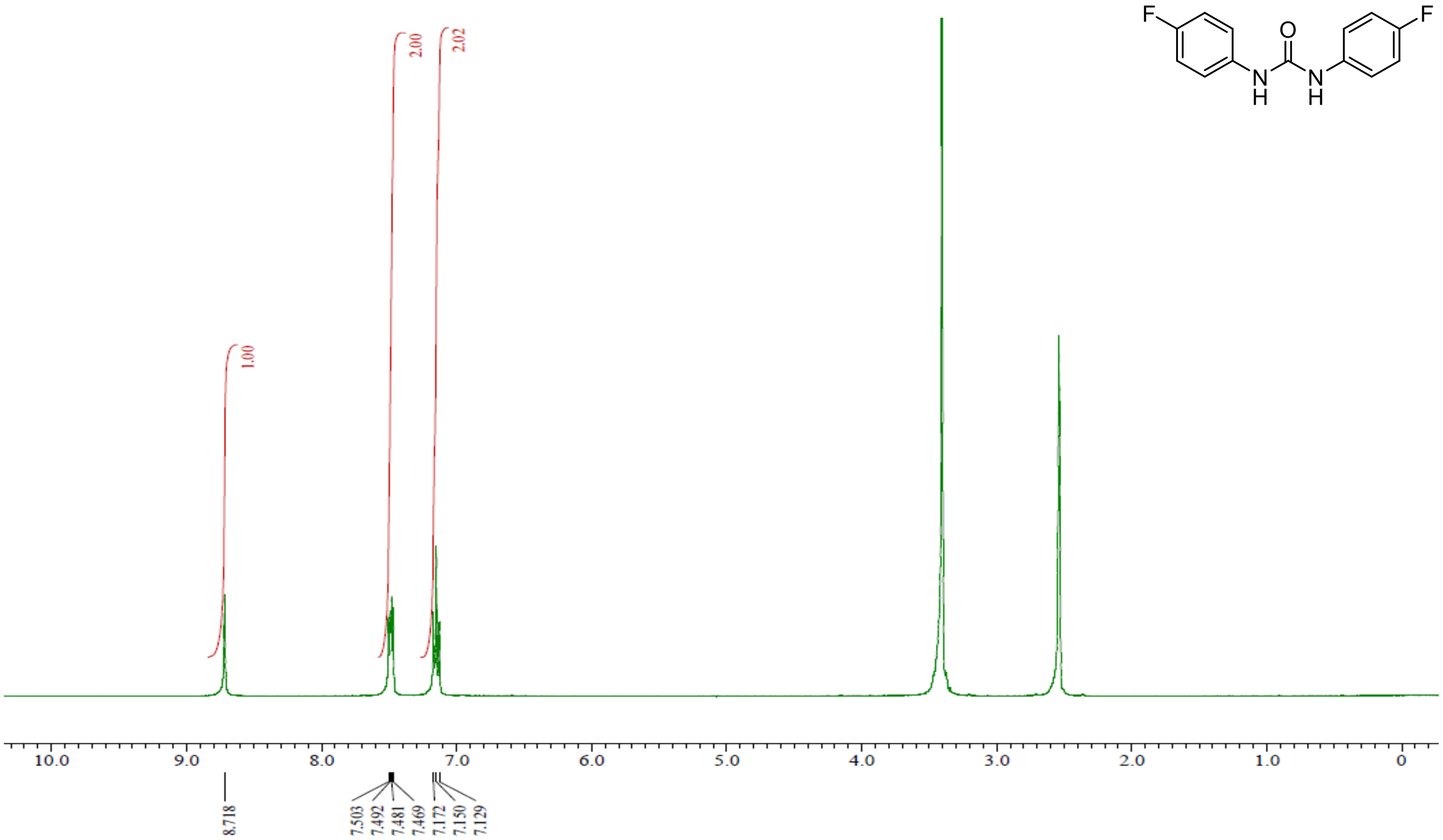

^{13}C NMR spectrum (101 MHz, DMSO-d_6) of 1,3-bis(4-fluorophenyl)urea (**1b**).

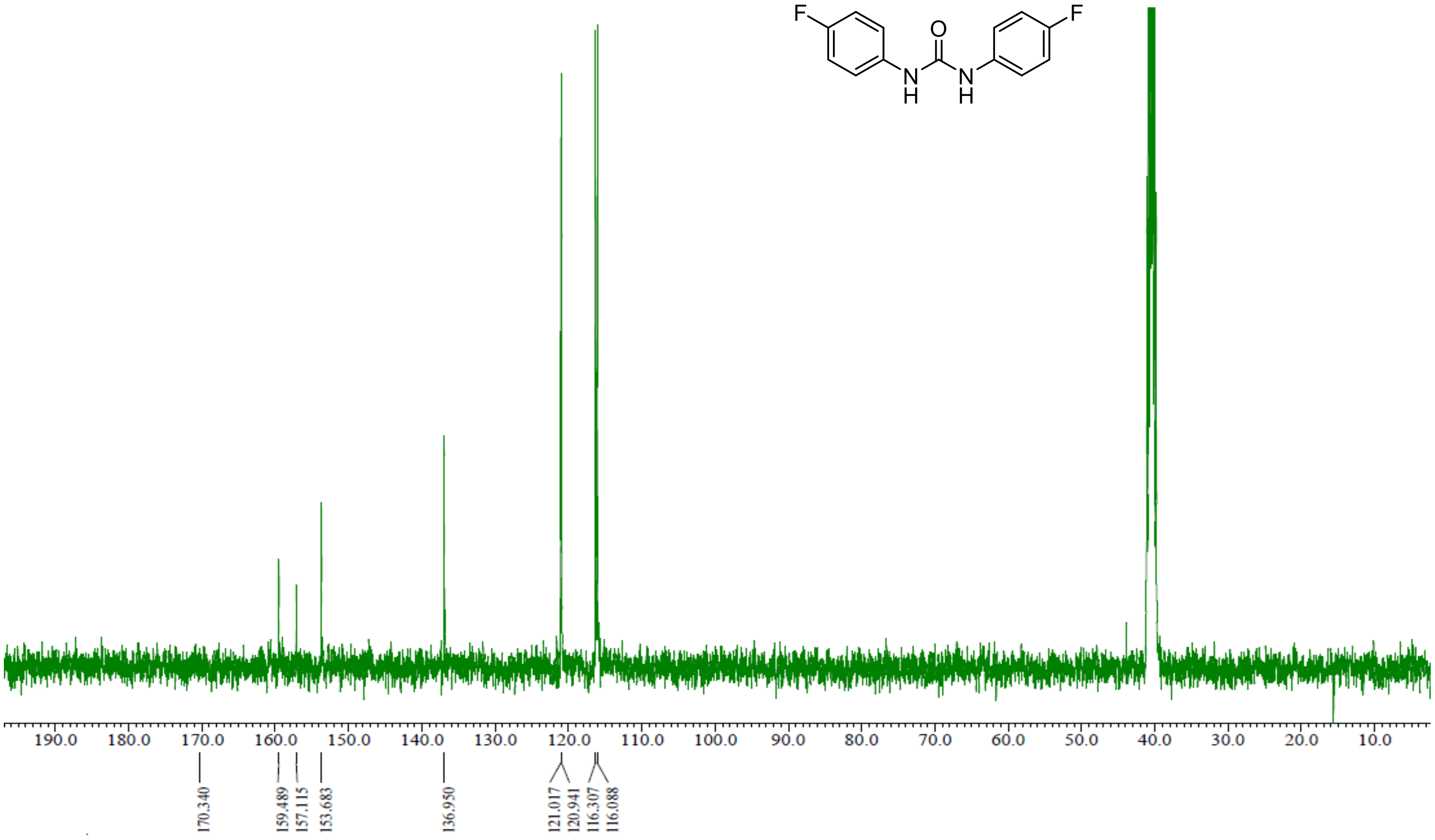

^{19}F NMR spectrum (376 MHz, DMSO-d_6) of 1,3-bis(4-fluorophenyl)urea (**1b**).

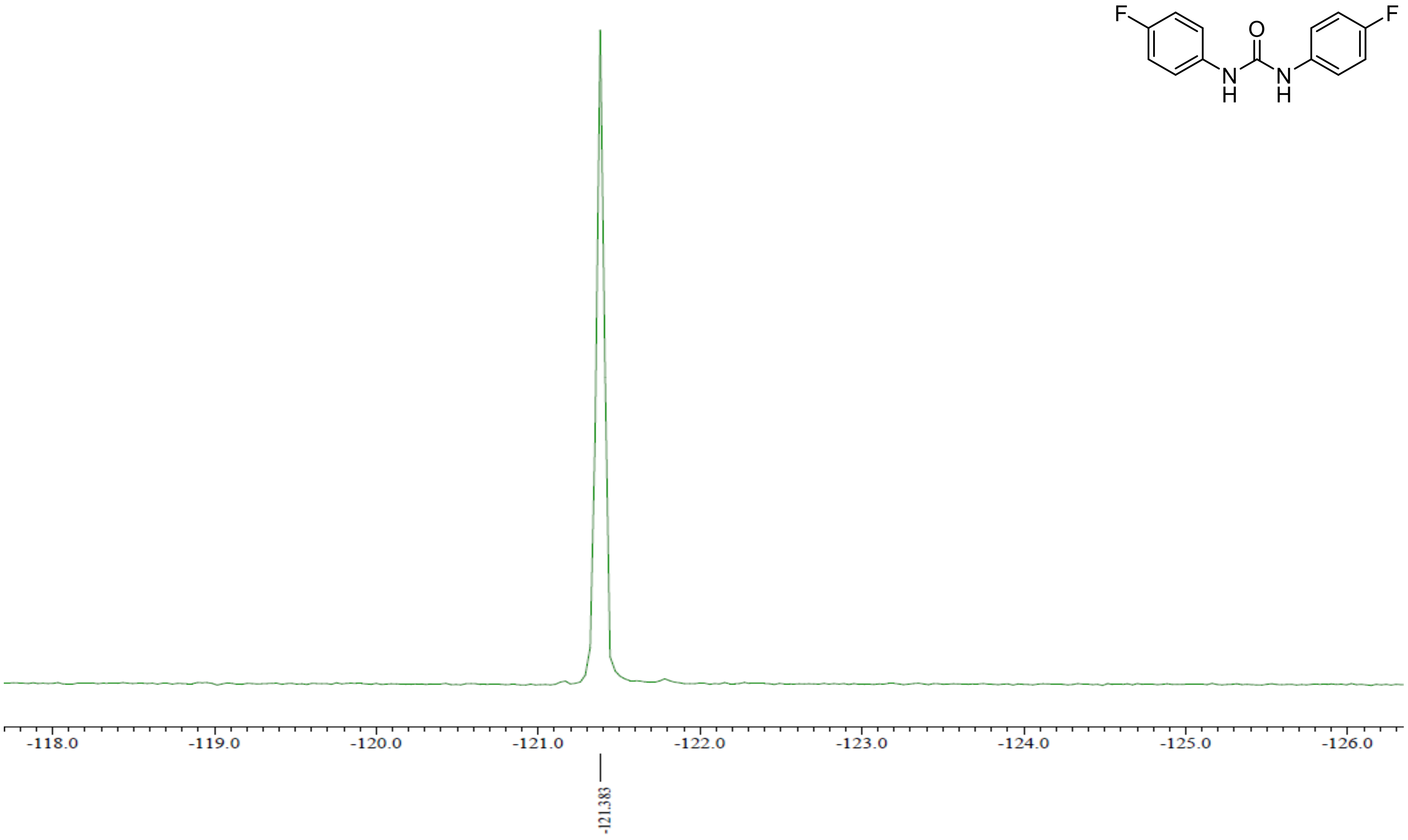

^{1}H NMR spectrum (400 MHz, DMSO-d_6) of 1,3-bis(4-chlorophenyl)urea (**1c**).

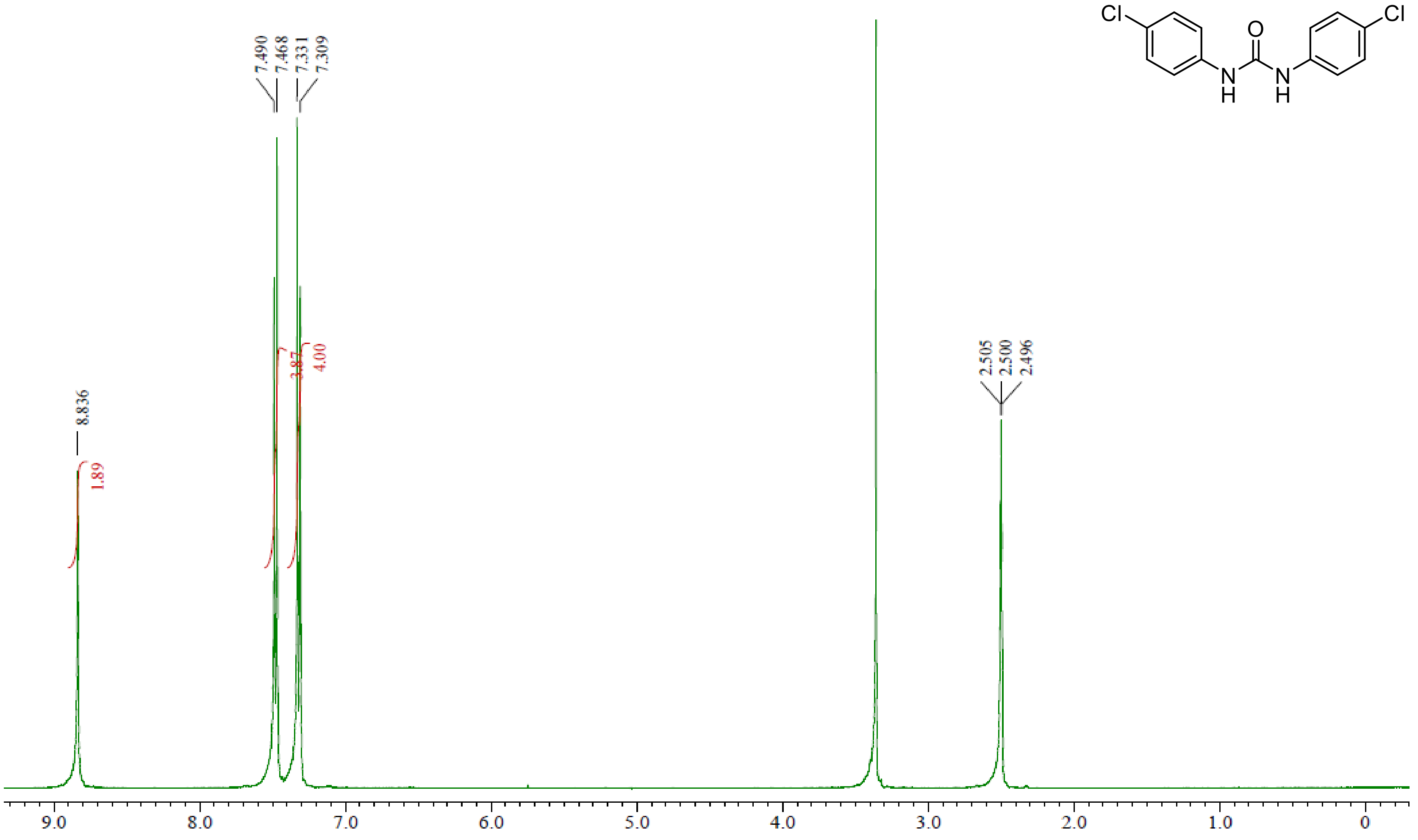

^{13}C NMR spectrum (101 MHz, DMSO-d_6) of 1,3-bis(4-chlorophenyl)urea (**1c**).

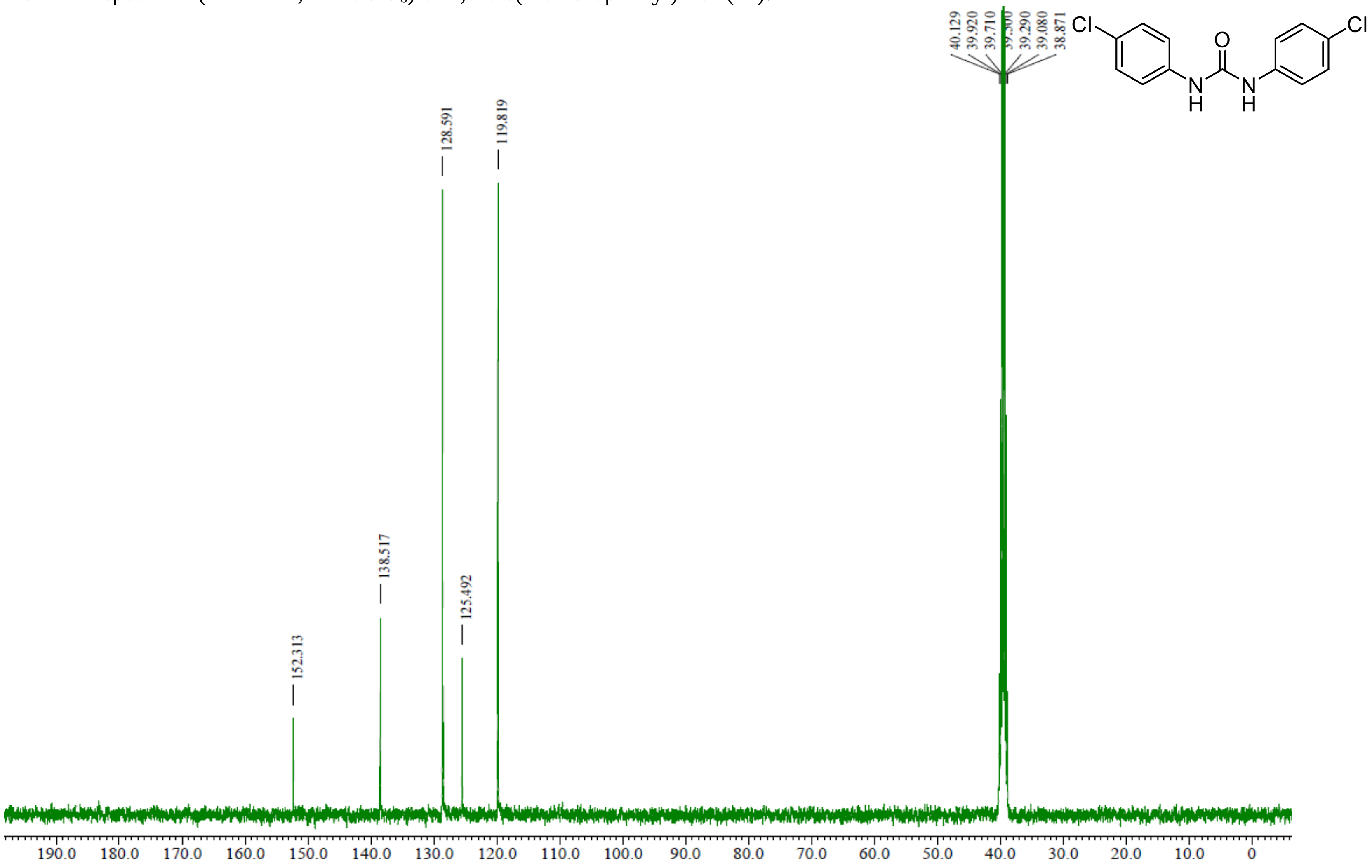

^{1}H NMR spectrum (400 MHz, DMSO-d_6) of 1,3-bis(3-chlorophenyl)urea (**1d**).

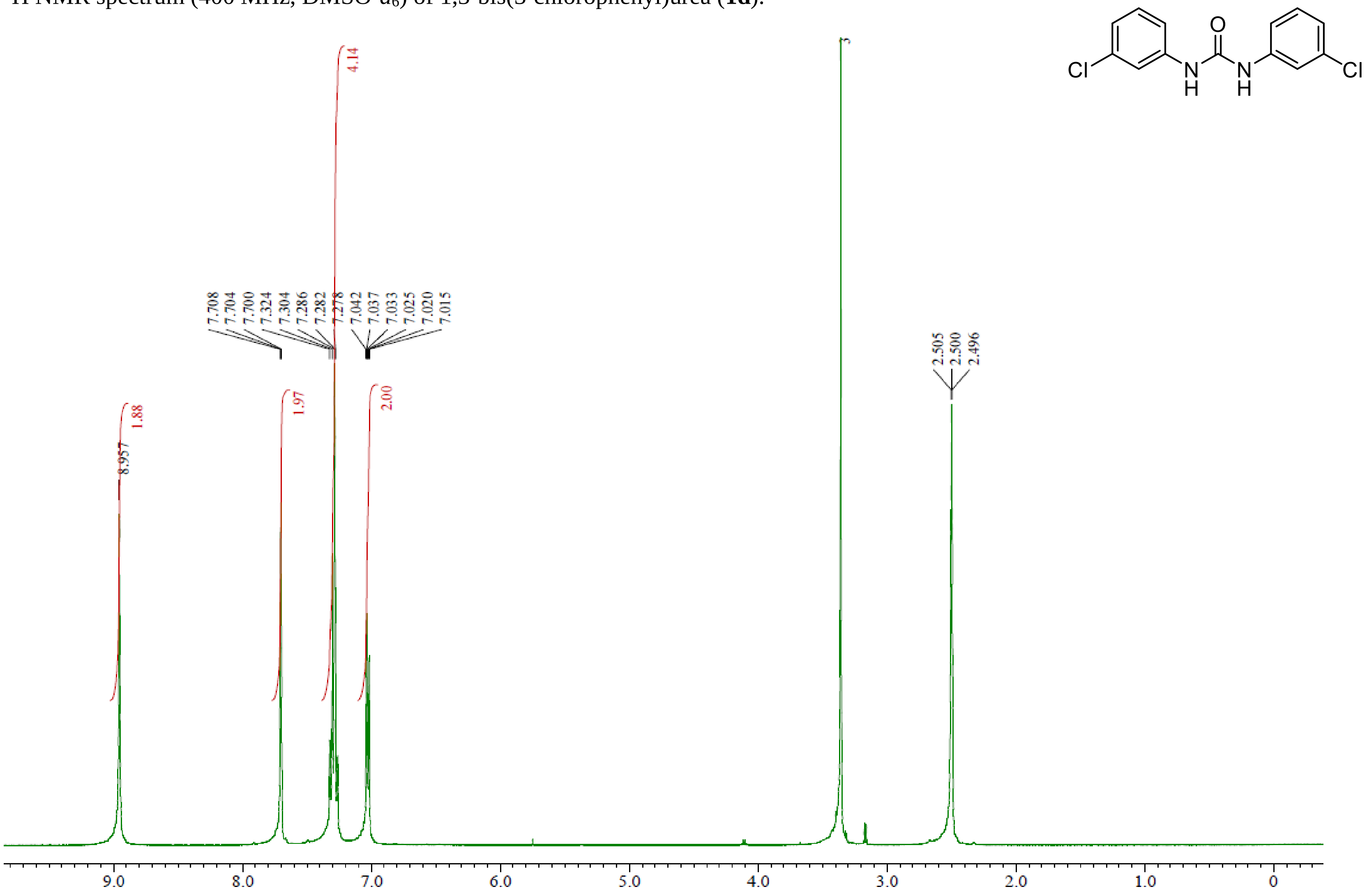

^{13}C NMR spectrum (101 MHz, DMSO-d_6) of 1,3-bis(3-chlorophenyl)urea (**1d**).

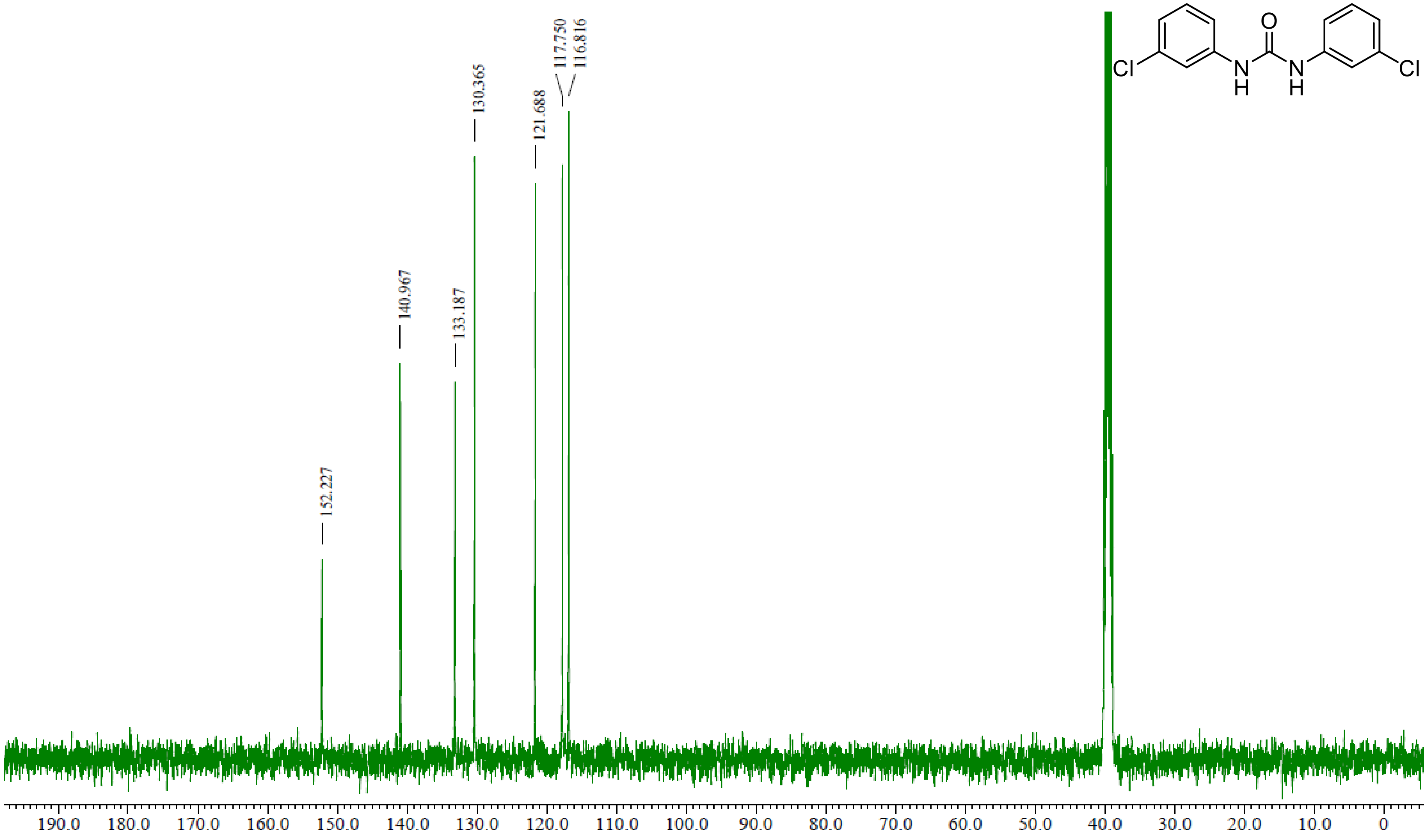

^{1}H NMR spectrum (400 MHz, DMSO-d_6) of 1,3-bis(2-chlorophenyl)urea (**1e**).

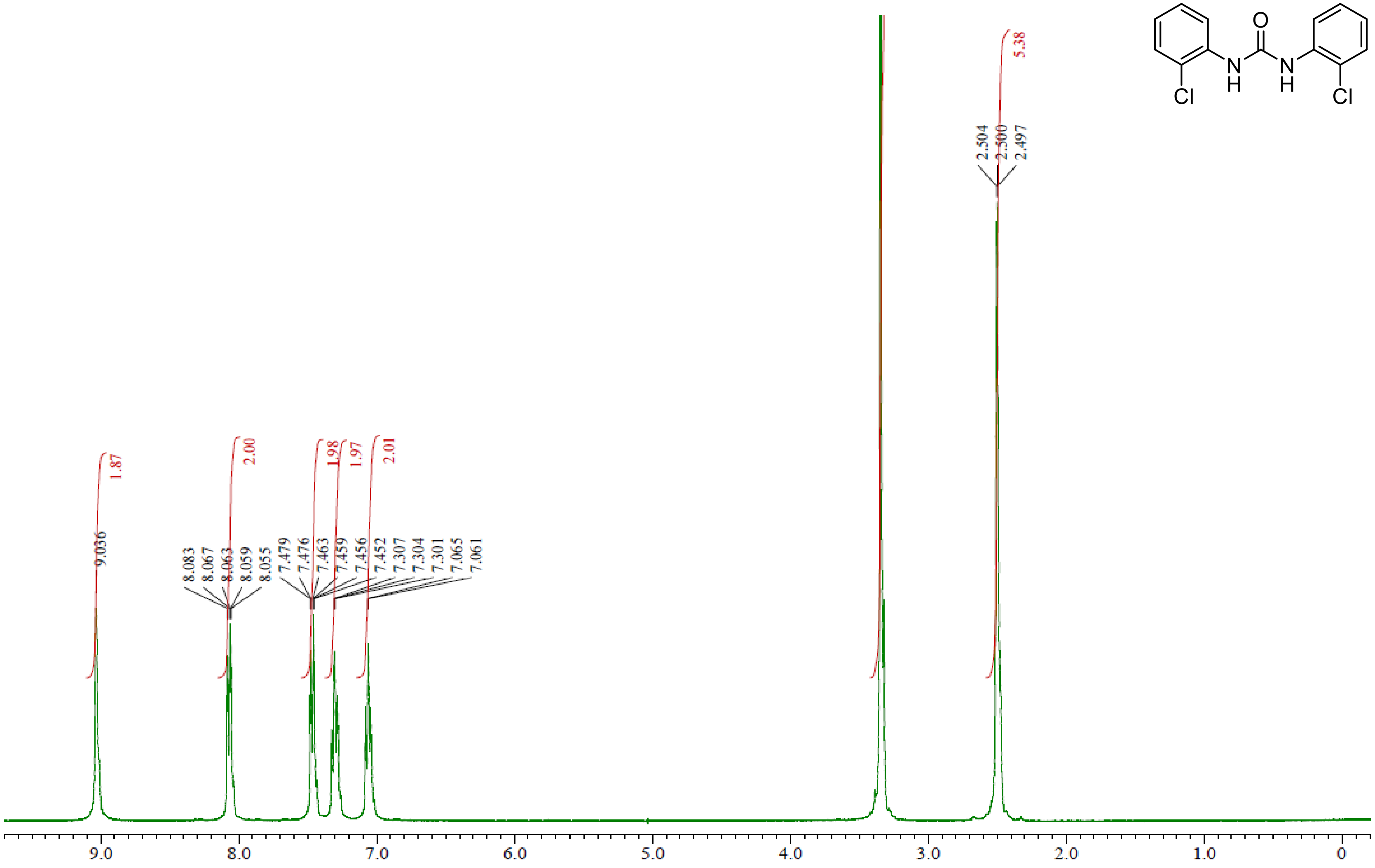

^{13}C NMR spectrum (101 MHz, DMSO-d_6) of 1,3-bis(2-chlorophenyl)urea (**1e**).

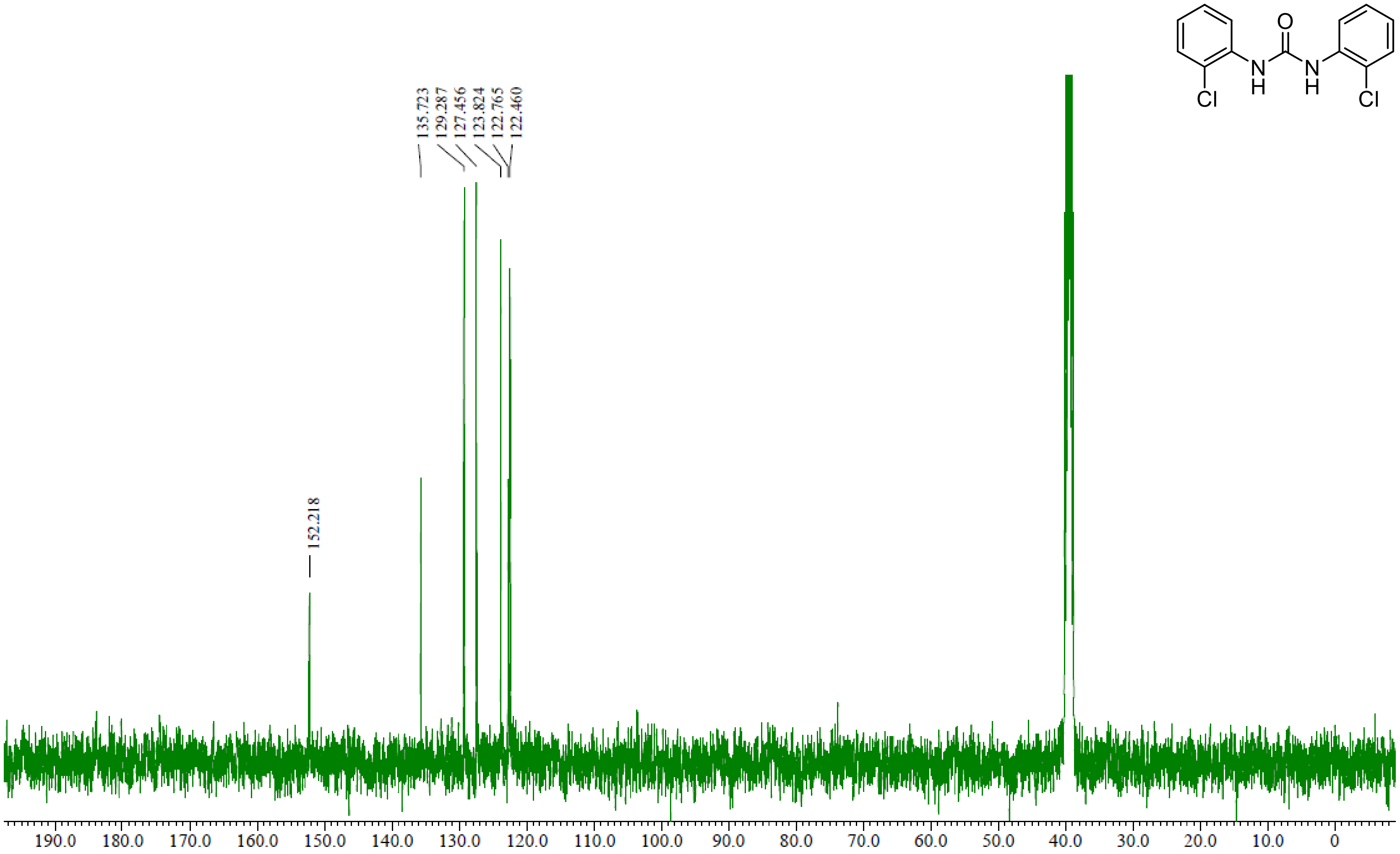

^{1}H NMR spectrum (400 MHz, DMSO-d_6) of 1,3-bis(4-bromophenyl)urea (**1f**).

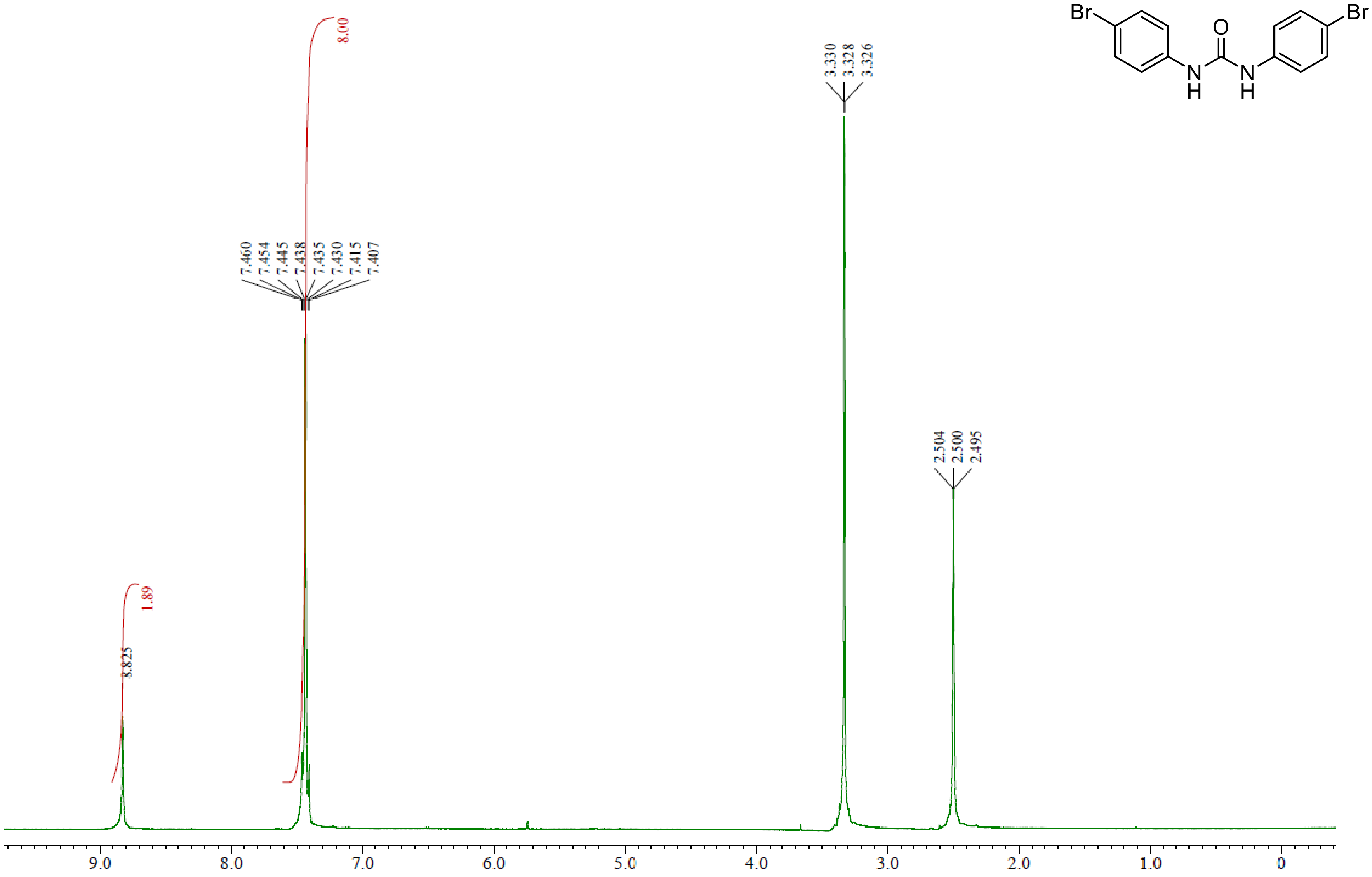

^{13}C NMR spectrum (101 MHz, DMSO-d_6) of 1,3-bis(4-bromophenyl)urea (**1f**).

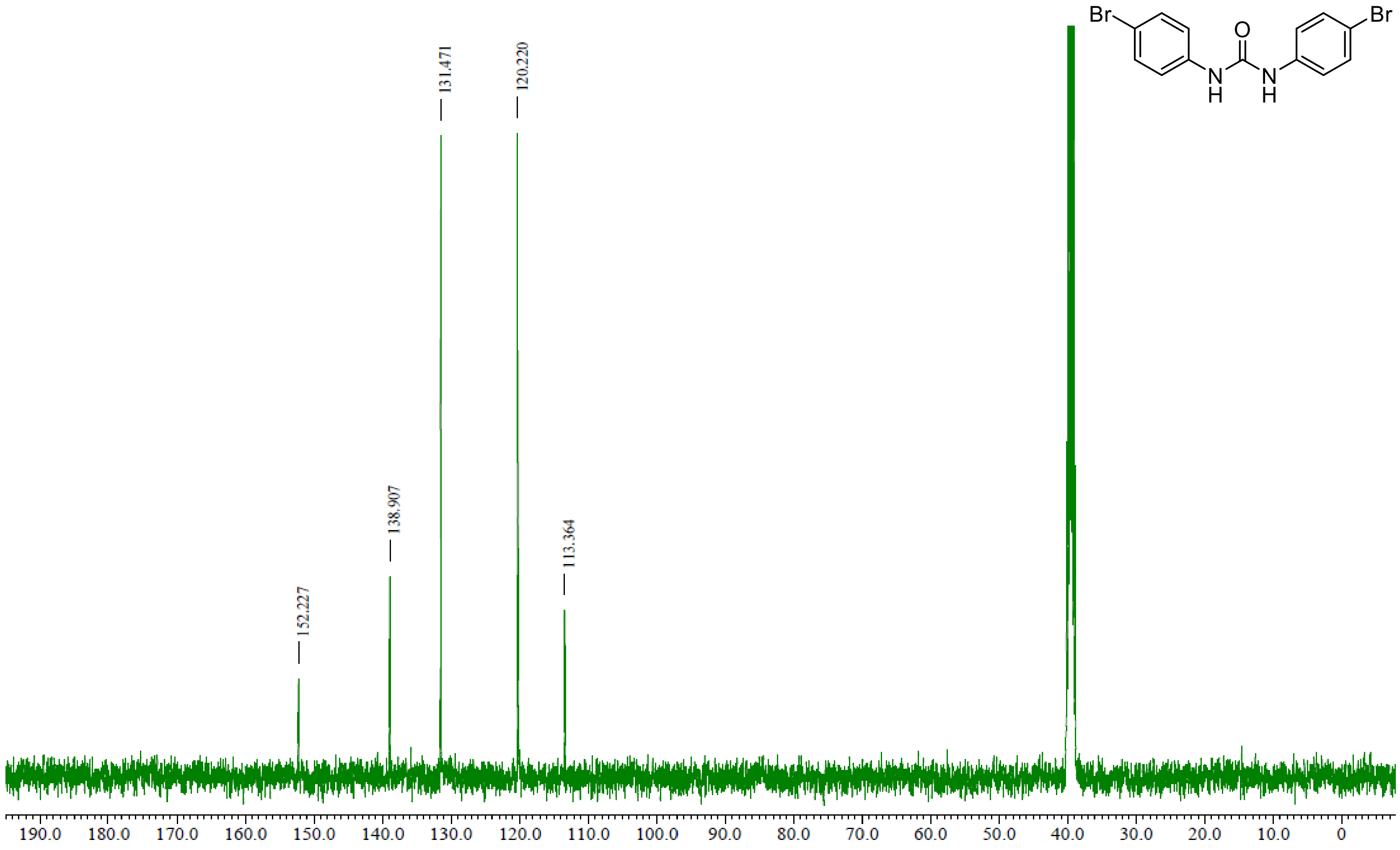

^{1}H NMR spectrum (500 MHz, DMSO-d_6) of 1,3-bis(4-*tert*-butylphenyl)urea (**1g**).

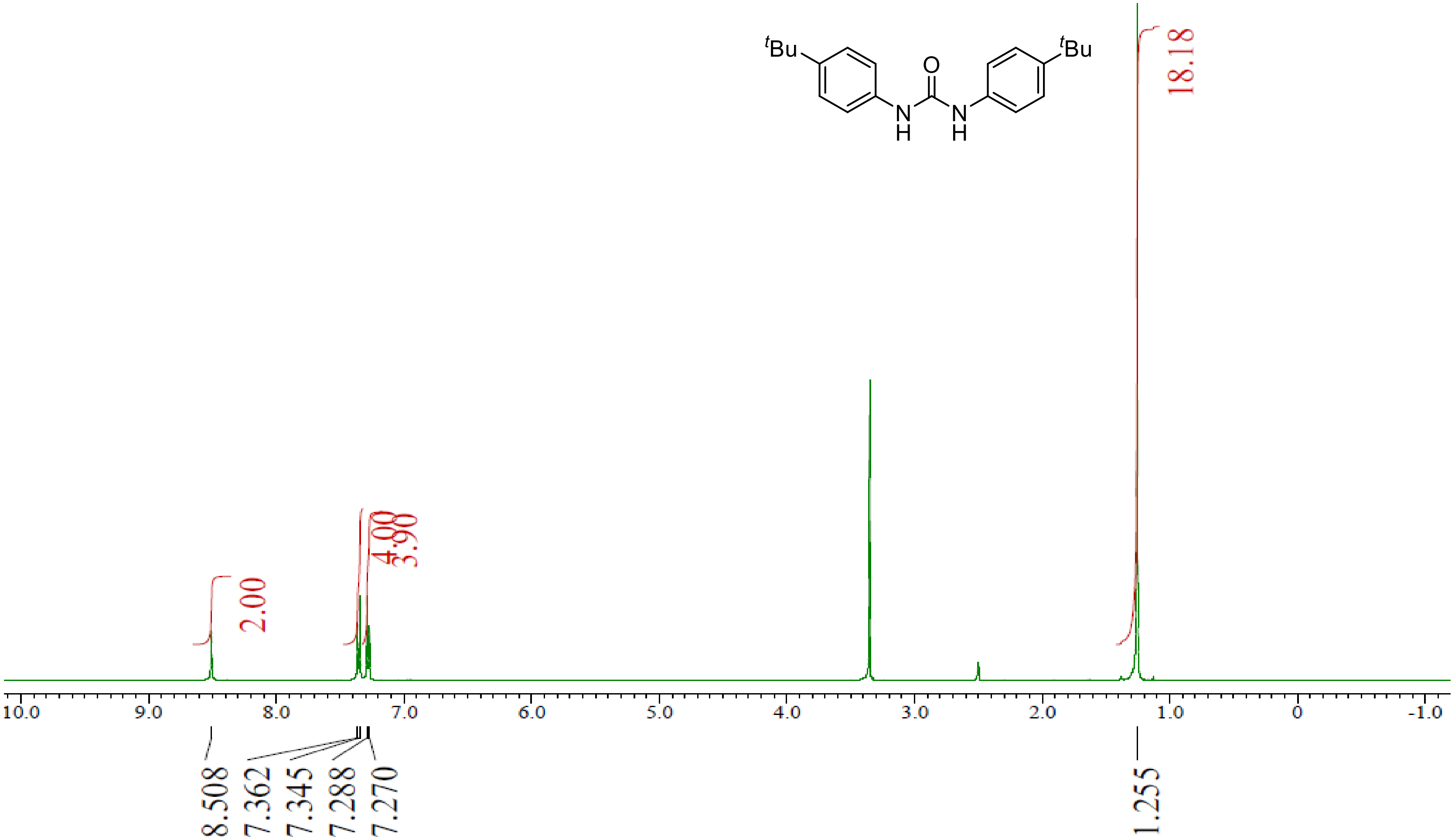

^{13}C NMR spectrum (126 MHz, DMSO-d_6) of 1,3-bis(4-*tert*-butylphenyl)urea (**1g**).

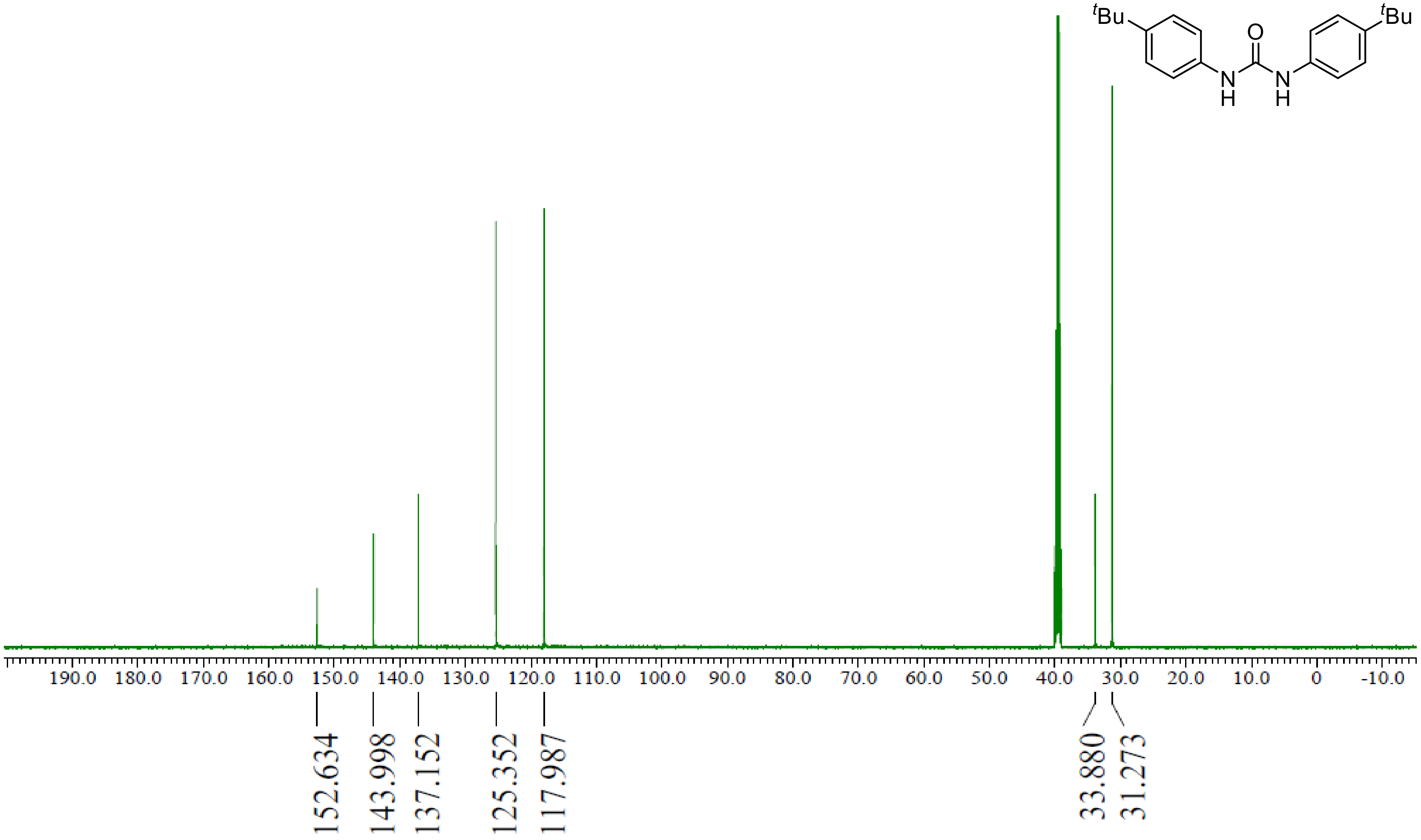

^{1}H NMR spectrum (400 MHz, DMSO-d_6) of 1,3-bis(4-methoxyphenyl)urea (**1h**).

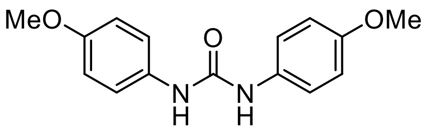

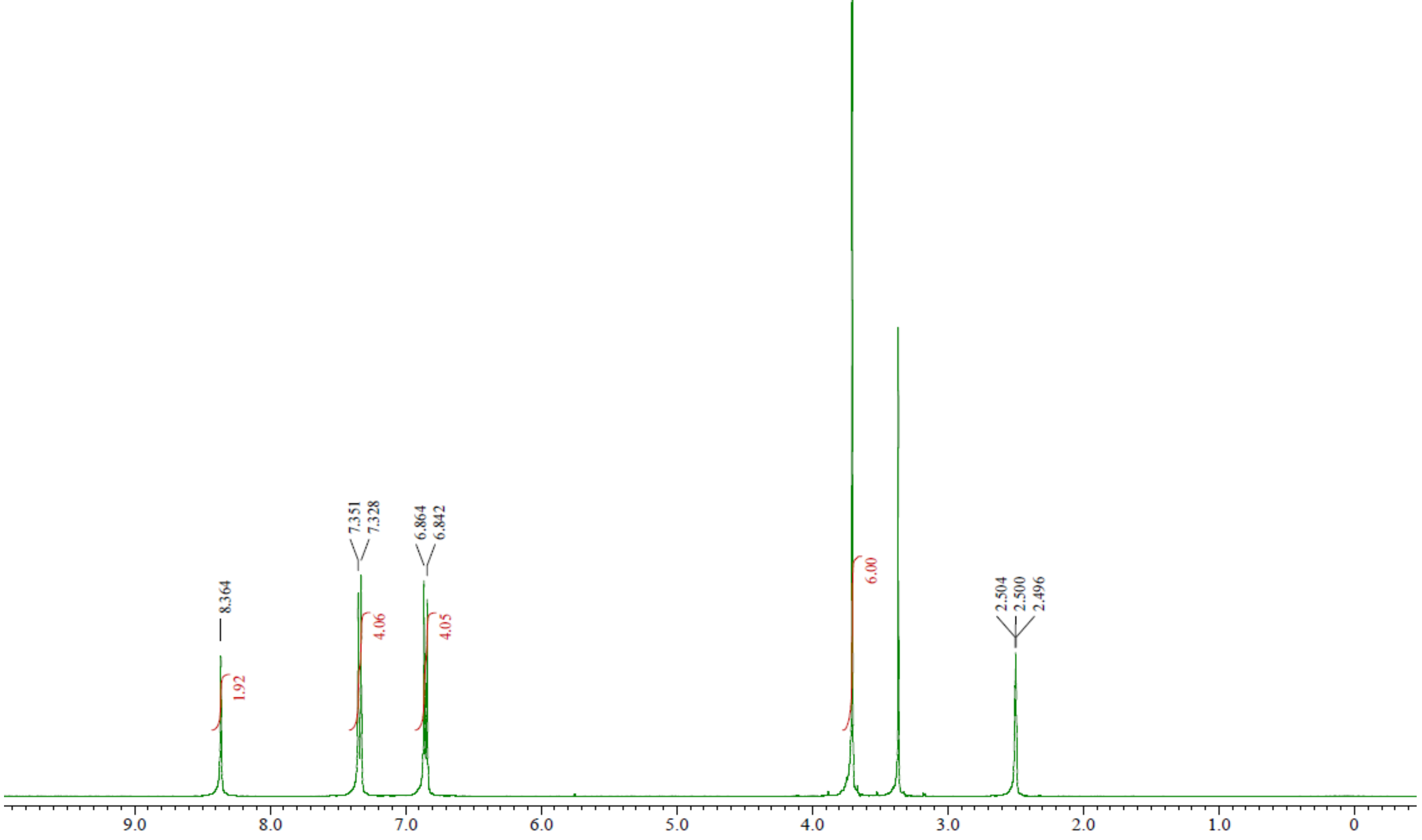

^{13}C NMR spectrum (101 MHz, DMSO-d_6) of 1,3-bis(4-methoxyphenyl)urea (**1h**).

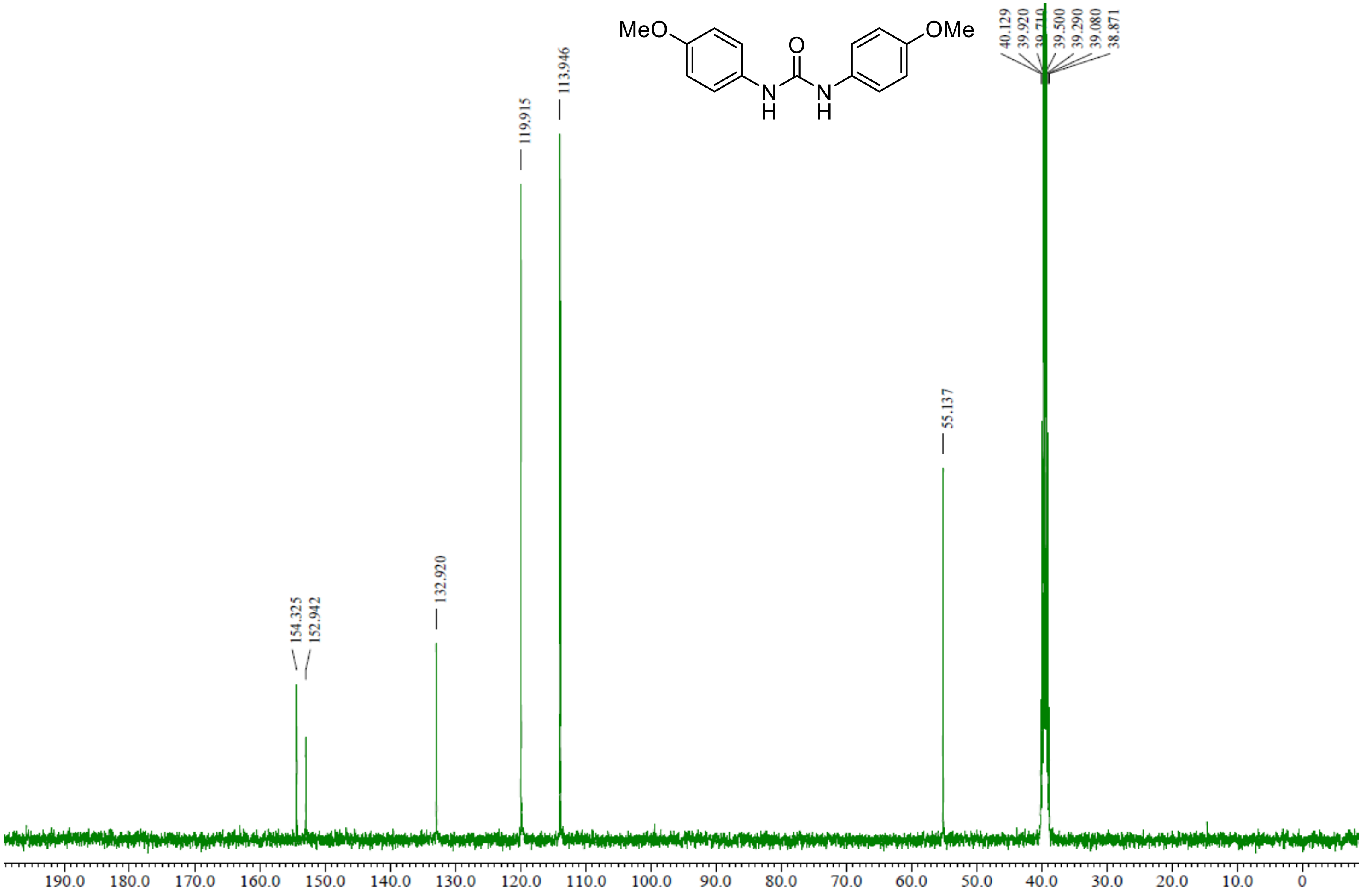

^{1}H NMR spectrum (400 MHz, DMSO-d_6) of 1,3-bis(4-dimethylaminophenyl)urea (**1i**).

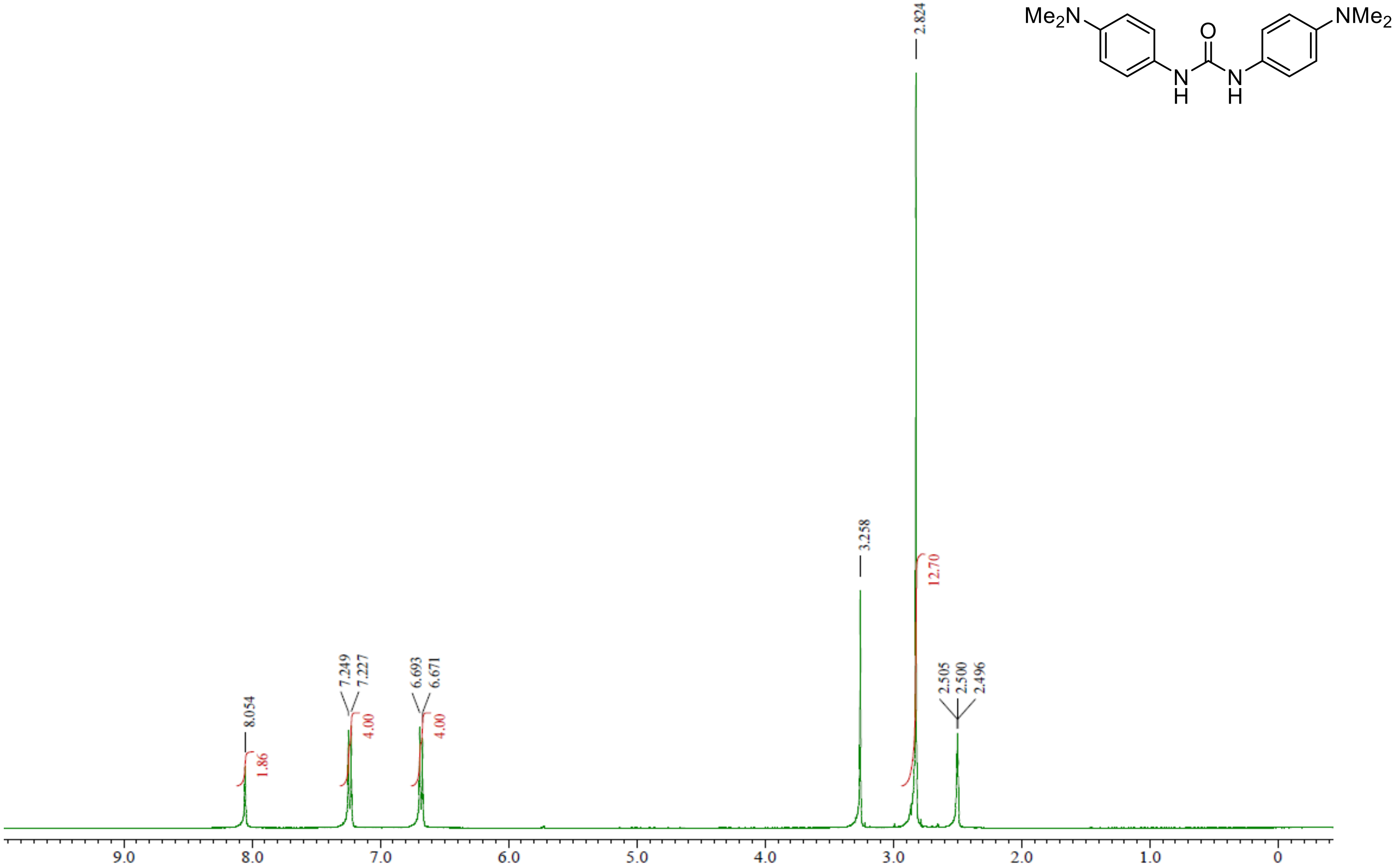

^{13}C NMR spectrum (101 MHz, DMSO-d_6) of 1,3-bis(4-dimethylaminophenyl)urea (**1i**).

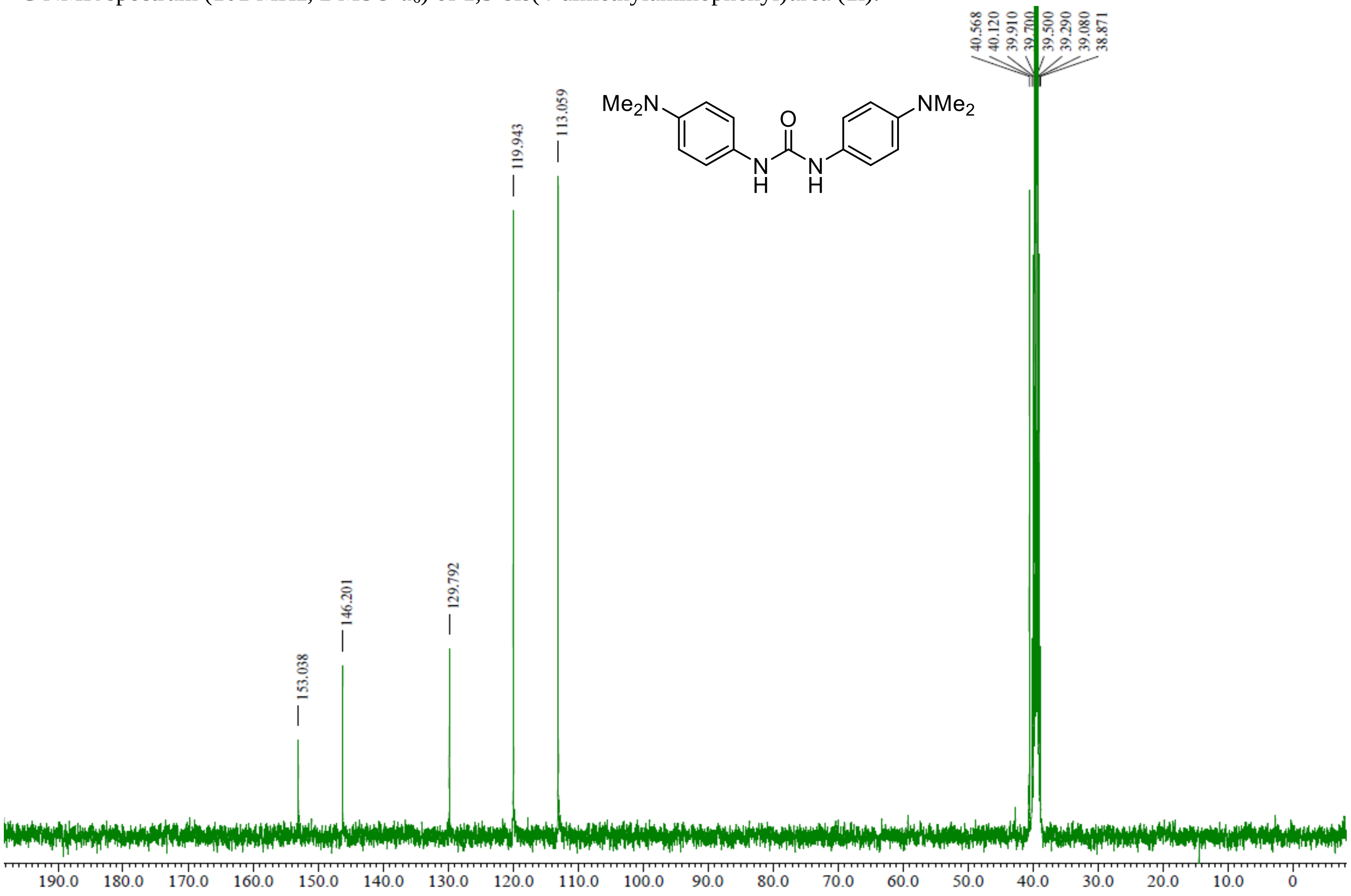

^{1}H NMR spectrum (400 MHz, DMSO-d_6) of diethyl 4,4'-(carbonylbis(azanediyl))dibenzoate (**1j**).

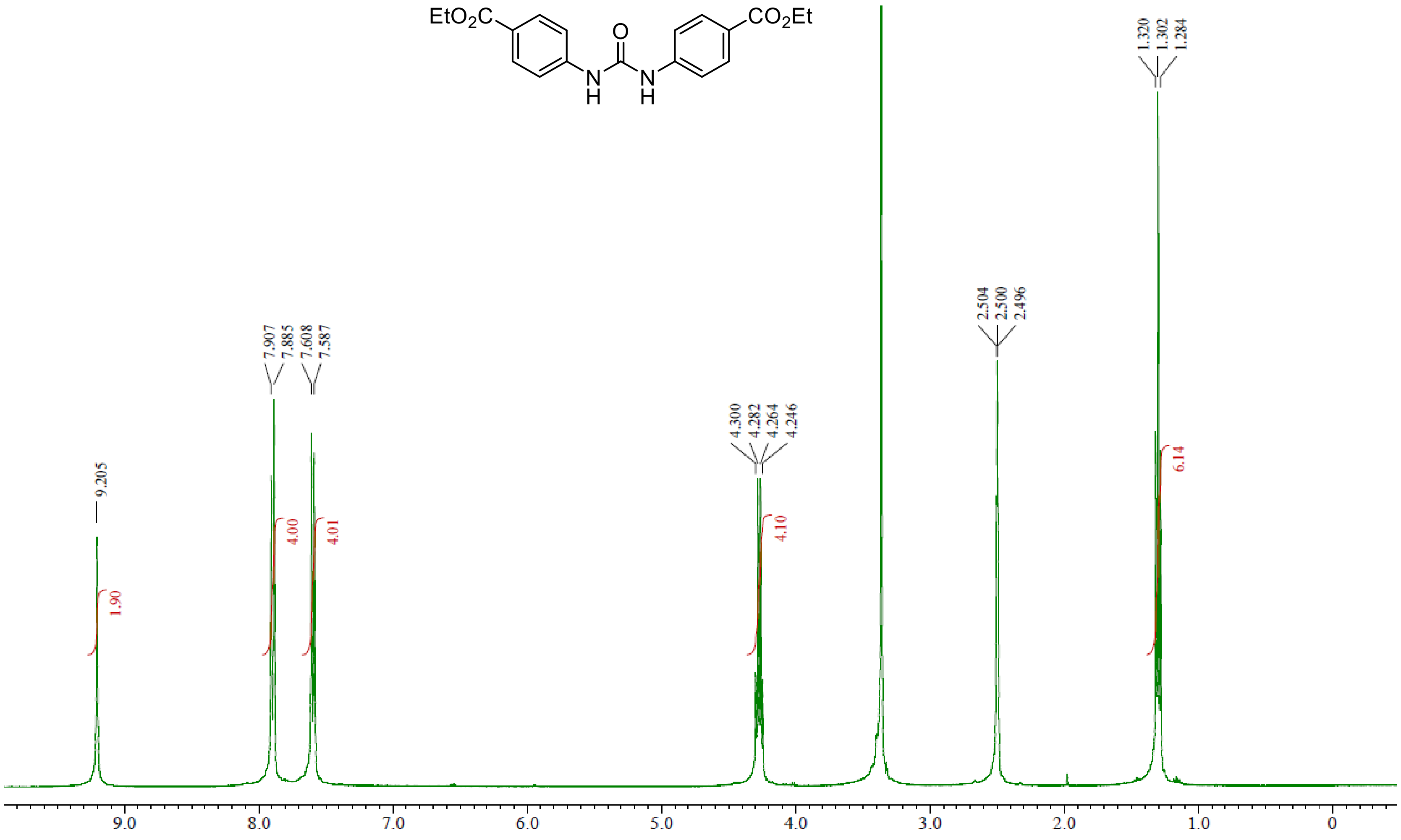

^{13}C NMR spectrum (101 MHz, DMSO-d_6) of diethyl 4,4'-(carbonylbis(azanediyl))dibenzoate (**1j**).

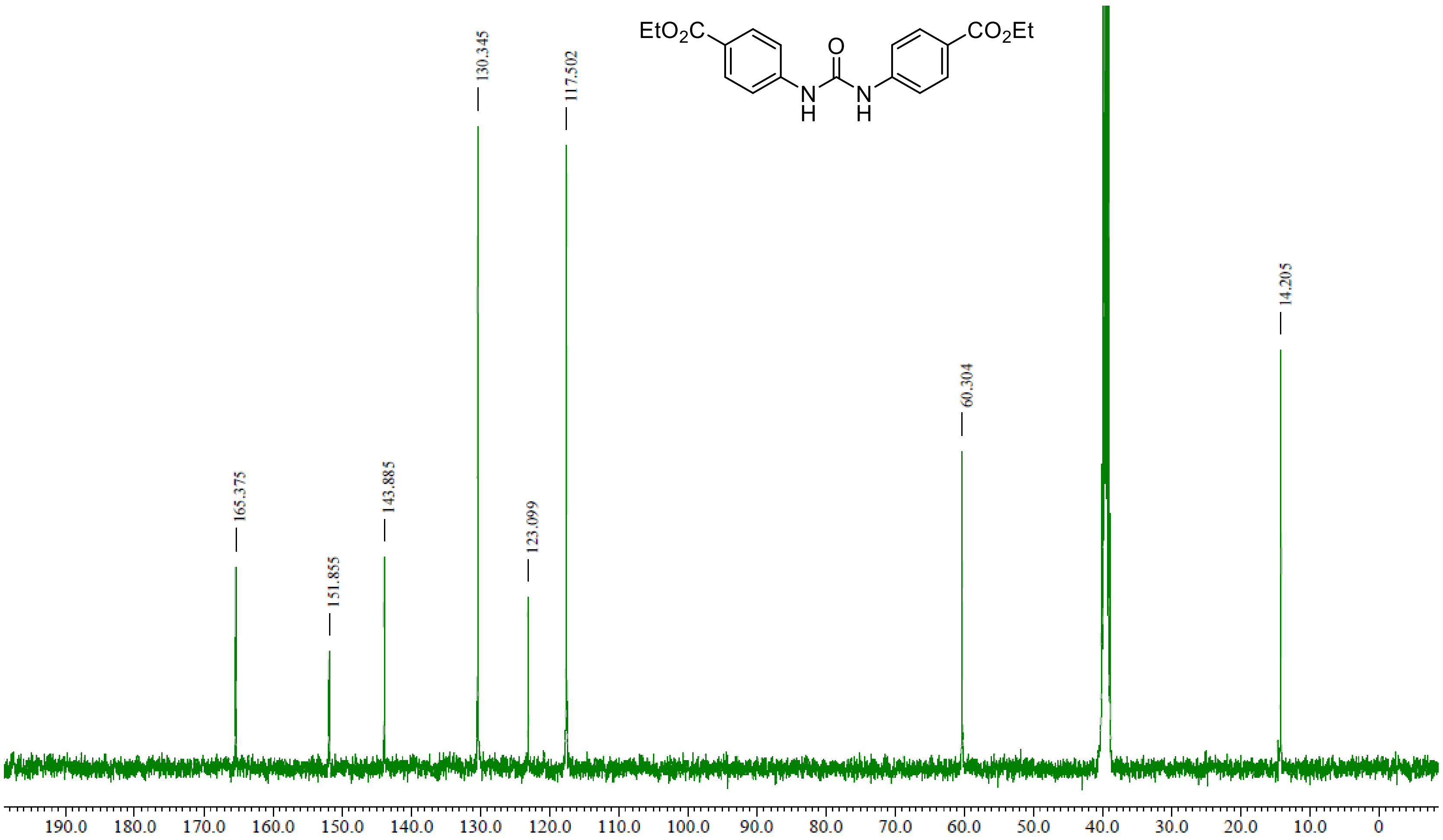

^{1}H NMR spectrum (400 MHz, DMSO-d_6) of 4,4'-(carbonylbis(azanediyl))bis(*N*,*N*-dipropylbenzamide) (**1k**).

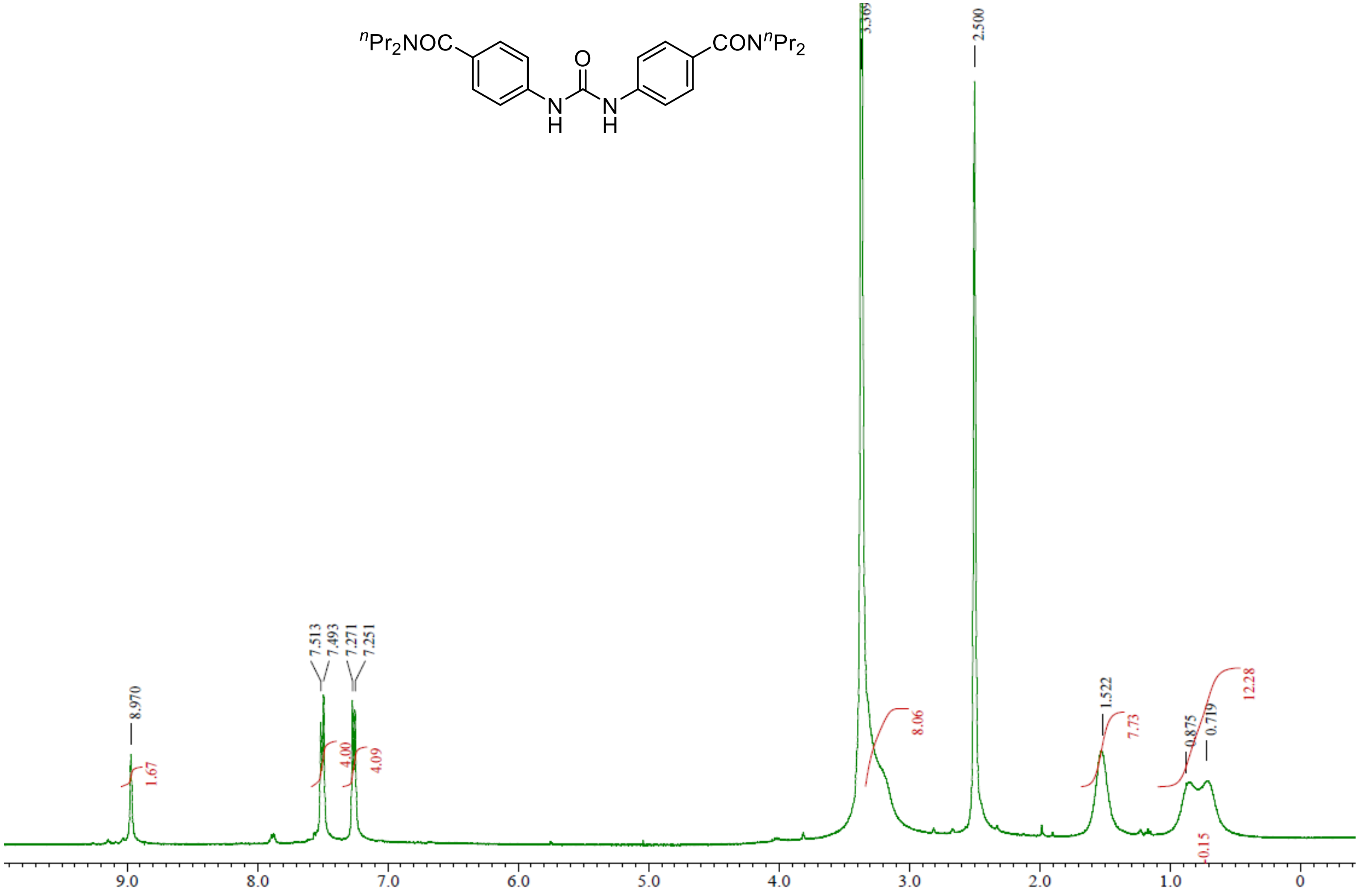

^{13}C NMR spectrum (101 MHz, DMSO-d_6) of 4,4'-(carbonylbis(azanediyl))bis(*N*,*N*-dipropylbenzamide) (**1k**).

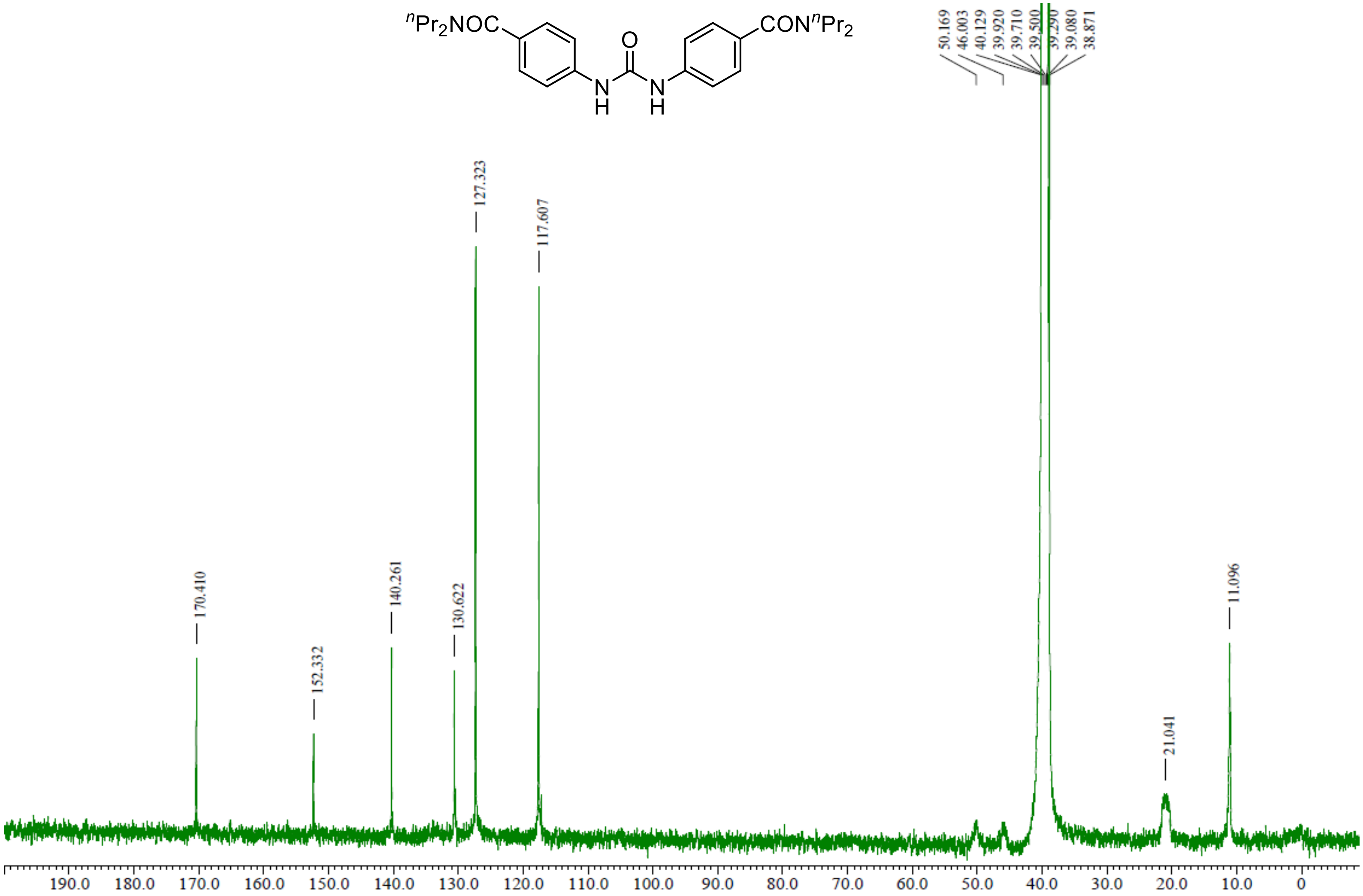

^{1}H NMR spectrum (400 MHz, DMSO-d_6) of 1,3-bis(4-cyanophenyl)urea (**1*l***).

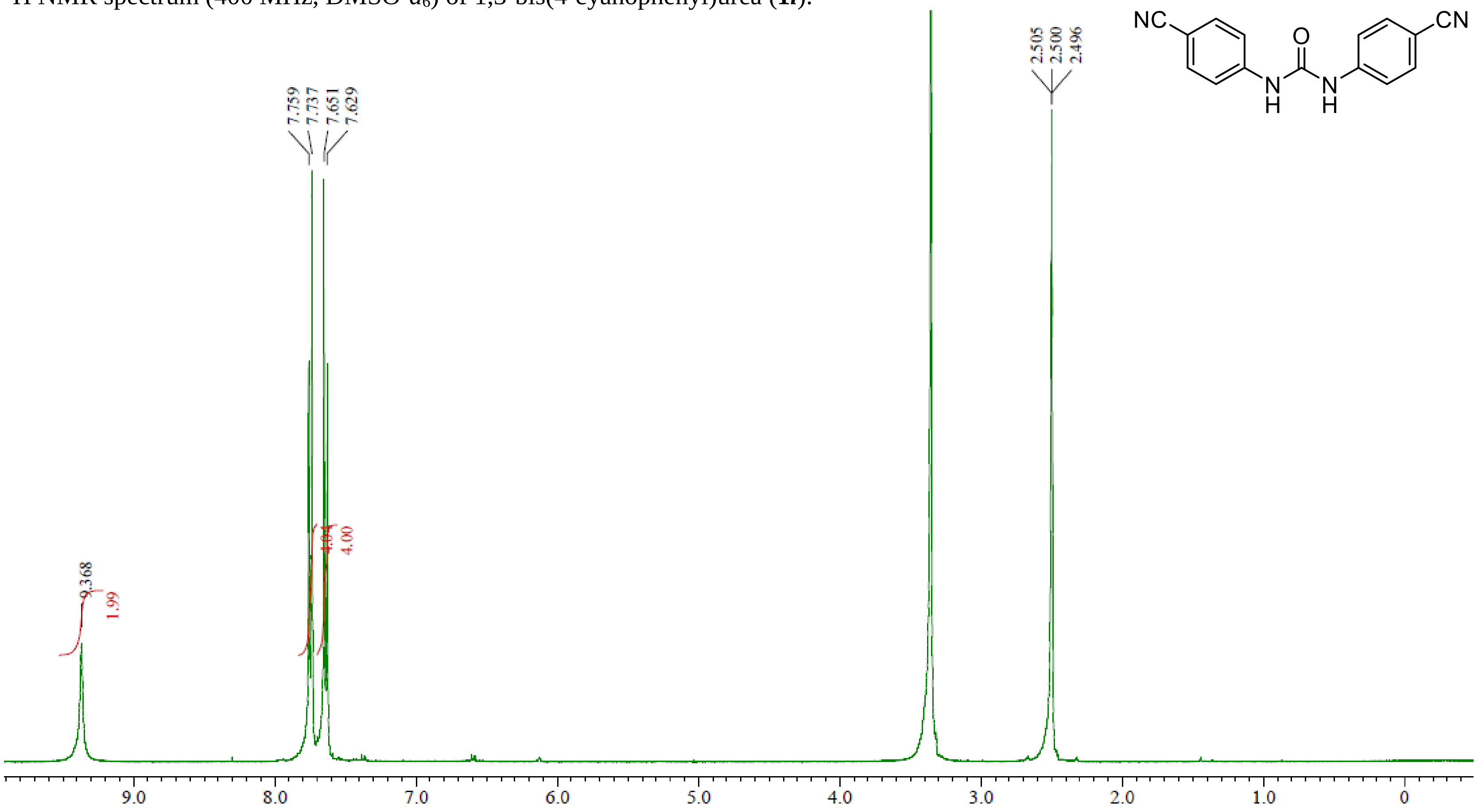

^{13}C NMR spectrum (101 MHz, DMSO-d_6) of 1,3-bis(4-cyanophenyl)urea (**1*l***).

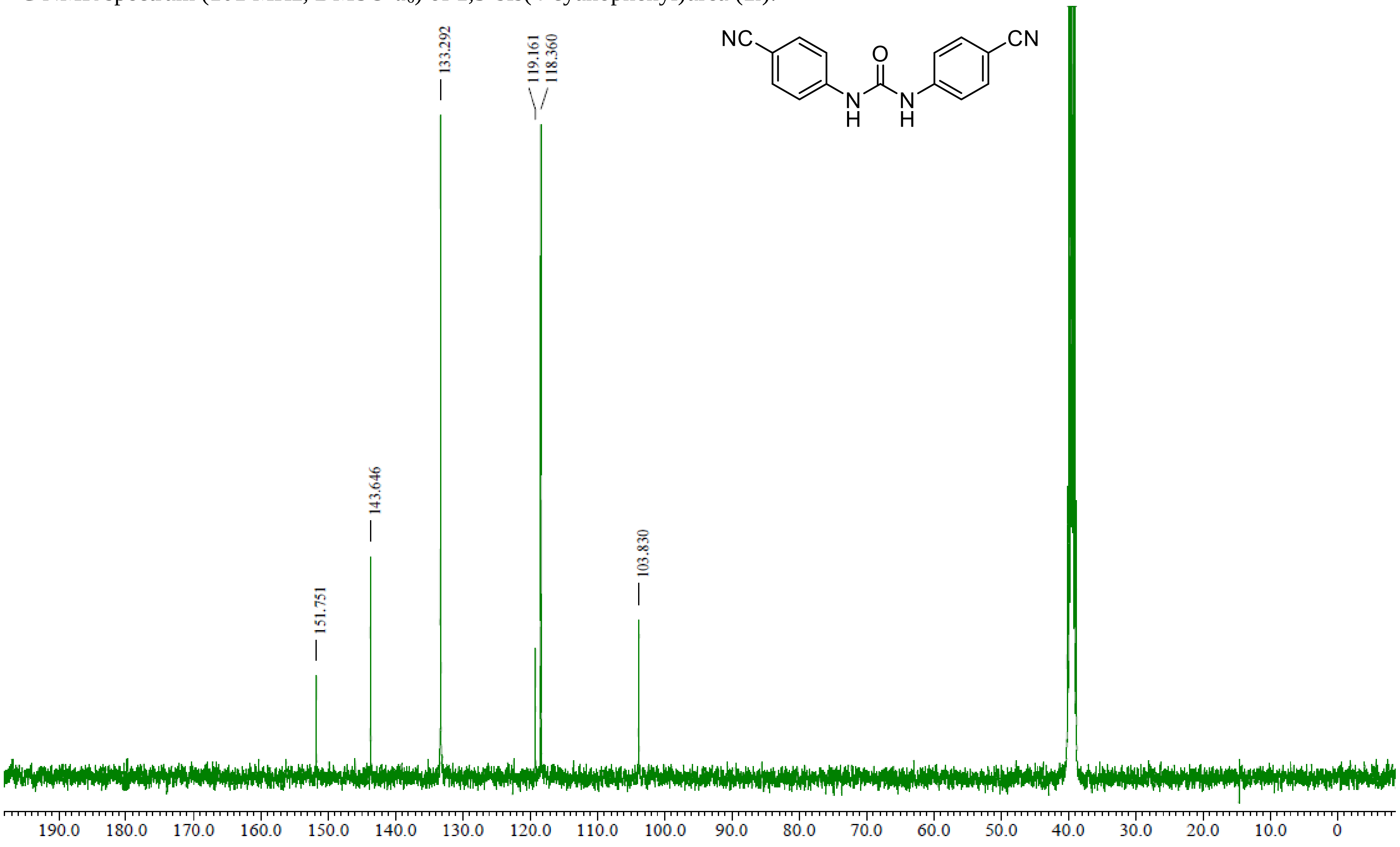

^{1}H NMR spectrum (400 MHz, DMSO-d_6) of 1-methyl-1,3-diphenylurea (**1ao**).

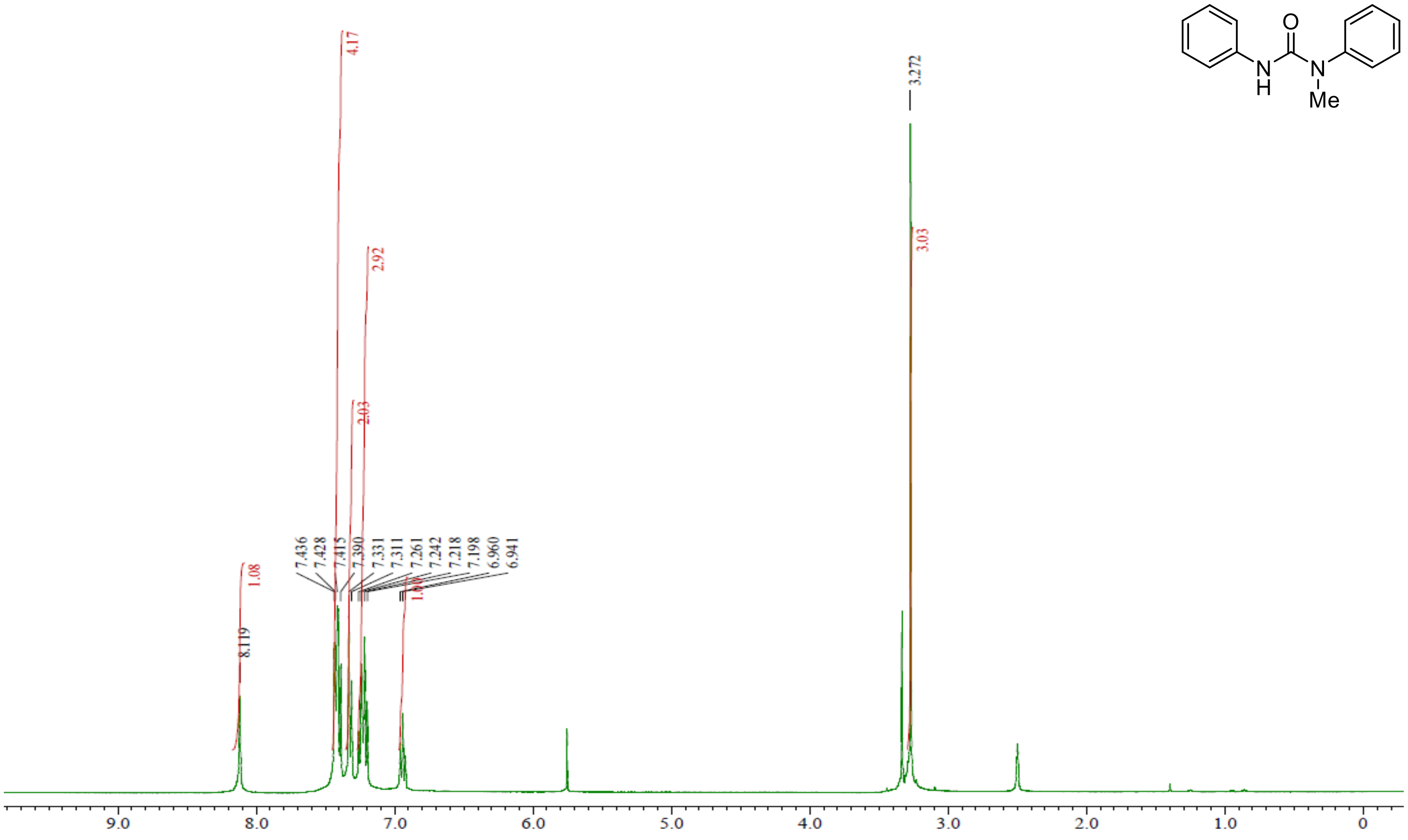

^{13}C NMR spectrum (101 MHz, DMSO-d_6) of 1-methyl-1,3-diphenylurea (**1ao**).

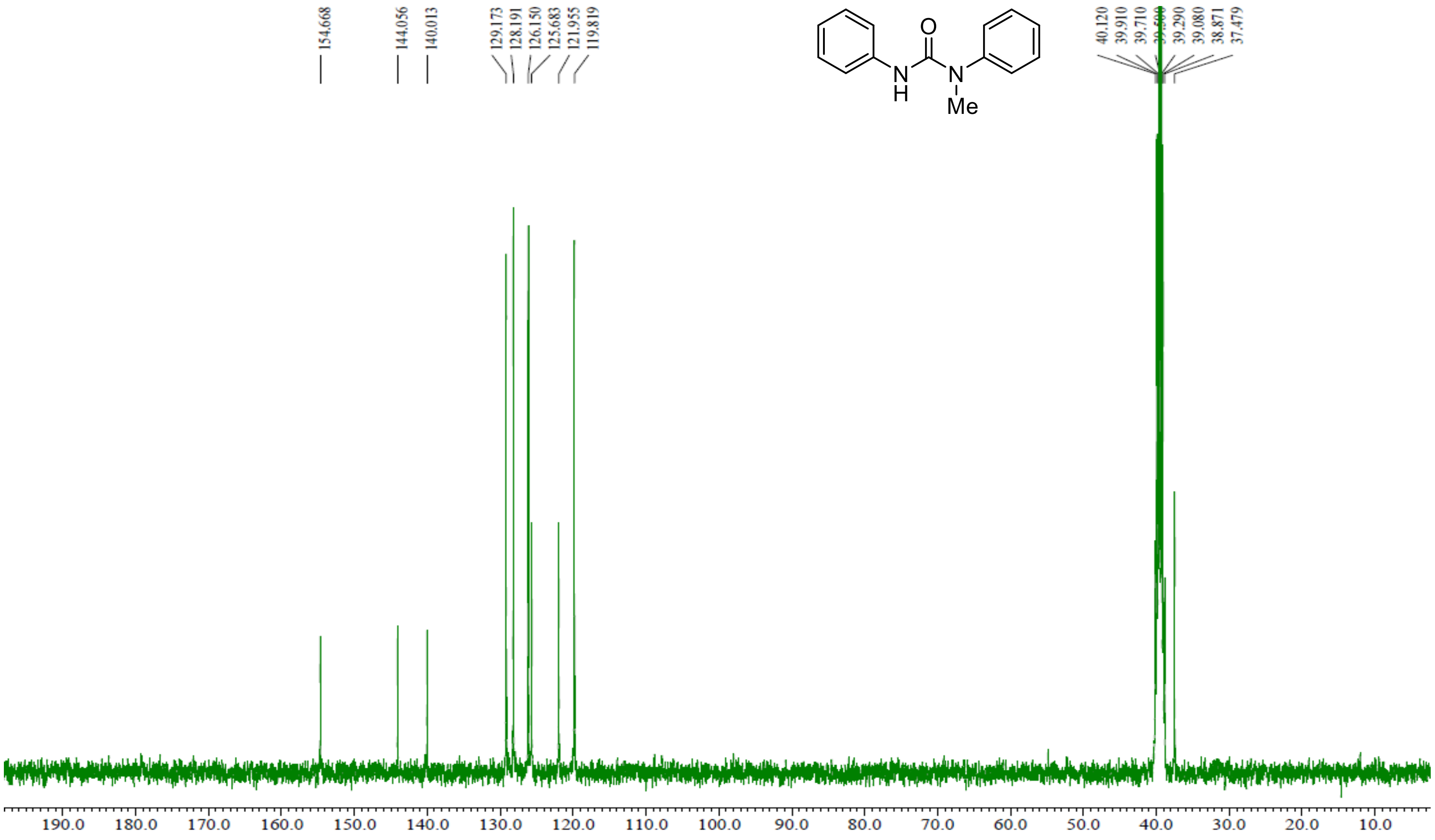

^{1}H NMR spectrum (400 MHz, $CDCl_3$) of 1-(4-chlorophenyl)-1-methyl-3-phenylurea (**1ap**).

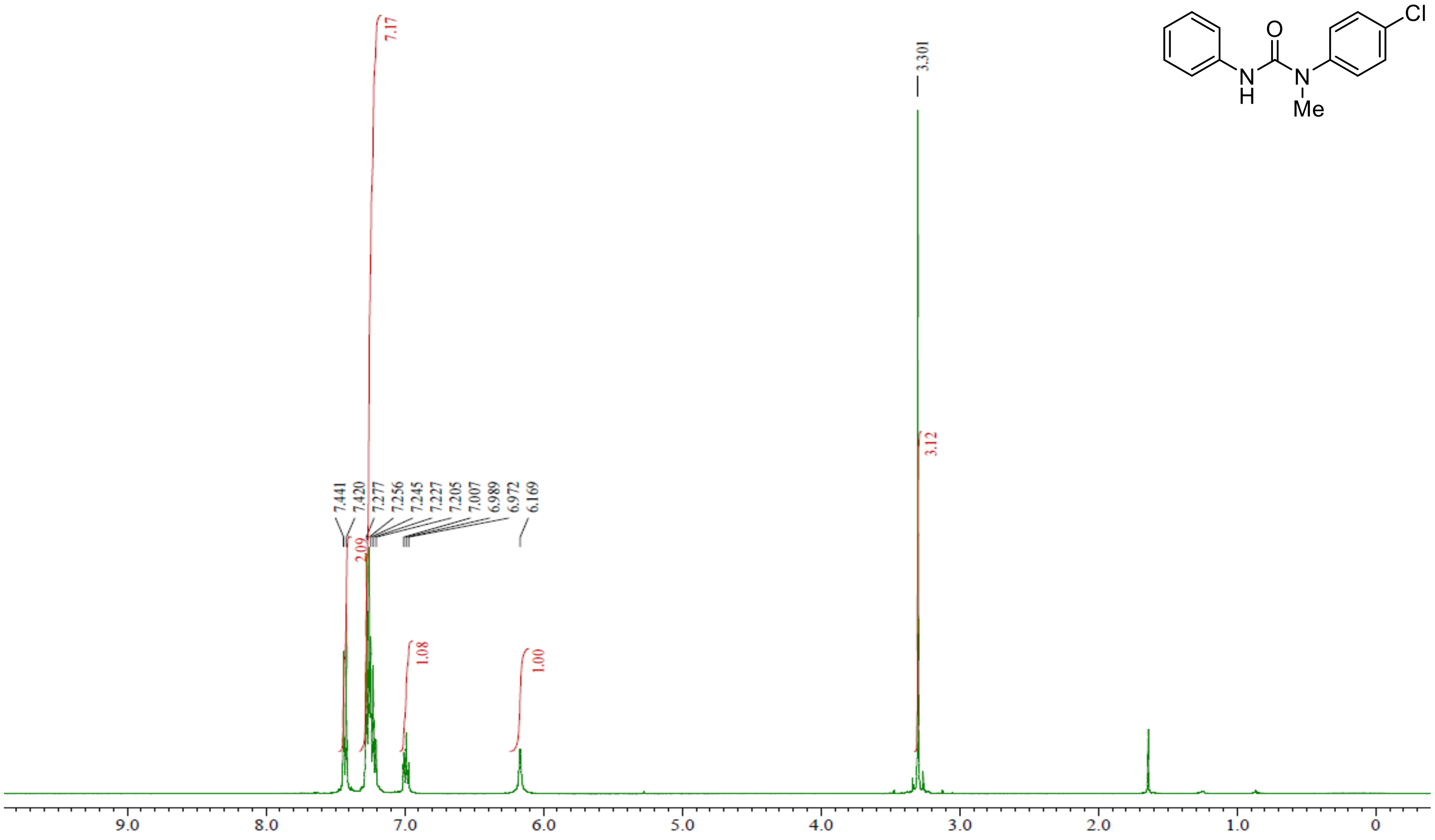

^{13}C NMR spectrum (101 MHz, $CDCl_3$) of 1-(4-chlorophenyl)-1-methyl-3-phenylurea (**1ap**).

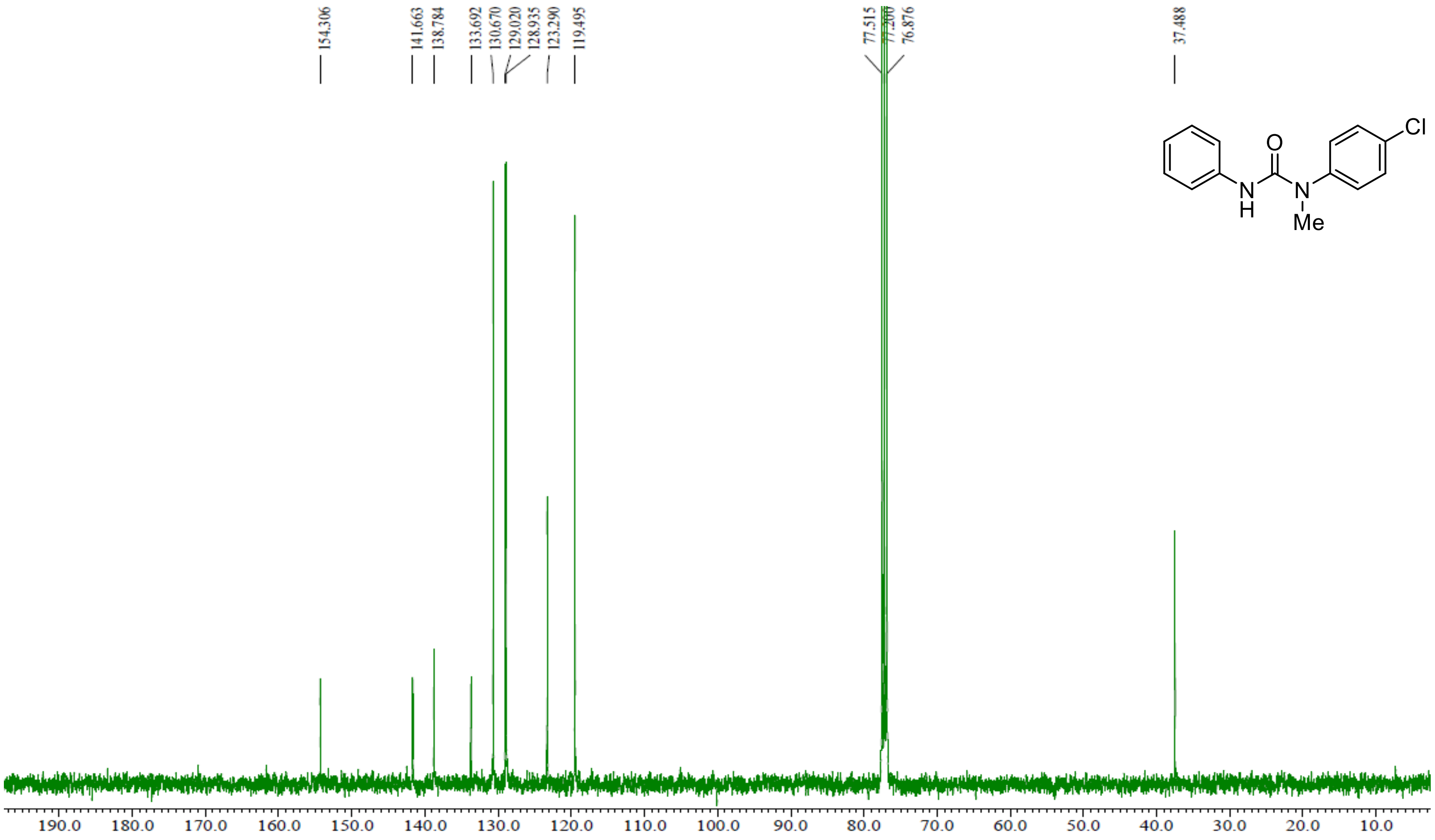

^{1}H NMR spectrum (400 MHz, $CDCl_3$) of 1,3-bis(4-chlorophenyl)-1-methylurea (**1cp**).

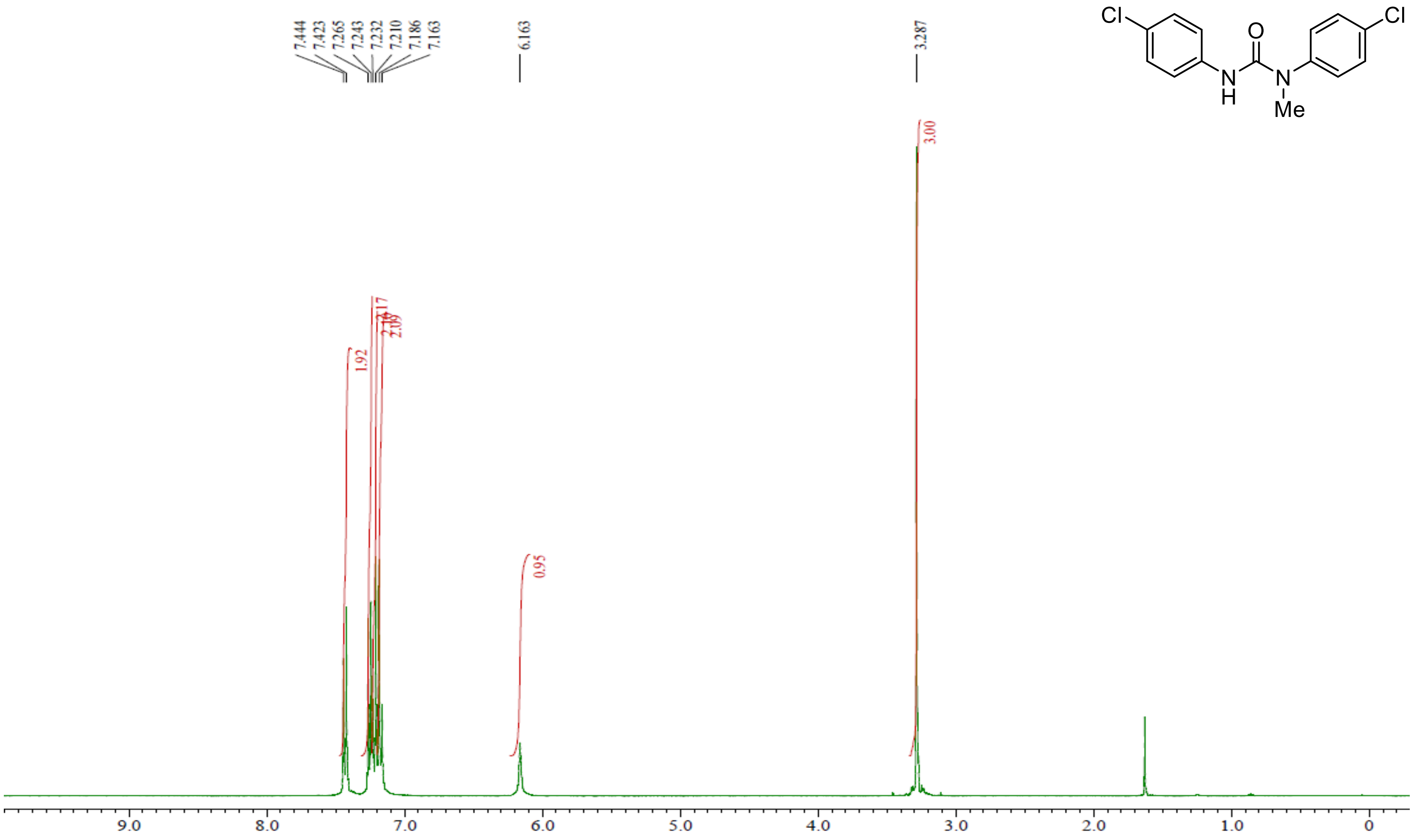

^{13}C NMR spectrum (101 MHz, $CDCl_3$) of 1,3-bis(4-chlorophenyl)-1-methylurea (**1cp**).

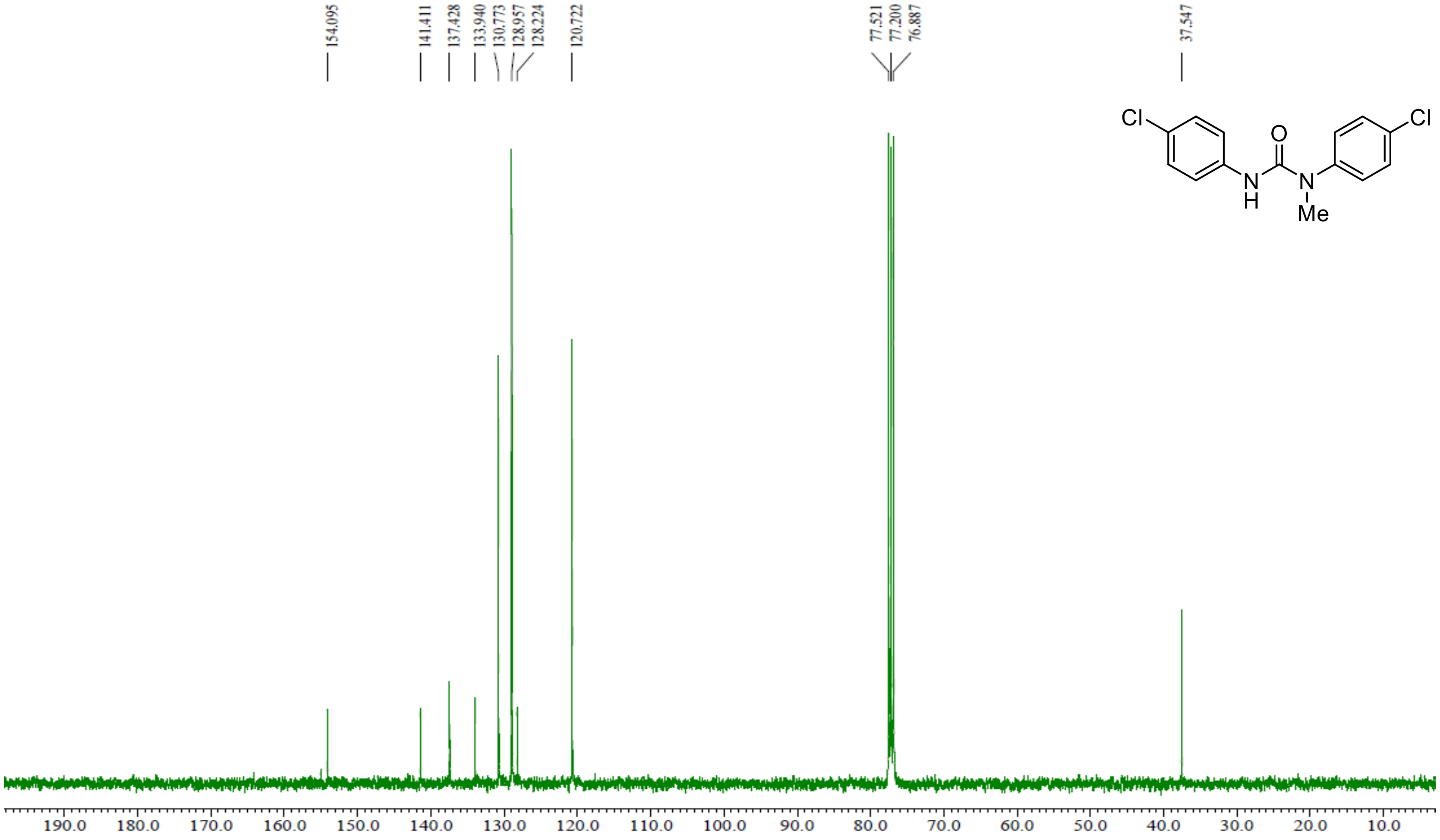

^{1}H NMR spectrum (400 MHz, $CDCl_3$) of 3-(4-butylphenyl)-1-(4-chlorophenyl)-1-methylurea (**1qp**).

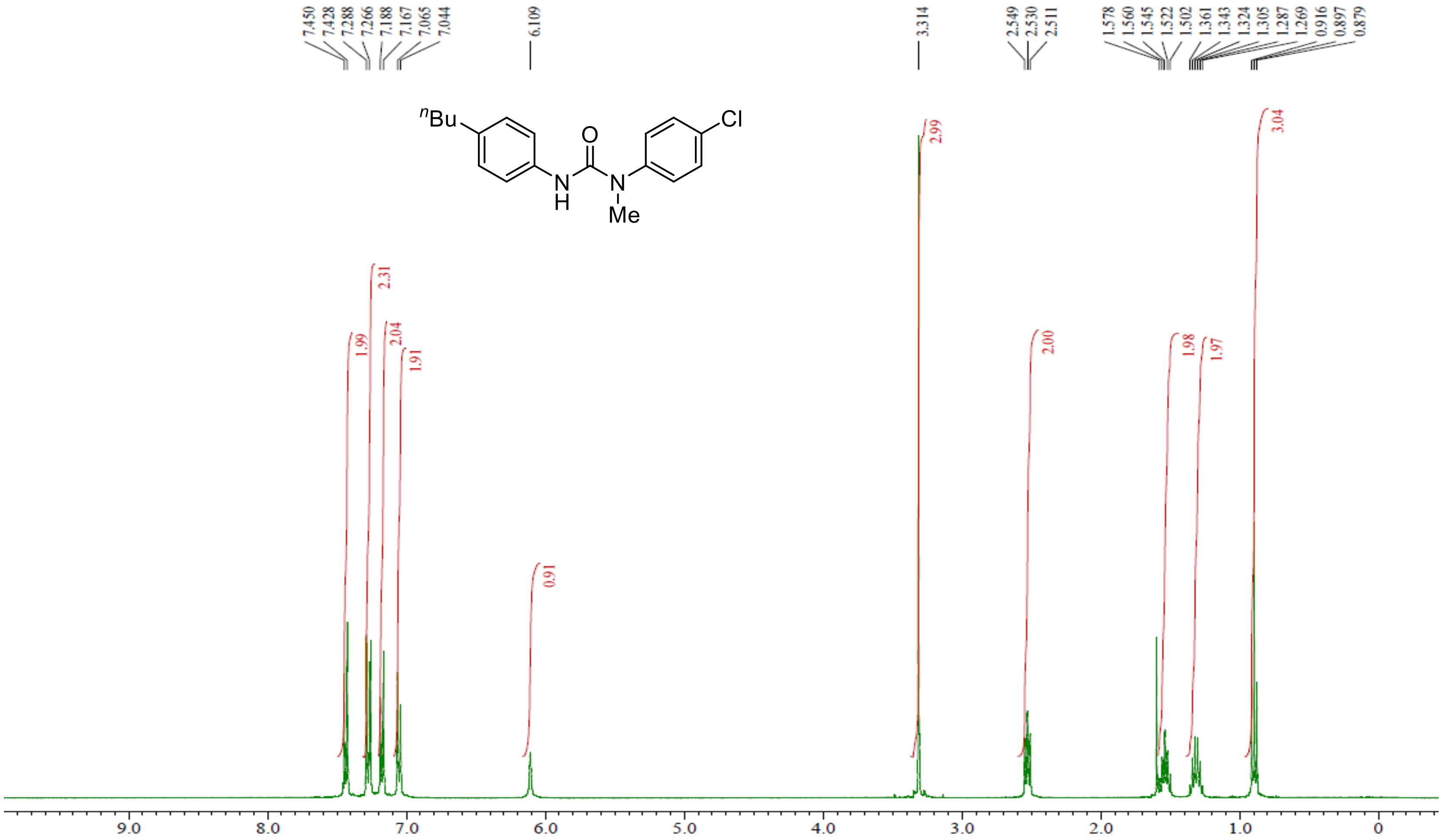

^{13}C NMR spectrum (101 MHz, $CDCl_3$) of 3-(4-butylphenyl)-1-(4-chlorophenyl)-1-methylurea (**1qp**).

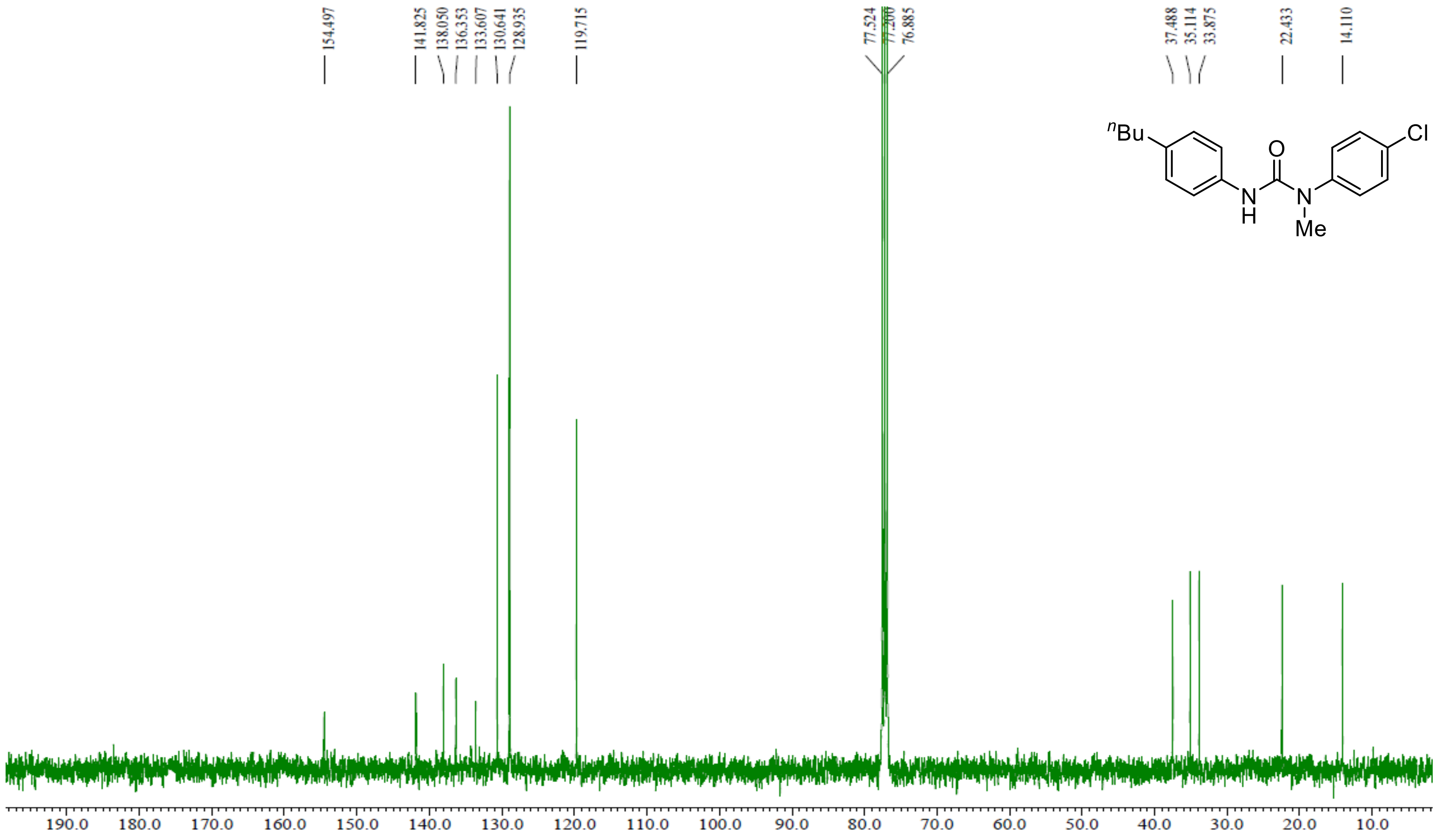

^{1}H NMR spectrum (400 MHz, $CDCl_3$) of 3-(4-chlorophenyl)-1-(4-methoxylphenyl)-1-methylurea (**1cr**).

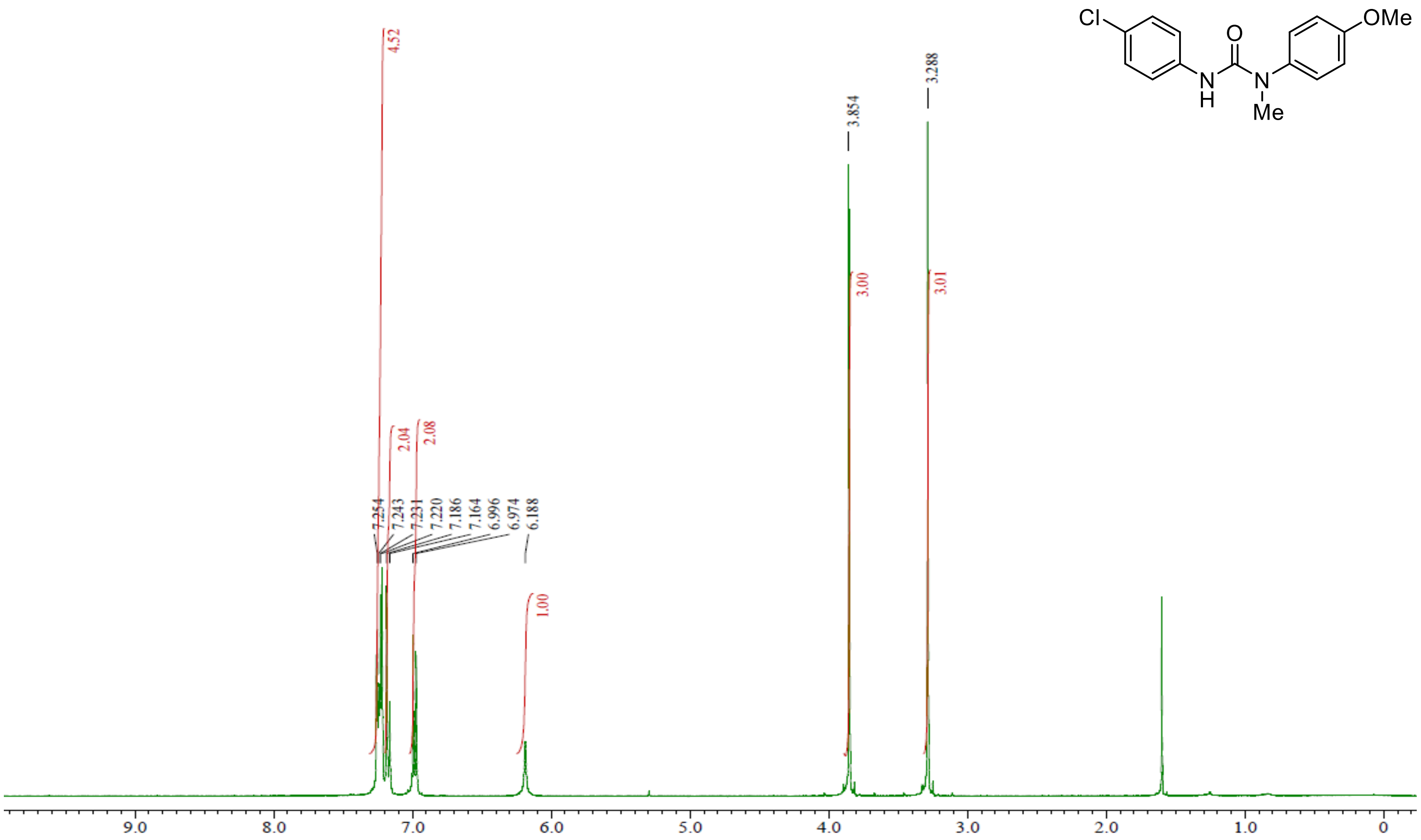

^{13}C NMR spectrum (101 MHz, $CDCl_3$) of 3-(4-chlorophenyl)-1-(4-methoxylphenyl)-1-methylurea (**1cr**).

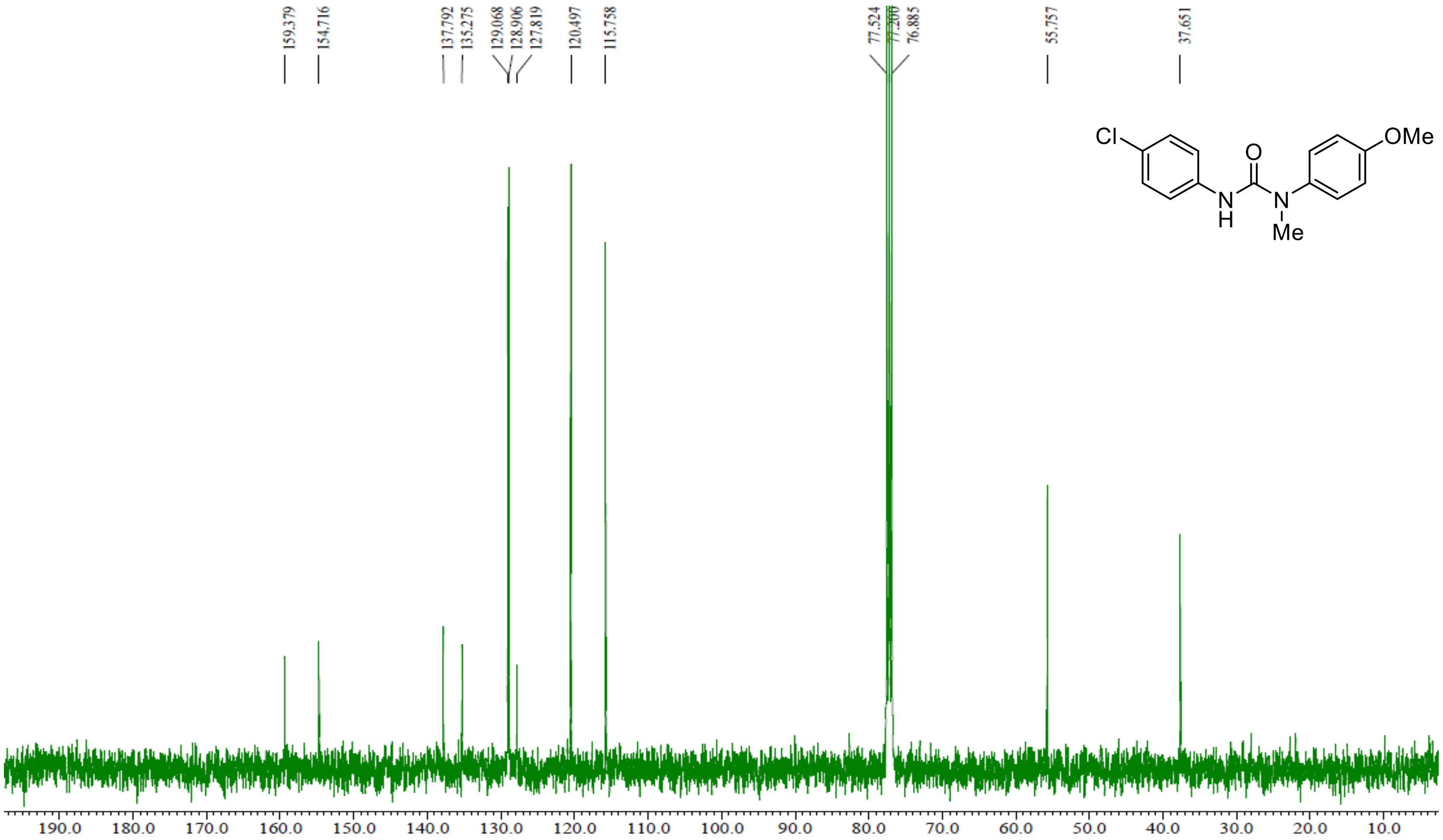

^{1}H NMR spectrum (400 MHz, $CDCl_3$) of 3-(4-butylphenyl)-1-(4-methoxylphenyl)-1-methylurea (**1qr**).

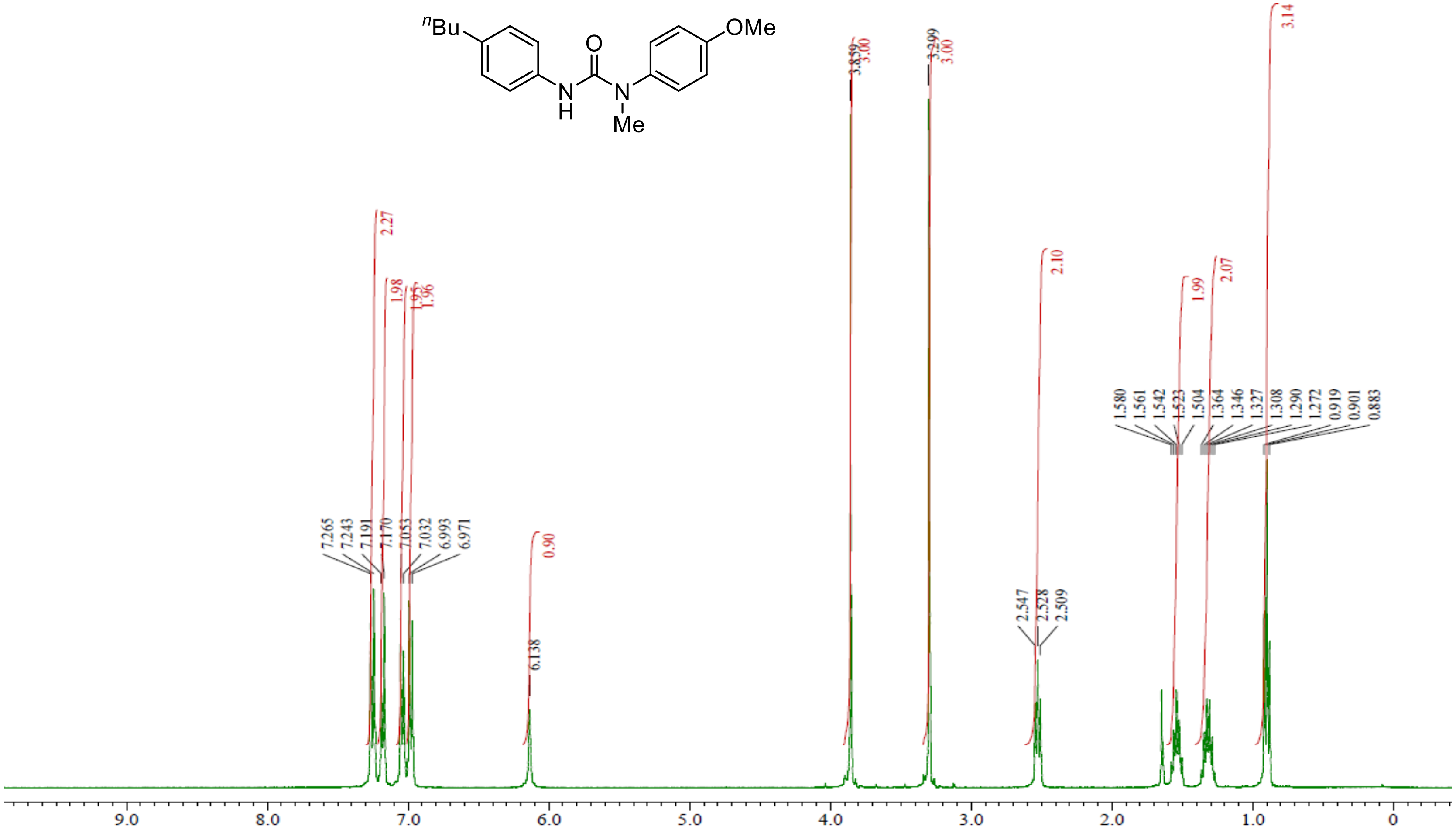

^{13}C NMR spectrum (101 MHz, $CDCl_3$) of 3-(4-butylphenyl)-1-(4-methoxylphenyl)-1-methylurea (**1qr**).

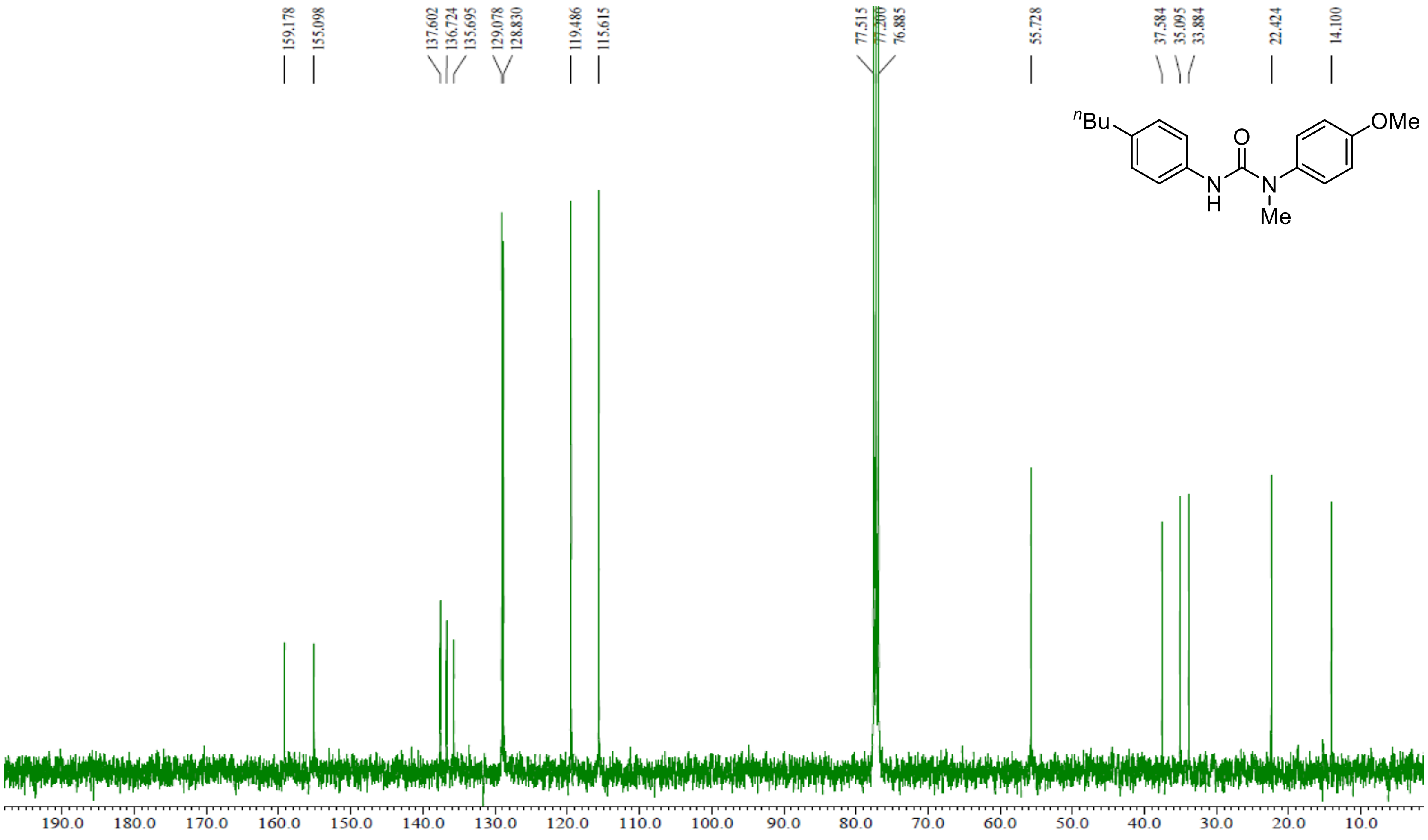

^{1}H NMR spectrum (400 MHz, DMSO-d_6) of *N*-phenylmorpholine-4-carboxamide (**1as**).

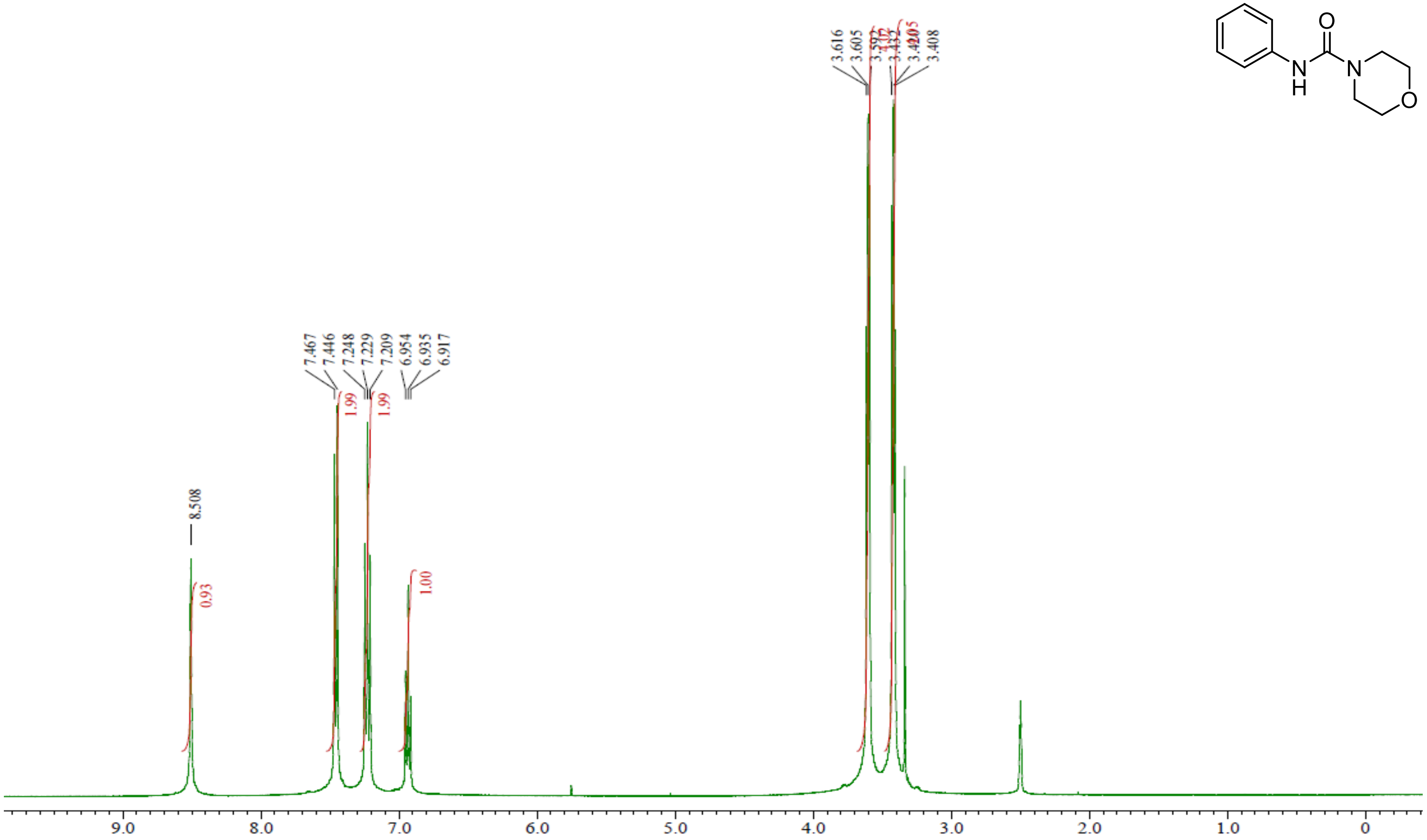

^{13}C NMR spectrum (101 MHz, DMSO-d_6) of *N*-phenylmorpholine-4-carboxamide (**1as**).

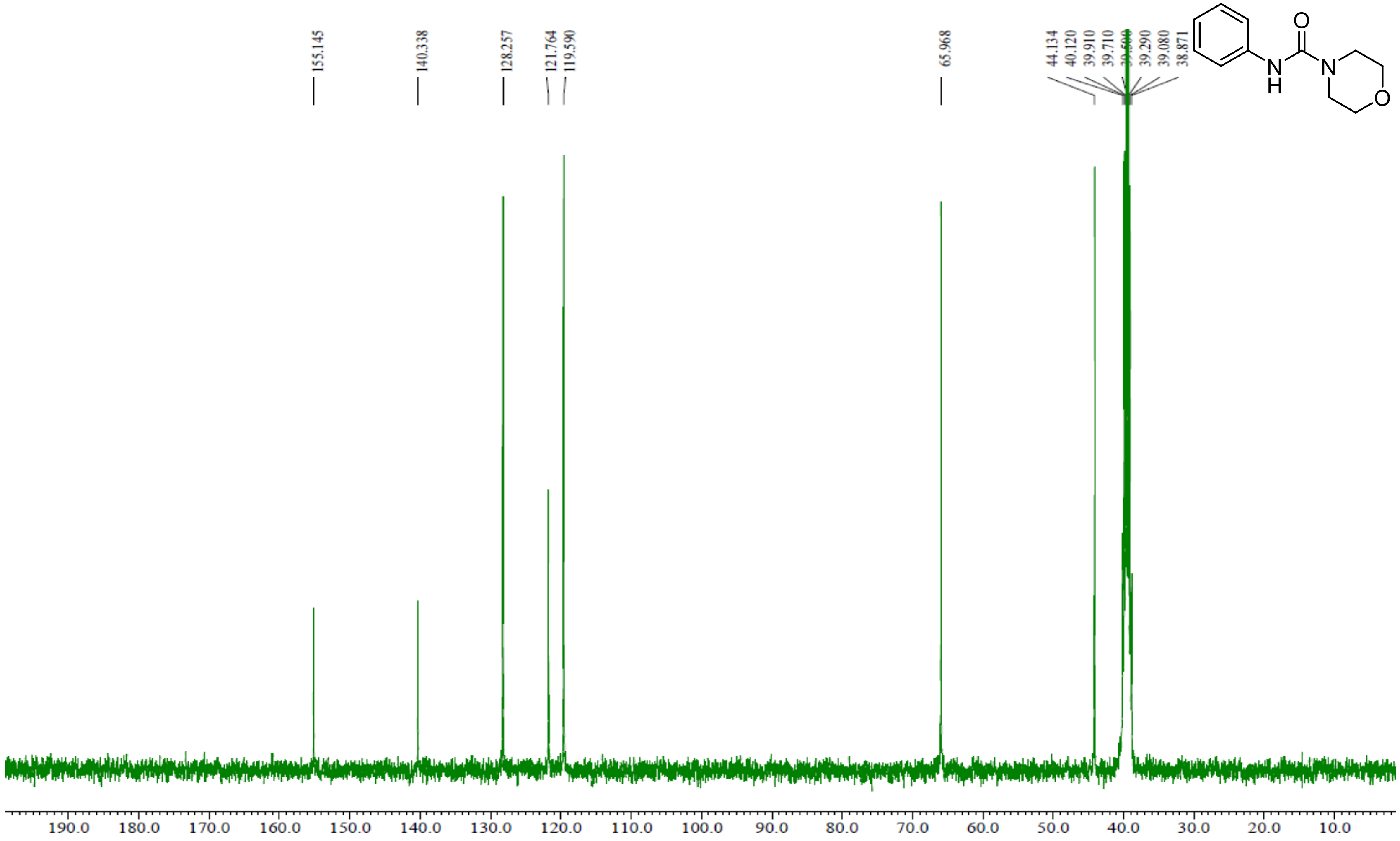

^{1}H NMR spectrum (500 MHz, DMSO-d_6) of polyurea resin **17** (× impurities).

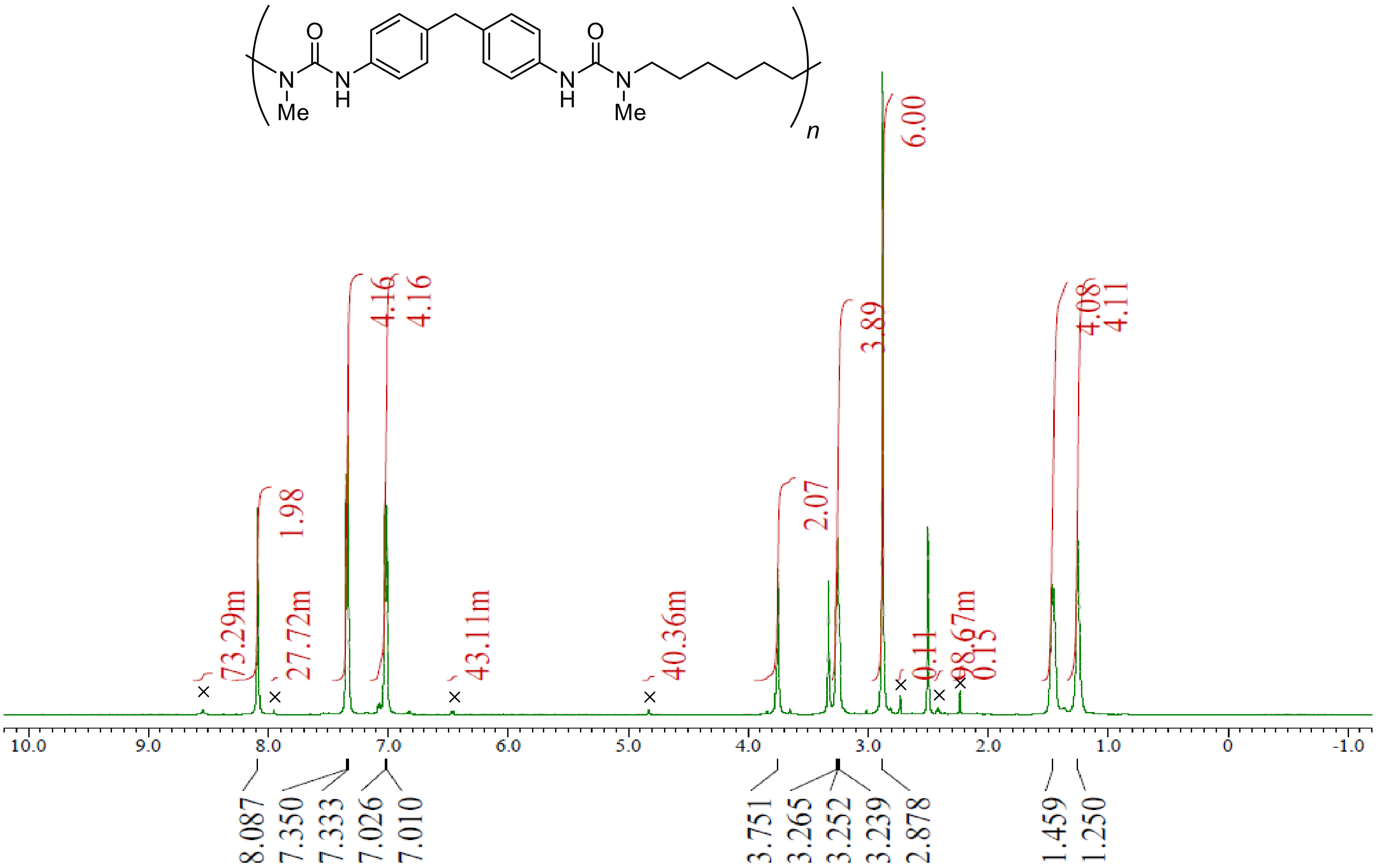

^{13}C NMR spectrum (126 MHz, DMSO-d_6) of polyurea resin **17** (× impurities).

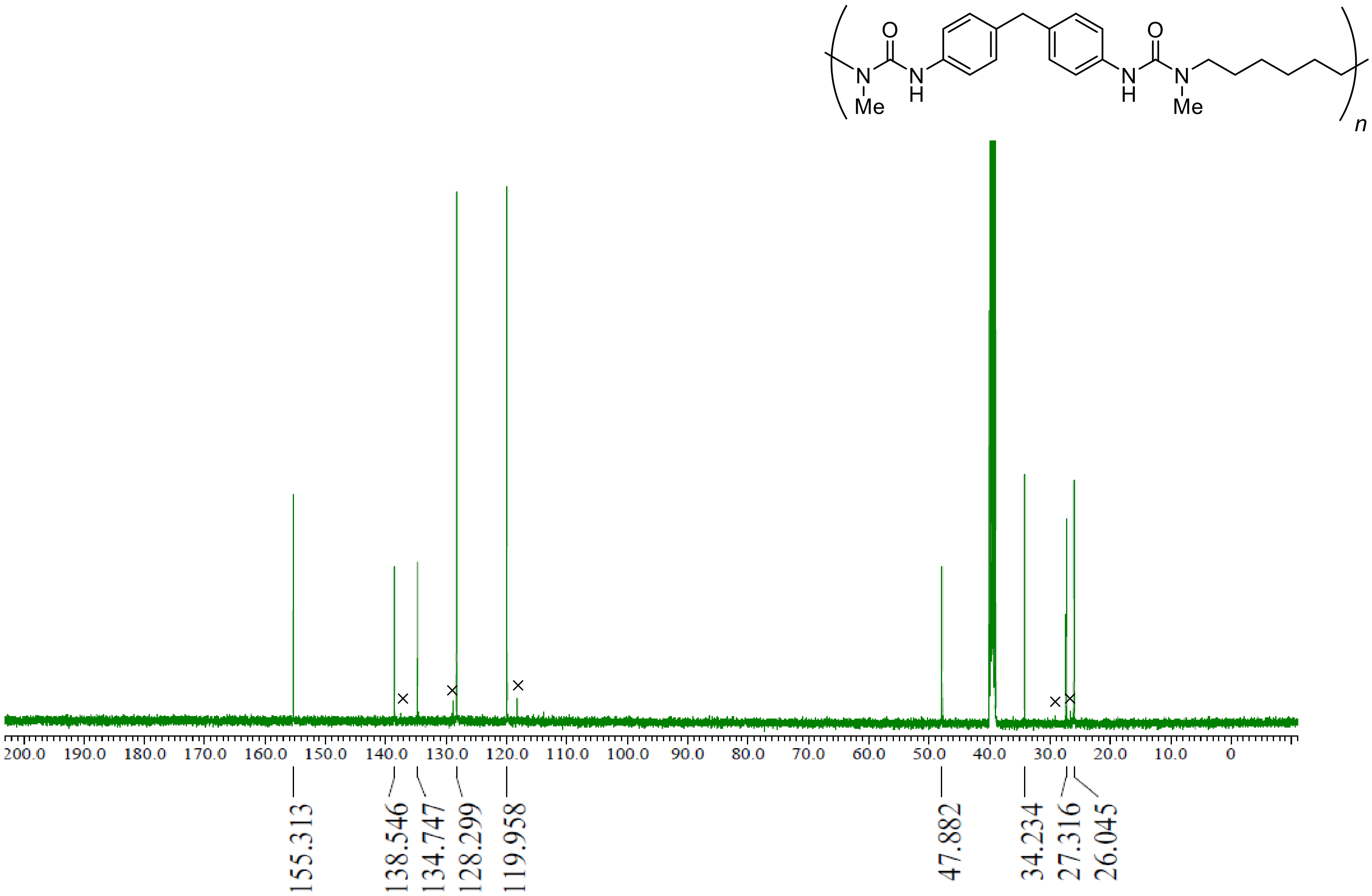

GPC chart of polyurea resin **17**

<Chromatogram>

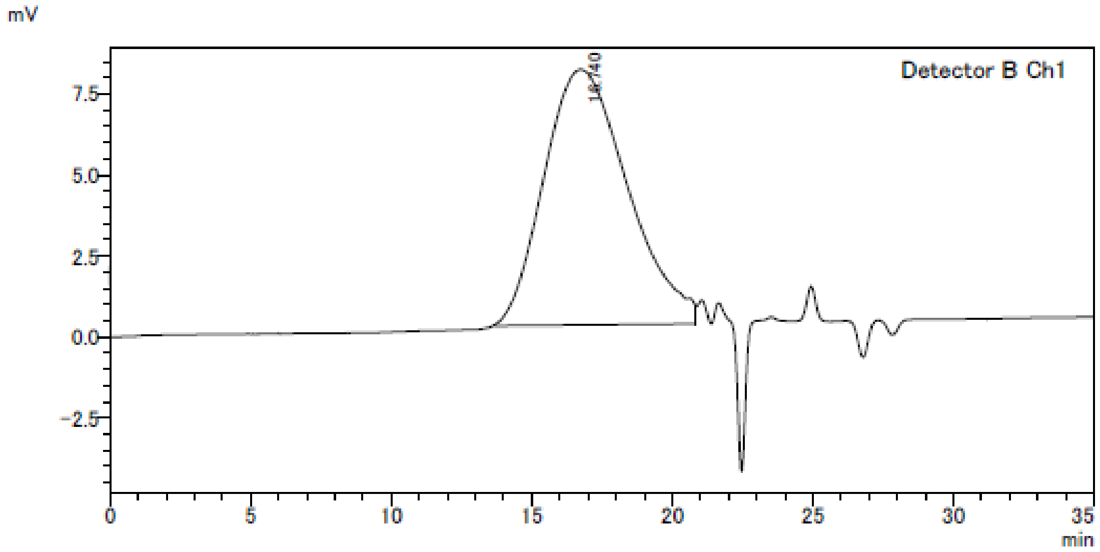

<Peak Report>

Detector B Ch1

Peak#	Retention Time	Area	Height	Concentration	Unit	Mark	Compound
1	16.740	1630520	7886	0.000		M	
Total		1630520	7886				

<GPC Results>

Peak#:1 (Detector B Ch1)
[Average Molecular Weight]

Number Average Molecular Weight(Mn)	64482
Weight Average Molecular Weight(Mw)	89152
Z Average Molecular Weight(Mz)	113471
Mw/Mn	1.38260
Mz/Mw	1.27277

^{1}H NMR spectrum (500 MHz, $CDCl_3$) of *N*,*N'*-(hexane-1,6-diyl)bis(*N*-methylformamide) (**21**). Multi isomers were observed.

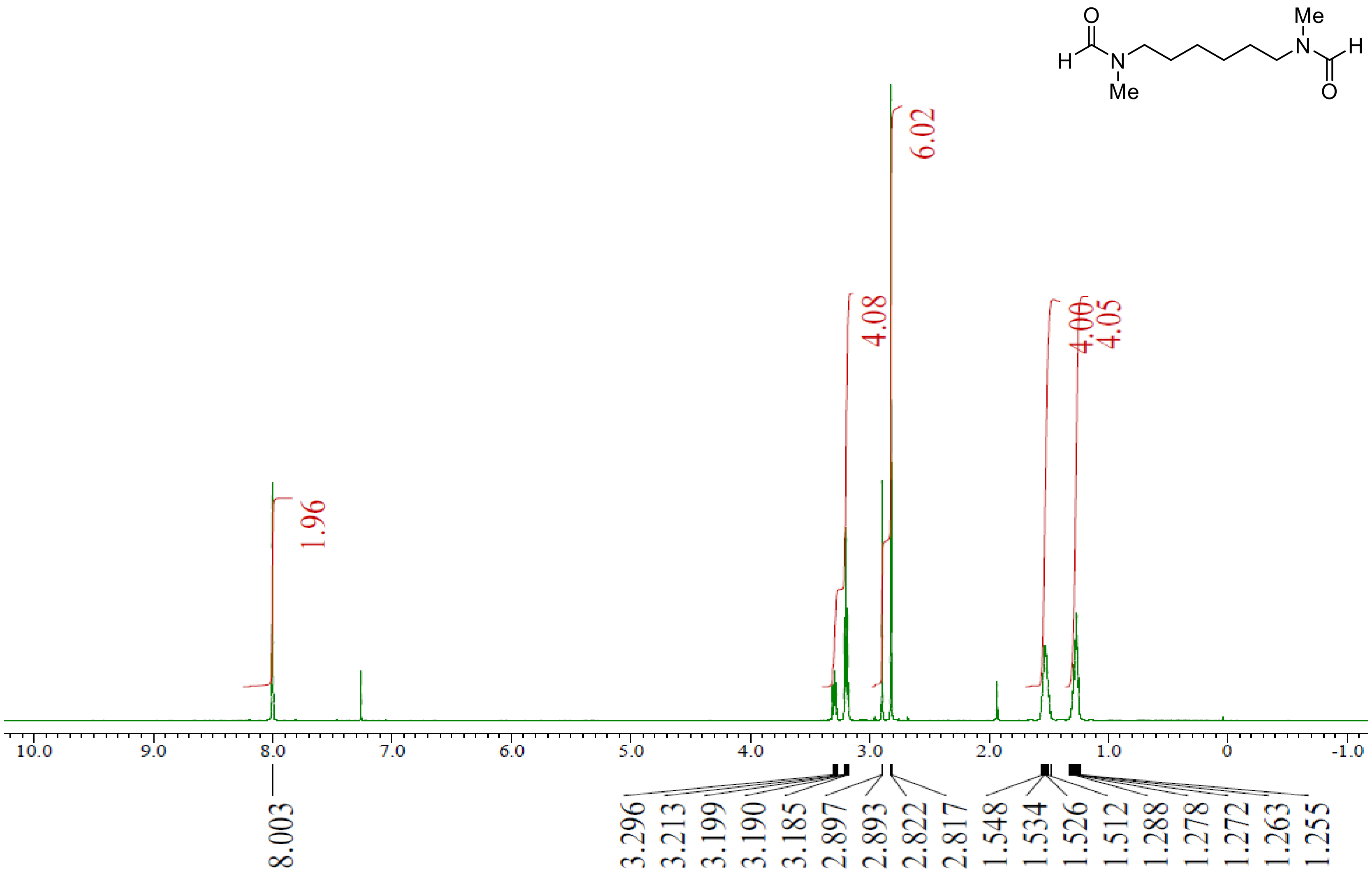

^{13}C NMR spectrum (126 MHz, $CDCl_3$) of *N*,*N'*-(hexane-1,6-diyl)bis(*N*-methylformamide) (**21**). Multi isomers were observed.

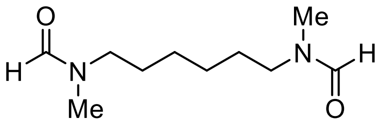

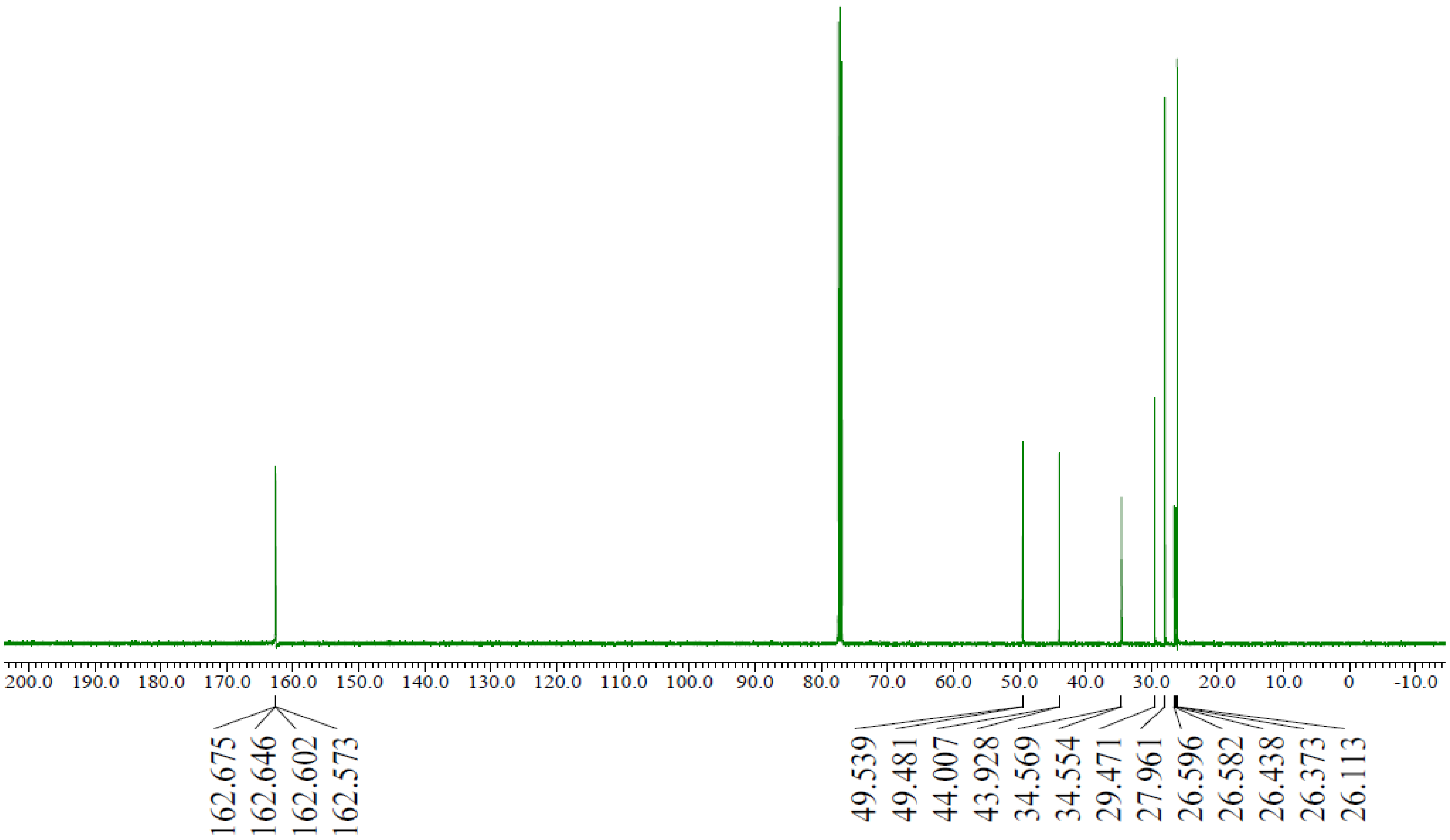

^{1}H NMR spectrum (500 MHz, $CDCl_3$) of *N*-methyl-*N*-(6-(methylamino)hexyl)formamide (**22**). Multi isomers were observed.

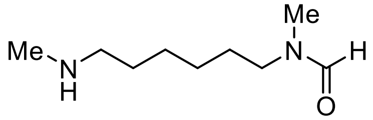

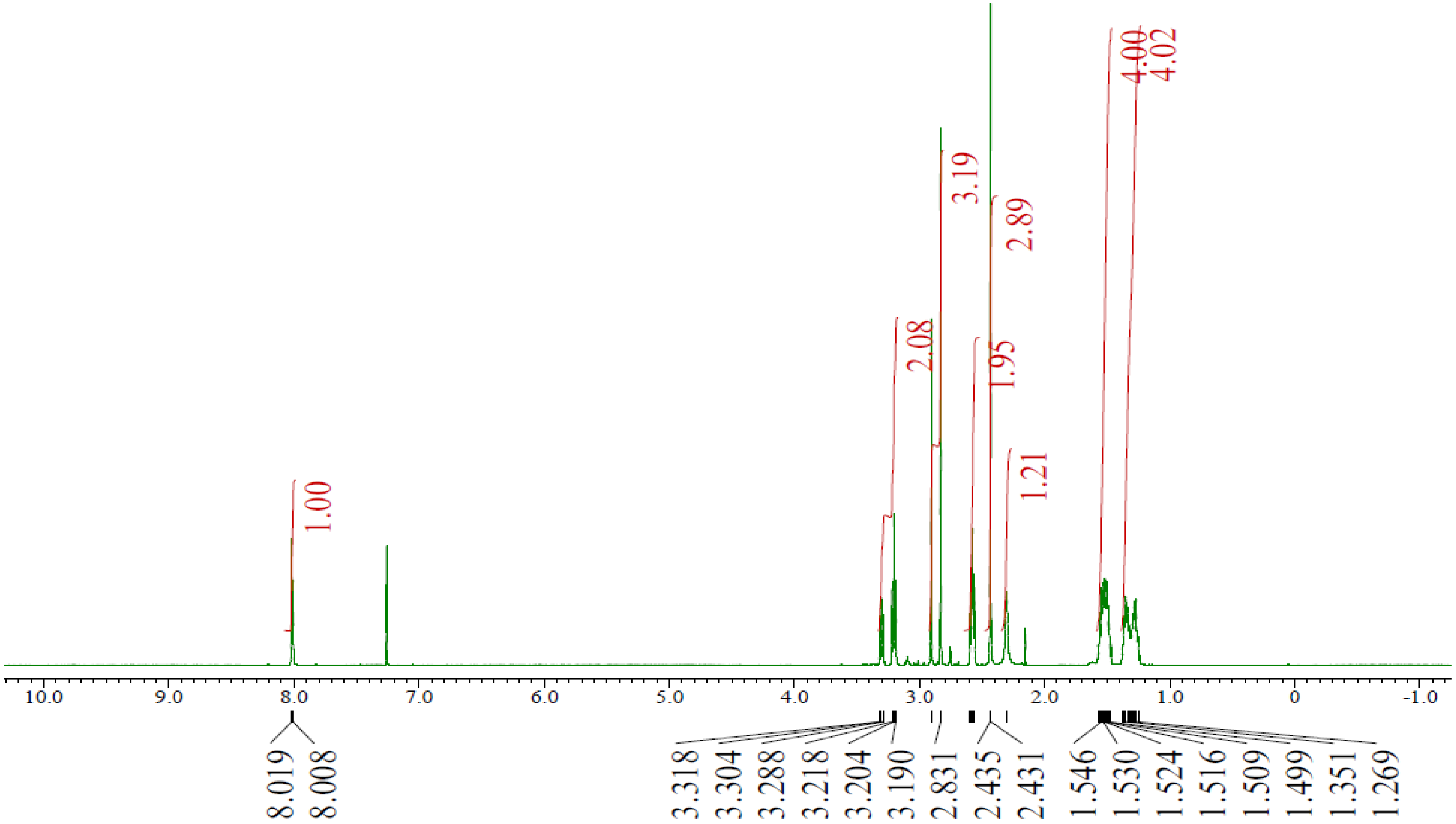

^{13}C NMR spectrum (126 MHz, $CDCl_3$) of *N*-methyl-*N*-(6-(methylamino)hexyl)formamide (**22**). Multi isomers were observed.

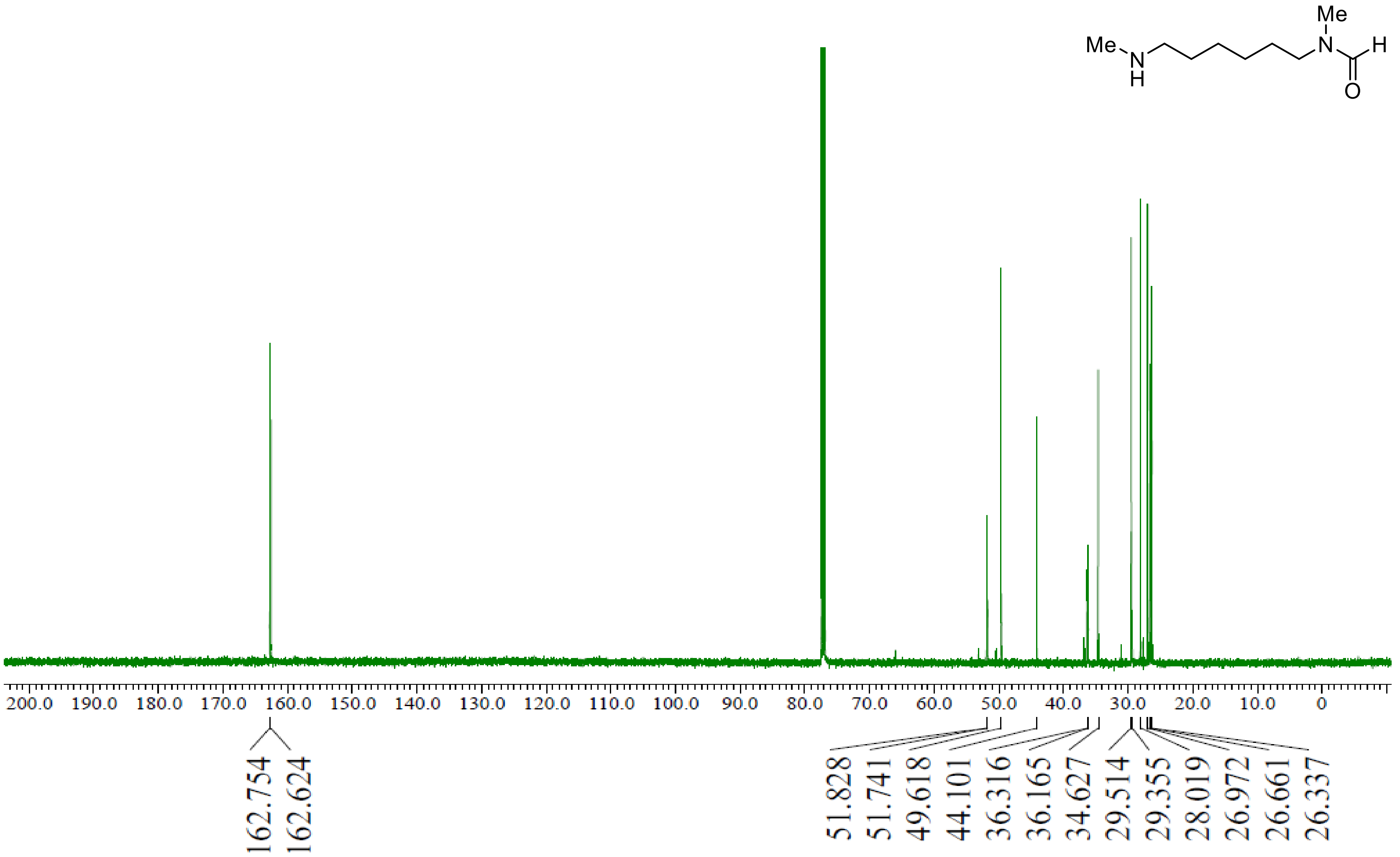

^{1}H NMR spectrum (500 MHz, $CDCl_3$) of formanilide (**2a**). Two tautomers were observed in approximately 0.5:0.5 ratio.

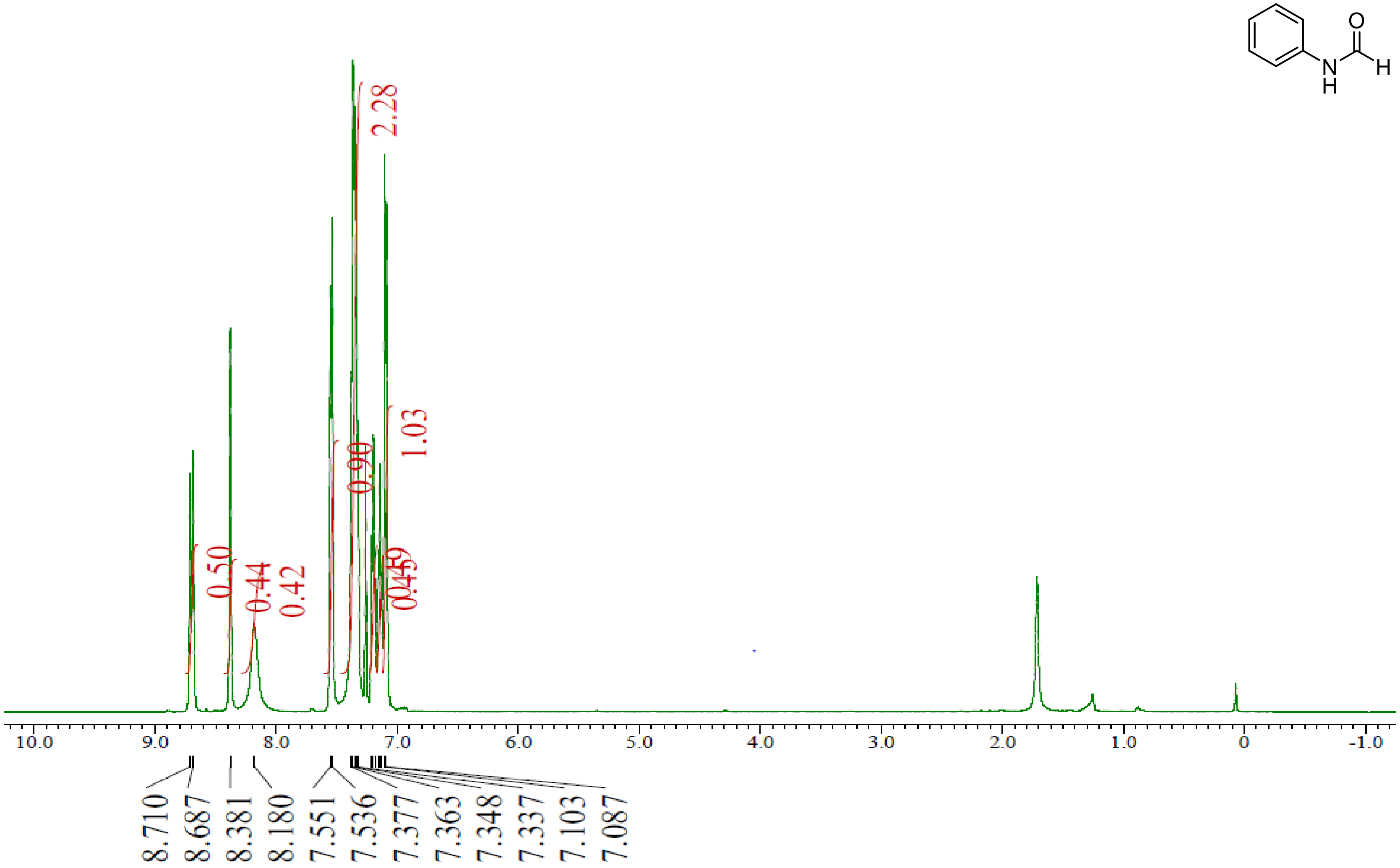

^{13}C NMR spectrum (126 MHz, $CDCl_3$) of formanilide (**2a**). Two tautomers were observed in approximately 0.5:0.5 ratio.

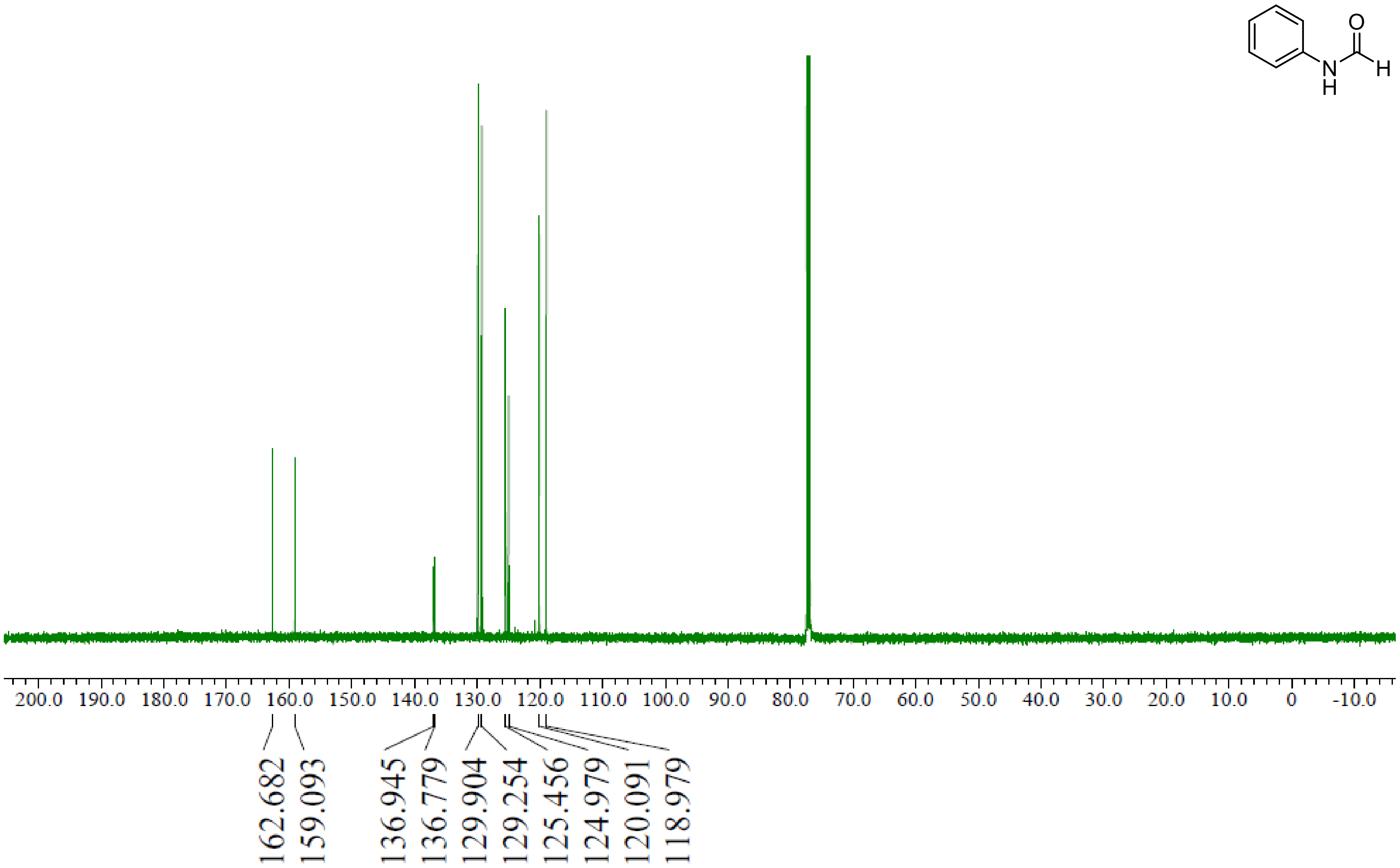

^{1}H NMR spectrum (500 MHz, DMSO-d_6) of aniline hydrochloride (**3a•HCl**).

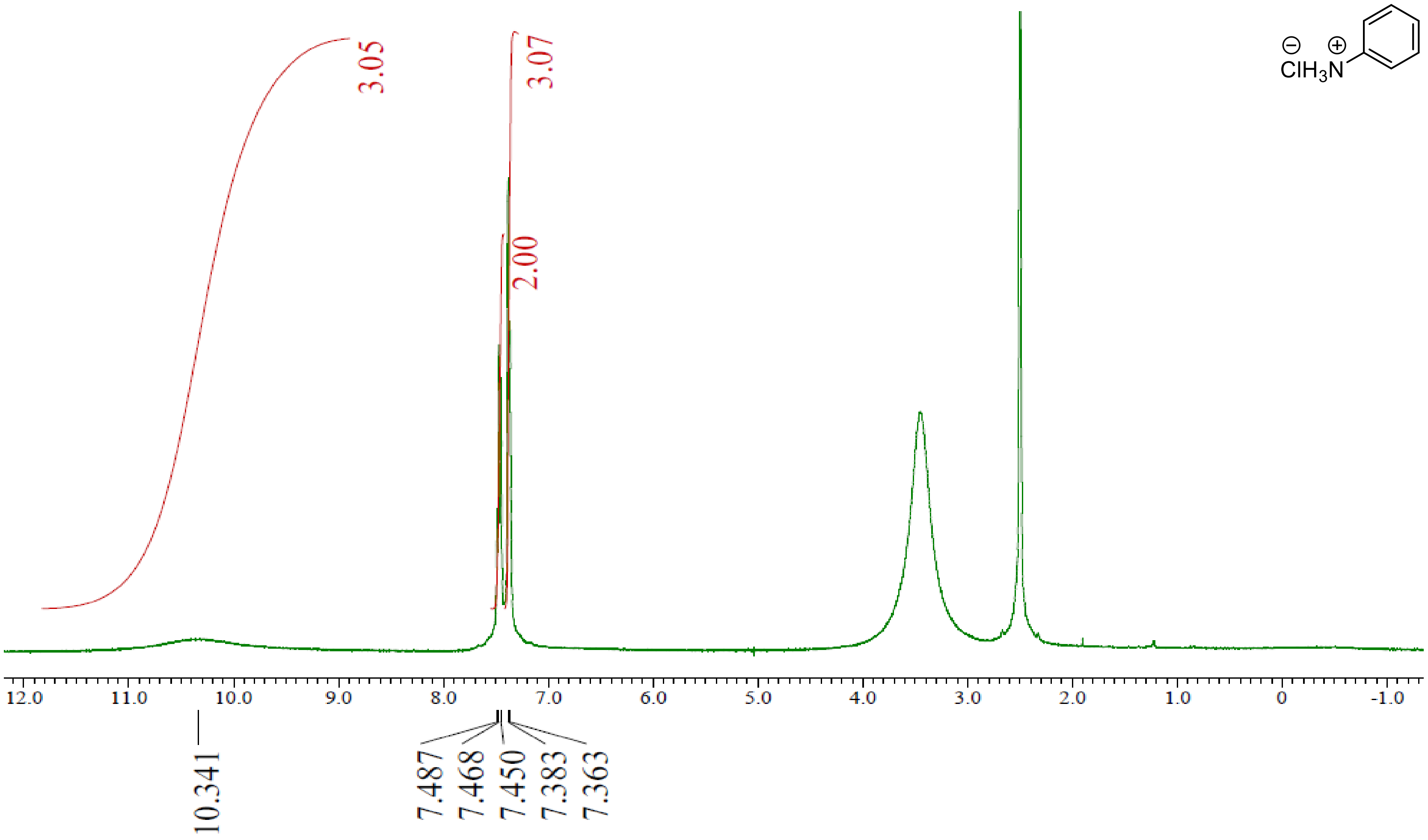

^{13}C NMR spectrum (126 MHz, DMSO-d_6) of aniline hydrochloride (**3a•HCl**).

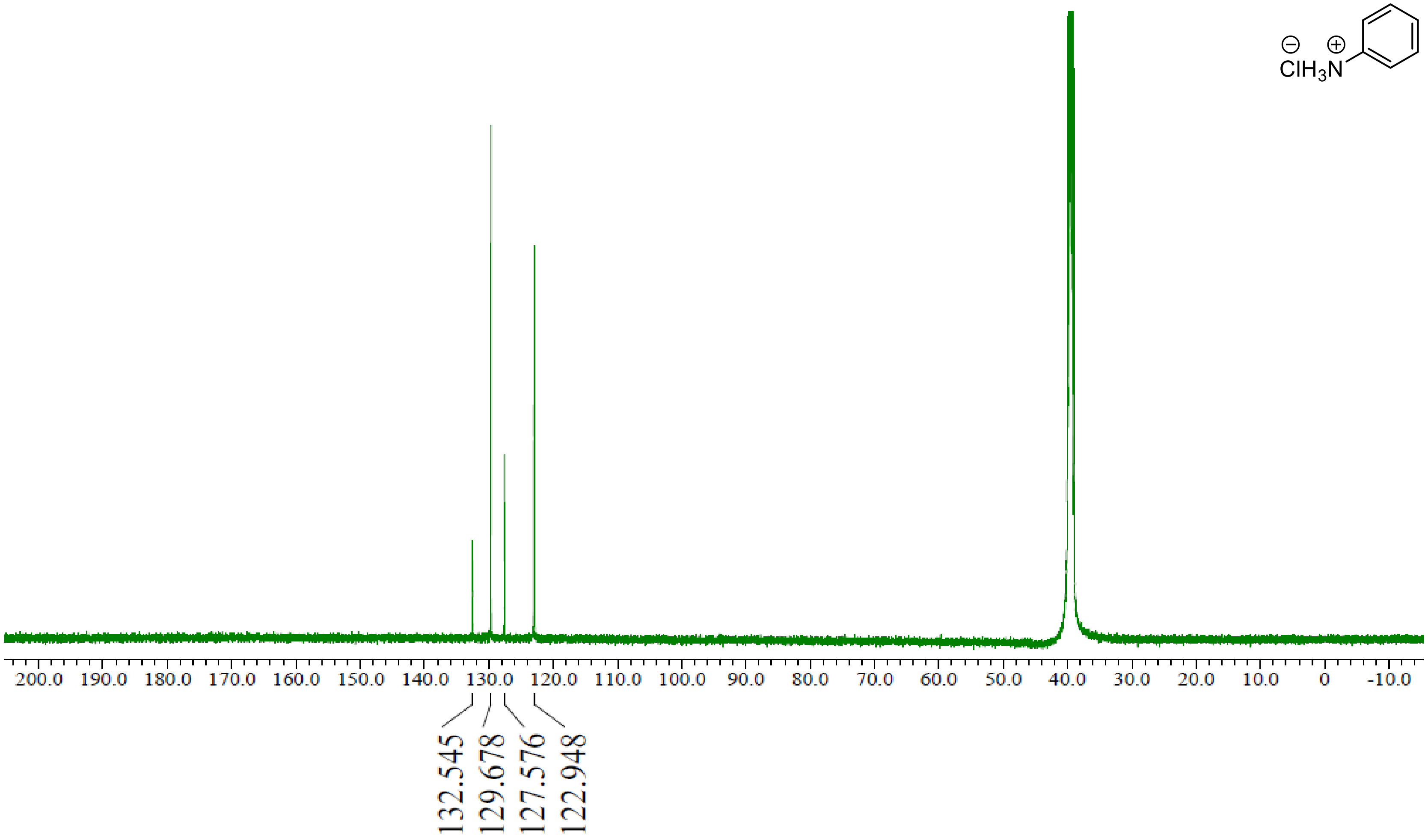

^{1}H NMR spectrum (500 MHz, $CDCl_3$) of *N*-(4-fluorophenyl)formamide (**2b**). Two tautomers were observed in approximately 0.4:0.6 ratio.

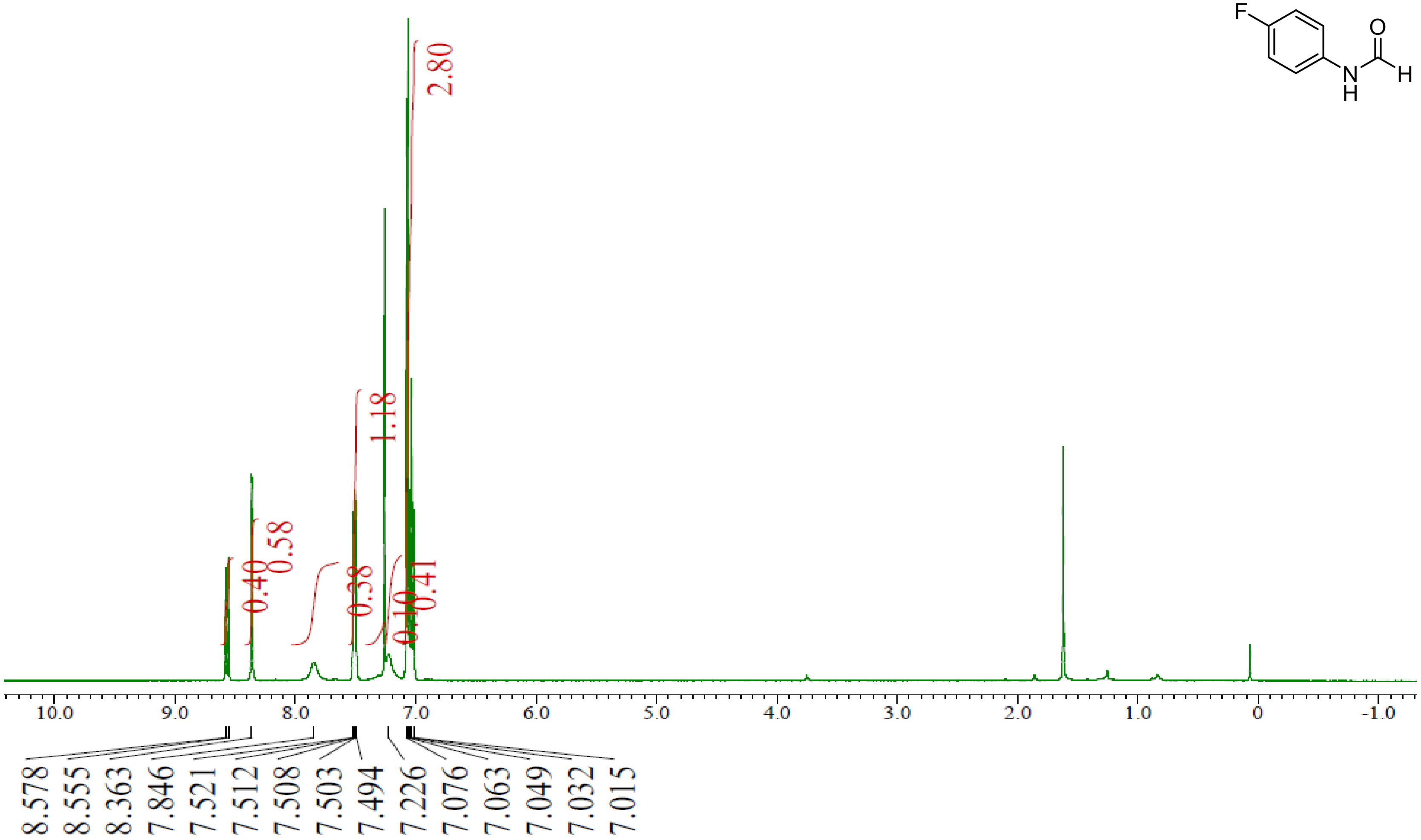

^{13}C NMR spectrum (126 MHz, $CDCl_3$) of *N*-(4-fluorophenyl)formamide (**2b**). Two tautomers were observed in approximately 0.4:0.6 ratio.

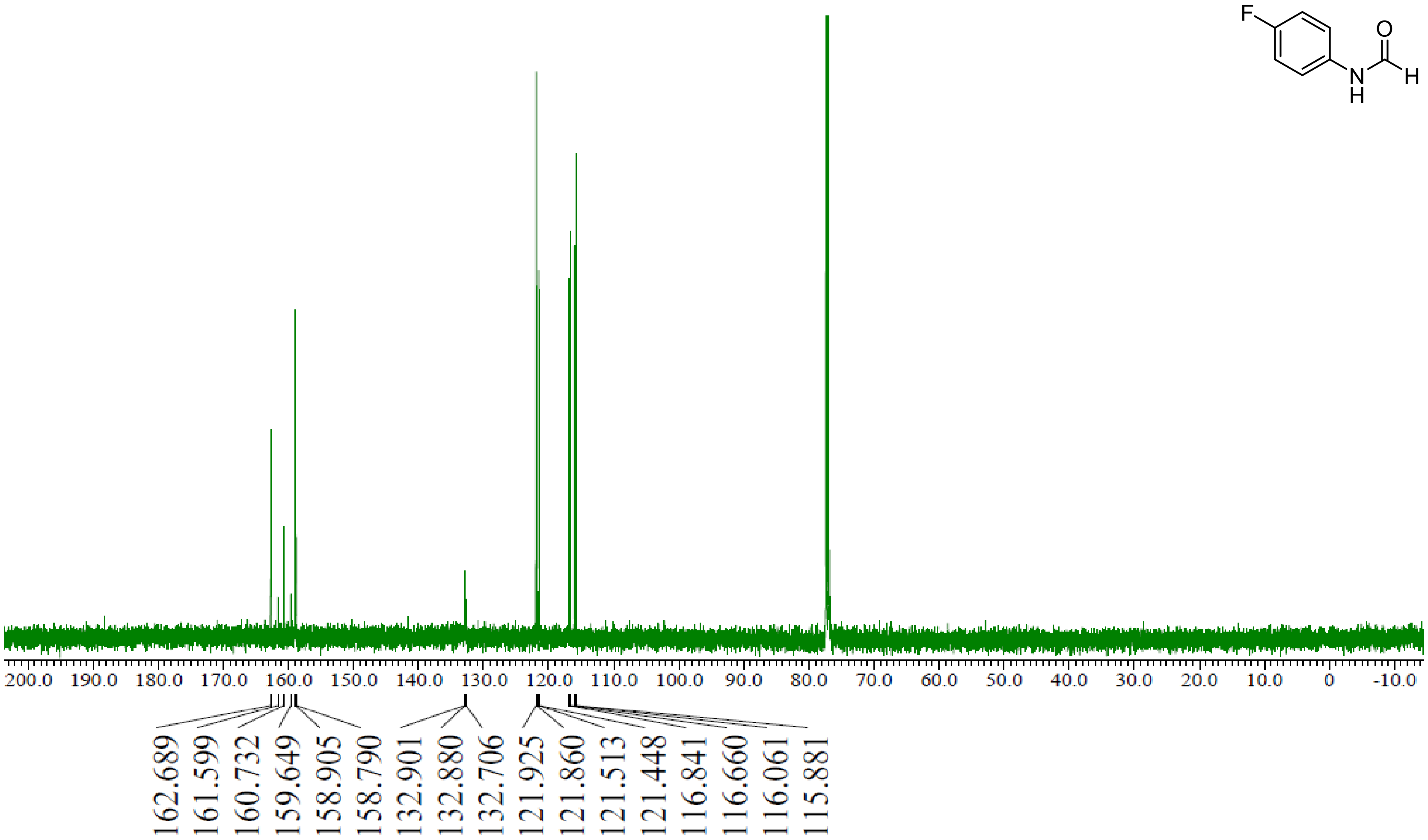

^{19}F NMR spectrum (471 MHz, $CDCl_3$) of *N*-(4-fluorophenyl)formamide (**2b**). Two tautomers were observed in approximately 0.4:0.6 ratio.

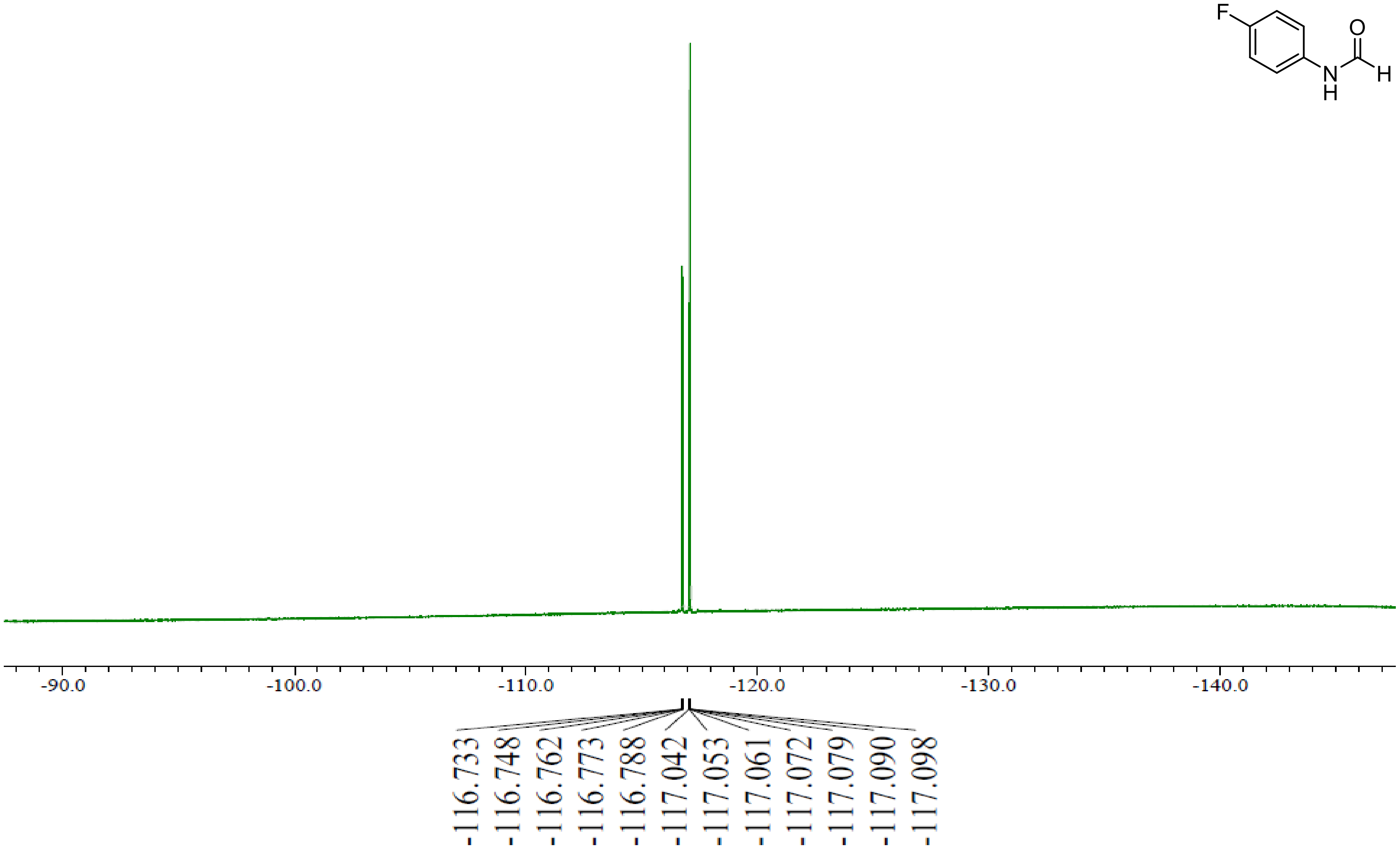

^{1}H NMR spectrum (500 MHz, DMSO-d_6) of 4-fluoroaniline hydrochloride (**3b•HCl**).

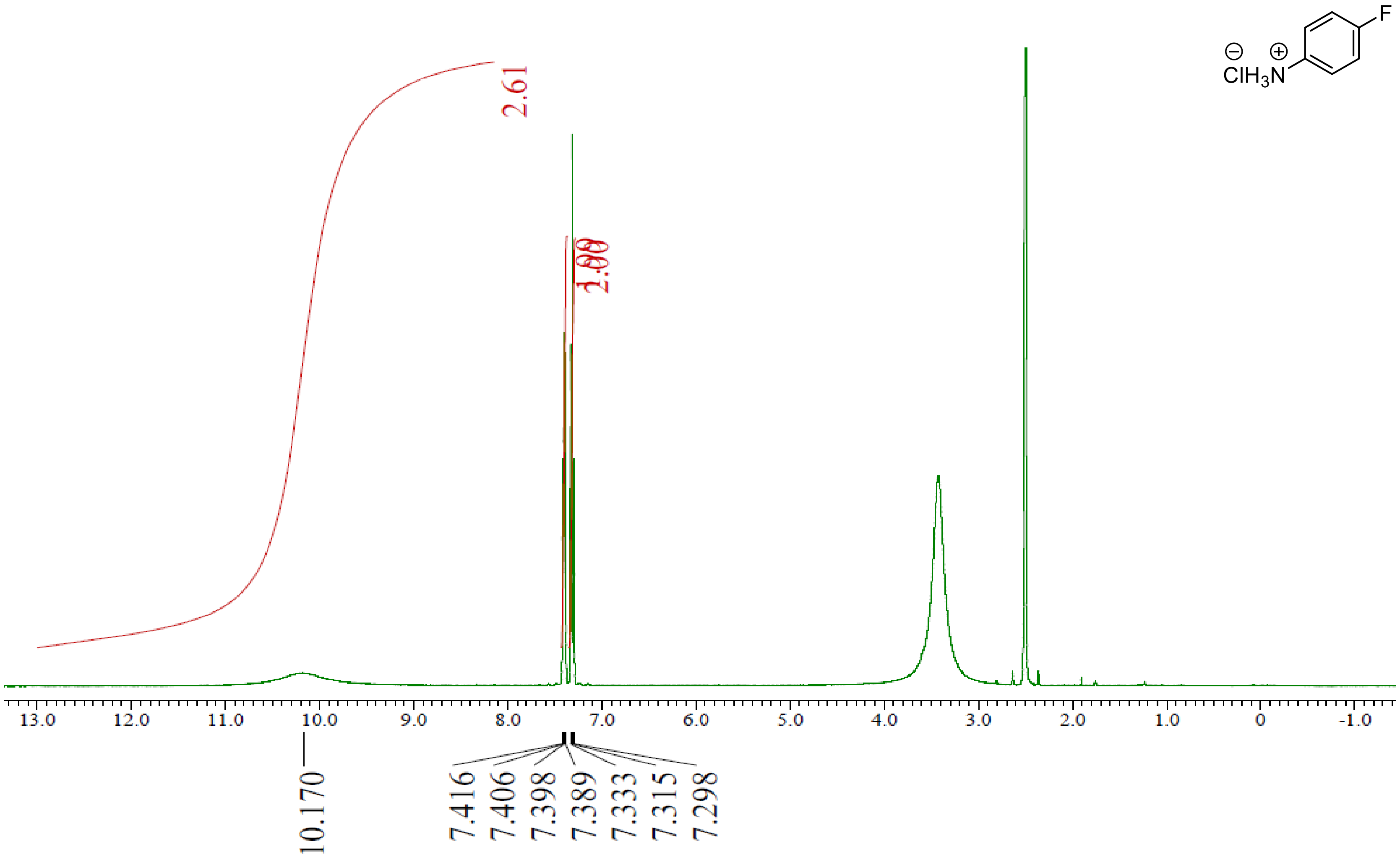

^{13}C NMR spectrum (126 MHz, DMSO-d_6) of 4-fluoroaniline hydrochloride (**3b•HCl**).

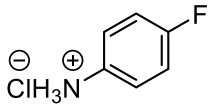

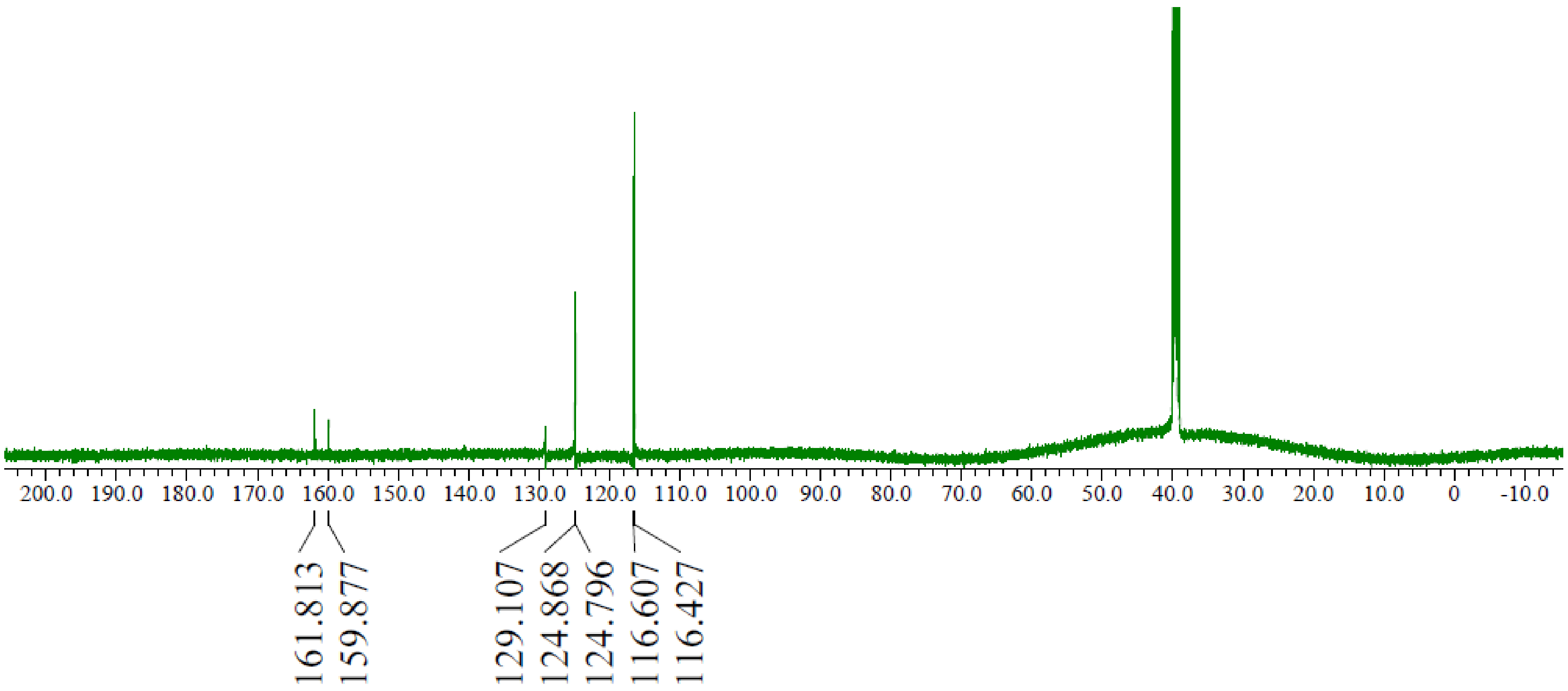

^{19}F NMR spectrum (471 MHz, CD_3OD) of 4-fluoroaniline hydrochloride (**3b•HCl**).

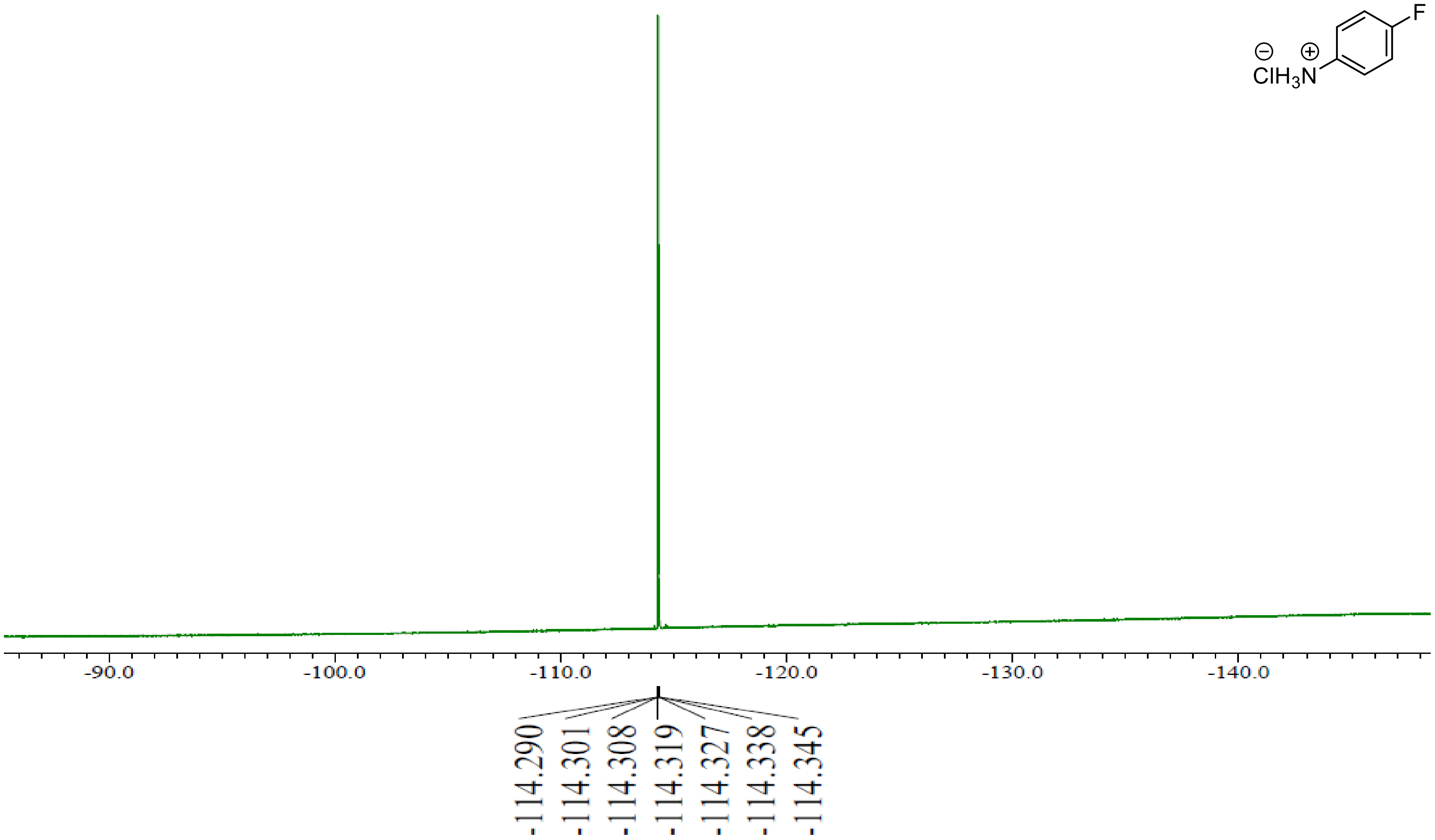

^{1}H NMR spectrum (500 MHz, $CDCl_3$) of *N*-(4-chlorophenyl)formamide (**2c**). Two tautomers were observed in approximately 0.4:0.6 ratio.

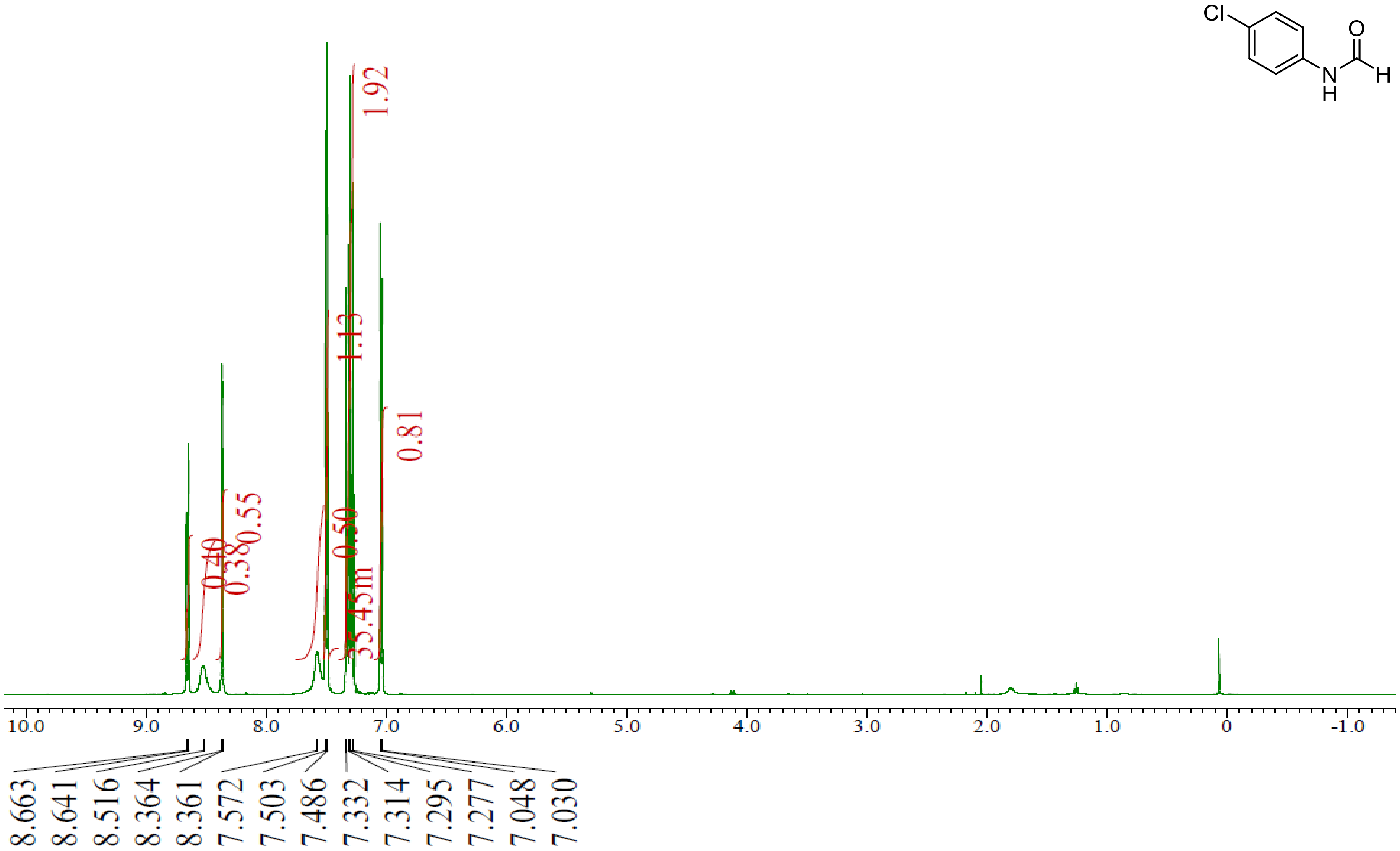

^{13}C NMR spectrum (126 MHz, $CDCl_3$) of *N*-(4-chlorophenyl)formamide (**2c**). Two tautomers were observed in approximately 0.4:0.6 ratio.

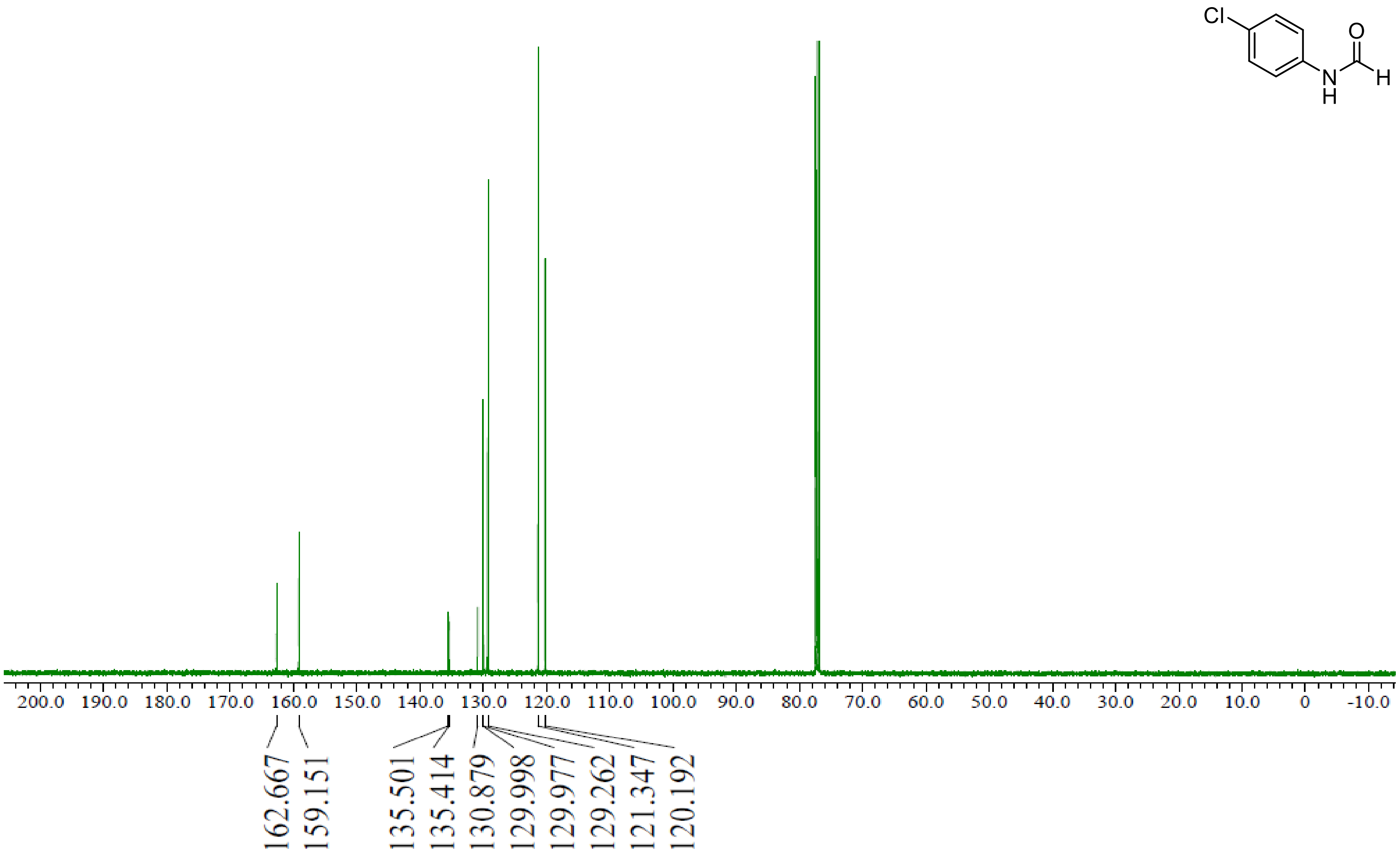

^{1}H NMR spectrum (500 MHz, $CDCl_3$) of 4-chloroaniline (**3c**).

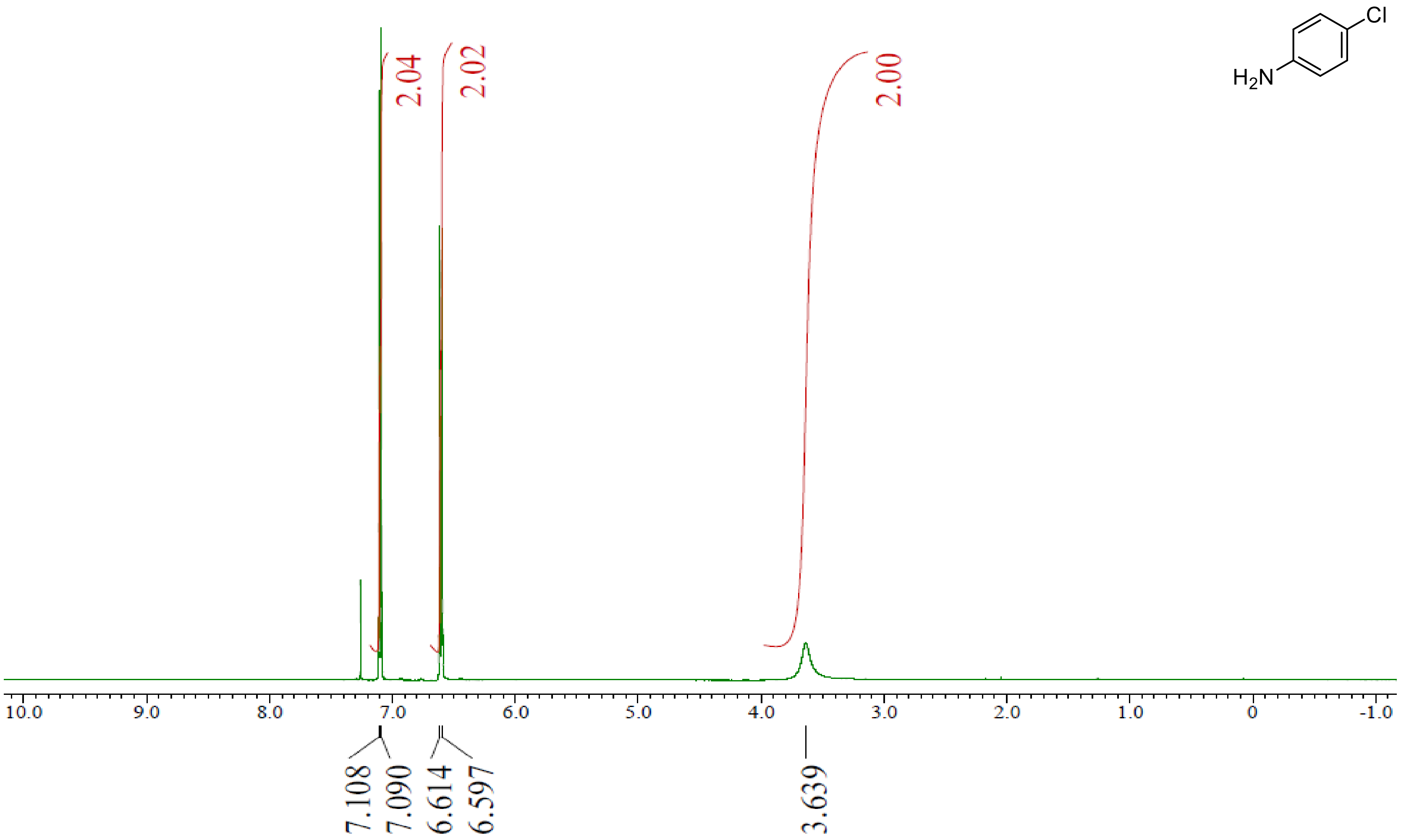

^{13}C NMR spectrum (126 MHz, $CDCl_3$) of 4-chloroaniline (**3c**).

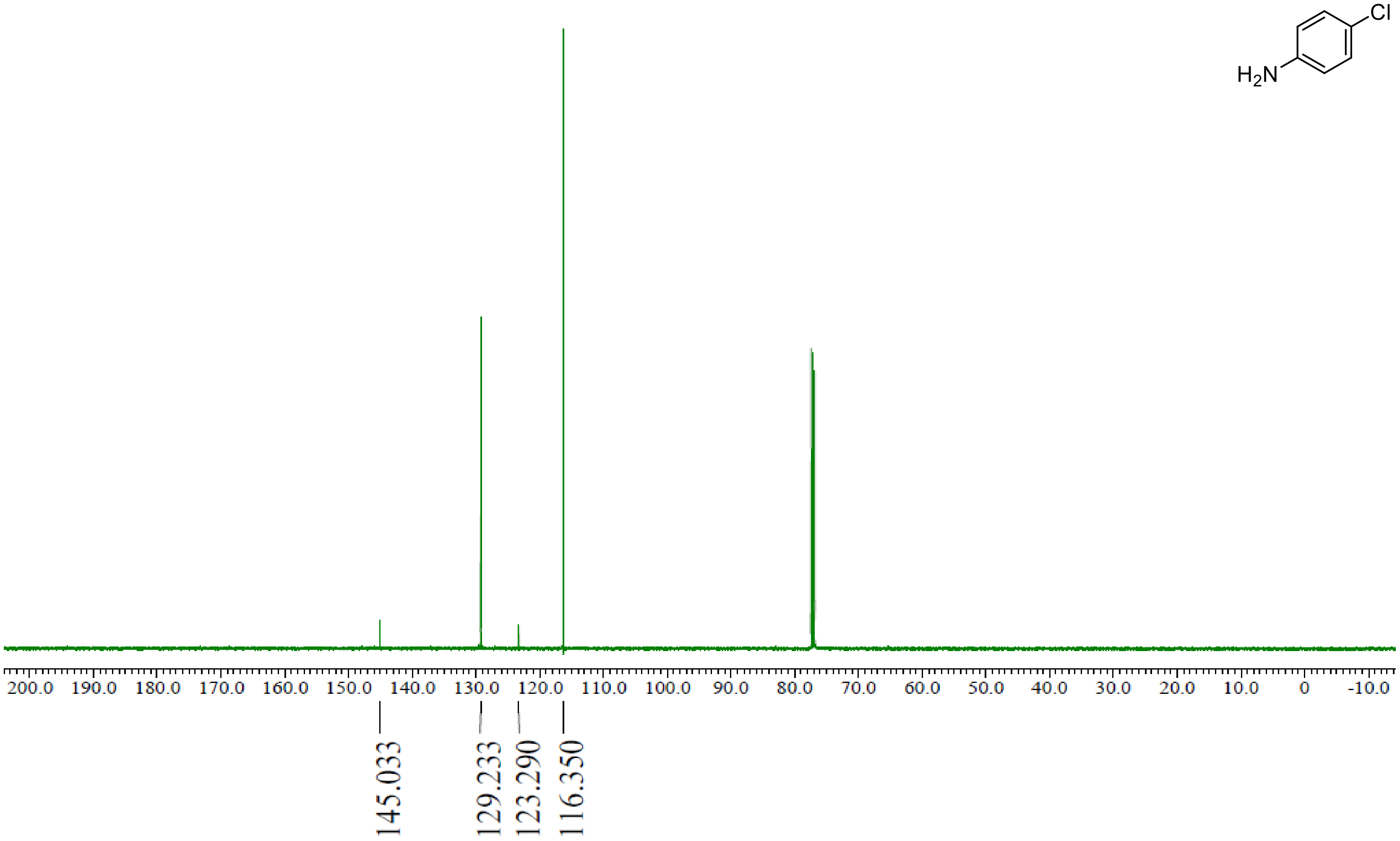

^{1}H NMR spectrum (500 MHz, $CDCl_3$) of *N*-(3-chlorophenyl)formamide (**2d**). Two tautomers were observed in approximately 0.4:0.6 ratio.

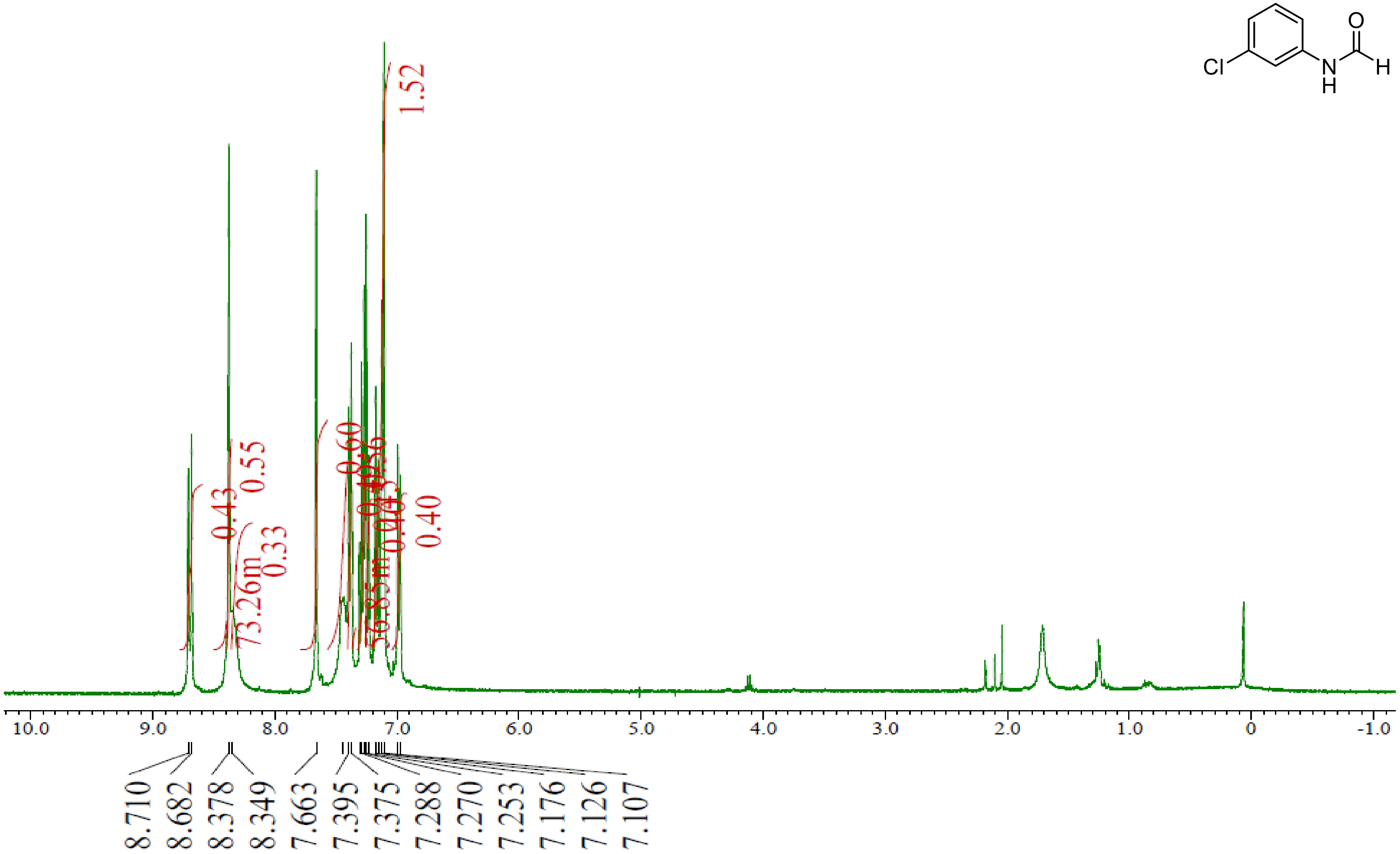

^{13}C NMR spectrum (126 MHz, $CDCl_3$) of *N*-(3-chlorophenyl)formamide (**2d**). Two tautomers were observed in approximately 0.4:0.6 ratio.

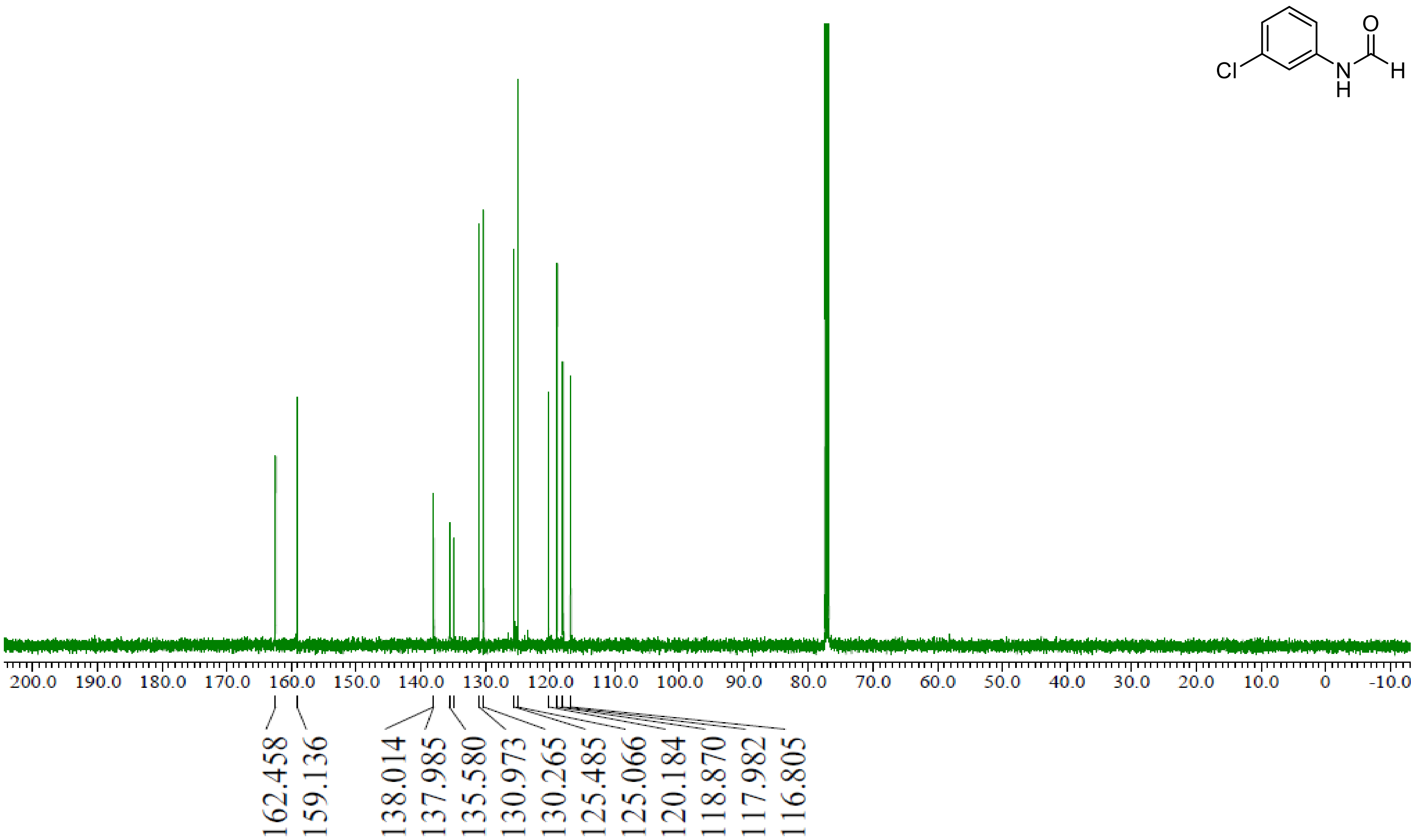

^{1}H NMR spectrum (500 MHz, D_2O) of 3-chloroaniline hydrochloride (**3d•HCl**).

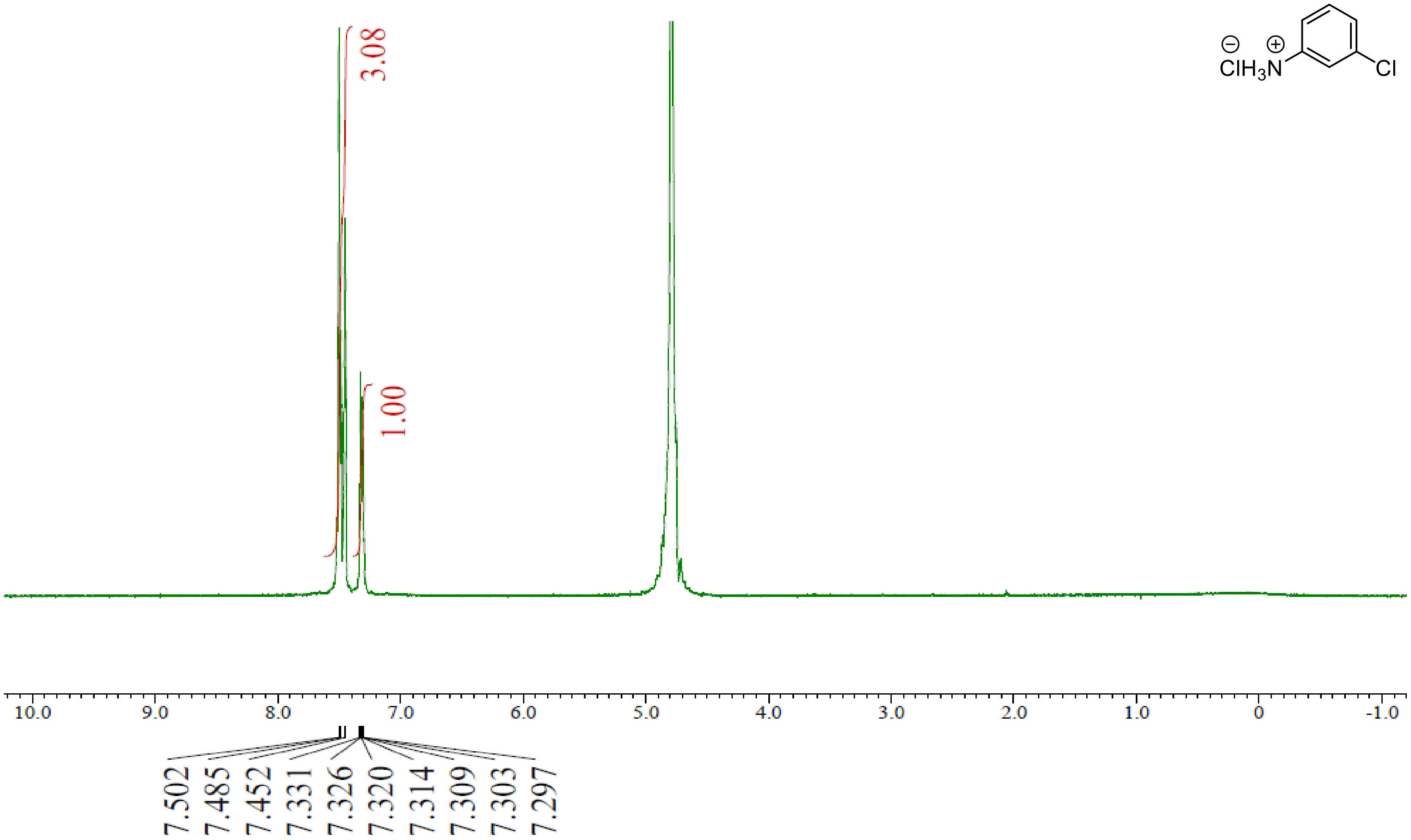

^{13}C NMR spectrum (126 MHz, D_2O) of 3-chloroaniline hydrochloride (**3d•HCl**).

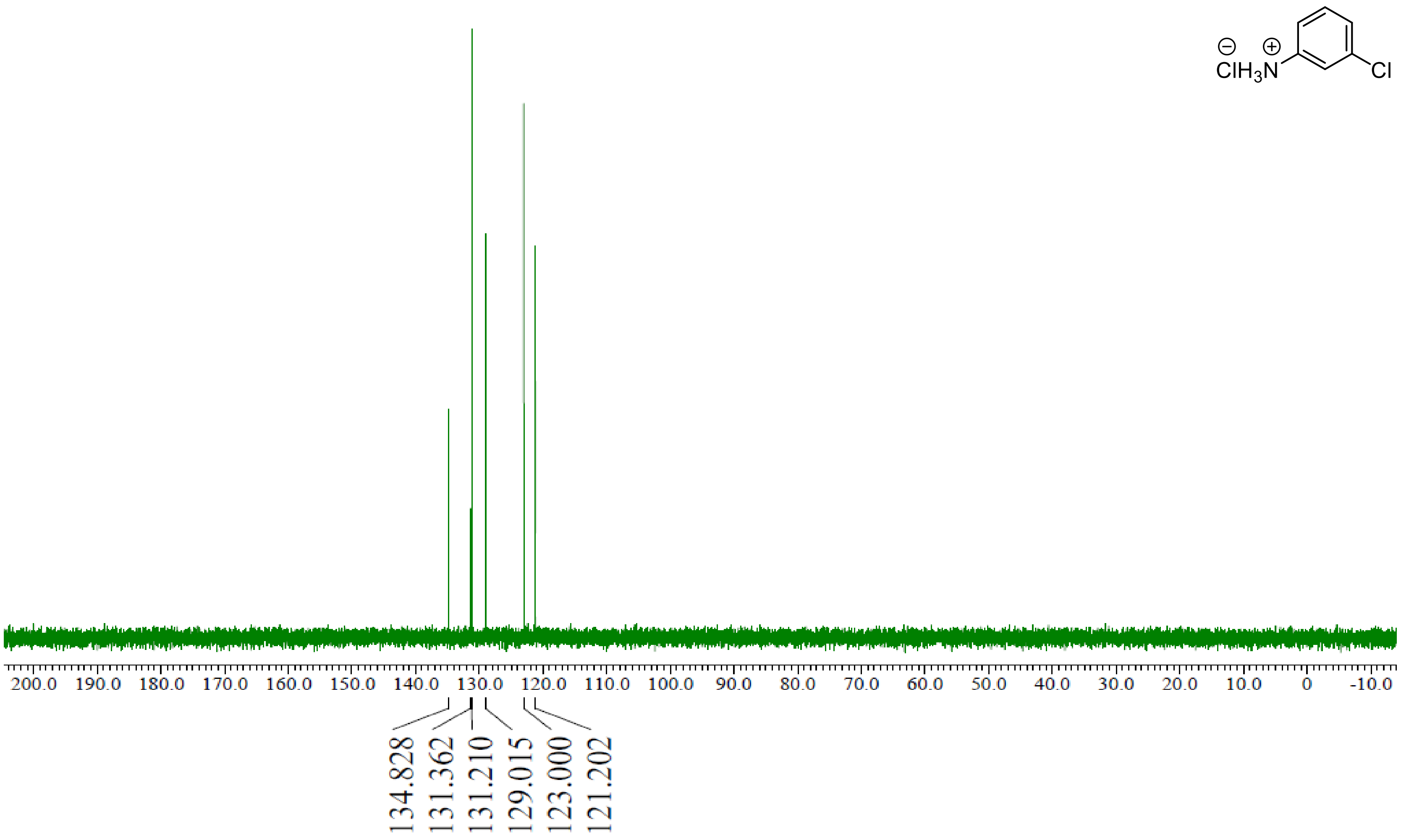

^{1}H NMR spectrum (500 MHz, $CDCl_3$) of *N*-(2-chlorophenyl)formamide (**2e**). Two tautomers were observed in approximately 0.33:0.67 ratio.

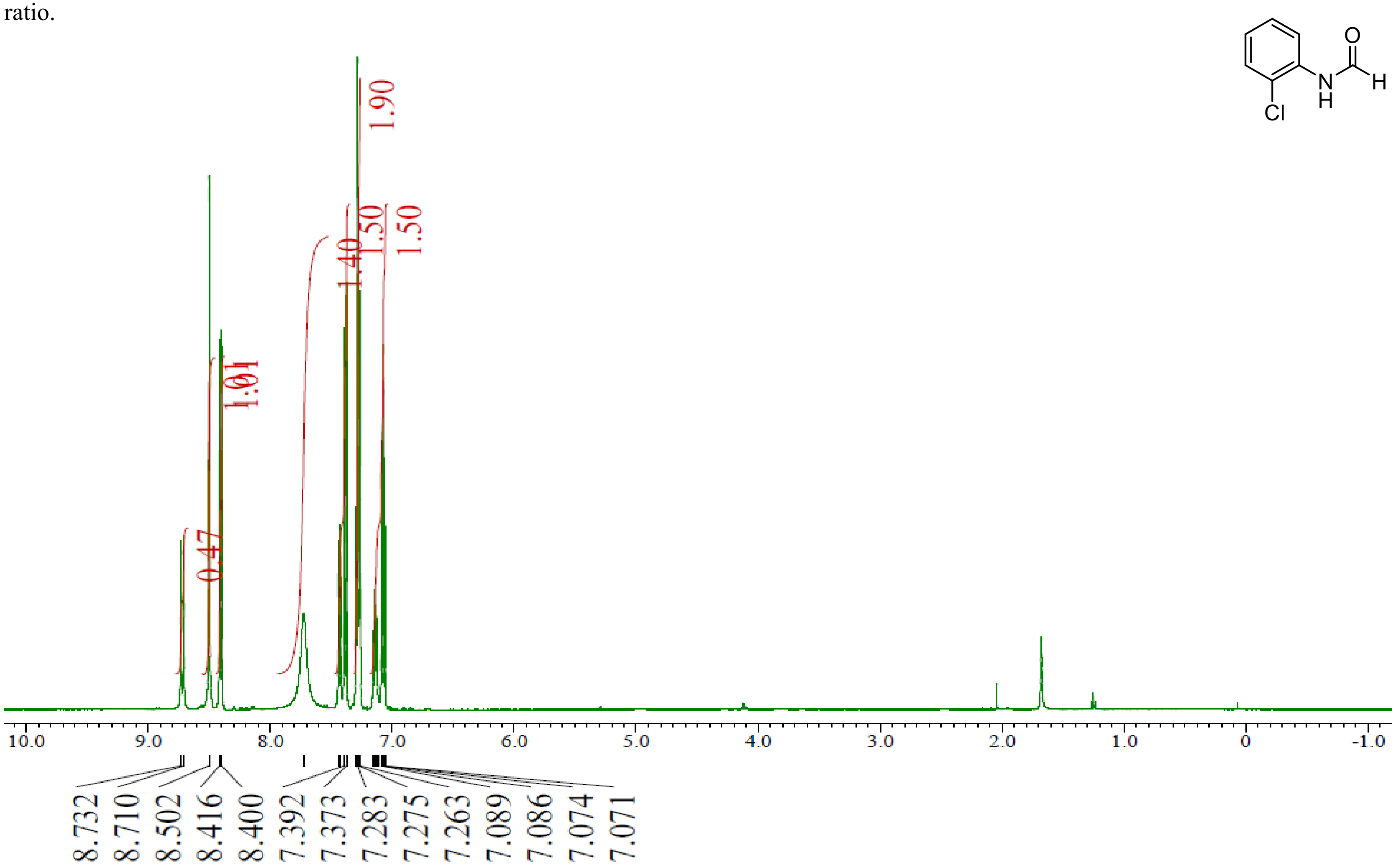

^{13}C NMR spectrum (126 MHz, $CDCl_3$) of *N*-(2-chlorophenyl)formamide (**2e**). Two tautomers were observed in approximately 0.33:0.67 ratio.

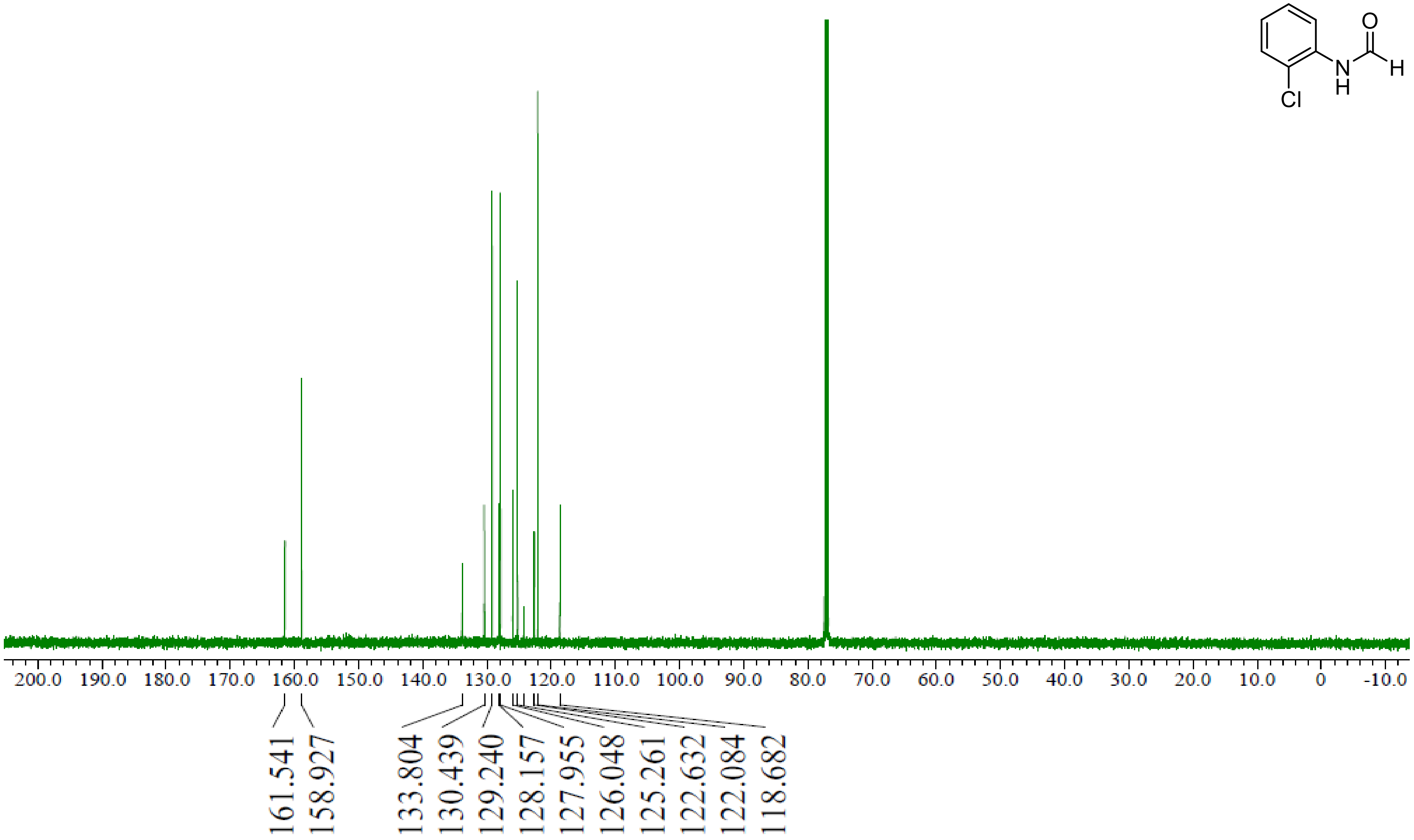

^{1}H NMR spectrum (500 MHz, D_2O) of 2-chloroaniline hydrochloride (**3e•HCl**).

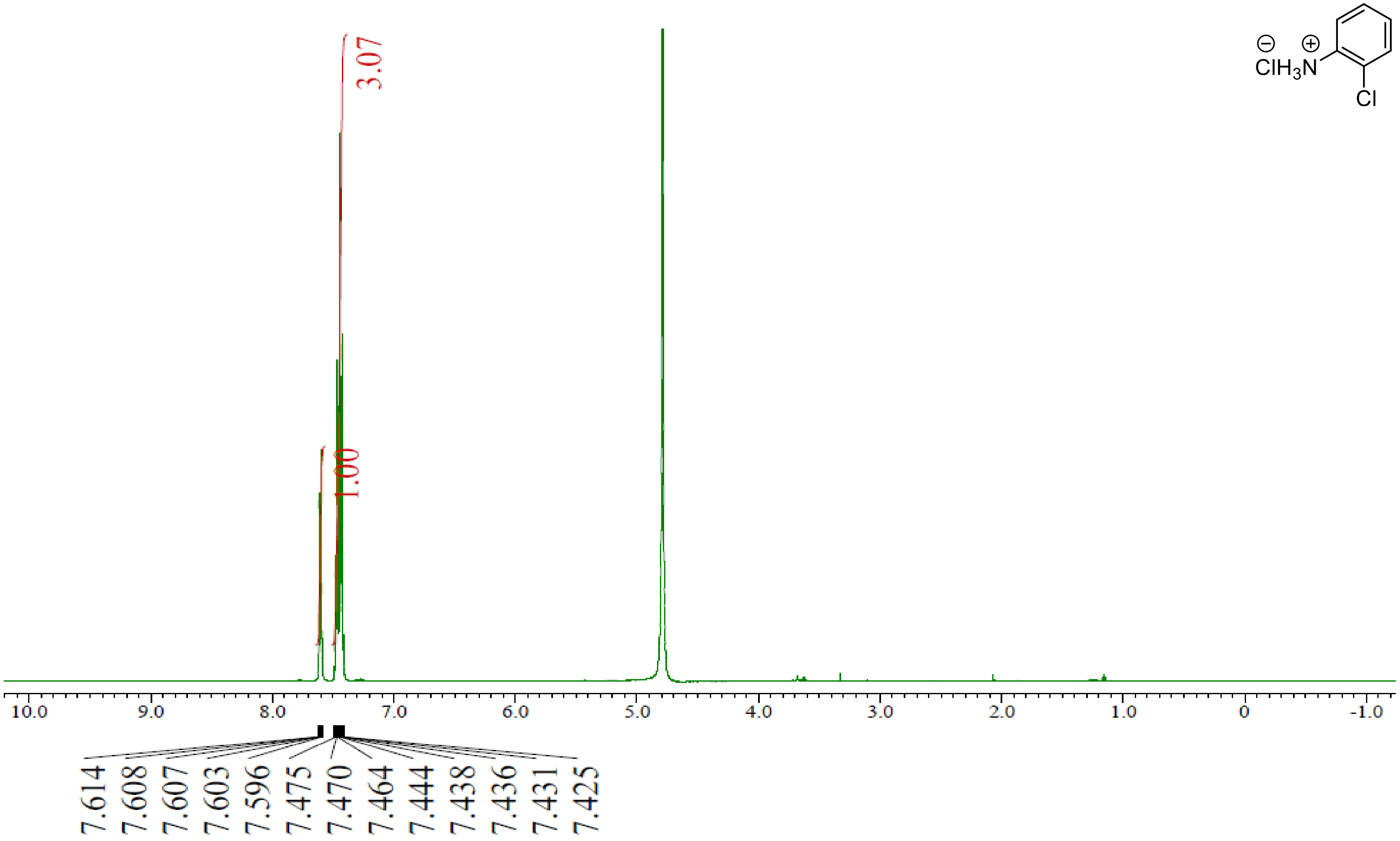

^{13}CNMR spectrum (126 MHz, D_2O) of 2-chloroaniline hydrochloride (**3e•HCl**).

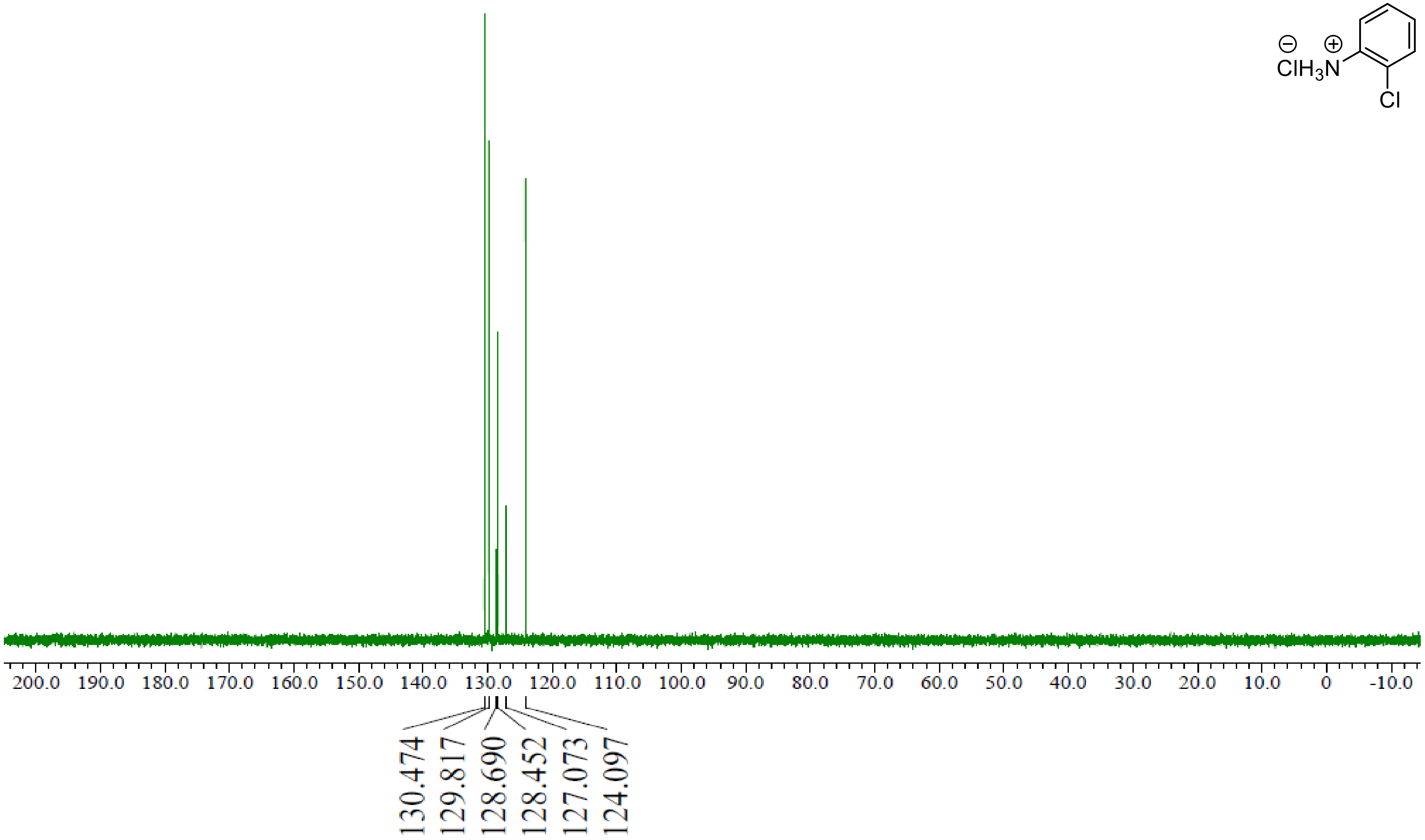

^{1}H NMR spectrum (500 MHz, $CDCl_3$) of *N*-(4-bromophenyl)formamide (**2f**). Two tautomers were observed in approximately 0.4:0.6 ratio.

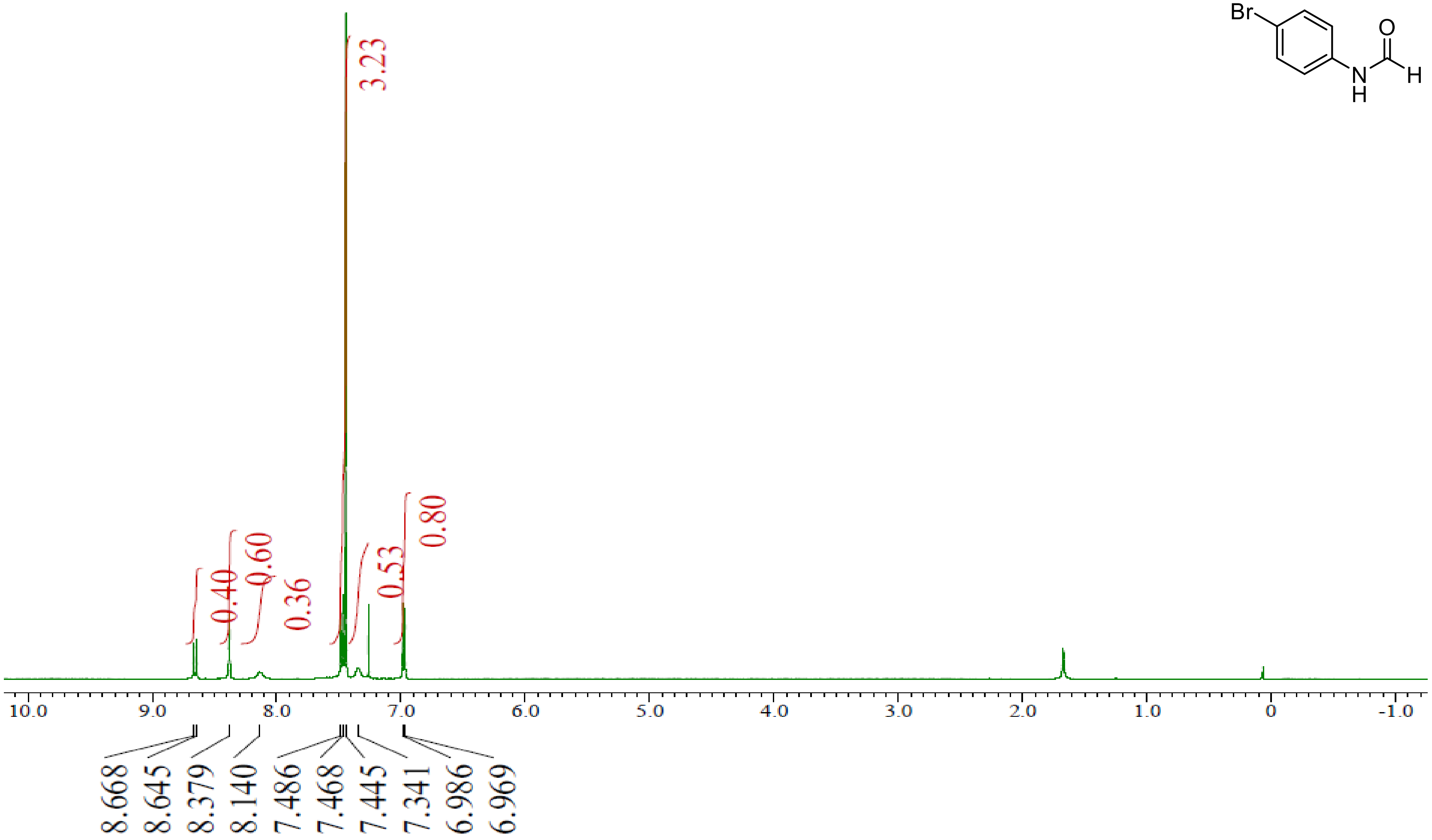

^{13}C NMR spectrum (126 MHz, $CDCl_3$) of *N*-(4-bromophenyl)formamide (**2f**). Two tautomers were observed in approximately 0.4:0.6 ratio.

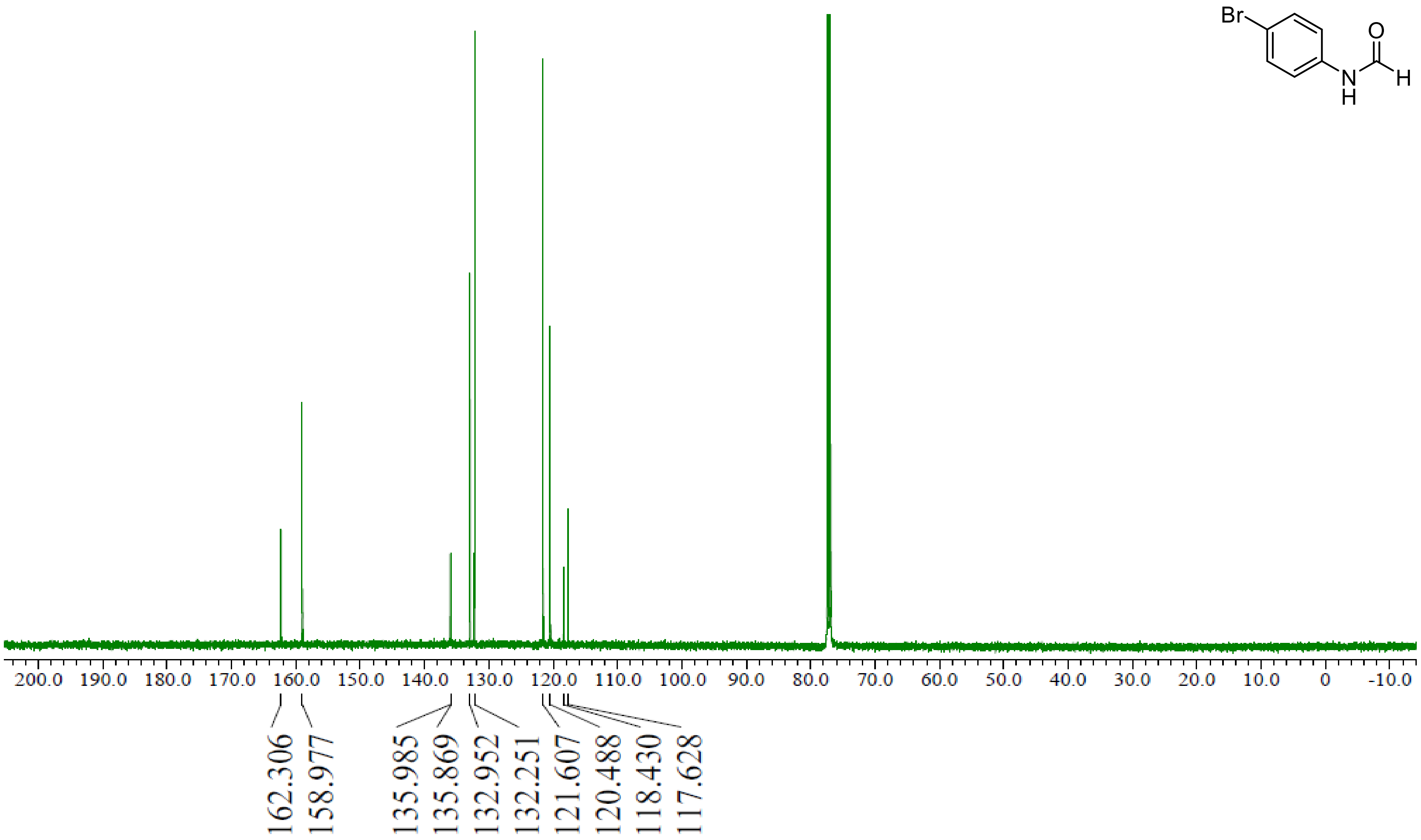

^{1}H NMR spectrum (500 MHz, $CDCl_3$) of 4-bromoaniline (**3f**).

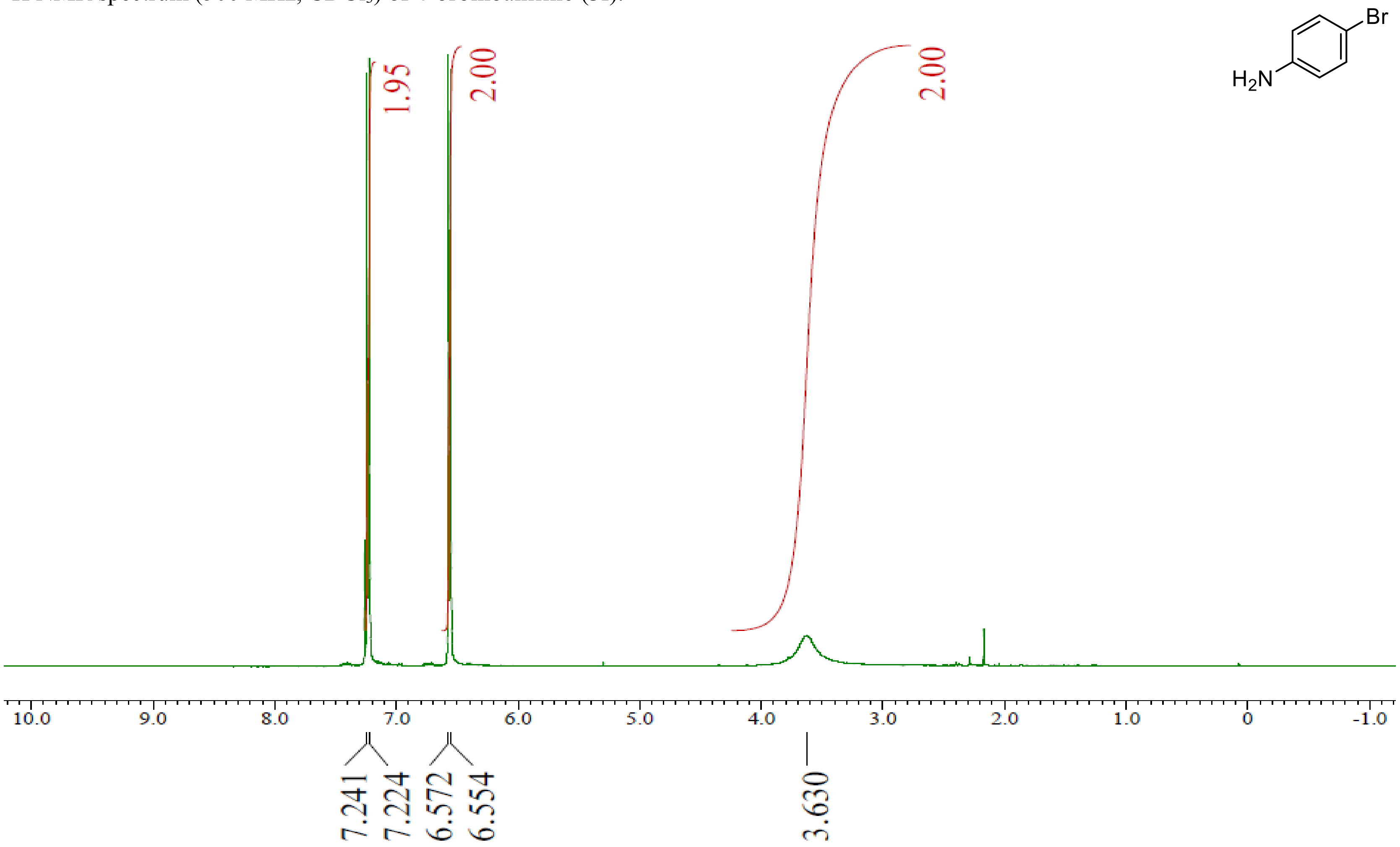

^{13}C NMR spectrum (126 MHz, $CDCl_3$) of 4-bromoaniline (**3f**).

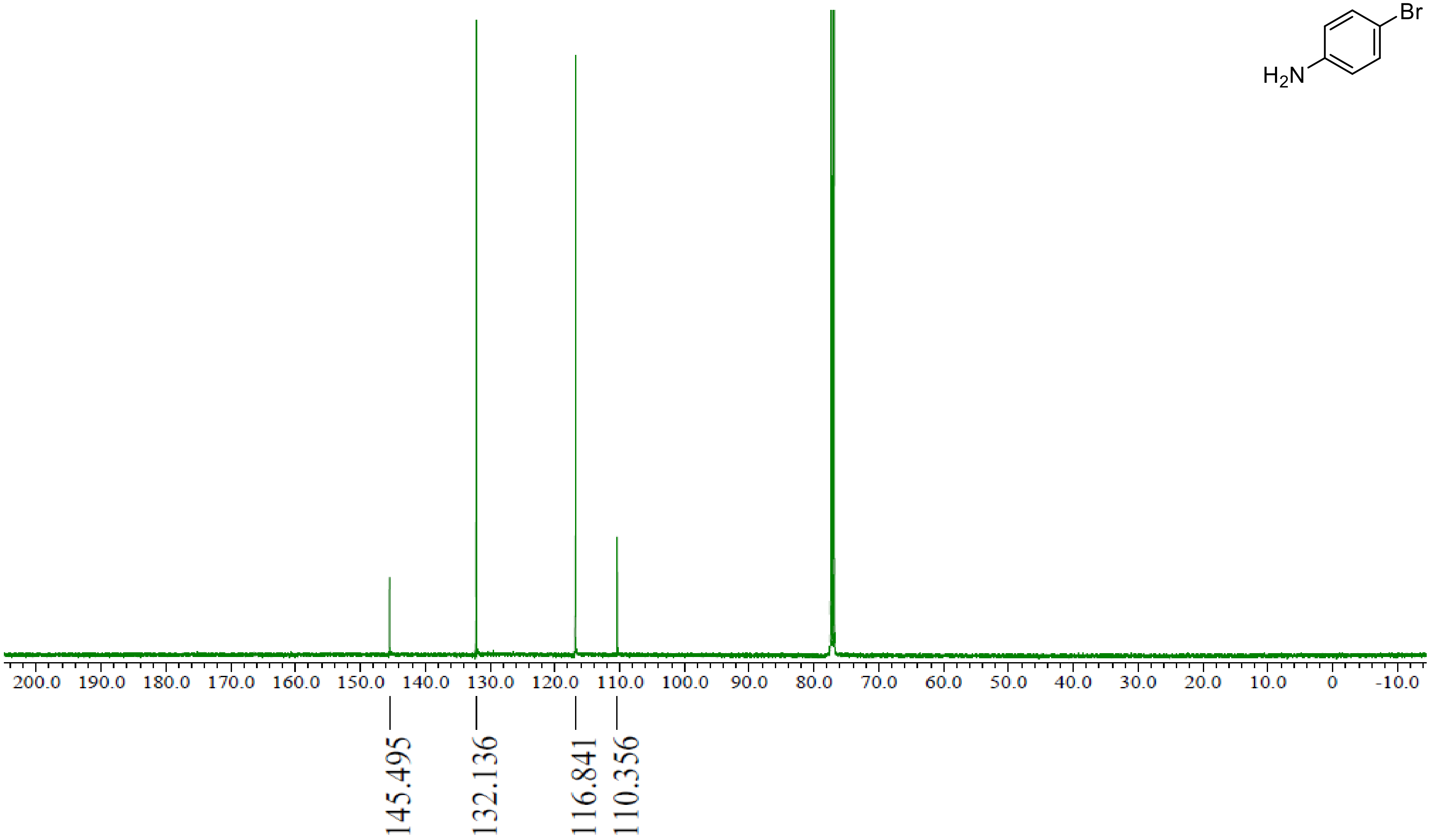

^{1}H NMR spectrum (500 MHz, $CDCl_3$) of *N*-(4-*tert*-butylphenyl)formamide (**2g**). Two tautomers were observed in approximately 0.5:0.5 ratio.

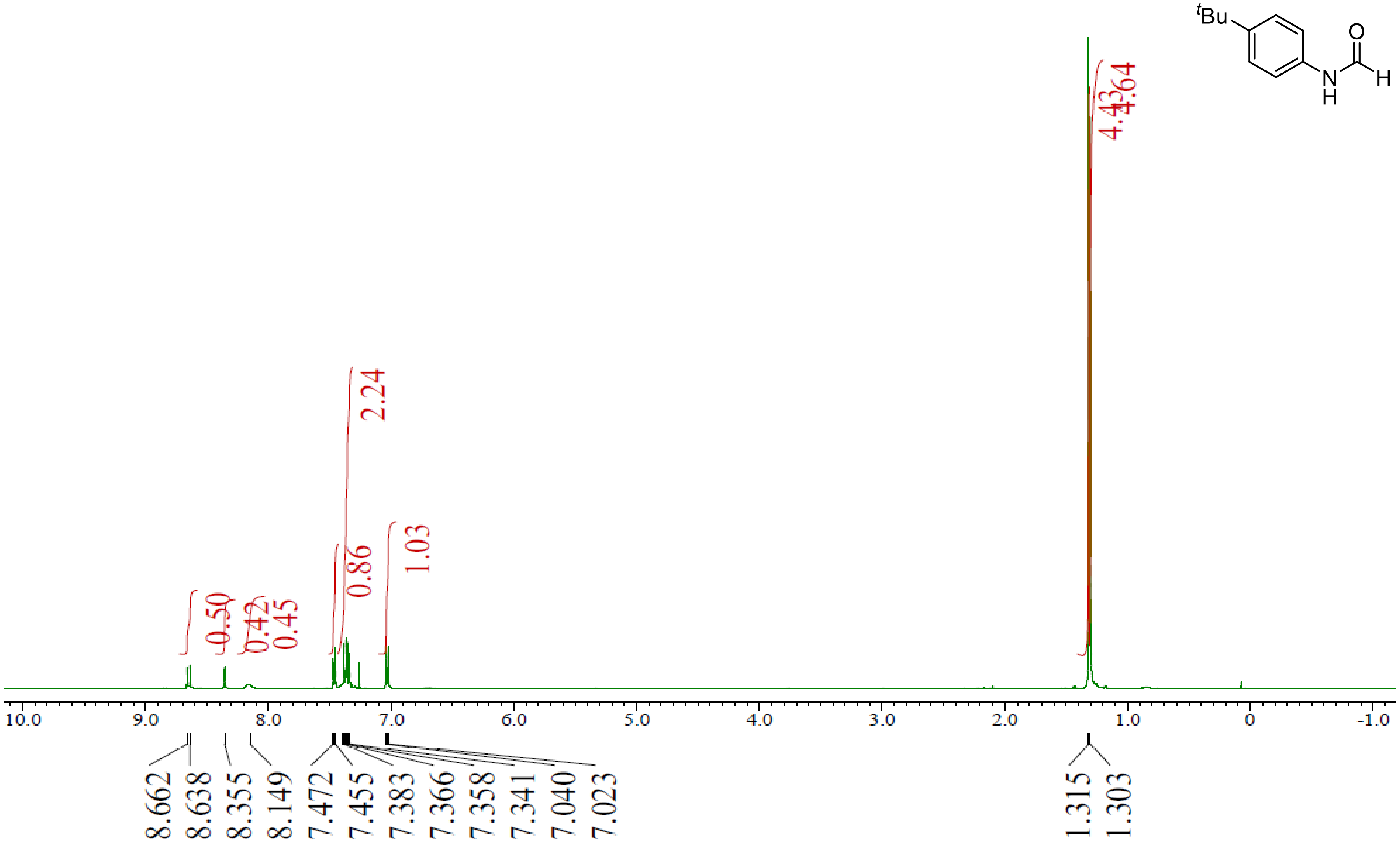

^{13}C NMR spectrum (126 MHz, $CDCl_3$) of *N*-(4-*tert*-butylphenyl)formamide (**2g**). Two tautomers were observed in approximately 0.5:0.5 ratio.

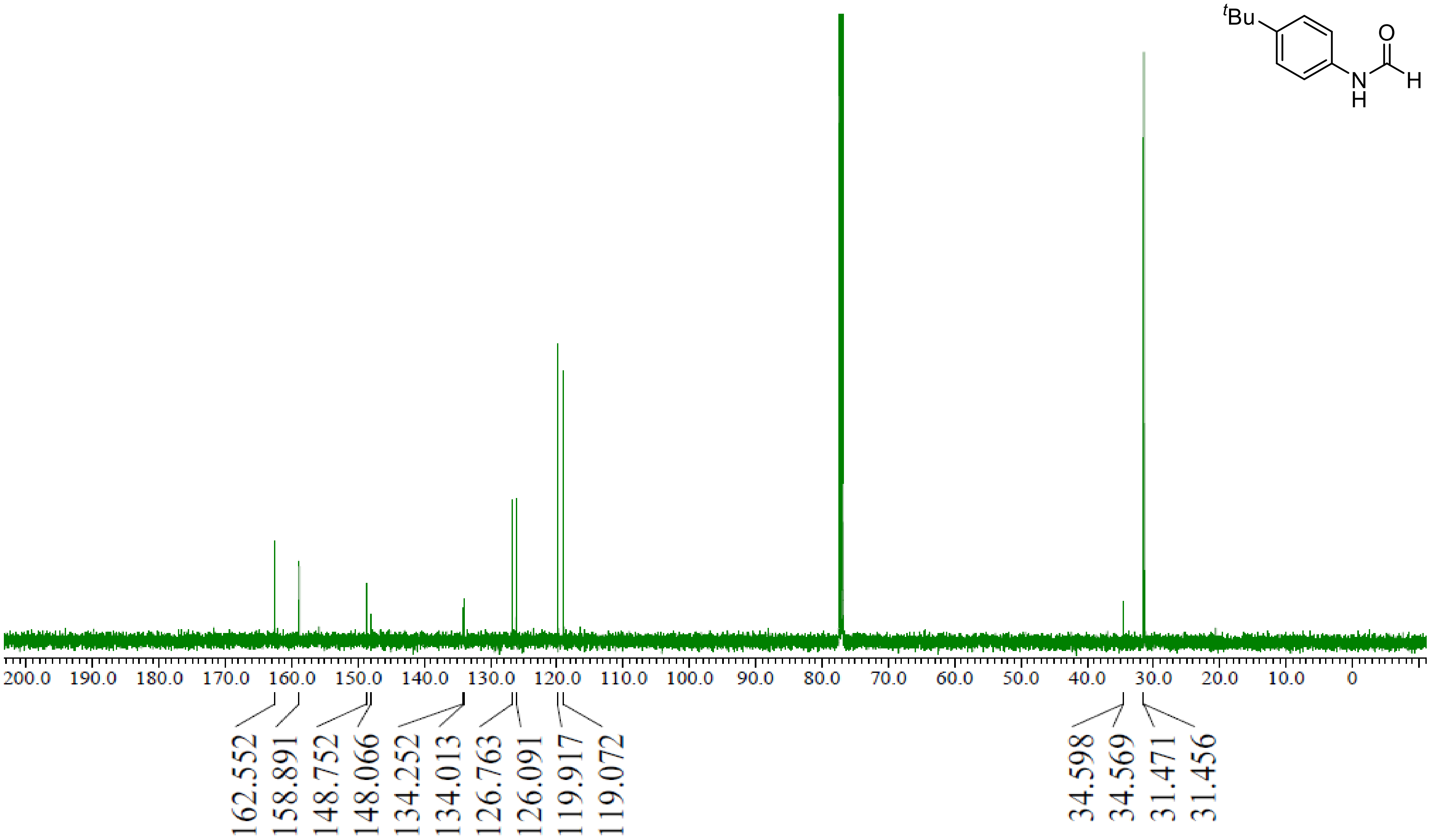

^{1}H NMR spectrum (500 MHz, $CDCl_3$) of 4-*tert*-butylaniline hydrochloride (**3g•HCl**).

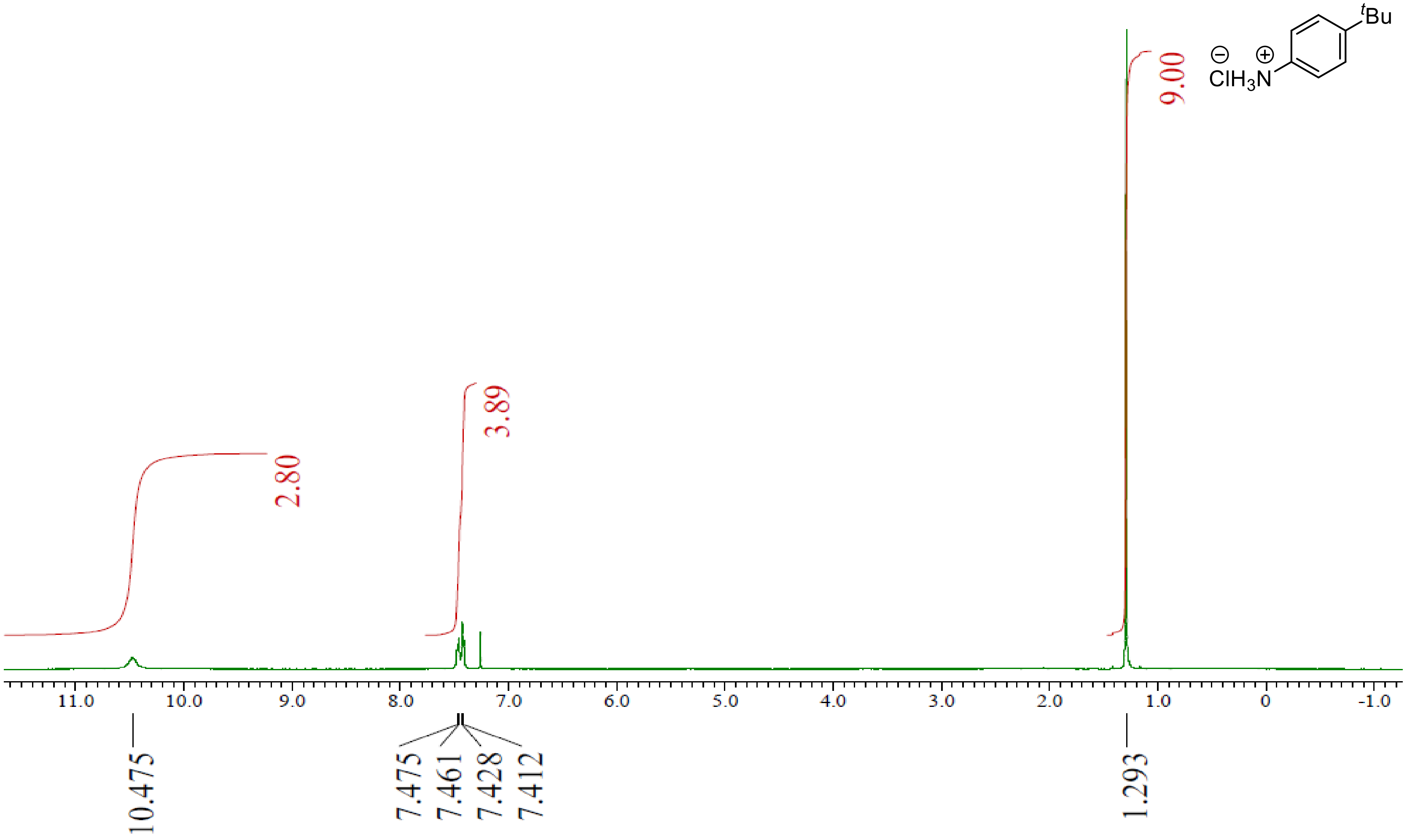

^{13}C NMR spectrum (126 MHz, $CDCl_3$) of 4-*tert*-butylaniline hydrochloride (**3g•HCl**).

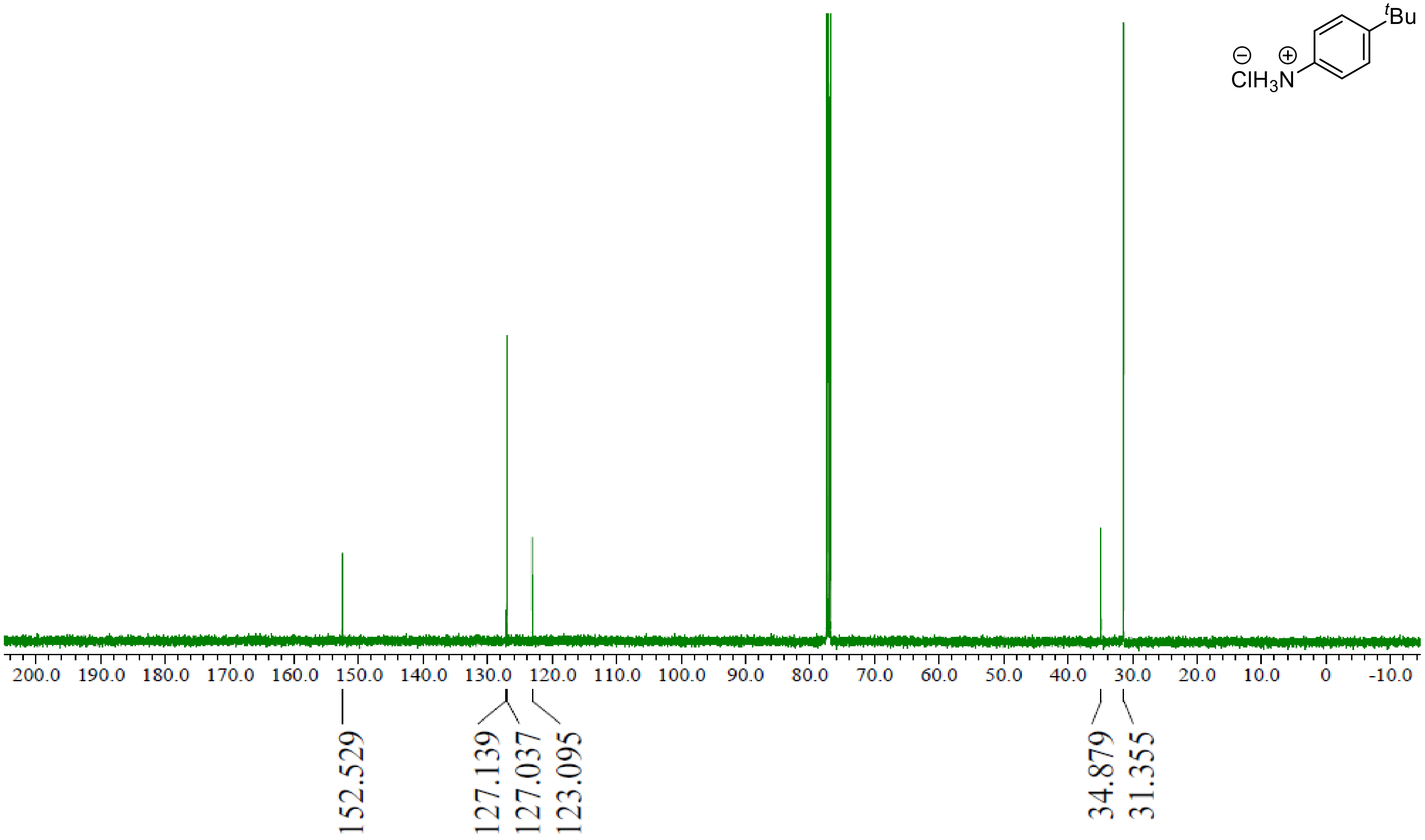

^{1}H NMR spectrum (500 MHz, $CDCl_3$) of *N*-(4-methoxyphenyl)formamide (**2h**). Two tautomers were observed in approximately 0.5:0.5 ratio.

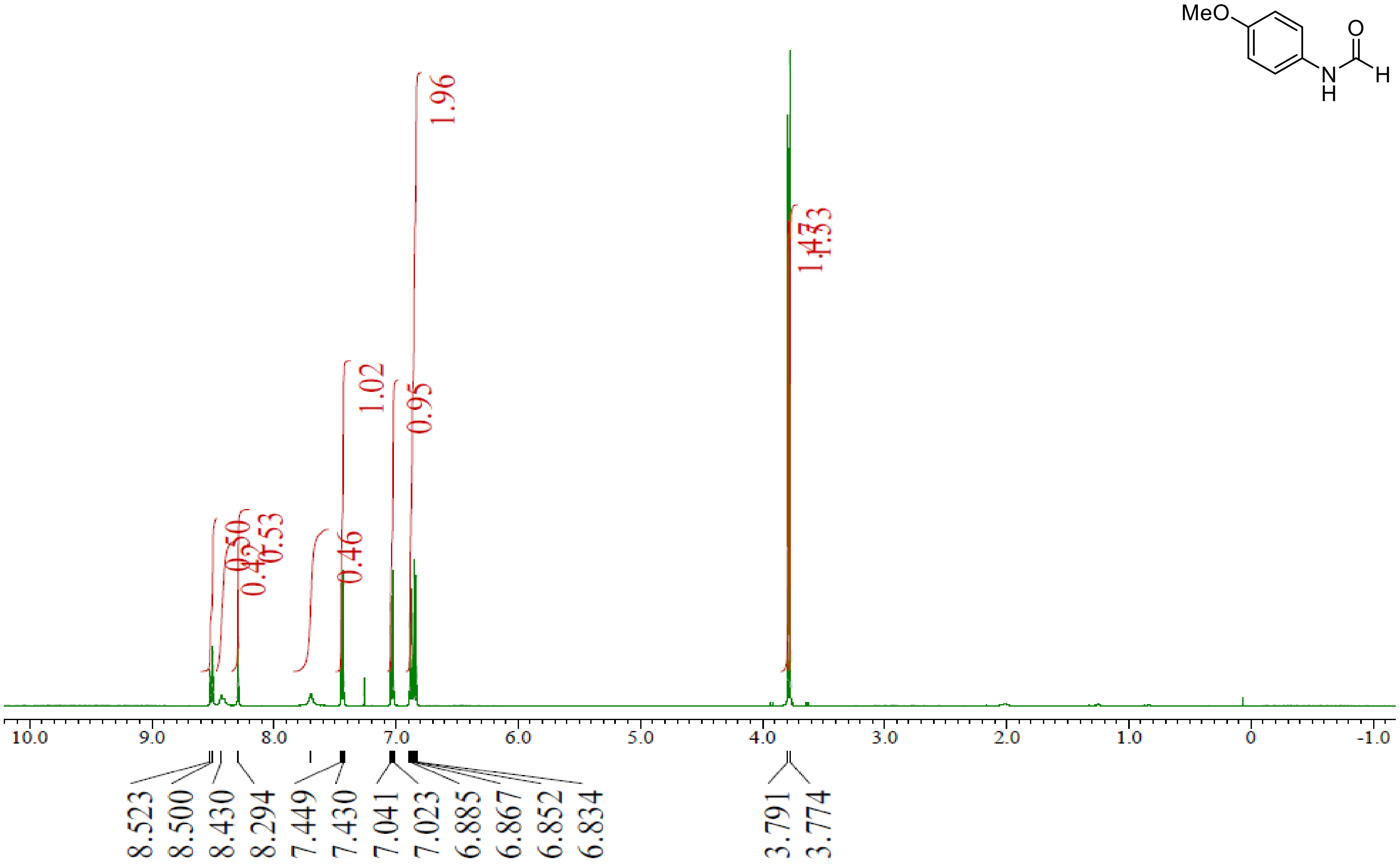

^{13}C NMR spectrum (126 MHz, $CDCl_3$) of *N*-(4-methoxyphenyl)formamide (**2h**). Two tautomers were observed in approximately 0.5:0.5 ratio.

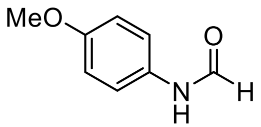

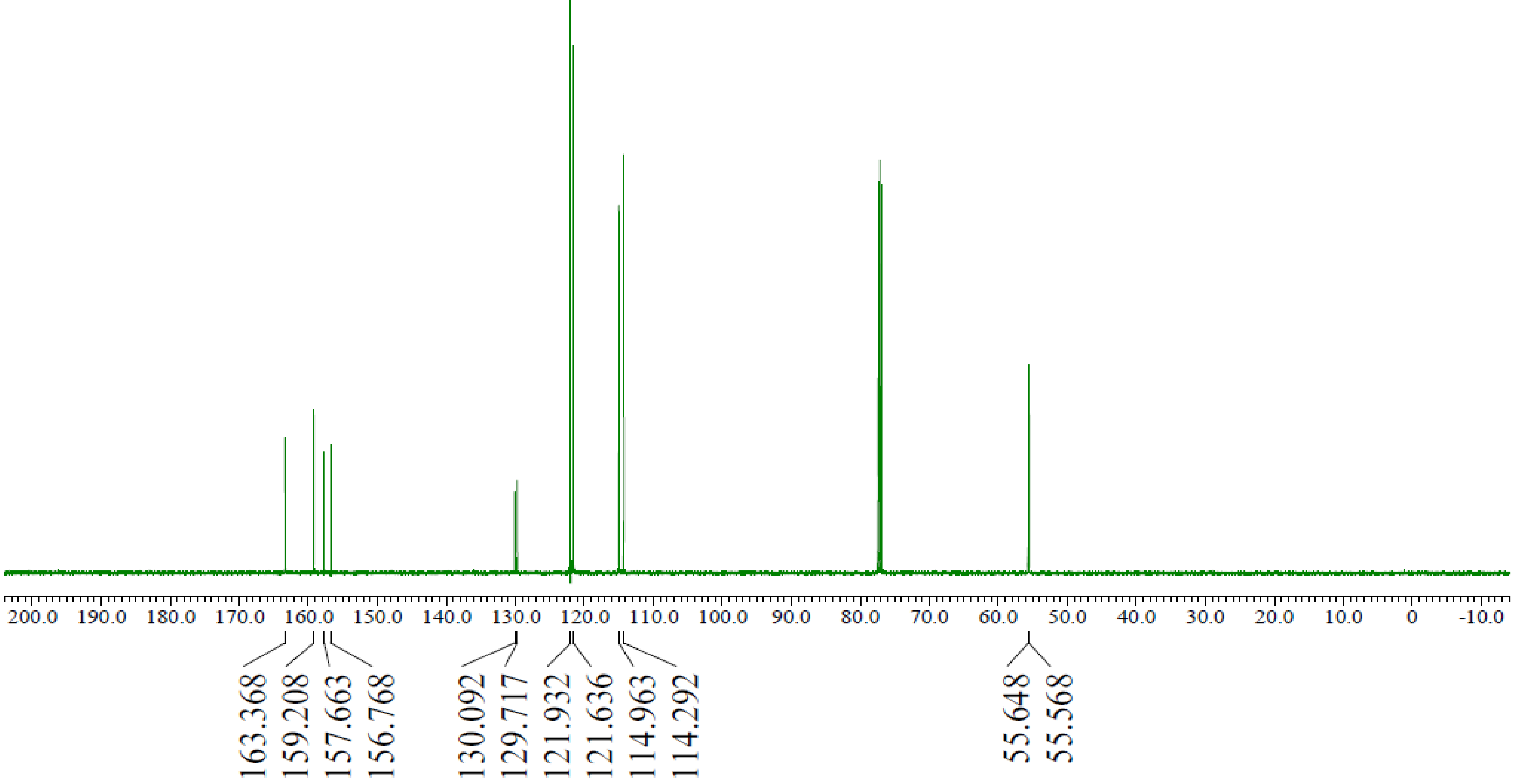

^{1}H NMR spectrum (500 MHz, DMSO-d_6) of 4-methoxyaniline hydrochloride (**3h•HCl**).

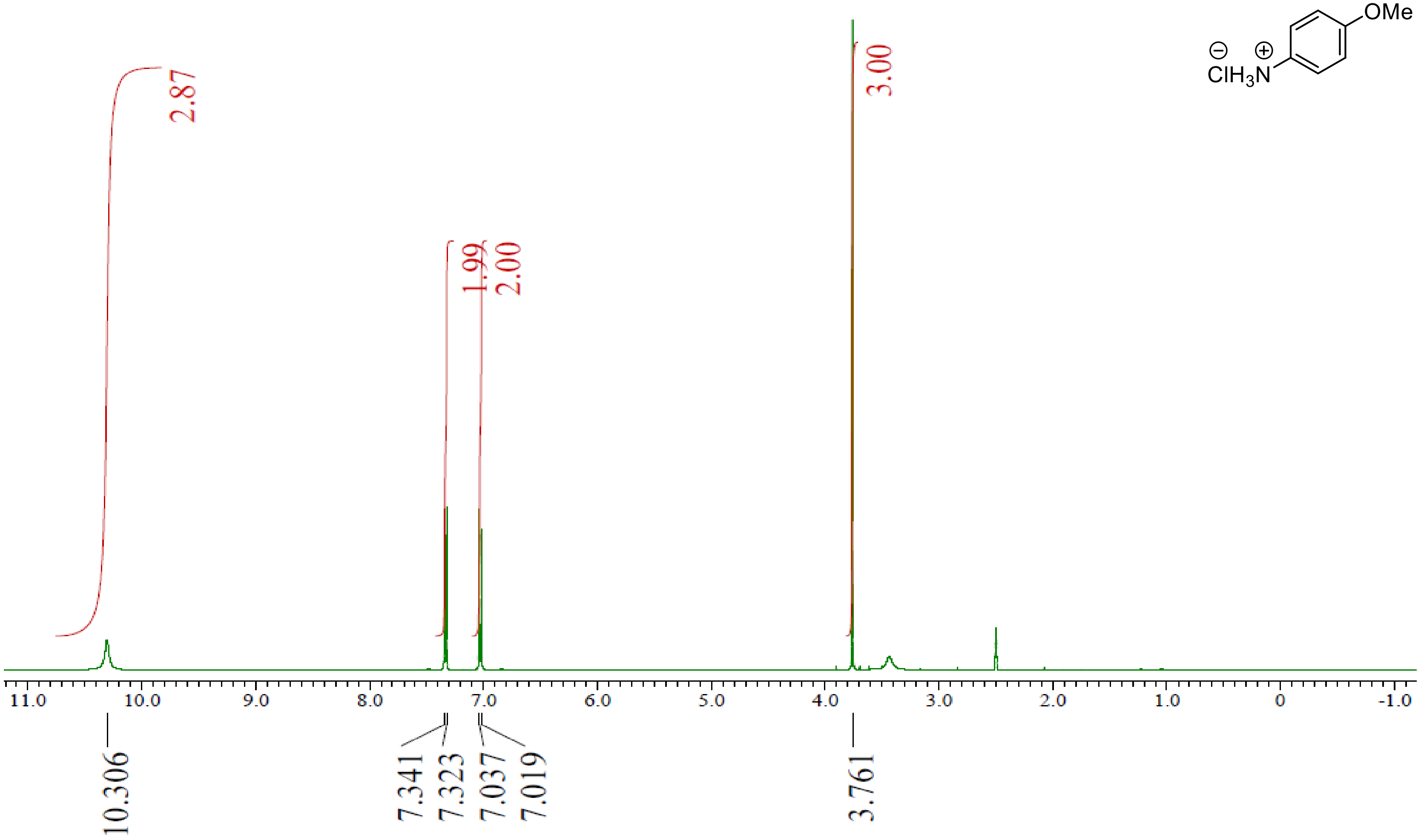

^{13}C NMR spectrum (126 MHz, DMSO-d_6) of 4-methoxyaniline hydrochloride (**3h•HCl**).

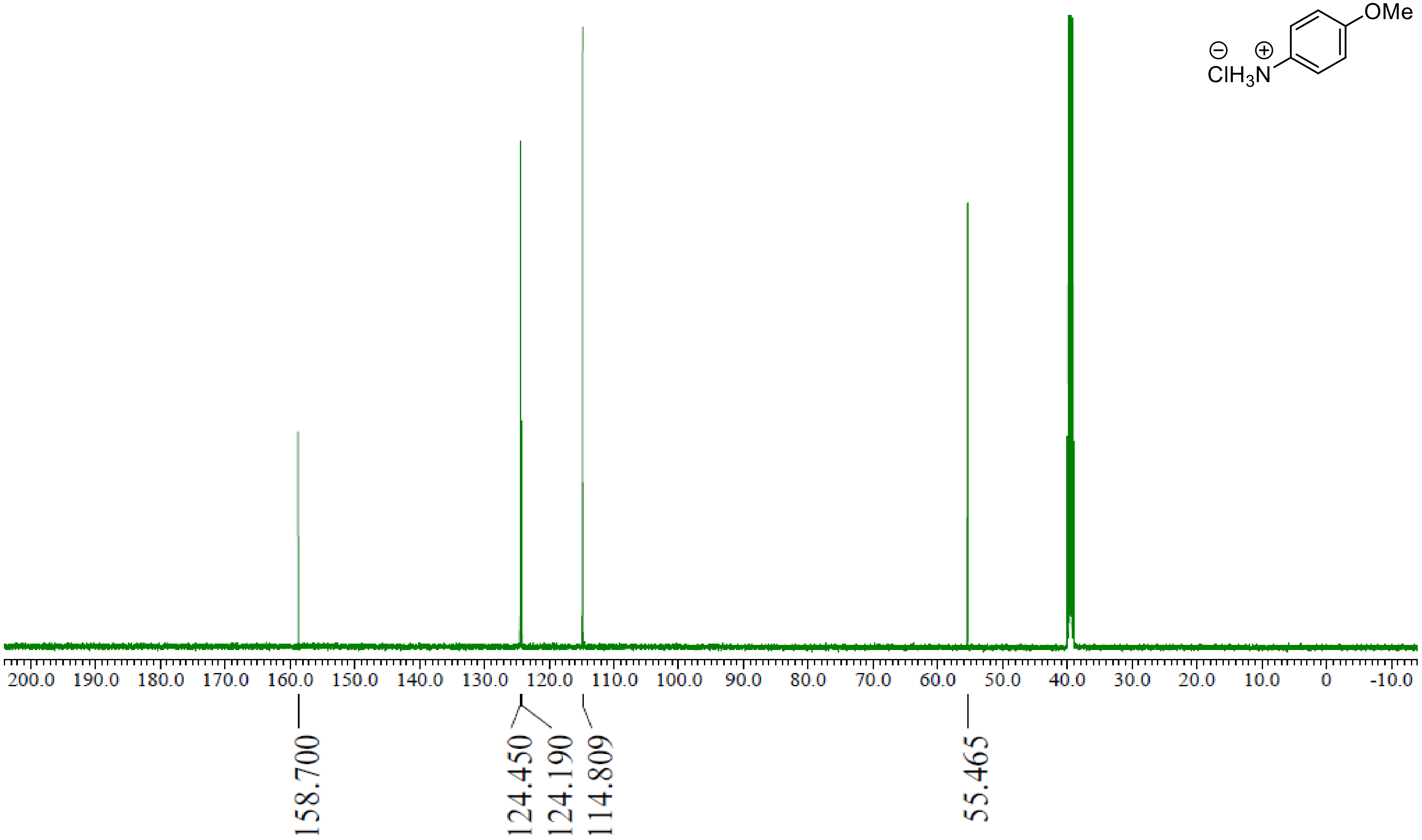

^{1}H NMR spectrum (500 MHz, $CDCl_3$) of *N*-(4-dimethylaminophenyl)formamide (**2i**). Two tautomers were observed in approximately 0.5:0.5 ratio.

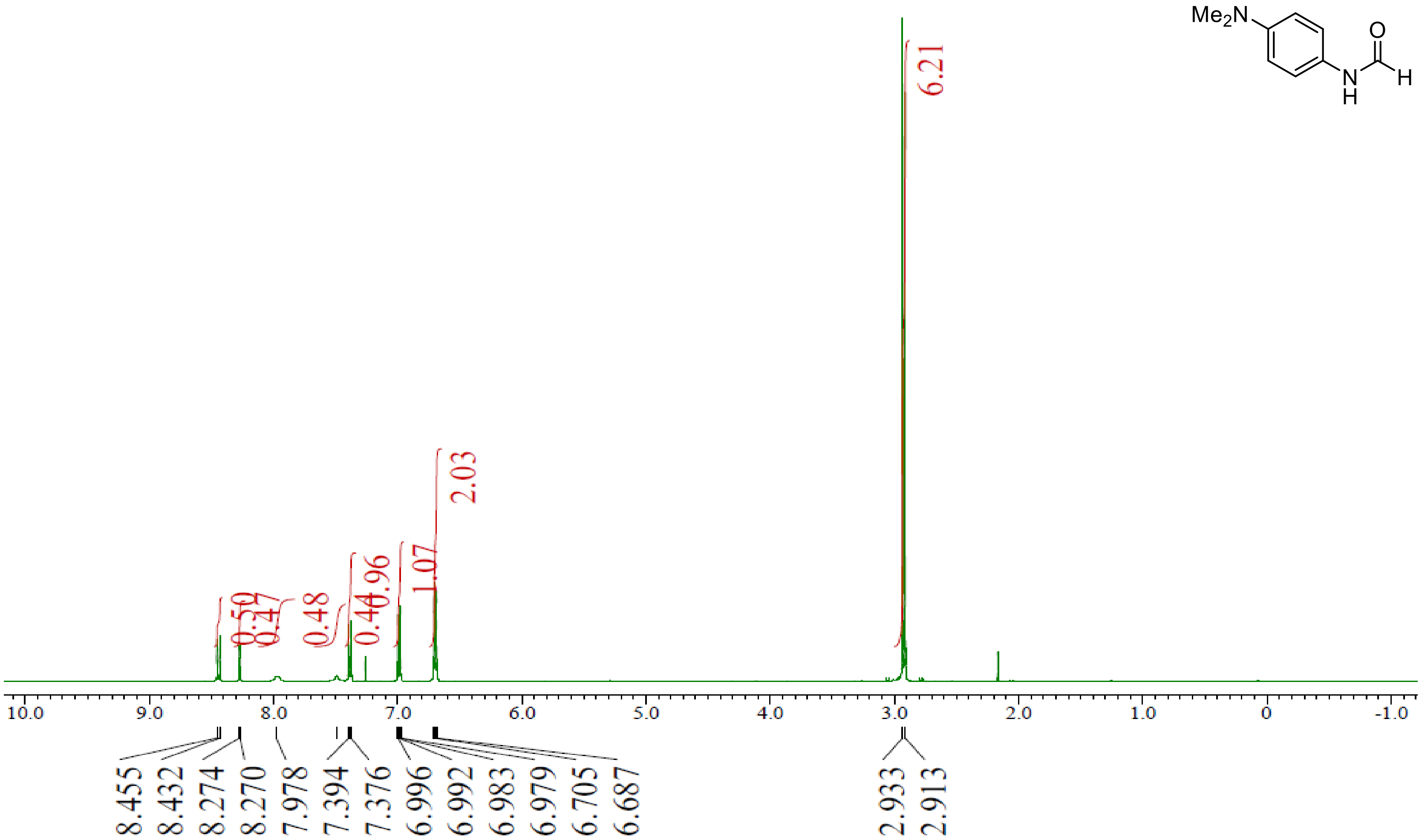

^{13}C NMR spectrum (126 MHz, $CDCl_3$) of *N*-(4-dimethylaminophenyl)formamide (**2i**). Two tautomers were observed in approximately 0.5:0.5 ratio.

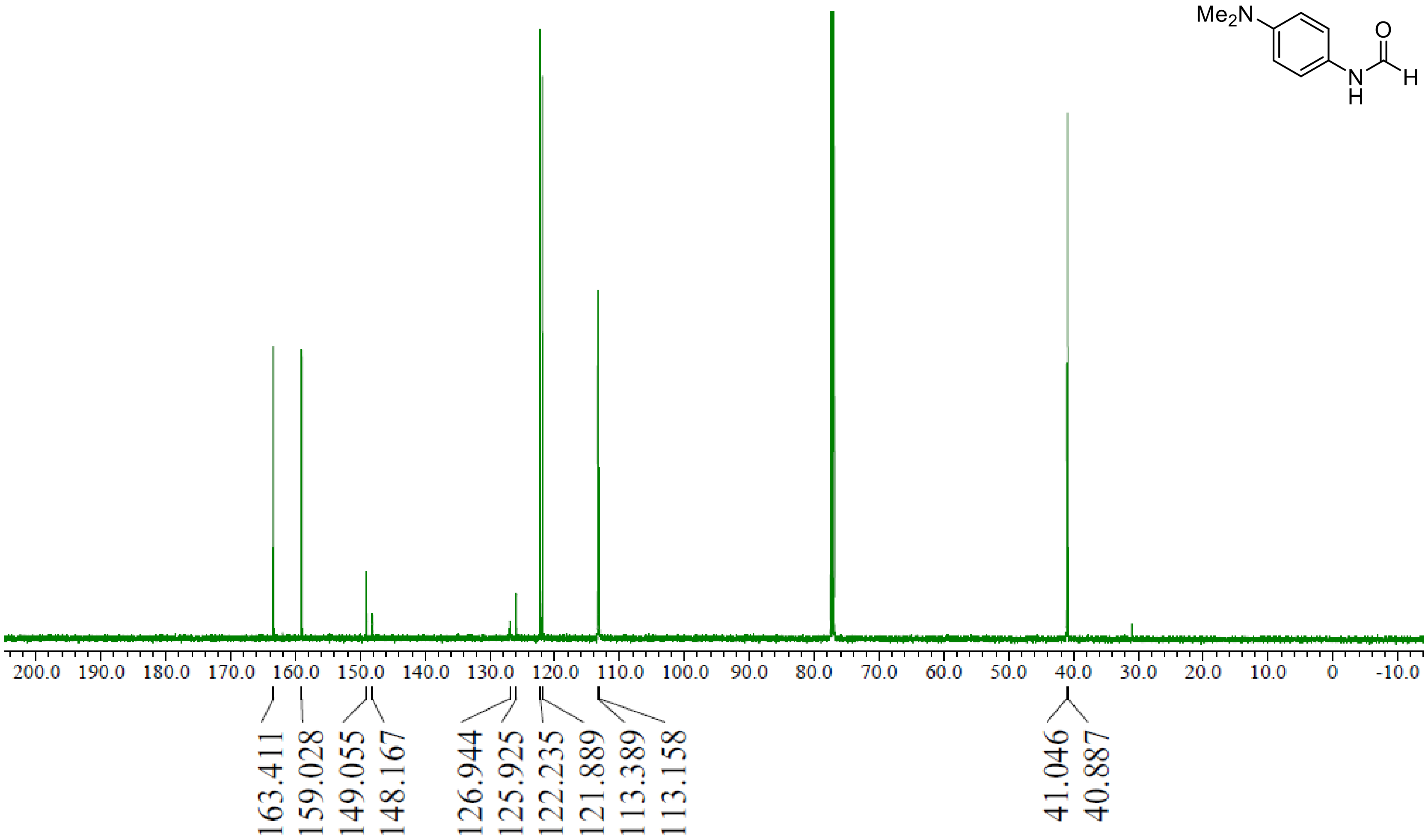

^{1}H NMR spectrum (500 MHz, $CDCl_3$) of 4-dimethylaminoaniline (**3i**).

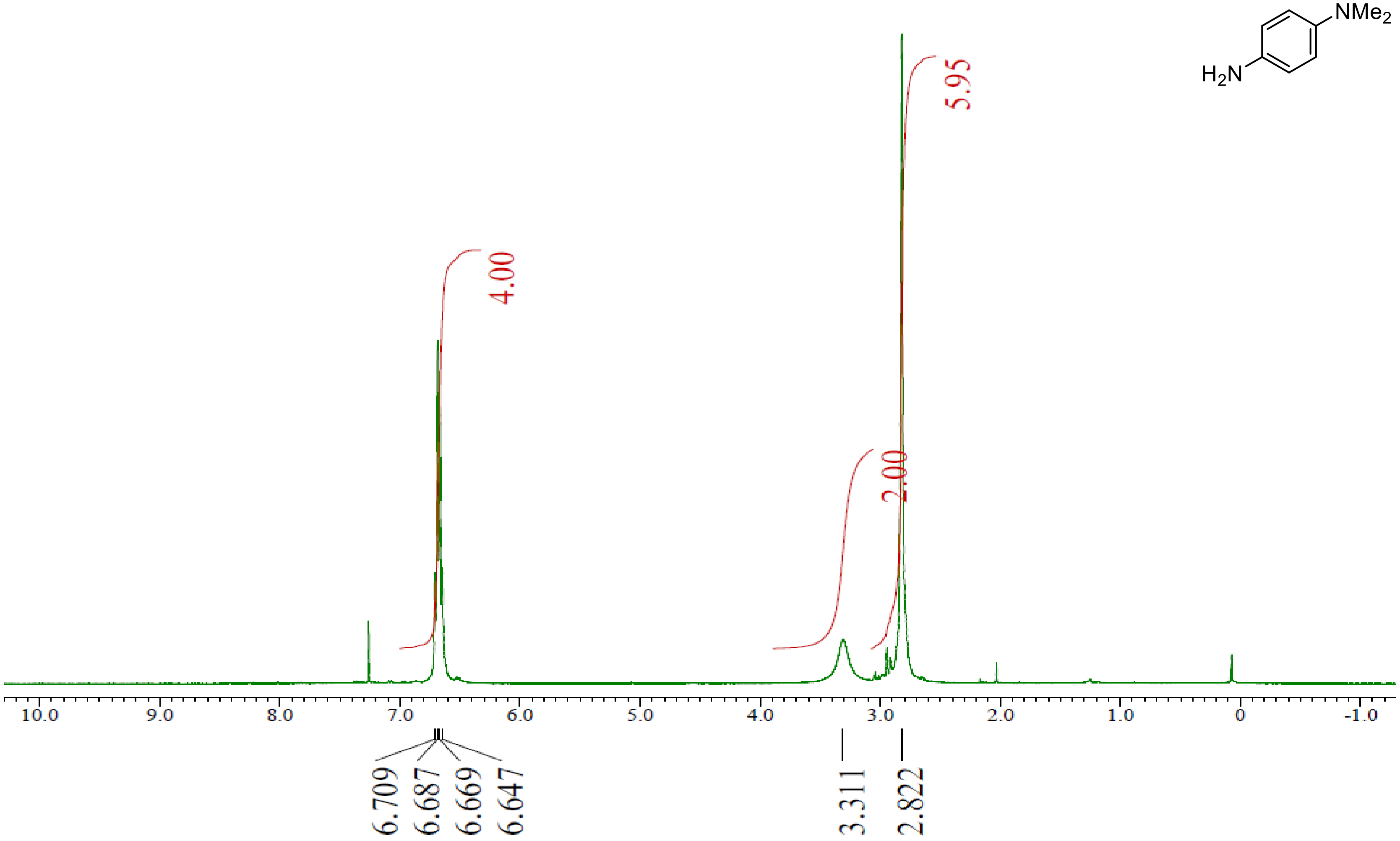

^{13}C NMR spectrum (126 MHz, $CDCl_3$) of 4-dimethylaminoaniline (**3i**).

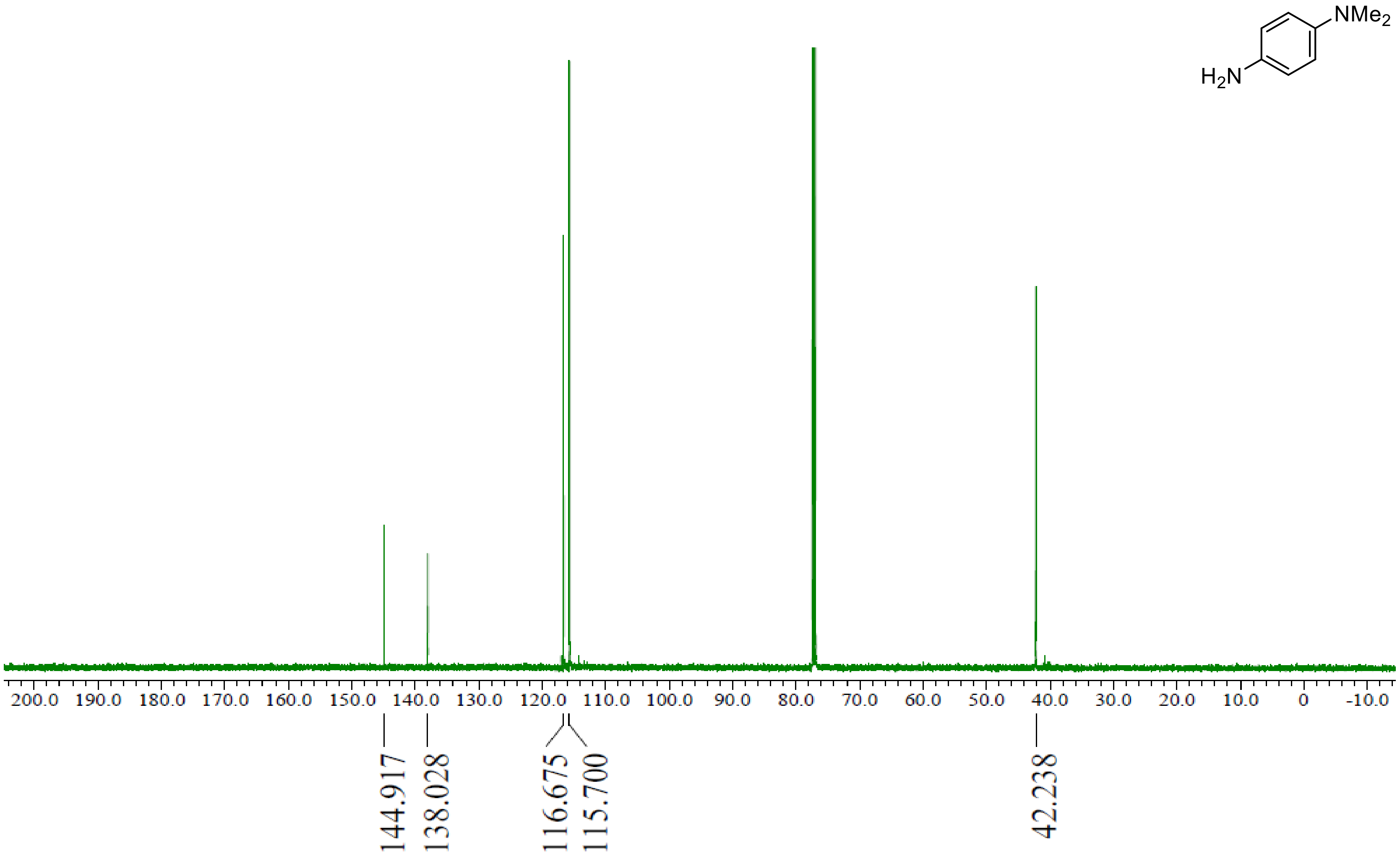

^{1}H NMR spectrum (500 MHz, $CDCl_3$) of ethyl 4-formamidobenzoate (**2j**). Two tautomers were observed in approximately 0.44:0.56 ratio.

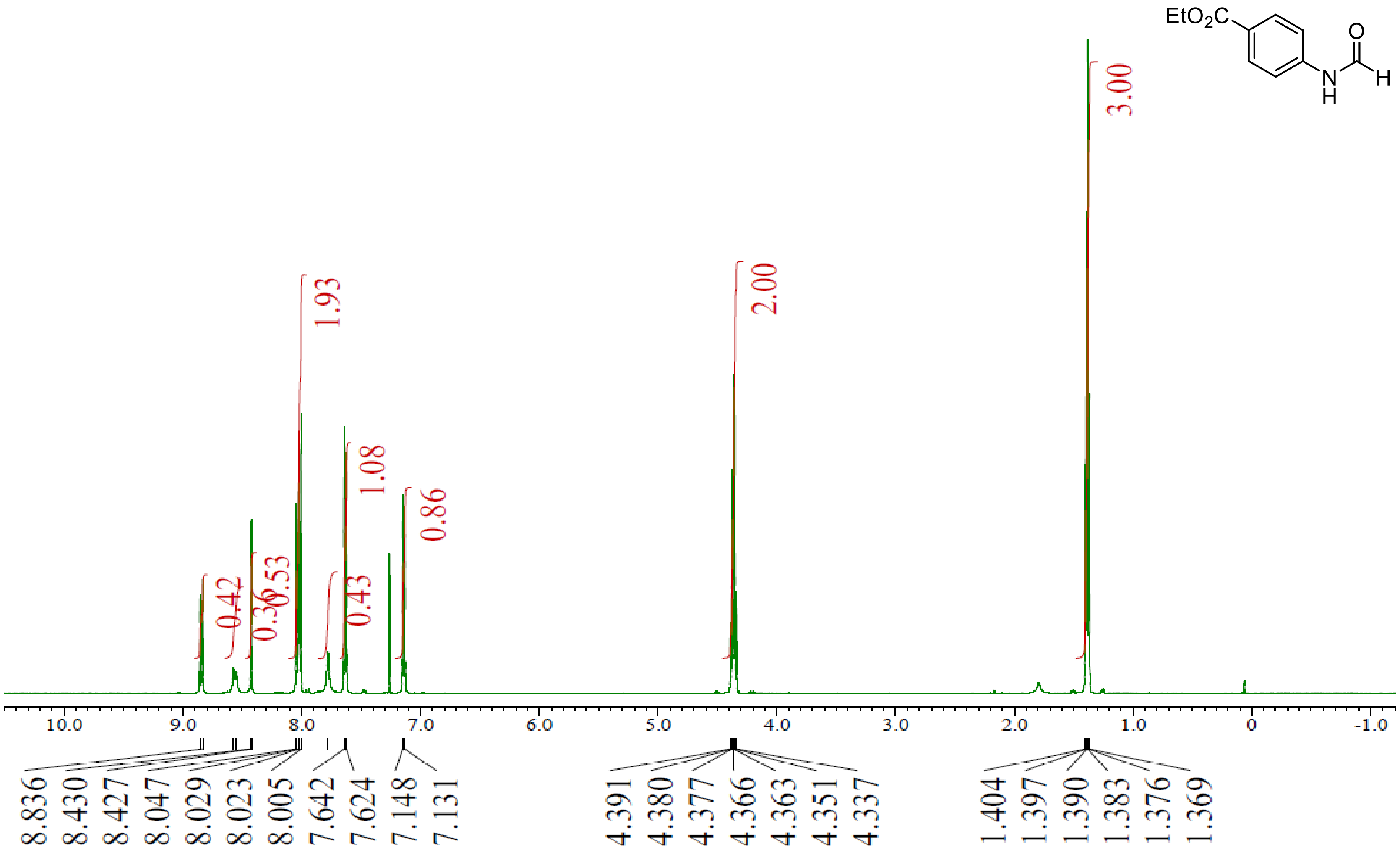

^{13}C NMR spectrum (126 MHz, $CDCl_3$) of ethyl 4-formamidobenzoate (**2j**). Two tautomers were observed in approximately 0.44:0.56 ratio.

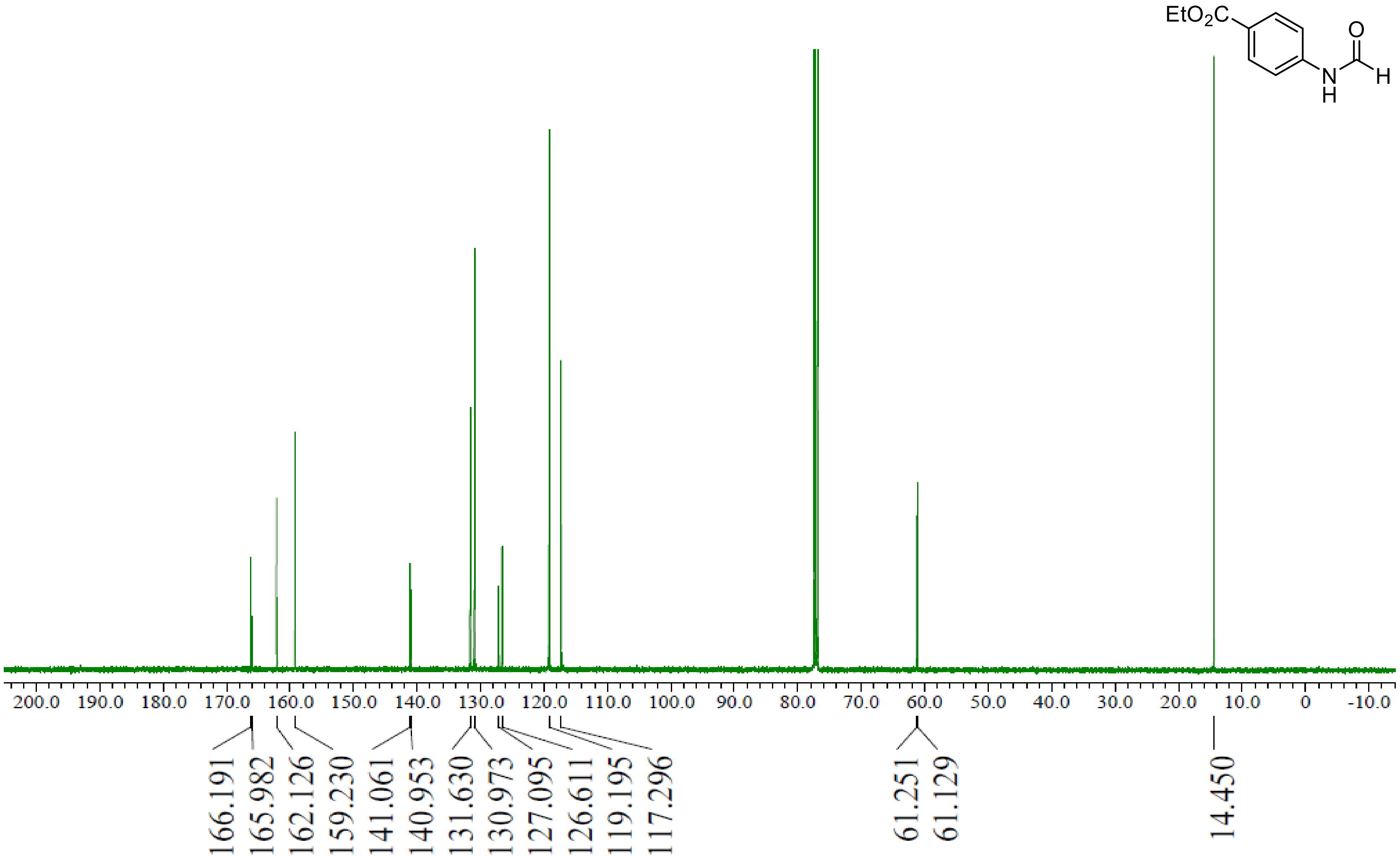

^{1}H NMR spectrum (500 MHz, $CDCl_3$) of ethyl 4-aminobenzoate (**3j**).

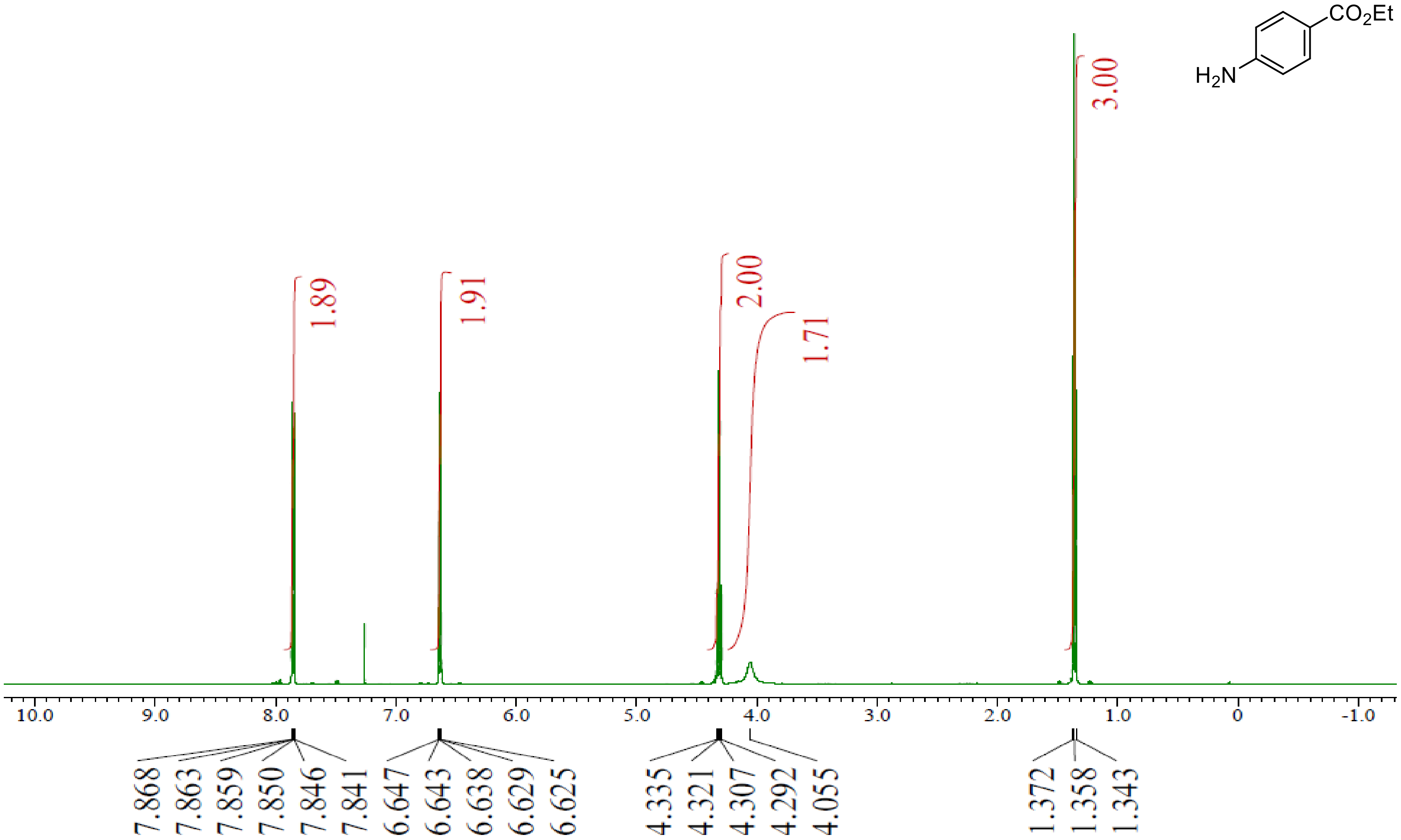

^{13}C NMR spectrum (126 MHz, $CDCl_3$) of ethyl 4-aminobenzoate (**3j**).

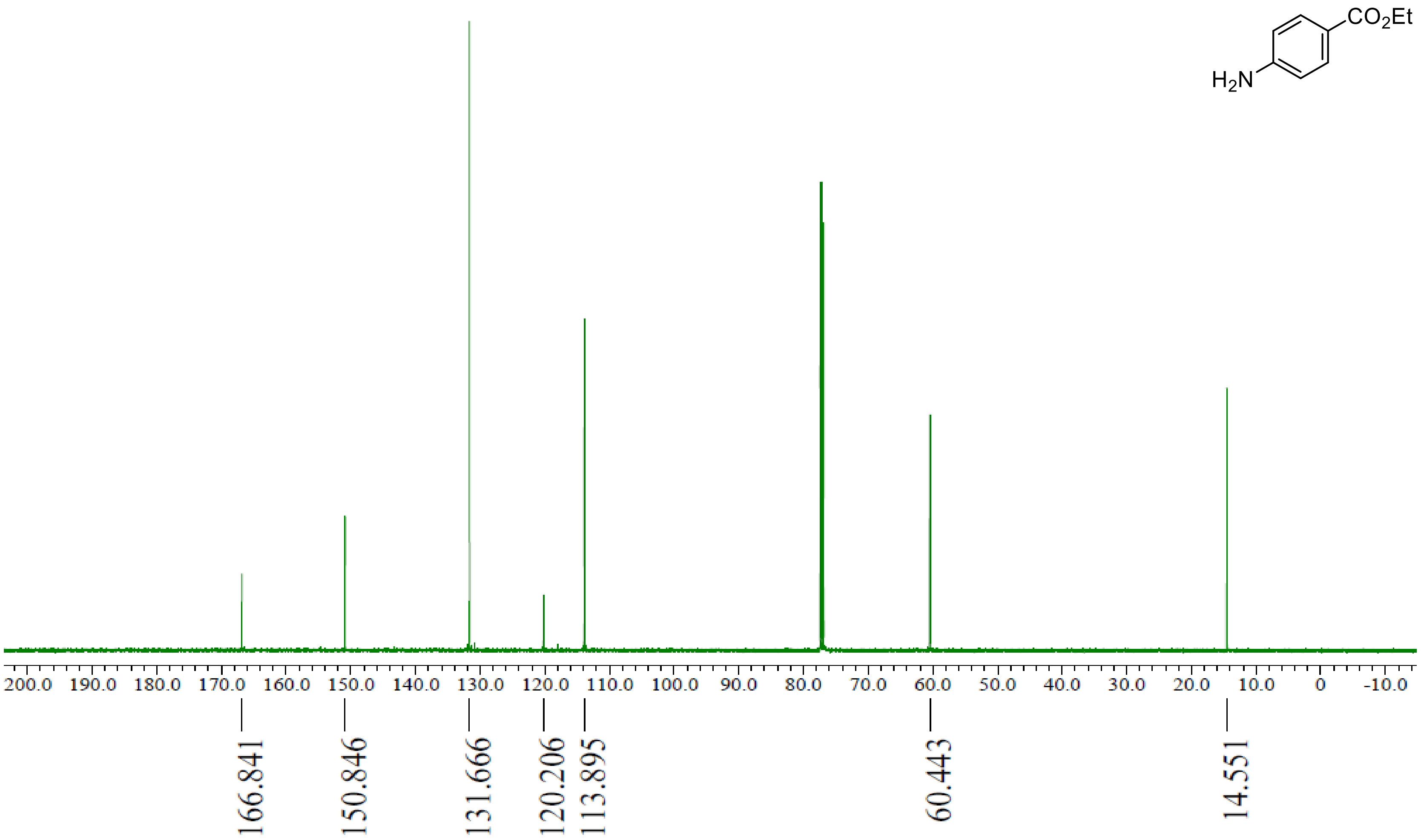

^{1}H NMR spectrum (500 MHz, $CDCl_3$) of 4-formamido-*N*,*N*-dipropylbenzamide (**2k**). Two tautomers were observed in approximately 0.3:0.7 ratio.

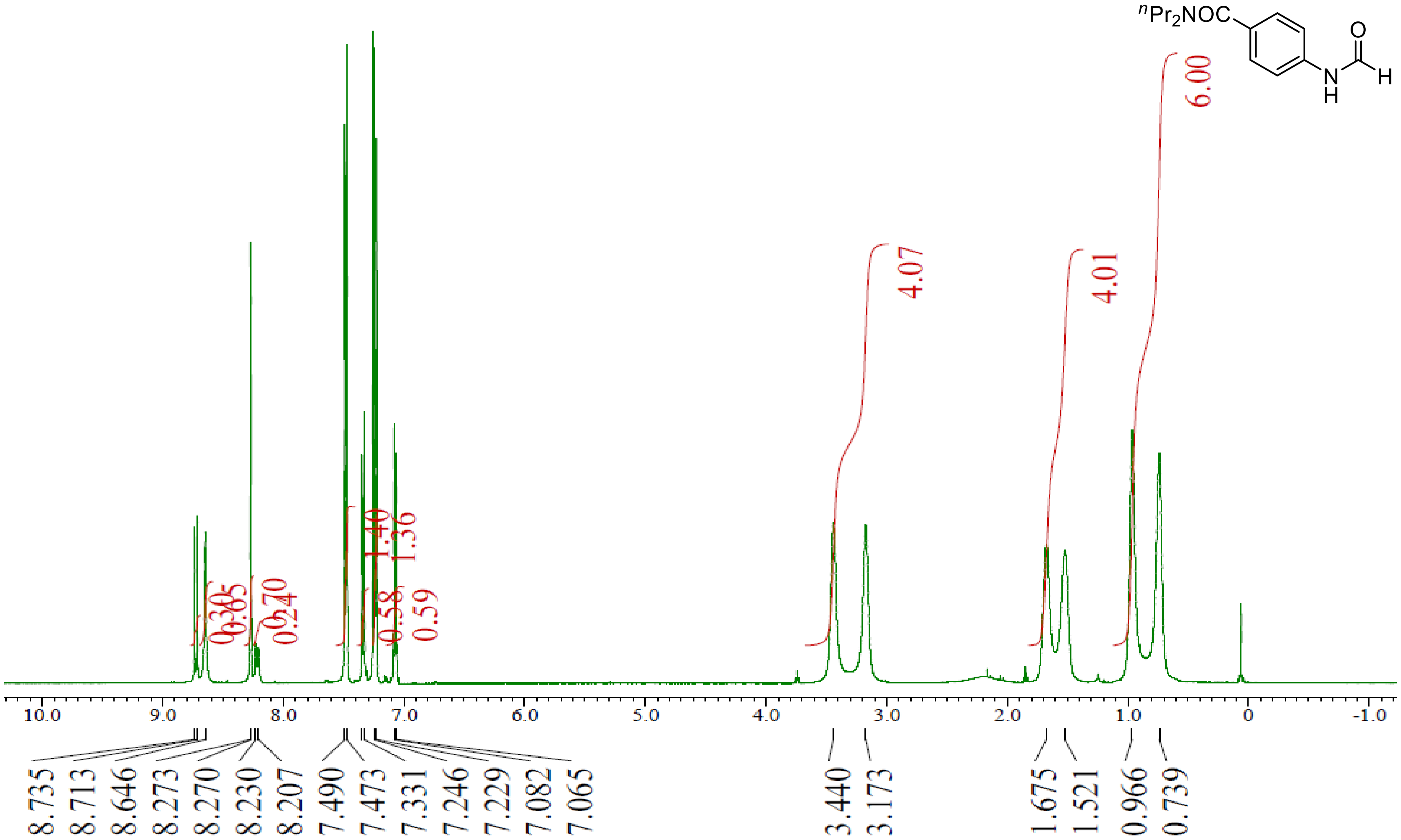

^{13}C NMR spectrum (126 MHz, $CDCl_3$) of 4-formamido-*N*,*N*-dipropylbenzamide (**2k**). Two tautomers were observed in approximately 0.3:0.7 ratio.

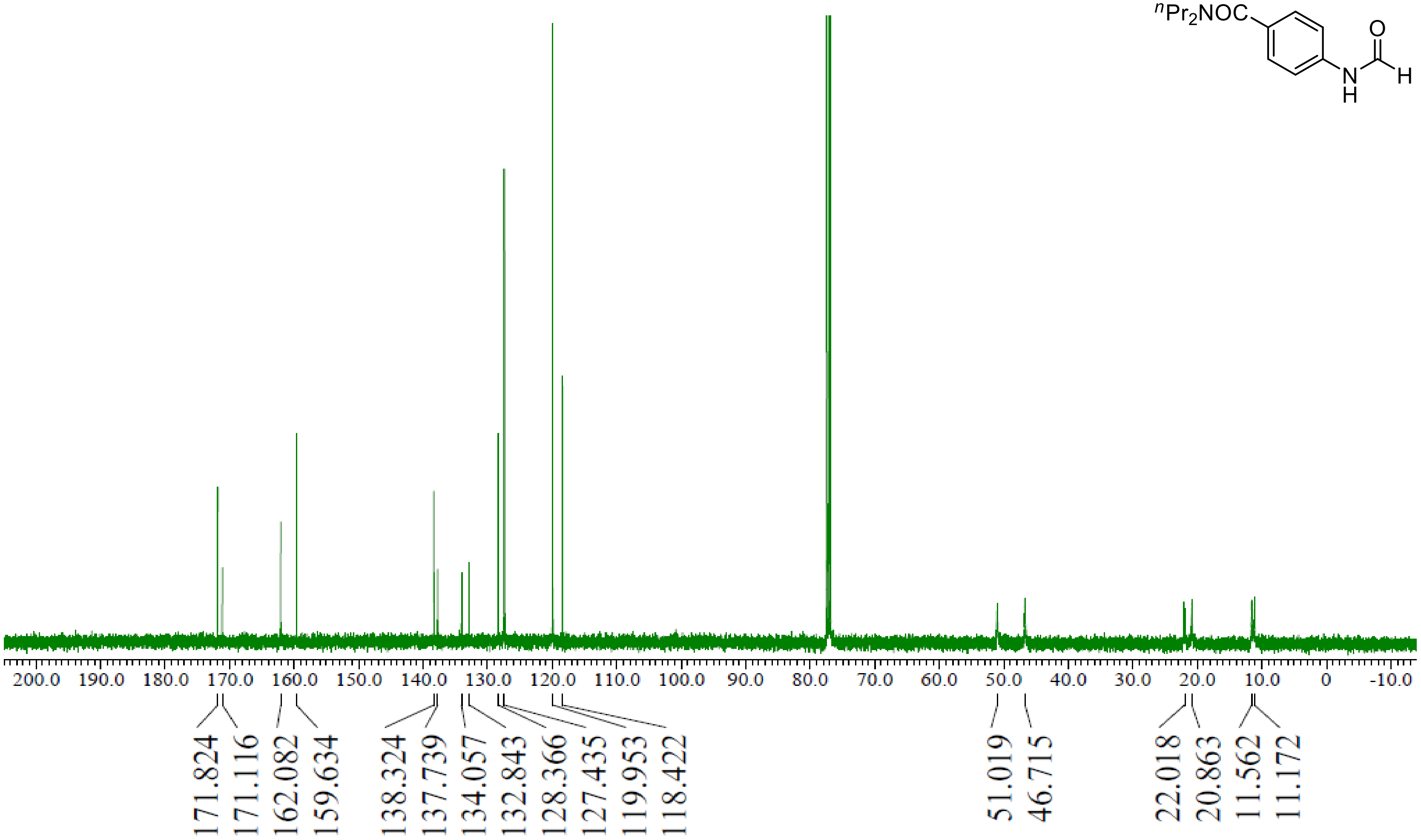

^{1}H NMR spectrum (500 MHz, $CDCl_3$) of 4-amino-*N*,*N*-dipropylbenzamide (**3k**).

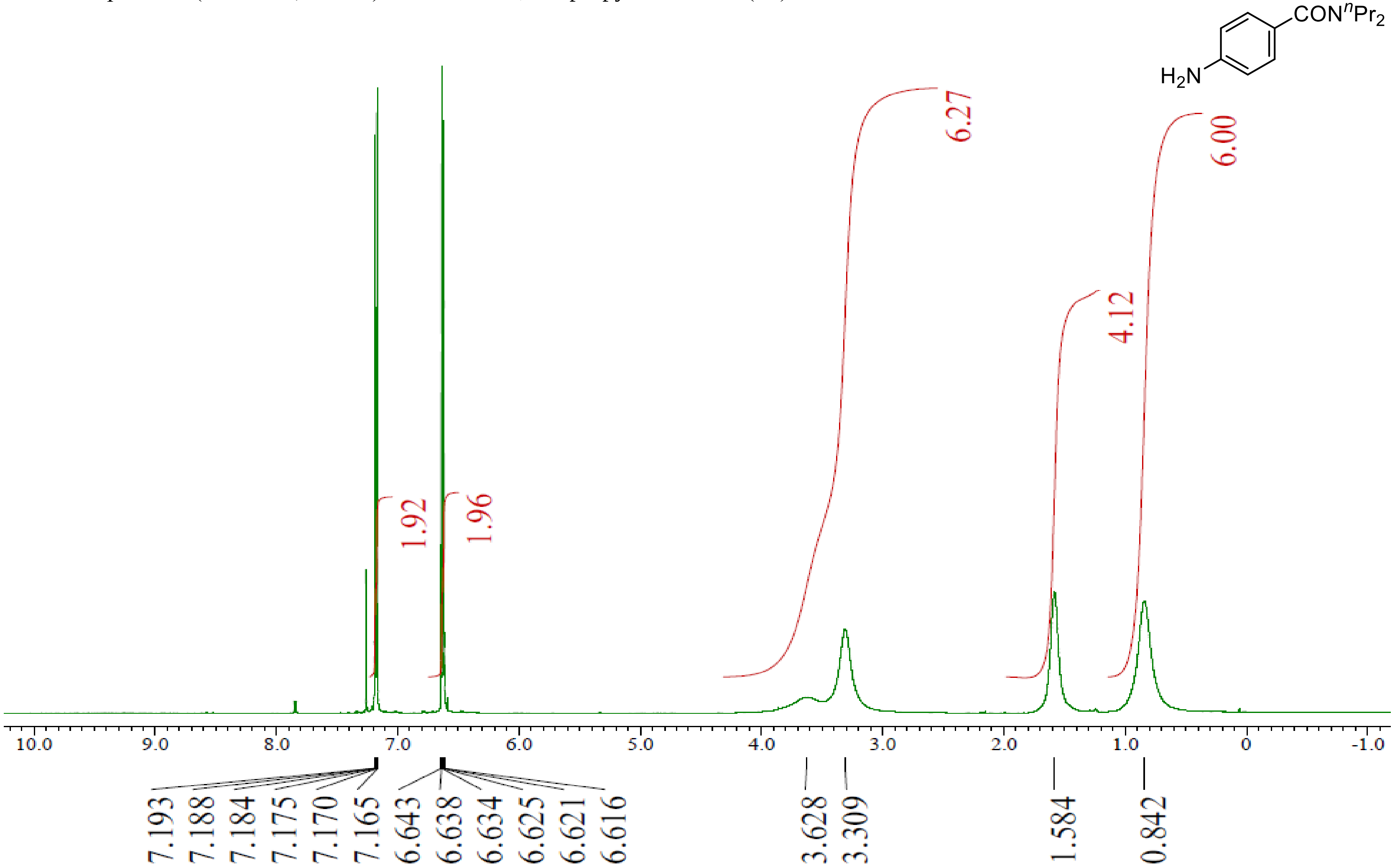

^{13}C NMR spectrum (126 MHz, $CDCl_3$) of 4-amino-*N*,*N*-dipropylbenzamide (**3k**).

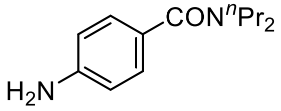

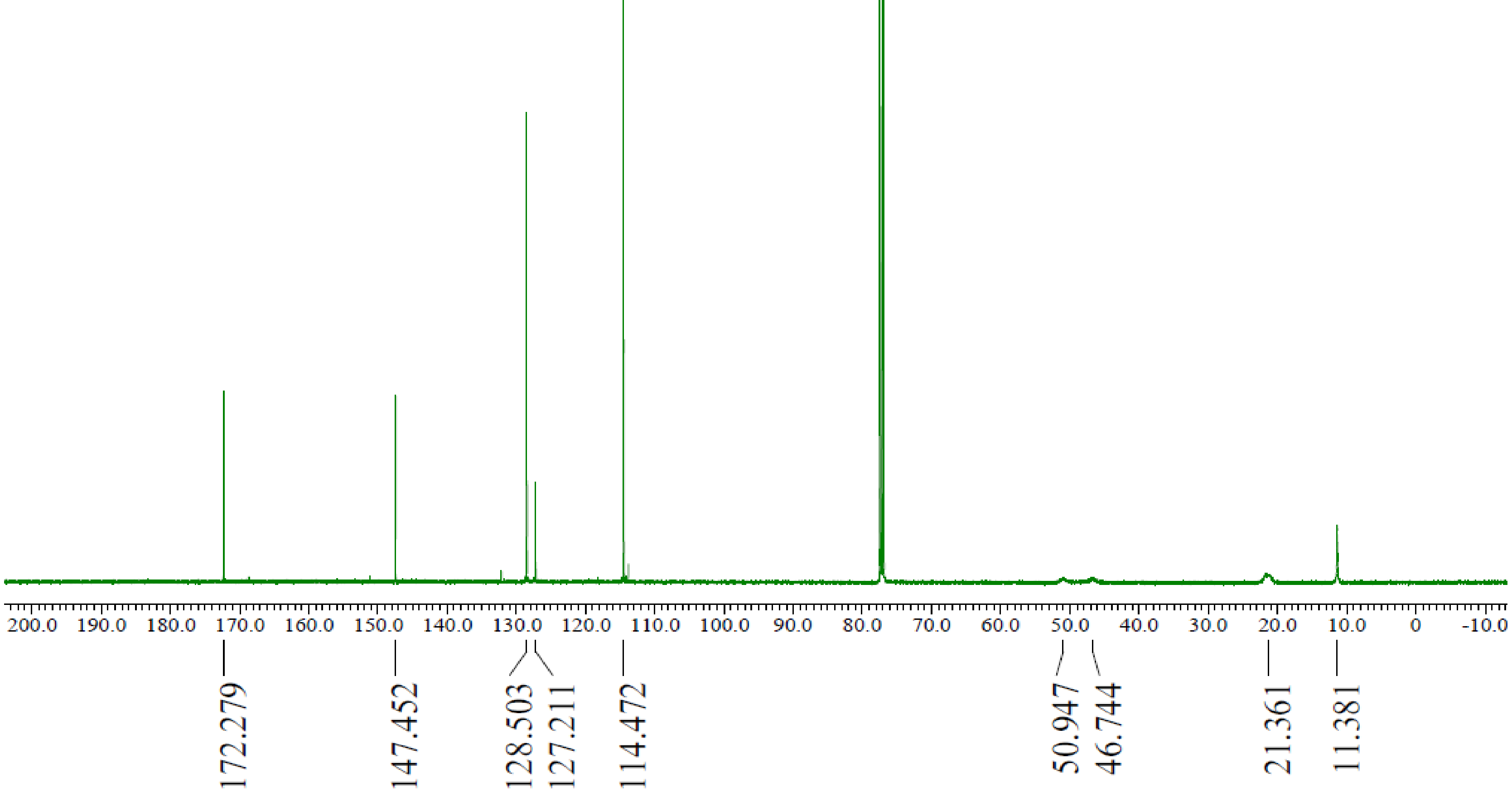

^{1}H NMR spectrum (500 MHz, DMSO-d_6) of *N*-(4-cyanophenyl)formamide (**2*l***). Two tautomers were observed in approximately 0.25:0.75 ratio.

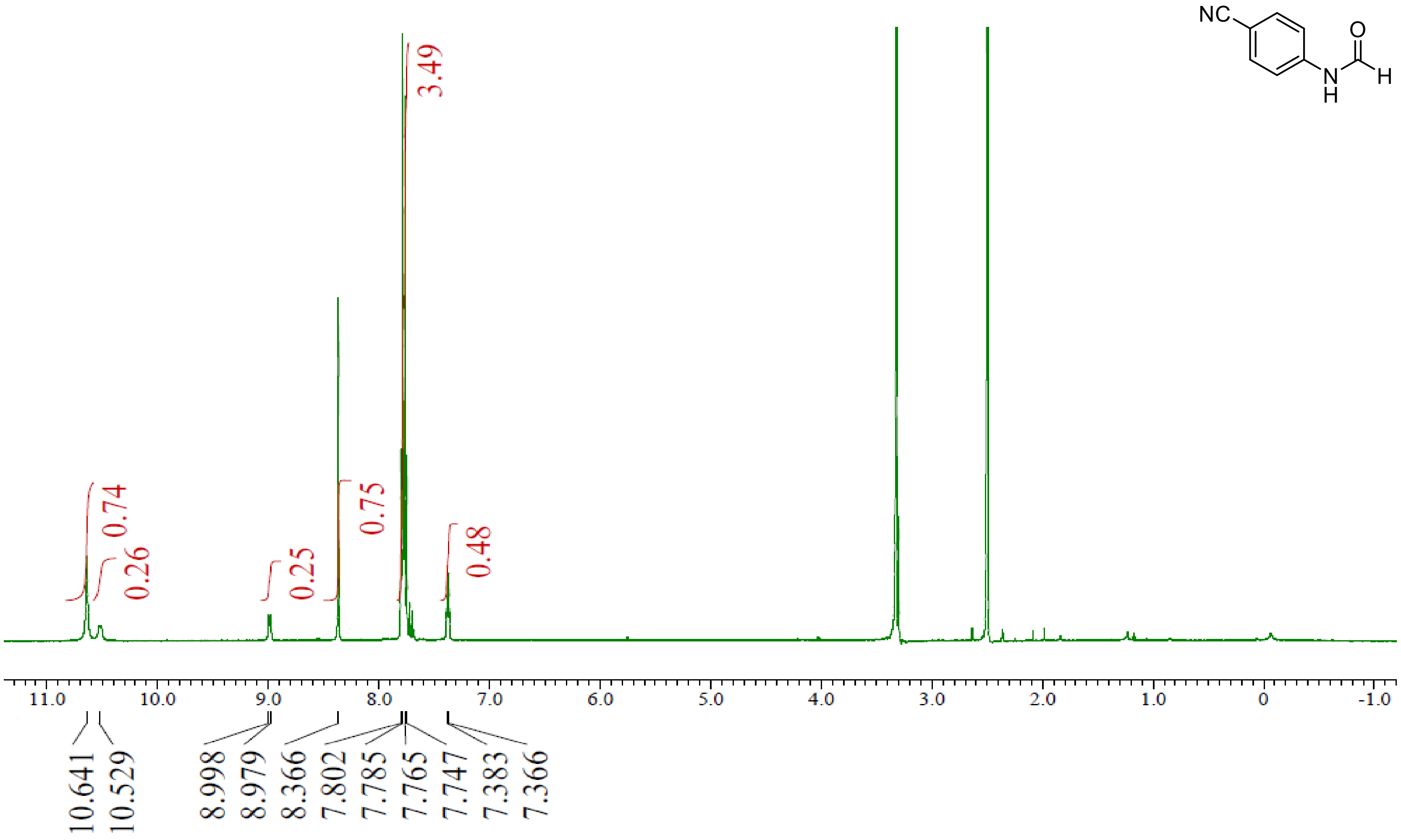

^{13}C NMR spectrum (126 MHz, DMSO-d_6) of *N*-(4-cyanophenyl)formamide (**2*l***). Two tautomers were observed in approximately 0.25:0.75 ratio.

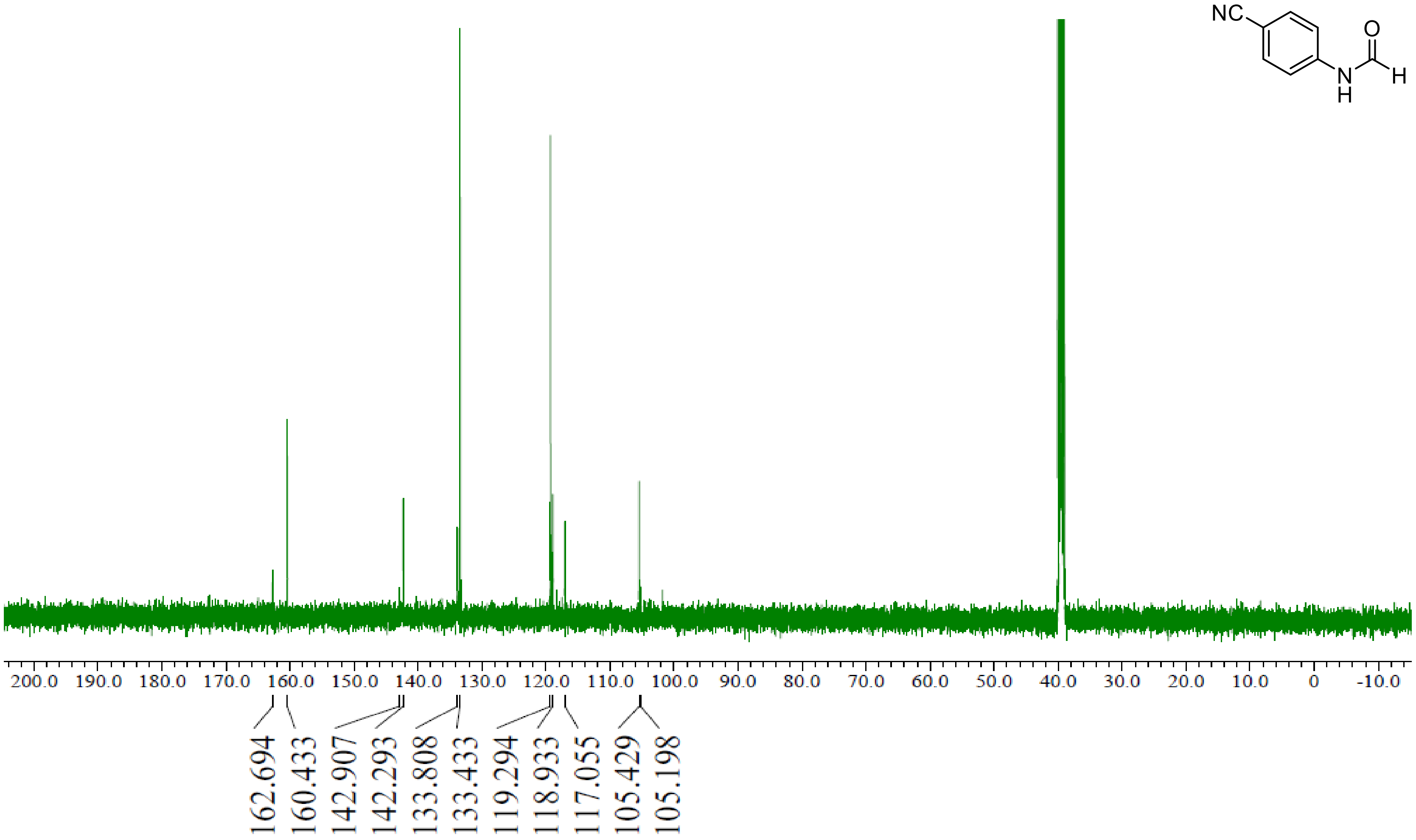

^{1}H NMR spectrum (500 MHz, $CDCl_3$) of 4-aminobenzonitrile (**3*l***).

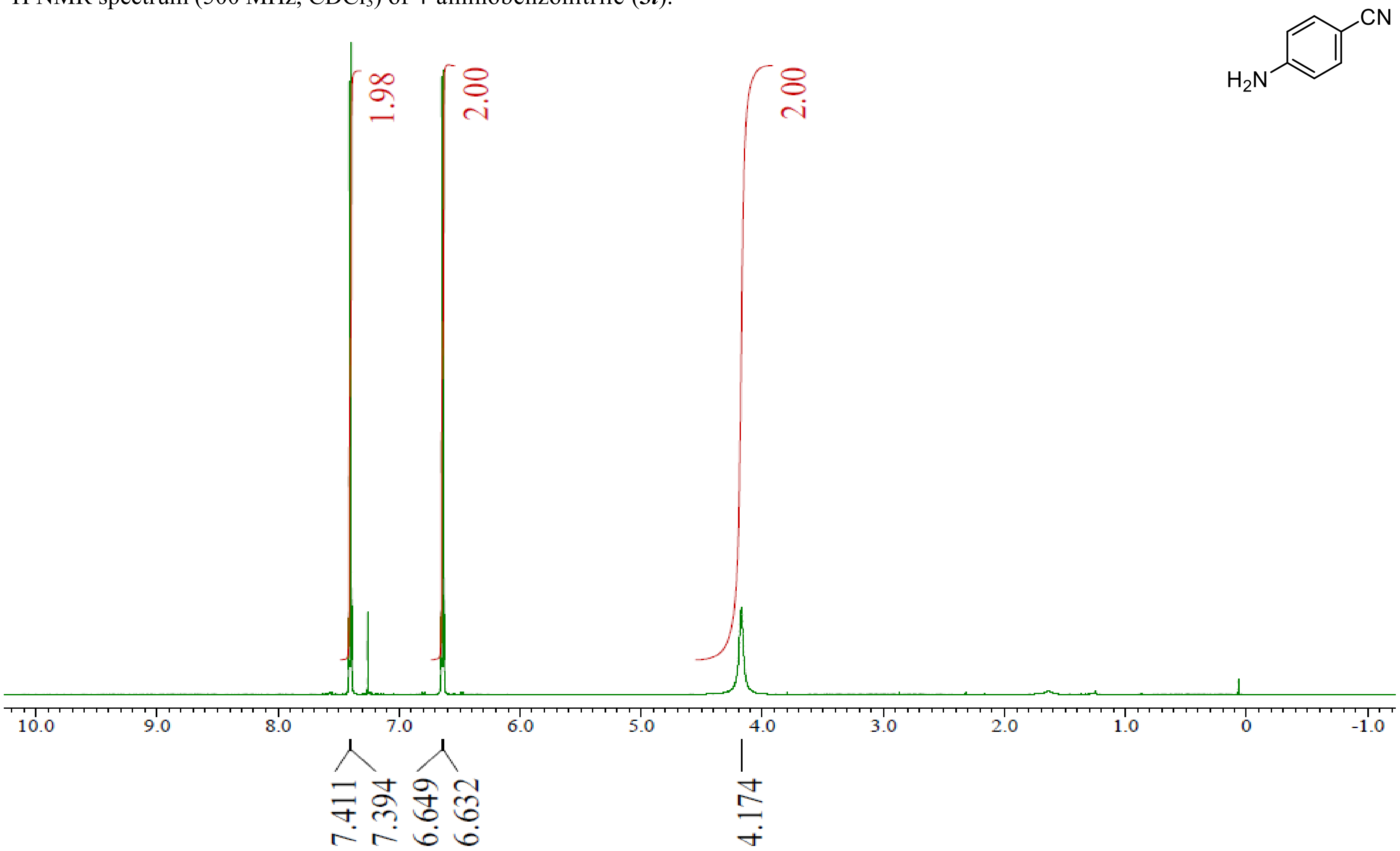

^{13}C NMR spectrum (126 MHz, $CDCl_3$) of 4-aminobenzonitrile (**3*l***).

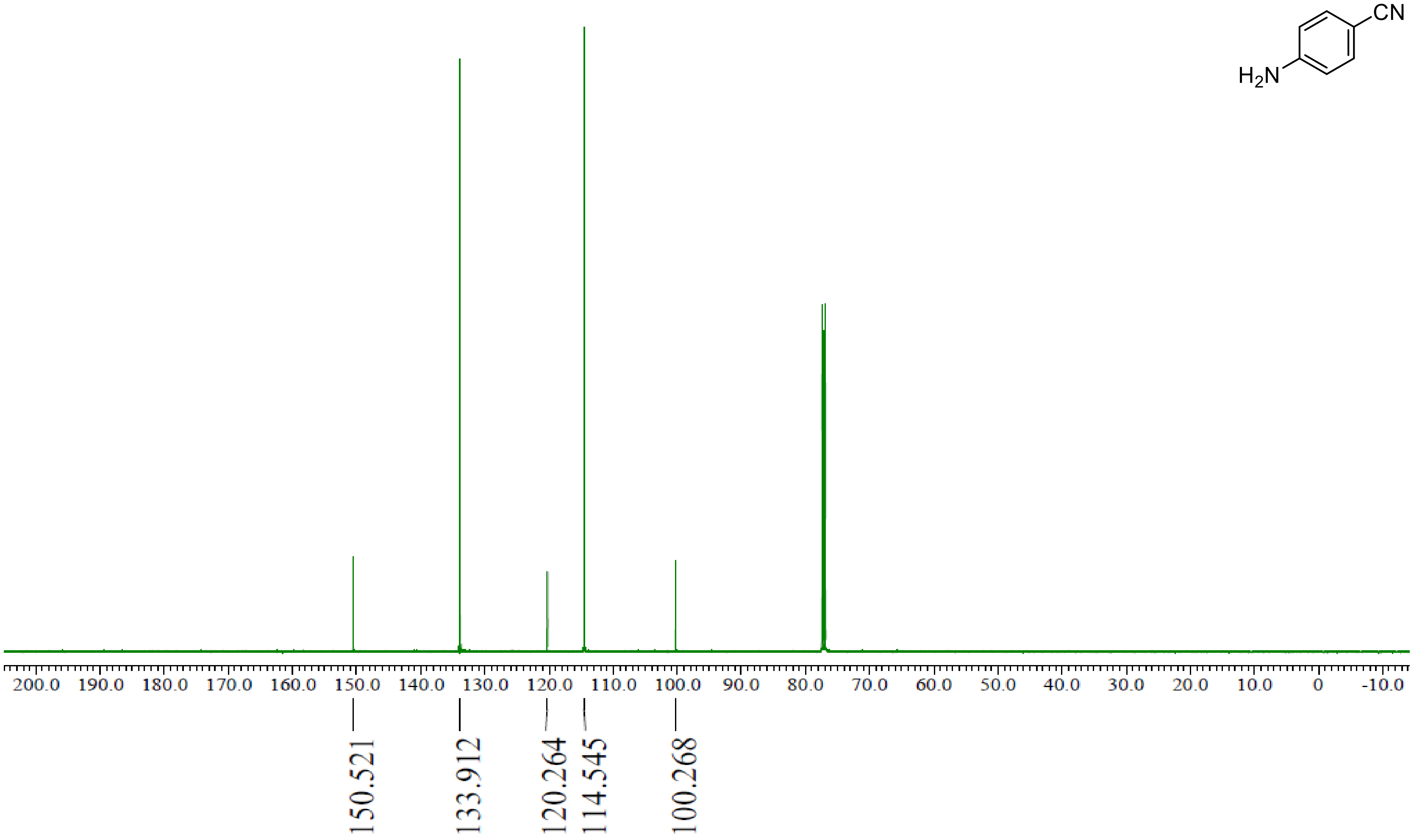

^{1}H NMR spectrum (500 MHz, $CDCl_3$) of *N*-benzylformamide (**2m**). Two tautomers were observed in approximately 0.15:0.85 ratio.

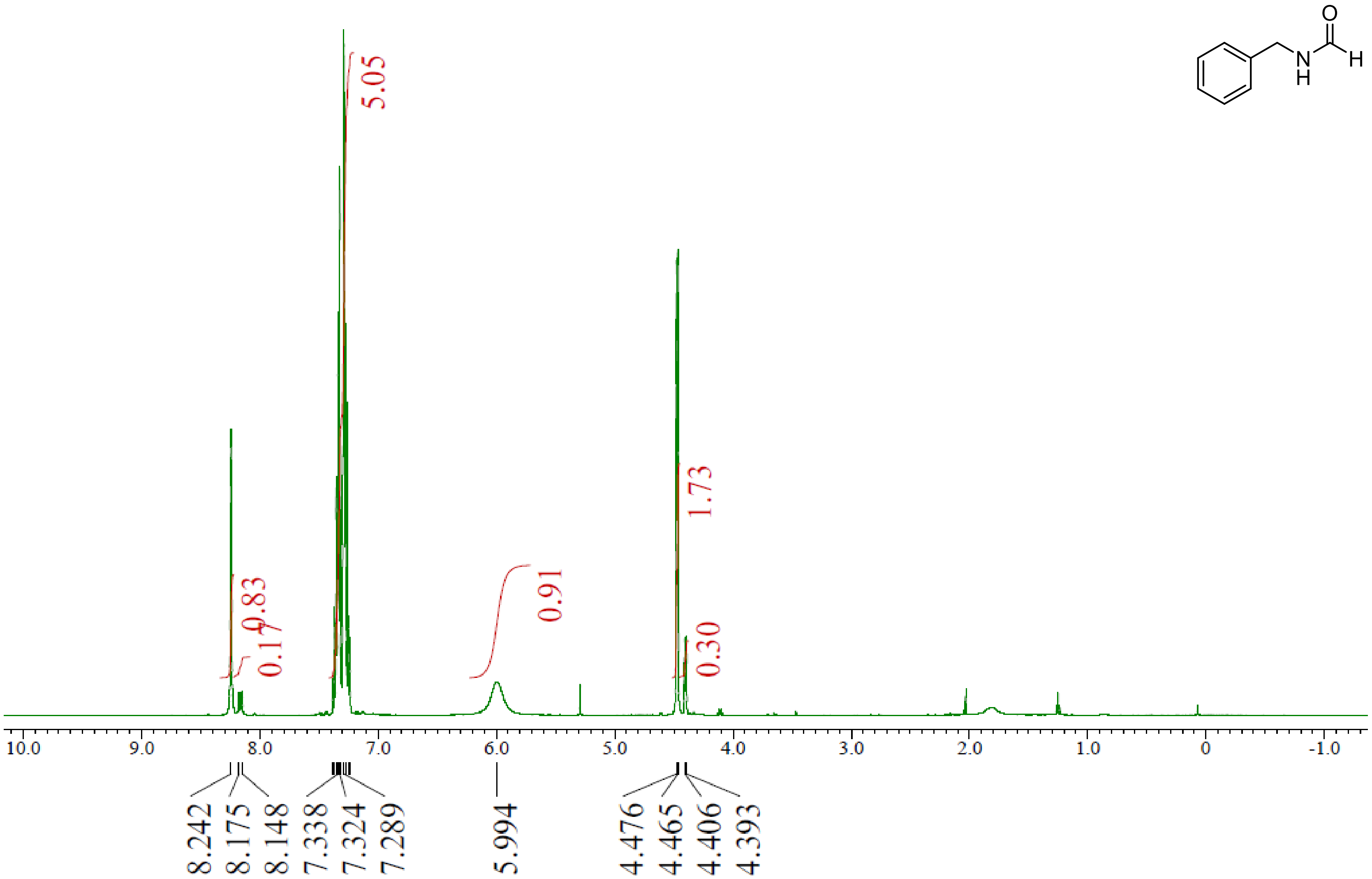

^{13}C NMR spectrum (126 MHz, $CDCl_3$) of *N*-benzylformamide (**2m**). Two tautomers were observed in approximately 0.15:0.85 ratio.

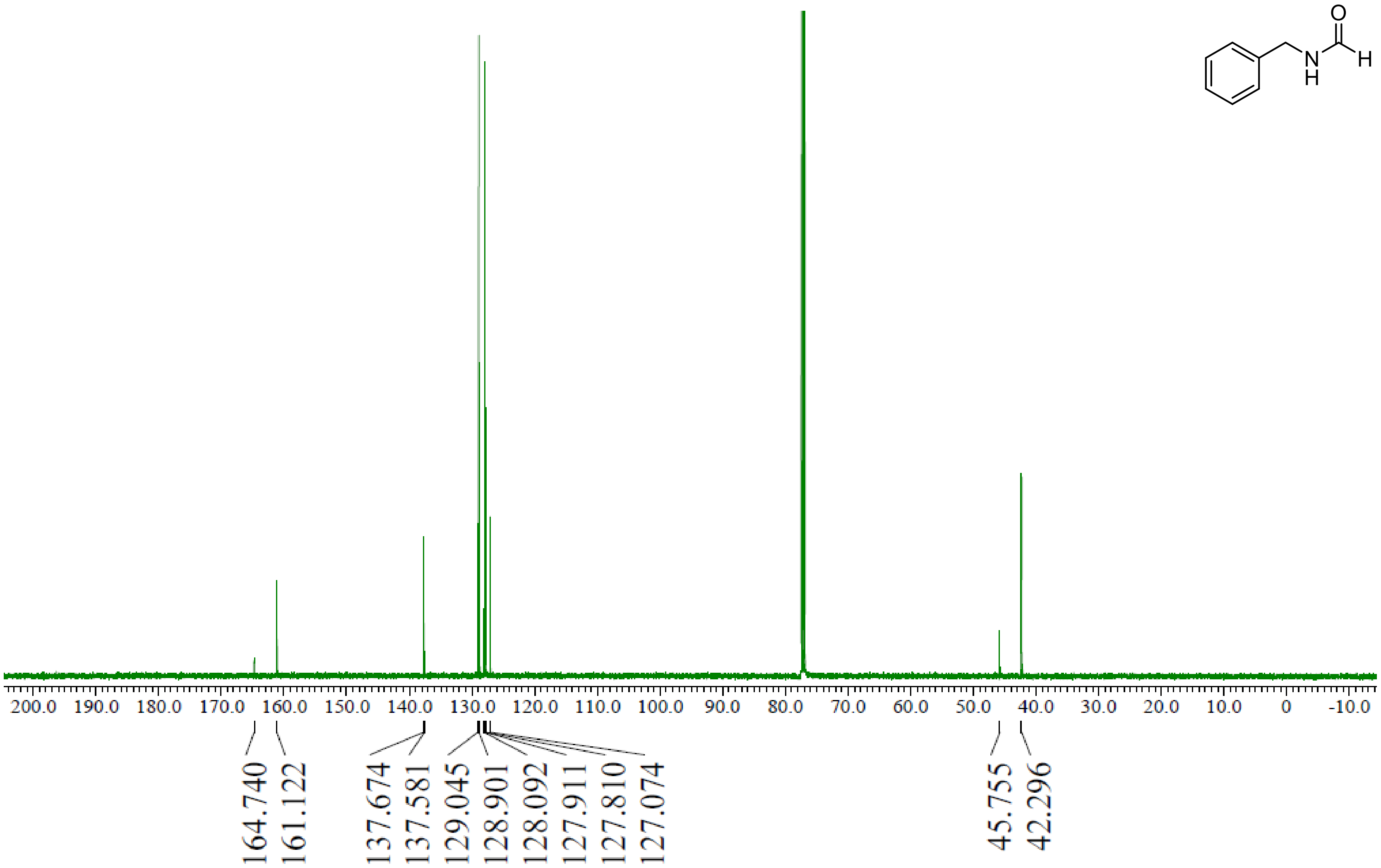

^{1}H NMR spectrum (500 MHz, DMSO-d_6) of benzylamine hydrochloride (**3m•HCl**).

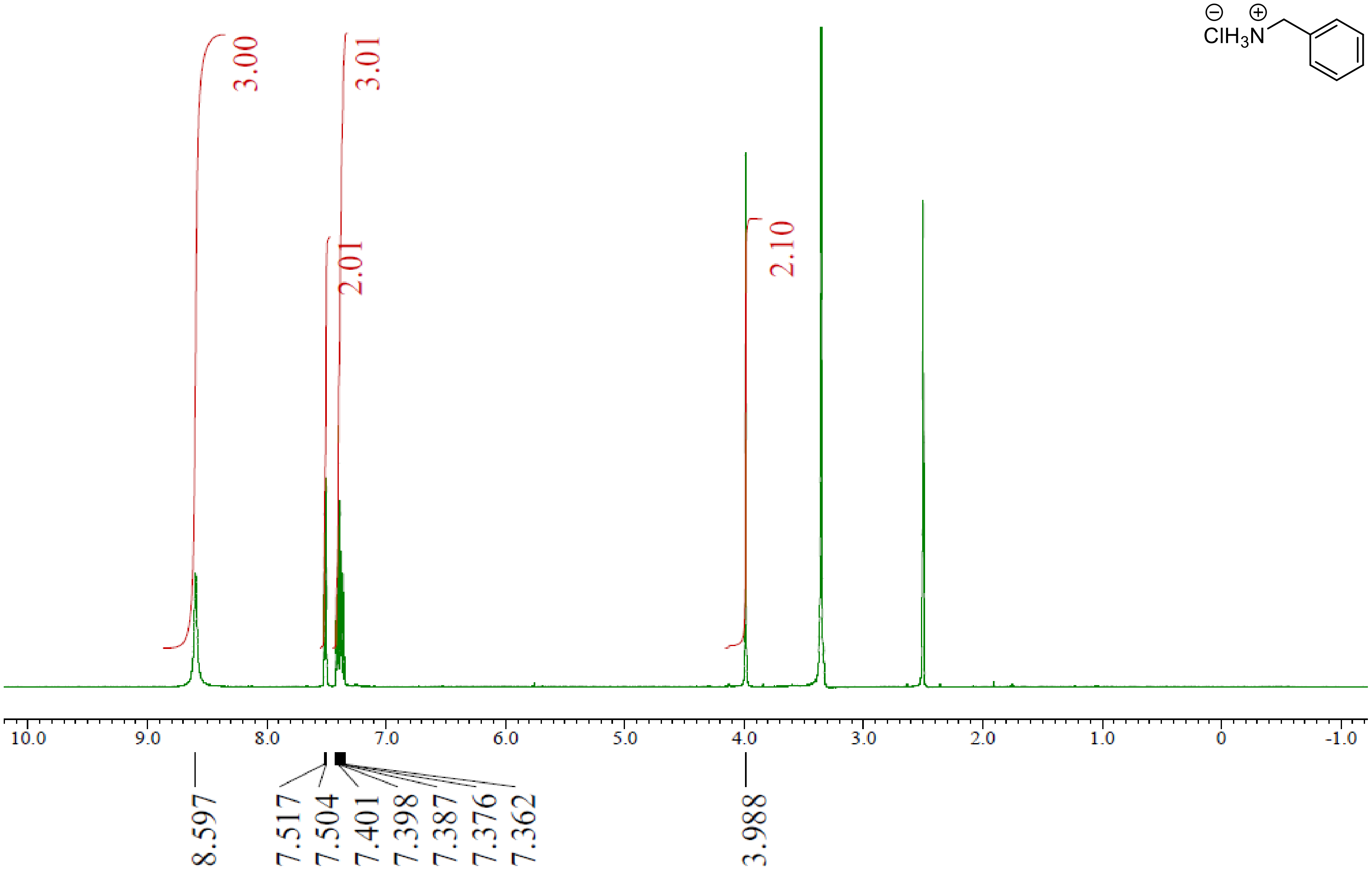

^{13}C NMR spectrum (126 MHz, DMSO-d_6) of benzylamine hydrochloride (**3m•HCl**).

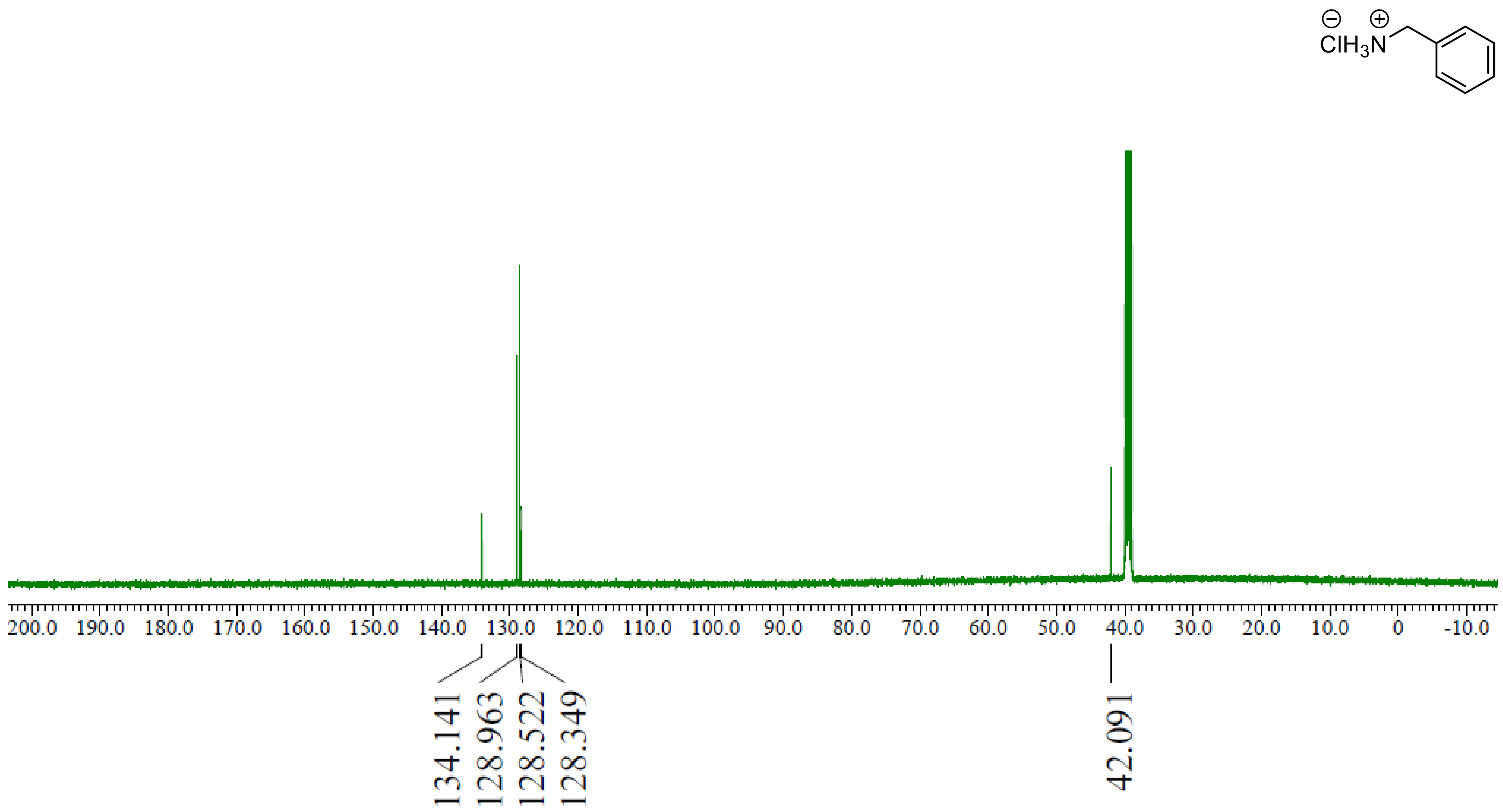

^{1}H NMR spectrum (500 MHz, $CDCl_3$) of formanilide (**2a**). Two tautomers were observed in approximately 0.5:0.5 ratio.

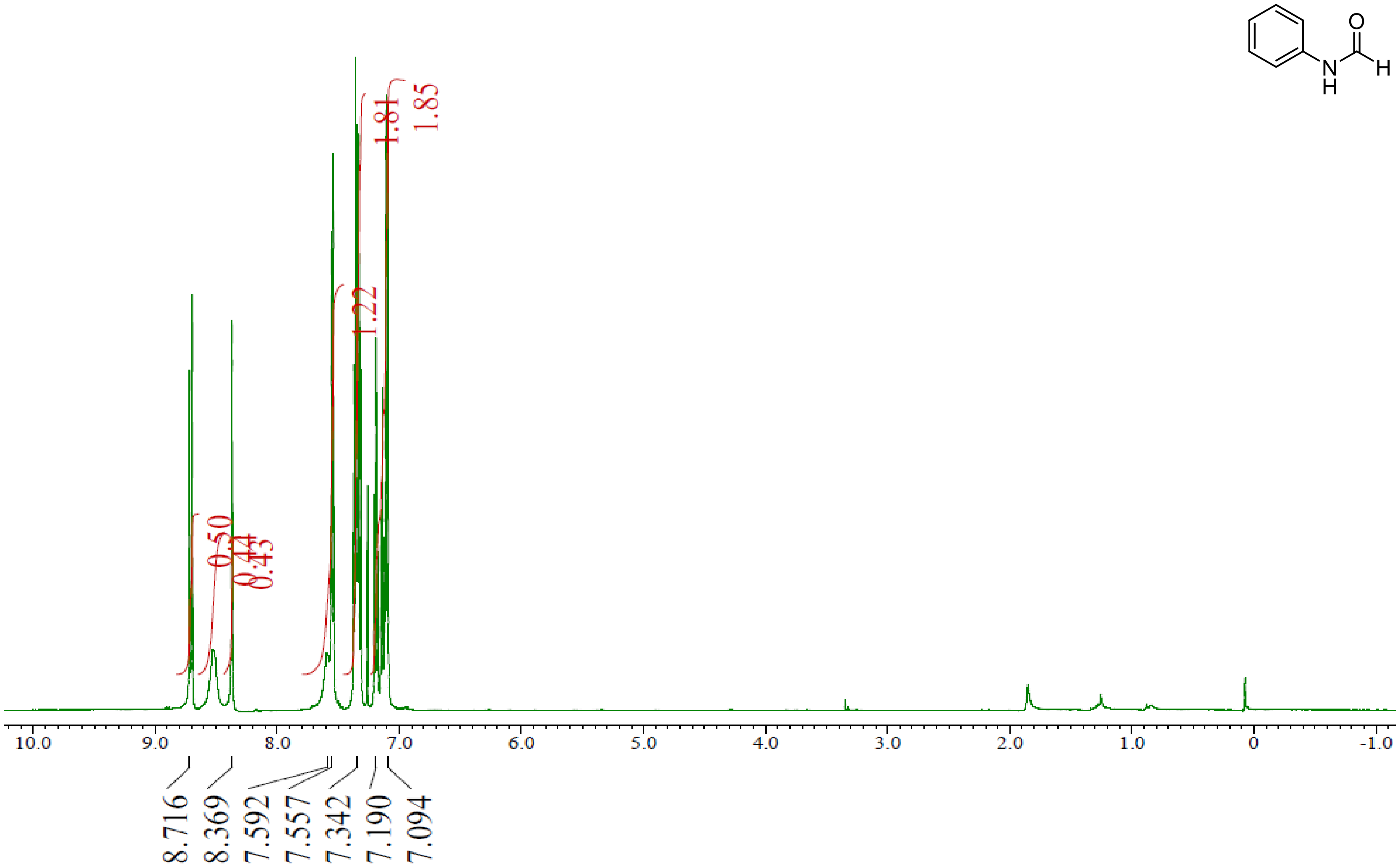

^{13}C NMR spectrum (126 MHz, $CDCl_3$) of formanilide (**2a**). Two tautomers were observed in approximately 0.5:0.5 ratio.

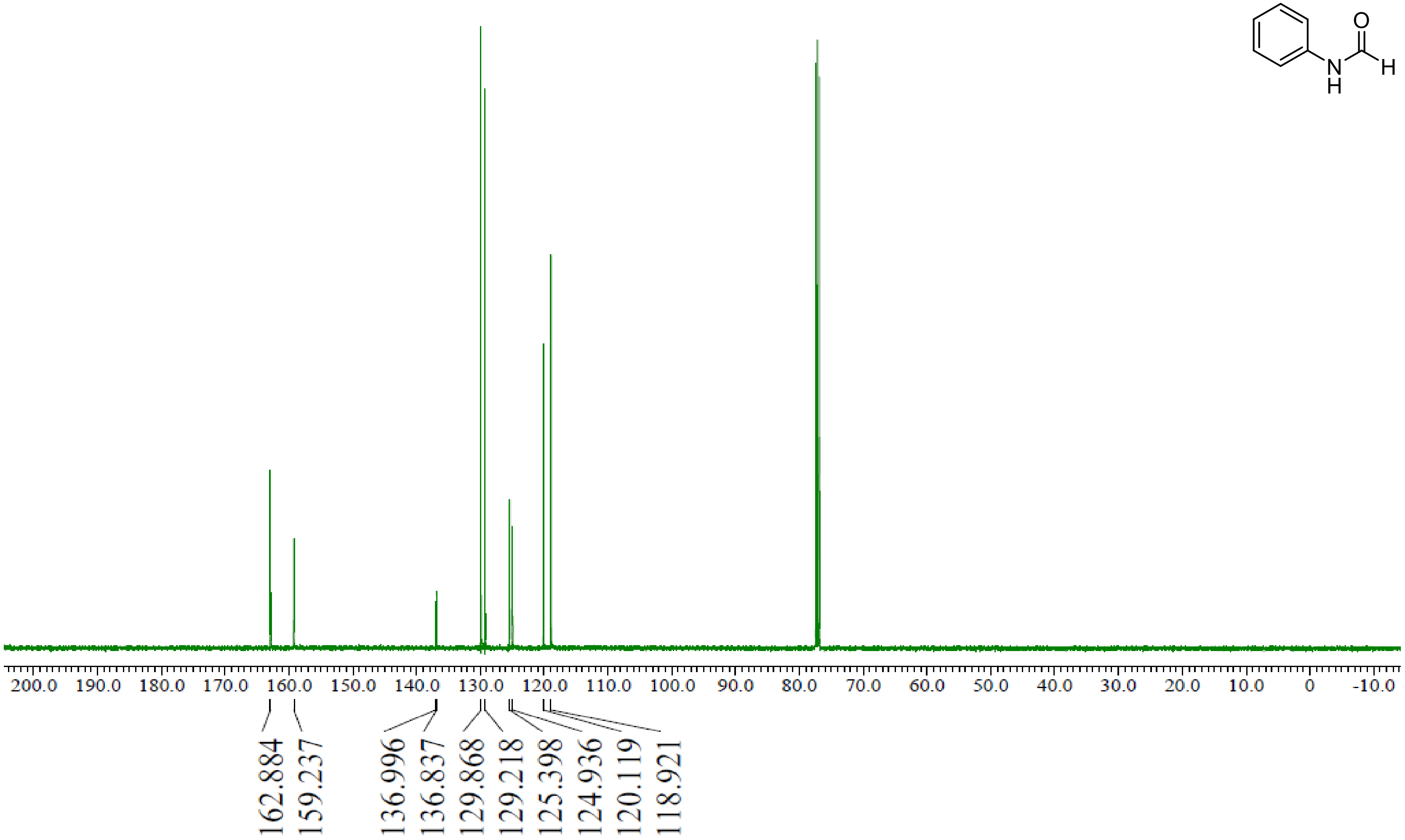

^{1}H NMR spectrum (500 MHz, $CDCl_3$) of *N*-methylbenzenaminium chloride (**3o•HCl**) (× impurity: **3a•HCl**).

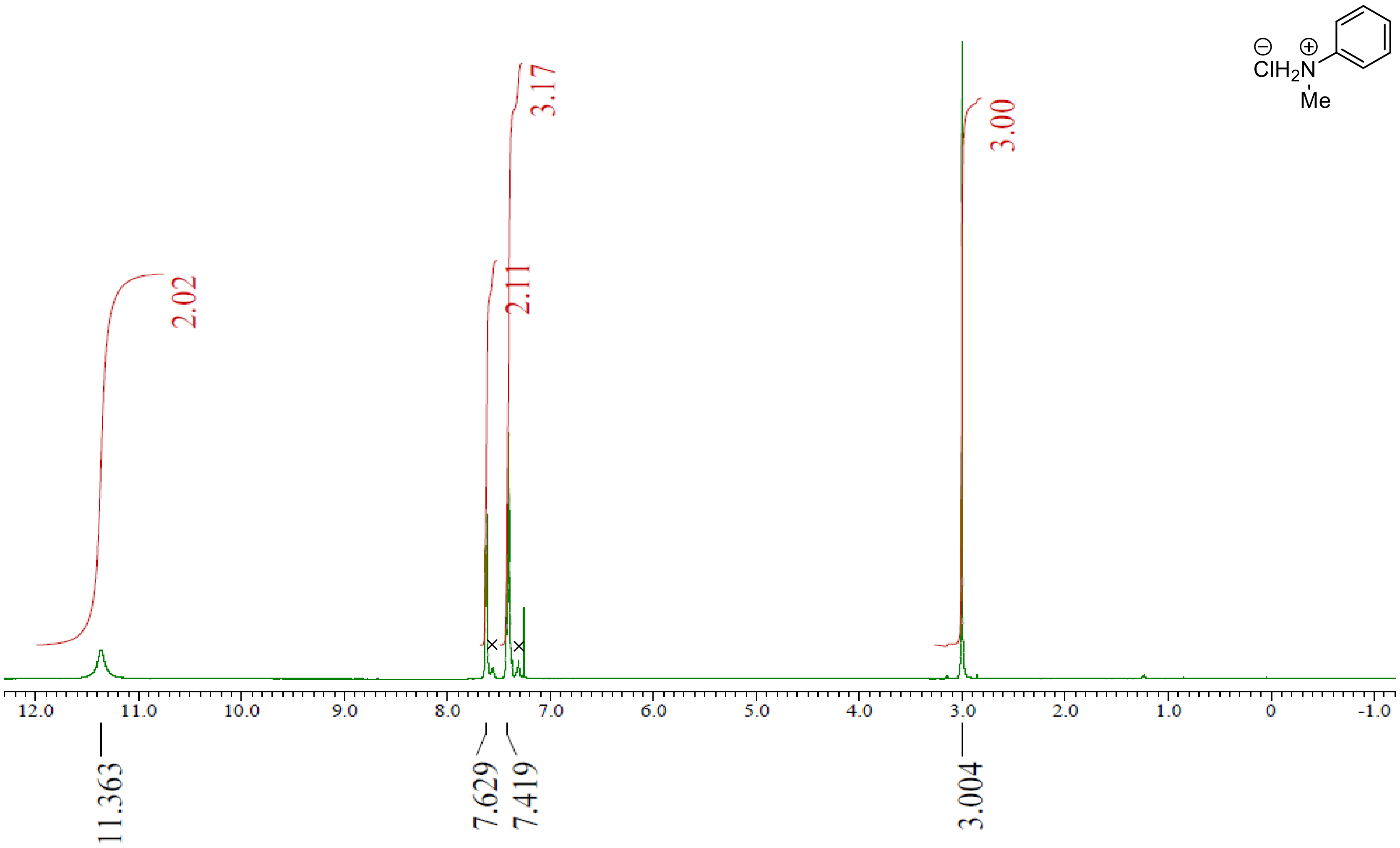

^{13}C NMR spectrum (126 MHz, $CDCl_3$) of *N*-methylbenzenaminium chloride (**3o•HCl**) (× impurity: **3a•HCl**).

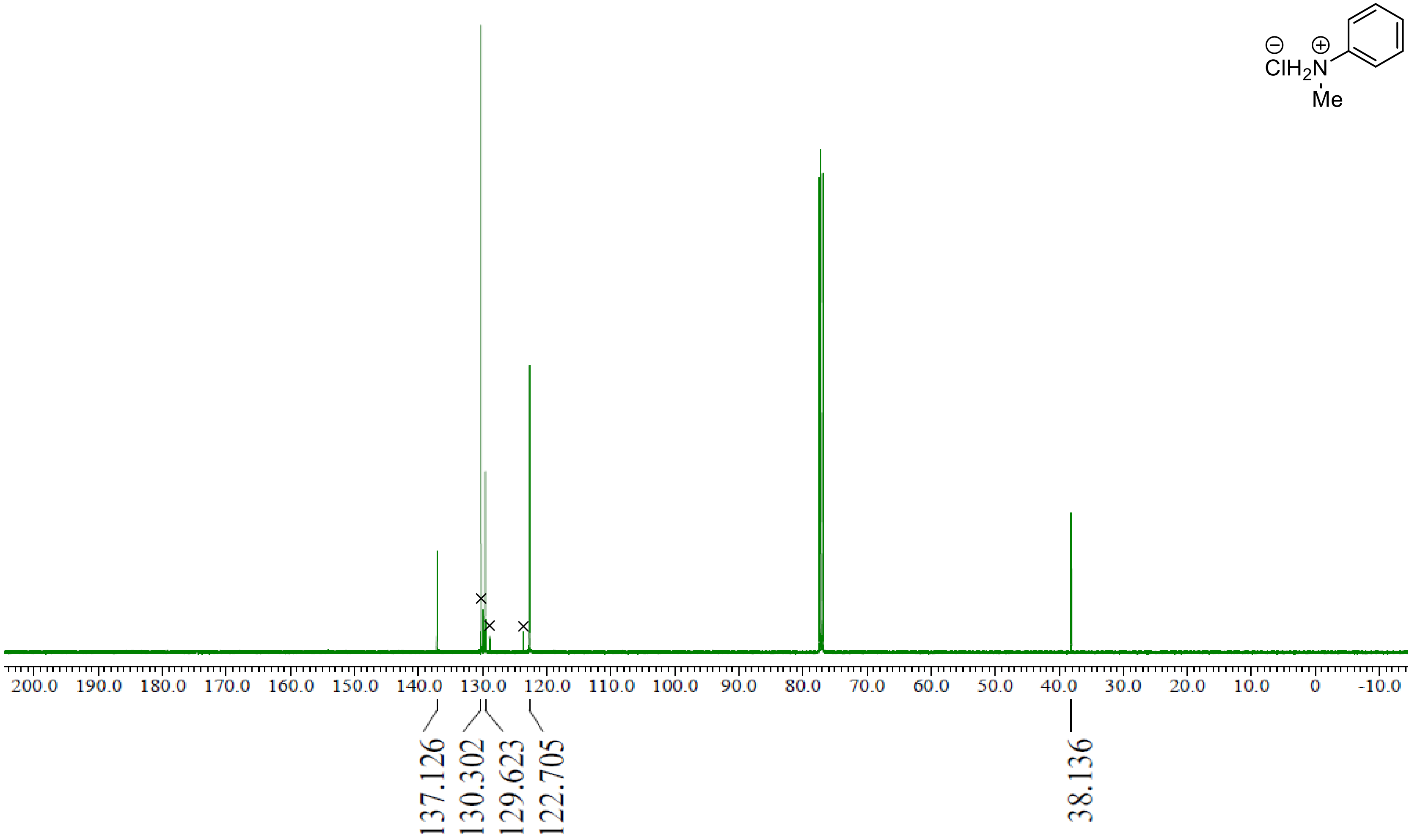

^{1}H NMR spectrum (500 MHz, DMSO-d_6) of *N*,*N'*-(methylenebis(4,1-phenylene))diformamide (**18**). Two tautomers were observed in approximately 0.25:0.75 ratio.

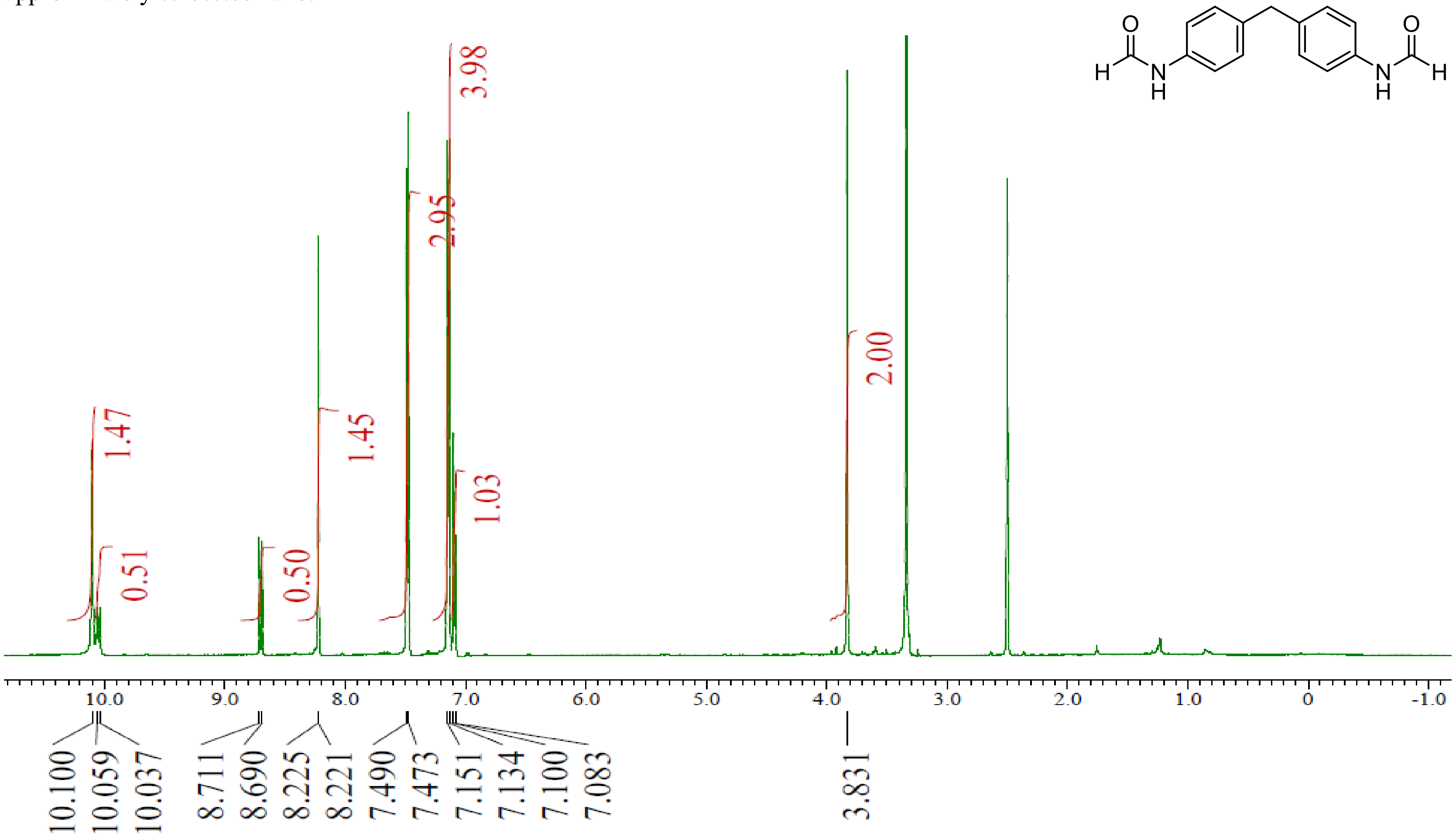

^{13}C NMR spectrum (126 MHz, DMSO-d_6) of *N*,*N'*-(methylenebis(4,1-phenylene))diformamide (**18**). Two tautomers were observed in approximately 0.25:0.75 ratio.

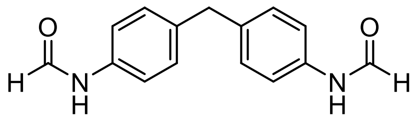

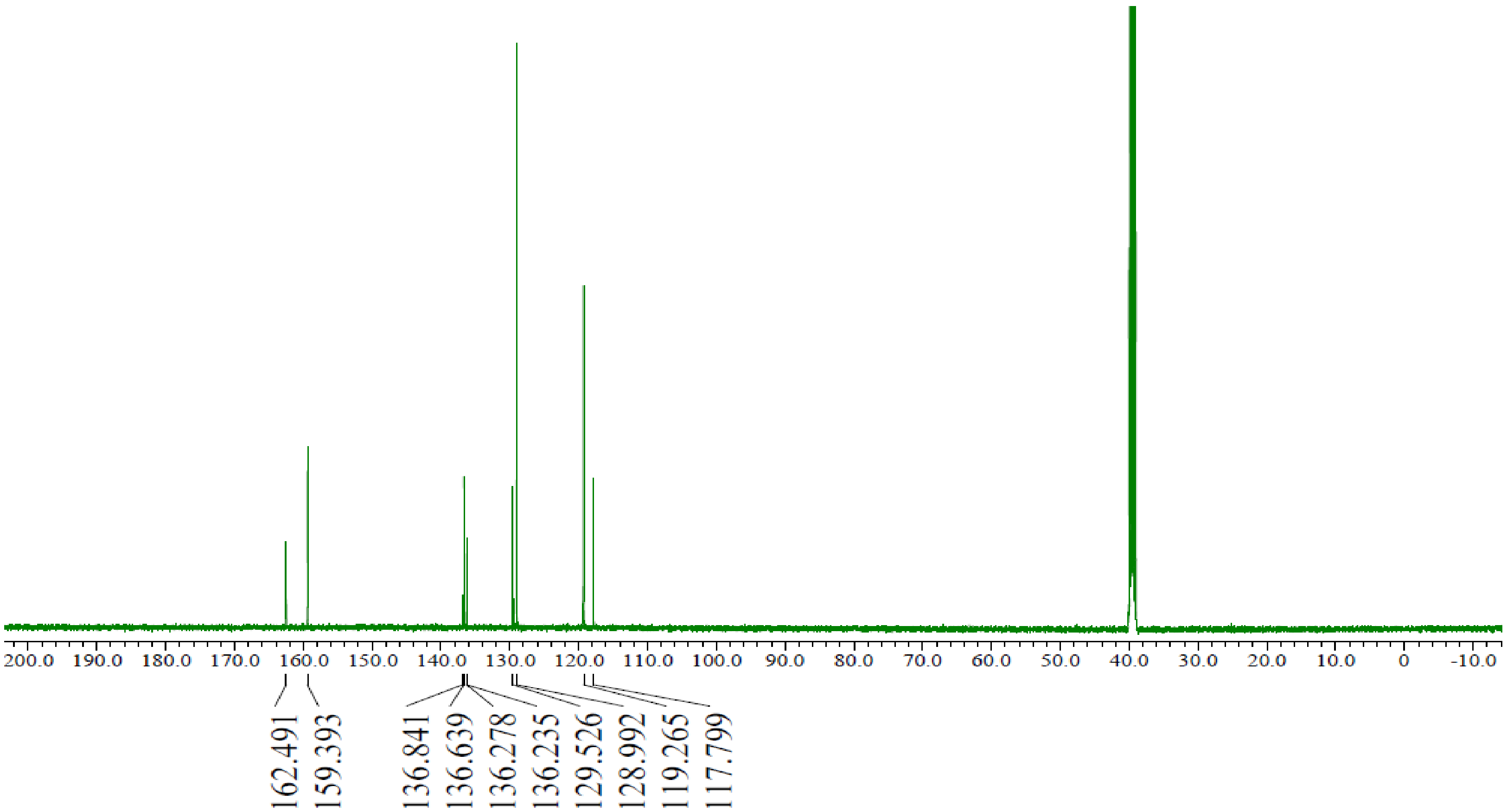

^{1}H NMR spectrum (500 MHz, CDCl3) of *N*,*N*'-dimethyl-1,6-diaminohexane (**16**).

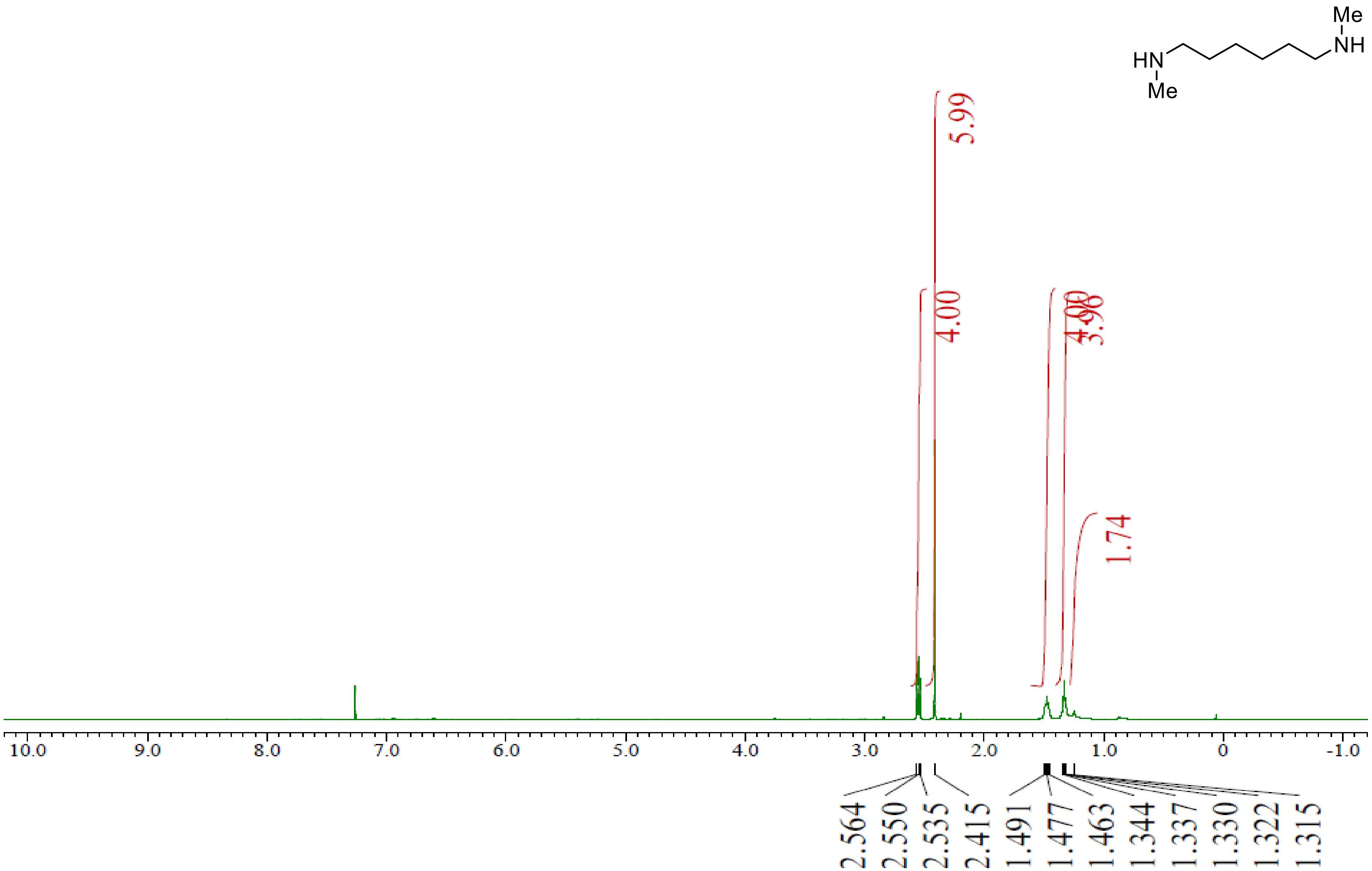

^{13}C NMR spectrum (126 MHz, $CDCl_3$) of *N*,*N'*-dimethyl-1,6-diaminohexane (**16**).

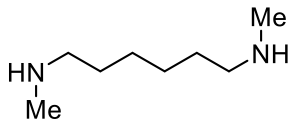

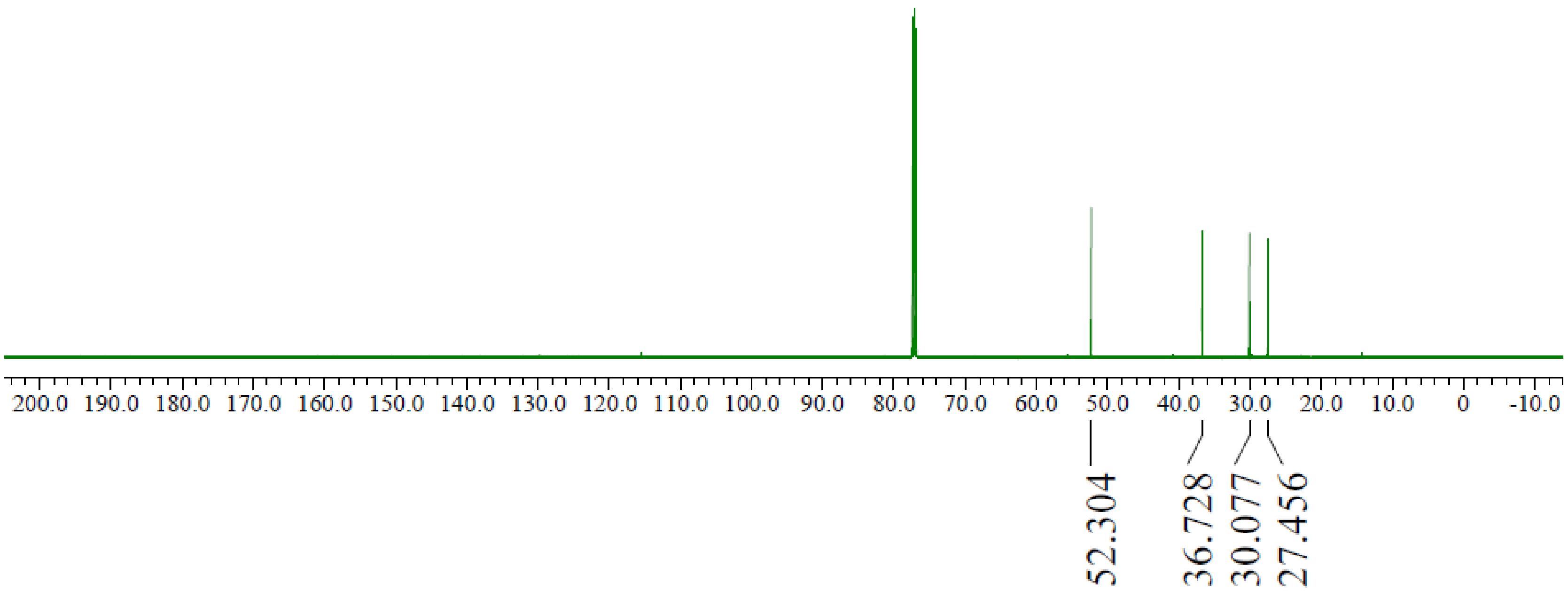

10. Computational Studies

Density function theory (DFT) calculations were performed with the M06 functional[75] as implemented in the Gaussian 16 suite of programs[76]. Molecular structures were optimized at M06/Def2TZVP level of theory with the SMD[77] model (THF). NBO analyses, shown in Section 1-8, were performed at M06/Def2TZVP with the SMD model (THF) using the NBO6 program[78]. CYLview[79] was used for visualization of the theoretical calculations.

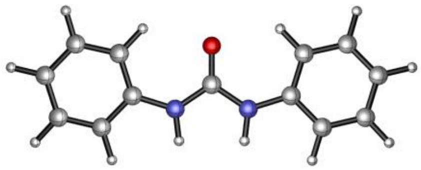

1a

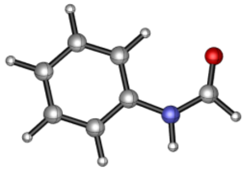

2a

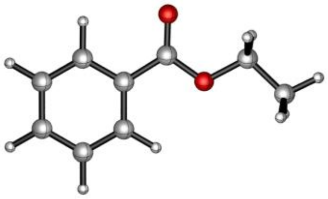

13

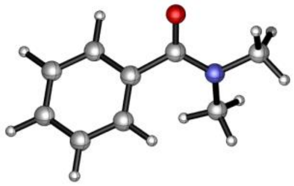

1k'

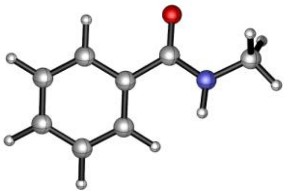

1k"

Supplementary Fig 114.

Optimized structures of urea **1a**, formanilide (**2a**), ethyl benzoate (**13**), and benzamides (**1k'** and **1k"**). Carbon (gray), nitrogen (blue), oxygen (red), and hydrogen (white).

Cartesian coordinates (x, y, z) of optimized structures

28

1a, electronic energy = -687.147534 a.u.

C	0.000000	0.058300	0.000000
N	-1.139400	-0.712700	-0.022900
H	-1.021000	-1.713300	-0.091800
N	1.139400	-0.712800	0.022700
H	1.021000	-1.713300	0.091500
O	0.000000	1.270900	0.000200
C	-2.472700	-0.296000	-0.009100
C	-2.881400	1.025500	0.155900
C	-3.440700	-1.290300	-0.156700
C	-4.233900	1.326100	0.169700
H	-2.143500	1.804900	0.270100
C	-4.784300	-0.975300	-0.138900
H	-3.122600	-2.320500	-0.286500
C	-5.193400	0.339400	0.024200
H	-4.536900	2.359400	0.299500
H	-5.517500	-1.765700	-0.254800
H	-6.247600	0.589700	0.037800
C	2.472700	-0.296000	0.009000
C	2.881400	1.025400	-0.156100

C 3.440700 -1.290300 0.156800
C 4.233900 1.326100 -0.169800
H 2.143500 1.804900 -0.270400
C 4.784300 -0.975300 0.139000
H 3.122600 -2.320500 0.286600
C 5.193400 0.339400 -0.024100
H 4.536800 2.359400 -0.299700
H 5.517500 -1.765600 0.255000
H 6.247600 0.589700 -0.037700

16
2a, electronic energy = -400.827471 a.u.
C 2.592200 -0.249900 0.000100
N 1.402500 -0.901800 0.000200
H 1.468900 -1.910400 0.000300
O 2.766000 0.945900 -0.000500
C 0.101200 -0.383300 0.000200
C -0.178600 0.980400 0.000300
C -0.950500 -1.297500 -0.000100
C -1.497100 1.405500 0.000100
H 0.632000 1.693900 0.000500
C -2.259600 -0.859000 -0.000200
H -0.728100 -2.360300 -0.000200
C -2.542500 0.498400 -0.000100
H -1.704400 2.469900 0.000200
H -3.065000 -1.584700 -0.000400
H -3.569400 0.844100 -0.000200
H 3.429400 -0.974500 0.000700

21
13, electronic energy = -499.302286 a.u.
C -2.288400 -1.571600 0.000000
C -0.973300 -1.143100 0.000000
C -0.684100 0.217700 0.000000
C -1.724700 1.140200 -0.000000
C -3.037800 0.709800 -0.000000
C -3.320900 -0.647300 -0.000000
H -2.509200 -2.632800 0.000000
H -0.167200 -1.865600 0.000100
H -1.487500 2.197500 -0.000000
H -3.844500 1.433700 -0.000100
H -4.350700 -0.986100 -0.000000
C 0.707000 0.734600 0.000000
O 0.991800 1.905400 0.000000
C 2.990000 0.162400 0.000000
H 3.177600 0.781900 0.881500
H 3.177600 0.782000 -0.881500
C 3.831000 -1.077100 -0.000000
H 3.635800 -1.685000 -0.885700
H 4.888000 -0.805700 -0.000000
H 3.635800 -1.685100 0.885600

O 1.614500 -0.241600 0.000000

22
1k’, electronic energy = -479.409576 a.u.

C	-2.293800	1.192100	-0.617900
C	-0.937400	0.917900	-0.657700
C	-0.440000	-0.244900	-0.081000
C	-1.318600	-1.131000	0.530400
C	-2.670700	-0.845100	0.591300
C	-3.161100	0.315900	0.014300
H	-2.675300	2.093400	-1.083900
H	-0.261000	1.604900	-1.155700
H	-0.930800	-2.049100	0.957600
H	-3.346800	-1.535200	1.083200
H	-4.222100	0.535000	0.052100
C	0.998300	-0.637300	-0.202300
O	1.285700	-1.758900	-0.591900
C	3.330700	-0.022700	-0.158600
H	3.411300	-0.763800	-0.950300
H	3.817100	-0.418000	0.740400
C	1.728500	1.493400	0.865100
H	1.932300	2.391700	0.273400
H	0.710900	1.545400	1.243400
H	2.408100	1.493900	1.723400
N	1.942600	0.293900	0.087100
H	3.856600	0.886500	-0.459400

19
1k”, electronic energy = -440.127819 a.u.

C	-2.158700	-1.346100	-0.143700
C	-0.787400	-1.163000	-0.190700
C	-0.241700	0.107600	-0.040000
C	-1.093000	1.190300	0.149000
C	-2.462100	1.006500	0.206900
C	-2.998000	-0.263400	0.060600
H	-2.573600	-2.339100	-0.273600
H	-0.148400	-2.019600	-0.376200
H	-0.661000	2.179000	0.250900
H	-3.115400	1.857300	0.363800
H	-4.071500	-0.408900	0.099900
C	1.227400	0.379000	-0.099300
O	1.661800	1.482500	-0.387900
C	3.474300	-0.507500	0.174900
H	3.812300	0.194600	0.942700
H	3.819100	-0.136000	-0.792100
N	2.041800	-0.659500	0.182500
H	3.934300	-1.476500	0.360300
H	1.652400	-1.514600	0.543000

11. References

1 Khusnutdinova, J. R. & Milstein, D. Metal–ligand cooperation. *Angew. Chem. Int. Ed.* **54**, 12236–12273 (2015).

2 Higashi, T., Kusumoto, S. & Nozaki, K. Cleavage of Si–H, B–H, and C–H bonds by metal–ligand cooperation. *Chem. Rev.* **119**, 10393–10402 (2019).

3 Jessop, P. G. & Morris, R. H. Reaction of transition metal dihydrogen complexes. *Coord. Chem. Rev.* **121**, 155–284 (1992).

4 Glueck, D. S., Winslow, L. J. N. & Bergman, R. G. Iridium alkoxide and amide hydride complexes. Synthesis, reactivity, and the mechanism of O-H and N-H reductive elimination. *Organometallics* **10**, 1462–1479 (1991).

5 Stradella, L. & Argentero, M. A DSC, TG, IR study of the thermal decomposition of some alkyl- and aryl-ureas. *Thermochim. Acta* **268**, 1–7 (1995).

6 Hatanaka, M. A reaction paths toward isocyanate adducts. *Bull. Chem. Soc. Jpn.* **82**, 1149–1151 (2009).

7 Balaraman, E., Ben-David, Y. & Milstein, D. Unprecedented catalytic hydrogenation of urea derivatives to amines and methanol. *Angew. Chem. Int. Ed.* **50**, 11702–11705 (2011).

8 Miura, T., Held, I. E., Oishi, S., Naruto, M. & Saito, S. Catalytic hydrogenation of unactivated amides enabled by hydrogenation of catalyst precursor. *Tetrahedron Lett.* **54**, 2674–2678 (2013).

9 vom Stein, T., Meuresch, M., Limper, D., Schmitz, M., Hölscher, M., Coetzee, J., Cole-Hamilton, D. J., Klankermayer, J. & Leitner, W. Highly versatile catalytic hydrogenation of carboxylic and carbonic acid derivatives using a Ru-Triphos complex: Molecular control over selectivity and substrate scope. *J. Am. Chem. Soc.* **136**, 13217–13225 (2014).

10 Kothandaraman, J., Kar, S., Sen, R., Goeppert, A., Olah, G. A. & Prakash, G. K. S. Efficient reversible hydrogen carrier system based on amine reforming of methanol. *J. Am. Chem. Soc.* **139**, 2549–2552 (2017).

11 Miura, T., Naruto, M., Toda, K., Shimomura, T. & Saito, S. Multifaceted catalytic hydrogenation of amides via diverse activation of a sterically confined bipyridine–ruthenium framework. *Sci. Rep.* **7**, 1586 (2017).

12 Xie, Y., Hu, P., Ben-David, Y. & Milstein, D. A reversible liquid organic hydrogen carrier system based on methanol-ethylenediamine and ethylene urea. *Angew. Chem. Int. Ed.* **58**, 5105–5109 (2019).

13 Liu, X., Zuo, Y., Kallmeier, F., Mejia, E., Tin, S., de Vries, J. G. & Baráth, E. Hydrogenative depolymerization of silicon-modified polyureas. *Chem. Commun.* **58**, 5415–5418 (2022).

14 Das, U. K., Kumar, A., Ben-David, Y., Iron, M. A. & Milstein, D. Manganese catalyzed hydrogenation of carbamates and urea derivatives. *J. Am. Chem. Soc.* **141**, 12962–12966 (2019).

15 Liu, X., & Werner, T. Indirect reduction of CO_2 and recycling of polymers by manganese-catalyzed transfer hydrogenation of amides, carbamates, urea derivatives, and polyurethanes. *Chem. Sci.* **12**, 10590–10597 (2021).

16 Kumar, A. & Luk, J. Catalytic hydrogenation of urea derivatives and polyureas. *Eur. J. Org. Chem.* **2021**, 4546–4550 (2021).

17 Smidt, S. P., Pfaltz, A., Martínez-Viviente, E., Pregosin, P. S. & Albinati, A. X-ray and NOE studies on trinuclear iridium hydride phosphino oxazoline (PHOX) complexes. *Organometallics* **22**, 1000–1009 (2003).

18 Mazet, C., Smidt, S. P., Meuwly, M. & Pfaltz, A. A combined experimental and computational study of dihydrido(phosphinooxazoline)iridium complexes. *J. Am. Chem. Soc.* **126**, 14176–14181 (2004).

19 Wuts, P. G. M. *Green's protective Groups in Organic Synthesis. Fifth edition* (Wiley, 2014).

20 Shojaei, B., Najafi, M., Yazdanbakhsh, A., Abtahi, M. & Zhang, C. A review on the applications of polyurea in the construction industry. *Polym. Adv. Technol.* **32**, 2797–2812 (2021).

21 Yang, H., Du, G., Li, Z., Ran, X., Zhou, X., Li, T., Gao, W., Li, J., Lei, H. & Yang, L. Superstrong adhesive of isocyanate-free polyurea with a branched structure. *ACS Appl. Polym. Mater.* **3**, 1638−1651 (2021).

22 Chen, M., Feng, X., Xu, W., Wang, Y., Yang, Y., Jiang, Z. & Ding, J. PEGylated polyurea bearing hindered urea bond for drug delivery. *Molecules* **24**, 1538 (2019).

23 Grand view research, Polyurea Market Worth $1,404.9 Million By 2030 | CAGR: 6.1%. https://www.grandviewresearch.com/press-release/global-polyurea-market (2023).

24 Zhou, W., Neumann, P., Al Batal, M., Rominger, F., Hashmi, A. S. K. & Schaub, T. Depolymerization of technical-grade polyamide 66 and polyurethane materials through hydrogenation. *ChemSusChem* **14**, 4176–4180 (2021).

25 Zubar, V., Haedler, A. T., Schütte, M., Hashmi, A. S, K. & Schaub, T. Hydrogenative depolymerization of polyurethanes catalyzed by a manganese pincer complex. *ChemSusChem* **15**, e202101606 (2022).

26 Motokucho, S., Matsumoto, T., Nakayama, Y., Horiuchi, R., Morikawa, H. & Nakatani, H. Hydrolysis of polyurea under high pressure of carbon dioxide. *Polym. Bull.* **74**, 615–623 (2017).

27 Shang, J., Liu, S., Ma, X., Lu, L. & Deng, Y. A new route of CO_2 catalytic activation: Syntheses of N-substituted carbamates from dialkyl carbonates and polyureas. *Green Chem.* **14**, 2899–2906 (2012).

28 Li, Q., Wang, P., Liu, S., Fei, Y. & Deng, Y. Catalytic degradation of polyurea: Synthesis of N-substituted carbamates with CuO–ZnO as the catalyst. *Green Chem.* **18**, 6091–6098 (2016).

29 Langsted, C. R., Paulson, S. W., Bomann, B. H., Suhail, S., Aguirre, J. A., Saumer, E. J., Baclasky, A. R., Salmon, K. H., Law, A. C., Farmer, R. J., Furchtenicht, C. J., Stankowski, D. S., Johnson, M. L., Corcoran, L. G., Dolan, C. C., Carney, M. J. & Robertson, N. J. Isocyanate-free synthesis of ureas and

polyureas via ruthenium catalyzed dehydrogenation of amines and formamides. *J. Appl. Polym. Sci.* **139**, 52088 (2022).

30 Kotachi, S., Tsuji, Y., Kondo, T. & Watanabe, Y. Ruthenium catalysed N,N'-diarylurea synthesis from N-aryl substituted formamides and aminoarenes. *J. Chem. Soc., Chem. Commun.* 549−550 (1990).

31 Kondo, T., Kotachi, S., Tsuji, Y., Watanabe, Y. & Mitsudo, T. Novel ruthenium-complex-catalyzed synthesis of ureas from formamides and amines. *Organometallics* **16**, 2562−2570 (1997).

32 Lane, E. M., Hazari, N. & Bernskoetter, W. H. Iron-catalyzed urea synthesis: Dehydrogenative coupling of methanol and amines. *Chem. Sci.* **9**, 4003–4008 (2018).

33 Bruffaerts, J., von Wolff, N., Diskin-Posner, Y., Ben-David, Y. & Milstein, D. Formamides as isocyanate surrogates: A mechanistically driven approach to the development of atom-efficient, selective catalytic syntheses of ureas, carbamates, and heterocycles. *J. Am. Chem. Soc.* **141**, 16486−16493 (2019).

34 Owen, A. E., Preiss, A., McLuskie, A., Gao, C., Peters, G., Bühl, M. & Kumar, A. Manganese-catalyzed dehydrogenative synthesis of urea derivatives and polyureas. *ACS Catal.* **12**, 6923−6933 (2022).

35 Kim, S. H. & Hong, S. H. Ruthenium-catalyzed urea synthesis using methanol as the C1 source. *Org. Lett.* **18**, 212−215 (2016).

36 Krishnakumar, V., Chatterjee, B. & Gunanathan, C. Ruthenium-catalyzed urea synthesis by N−H activation of amines. *Inorg. Chem.* **56**, 7278−7284 (2017).

37 Kumar, A., Armstrong, D., Peters, G., Nagala, M. & Shirran, S. Direct synthesis of polyureas from the dehydrogenative coupling of diamines and methanol. *Chem. Commun.* **57**, 6153–6156 (2021).

38 Pangborn, A. B., Giardello, M. A., Grubbs, R. H., Rosen, R. K. & Timmers, F. J. Safe and convenient procedure for solvent purification. *Organometallics* **15**, 1518–1520 (1996).

39 Crabtree, R. H., Quirk, J. M., Felkin, H. & Fillebeen-khan, T. An efficient synthesis of $[Ir(cod)Cl]_2$ and its reaction with PMe_2Ph to give *fac*-$[IrH(PMe_2C_6H_4)(PMe_2Ph)_3]$. *Synth. React. Inorg. Met.-Org. Chem.* **12**, 407–413 (1982).

40 Baillie, C. & Xiao, J. Palladium-catalysed synthesis of biaryl phosphines. *Tetrahedron* **60**, 4159–4168 (2004).

41 Ma, S. S., Sun, R., Zhang, Z. H., Yu, Z. K. & Xu, B. H. Ruthenium-catalysed chemoselective alkylation of nitroarenes with alkanols. *Org. Chem. Front.* **8**, 6710–6719 (2021).

42 Perveen, S., Yasmin, A. & Khan, K. M. Effect of successive increase in alcohol chains on reaction with isocyanates and isothiocyanates. *Nat. Prod. Res.* **24**, 18–23 (2010).

43 Woodmansee, D. H., Müller, M.-A., Neuburger, M. & Pfaltz, A. Chiral pyridyl phosphinites with large aryl substituents as efficient ligands for the asymmetric iridium-catalyzed hydrogenation of difficult substrates. *Chem. Sci.* **1**, 72–78 (2010).

44 Hussong, C., Langanke, J. & Leitner, W. A green route to polyurethanes: Oxidative carbonylation of industrially relevant aromatic diamines by CO_2-based methyl formate. *Green Chem.* **22**, 8260–8270 (2020).

45 Burla, M. C., Caliandro, R., Camalli, M., Carrozzini, B., Cascarano, G. L., De Caro, L., Giacovazzo, C., Polidori, G., Siliqi, D. & Spagna, R. IL MILIONE: A suite of computer programs for crystal structure solution of proteins. *J. Appl. Cryst.* **40**, 609–613 (2007).

46 Sheldrick, G. M. A short history of SHELX. *Acta Cryst., Sect. A: Found. Cryst.* **64**, 112–122 (2008).

47 Dolomanov, O. V., Bourhis, L. J., Gildea, R. J., Howard, J. A. K. & Puschmann, H. OLEX2: A complete structure solution, refinement and analysis program. *J. Appl. Cryst.* **42**, 339–341 (2009).

48 Wang, M., Han, J., Si, X., Hu, T., Zhu, J. & Sun, X. Effective approach to ureas through organocatalyzed one-pot process. *Tetrahedron Lett.* **59**, 1614–1618 (2018).

49 Rekunge, D. S., Khatri, C. K. & Chaturbhuj, G. U. Sulfated polyborate-catalyzed efficient and expeditious synthesis of (un)symmetrical ureas and benzimidazolones. *Tetrahedron Lett.* **58**, 4304–4307 (2017).

50 Zhao, Y., Guo, X., Si, Z., Hu, Y., Sun, Y., Liu, Y., Ji, Z. & You, J. Hydrosilane-assisted synthesis of urea derivatives from CO_2 and amines. *J. Org. Chem.* **85**, 13347–13353 (2020).

51 Pfeifer, L., Engle, K. M., Pidgeon, G. W., Sparkes, H. A., Thompson, A. L., Brown, J. M. & Gouverneur, V. Hydrogen-bonded homoleptic fluoride–diarylurea complexes: Structure, reactivity, and coordinating power. *J. Am. Chem. Soc.* **138**, 13314–13325 (2016).

52 Park, J. H., Yoon, J. C. & Chung, Y. K. Cobalt/rhodium heterobimetallic nanoparticle-catalyzed oxidative carbonylation of amines in the presence of carbon monoxide and molecular oxygen to ureas. *Adv. Synth. Catal*. **351**, 1233–1237 (2009).

53 Zhu, X., Xu, M., Sun, J., Guo, D., Zhang, Y., Zhou, S. & Wang, S. Hydroamination and hydrophosphination of isocyanates/isothiocyanates under catalyst-free conditions. *Eur. J. Org. Chem.* **2021**, 5213–5218 (2021).

54 Li, J. S., Yang, P. P., Xie, X. Y., Jiang, S., Tao, L., Li, Z. W., Lu, C. H. & Liu, W. D. Catalyst-free electrosynthesis of benzimidazolones through intramolecular oxidative C−N coupling. *Adv. Synth. Catal*. **362**, 1977–1981 (2020).

55 Sanabria, C. M., do Casal, M. T., de Souza, R. B. A., de Aguiar, L. C. S. & de Mattos, M. C. S. Highly regioselective iodination of N-phenylureas with iodine/trichloroisocyanuric acid. *Synthesis* **49**, 1648–1654 (2017).

56 Zaragoza-Dörwald, F. & von Kiedrowski, G. A simple and economic synthesis of monoacylated alkanediamines by thermal transamidation. *Synthesis* **11**, 917–918 (1988).

57 Zhang, H., Liu, C., Yin, G., Du, C. & Zhang, B. Efficiently luminescent heteroleptic neutral

platinum(II) complexes based on N^O and N^P benzimidazole ligands. *Dalton Trans.* **50**, 17319–17327 (2021).

58 Labed, A., Jiang, F., Labed, I., Lator, A., Peters, M., Achard, M., Kabouche, A., Kabouche, Z., Sharma, G. V. M. & Bruneau, C. Iridium-catalyzed sustainable access to functionalized julolidines through hydrogen autotransfer. *ChemCatChem* **7**, 1090–1096 (2015).

59 Suchý, M., Elmehriki, A. A. H. & Hudson, R. H. E. A remarkably simple protocol for the N-formylation of amino acid esters and primary amines. *Org. Lett.* **13**, 3952–3955 (2011).

60 Tumma, H., Nagaraju, N. & Reddy, K. V. A facile method for the N-formylation of primary and secondary amines by liquid phase oxidation of methanol in the presence of hydrogen peroxide over basic copper hydroxyl salts. *J. Mol. Catal. A. Chem.* **310**, 121–129 (2009).

61 Kitamura, M., Suga, T., Chiba, S. & Narasaka, K. Synthesis of primary amines by the electrophilic amination of Grignard reagents with 1,3-dioxolan-2-one O-sulfonyloxime. *Org. Lett.* **6**, 4619–4621 (2004).

62 Guo, L., Chen, Z., Zhu, H., Li, M. & Gu, Y. Acid-catalyzed chemodivergent reactions of 2,2-dimethoxyacetaldehyde and anilines. *Chin. Chem. Lett.* **32**, 1419–1422 (2021).

63 Chen, X., Wang, H., Du, S., Driess, M. & Mo, Z. Deoxygenation of nitrous oxide and nitro compounds using bis(N-heterocyclic silylene)amido iron complexes as catalysts. *Angew. Chem. Int. Ed.* **61**, e202114598 (2022).

64 Shen, Q. & Hartwig, J. F. Palladium-catalyzed coupling of ammonia and lithium amide with aryl halides. *J. Am. Chem. Soc.* **128**, 10028–10029 (2006).

65 Timelthaler, D., Schofberger, W. & Topf, C. Selective and additive-free hydrogenation of nitroarenes mediated by a DMSO-tagged molecular cobalt corrole catalyst. *Eur. J. Org. Chem.* **2021**, 2114–2120 (2021).

66 Chen, J., Jia, J., Guo, Z., Zhang, J. & Xie, M. NH_4I-promoted N-acylation of amines *via* the transamidation of DMF and DMA under metal-free conditions. *Tetrahedron Lett.* **60**, 1426–1429 (2019).

67 Yin, J., Zhang, J., Cai, C., Deng, G. J. & Gong, H. Catalyst-free transamidation of aromatic amines with formamide derivatives and tertiary amides with aliphatic amines. *Org. Lett.* **21**, 387–392 (2019).

68 Hyodo, K., Hasegawa, G., Maki, H. & Uchida, K. Deacetylative amination of acetyl arenes and alkanes with C–C bond cleavage. *Org. Lett.* **21**, 2818–2822 (2019).

69 Gim, H. J., Kang, B. & Jeon, R. Synthesis and biological activity of 5-(4-[2-(methyl-p-substituted phenylamino)ethoxy]benzyl)thiazolidine-2,4-diones. *Arch. Pharm. Res.* **30**, 1055–1061 (2007).

70 Romero, A. H. & Cerecetto, H. A common, facile and eco-friendly method for the reduction of nitroarenes, selective reduction of poly-nitroarenes and deoxygenation of N-oxide containing

heteroarenes using elemental sulfur. *Eur. J. Org. Chem.* **2020**, 1853–1865 (2020).

71 Lin, W. C., Yatabe, T. & Yamaguchi, K. Selective primary aniline synthesis through supported Pd-catalyzed acceptorless dehydrogenative aromatization by utilizing hydrazine. *Chem. Commun.* **57**, 6530–6533 (2021).

72 Zhang, R., Zhang, J. C., Zhang, W. Y., He, Y. Q., Cheng, H., Chen, C. & Gu, Y. C. A practical approach for the transamidation of N,N-dimethyl amides with primary amines promoted by sodium tert-butoxide under solvent-free conditions. *Synthesis* **52**, 3286–3294 (2020).

73 Nixon, T. D., Whittlesey, M. K. & Williams, J. M. J. Ruthenium-catalysed transfer hydrogenation reactions with dimethylamine borane. *Tetrahedron Lett.* **52**, 6652–6654 (2011).

74 Bugarin, A., Jones, K. D. & Connell, B. T. Efficient, direct α-methylenation of carbonyls mediated by diisopropylammonium trifluoroacetate. *Chem. Commun.* **46**, 1715–1717 (2010).

75 Zhao, Y. & Truhlar, D. G. The M06 suite of density functionals for main group thermochemistry, thermochemical kinetics, noncovalent interactions, excited states, and transition elements: Two new functionals and systematic testing of four M06-class functionals and 12 other functionals. *Theor. Chem. Acc.* **120**, 215–241 (2008).

76 Frisch, M. J., Trucks, G. W., Schlegel, H. B., Scuseria, G. E., Robb, M. A., Cheeseman, J. R., Scalmani, G., Barone, V., Petersson, G. A., Nakatsuji, H., Li, X., Caricato, M., Marenich, A. V., Bloino, J., Janesko, B. G., Gomperts, R., Mennucci, B., Hratchian, H. P., Ortiz, J. V., Izmaylov, A. F., Sonnenberg, J. L., Williams-Young, D., Ding, F., Lipparini, F., Egidi, F., Goings, J., Peng, B., Petrone, A., Henderson, T., Ranasinghe, D., Zakrzewski, V. G., Gao, J., Rega, N., Zheng, G., Liang, W., Hada, M., Ehara, M., Toyota, K., Fukuda, R., Hasegawa, J., Ishida, M., Nakajima, T., Honda, Y., Kitao, O., Nakai, H., Vreven, T., Throssell, K., Montgomery, Jr., J. A., Peralta, J. E., Ogliaro, F., Bearpark, M. J., Heyd, J. J., Brothers, E. N., Kudin, K. N., Staroverov, V. N., Keith, T. A., Kobayashi, R., Normand, J., Raghavachari, K., Rendell, A. P., Burant, J. C., Iyengar, S. S., Tomasi, J., Cossi, M., Millam, J. M., Klene, M., Adamo, C., Cammi, R., Ochterski, J. W., Martin, R. L., Morokuma, K., Farkas, O., Foresman, J. B. & Fox, D. J. *Gaussian 16, revision C.01* (Gaussian, Inc., 2019).

77 Marenich, A. V., Cramer, C. J. & Truhlar, D. G. Universal solvation model based on solute electron density and on a continuum model of the solvent defined by the bulk dielectric constant and atomic surface tensions. *J. Phys. Chem. B* **113**, 6378–6396 (2009).

78 Glendening, E. D., Badenhoop, J. K., Reed, A. E., Carpenter, J. E., Bohmann, J. A., Morales, C. M., Landis, C. R. & Weinhold, F. *NBO 6.0* (Theoretical Chemistry Institute, University of Wisconsin, Madison, 2013).

79 Legault, C. Y. *Cylview 1.0b* (Université de Sherbrooke, 2009).
